# Synthesis and diversification of SuFEx-active 2*H*-indazoles from azobenzenes and ethenesulfonyl fluoride

**DOI:** 10.1039/d6ra09632h

**Published:** 2026-10-19

**Authors:** Lingayya Rajaka, Hyosik Woo, Myunghwa Jung, Chanhee Jang, Seungwan Kim, Jihyo Lee, Saegun Kim, Sangil Han, Han Yong Bae, Sang Hoon Han

**Affiliations:** a College of Pharmacy, Korea University Sejong 30019 Republic of Korea hans3@Korea.ac.kr; b Institute of Pharmaceutical Science and Translational Research, Korea University Sejong 30019 Republic of Korea; c Department of Innovative Pharmaceutical Sciences, Kyungpook National University Daegu 41566 Republic of Korea sangil.han@knu.ac.kr; d College of Pharmacy, Research Institute of Pharmaceutical Sciences, Sookmyung Women's University Seoul 04310 Republic of Korea; e Department of Chemistry, Sungkyunkwan University Suwon 16419 Republic of Korea hybae@skku.edu

## Abstract

A direct approach to SuFEx-active 2*H*-indazoles from azobenzenes and ethenesulfonyl fluoride was developed through Rh(iii)-catalyzed tandem C–H activation and annulation. The transformation provided a range of SO_2_F-containing 2*H*-indazoles with good functional group tolerance while preserving the synthetically valuable sulfonyl fluoride functionality. The practicality of the method was demonstrated by gram-scale synthesis and subsequent SuFEx conjugation with representative biologically relevant molecules. This strategy provides efficient access to functionalized 2*H*-indazoles bearing a versatile SuFEx handle for further molecular diversification in medicinal chemistry and chemical biology.

## Abstract 2

Rh(iii)-catalyzed annulation of azobenzenes with ESF enables direct access to SuFEx-active SO_2_F-containing 2*H*-indazoles.

## Graphical abstract

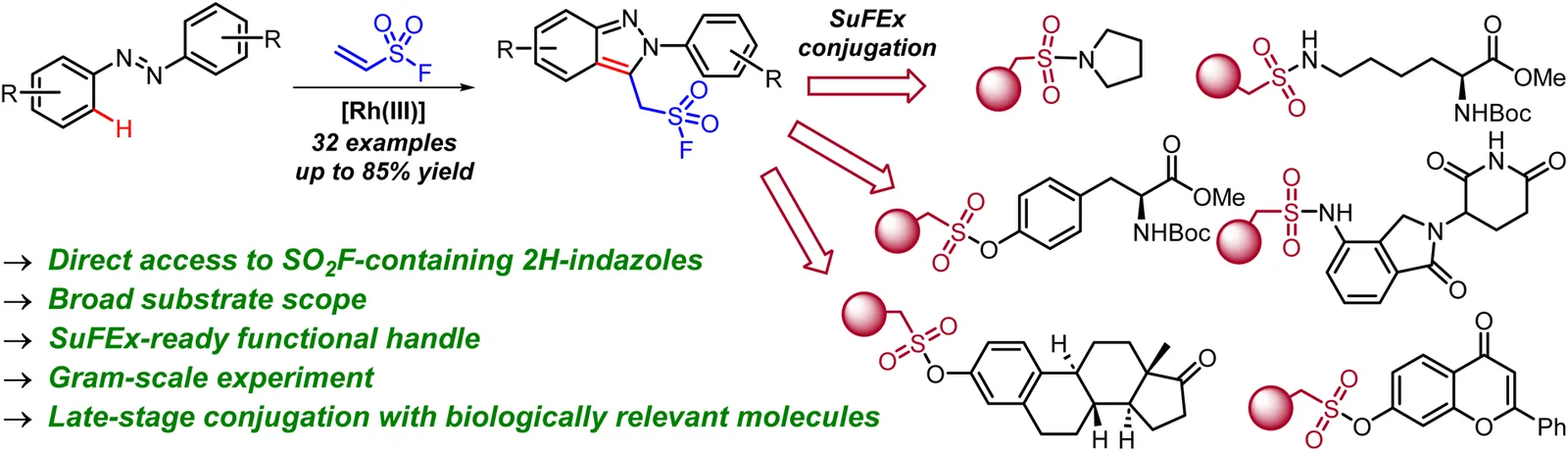

Nitrogen-containing heterocycles constitute fundamental structural motifs widely found in pharmaceuticals, agrochemicals, and functional materials, and their efficient synthesis remains a central objective in synthetic chemistry.^1^ Among them, indazole scaffolds, particularly 2*H*-indazoles, have attracted considerable attention owing to their broad spectrum of biological activities and frequent occurrence in bioactive molecules, including anti-inflammatory agents, anticancer compounds, and enzyme inhibitors (Scheme 1A).^2^ Consequently, the development of efficient and versatile synthetic strategies for indazole construction has been actively pursued, among which transition-metal-catalyzed C–H activation has emerged as a powerful approach for the rapid construction of molecular complexity through direct carbon–carbon bond formation and annulation processes.^3^ Notably, azobenzenes have proven to be valuable precursors for the synthesis of indazole derivatives, as their intrinsic azo (N=N) functionality can act as a directing group to facilitate site-selective C–C bond formation and subsequent cyclization.^4^ Indeed, structurally diverse indazoles have been accessed from azobenzenes *via* C–H functionalization strategies employing a range of coupling partners, including acrylates,^5^ aldehydes,^6^ alkynes,^7^ diazo compounds,^8^ sulfonium ylides,^9^ and vinylene carbonate,^10^ under transition metal catalysis (Scheme 1B). Beyond scaffold construction, strategies that simultaneously introduce a versatile functional handle for subsequent molecular diversification would further expand the synthetic and medicinal utility of these approaches.

**Scheme 1:**
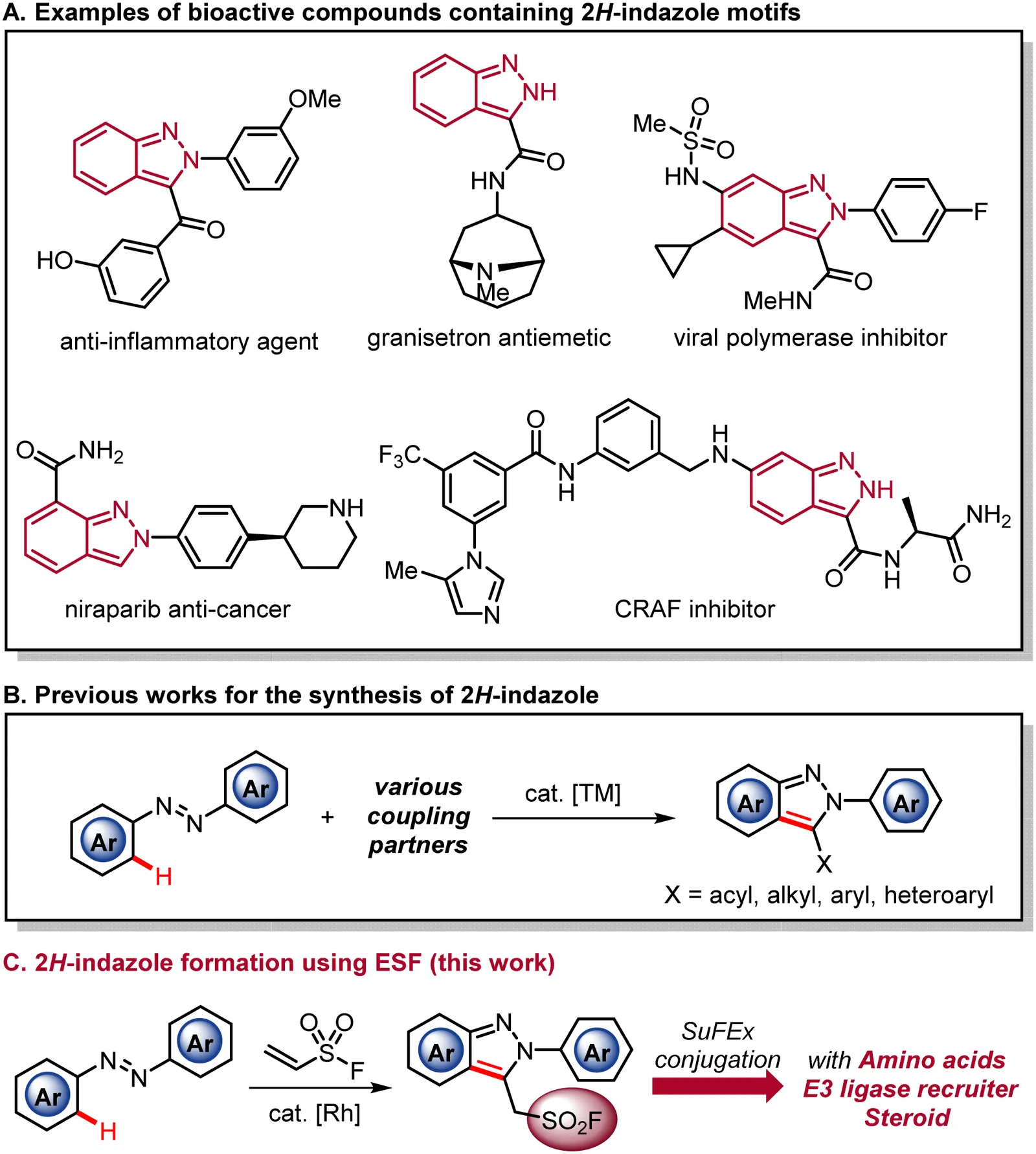
Access to SuFEx-active 2*H*-indazoles *via* catalytic C–H activation. (A) Examples of bioactive compounds containing 2*H*-indazole motifs; (B) previous approaches to the synthesis of 2*H*-indazoles; (C) 2*H*-indazole formation using ESF (this work).

Ethenesulfonyl fluoride (ESF) has attracted significant attention in recent years due to its unique reactivity and its ability to serve as a versatile synthetic linchpin.^11^ ESF combines an electron-deficient alkene that readily participates in diverse bond-forming reactions with a sulfonyl fluoride functional group that can be retained for subsequent sulfur(vi)-fluoride exchange (SuFEx) chemistry. In particular, SuFEx click chemistry has emerged as a powerful platform for bioconjugation, materials science, and chemical biology.^12^ Driven by these advantages, ESF has been employed in a variety of synthetic transformations.^13^ For example, various transformations based on photocatalysis,^14^ organocatalysis,^15^ and transition-metal catalysis^16^ have been reported, enabling efficient construction of C–C and C–heteroatom bonds while preserving the synthetically valuable sulfonyl fluoride functionality. Despite these advances, transition-metal-catalyzed C–H activation strategies involving ESF for the direct construction of SO_2_F-containing heterocyclic scaffolds remain comparatively rare.^17^ Although such transformations require careful control over the dual reactivity of ESF while preserving the SO_2_F functional group, they offer an attractive means of constructing heterocyclic SuFEx hubs that can undergo downstream molecular diversification.

Herein, we report efficient access to SuFEx-enabled indazole scaffolds from azobenzenes and ESF through Rh(iii)-catalyzed tandem C–H activation and cyclization. This protocol accommodates a broad range of mono- and disubstituted azobenzenes, providing structurally diverse SO_2_F-containing 2*H*-indazoles. The synthetic utility of this approach was further demonstrated by gram-scale synthesis and subsequent SuFEx conjugation with protected amino acid derivatives and biologically relevant molecules, including lenalidomide and estrone. These transformations demonstrate the potential of the resulting SO_2_F-containing 2*H*-indazoles as versatile SuFEx hubs for further molecular modification.

We began our investigation by examining the coupling reaction of azobenzene 1a with ethenesulfonyl fluoride (2a) under Rh(iii) catalysis, and the results are summarized in Table 1. Initial conditions using [RhCp*Cl_2_]_2_, AgSbF_6_, and Cu(OAc)_2_ in 1,4-dioxane at 60 °C afforded the desired annulated indazole 3a in 18% yield, without formation of the alkylated product 3aa (entry 1). Zn(OAc)_2_ or NaOAc was ineffective under the standard conditions (entries 2 and 3). The use of AgOAc gave 3a only in 9% yield (entry 4). Solvent screening revealed that THF provided 3a in 16% yield (entry 5), while changing the solvent to DCE significantly altered the reaction pathway, predominantly affording alkylated product 3aa in 82% yield along with 3a in 7% yield (entry 6). In contrast, toluene, DMF, and EtOH completely suppressed the reaction (entries 7–9). We next examined the effect of silver additives under otherwise identical conditions. AgTFA and AgOAc failed to promote the reaction (entries 10 and 11), whereas AgBF_4_ afforded 3a in 24% yield without detectable formation of 3aa (entry 12). Notably, AgOTf significantly improved the reaction efficiency, delivering 3a in 39% yield (entry 13). Further adjustment of the additive loading revealed that increasing the amount of AgOTf to 150 mol% substantially enhanced the yield, affording 3a in 80% yield in the presence of Cu(OAc)_2_ (25 mol%) (entry 14). Control experiments confirmed that the Rh(iii) catalyst was essential, as no reaction occurred in its absence (entry 15). In contrast, omission of Cu(OAc)_2_ resulted in incomplete consumption of the starting material and a diminished yield of 3a (entry 16). Finally, reducing the catalyst loading to 1 mol% still afforded 3a in 71% yield, with only a small amount of starting material remaining (entry 17).

**Table 1:** Selected optimization of reaction conditions^a^

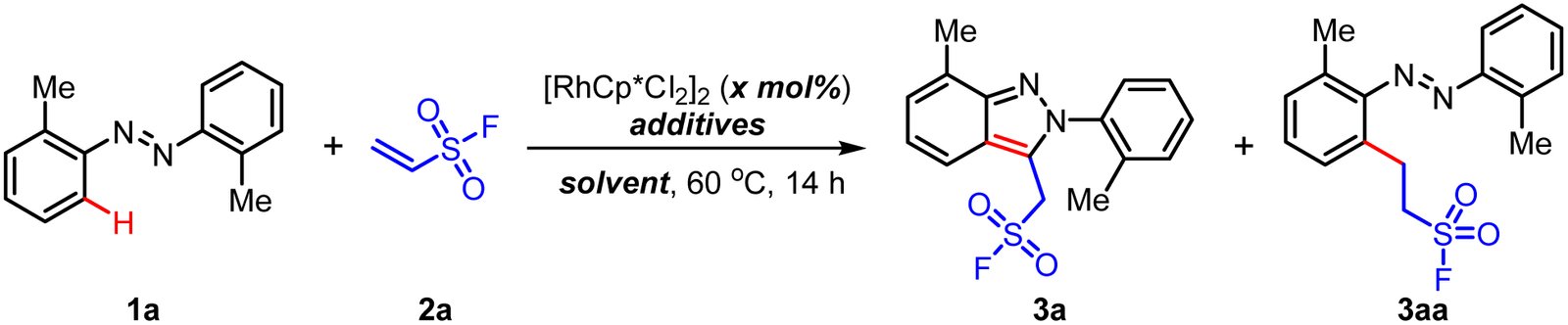				
Entry	Additives (mol%)	Solvent	Yield^b^	
			3a	3aa
1	AgSbF_6_ (10), Cu(OAc)_2_ (100)	1,4-Dioxane	18	0
2	AgSbF_6_ (10), Zn(OAc)_2_ (100)	1,4-Dioxane	N.R.	
3	AgSbF_6_ (10), NaOAc (100)	1,4-Dioxane	N.R.	
4	AgSbF_6_ (10), AgOAc (100)	1,4-Dioxane	9	0
5	AgSbF_6_ (10), Cu(OAc)_2_ (100)	THF	16	0
6	AgSbF_6_ (10), Cu(OAc)_2_ (100)	1,2-DCE	7	82
7	AgSbF_6_ (10), Cu(OAc)_2_ (100)	Toluene	N.R.	
8	AgSbF_6_ (10), Cu(OAc)_2_ (100)	DMF	N.R.	
9	AgSbF_6_ (10), Cu(OAc)_2_ (100)	EtOH	N.R.	
10	AgTFA (10), Cu(OAc)_2_ (100)	1,4-Dioxane	N.R.	
11	AgOAc (10), Cu(OAc)_2_ (100)	1,4-Dioxane	N.R.	
12	AgBF_4_ (10), Cu(OAc)_2_ (100)	1,4-Dioxane	24	0
13	AgOTf (10), Cu(OAc)_2_ (100)	1,4-Dioxane	39	0
**14**	**AgOTf (150), Cu(OAc)**_**2**_ **(25)**	**1,4-Dioxane**	**80**	**0**
15^c^	AgOTf (150), Cu(OAc)_2_ (25)	1,4-Dioxane	N.R.	
16	AgOTf (150)	1,4-Dioxane	48	0
17^d^	AgOTf (150), Cu(OAc)_2_ (25)	1,4-Dioxane	71	0

a Reaction conditions: 1a (0.2 mmol), 2a (0.6 mmol), [RhCp*Cl_2_]_2_ (2.5 mol%), additives (quantity noted), solvent (1 mL) at 60 °C for 14 h under air in a pressure tube.

b Isolated yield determined by flash column chromatography.

c This reaction was carried out in the absence of [RhCp*Cl_2_]_2_ catalyst.

d 1 mol% of [RhCp*Cl_2_]_2_ was used.

With the optimized conditions in hand, we next explored the scope of mono-substituted azobenzenes under the standard reaction conditions (Scheme 2). We first investigated *ortho*-substituted azobenzenes. Alkyl- and halogen-substituted substrates smoothly underwent annulation to furnish products 3a–3d in good yields. *Meta*-substituted azobenzenes were also compatible with the reaction conditions, affording products 3e–3i in 47–74% yields. We next examined *para*-substituted azobenzenes. A variety of electron-donating and electron-withdrawing substituents were compatible with the reaction conditions, furnishing products 3j–3p in 43–82% yields. Notably, the ketone-substituted substrate afforded 3o in the highest yield of the series.

**Scheme 2:**
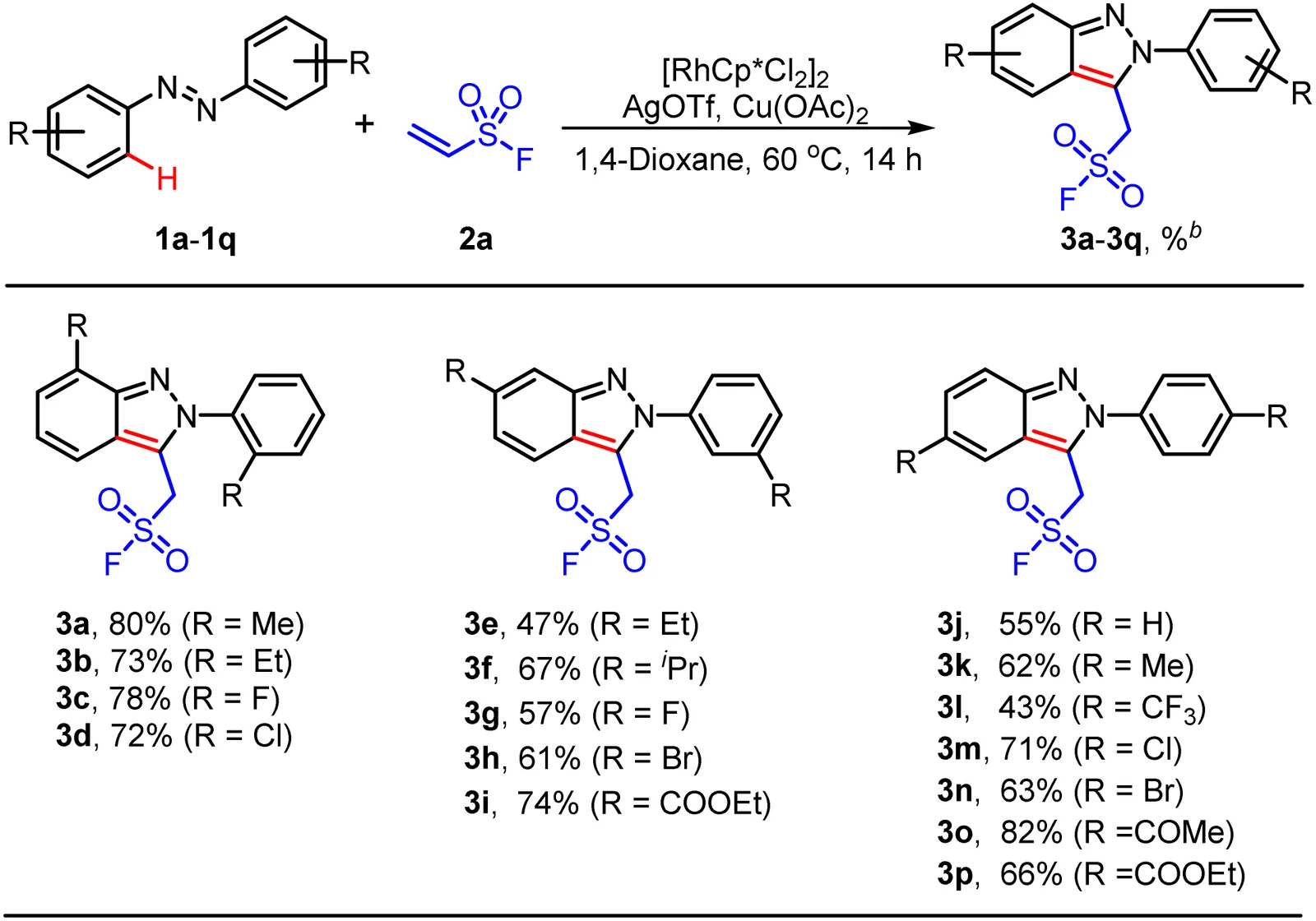
Scope of mono-substituted azobenzenes for synthesis of indazoles^*a*^. ^*a*^Reaction conditions: 1 (0.2 mmol), 2a (0.6 mmol), [RhCp*Cl_2_]_2_ (2.5 mol%), AgOTf (150 mol%), Cu(OAc)_2_ (25 mol%), 1,4-dioxane (1 mL) at 60 °C for 14 h under air in a pressure tube. ^*b*^Isolated yield determined by flash column chromatography.

To further evaluate the substrate scope of the annulation protocol, a series of di-substituted azobenzenes were subjected to the optimized conditions (Scheme 3). 2,3-Disubstituted azobenzenes proved to be suitable substrates, furnishing the corresponding products 5a–5d in moderate to good yields. The reaction was further extended to 2,4-disubstituted azobenzenes. Products 5e–5h were obtained in good to excellent yields, whereas bromine-containing substrate afforded 5i in only 21% yield. We next examined 2,5-disubstituted azobenzenes, which furnished products 5j and 5k in low yields. The relatively lower yields observed for these substrates may reflect the combined influence of steric and electronic effects associated with the substitution pattern. Furthermore, 3,4-disubstituted azobenzenes were also compatible with the reaction conditions, delivering products 5l and 5m in moderate yields. Finally, 3,5-disubstituted azobenzenes underwent the annulation reaction to furnish the corresponding products 5n–5p in yields comparable to those observed for the 2,5-disubstituted substrates. Collectively, these results demonstrate the broad substrate scope of the Rh(iii)-catalyzed annulation protocol for the synthesis of SO_2_F-containing indazoles.

**Scheme 3:**
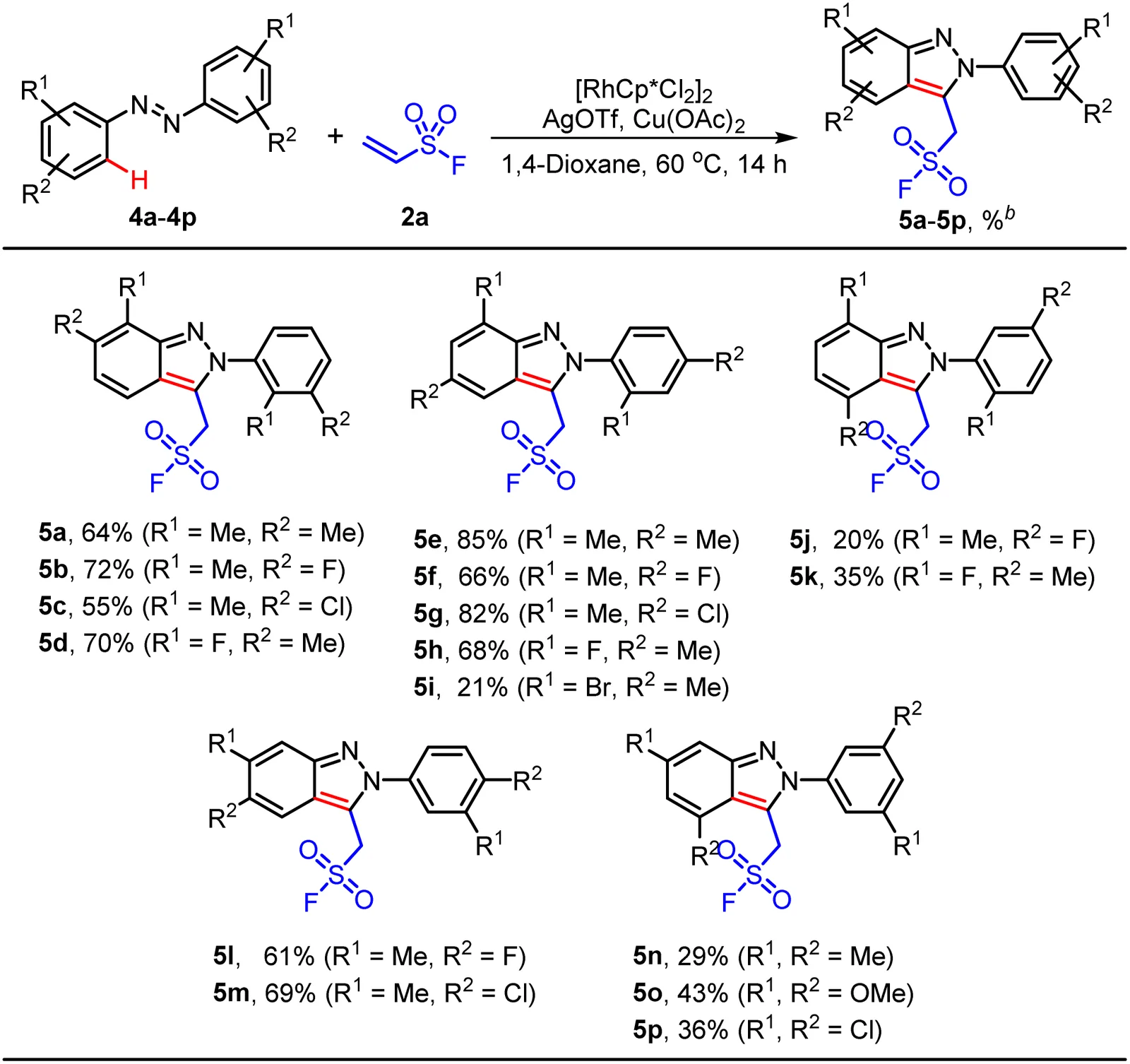
Scope of di-substituted azobenzenes for synthesis of indazoles^*a*^. ^*a*^Reaction conditions: 1 (0.2 mmol), 2a (0.6 mmol), [RhCp*Cl_2_]_2_ (2.5 mol%), AgOTf (150 mol%), Cu(OAc)_2_ (25 mol%), 1,4-dioxane (1 mL) at 60 °C for 14 h under air in a pressure tube. ^*b*^Isolated yield determined by flash column chromatography.

To examine the regioselectivity of the annulation, an unsymmetrically substituted azobenzene was subjected to the standard reaction conditions (Scheme 4a), affording regioisomeric indazoles 5q and 5q′ in 84% combined yield with a 5q : 5q′ ratio of 1.3 : 1. To gain further insight into the relative reactivity of differently substituted azobenzenes, intermolecular competition experiments were conducted under the optimized conditions (Scheme 4b and 4c). When equimolar amounts of 1k and 1m were subjected to the standard reaction conditions, the corresponding SO_2_F-containing indazoles 3k and 3m were obtained in 32% and 76% yields, respectively. Likewise, a competition experiment between 1k and 1p afforded the corresponding products 3k and 3p in 20% and 86% yields. These results suggest that the electronic nature of the azobenzene substrate influences its relative reactivity under optimized reaction conditions.

**Scheme 4:**
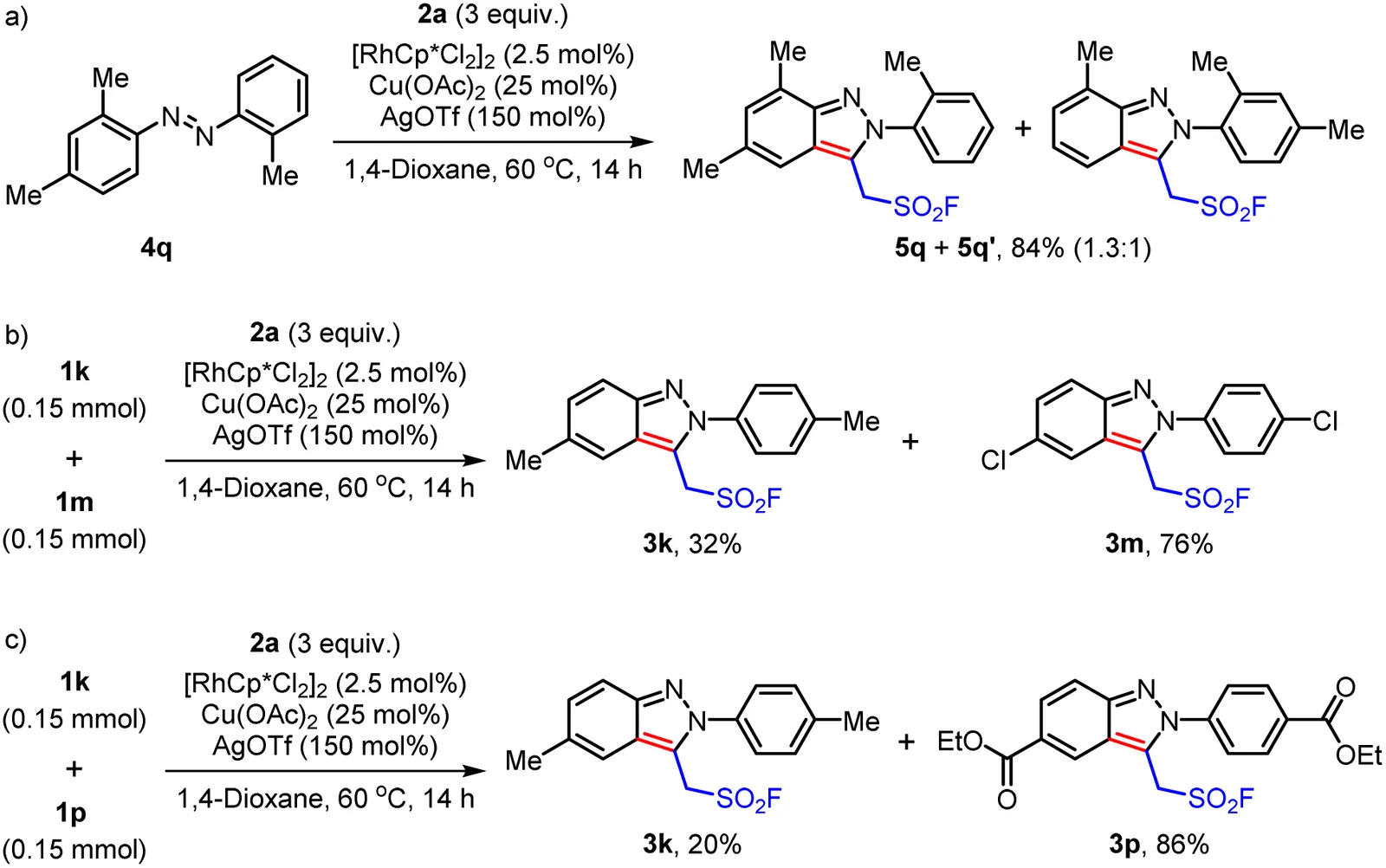
Regioselectivity and intermolecular competition experiments. (a) Regioselectivity experiment with an unsymmetrically substituted azobenzene; (b) and (c) intermolecular competition experiments.

The reaction mechanism was further investigated through deuterium-labelling experiments using MeOD under the standard reaction conditions (Scheme 5). In the absence of ESF (2a), substantial deuterium incorporation (43% D) was observed at the *ortho* position of recovered azobenzene 1a, supporting reversible Rh(III)-mediated C–H activation. When the experiment was conducted in the presence of 2a, product 3a was obtained in 63% yield without detectable deuterium incorporation, and no detectable H/D exchange was observed in the recovered 1a. In addition, treatment of the alkylation product 3aa under the standard annulation conditions, either in the presence or absence of ESF, did not afford 3a. This result suggests that the protonated alkylation product 3aa is unlikely to be a productive intermediate in the annulation pathway.

**Scheme 5:**
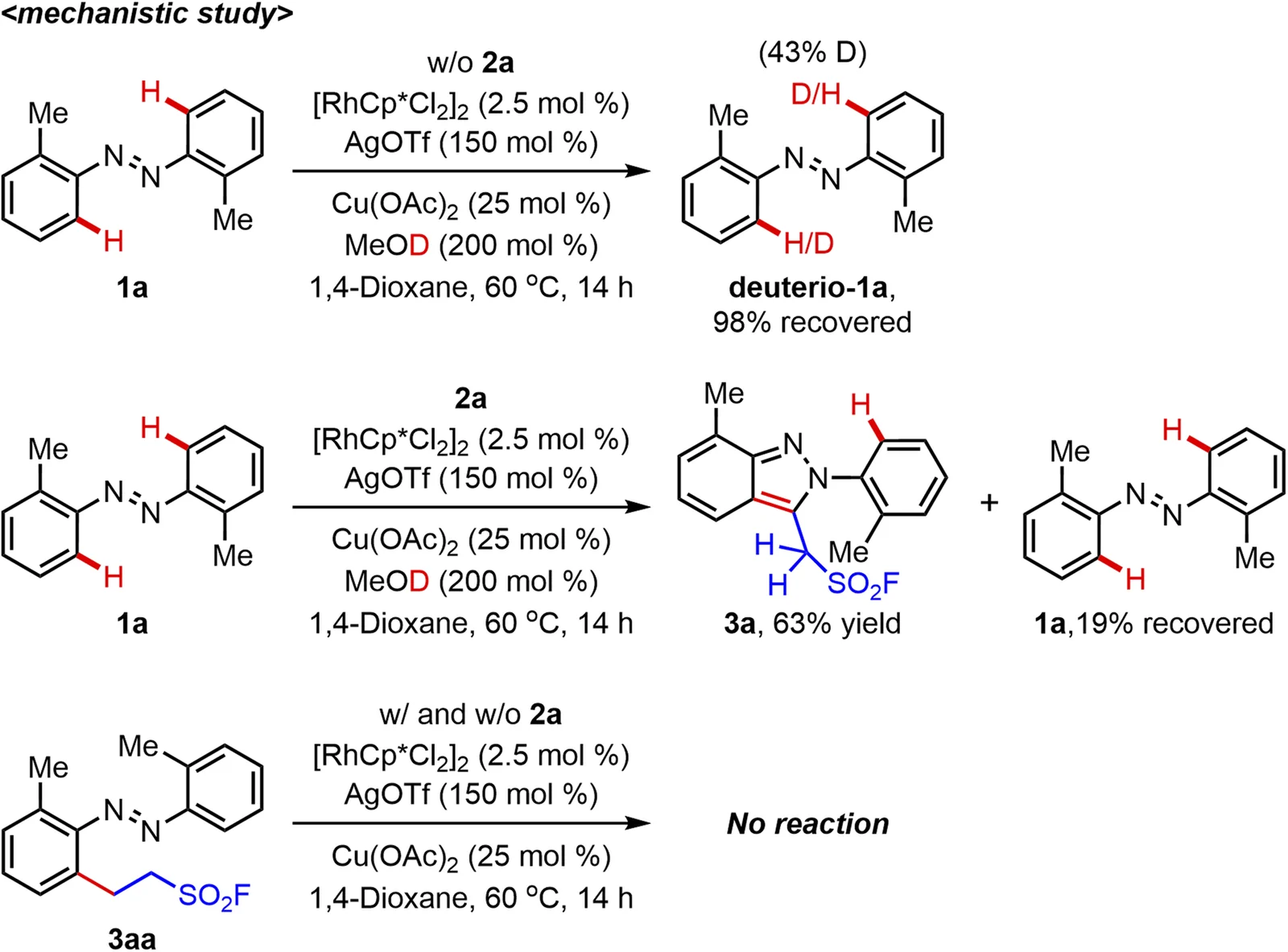
Mechanistic investigation.

Based on our mechanistic experiments and precedent reports on Rh(iii)-catalyzed C–H functionalization of azobenzenes,^5,6,8–10^ a plausible reaction mechanism for the formation of SO_2_F-containing indazoles is depicted in Scheme 6. Initially, generation of a cationic Rh(iii) species from [RhCp*Cl_2_]_2_ and AgOTf followed by *ortho*-C–H activation of azobenzene (1a) affords rhodacycle intermediate I. Subsequent coordination of ethenesulfonyl fluoride 2a and migratory insertion into the Rh–C bond produces seven-membered rhodacycle intermediate III*via* intermediate II. β-Hydride elimination from intermediate III is proposed to generate intermediate IV, which undergoes intramolecular aza-Michael addition to form cyclic ammonium intermediate V while regenerating the active Rh(iii) catalyst. Subsequent proton transfer and rearomatization furnish indazole (3a).

**Scheme 6:**
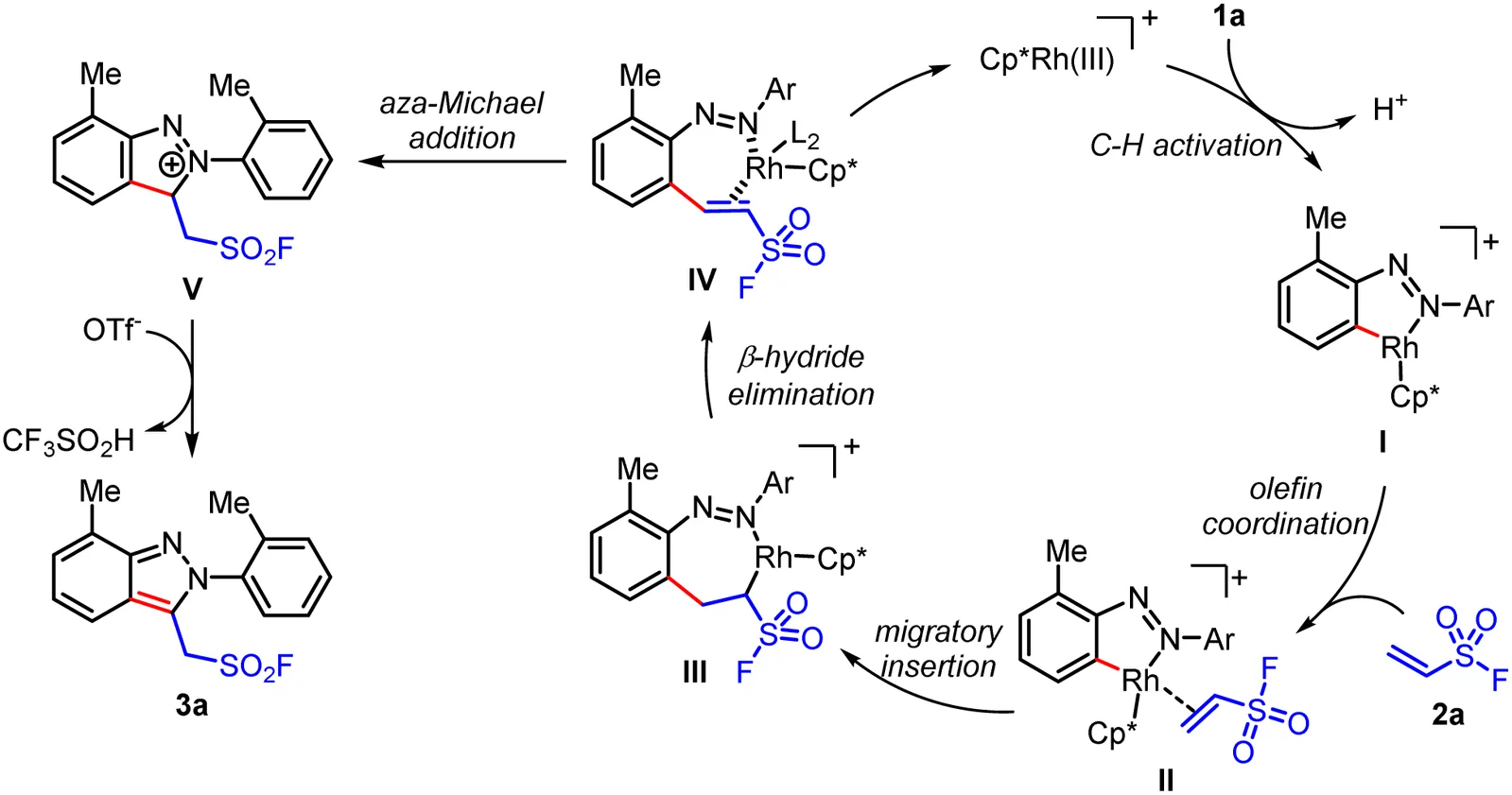
Plausible reaction mechanism.

To demonstrate the practicality and synthetic utility of the developed protocol, a gram-scale experiment and subsequent SuFEx derivatizations were performed (Scheme 7). The annulation reaction of azobenzene 1a with ethenesulfonyl fluoride 2a proceeded smoothly on a 1 g scale, affording the SO_2_F-containing indazole scaffold 3a in 70% yield without significant loss of efficiency. The synthetic versatility of 3a was further demonstrated through sulfur(vi)-fluoride exchange reactions with a simple secondary amine and a variety of biologically relevant nucleophiles. As a simple example, treatment of 3a with pyrrolidine smoothly afforded sulfonamide 6a in 81% yield. Likewise, SuFEx reactions of 3a with protected lysine and tyrosine derivatives furnished 6b and 6c, respectively, demonstrating the compatibility of the sulfonyl fluoride functionality with both N- and O-containing amino acid analogues. Furthermore, conjugation of 3a with lenalidomide, a cereblon (CRBN) E3 ligase ligand, afforded conjugate 6d, highlighting the utility of the SO_2_F-containing indazole scaffold for conjugation with medicinally relevant molecular architectures. In addition, reactions of 3a with a steroidal hormone estrone and a natural product 7-hydroxyflavone gave conjugates 6e and 6f, respectively, further highlighting the utility of the sulfonyl fluoride-containing indazole scaffold for the late-stage modification of bioactive molecules. These transformations demonstrate that the sulfonyl fluoride functional group is preserved throughout the reaction process and serves as a versatile SuFEx handle for the rapid generation of structurally diverse and medicinally relevant conjugates.

**Scheme 7:**
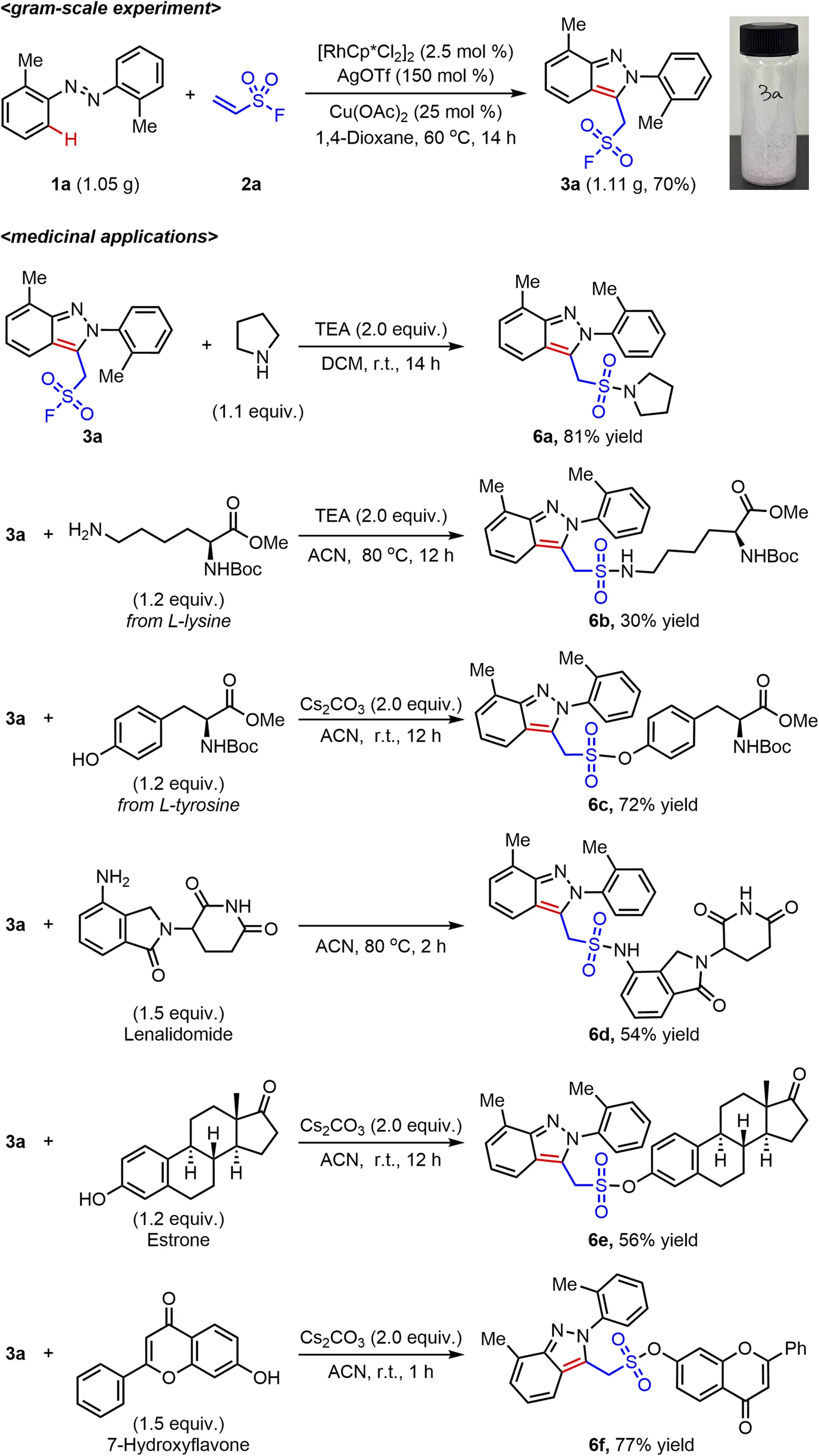
Gram-scale synthesis and medicinally relevant SuFEx derivatizations.

## Conclusions

In summary, we have developed a Rh(iii)-catalyzed C–H activation and annulation of azobenzenes with ethenesulfonyl fluoride, providing efficient access to SO_2_F-containing 2*H*-indazoles. The reaction proceeds under mild conditions and exhibits broad substrate scope across a diverse range of mono- and di-substituted azobenzenes. Experiments with an unsymmetrical azobenzene and intermolecular competition experiments provided insight into the regioselectivity and relative reactivity of azobenzene substrates under the optimized reaction conditions, and a plausible reaction mechanism has been proposed based on mechanistic investigations and precedent studies. Notably, the resulting sulfonyl fluoride-containing indazole scaffolds serve as versatile SuFEx hubs and undergo efficient conjugation with pyrrolidine, protected amino acid derivatives, lenalidomide, estrone, and 7-hydroxyflavone, demonstrating their utility for medicinal chemistry and chemical biology applications. We anticipate that this strategy will provide a useful platform for the synthesis of SO_2_F-containing heterocyclic scaffolds and their subsequent diversification through SuFEx chemistry.

## Author contributions

Lingayya Rajaka: validation, formal analysis, investigation, data curation, writing – review & editing. Hyosik Woo: validation, formal analysis, investigation, data curation. Myunghwa Jung: validation, investigation. Chanhee Jang: investigation, resources. Seungwan Kim: validation, investigation. Jihyo Lee: validation, investigation. Saegun Kim: conceptualization, methodology. Sangil Han: conceptualization, methodology, resources, writing – review & editing, supervision, funding acquisition. Han Yong Bae: conceptualization, resources, writing – review & editing, supervision. Sang Hoon Han: conceptualization, methodology, resources, writing – original draft, writing – review & editing, visualization, supervision, project administration, funding acquisition.

## Conflicts of interest

There are no conflicts to declare.

## Supplementary Material

RA-OLF-D6RA09632H-s001

## Data Availability

The data supporting this article have been included as part of the supplementary information (SI). Supplementary information: experimental procedures, compound characterization data, NMR spectra, and HRMS data. See DOI: https://doi.org/10.1039/d6ra09632h.
