# Supplementary material for: Synthesis and diversification of SuFEx-active 2H-indazoles from azobenzenes and ethenesulfonyl fluoride
Source: RSC Adv. 2026 Oct 19;16(48):64314–9. doi: 10.1039/d6ra09632h (PMC13678690; doi:10.1039/d6ra09632h)

Supplementary Information (SI) for RSC Advances.
This journal is © The Royal Society of Chemistry

Supporting Information

Synthesis and Diversification of SuFEx-Active 2*H*-Indazoles from Azobenzenes and Ethenesulfonyl Fluoride

Lingayya Rajaka,[a,b†] Hyosik Woo,[a,†] Myunghwa Jung,[a] Chanhee Jang,[c] Seungwan Kim,[a] Jihyo Lee,[a] Saegun Kim,[d] Sangil Han,[c,*] Han Yong Bae,[e,*] and Sang Hoon Han [a,*]

[a] *College of Pharmacy, Korea University, Sejong 30019, Republic of Korea*

[b] *Institute of Pharmaceutical Science and Translational Research, Korea University, Sejong 30019, Republic of Korea*

[c] *Department of Innovative Pharmaceutical Sciences, Kyungpook National University, Daegu 41566, Republic of Korea*

[d] *College of Pharmacy and Research Institute of Pharmaceutical Sciences, Sookmyung Women's University, Seoul 04310, Republic of Korea*

[e] *Department of Chemistry, Sungkyunkwan University, Suwon 16419, Republic of Korea*

* Corresponding authors E-mails: hans3@Korea.ac.kr (S. H. H.), sangil.han@knu.ac.kr (S. H.), hybae@skku.edu (H. Y. B)

[†] L.R. and H.W. equally contributed.

List of the Contents

1. General information and methods -- **S3**

2. Typical procedure for the synthesis of 2*H*-indazoles -- **S4**

3. Competition experiments --- **S5**

4. Deuterium-labelling experiments ---**S6**

5. Experimental procedure for the gram scale reaction --------------------------------------- **S7**

6. Experimental procedure for the applications --- **S8**

7. Characterization data for all products (3a–3p, 4a, 5a–5p, 6a-6f) ----------------------- **S11**

8. ^{1}H NMR, ^{13}C NMR and ^{19}F NMR spectra of products ------------------------------------ **S32**

9. HR-MS analytical data of the products --- **S108**

1. General information and methods

All the experiments were carried out in a well-ventilated fume hood under ambient reaction conditions. Commercially available reagents were used without additional purification, unless otherwise stated. Ethenesulfonyl fluoride (**2a**) was purchased from TCI. Azobenzenes **1a−1p** and **4a−4p**[1, 2] were prepared according to the reported literature. Sealed tubes (13 × 100 mm) were purchased from Fisher Scientific and dried in an oven overnight and cooled at room temperature before use. Thin-layer chromatography was carried out using plates coated with Kieselgel 60 F_{254} (Merck). Nuclear magnetic resonance spectra (^{1}H, ^{13}C and ^{19}F NMR) were recorded on a Jeol 400 and a Bruker 600 MHz NMR instrument in $CDCl_3$ solution and chemical shifts are reported as parts per million (ppm). Resonance patterns are reported with the notations s (singlet), d (doublet), t (triplet), q (quartet), m (multiplet) and br (broad). Coupling constants (*J*) are reported in hertz (Hz). IR spectra were recorded on a JASCO FT/IR4600 spectrophotometer and are reported as cm^{-1}. High resolution mass spectra (HRMS) were recorded on a JEOL JMS-700 spectrometer.

References

[1] Zhang, C.; Jiao, N. *Angew. Chem. Int. Ed.* **2010**, *49*, 6174−6177.
[2] Zhao, R.; Tan, C.; Xie, Y.; Gao, C.; Liu, H.; Jiang, Y. *Tetrahedron Lett.* **2011**, *52*, 3805−3809.

2. Typical procedure for the synthesis of 2*H*-indazoles

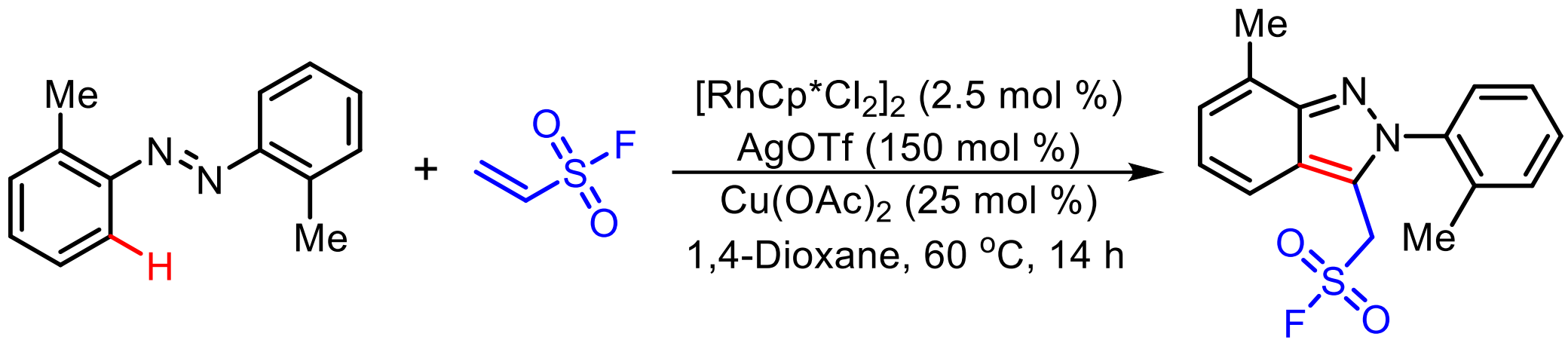

1a **2a** **3a**

To an oven-dried sealed tube charged with 1,2-di-*o*-tolyldiazene (**1a**) (42.0 mg, 0.2 mmol, 100 mol %), $[RhCp^*Cl_2]_2$ (3.1 mg, 0.005 mmol, 2.5 mol %), $Cu(OAc)_2$ (9.0 mg, 0.05 mmol, 25 mol %), AgOTf (77.0 mg, 0.3 mmol, 150 mol %), 1,4-dioxane (1 mL), was added ethenesulfonyl fluoride (**2a**) (66.0 mg, 0.6 mmol, 300 mol %). The reaction mixture was allowed to stir for 14 h at 60 °C. After completion of reaction, cool down reaction mass to room temperature. The reaction mass quenched with H_2O, diluted with EtOAc (10 mL) and did workup. EtOAc layer dried with $MgSO_4$, filtered, concentrated in vacuo. The residue was purified by flash column chromatography (*n*-hexanes/EtOAc = 9:1) to afford **3a** (50.9 mg) in 80% yield.

3. Competition experiments

b)

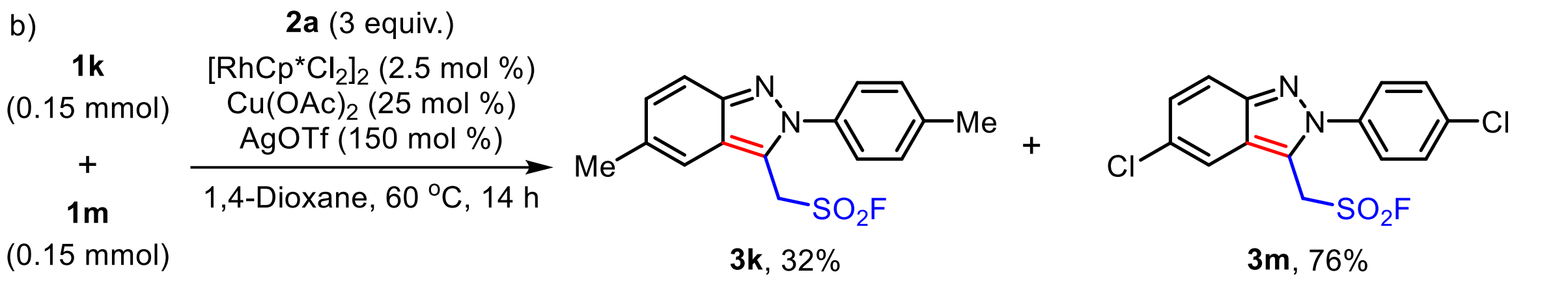

c)

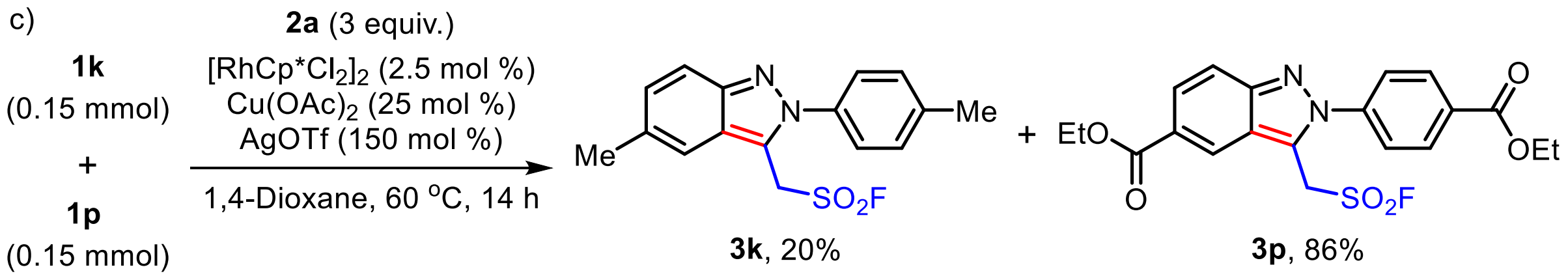

b) To an oven-dried sealed tube charged with 1,2-di-*p*-tolyldiazene (**1k**) (31.5 mg, 0.15 mmol, 50 mol%), 1,2-bis(4-chlorophenyl)diazene (**1m**) (37.6 mg, 0.15 mmol, 50 mol%), $[RhCp^*Cl_2]_2$ (4.6 mg, 0.0075 mmol, 2.5 mol %), $Cu(OAc)_2$ (13.6 mg, 0.075 mmol, 25 mol %), AgOTf (115.6 mg, 0.45 mmol, 150 mol %), 1,4-dioxane (1 mL), was added ethenesulfonyl fluoride (**2a**) (99.1 mg, 0.9 mmol, 300 mol %). The reaction mixture was allowed to stir for 14 h at 60 °C. After completion of reaction, cool down reaction mass to room temperature. The reaction mass quenched with H_2O, diluted with EtOAc (10 mL) and did workup. EtOAc layer dried with $MgSO_4$, filtered, concentrated in vacuo. The residue was purified by flash column chromatography (*n*-hexanes/EtOAc = 9:1) to afford **3k** (15.2 mg) in 32% yield and **3m** (41.2 mg) in 76% yield.

c) To an oven-dried sealed tube charged with 1,2-di-*p*-tolyldiazene (**1k**) (31.5 mg, 0.15 mmol, 50 mol%), diethyl 4,4'-(diazene-1,2-diyl)dibenzoate (**1p**) (48.9 mg, 0.15 mmol, 50 mol%), $[RhCp^*Cl_2]_2$ (4.6 mg, 0.0075 mmol, 2.5 mol %), $Cu(OAc)_2$ (13.6 mg, 0.075 mmol, 25 mol %), AgOTf (115.6 mg, 0.45 mmol, 150 mol %), 1,4-dioxane (1 mL), was added ethenesulfonyl fluoride (**2a**) (99.1 mg, 0.9 mmol, 300 mol %). The reaction mixture was allowed to stir for 14 h at 60 °C. After completion of reaction, cool down reaction mass to room temperature. The reaction mass quenched with H_2O, diluted with EtOAc (10 mL) and did workup. EtOAc layer dried with $MgSO_4$, filtered, concentrated in vacuo. The residue was purified by flash column chromatography (*n*-hexanes/EtOAc = 9:1) to afford **3k** (9.6 mg) in 20% yield and **3p** (56.0 mg) in 86% yield.

4. Deuterium-labelling experiments

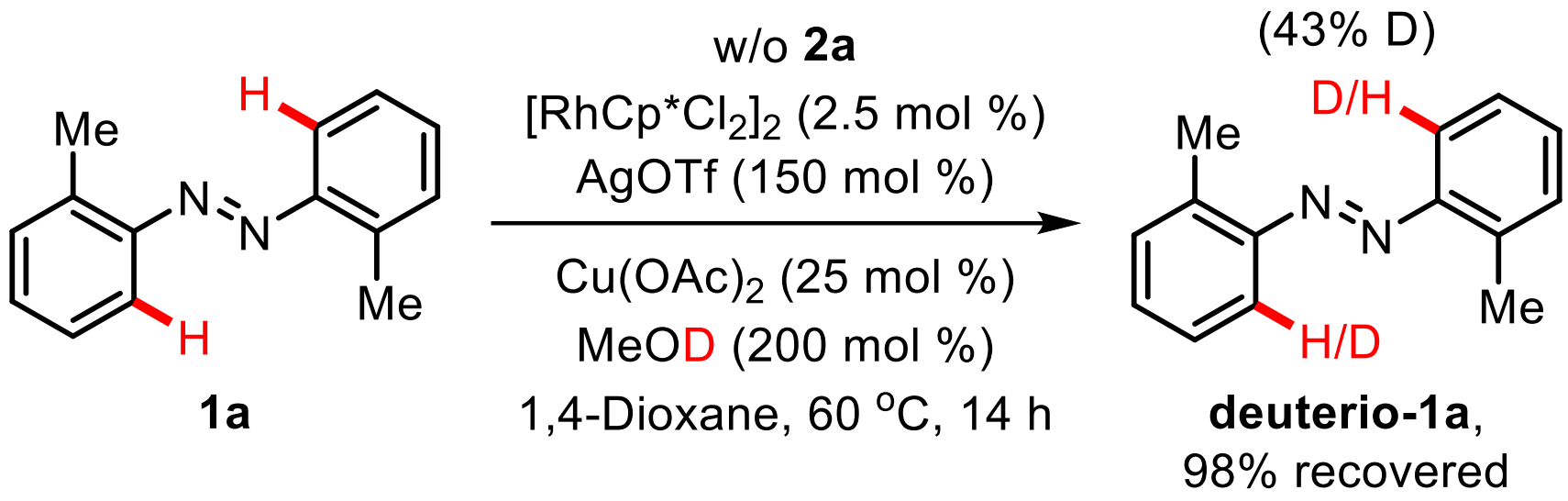

To an oven-dried sealed tube charged with 1,2-di-*o*-tolyldiazene (**1a**) (42.0 mg, 0.2 mmol, 100 mol %), $[RhCp*Cl_2]_2$ (3.1 mg, 0.005 mmol, 2.5 mol %), $Cu(OAc)_2$ (9.0 mg, 0.05 mmol, 25 mol %), AgOTf (77.0 mg, 0.3 mmol, 150 mol %), 1,4-dioxane (1 mL), was added MeOD (14.4 mg, 0.4 mmol, 200 mol %). The reaction mixture was allowed to stir for 14 h at 60 °C. After completion of reaction, cool down reaction mass to room temperature. The reaction mass quenched with H_2O, diluted with EtOAc (10 mL) and did workup. EtOAc layer dried with $MgSO_4$, filtered, concentrated in vacuo. The residue was purified by flash column chromatography (*n*-hexanes/EtOAc = 9:1) to afford **deuterio-1a** (41.3 mg) in 98% yield.

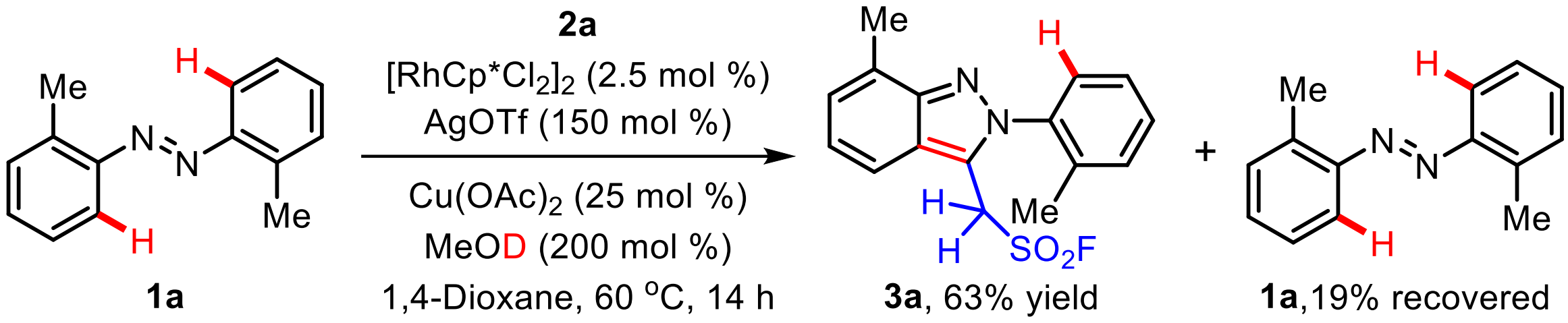

To an oven-dried sealed tube charged with 1,2-di-*o*-tolyldiazene (**1a**) (42.0 mg, 0.2 mmol, 100 mol %), $[RhCp*Cl_2]_2$ (3.1 mg, 0.005 mmol, 2.5 mol %), $Cu(OAc)_2$ (9.0 mg, 0.05 mmol, 25 mol %), AgOTf (77.0 mg, 0.3 mmol, 150 mol %), 1,4-dioxane (1 mL), MeOD (14.4 mg, 0.4 mmol, 200 mol %), was added ethenesulfonyl fluoride (**2a**) (66.0 mg, 0.6 mmol, 300 mol %). The reaction mixture was allowed to stir for 14 h at 60 °C. After completion of reaction, cool down reaction mass to room temperature. The reaction mass quenched with H_2O, diluted with EtOAc (10 mL) and did workup. EtOAc layer dried with $MgSO_4$, filtered, concentrated in vacuo. The residue was purified by flash column chromatography (*n*-hexanes/EtOAc = 9:1) to afford **3a** (39.9 mg) in 63% yield and recovered **1a** (8.1 mg) in 19% yield.

5. Experimental procedure for the gram scale reaction

To an oven-dried sealed tube charged with 1,2-di-*o*-tolyldiazene (**1a**) (1051.4 mg, 5.0 mmol, 100 mol %), $[RhCp^{*}Cl_2]_2$ (77.2 mg, 0.125 mmol, 2.5 mol %), $Cu(OAc)_2$ (227.0 mg, 1.25 mmol, 25 mol %), AgOTf (1927.0 mg, 7.5 mmol, 150 mol %), 1,4-dioxane (10 mL), was added ethenesulfonyl fluoride (**2a**) (1651.5 mg, 15.0 mmol, 300 mol %). The reaction mixture was allowed to stir for 14 h at 60 ºC. After completion of reaction, cool down reaction mass to room temperature. The reaction mass quenched with H_2O, diluted with EtOAc (25 mL) and did workup. EtOAc layer dried with $MgSO_4$, filtered, concentrated in vacuo. The residue was purified by flash column chromatography (*n*-hexanes/EtOAc = 9:1) to afford **3a** (1110 mg) in 70% yield.

6. Experimental procedure for the applications

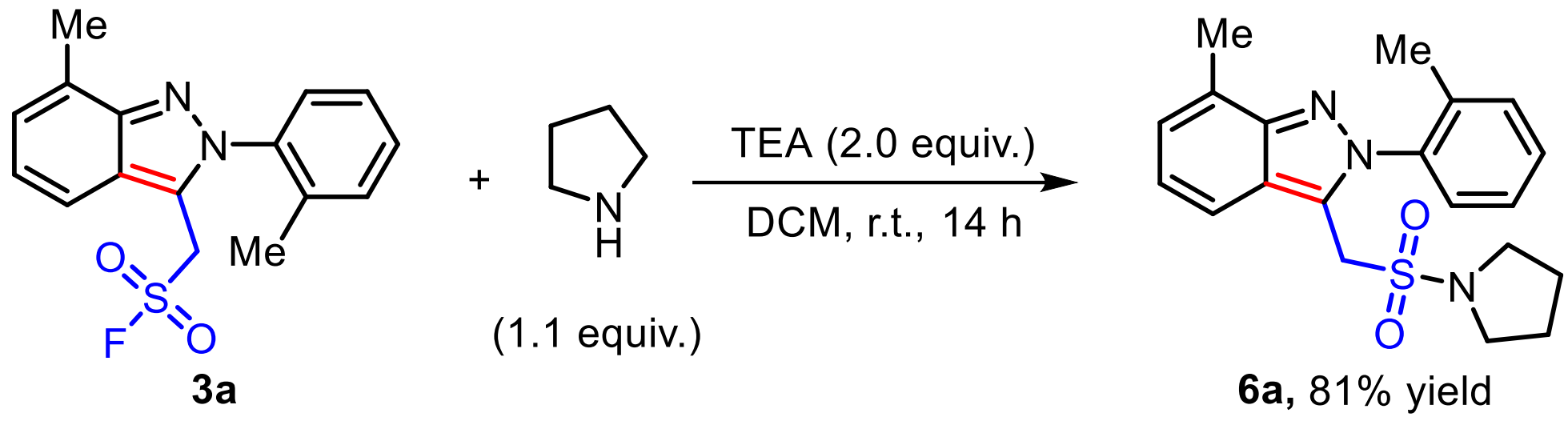

To an oven-dried sealed tube charged with (7-methyl-2-(*o*-tolyl)-2*H*-indazol-3-yl)methanesulfonyl fluoride (**3a**) (31.8 mg, 0.1 mmol, 100 mol %), pyrrolidine (7.8 mg, 0.11 mmol, 110 mol %), TEA (20.2 mg, 0.2 mmol, 200 mol %), DCM (1 mL). The reaction mixture was allowed to stir for 14 h at 25 °C. After completion of reaction as indicated by TLC analysis, reaction mass quenched with H_2O, diluted with EtOAc (10 mL) and did workup. EtOAc layer dried with $MgSO_4$, filtered, concentrated in vacuo. The residue was purified by flash column chromatography (*n*-hexanes/EtOAc = 2:1) to afford **6a** (30.0 mg) in 81% yield.

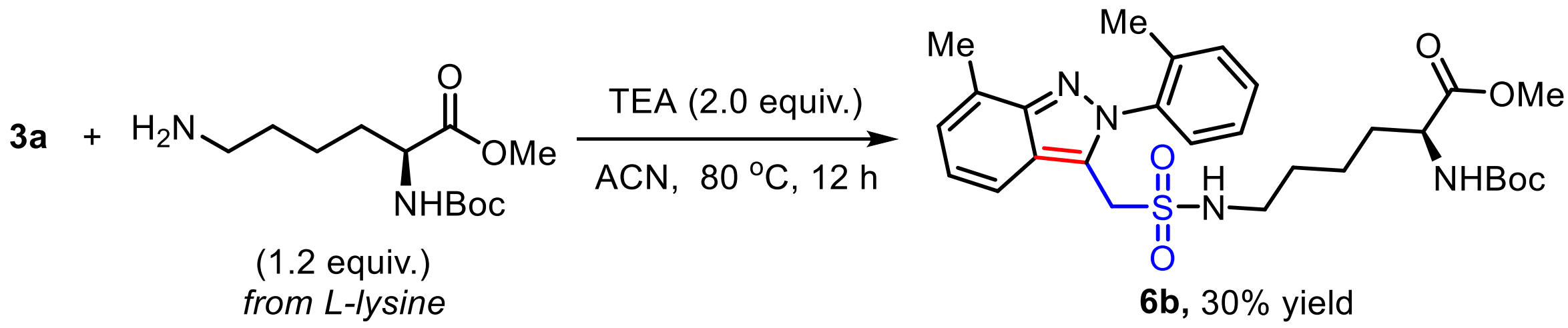

To an oven-dried sealed tube charged with (7-methyl-2-(*o*-tolyl)-2*H*-indazol-3-yl)methanesulfonyl fluoride (**3a**) (31.8 mg, 0.1 mmol, 100 mol %), protected lysine amino acid derivative (31.2 mg, 0.12 mmol, 120 mol %), TEA (20.2 mg, 0.2 mmol, 200 mol %), acetonitrile (1 mL). The reaction mixture was allowed to stir for 12 h at 80 °C. After completion of reaction as indicated by TLC analysis, reaction mass quenched with H_2O, diluted with EtOAc (10 mL) and did workup. EtOAc layer dried with $MgSO_4$, filtered, concentrated in vacuo. The residue was purified by flash column chromatography (*n*-hexanes/EtOAc = 1:1) to afford **6b** (16.8 mg) in 30% yield.

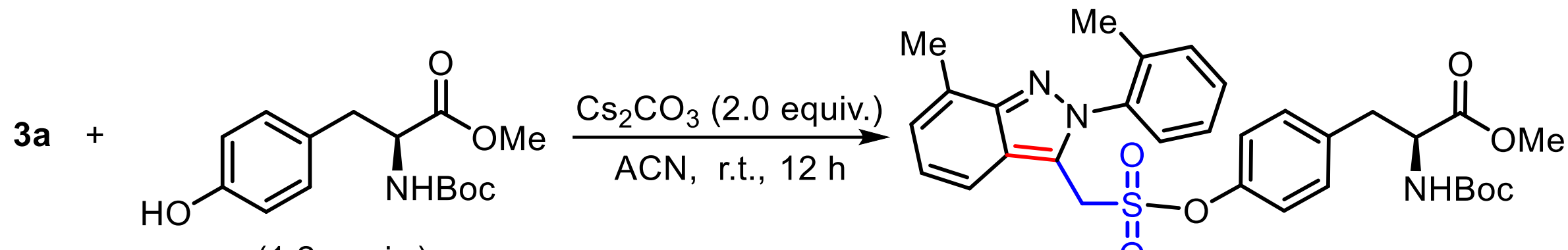

(1.2 equiv.)
from L-tyrosine

6c, 72% yield

To an oven-dried sealed tube charged with (7-methyl-2-(*o*-tolyl)-2*H*-indazol-3-yl)methanesulfonyl fluoride (**3a**) (31.8 mg, 0.1 mmol, 100 mol %), protected tyrosine amino acid derivative (35.4 mg, 0.12 mmol, 120 mol %), Cs_2CO_3 (65.1 mg, 0.2 mmol, 200 mol %), acetonitrile (1 mL). The reaction mixture was allowed to stir for 12 h at 25 °C. After completion of reaction as indicated by TLC analysis, reaction mass quenched with H_2O, diluted with EtOAc (10 mL) and did workup. EtOAc layer dried with $MgSO_4$, filtered, concentrated in vacuo. The residue was purified by flash column chromatography (*n*-hexanes/EtOAc = 3:1) to afford **6c** (42.8 mg) in 72% yield.

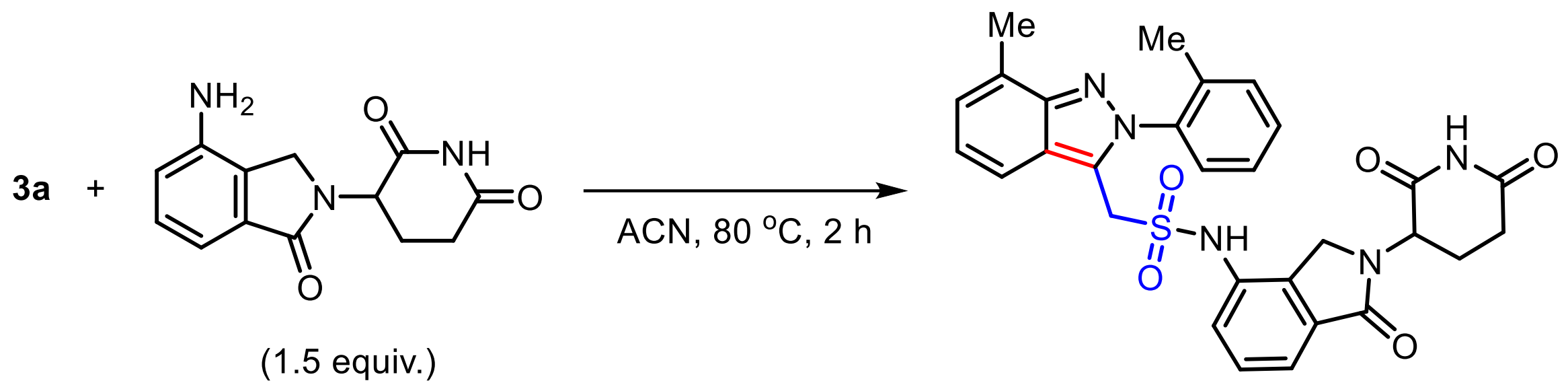

(1.5 equiv.)
Lenalidomide

6d, 54% yield

To an oven-dried sealed tube charged with (7-methyl-2-(*o*-tolyl)-2*H*-indazol-3-yl)methanesulfonyl fluoride (**3a**) (31.8 mg, 0.1 mmol, 100 mol %), lenalidomide (38.9 mg, 0.15 mmol, 150 mol %), acetonitrile (1 mL). The reaction mixture was allowed to stir for 2 h at 80 °C. After completion of reaction as indicated by TLC analysis, reaction mass quenched with H_2O, diluted with EtOAc (10 mL) and did workup. EtOAc layer dried with $MgSO_4$, filtered, concentrated in vacuo. The residue was purified by flash column chromatography (*n*-hexanes/EtOAc = 1:4) to afford **6d** (30.2 mg) in 54% yield.

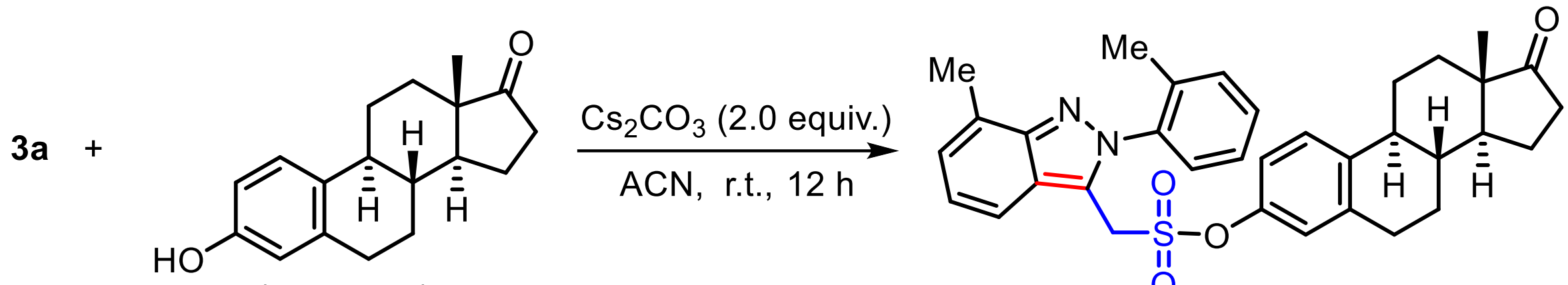

(1.2 equiv.)
Estrone

6e, 56% yield

To an oven-dried sealed tube charged with (7-methyl-2-(*o*-tolyl)-2*H*-indazol-3-yl)methanesulfonyl fluoride (**3a**) (31.8 mg, 0.1 mmol, 100 mol %), estrone (32.4 mg, 0.12 mmol, 120 mol %), Cs_2CO_3 (65.1 mg, 0.2 mmol, 200 mol %), acetonitrile (1 mL). The reaction mixture was allowed to stir for 12 h at 25 ºC. After completion of reaction as indicated by TLC analysis, reaction mass quenched with H_2O, diluted with EtOAc (10 mL) and did workup. EtOAc layer dried with $MgSO_4$, filtered, concentrated in vacuo. The residue was purified by flash column chromatography (*n*-hexanes/EtOAc = 2:1) to afford **6e** (31.9 mg) in 56% yield.

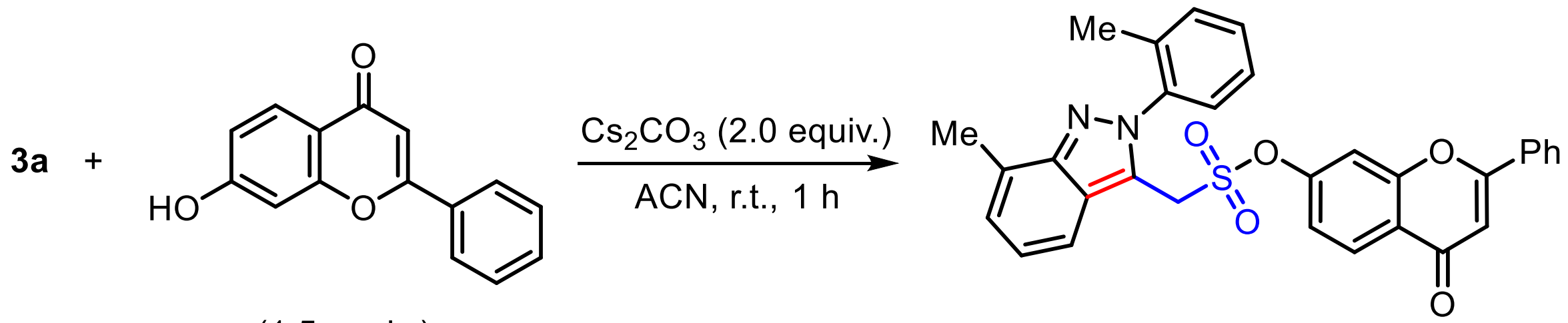

(1.5 equiv.)
7-Hydorxyflavone

6f, 77% yield

To an oven-dried sealed tube charged with (7-methyl-2-(*o*-tolyl)-2*H*-indazol-3-yl)methanesulfonyl fluoride (**3a**) (31.8 mg, 0.1 mmol, 100 mol %), 7-hydroxyflavone (35.7 mg, 0.15 mmol, 150 mol %), Cs_2CO_3 (65.1 mg, 0.2 mmol, 200 mol %), acetonitrile (1 mL). The reaction mixture was allowed to stir for 1 h at 25 ºC. After completion of reaction as indicated by TLC analysis, reaction mass quenched with H_2O, diluted with EtOAc (10 mL) and did workup. EtOAc layer dried with $MgSO_4$, filtered, concentrated in vacuo. The residue was purified by flash column chromatography (*n*-hexanes/EtOAc = 3:1) to afford **6f** (41.5 mg) in 77% yield.

7. Characterization data for all products (3a–3p, 3aa, 5a–5q, 5q', 6a-6f)

(7-Methyl-2-(*o*-tolyl)-2*H*-indazol-3-yl)methanesulfonyl fluoride (3a)

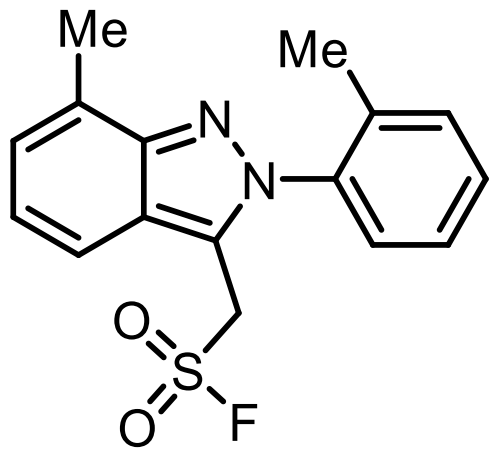

3a

50.9 mg (80%); Pale violet solid; ^{1}H NMR (600 MHz, $CDCl_3$) δ 7.58 (d, *J* = 8.2 Hz, 1H), 7.51 – 7.48 (m, 1H), 7.43 – 7.41 (m, 1H), 7.40 – 7.38 (m, 2H), 7.21 (d, *J* = 6.7 Hz, 1H), 7.19 – 7.17 (m, 1H), 4.91 (d, *J* = 89.5 Hz, 2H), 2.67 (s, 3H), 2.02 (s, 3H) ppm; ^{13}C NMR (150 MHz, $CDCl_3$) δ 149.23, 137.24, 136.50, 131.74, 130.78, 128.93, 127.89, 127.09, 126.34, 124.51, 122.00, 120.19, 116.57, 47.61 (d, *J* = 20.8 Hz), 17.20, 17.12 ppm; ^{19}F NMR (376 MHz, $CDCl_3$) δ 55.66 ppm; FT-IR ($CHCl_3$, film) υ 3028, 2919, 1503, 1409, 1204, 869 cm^{-1}; HRMS (EI+) calcd for $C_{16}H_{15}FN_2O_2S$ $[M]^+$ 318.0838, found 318.0834.

(7-Ethyl-2-(2-ethylphenyl)-2*H*-indazol-3-yl)methanesulfonyl fluoride (3b)

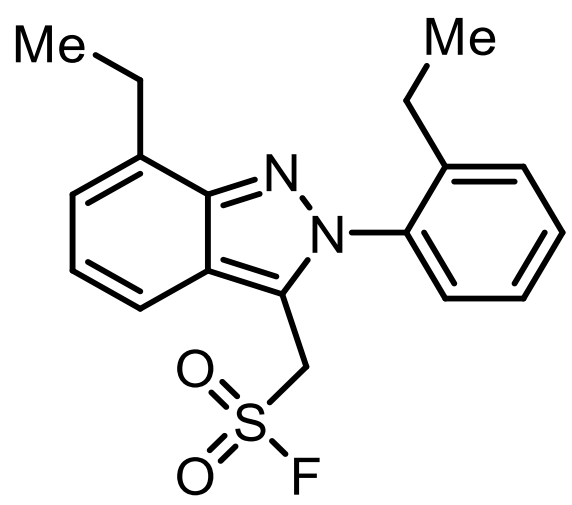

3b

50.3 mg (73%); Yellow solid; ^{1}H NMR (600 MHz, $CDCl_3$) δ 7.51 (d, *J* = 8.3 Hz, 1H), 7.48 – 7.45 (m, 1H), 7.40 (d, *J* = 7.4 Hz, 1H), 7.33 – 7.31 (m, 2H), 7.18 – 7.11 (m, 2H), 4.82 (d, *J* = 109.5 Hz, 2H), 3.02 (d, *J* = 20.6 Hz, 2H), 2.15 (d, *J* = 7.2 Hz, 2H), 1.33 (t, *J* = 7.6 Hz, 3H), 1.04 (t, *J* = 7.6 Hz, 3H) ppm; ^{13}C NMR (150 MHz, $CDCl_3$) δ 148.40, 142.26, 136.73, 135.01, 130.99, 129.93, 127.99, 126.97, 124.58, 124.33, 122.15, 120.31, 116.58 (d, *J* = 1.1 Hz), 47.72 (d, *J* = 21.4 Hz), 24.47, 23.82, 14.74, 14.20 ppm; ^{19}F NMR (376 MHz, $CDCl_3$) δ 55.80 ppm; FT-IR ($CHCl_3$, film) υ 2968, 2930, 1534, 1498, 1460, 1411, 1236, 1206, 1089, 889 cm^{-1}; HRMS

(EI+) calcd for $C_{18}H_{19}FN_2O_2S$ $[M]^+$ 346.1151, found 346.1146.

(7-Fluoro-2-(2-fluorophenyl)-2*H*-indazol-3-yl)methanesulfonyl fluoride (3c)

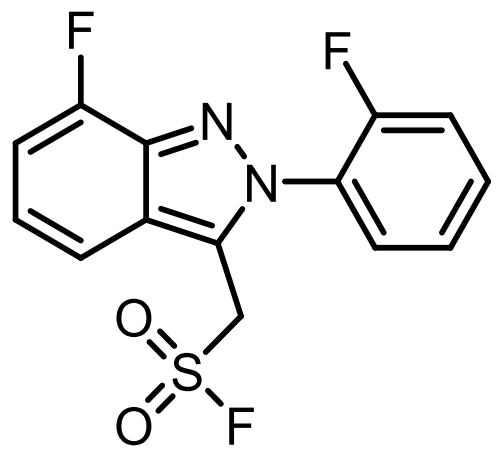

3c

51.0 mg (78%); Brown solid; ^1H NMR (600 MHz, $CDCl_3$) δ 7.67 (td, *J* = 7.6, 1.7 Hz, 1H), 7.64 – 7.60 (m, 1H), 7.51 (d, *J* = 8.5 Hz, 1H), 7.41 (t, *J* = 8.0 Hz, 1H), 7.35 (t, *J* = 8.5 Hz, 1H), 7.20 (td, *J* = 8.0, 4.3 Hz, 1H), 7.06 (dd, *J* = 11.0, 7.5 Hz, 1H), 5.01 (d, *J* = 3.5 Hz, 2H) ppm; ^{13}C NMR (150 MHz, $CDCl_3$) δ 157.25, 155.58, 154.13, 152.42, 140.96 (d, *J* = 17.0 Hz), 132.80 (d, *J* = 7.7 Hz), 130.06, 126.10 (d, *J* = 11.5 Hz), 125.66 (d, *J* = 3.9 Hz), 125.44 (d, *J* = 5.5 Hz), 124.73 (d, *J* = 6.0 Hz), 122.20 (d, *J* = 3.3 Hz), 117.08 (d, *J* = 18.7 Hz), 115.09 (d, *J* = 4.9 Hz), 110.57 (d, *J* = 15.9 Hz), 47.44 (dd, *J* = 21.7, 3.0 Hz) ppm; ^{19}F NMR (376 MHz, $CDCl_3$) δ 55.66, -122.37, -127.36 ppm; FT-IR ($CHCl_3$, film) υ 2947, 2921, 1507, 1405, 1267, 1240, 1206, 1090, 875 cm^{-1}; HRMS (EI+) calcd for $C_{14}H_9F_3N_2O_2S$ $[M]^+$ 326.0337, found 326.0336.

(7-Chloro-2-(2-chlorophenyl)-2*H*-indazol-3-yl)methanesulfonyl fluoride (3d)

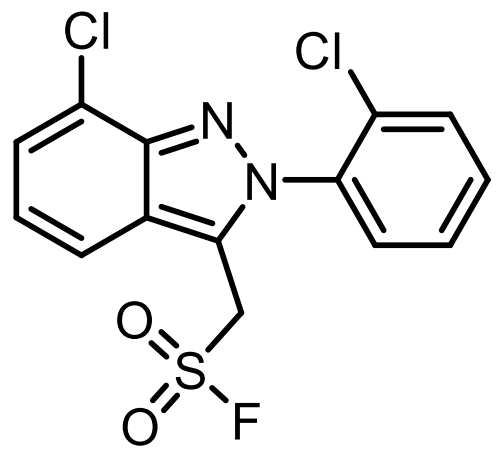

3d

51.8 mg (72%); Pale green solid; ^1H NMR (600 MHz, $CDCl_3$) δ 7.70 – 7.65 (m, 2H), 7.63 – 7.57 (m, 2H), 7.54 (td, *J* = 7.6, 1.7 Hz, 1H), 7.47 (d, *J* = 7.2 Hz, 1H), 7.23 (dd, *J* = 8.5, 7.2 Hz, 1H), 5.14 (d, *J* = 15.6 Hz, 1H), 4.73 (dd, *J* = 15.6, 5.4 Hz, 1H) ppm; ^{13}C NMR (150 MHz, $CDCl_3$) δ 146.98, 135.65, 132.38, 131.42, 130.80, 130.50, 128.42, 127.07, 124.79, 124.14, 123.41, 122.50, 118.09, 47.57 (d, *J* = 22.0 Hz) ppm; ^{19}F NMR (376 MHz, $CDCl_3$) δ 55.75 ppm; FT-IR ($CHCl_3$, film) υ 2923, 2851, 1490, 1407, 1264, 1200, 1052, 869 cm^{-1}; HRMS (EI+) calcd

for $C_{14}H_9Cl_2FN_2O_2S$ $[M]^+$ 357.9746, found 357.9742.

(6-Ethyl-2-(3-ethylphenyl)-2H-indazol-3-yl)methanesulfonyl fluoride (3e)

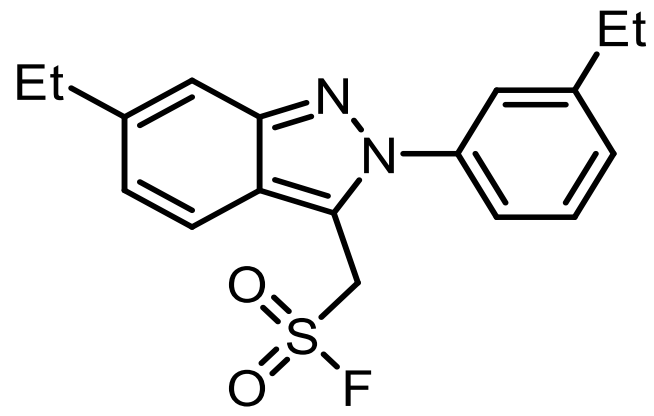

3e

32.8 mg (47%); Yellow solid; ^{1}H NMR (600 MHz, $CDCl_3$) δ 7.66 (d, J = 8.6 Hz, 1H), 7.58 (s, 1H), 7.49 (t, J = 7.7 Hz, 1H), 7.42 (d, J = 6.6 Hz, 2H), 7.39 (t, J = 8.1 Hz, 1H), 7.16 (dd, J = 8.7, 1.5 Hz, 1H), 5.04 (d, J = 2.9 Hz, 2H), 2.81 – 2.78 (m, 2H), 2.78 – 2.74 (m, 2H), 1.32 (t, J = 7.5 Hz, 3H), 1.29 (t, J = 7.6 Hz, 3H) ppm; ^{13}C NMR (150 MHz, $CDCl_3$) δ 149.51, 146.52, 143.89, 138.58, 129.83, 129.64, 126.33, 126.25, 123.79, 121.43, 119.27, 118.81, 115.49, 48.09 (d, J = 21.4 Hz), 29.55, 28.77, 15.40, 15.32 ppm; ^{19}F NMR (376 MHz, $CDCl_3$) δ 55.82 ppm; FT-IR ($CHCl_3$, film) υ 2962, 2929, 2873, 1495, 1408, 1206, 1054, 862 cm^{-1}; HRMS (EI+) calcd for $C_{18}H_{19}FN_2O_2S$ $[M]^+$ 346.1151, found 346.1152.

(6-Isopropyl-2-(3-isopropylphenyl)-2H-indazol-3-yl)methanesulfonyl fluoride (3f)

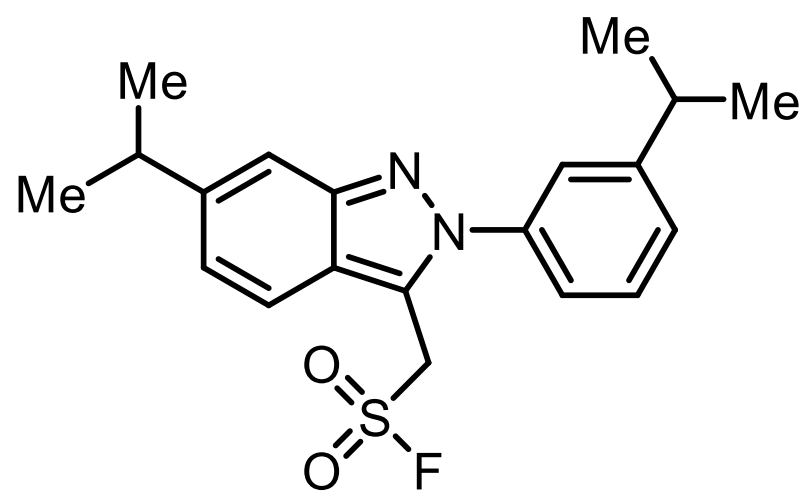

3f

50.2 mg (67%); Yellow solid; ^{1}H NMR (600 MHz, $CDCl_3$) δ 7.67 (d, J = 8.8 Hz, 1H), 7.62 (s, 1H), 7.49 (t, J = 7.7 Hz, 1H), 7.46 – 7.43 (m, 2H), 7.38 – 7.36 (m, 1H), 7.21 (dd, J = 8.8, 1.4 Hz, 1H), 5.02 (d, J = 2.9 Hz, 2H), 3.08 – 3.04 (m, 1H), 3.03 – 2.98 (m, 1H), 1.34 (d, J = 6.9 Hz, 6H), 1.30 (d, J = 6.9 Hz, 6H) ppm; ^{13}C NMR (150 MHz, $CDCl_3$) δ 151.19, 149.45, 148.47, 138.56, 129.63, 128.50, 125.10, 124.90, 123.89, 121.51, 119.25, 118.87, 114.01, 48.09 (d, J = 20.8 Hz), 34.65, 34.16, 23.90, 23.79 ppm; ^{19}F NMR (376 MHz, $CDCl_3$) δ 55.86 ppm; FT-IR ($CHCl_3$, film) υ 2964, 2874, 1497, 1375, 1040, 866 cm^{-1}; HRMS (EI+) calcd for $C_{20}H_{23}FN_2O_2S$

[M]+ 374.1464, found 374.1465.

(6-Fluoro-2-(3-fluorophenyl)-2*H*-indazol-3-yl)methanesulfonyl fluoride (3g)

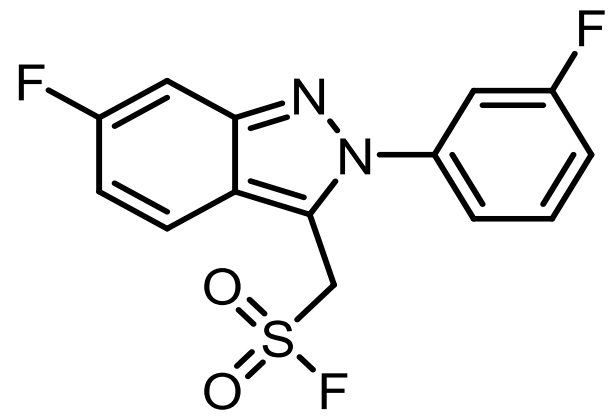

3g

37.2 mg (57%); Pale yellow solid; ^{1}H NMR (600 MHz, $CDCl_3$) δ 7.60 (d, *J* = 5.1 Hz, 1H), 7.58 (d, *J* = 8.2 Hz, 1H), 7.40 (d, *J* = 7.9 Hz, 1H), 7.35 – 7.33 (m, 2H), 7.32 (s, 1H), 6.89 (dd, *J* = 10.7, 7.5 Hz, 1H), 5.18 (d, *J* = 4.0 Hz, 2H) ppm; ^{13}C NMR (150 MHz, $CDCl_3$) δ 162.87 (d, *J* = 249.5 Hz), 155.18 (d, *J* = 252.2 Hz), 150.89 (d, *J* = 5.0 Hz), 139.29 (d, *J* = 9.8 Hz), 131.33 (d, *J* = 8.8 Hz), 127.92 (d, *J* = 6.6 Hz), 122.42 (d, *J* = 3.3 Hz), 118.98 (d, *J* = 5.5 Hz), 117.88 (d, *J* = 20.8 Hz), 114.84 (d, *J* = 2.7 Hz), 114.81, 114.67, 107.35 (d, *J* = 17.6 Hz), 47.91 (dd, *J* = 20.8, 2.2 Hz) ppm; ^{19}F NMR (376 MHz, $CDCl_3$) δ 55.23 (d, *J* = 22.6 Hz), -108.89, -121.54 ppm; FT-IR ($CHCl_3$, film) υ 2920, 2847, 1602, 1567, 1408, 1236, 1201, 1088, 871, 849, 811 cm^{-1}; HRMS (EI+) calcd for $C_{14}H_9F_3N_2O_2S$ $[M]^+$ 326.0337, found 326.0333.

(6-Bromo-2-(3-bromophenyl)-2*H*-indazol-3-yl)methanesulfonyl fluoride (3h)

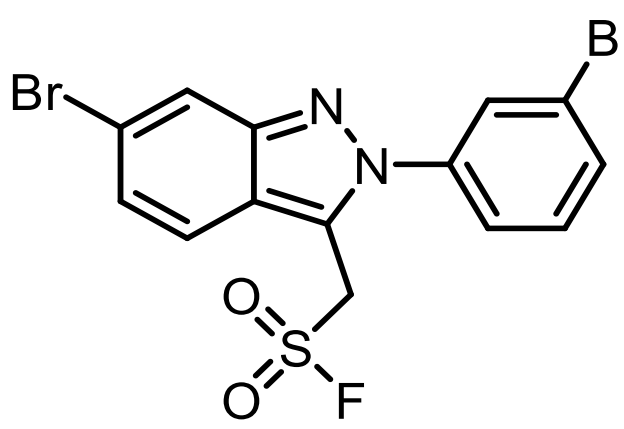

3h

54.7 mg (61%); Brown solid; ^{1}H NMR (600 MHz, $CDCl_3$) δ 7.92 (d, *J* = 1.7 Hz, 1H), 7.69 – 7.67 (m, 1H), 7.55 (d, *J* = 9.0 Hz, 1H), 7.50 – 7.49 (m, 1H), 7.41 (t, *J* = 8.0 Hz, 1H), 7.29 (dd, *J* = 9.0, 1.6 Hz, 1H), 7.19 (s, 1H), 4.97 (d, *J* = 2.3 Hz, 2H) ppm; ^{13}C NMR (150 MHz, $CDCl_3$) δ 149.67, 139.27, 133.81, 131.19, 129.96, 128.55, 125.17, 123.53, 121.97, 121.52, 121.03, 120.62, 120.50, 47.68 (d, *J* = 22.0 Hz) ppm; ^{19}F NMR (376 MHz, $CDCl_3$) δ 56.12 ppm; FT-IR ($CHCl_3$, film) υ 2924, 2853, 1581, 1476, 1259, 1196, 1035, 862 cm^{-1}; HRMS (EI+) calcd for

$C_{14}H_9Br_2FN_2O_2S$ $[M]^+$ 445.8736, found 445.8737.

Ethyl 2-(3-(ethoxycarbonyl)phenyl)-3-((fluorosulfonyl)methyl)-2*H*-indazole-6-carboxylate (3i)

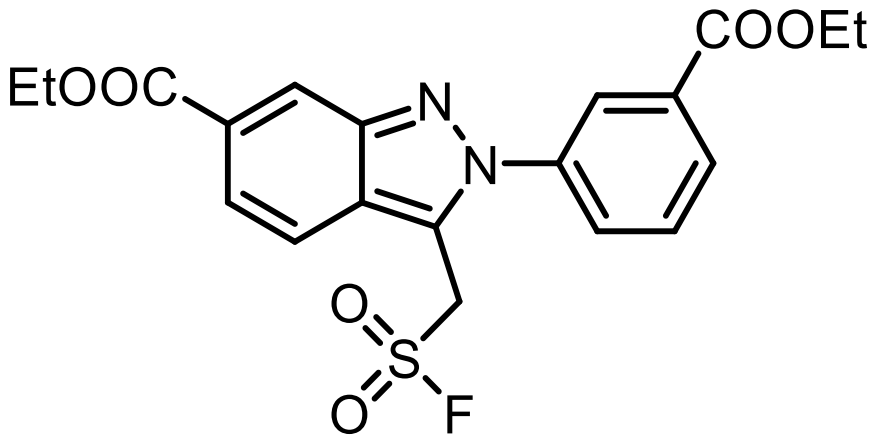

3i

63.9 mg (74%); Yellow solid; ^{1}H NMR (600 MHz, $CDCl_3$) δ 8.61 (t, *J* = 1.2 Hz, 1H), 8.29 (dt, *J* = 7.9, 1.3 Hz, 1H), 8.27 (t, *J* = 1.8 Hz, 1H), 7.92 (dd, *J* = 8.9, 1.3 Hz, 1H), 7.85 – 7.83 (m, 1H), 7.79 (d, *J* = 9.0 Hz, 1H), 7.72 (t, *J* = 7.9 Hz, 1H), 5.09 (d, *J* = 2.8 Hz, 2H), 4.46 – 4.43 (m, 2H), 4.43 – 4.40 (m, 2H), 1.44 (t, *J* = 7.1 Hz, 3H), 1.40 (t, *J* = 7.1 Hz, 3H) ppm; ^{13}C NMR (150 MHz, $CDCl_3$) δ 166.49, 165.06, 148.42, 138.54, 132.59, 131.55, 130.76, 130.26, 130.14, 127.42, 124.81, 124.18, 122.28, 120.32, 119.28, 61.91, 61.49, 47.74 (d, *J* = 21.4 Hz), 14.46, 14.36 ppm; ^{19}F NMR (376 MHz, $CDCl_3$) δ 56.04 ppm; FT-IR ($CHCl_3$, film) υ 2960, 2923, 2855, 1701, 1408, 1368, 1322, 1206, 1080, 865 cm^{-1}; HRMS (EI+) calcd for $C_{20}H_{19}FN_2O_6S$ $[M]^+$ 434.0948, found 434.0950.

(2-Phenyl-2*H*-indazol-3-yl)methanesulfonyl fluoride (3j)

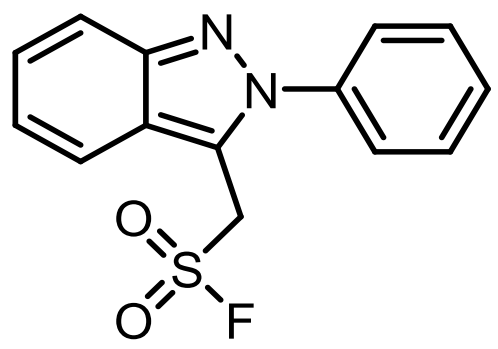

3j

32.0 mg (55%); Yellow solid; ^{1}H NMR (600 MHz, $CDCl_3$) δ 7.83 (dt, *J* = 8.8, 1.0 Hz, 1H), 7.74 (dd, *J* = 8.6, 1.1 Hz, 1H), 7.60 – 7.59 (m, 5H), 7.42 (ddd, *J* = 8.8, 6.7, 1.1 Hz, 1H), 7.28 (ddd, *J* = 8.5, 6.7, 1.0 Hz, 1H), 5.06 (d, *J* = 3.0 Hz, 2H) ppm; ^{13}C NMR (150 MHz, $CDCl_3$) δ 148.90, 138.53, 130.34, 129.90, 127.52, 126.71, 124.32, 122.83, 119.57, 119.17 (d, *J* = 1.7 Hz), 118.64, 47.92 (d, *J* = 21.4 Hz) ppm; ^{19}F NMR (376 MHz, $CDCl_3$) δ 55.90 ppm; FT-IR ($CHCl_3$, film) υ 2922, 2853, 1498, 1408, 1204, 812 cm^{-1}; HRMS (EI+) calcd for $C_{14}H_{11}FN_2O_2S$ $[M]^+$

290.0525, found 290.0525.

(5-Methyl-2-(*p*-tolyl)-2*H*-indazol-3-yl)methanesulfonyl fluoride (3k)

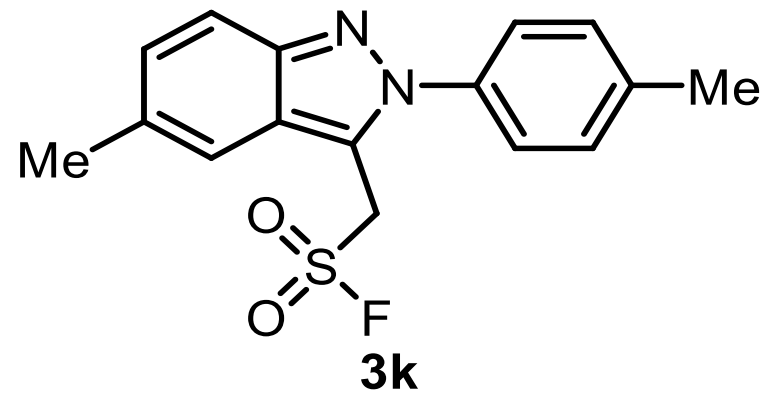

3k

39.6 mg (62%); Red solid; ^{1}H NMR (600 MHz, $CDCl_3$) δ 7.71 (d, *J* = 9.0 Hz, 1H), 7.46 (d, *J* = 5.1 Hz, 2H), 7.44 (s, 1H), 7.37 (d, *J* = 8.3 Hz, 2H), 7.24 (dd, *J* = 8.9, 1.7 Hz, 1H), 5.02 (d, *J* = 3.0 Hz, 2H), 2.49 (s, 3H), 2.48 (s, 3H) ppm; ^{13}C NMR (150 MHz, $CDCl_3$) δ 147.81, 140.48, 136.11, 133.95, 130.39, 130.37, 126.48, 123.12, 118.50, 118.25, 117.10, 48.00 (d, *J* = 20.9 Hz), 22.17, 21.42 ppm; ^{19}F NMR (376 MHz, $CDCl_3$) δ 55.77 ppm; FT-IR ($CHCl_3$, film) υ 2962, 2921, 2859, 1604, 1511, 1406, 1204, 1037, 865 cm^{-1}; HRMS (EI+) calcd for $C_{16}H_{15}FN_2O_2S$ $[M]^+$ 318.0838, found 318.0834.

(5-(Trifluoromethyl)-2-(4-(trifluoromethyl)phenyl)-2*H*-indazol-3-yl)methanesulfonyl fluoride (3l)

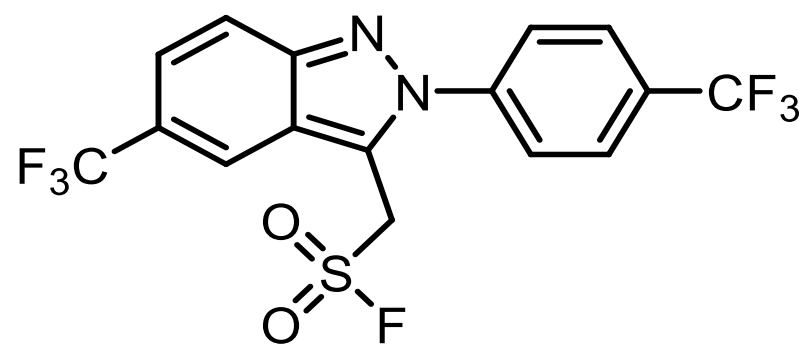

3l

36.3 mg (43%); White solid; ^{1}H NMR (600 MHz, $CDCl_3$) δ 8.12 (s, 1H), 7.95 (d, *J* = 9.1 Hz, 1H), 7.92 (d, *J* = 8.5 Hz, 2H), 7.79 (d, *J* = 8.3 Hz, 2H), 7.61 (dd, *J* = 9.1, 1.7 Hz, 1H), 5.10 (d, *J* = 2.2 Hz, 2H) ppm; ^{13}C NMR (150 MHz, $CDCl_3$) δ 149.54, 141.01, 132.95 (q, *J* = 33.2 Hz), 127.36 (q, *J* = 3.7 Hz), 127.18, 126.94, 125.17, 124.36, 124.00 (q, *J* = 2.9 Hz), 123.37, 122.56, 122.07, 121.80, 120.13, 118.19 (q, *J* = 5.1 Hz), 47.60 (d, *J* = 22.5 Hz) ppm; ^{19}F NMR (376 MHz, $CDCl_3$) δ 56.31, -62.31, -62.73 ppm; FT-IR ($CHCl_3$, film) υ 2922, 1614, 1423, 1390, 1319, 1202, 1156, 1115, 1063, 891, 856, 804 cm^{-1}; HRMS (EI+) calcd for $C_{16}H_9F_7N_2O_2S$ $[M]^+$ 426.0273, found 426.0270.

(5-Chloro-2-(4-chlorophenyl)-2*H*-indazol-3-yl)methanesulfonyl fluoride (3m)

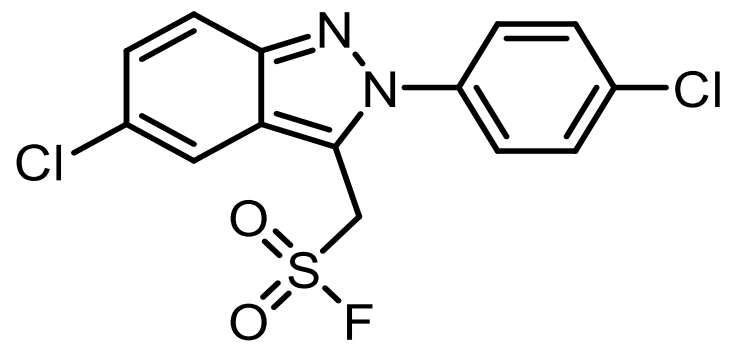

3m

51.0 mg (71%); Yellow solid; ^{1}H NMR (600 MHz, $CDCl_3$) δ 7.74 (d, J = 9.5 Hz, 1H), 7.71 (s, 1H), 7.59 – 7.56 (m, 2H), 7.54 – 7.52 (m, 2H), 7.35 (dd, J = 9.2, 1.9 Hz, 1H), 4.99 (d, J = 2.4 Hz, 2H) ppm; ^{13}C NMR (150 MHz, $CDCl_3$) δ 147.35, 136.80, 136.69, 130.46, 130.21, 129.33, 127.86, 123.36, 120.21, 119.44, 117.91, 47.68 (d, J = 22.0 Hz) ppm; ^{19}F NMR (376 MHz, $CDCl_3$) δ 56.12 ppm; FT-IR ($CHCl_3$, film) υ 2919, 2845, 1496, 1405, 1202, 1092, 1013, 840 cm^{-1}; HRMS (EI+) calcd for $C_{14}H_9Cl_2FN_2O_2S$ $[M]^+$ 357.9746, found 357.9748.

(5-Bromo-2-(4-bromophenyl)-2*H*-indazol-3-yl)methanesulfonyl fluoride (3n)

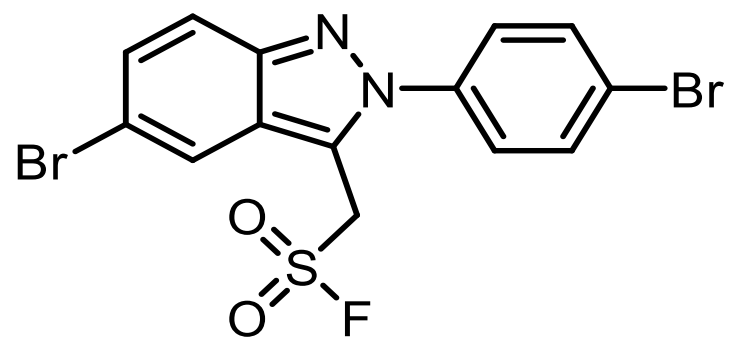

3n

56.5 mg (63%); Yellow solid; ^{1}H NMR (600 MHz, $CDCl_3$) δ 7.91 (s, 1H), 7.76 – 7.73 (m, 2H), 7.69 (d, J = 9.1 Hz, 1H), 7.49 – 7.46 (m, 3H), 4.99 (d, J = 2.3 Hz, 2H) ppm; ^{13}C NMR (150 MHz, $CDCl_3$) δ 147.48, 137.20, 133.24, 131.65, 128.11, 124.93, 124.12, 121.35, 120.38, 119.23, 118.41, 47.71 (d, J = 22.0 Hz) ppm; ^{19}F NMR (376 MHz, $CDCl_3$) δ 56.17 ppm; FT-IR ($CHCl_3$, film) υ 2921, 2847, 1488, 1409, 1256, 1200, 1075, 1009, 835 cm^{-1}; HRMS (EI+) calcd for $C_{14}H_9Br_2FN_2O_2S$ $[M]^+$ 445.8736, found 445.8737.

(5-Acetyl-2-(4-acetylphenyl)-2*H*-indazol-3-yl)methanesulfonyl fluoride (3o)

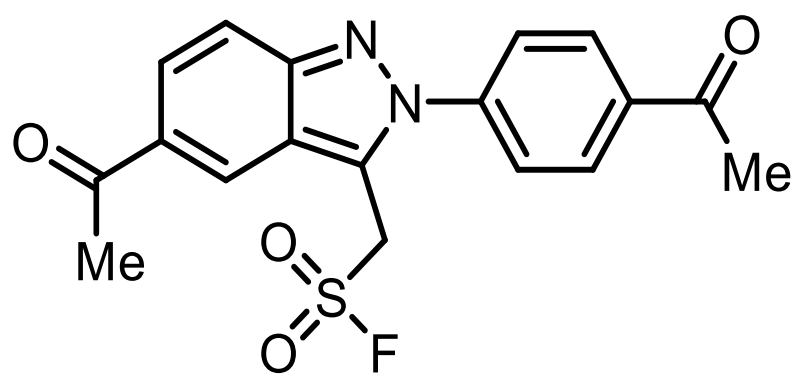

3o

61.6 mg (82%); White solid; ^{1}H NMR (600 MHz, $CDCl_3$) δ 8.45 (s, 1H), 8.21 (d, *J* = 8.5 Hz, 2H), 8.03 (dd, *J* = 9.1, 1.6 Hz, 1H), 7.85 (d, *J* = 9.2 Hz, 1H), 7.76 (d, *J* = 8.5 Hz, 2H), 5.16 (d, *J* = 2.0 Hz, 2H), 2.70 (s, 3H), 2.70 (s, 3H) ppm; ^{13}C NMR (150 MHz, $CDCl_3$) δ 197.32, 196.69, 150.32, 141.67, 138.53, 133.87, 131.41, 130.05, 126.77, 126.53, 122.62, 122.53, 119.01, 47.72 (d, *J* = 22.0 Hz), 26.97, 26.75 ppm; ^{19}F NMR (376 MHz, $CDCl_3$) δ 56.31 ppm; FT-IR ($CHCl_3$, film) υ 2923, 2850, 1684, 1665, 1602, 1489, 1408, 1265, 1198, 809 cm^{-1}; HRMS (EI+) calcd for $C_{18}H_{15}FN_2O_4S$ $[M]^+$ 374.0737, found 374.0737.

Ethyl 2-(4-(ethoxycarbonyl)phenyl)-3-((fluorosulfonyl)methyl)-2*H*-indazole-5-carboxylate (3p)

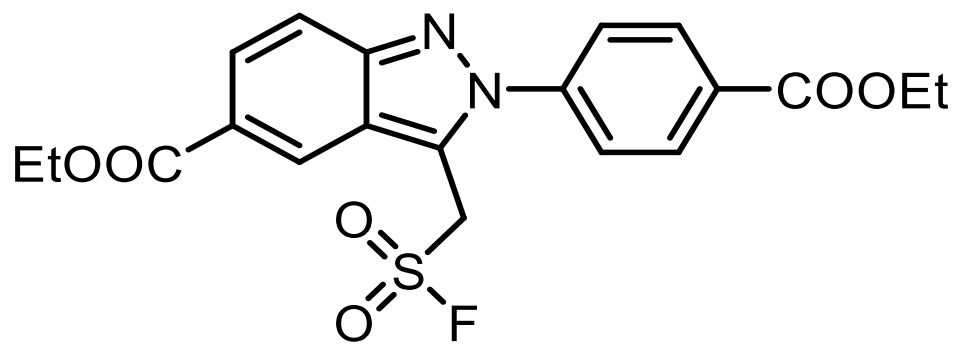

3p

57.3 mg (66%); White solid; ^{1}H NMR (600 MHz, $CDCl_3$) δ 8.57 (s, 1H), 8.29 (d, *J* = 8.5 Hz, 2H), 8.04 (dd, *J* = 9.1, 1.6 Hz, 1H), 7.83 (d, *J* = 9.1 Hz, 1H), 7.71 (d, *J* = 8.5 Hz, 2H), 5.14 (d, *J* = 2.6 Hz, 2H), 4.43 (dq, *J* = 10.8, 7.1 Hz, 4H), 1.43 (td, *J* = 7.2, 2.5 Hz, 6H) ppm; ^{13}C NMR (150 MHz, $CDCl_3$) δ 166.38, 165.30, 150.25, 141.64, 132.50, 131.30, 127.64, 126.96, 126.48, 123.36, 122.56, 122.30, 118.61, 61.84, 61.45, 47.59 (d, *J* = 22.0 Hz), 14.49, 14.41 ppm; ^{19}F NMR (376 MHz, $CDCl_3$) δ 56.33 ppm; FT-IR ($CHCl_3$, film) υ 2922, 1719, 1697, 1605, 1402, 1270, 1196, 1105, 815 cm^{-1}; HRMS (EI+) calcd for $C_{20}H_{19}FN_2O_6S$ $[M]^+$ 434.0948, found 434.0944.

(*E*)-2-(3-Methyl-2-(*o*-tolyldiazenyl)phenyl)ethane-1-sulfonyl fluoride (3aa)

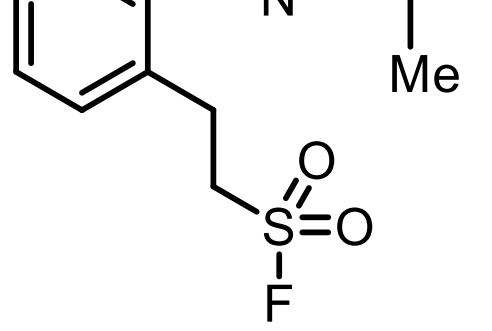

3aa

52.6 mg (82%); Red solid; ^{1}H NMR (600 MHz, $CDCl_3$) δ 7.61 (dd, *J* = 8.0, 1.6 Hz, 1H), 7.45 – 7.40 (m, 2H), 7.34 – 7.32 (m, 1H), 7.29 (dd, *J* = 5.1, 3.8 Hz, 2H), 7.25 (q, *J* = 3.4 Hz, 1H), 3.75 – 3.72 (m, 2H), 3.47 – 3.44 (m, 2H), 2.74 (s, 3H), 2.48 (s, 3H) ppm; ^{13}C NMR (150 MHz, $CDCl_3$) δ 151.28, 150.25, 138.63, 131.91, 131.77, 131.75, 131.52, 131.27, 129.44, 128.73, 126.83, 115.02, 52.13 (d, *J* = 14.8 Hz), 27.29, 20.40, 18.10 ppm; FT-IR ($CHCl_3$, film) υ 3023, 2912, 1502, 1408, 1204, 869 cm^{-1}; HRMS (ESI-) calcd for $C_{16}H_{16}FN_2O_2S$ $[M-H]^-$ 319.0917, found 319.0921.

(2-(2,3-Dimethylphenyl)-6,7-dimethyl-2*H*-indazol-3-yl)methanesulfonyl fluoride (5a)

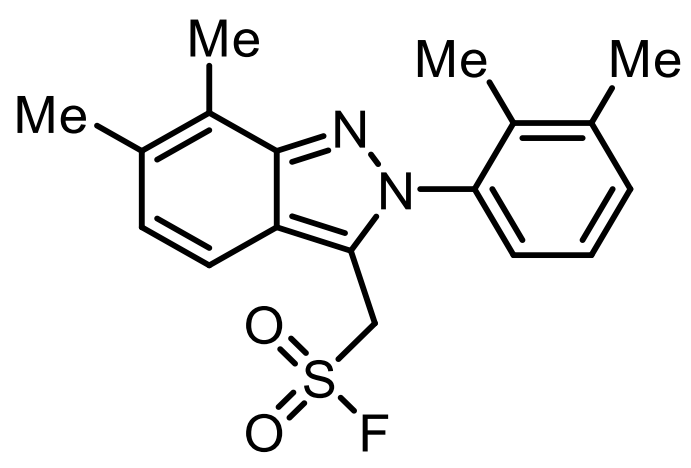

5a

44.2 mg (64%); Yellow solid; ^{1}H NMR (600 MHz, $CDCl_3$) δ 7.48 (d, *J* = 8.5 Hz, 1H), 7.36 (d, *J* = 7.5 Hz, 1H), 7.26 (t, *J* = 8.0 Hz, 1H), 7.20 (d, *J* = 7.7 Hz, 1H), 7.11 (d, *J* = 8.6 Hz, 1H), 4.96 (dd, *J* = 15.3, 2.9 Hz, 1H), 4.78 (dd, *J* = 15.3, 4.2 Hz, 1H), 2.58 (s, 3H), 2.42 (s, 3H), 2.36 (s, 3H), 1.83 (s, 3H) ppm; ^{13}C NMR (150 MHz, $CDCl_3$) δ 149.88, 139.26, 137.33, 135.17, 133.47, 132.00, 128.26, 126.40, 125.55, 125.30, 120.63, 120.16, 115.69, 47.67 (d, *J* = 20.9 Hz), 20.37, 19.38, 14.11, 13.22 ppm; ^{19}F NMR (376 MHz, $CDCl_3$) δ 55.54 ppm; FT-IR ($CHCl_3$, film) υ 2921, 1485, 1404, 1200, 1016, 842 cm^{-1}; HRMS (EI+) calcd for $C_{18}H_{19}FN_2O_2S$ $[M]^+$ 346.1151, found 346.1147.

(6-Fluoro-2-(3-fluoro-2-methylphenyl)-7-methyl-2*H*-indazol-3-yl)methanesulfonyl fluoride (5b)

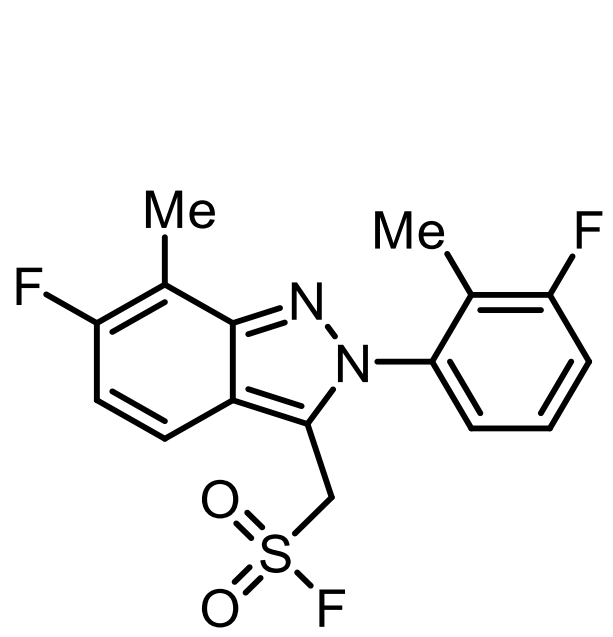

5b

51.3 mg (72%); Pale green solid; ^{1}H NMR (600 MHz, $CDCl_3$) δ 7.55 (dd, J = 9.2, 4.8 Hz, 1H), 7.40 – 7.37 (m, 1H), 7.30 (t, J = 8.7 Hz, 1H), 7.22 (d, J = 7.8 Hz, 1H), 7.09 (t, J = 9.4 Hz, 1H), 4.90 (d, J = 88.5 Hz, 2H), 2.54 (d, J = 2.0 Hz, 3H), 1.93 (d, J = 2.1 Hz, 3H) ppm; ^{13}C NMR (150 MHz, $CDCl_3$) δ 162.60, 160.95, 159.87, 158.26, 149.83 (d, J = 10.5 Hz), 138.40 (d, J = 6.4 Hz), 127.69 (d, J = 9.5 Hz), 124.93 (d, J = 19.4 Hz), 123.57 (d, J = 3.6 Hz), 117.82 (d, J = 5.0 Hz), 117.71 (d, J = 7.4 Hz), 116.55 (d, J = 29.8 Hz), 111.97 (d, J = 20.0 Hz), 47.39 (d, J = 22.0 Hz), 9.82 (d, J = 4.1 Hz), 8.89 (d, J = 3.8 Hz) ppm; ^{19}F NMR (376 MHz, $CDCl_3$) δ 55.77, -112.29, -118.85 ppm; FT-IR ($CHCl_3$, film) υ 2923, 2852, 1583, 1409, 1245, 1199, 792 cm^{-1}; HRMS (EI+) calcd for $C_{16}H_{13}F_3N_2O_2S$ $[M]^+$ 354.0650, found 354.0651.

(6-Chloro-2-(3-chloro-2-methylphenyl)-7-methyl-2*H*-indazol-3-yl)methanesulfonyl fluoride (5c)

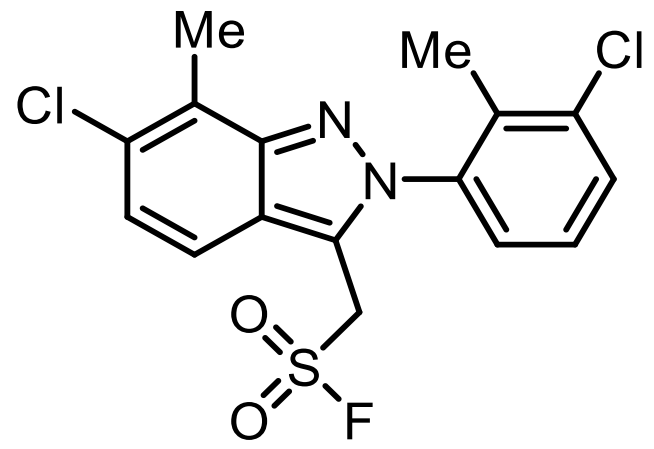

5c

42.5 mg (55%); Pale green solid; ^{1}H NMR (600 MHz, $CDCl_3$) δ 7.63 (dd, J = 7.7, 1.7 Hz, 1H), 7.51 (d, J = 8.9 Hz, 1H), 7.37 (d, J = 7.7 Hz, 1H), 7.35 – 7.32 (m, 1H), 7.27 – 7.25 (m, 1H), 4.98 (d, J = 15.2 Hz, 1H), 4.77 (dd, J = 15.4, 3.9 Hz, 1H), 2.68 (s, 3H), 1.98 (s, 3H) ppm; ^{13}C NMR (150 MHz, $CDCl_3$) δ 149.77, 138.13, 136.56, 135.40, 131.90, 127.53, 126.87, 126.56, 126.31, 121.25, 120.53, 117.32, 47.37 (d, J = 22.0 Hz), 15.14, 13.99 ppm; ^{19}F NMR (376 MHz, $CDCl_3$) δ 55.82 ppm; FT-IR ($CHCl_3$, film) υ 2922, 1484, 1414, 1384, 1207, 1100, 999, 886, 786, 754, 712 cm^{-1}; HRMS (EI+) calcd for $C_{16}H_{13}Cl_2FN_2O_2S$ $[M]^+$ 386.0059, found 386.0059.

(7-Fluoro-2-(2-fluoro-3-methylphenyl)-6-methyl-2*H*-indazol-3-yl)methanesulfonyl fluoride (5d)

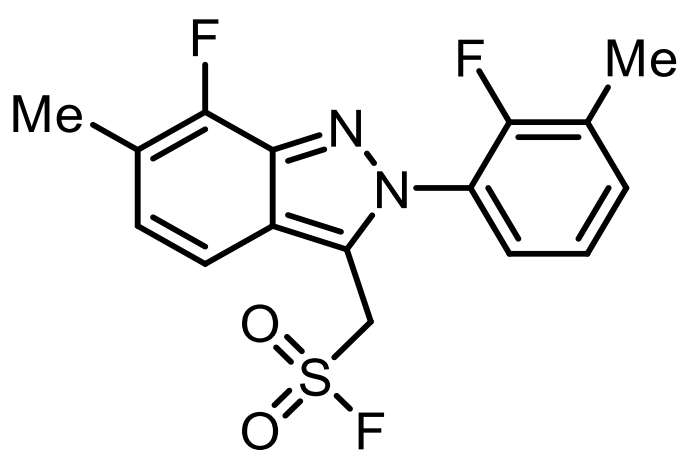

5d

49.7 mg (70%); Yellow solid; ^{1}H NMR (600 MHz, $CDCl_3$) δ 7.44 (q, *J* = 7.0 Hz, 2H), 7.39 (d, *J* = 8.6 Hz, 1H), 7.27 – 7.25 (m, 1H), 7.07 (dd, *J* = 8.6, 6.0 Hz, 1H), 4.97 (d, *J* = 3.6 Hz, 2H), 2.41 (d, *J* = 2.5 Hz, 3H), 2.39 (d, *J* = 2.0 Hz, 3H) ppm; ^{13}C NMR (150 MHz, $CDCl_3$) δ 156.00, 154.33, 151.10, 149.41, 141.19 (d, *J* = 17.5 Hz), 134.05 (d, *J* = 5.0 Hz), 128.62 (d, *J* = 3.3 Hz), 127.22, 127.01 (d, *J* = 15.6 Hz), 125.93 (d, *J* = 12.8 Hz), 124.77 (d, *J* = 4.7 Hz), 123.93 (d, *J* = 4.9 Hz), 121.76 (d, *J* = 3.1 Hz), 120.01 (d, *J* = 12.6 Hz), 114.28 (d, *J* = 5.4 Hz), 47.49 (d, *J* = 22.2 Hz), 14.69 (d, *J* = 3.5 Hz), 14.15 (d, *J* = 3.8 Hz) ppm; ^{19}F NMR (376 MHz, $CDCl_3$) δ 55.53, -126.77, -133.38 ppm; FT-IR ($CHCl_3$, film) υ 2924, 2850, 1545, 1499, 1406, 1221, 1208, 1068, 859 cm^{-1}; HRMS (EI+) calcd for $C_{16}H_{13}F_3N_2O_2S$ $[M]^+$ 354.0650, found 354.0648.

(2-(2,4-Dimethylphenyl)-5,7-dimethyl-2*H*-indazol-3-yl)methanesulfonyl fluoride (5e)

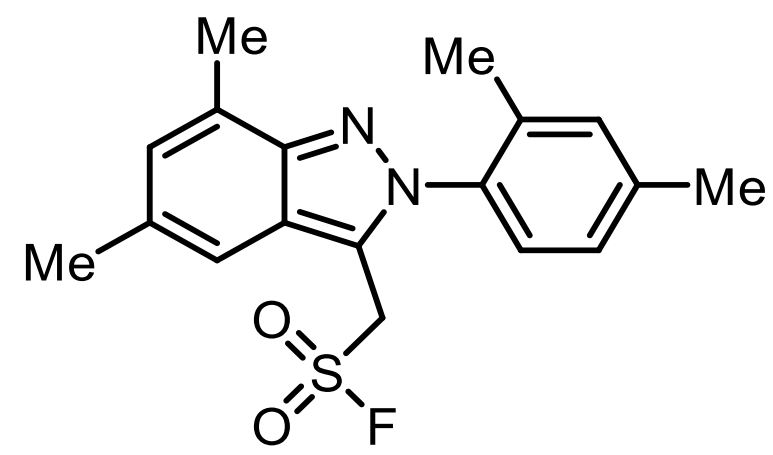

5e

59.0 mg (85%); Yellow gummy solid; ^{1}H NMR (600 MHz, $CDCl_3$) δ 7.22 (s, 1H), 7.16 (d, *J* = 7.9 Hz, 1H), 7.11 (s, 1H), 7.07 (d, *J* = 7.9 Hz, 1H), 6.93 (s, 1H), 4.77 (d, *J* = 76.7 Hz, 2H), 2.54 (s, 3H), 2.36 (s, 3H), 2.33 (s, 3H), 1.87 (s, 3H) ppm; ^{13}C NMR (150 MHz, $CDCl_3$) δ 148.14, 140.78, 136.07, 134.77, 134.06, 132.23, 129.22, 128.43, 127.63, 127.59, 122.31, 119.16, 114.54, 47.64 (d, *J* = 20.9 Hz), 22.09, 21.34, 17.06, 17.01 ppm; ^{19}F NMR (376 MHz, $CDCl_3$) δ 55.52 ppm; FT-IR ($CHCl_3$, film) υ 2960, 2917, 2856, 1508, 1403, 1268, 1209, 813 cm^{-1}; HRMS (EI+) calcd for $C_{18}H_{19}FN_2O_2S$ $[M]^+$ 346.1151, found 346.1150.

(5-Fluoro-2-(4-fluoro-2-methylphenyl)-7-methyl-2*H*-indazol-3-yl)methanesulfonyl

fluoride (5f)

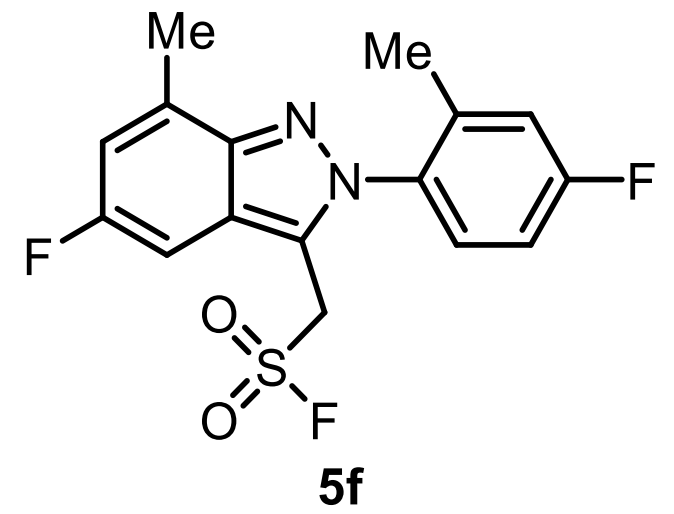

5f

46.9 mg (66%); Brown solid; ^{1}H NMR (600 MHz, $CDCl_3$) δ 7.38 (dd, *J* = 8.5, 5.1 Hz, 1H), 7.14 (d, *J* = 9.0 Hz, 2H), 7.10 (td, *J* = 8.1, 3.0 Hz, 1H), 7.00 (dd, *J* = 9.8, 1.1 Hz, 1H), 4.84 (d, *J* = 94.0 Hz, 2H), 2.65 (s, 3H), 2.00 (s, 3H) ppm; ^{13}C NMR (150 MHz, $CDCl_3$) δ 163.57 (d, *J* = 250.0 Hz), 159.91 (d, *J* = 242.8 Hz), 146.80, 139.42 (d, *J* = 9.3 Hz), 133.15 (d, *J* = 3.3 Hz), 132.05 (d, *J* = 9.9 Hz), 129.76 (d, *J* = 9.3 Hz), 121.57 (d, *J* = 12.1 Hz), 120.68 (d, *J* = 8.8 Hz), 118.48 (d, *J* = 22.0 Hz), 118.20 (d, *J* = 28.0 Hz), 114.18 (d, *J* = 22.5 Hz), 99.15 (d, *J* = 24.7 Hz), 47.50 (d, *J* = 21.4 Hz), 17.43, 17.03 ppm; ^{19}F NMR (376 MHz, $CDCl_3$) δ 55.72, -109.36, -115.45 ppm; FT-IR ($CHCl_3$, film) υ 2992, 2925, 2851, 1504, 1408, 1273, 1225, 882 cm^{-1}; HRMS (EI+) calcd for $C_{16}H_{13}F_3N_2O_2S$ $[M]^+$ 354.0650, found 354.0646.

(5-Chloro-2-(4-chloro-2-methylphenyl)-7-methyl-2*H*-indazol-3-yl)methanesulfonyl fluoride (5g)

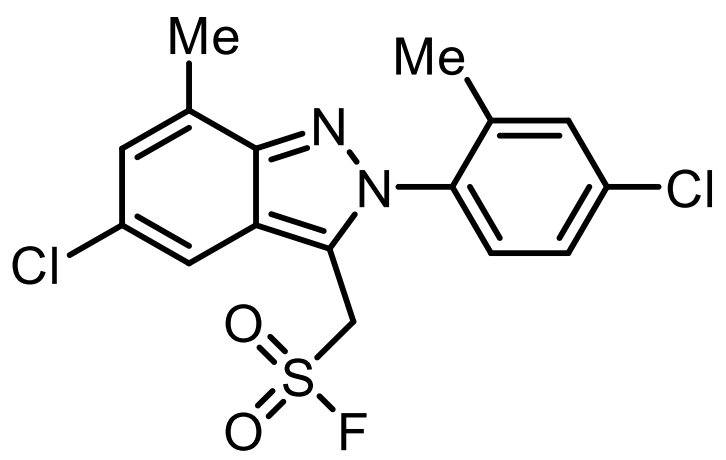

5g

64.0 mg (82%); Pale yellow solid; ^{1}H NMR (600 MHz, $CDCl_3$) δ 7.55 (d, *J* = 1.9 Hz, 1H), 7.43 (d, *J* = 2.3 Hz, 1H), 7.38 (dd, *J* = 8.3, 2.4 Hz, 1H), 7.32 (d, *J* = 8.4 Hz, 1H), 7.14 (t, *J* = 1.6 Hz, 1H), 4.87 (s, 2H), 2.63 (s, 3H), 1.99 (s, 3H) ppm; ^{13}C NMR (150 MHz, $CDCl_3$) δ 147.87, 138.45, 136.87, 135.47, 131.75, 131.04, 130.40, 129.02, 127.93, 127.34, 122.41, 120.12, 115.23, 47.33 (d, *J* = 21.9 Hz), 17.18, 16.83 ppm; ^{19}F NMR (376 MHz, $CDCl_3$) δ 55.86 ppm; FT-IR ($CHCl_3$, film) υ 2918, 2852, 1494, 1411, 1347, 1202, 888, 792 cm^{-1}; HRMS (EI+) calcd

for $C_{16}H_{13}Cl_2FN_2O_2S$ $[M]^+$ 386.0059, found 386.0054.

(7-Fluoro-2-(2-fluoro-4-methylphenyl)-5-methyl-2*H*-indazol-3-yl)methanesulfonyl fluoride (5h)

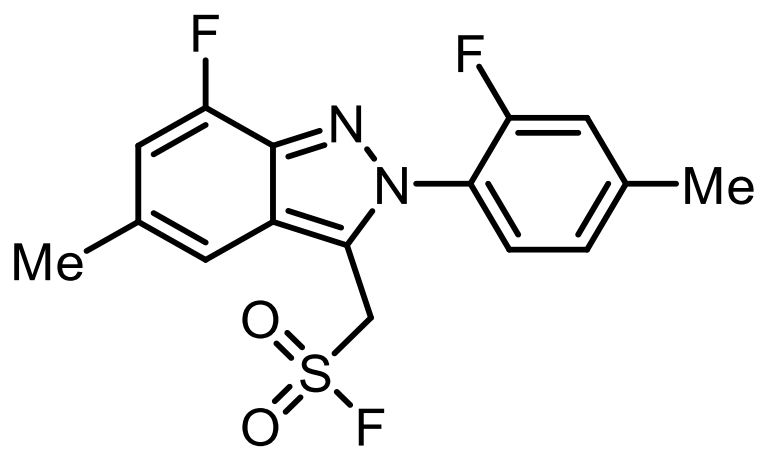

5h

48.5 mg (68%); Yellow solid; ^{1}H NMR (600 MHz, $CDCl_3$) δ 7.50 (t, *J* = 7.9 Hz, 1H), 7.23 (s, 1H), 7.16 (d, *J* = 8.1 Hz, 1H), 7.11 (d, *J* = 10.8 Hz, 1H), 6.89 (d, *J* = 11.7 Hz, 1H), 4.96 (d, *J* = 3.5 Hz, 2H), 2.47 (s, 3H), 2.46 (s, 3H) ppm; ^{13}C NMR (150 MHz, $CDCl_3$) δ 156.96, 155.29, 153.56, 151.85, 143.87 (d, *J* = 7.7 Hz), 139.74 (d, *J* = 17.0 Hz), 135.00 (d, *J* = 5.3 Hz), 129.43, 126.16 (d, *J* = 3.4 Hz), 125.45 (d, *J* = 5.5 Hz), 123.38 (d, *J* = 12.2 Hz), 121.07 (d, *J* = 3.5 Hz), 117.29 (d, *J* = 18.9 Hz), 113.14, 113.02 (d, *J* = 2.9 Hz), 112.99, 47.43 (d, *J* = 18.9 Hz), 22.16, 21.57 ppm; ^{19}F NMR (376 MHz, $CDCl_3$) δ 55.50, -123.43, -128.35 (d, *J* = 17.9 Hz) ppm; FT-IR ($CHCl_3$, film) υ 2923, 2852, 1557, 1521, 1410, 1208, 819 cm^{-1}; HRMS (EI+) calcd for $C_{16}H_{13}F_3N_2O_2S$ $[M]^+$ 354.0650, found 354.0650.

(7-Bromo-2-(2-bromo-4-methylphenyl)-5-methyl-2H-indazol-3-yl)methanesulfonyl fluoride (5i)

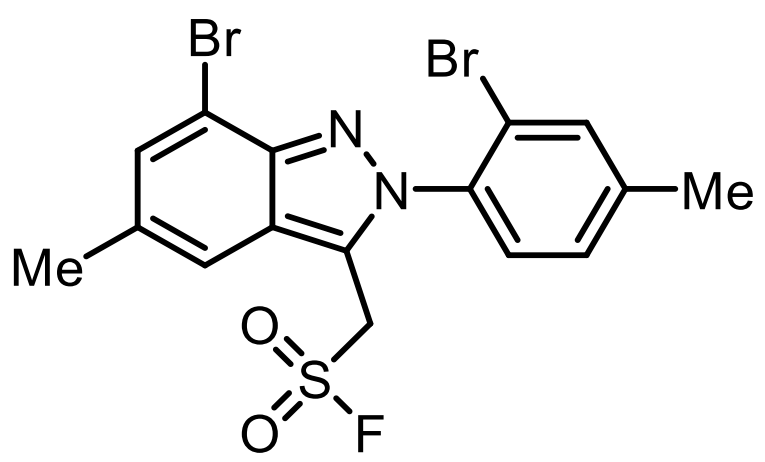

5i

20.4 mg (21%); Pale yellow solid; ^{1}H NMR (600 MHz, $CDCl_3$) δ 7.57 (s, 1H), 7.53 (d, *J* = 8.0 Hz, 1H), 7.51 (s, 1H), 7.43 (s, 1H), 7.34 (d, *J* = 8.0 Hz, 1H), 5.07 (d, *J* = 15.5 Hz, 1H), 4.66 (dd, *J* = 15.5, 5.4 Hz, 1H), 2.47 (d, *J* = 4.8 Hz, 6H) ppm; ^{13}C NMR (150 MHz, $CDCl_3$) δ 146.83, 143.34, 135.19, 134.70, 133.80, 133.21, 130.53, 129.64, 123.20, 121.32, 120.91, 116.80,

111.95, 47.85 (d, J = 21.4 Hz), 21.84, 21.27 ppm; ^{19}F NMR (376 MHz, $CDCl_3$) δ 55.66 ppm; FT-IR ($CHCl_3$, film) υ 2954, 2918, 2850, 1406, 1267, 1204, 1040, 816 cm^{-1}; HRMS (EI) calcd for $C_{16}H_{13}Br_2FN_2O_2S$ $[M]^+$ 473.9049, found 473.9047.

(4-Fluoro-2-(5-fluoro-2-methylphenyl)-7-methyl-2*H*-indazol-3-yl)methanesulfonyl fluoride (5j)

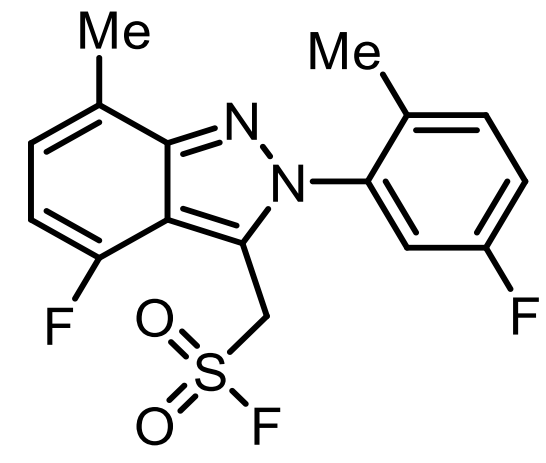

5j

14.2 mg (20%); Brown solid; ^{1}H NMR (600 MHz, $CDCl_3$) δ 7.40 (dd, J = 8.8, 5.7 Hz, 1H), 7.24 (dd, J = 8.3, 2.7 Hz, 1H), 7.18 (dd, J = 8.1, 2.7 Hz, 1H), 7.06 (ddd, J = 7.6, 4.9, 1.3 Hz, 1H), 6.80 (dd, J = 10.7, 7.6 Hz, 1H), 5.25 – 4.80 (m, 2H), 2.59 (s, 3H), 2.00 (s, 3H) ppm; ^{13}C NMR (150 MHz, $CDCl_3$) δ 160.89 (d, J = 246.7 Hz), 153.61 (d, J = 249.5 Hz), 150.71 (d, J = 5.0 Hz), 137.36 (d, J = 9.3 Hz), 132.94 (d, J = 8.2 Hz), 132.22 (d, J = 3.3 Hz), 126.12 (d, J = 6.6 Hz), 124.91 (d, J = 5.5 Hz), 119.57 (d, J = 6.0 Hz), 118.20 (d, J = 20.9 Hz), 115.48 (d, J = 24.2 Hz), 113.90 (d, J = 18.7 Hz), 107.27 (d, J = 17.0 Hz), 47.68 (d, J = 18.7 Hz), 16.68, 16.52 ppm; ^{19}F NMR (376 MHz, $CDCl_3$) δ 55.15 (d, J = 22.6 Hz), -114.12, -126.05 ppm; FT-IR ($CHCl_3$, film) υ 2923, 2854, 1573, 1508, 1409, 1389, 1246, 1206, 1160, 866, 804 cm^{-1}; HRMS (EI+) calcd for $C_{16}H_{13}F_3N_2O_2S$ $[M]^+$ 354.0650, found 354.0650.

(7-Fluoro-2-(2-fluoro-5-methylphenyl)-4-methyl-2*H*-indazol-3-yl)methanesulfonyl fluoride (5k)

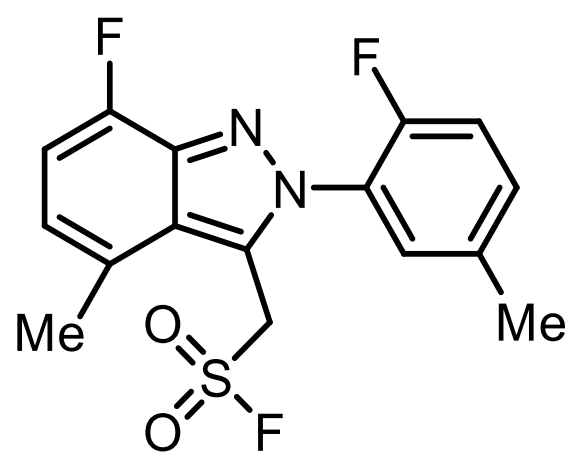

5k

24.8 mg (35%); White solid; ^{1}H NMR (600 MHz, $CDCl_3$) δ 7.48 (dd, J = 7.0, 2.6 Hz, 1H), 7.39

– 7.36 (m, 1H), 7.20 (dd, J = 9.8, 8.5 Hz, 1H), 6.93 – 6.91 (m, 1H), 6.89 – 6.86 (m, 1H), 5.16 (s, 2H), 2.68 (s, 3H), 2.43 (s, 3H) ppm; ^{13}C NMR (150 MHz, $CDCl_3$) δ 153.95 (d, J = 247.8 Hz), 151.78 (d, J = 253.9 Hz), 140.99 (d, J = 17.0 Hz), 135.72 (d, J = 3.8 Hz), 132.92 (d, J = 7.2 Hz), 130.30, 125.61 (d, J = 12.6 Hz), 125.37 (d, J = 5.0 Hz), 124.81 (d, J = 4.4 Hz), 124.45 (d, J = 5.5 Hz), 122.09 (d, J = 3.3 Hz), 116.33 (d, J = 19.2 Hz), 109.97 (d, J = 15.4 Hz), 47.99 (dd, J = 20.9, 3.8 Hz), 20.63, 18.80 ppm; ^{19}F NMR (376 MHz, $CDCl_3$) δ 55.52, -127.88, -131.84 ppm; FT-IR ($CHCl_3$, film) υ 2918, 2847, 1541, 1513, 1420, 1205, 1018, 869, 818 cm^{-1}; HRMS (EI+) calcd for $C_{16}H_{13}F_3N_2O_2S$ $[M]^+$ 354.0650, found 354.0647.

(5-Fluoro-2-(4-fluoro-3-methylphenyl)-6-methyl-2*H*-indazol-3-yl)methanesulfonyl fluoride (5l)

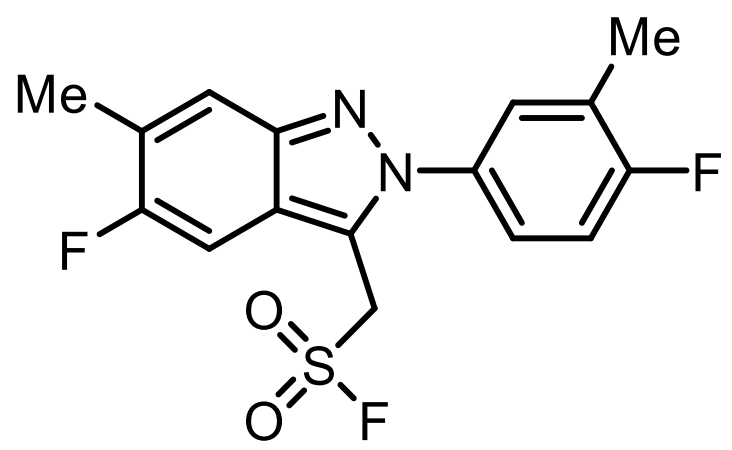

5l

43.3 mg (61%); Pale yellow solid; ^{1}H NMR (600 MHz, $CDCl_3$) δ 7.58 (d, J = 6.7 Hz, 1H), 7.42 (dd, J = 6.6, 2.9 Hz, 1H), 7.38 – 7.35 (m, 1H), 7.26 (d, J = 9.7 Hz, 1H), 7.20 (t, J = 8.8 Hz, 1H), 4.97 (d, J = 2.8 Hz, 2H), 2.42 (d, J = 1.4 Hz, 3H), 2.37 (d, J = 2.2 Hz, 3H) ppm; ^{13}C NMR (150 MHz, $CDCl_3$) δ 162.01 (d, J = 247.8 Hz), 159.56 (d, J = 244.5 Hz), 146.40, 134.11 (d, J = 3.3 Hz), 129.95 (d, J = 6.0 Hz), 129.35 (d, J = 23.6 Hz), 127.26 (d, J = 19.2 Hz), 125.62 (d, J = 8.8 Hz), 121.29 (d, J = 12.1 Hz), 119.64 (d, J = 5.5 Hz), 119.53 (d, J = 8.3 Hz), 116.31 (d, J = 24.2 Hz), 101.39 (d, J = 26.9 Hz), 47.90 (d, J = 21.4 Hz), 16.33 (d, J = 4.9 Hz), 14.74 (d, J = 3.3 Hz) ppm; ^{19}F NMR (376 MHz, $CDCl_3$) δ 55.81, -113.73, -118.61 ppm; FT-IR ($CHCl_3$, film) υ 2960, 2919, 2900, 1586, 1506, 1408, 1207, 1172, 854, 830, 794, 761 cm^{-1}; HRMS (EI+) calcd for $C_{16}H_{13}F_3N_2O_2S$ $[M]^+$ 354.0650, found 354.0650.

(5-Chloro-2-(4-chloro-3-methylphenyl)-6-methyl-2*H*-indazol-3-yl)methanesulfonyl fluoride (5m)

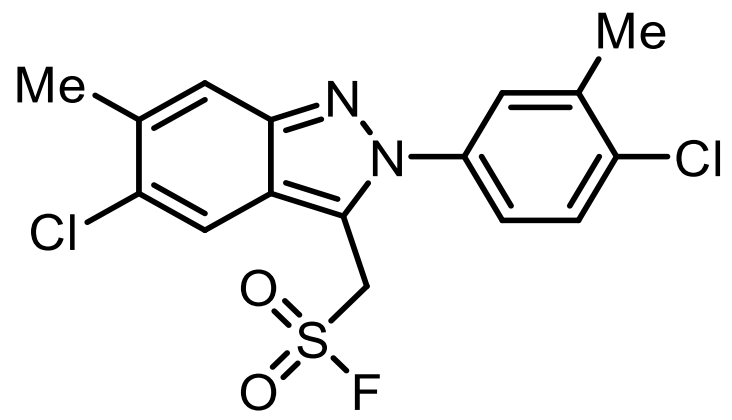

5m

53.6 mg (69%); Yellow solid; ^{1}H NMR (600 MHz, $CDCl_3$) δ 7.74 (s, 1H), 7.64 (s, 1H), 7.55 (d, J = 8.5 Hz, 1H), 7.47 (d, J = 2.6 Hz, 1H), 7.35 (dd, J = 8.4, 2.6 Hz, 1H), 4.99 (d, J = 2.6 Hz, 2H), 2.51 (d, J = 1.3 Hz, 3H), 2.47 (s, 3H) ppm; ^{13}C NMR (150 MHz, $CDCl_3$) δ 148.01, 138.50, 136.73, 136.65, 136.53, 132.43, 130.39, 128.96, 124.96, 122.18, 118.95, 118.22, 118.21, 47.83 (d, J = 21.9 Hz), 21.53, 20.35 ppm; ^{19}F NMR (376 MHz, $CDCl_3$) δ 56.02 ppm; FT-IR ($CHCl_3$, film) υ 2957, 2921, 2853, 1488, 1409, 1203, 1051, 826, 796 cm^{-1}; HRMS (EI+) calcd for $C_{16}H_{13}Cl_2FN_2O_2S$ $[M]^+$ 386.0059, found 386.0057.

(2-(3,5-Dimethylphenyl)-4,6-dimethyl-2*H*-indazol-3-yl)methanesulfonyl fluoride (5n)

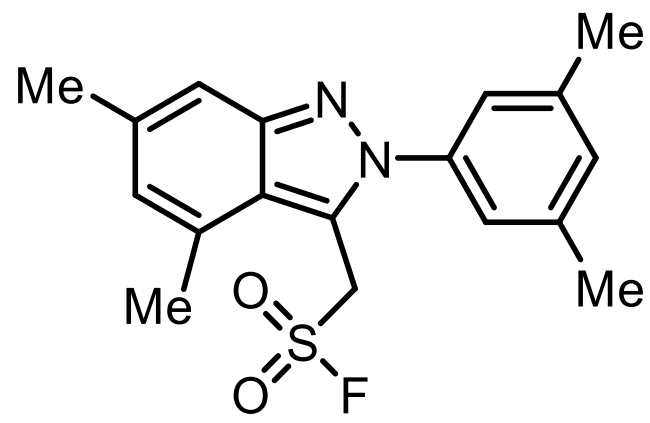

5n

19.9 mg (29%); Pale green solid; ^{1}H NMR (600 MHz, $CDCl_3$) δ 7.38 (s, 1H), 7.18 (s, 1H), 7.10 (s, 2H), 6.84 (s, 1H), 5.21 (d, J = 2.9 Hz, 2H), 2.68 (s, 3H), 2.43 (s, 3H), 2.40 (s, 6H) ppm; ^{13}C NMR (150 MHz, $CDCl_3$) δ 149.85, 139.74, 138.54, 137.30, 131.78, 130.95, 129.02, 127.85, 124.63, 123.80, 121.25, 119.34, 114.73, 48.93 (d, J = 19.8 Hz), 22.08, 21.32, 19.35 (d, J = 1.6 Hz) ppm; ^{19}F NMR (376 MHz, $CDCl_3$) δ 55.58 ppm; FT-IR ($CHCl_3$, film) υ 2918, 2852, 1595, 1483, 1407, 1205, 1166, 1083, 1032, 836, 805, 696 cm^{-1}; HRMS (EI+) calcd for $C_{18}H_{19}FN_2O_2S$ $[M]^+$ 346.1151, found 346.1153.

(2-(3,5-Dimethoxyphenyl)-4,6-dimethoxy-2*H*-indazol-3-yl)methanesulfonyl fluoride (5o)

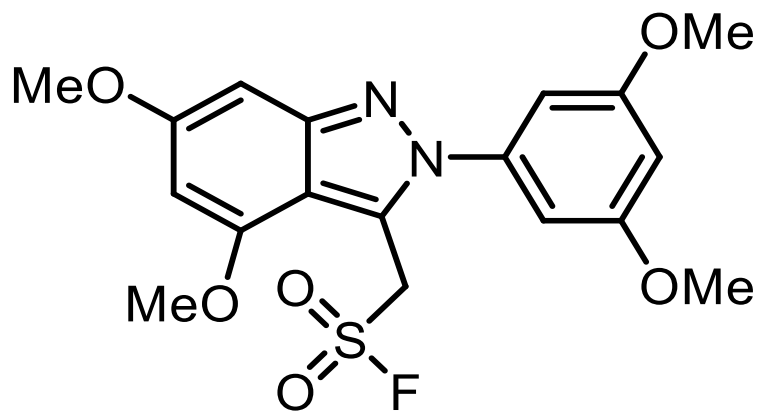

5o

35.5 mg (43%); White solid; ^{1}H NMR (600 MHz, $CDCl_3$) δ 6.68 (d, J = 2.2 Hz, 2H), 6.60 - 6.59 (m, 2H), 6.18 (d, J = 1.8 Hz, 1H), 5.24 (d, J = 4.4 Hz, 2H), 3.93 (s, 3H), 3.86 (s, 3H), 3.82 (s, 6H) ppm; ^{13}C NMR (150 MHz, $CDCl_3$) δ 161.34, 160.97, 153.80, 150.54, 139.96, 120.29, 112.72, 104.94, 102.26, 96.58, 87.80, 55.83, 55.75, 55.66, 48.39 (d, J = 19.8 Hz) ppm; ^{19}F NMR (376 MHz, $CDCl_3$) δ 55.83 ppm; FT-IR ($CHCl_3$, film) υ 3015, 2969, 2942, 2887, 2843, 1633, 1600, 1568, 1492, 1474, 1399, 1336, 1294, 1254, 1219, 1201, 1141, 1111, 1051, 1034, 820, 689 cm^{-1}; HRMS (EI+) calcd for $C_{18}H_{19}FN_2O_6S$ $[M]^+$ 410.0948, found 410.0946.

(4,6-Dichloro-2-(3,5-dichlorophenyl)-2*H*-indazol-3-yl)methanesulfonyl fluoride (5p)

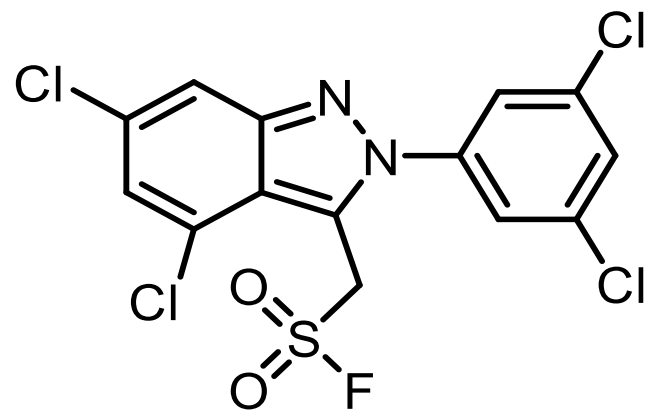

5p

31.2 mg (36%); Pale blue solid; ^{1}H NMR (600 MHz, $CDCl_3$) δ 7.71 (d, J = 1.6 Hz, 1H), 7.63 (t, J = 1.8 Hz, 1H), 7.48 (d, J = 1.9 Hz, 2H), 7.27 (d, J = 1.4 Hz, 1H), 5.38 (d, J = 3.6 Hz, 2H) ppm; ^{13}C NMR (150 MHz, $CDCl_3$) δ 149.58, 139.48, 136.46, 133.55, 131.18, 126.34, 125.85, 125.61, 121.67, 119.65, 116.75, 47.40 (d, J = 20.9 Hz) ppm; ^{19}F NMR (376 MHz, $CDCl_3$) δ 56.78 ppm; FT-IR ($CHCl_3$, film) υ 3092, 2919, 2847, 1623, 1579, 1535, 1470, 1437, 1407, 1207, 1099, 1068, 986, 862, 842, 806, 757 cm^{-1}; HRMS (EI+) calcd for $C_{14}H_7Cl_4FN_2O_2S$ $[M]^+$ 425.8966, found 425.8965.

(5,7-Dimethyl-2-(*o*-tolyl)-2*H*-indazol-3-yl)methanesulfonyl fluoride (**5q**) and (2-(2,4-dimethylphenyl)-7-methyl-2H-indazol-3-yl)methanesulfonyl fluoride (**5q'**): 56.0 mg (84%); **5q**:**5q'** = 1.3:1; Yellow solid; ^{1}H NMR (600 MHz, $CDCl_3$) δ 7.57 (d, J = 8.1 Hz, 1H), 7.50 - 7.46 (m, 1H), 7.41 (d, J = 7.5 Hz, 1H), 7.38 (d, J = 3.5 Hz, 2H), 7.32 (s, 1H), 7.26 (s, 1H), 7.25

(s, 1H), 7.22 (s, 1H), 7.22 – 7.16 (m, 2H), 7.03 (s, 1H), 4.89 (d, J = 74.5 Hz, 4H), 2.67 (s, 2H), 2.63 (s, 3H), 2.46 (s, 3H), 2.43 (s, 2H), 2.01 (s, 3H), 1.97 (s, 2H) ppm; ^{13}C NMR (150 MHz, $CDCl_3$) δ 149.17, 148.24, 140.95, 137.32, 136.57, 136.03, 134.70, 134.26, 132.30, 131.70, 130.69, 129.39, 128.93, 128.48, 127.94, 127.68, 127.63, 127.05, 126.23, 124.40, 122.40, 121.96, 120.22, 119.15, 116.56, 114.54, 47.72, 47.58, 22.14, 21.38, 17.18, 17.13, 17.12, 17.04 ppm; ^{19}F NMR (376 MHz, $CDCl_3$) δ 55.64, 55.57 ppm; HRMS (EI+) calcd for $C_{17}H_{17}FN_2O_2S$ $[M]^+$ 332.0995, found 332.0991.

7-Methyl-3-((pyrrolidin-1-ylsulfonyl)methyl)-2-(*o*-tolyl)-2*H*-indazole (6a)

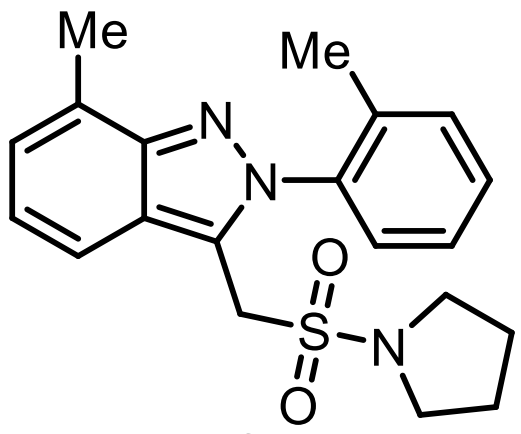

6a

30.0 mg (81%); White solid; ^{1}H NMR (600 MHz, $CDCl_3$) δ 7.63 – 7.60 (m, 1H), 7.47 – 7.43 (m, 2H), 7.39 – 7.35 (m, 2H), 7.12 – 7.10 (m, 2H), 4.56 (s, 2H), 3.12 – 3.10 (m, 4H), 2.65 (s, 3H), 2.02 (s, 3H), 1.81 – 1.79 (m, 4H) ppm; ^{13}C NMR (150 MHz, $CDCl_3$) δ 148.94, 137.95, 136.22, 131.28, 130.11, 128.33, 128.13, 126.55, 125.84, 124.77, 123.14, 121.58, 117.37, 48.07, 47.19, 25.89, 17.32, 17.12 ppm; FT-IR ($CHCl_3$, film) υ 3059, 2957, 2922, 2860, 1619, 1529, 1501, 1459, 1371, 1333, 1246, 1149, 1127, 1071, 1014, 945, 868, 766 cm^{-1}; HRMS (EI+) calcd for $C_{20}H_{23}N_3O_2S$ $[M]^+$ 369.1511, found 369.1507.

Methyl *N*^{2}-(tert-butoxycarbonyl)-*N*^{6}-(((7-methyl-2-(*o*-tolyl)-2*H*-indazol-3-yl)methyl)sulfonyl)-*L*-lysinate (6b)

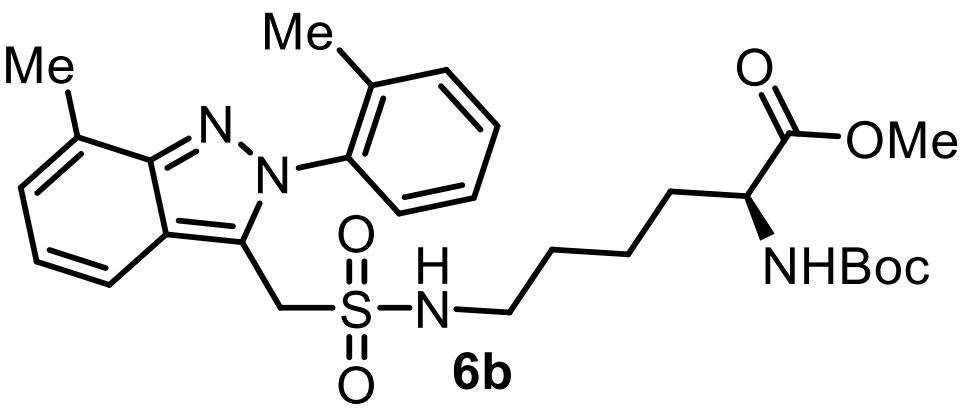

6b

10.6 mg (19%); White solid; ^{1}H NMR (600 MHz, $CDCl_3$) δ 7.62 (dt, J = 6.7, 3.0 Hz, 1H), 7.47 – 7.43 (m, 2H), 7.37 (dd, J = 15.6, 8.0 Hz, 2H), 7.13 - 7.10 (m, 2H), 5.04 (d, J = 8.3 Hz, 1H),

4.60 (s, 1H), 4.44 (t, J = 5.9 Hz, 2H), 4.24 (q, J = 7.7 Hz, 1H), 3.72 (s, 3H), 2.80 (q, J = 6.7 Hz, 2H), 2.64 (s, 3H), 2.00 (s, 3H), 1.74 – 1.68 (m, 2H), 1.54 (dq, J = 13.9, 7.6, 6.3 Hz, 2H), 1.42 (s, 9H), 1.33 – 1.27 (m, 2H) ppm; ^{13}C NMR (100 MHz, $CDCl_3$) δ 173.21, 155.61, 149.14, 138.04, 136.29, 131.45, 130.29, 128.56, 128.30, 126.83, 126.00, 124.79, 123.48, 121.74, 117.44, 80.23, 53.13, 52.48, 49.48, 43.43, 32.50, 29.81, 28.44, 22.24, 17.40, 17.19 ppm; FT-IR ($CHCl_3$, film) υ 2954, 2922, 2853, 1737, 1699, 1499, 1458, 1365, 1331, 1243, 1154, 1088, 1015, 865, 753, 722, 674 cm^{-1}; HRMS (EI+) calcd for $C_{28}H_{38}N_4O_6S$ [M]$^+$ 558.2512, found 558.2509.

Methyl (*S*)-2-((tert-butoxycarbonyl)amino)-3-(4-((((7-methyl-2-(*o*-tolyl)-2*H*-indazol-3-yl)methyl)sulfonyl)oxy)phenyl)propanoate (6c)

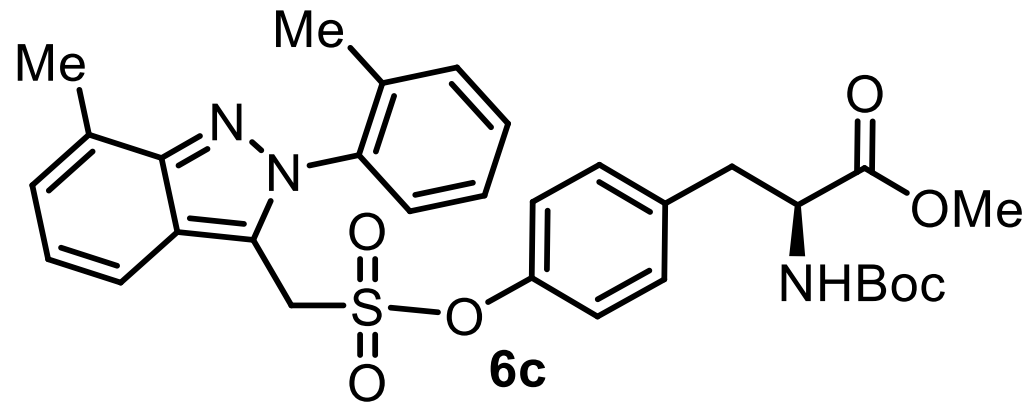

6c

85.4 mg (72%); White solid; ^{1}H NMR (600 MHz, $CDCl_3$) δ 7.66 – 7.65 (m, 1H), 7.46 (t, J = 7.6 Hz, 1H), 7.39 (q, J = 7.4 Hz, 2H), 7.34 (t, J = 7.0 Hz, 1H), 7.14 (d, J = 7.5 Hz, 2H), 7.07 (d, J = 8.5 Hz, 2H), 6.92 (d, J = 8.6 Hz, 2H), 5.00 – 4.74 (m, 2H), 4.55 (d, J = 7.8 Hz, 1H), 3.70 (s, 3H), 3.10 (dd, J = 14.0, 5.9 Hz, 1H), 3.00 (dd, J = 14.0, 6.5 Hz, 1H), 2.67 (s, 3H), 2.00 (s, 3H), 1.66 (s, 1H), 1.41 (s, 9H) ppm; ^{13}C NMR (150 MHz, $CDCl_3$) δ 172.10, 155.12, 149.21, 147.70, 137.63, 136.55, 135.65, 131.55, 130.92, 130.45, 128.60, 128.15, 126.88, 126.16, 123.90, 122.30, 121.97, 121.92, 117.43, 80.27, 54.37, 52.46, 47.88, 37.82, 28.40, 17.30, 17.19 ppm; FT-IR ($CHCl_3$, film) υ 3381, 2972, 2925, 2857, 1741, 1704, 1502, 1436, 1363, 1247, 1197, 1142, 1017, 862, 753 cm^{-1}; HRMS (EI+) calcd for $C_{31}H_{35}N_3O_7S$ [M]$^+$ 593.2196, found 593.2191.

***N*-(2-(2,6-Dioxopiperidin-3-yl)-1-oxoisoindolin-4-yl)-1-(7-methyl-2-(o-tolyl)-2H-indazol-3-yl)methanesulfonamide (6d)**

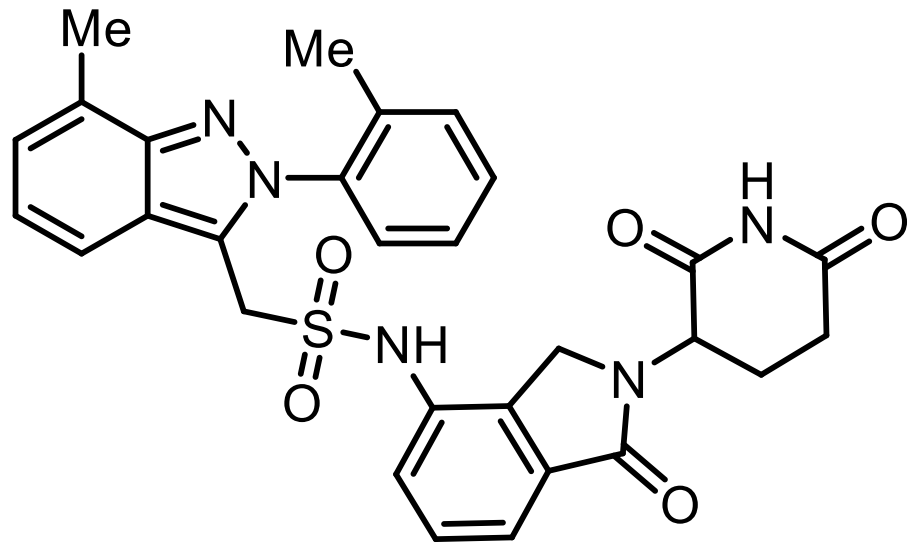

6d

30.0 mg (54%); White solid; ^{1}H NMR (400 MHz, DMSO-d_6) δ 11.01 (s, 1H), 7.72 (d, *J* = 8.4 Hz, 1H), 7.50 – 7.42 (m, 3H), 7.37 (td, *J* = 7.5, 1.9 Hz, 1H), 7.15 (t, *J* = 7.7 Hz, 1H), 7.06 (dt, *J* = 6.7, 1.1 Hz, 1H), 6.97 (dd, *J* = 8.5, 6.6 Hz, 1H), 6.92 (d, *J* = 6.8 Hz, 1H), 6.53 (d, *J* = 7.3 Hz, 1H), 6.12 (t, *J* = 5.3 Hz, 1H), 5.09 (dd, *J* = 13.2, 5.1 Hz, 1H), 4.60 (d, *J* = 5.6 Hz, 2H), 4.09 (d, *J* = 17.3 Hz, 1H), 4.01 (d, *J* = 17.2 Hz, 1H), 2.92 (ddd, *J* = 17.3, 13.5, 5.4 Hz, 1H), 2.62 (d, *J* = 17.3 Hz, 1H), 2.49 (s, 3H), 2.25 (qd, *J* = 13.2, 4.5 Hz, 1H), 2.07 – 2.00 (m, 1H), 1.93 (s, 3H) ppm; ^{13}C NMR (100 MHz, DMSO-D_6) δ 172.91, 171.21, 168.67, 148.23, 142.66, 138.69, 135.10, 133.71, 132.13, 130.90, 129.65, 129.07, 127.28, 126.83, 126.80, 126.53, 125.06, 121.39, 120.22, 118.12, 111.87, 110.80, 51.50, 45.62, 37.89, 31.24, 22.82, 16.82, 16.71 ppm; FT-IR ($CHCl_3$, film) υ 3384, 3194, 3084, 2919, 2852, 1729, 1707, 1671, 1612, 1526, 1493, 1458, 1421, 1375, 1348, 1328, 1233, 1190, 1148, 1006, 980, 916, 815, 790, 769, 747 cm^{-1}; HRMS (FAB-) calcd for $C_{29}H_{26}N_5O_5S$ [M-H]$^-$ 556.1654, found 556.1662.

(8*R*,9*S*,13*S*,14*S*)-13-methyl-17-oxo-7,8,9,11,12,13,14,15,16,17-decahydro-6*H*-cyclopenta[a]phenanthren-3-yl (7-methyl-2-(*o*-tolyl)-2*H*-indazol-3-yl)methanesulfonate (6e)

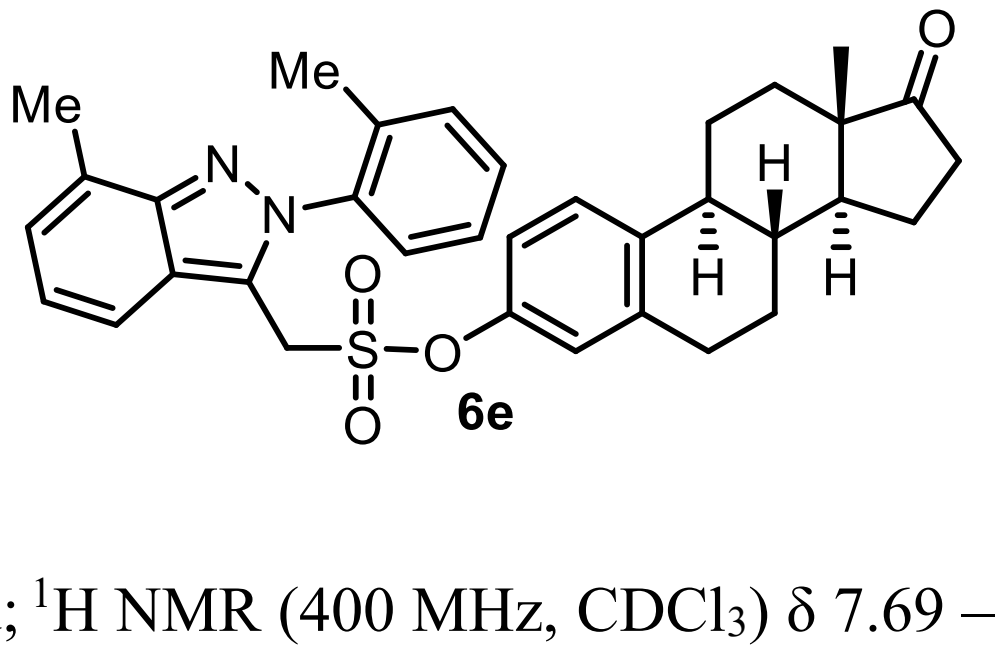

6e

63.7 mg (56%); White solid; ^{1}H NMR (400 MHz, $CDCl_3$) δ 7.69 – 7.65 (m, 1H), 7.45 (td, *J* = 7.4, 1.5 Hz, 1H), 7.43 – 7.36 (m, 2H), 7.36 – 7.30 (m, 1H), 7.20 (d, *J* = 8.7 Hz, 1H), 7.17 – 7.11 (m, 2H), 6.75 (dd, *J* = 8.6, 2.7 Hz, 1H), 6.68 (d, *J* = 2.6 Hz, 1H), 4.83 (d, *J* = 41.0 Hz, 2H),

2.81 (dd, J = 9.8, 5.2 Hz, 2H), 2.67 (s, 3H), 2.55 – 2.45 (m, 1H), 2.40 – 2.32 (m, 1H), 2.24 (td, J = 10.8, 7.1 Hz, 1H), 2.19 – 2.06 (m, 1H), 2.01 (s, 3H), 1.97 (s, 1H), 1.71 – 1.36 (m, 8H), 0.90 (s, 3H) ppm; ^{13}C NMR (150 MHz, $CDCl_3$) δ 149.16, 146.62, 139.15, 138.83, 137.64, 136.53, 131.47, 130.35, 128.48, 128.13, 126.93, 126.80, 126.10, 123.77, 122.48, 121.95, 121.78, 118.97, 117.52, 50.44, 47.94, 47.79, 44.16, 37.91, 35.89, 31.57, 29.36, 26.22, 25.77, 21.64, 17.28, 17.16, 13.88 ppm; FT-IR ($CHCl_3$, film) υ 2921, 2850, 1732, 1489, 1370, 1171, 1141, 916, 819, 774, 722 cm^{-1}; HRMS (EI+) calcd for $C_{34}H_{36}N_2O_4S$ [M]$^+$ 568.2396, found 568.2395.

4-Oxo-2-phenyl-4*H*-chromen-7-yl (7-methyl-2-(*o*-tolyl)-2*H*-indazol-3-yl)methanesulfonate (6f)

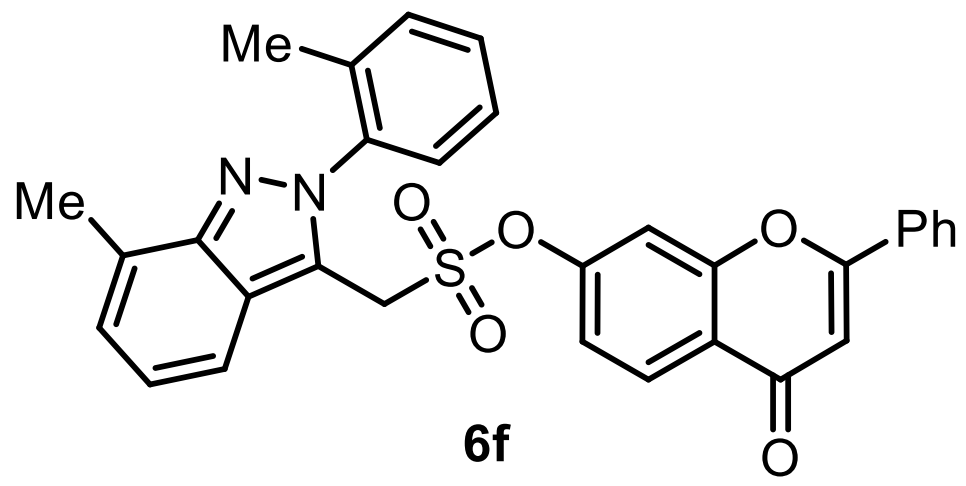

6f

41.5 mg (77%); Yellow solid; ^{1}H NMR (600 MHz, $CDCl_3$) δ 8.16 (d, J = 8.7 Hz, 1H), 7.87 (d, J = 6.8 Hz, 2H), 7.69 – 7.67 (m, 1H), 7.54 (dd, J = 11.6, 7.2 Hz, 4H), 7.45 – 7.37 (m, 3H), 7.33 (t, J = 7.6 Hz, 1H), 7.19 (dd, J = 18.6, 4.0 Hz, 3H), 6.99 (dd, J = 8.8, 2.3 Hz, 1H), 6.80 (s, 1H), 4.96 (d, J = 99.4 Hz, 2H), 2.68 (s, 3H), 2.02 (s, 3H) ppm; ^{13}C NMR (150 MHz, $CDCl_3$) δ 177.27, 163.97, 156.53, 152.09, 149.22, 137.51, 136.49, 132.07, 131.61, 131.26, 130.53, 129.25, 128.78, 128.03, 127.89, 126.90, 126.38, 126.25, 124.19, 122.84, 122.10, 121.66, 119.18, 117.14, 111.49, 107.80, 48.44, 17.31, 17.16 ppm; FT-IR ($CHCl_3$, film) υ 2922, 2850, 1734, 1489, 1377, 1177, 1147, 915, 819, 774, 722 cm^{-1}; HRMS (FAB+) calcd for $C_{31}H_{25}N_2O_5S$ [M+H]$^+$ 537.1484, found 537.1486.

8. ^{1}H NMR, ^{13}C NMR and ^{19}F NMR spectra of products

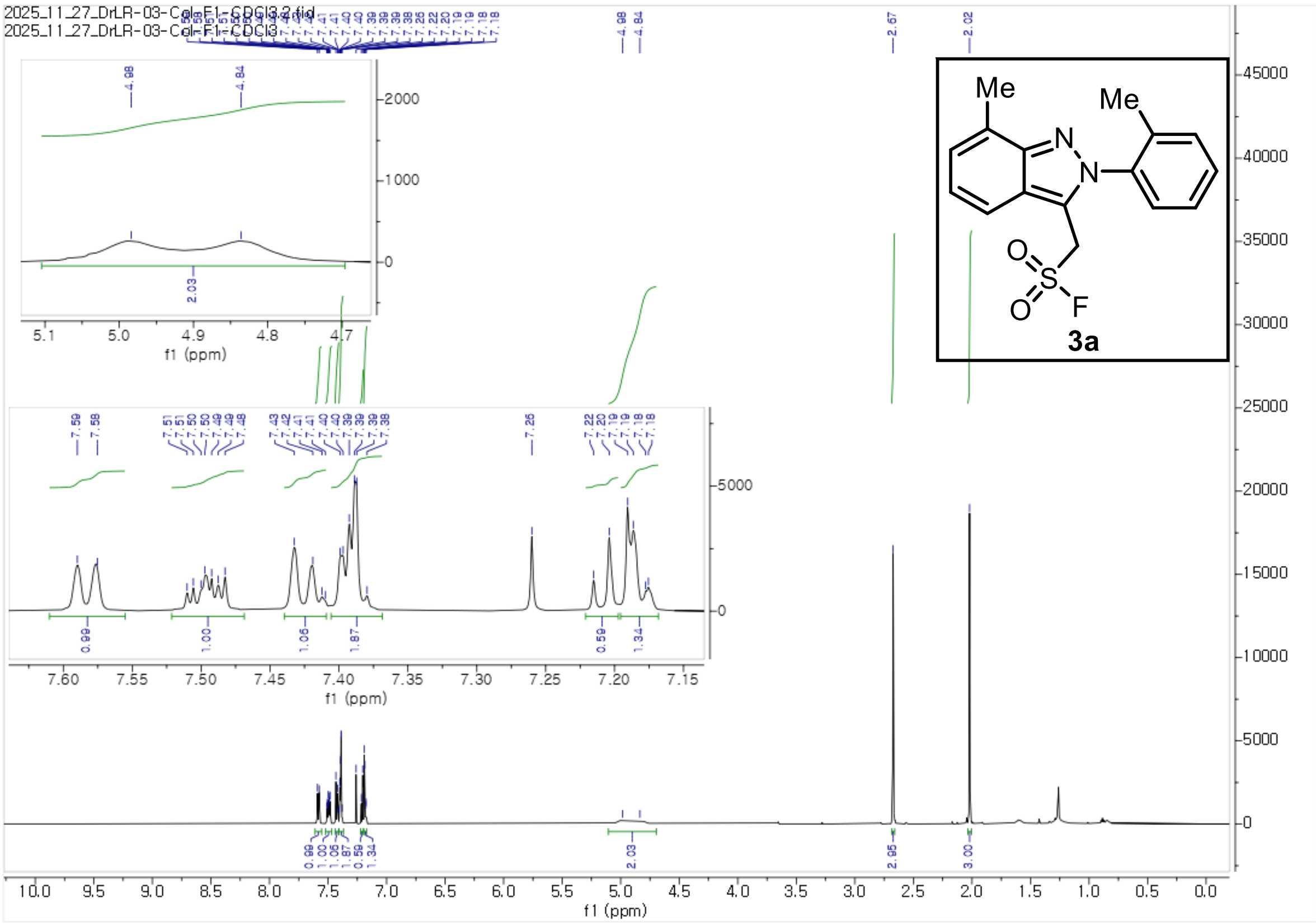

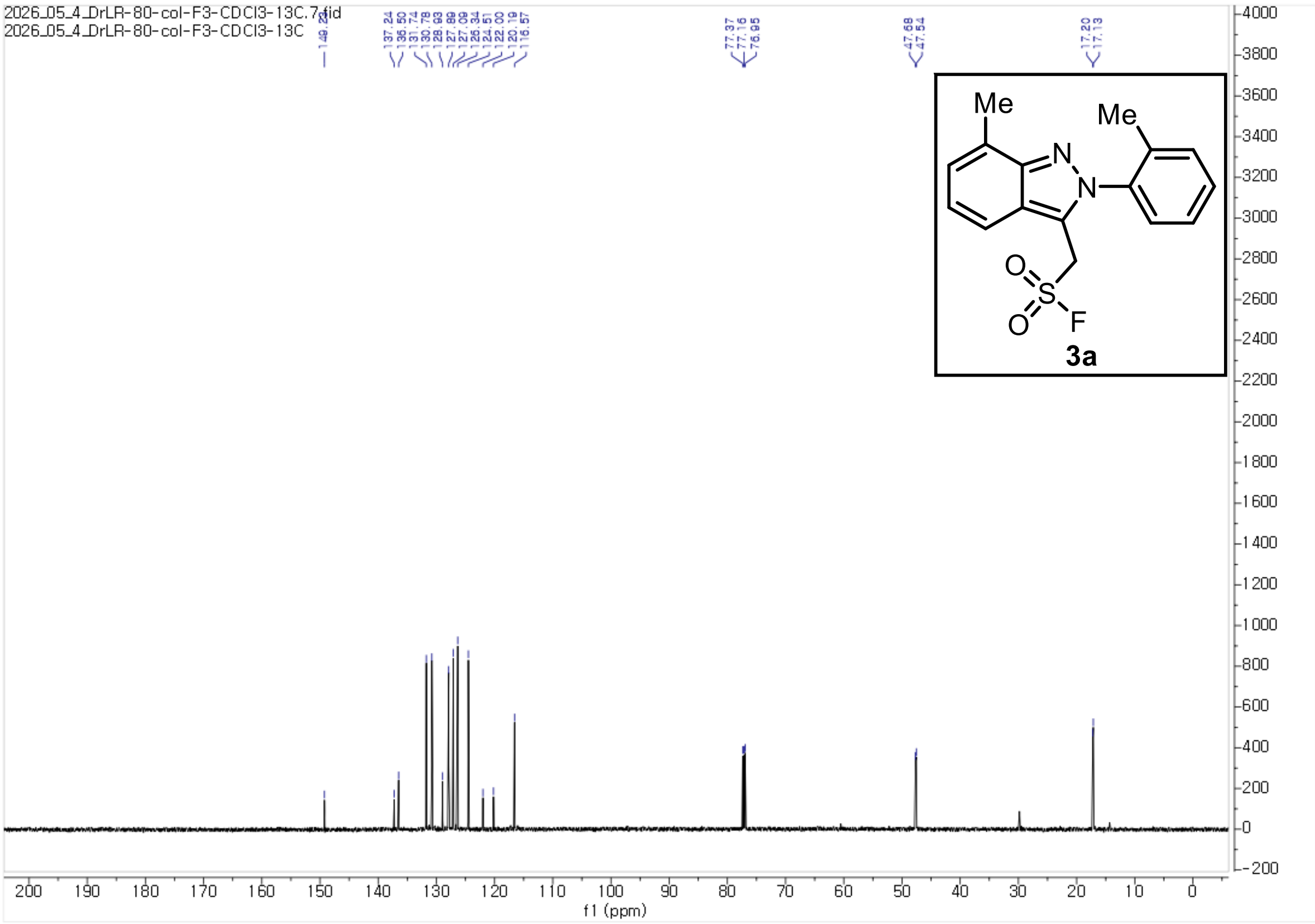
2026_05_4_DrLR-80-col-F3-CDCl3-13C.7.fid
2026_05_4_DrLR-80-col-F3-CDCl3-13C
Me
Me
N
N
O
S
O
F
3a
f1 (ppm)

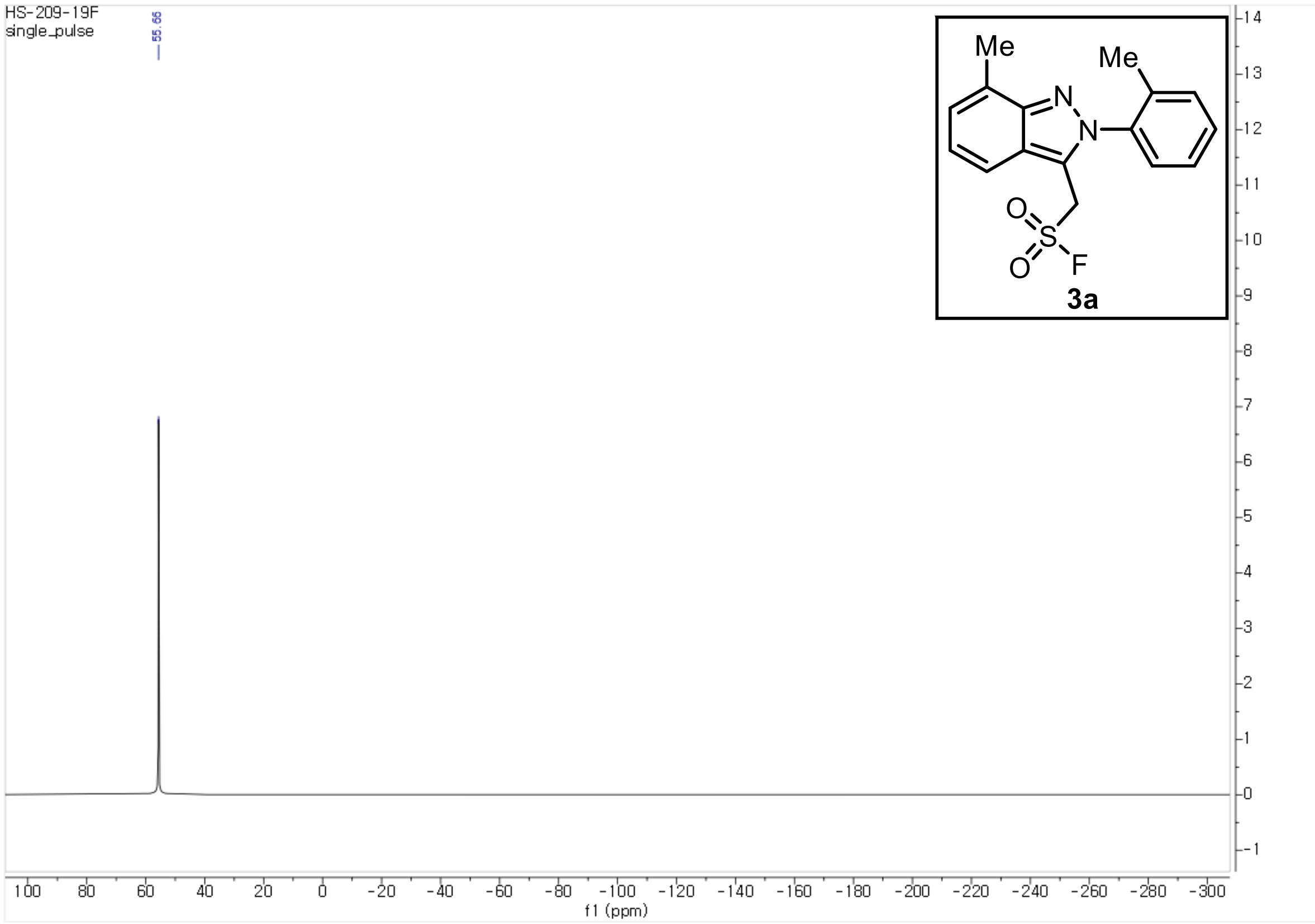
HS-209-19F
single_pulse
55.66
Me
Me
N
N
O
S
O
F
3a
f1 (ppm)

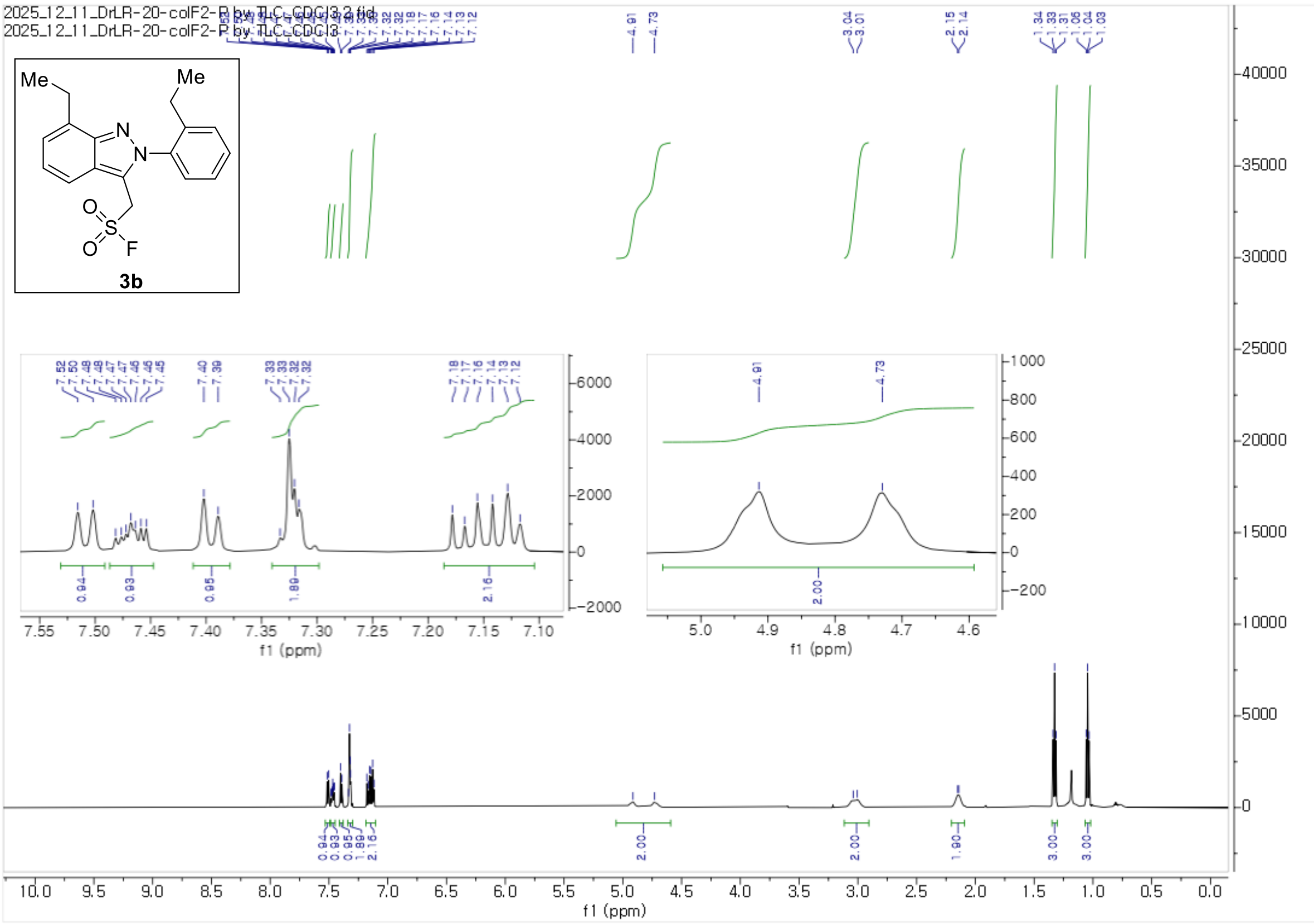

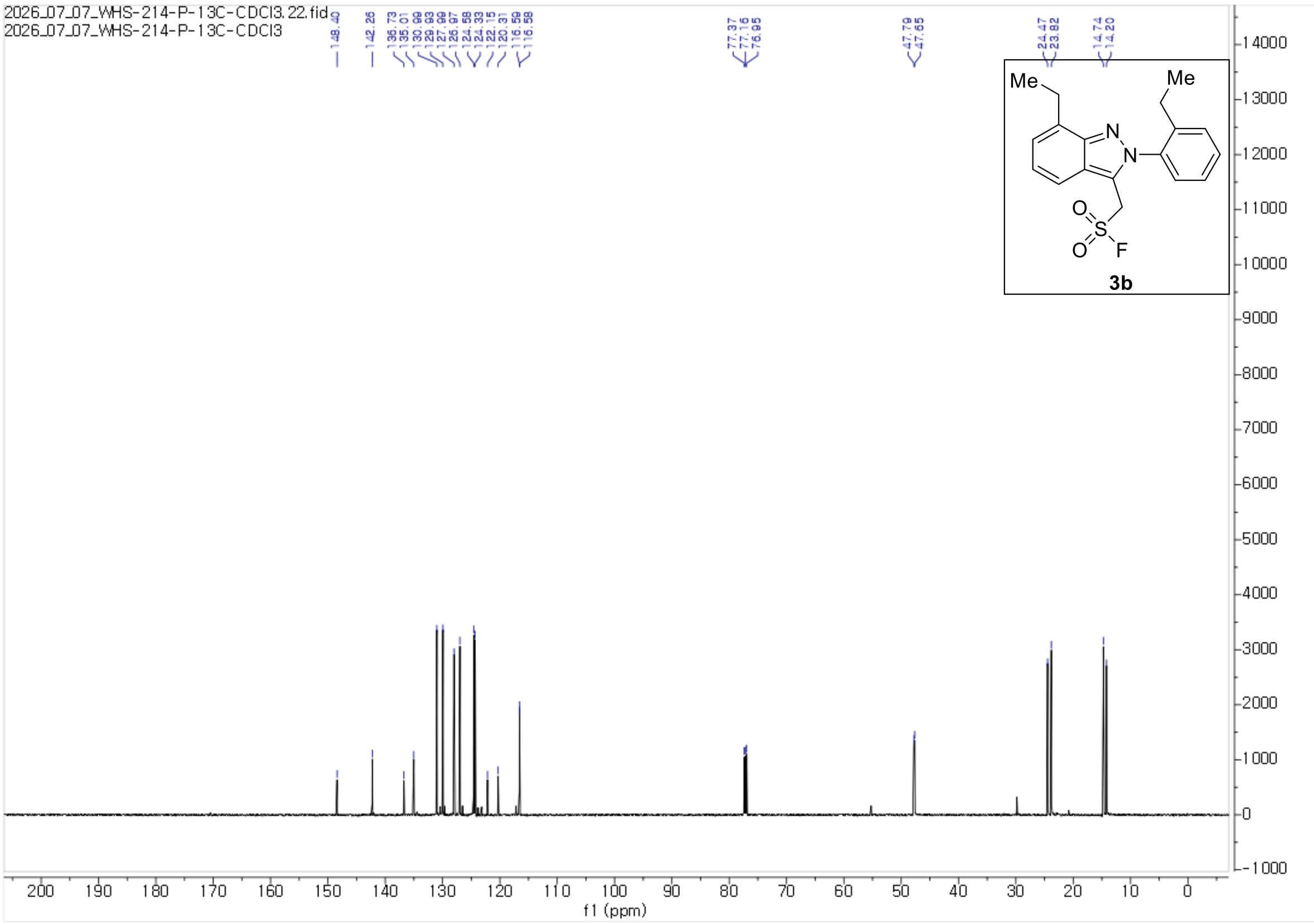

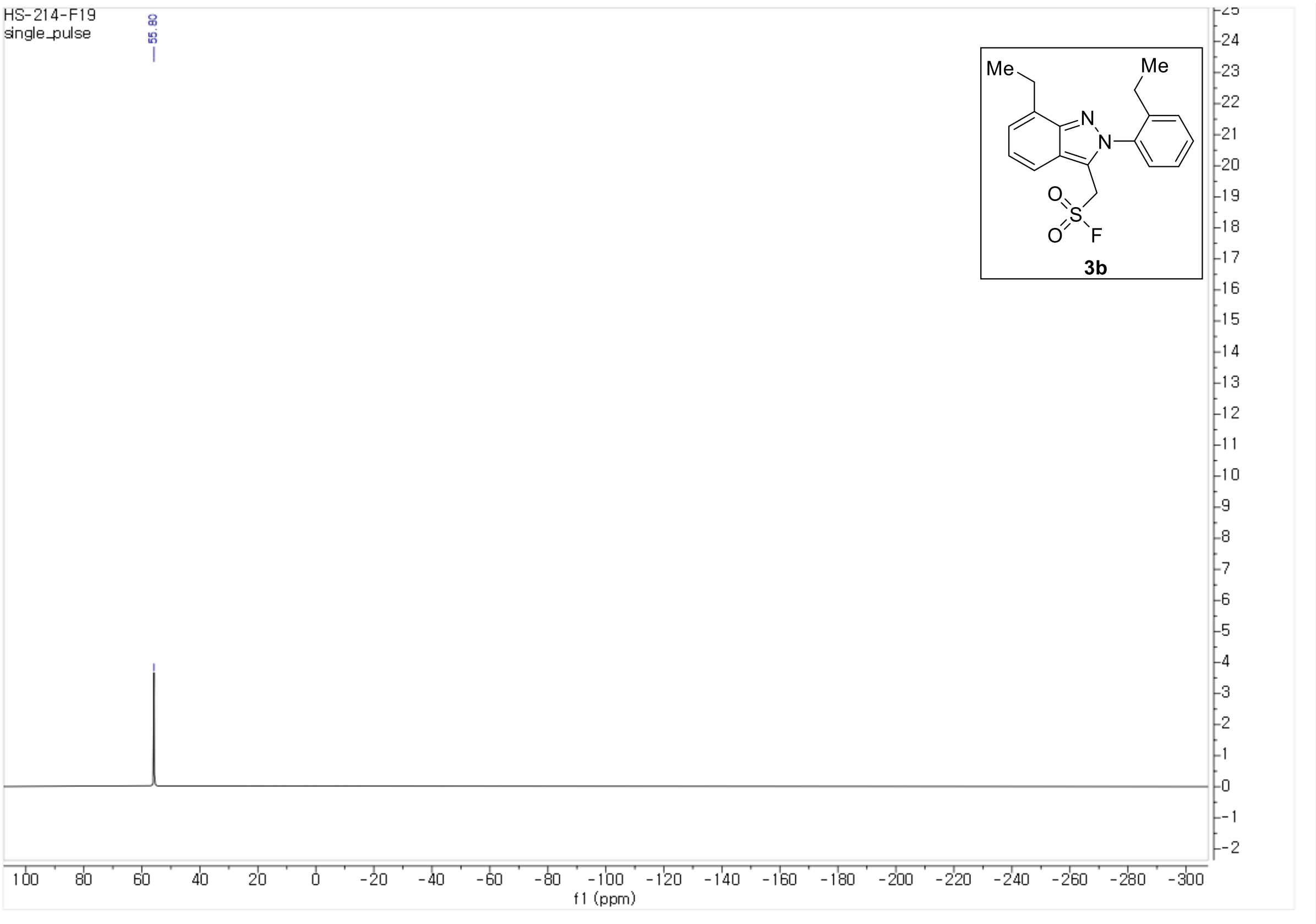
HS-214-F19
single_pulse
55.80
Me
Me
N
N
O
S
O
F
3b
f1 (ppm)

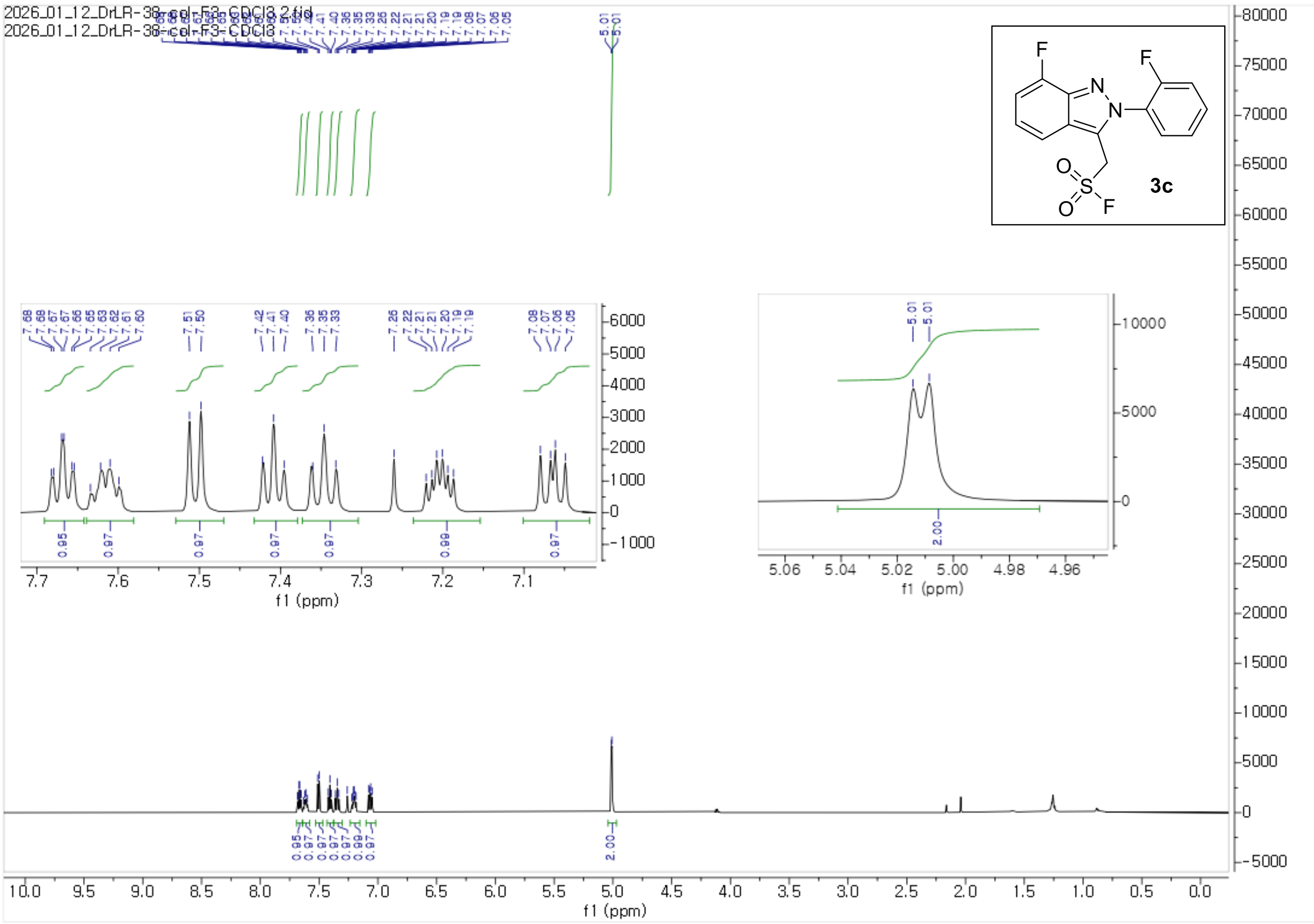
2026_01_12_DrLR-38-col-F3-CDCl3.2.fid
2026_01_12_DrLR-38-col-F3-CDCl3
f1 (ppm)
3c

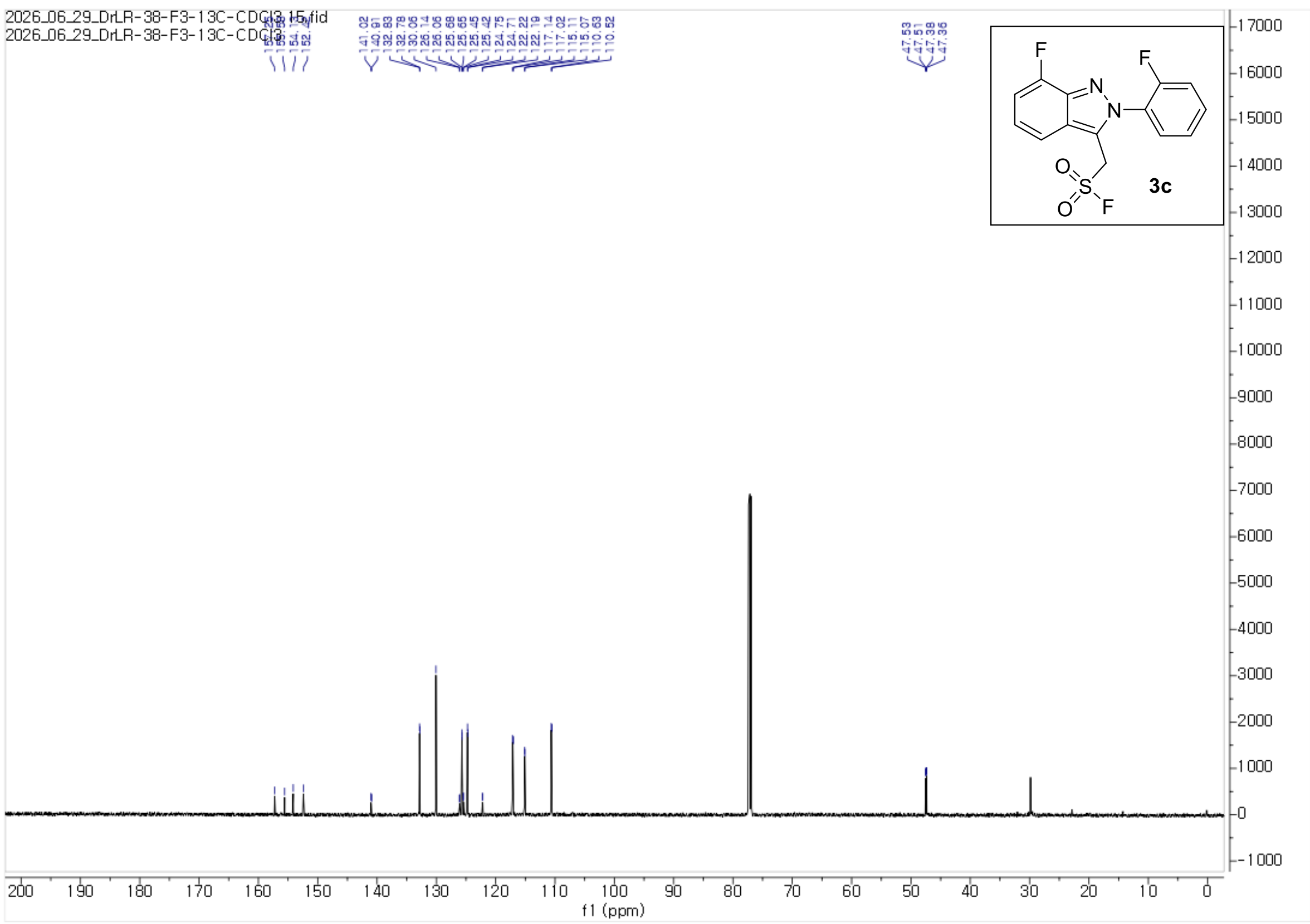
2026_06_29_DrLR-38-F3-13C-CDCl3.15.fid
2026_06_29_DrLR-38-F3-13C-CDCl3
f1 (ppm)
3c

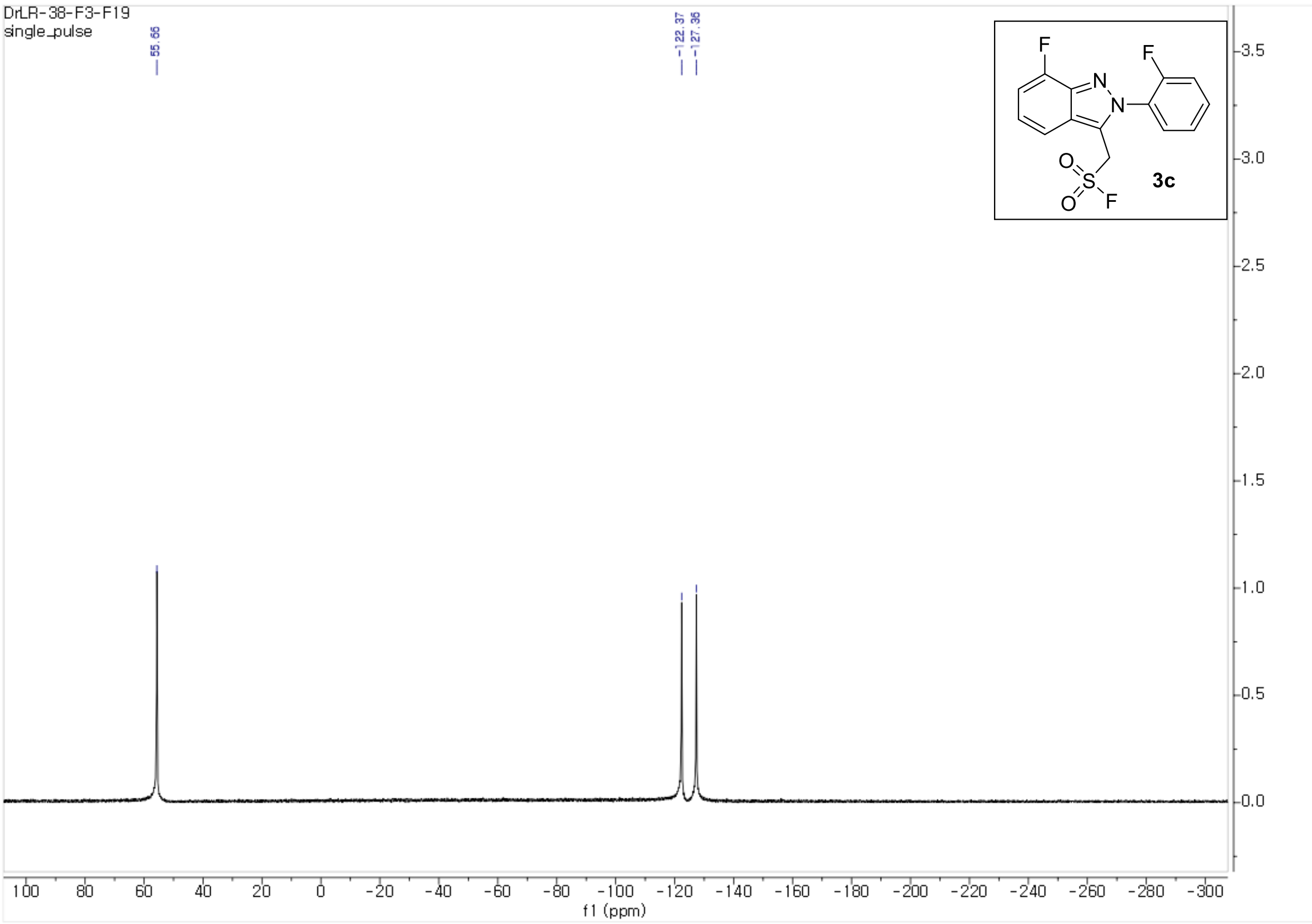
DrLR-38-F3-F19
single_pulse
55.66
-122.37
-127.36
3c
f1 (ppm)

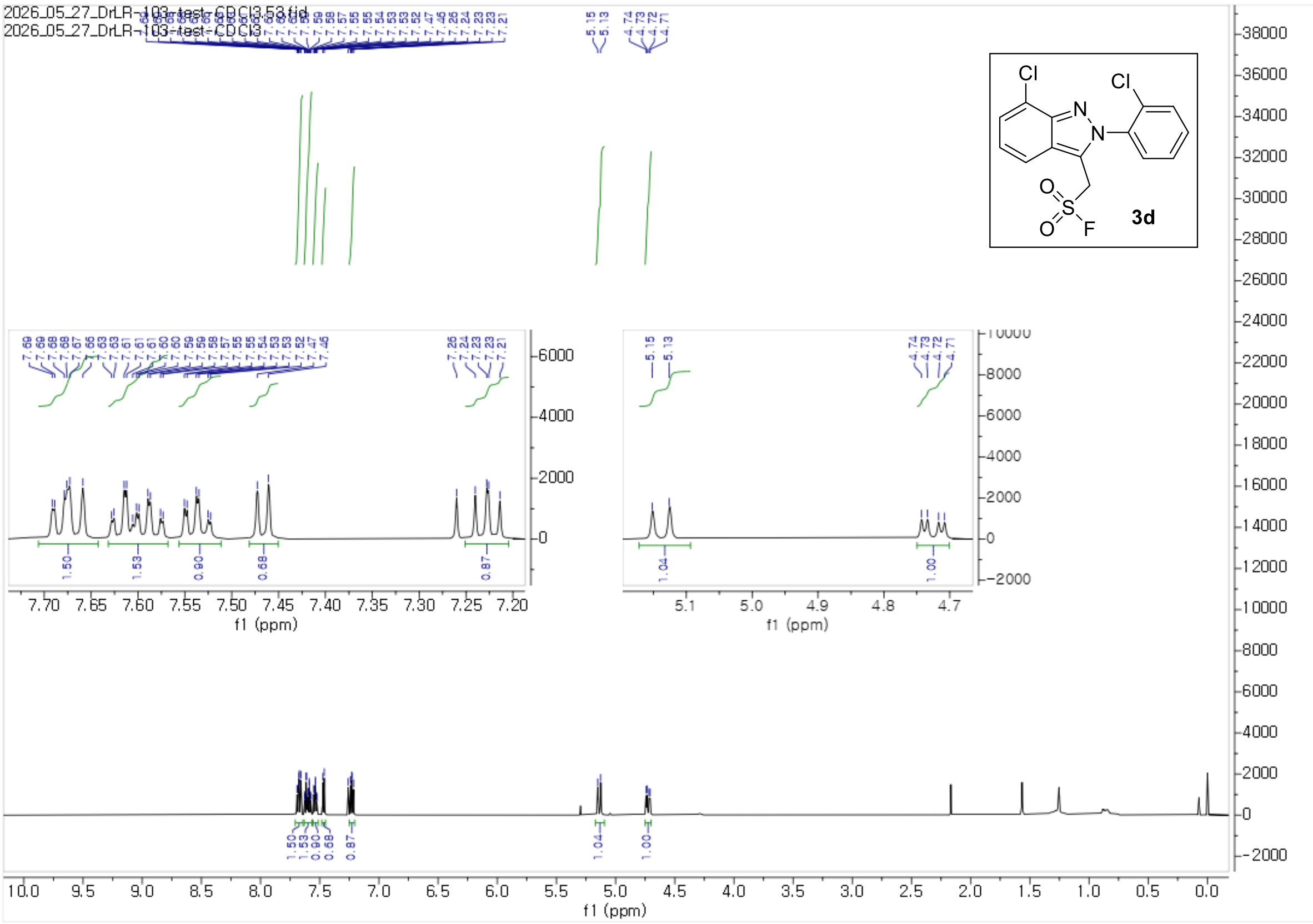
2026_05_27_DrLR-103-test-CDCl3.53.fid
2026_05_27_DrLR-103-test-CDCl3
3d
f1 (ppm)

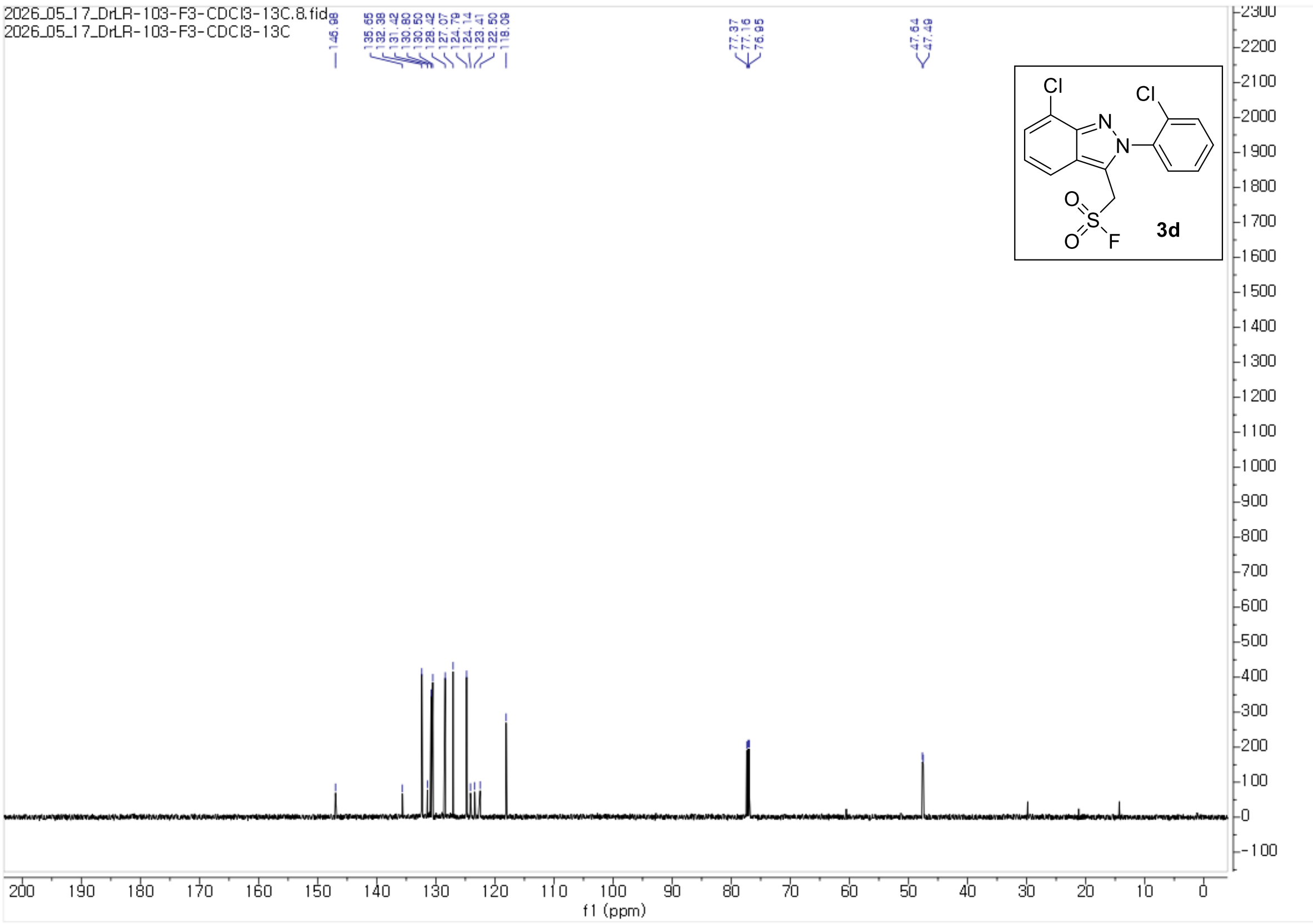
2026_05_17_DrLR-103-F3-CDCl3-13C.8.fid
2026_05_17_DrLR-103-F3-CDCl3-13C
146.98
135.65
132.38
131.42
130.80
130.50
128.42
127.07
125.79
124.14
123.41
122.50
118.09
77.37
77.16
76.95
47.64
47.49
3d
f1 (ppm)

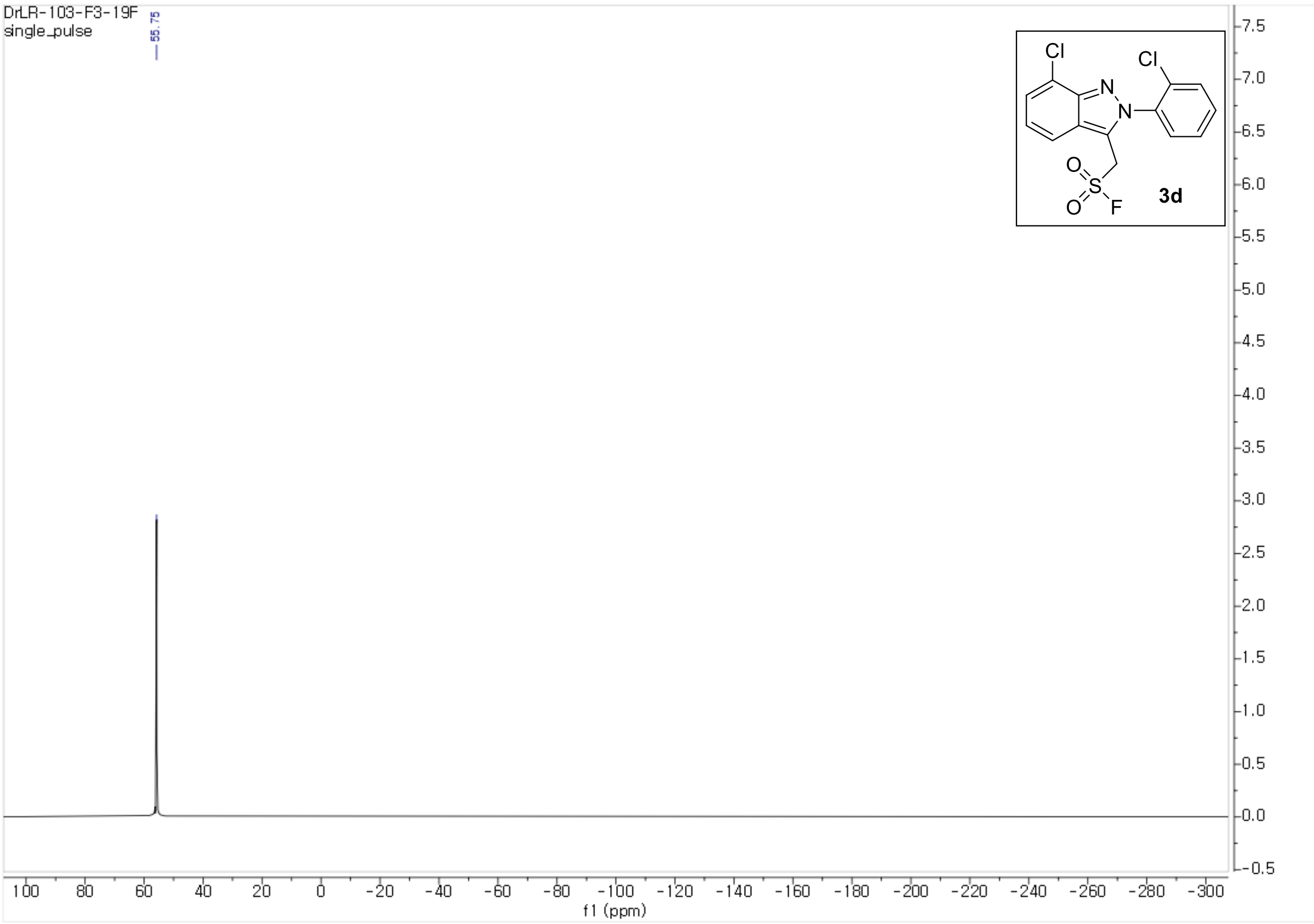

DrLR-103-F3-19F
single_pulse
55.75
Cl
Cl
N
N
O
S
O
F
3d
f1 (ppm)

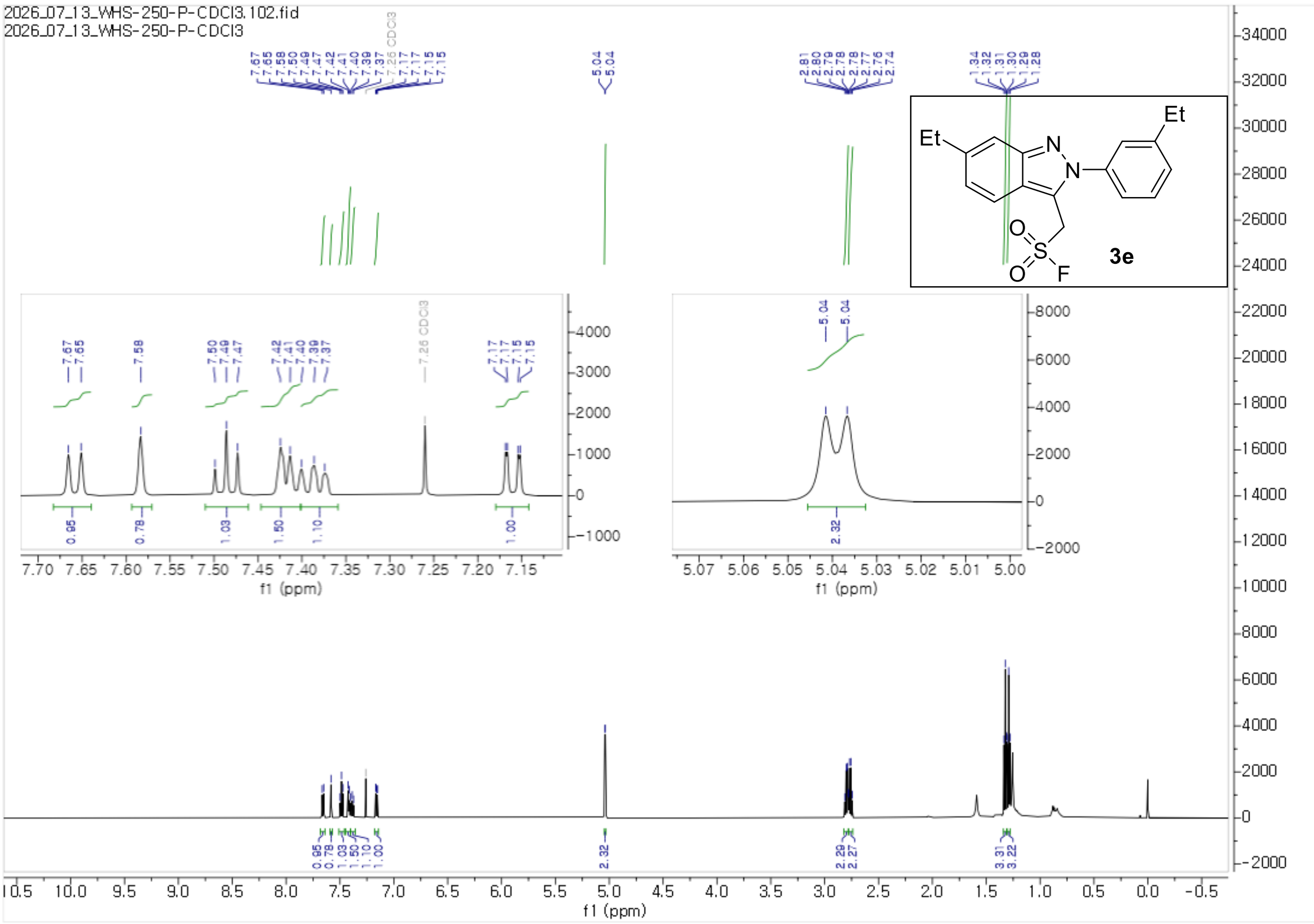
2026_07_13_WHS-250-P-CDCl3.102.fid
2026_07_13_WHS-250-P-CDCl3
Et
N
N
Et
O
S
O
F
3e
f1 (ppm)

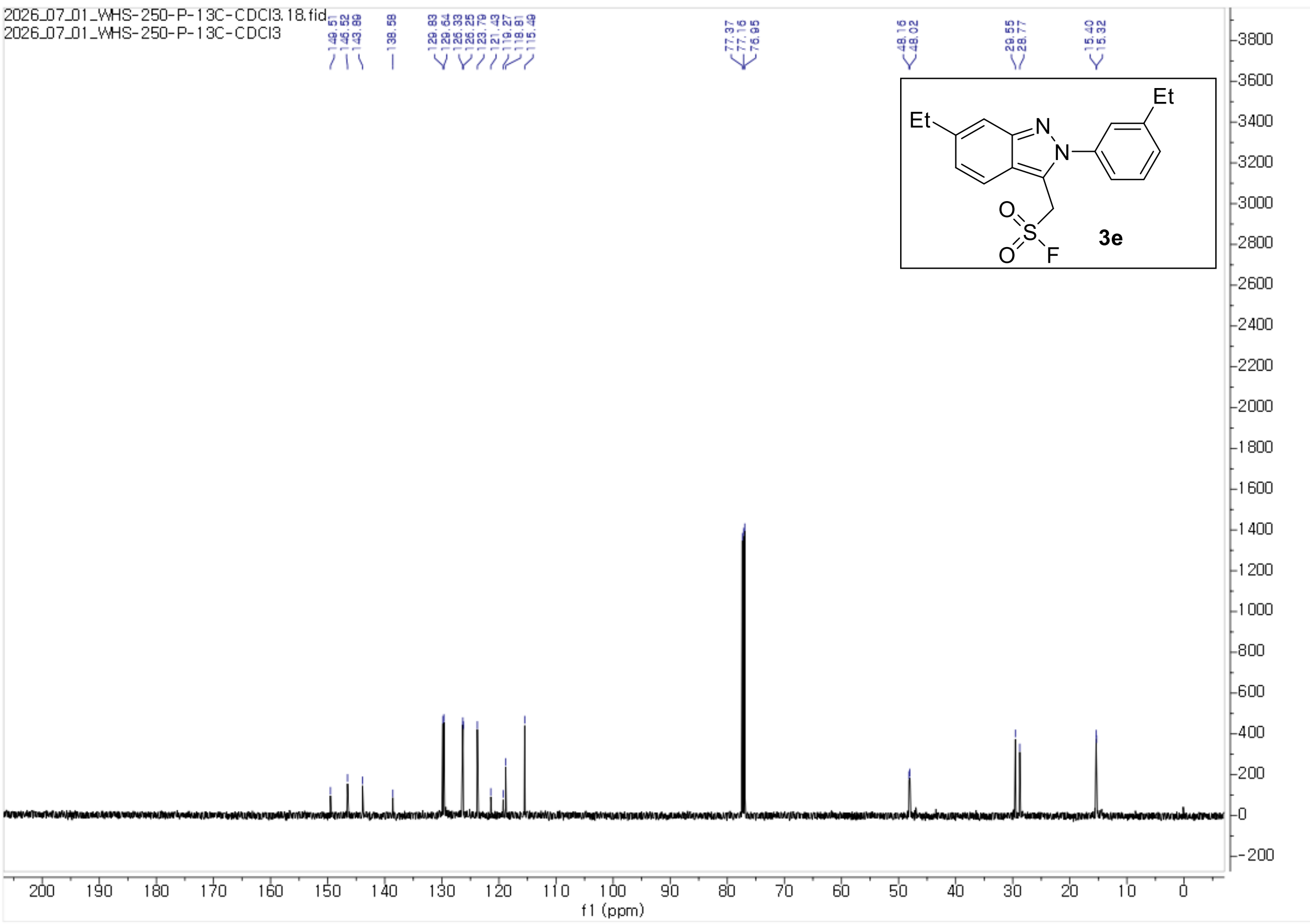
2026_07_01_WHS-250-P-13C-CDCl3.18.fid
2026_07_01_WHS-250-P-13C-CDCl3
Et
N
N
Et
O
S
O
F
3e
f1 (ppm)

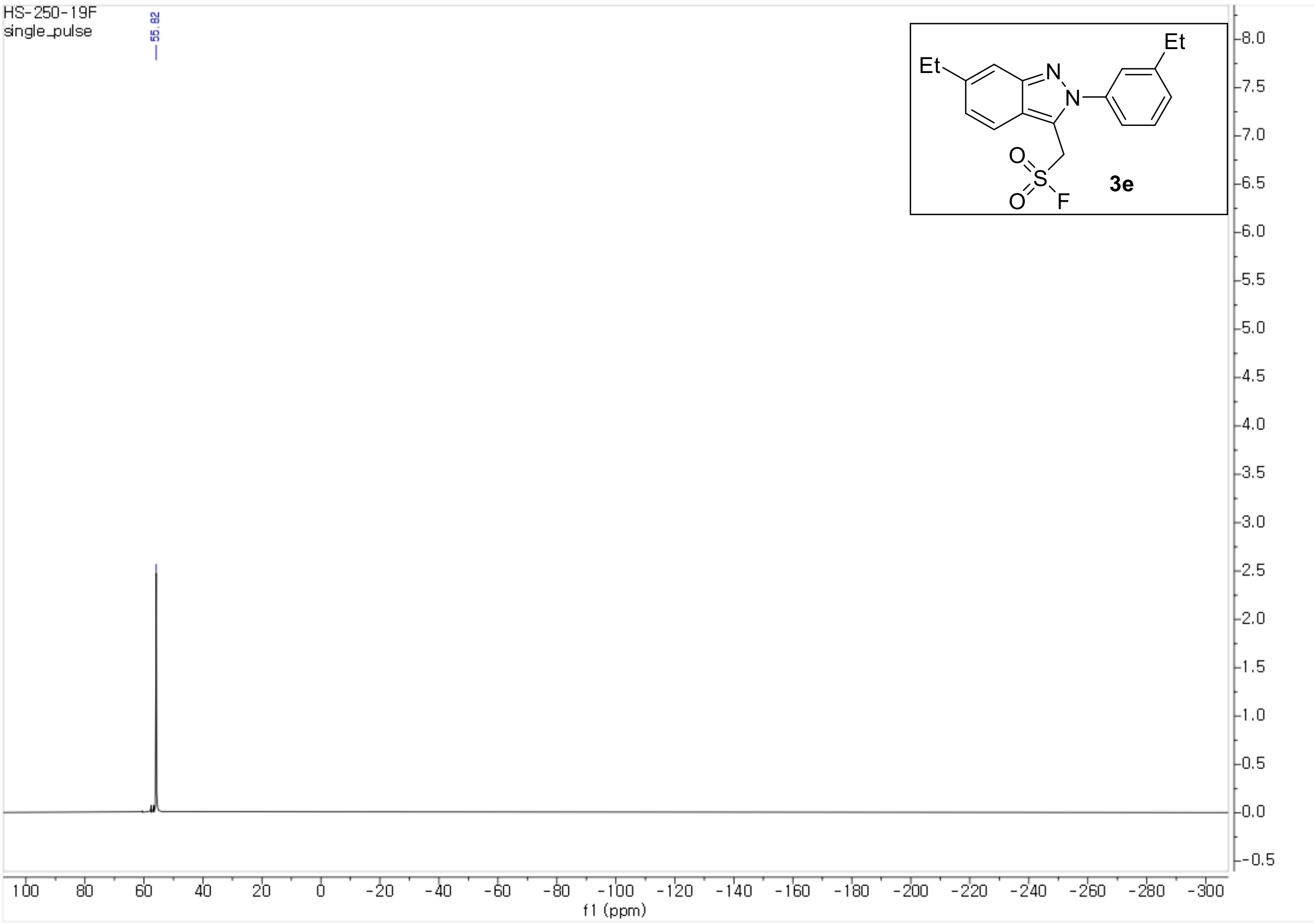
HS-250-19F
single_pulse
55.82
Et
N
N
Et
O
S
O
F
3e
f1 (ppm)

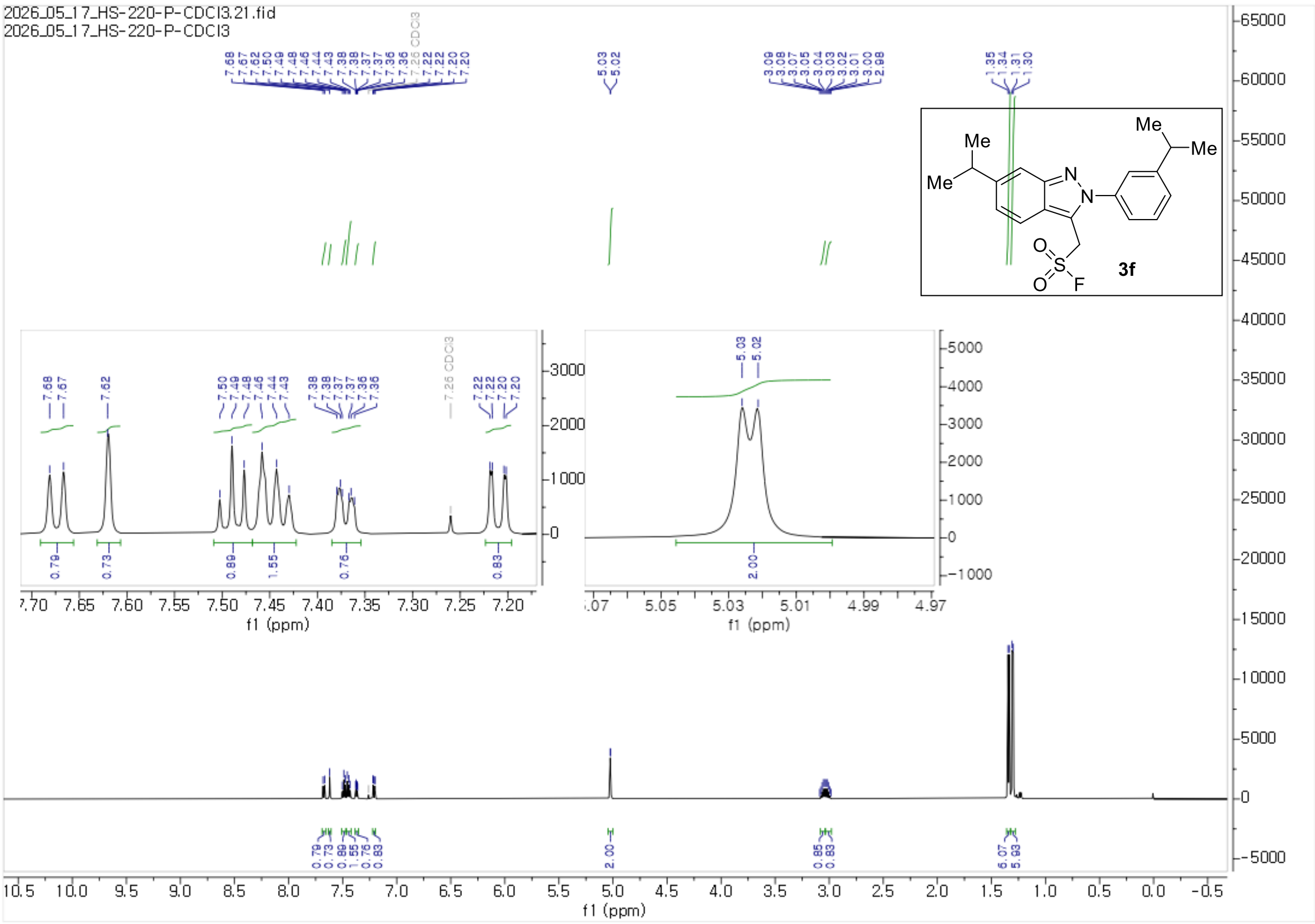
2026_05_17_HS-220-P-CDCl3.21.fid
2026_05_17_HS-220-P-CDCl3
3f
f1 (ppm)

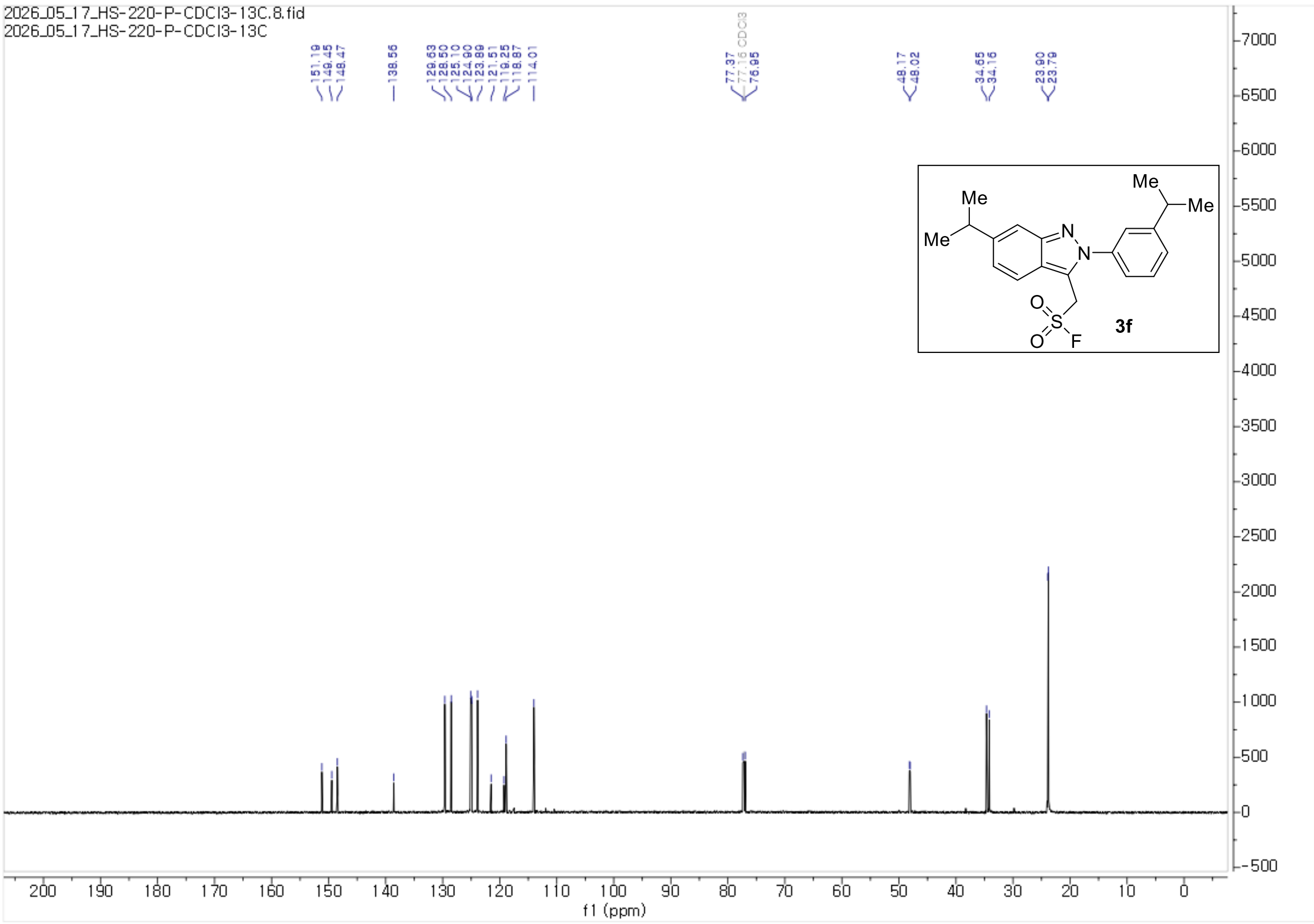
2026_05_17_HS-220-P-CDCl3-13C.8.fid
2026_05_17_HS-220-P-CDCl3-13C
3f
f1 (ppm)

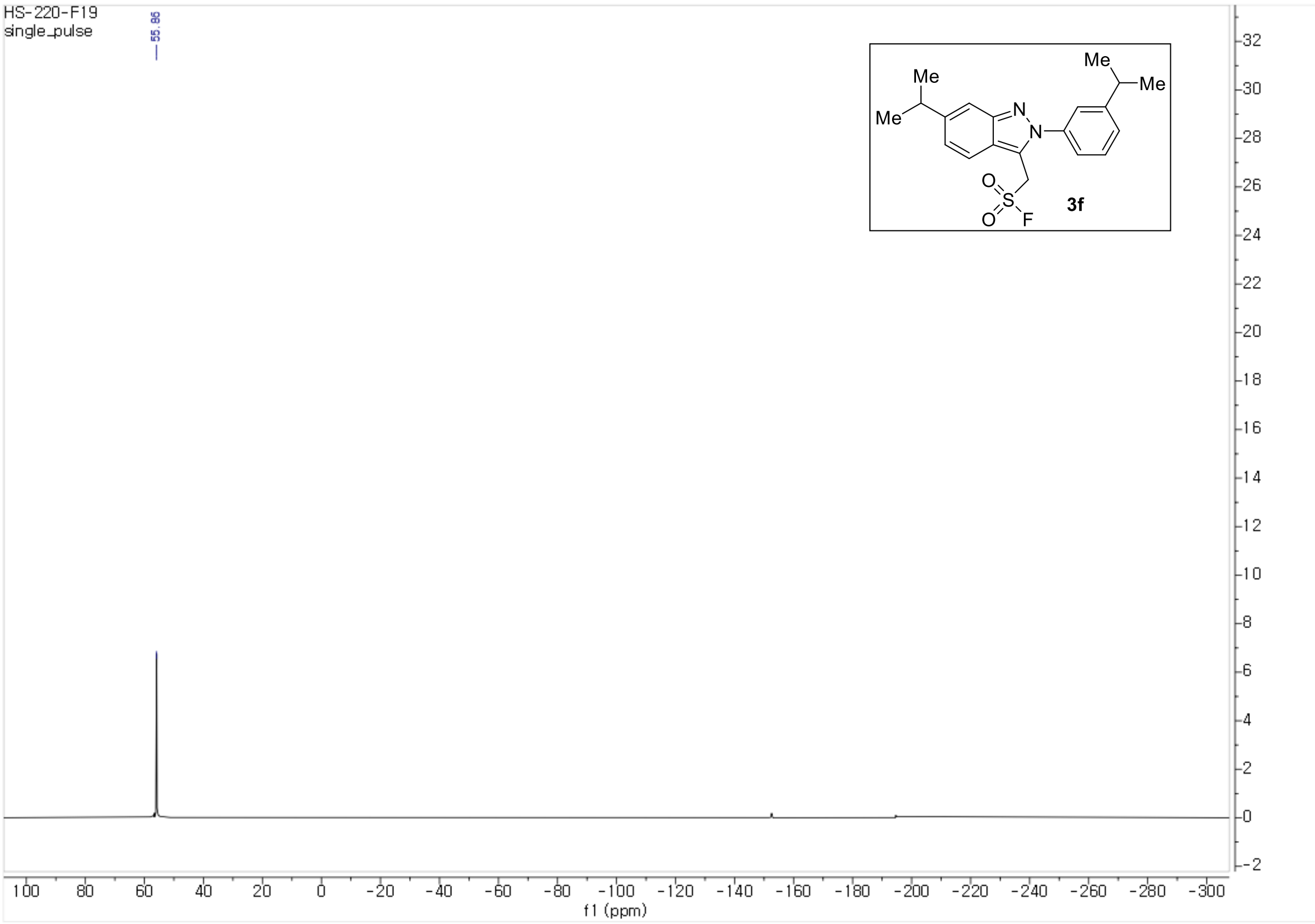
HS-220-F19
single_pulse
55.86
Me
Me
N
N
Me
Me
O
S
O
F
3f
f1 (ppm)

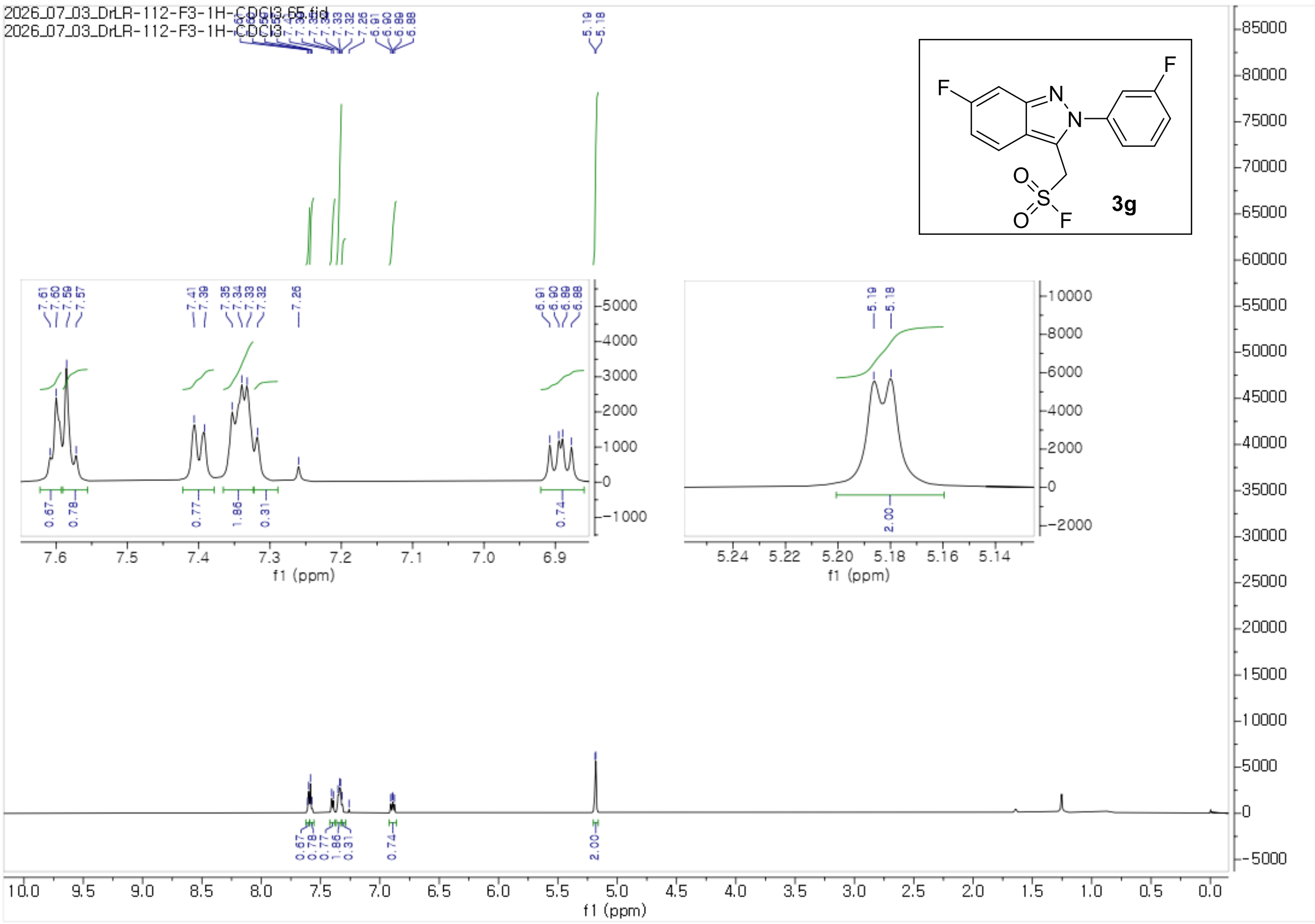
2026_07_03_DrLR-112-F3-1H-CDCl3.65.fid
2026_07_03_DrLR-112-F3-1H-CDCl3
3g
f1 (ppm)

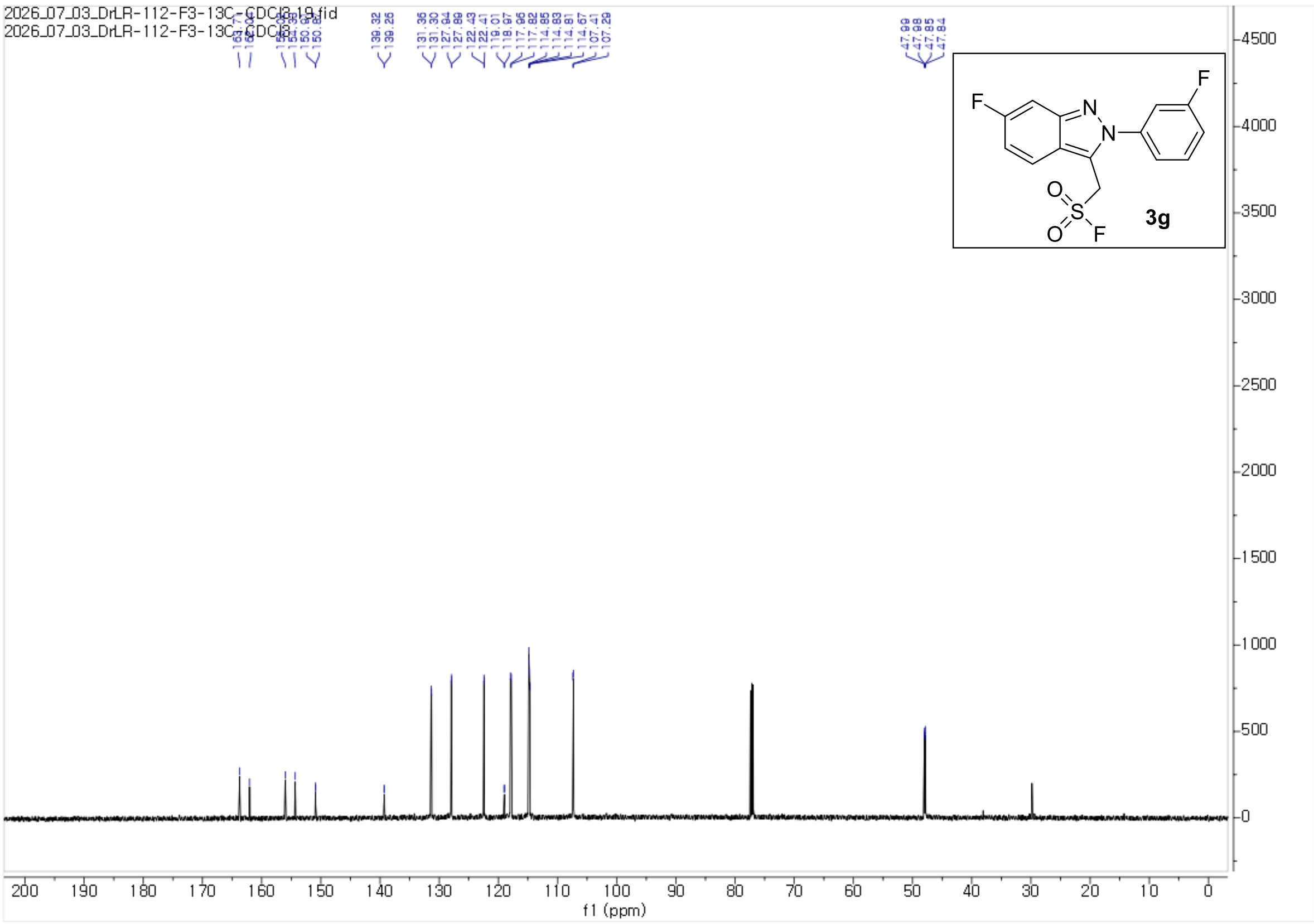
2026_07_03_DrLR-112-F3-13C-CDCl3.19.fid
2026_07_03_DrLR-112-F3-13C-CDCl3
3g
f1 (ppm)

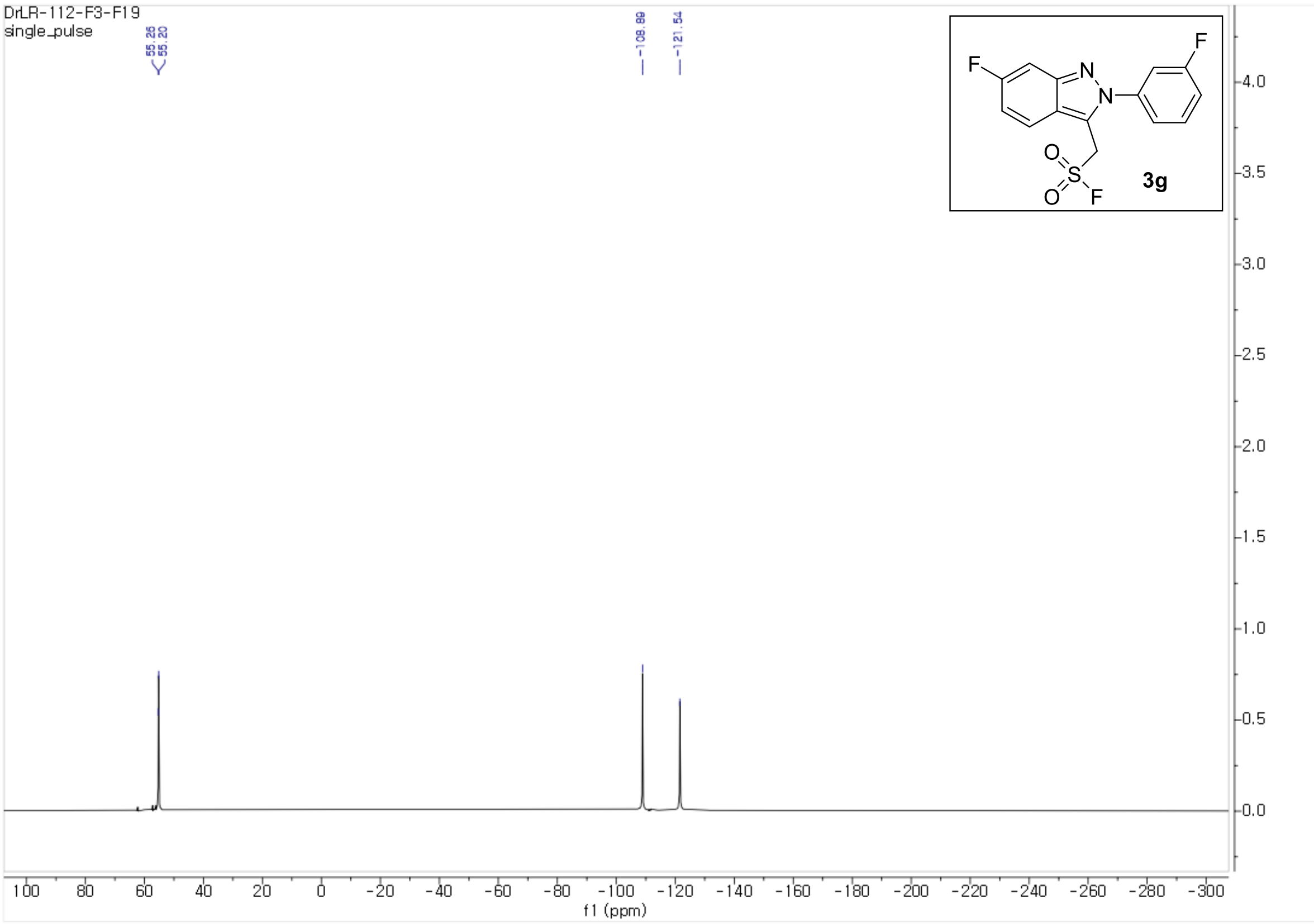
DrLR-112-F3-F19
single_pulse
55.26
55.20
−108.89
−121.54
f1 (ppm)
3g

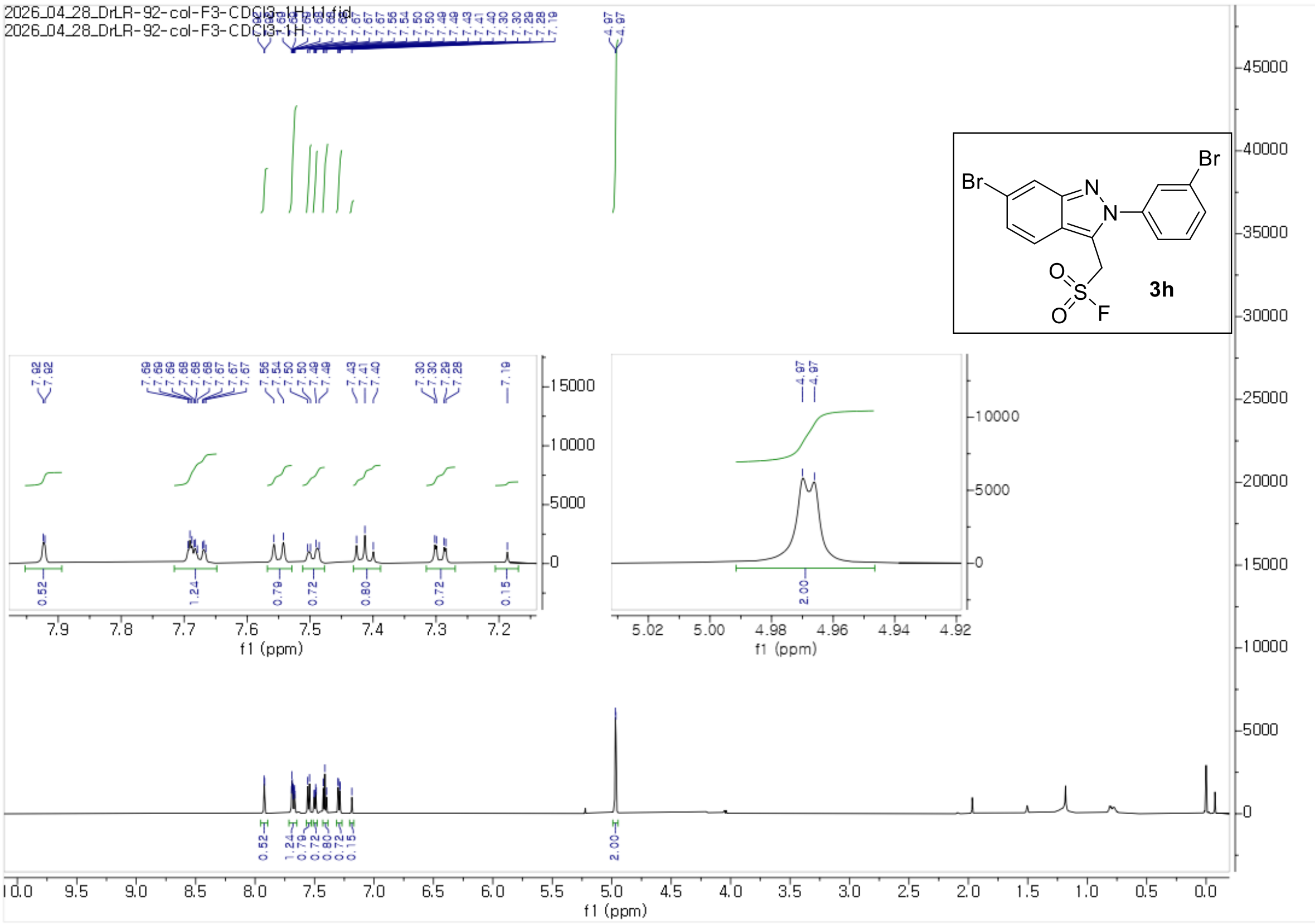
2026_04_28_DrLR-92-col-F3-CDCl3-1H.11.fid
2026_04_28_DrLR-92-col-F3-CDCl3-1H
3h
f1 (ppm)

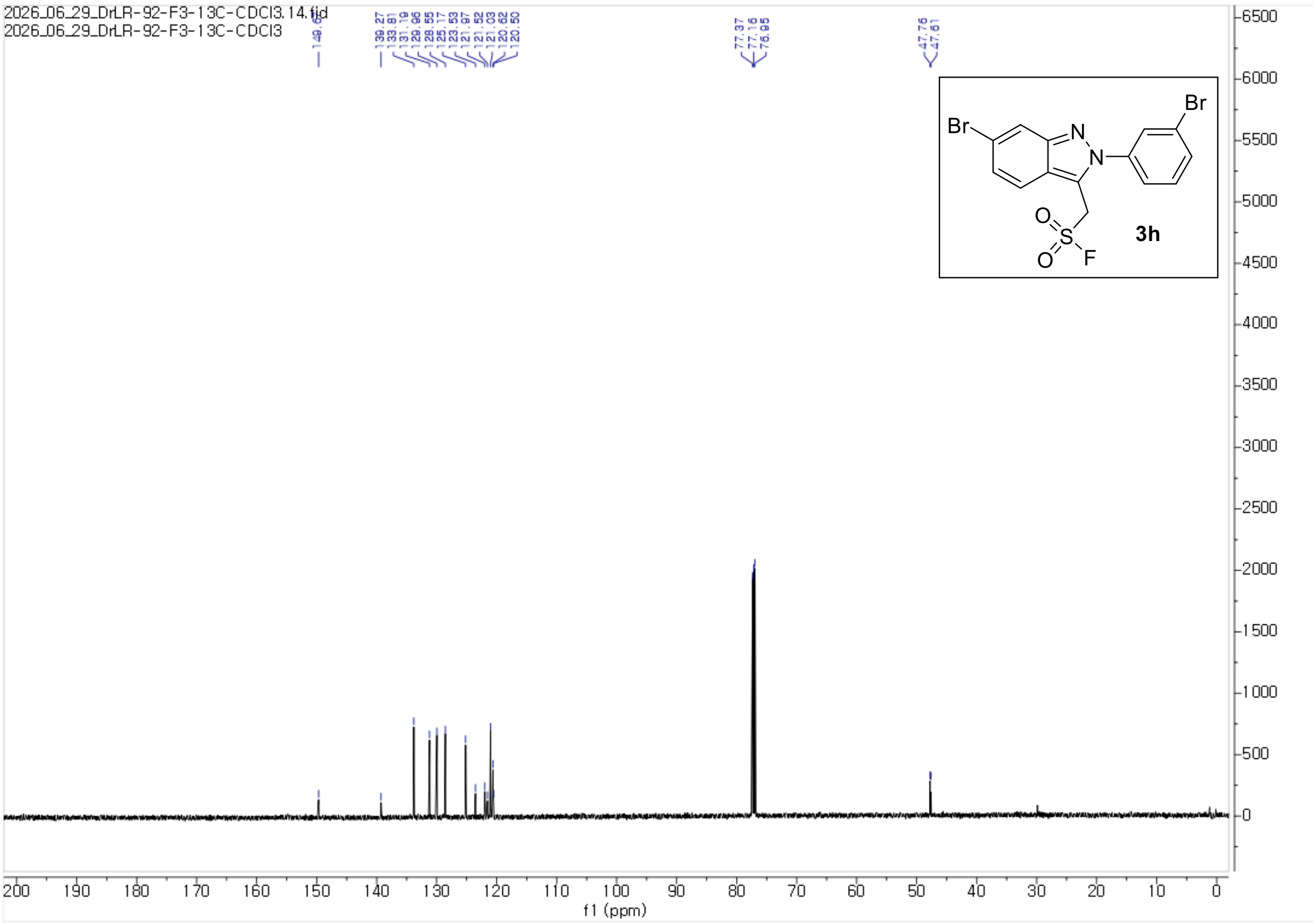
2026_06_29_DrLR-92-F3-13C-CDCl3.14.fid
2026_06_29_DrLR-92-F3-13C-CDCl3
3h
f1 (ppm)

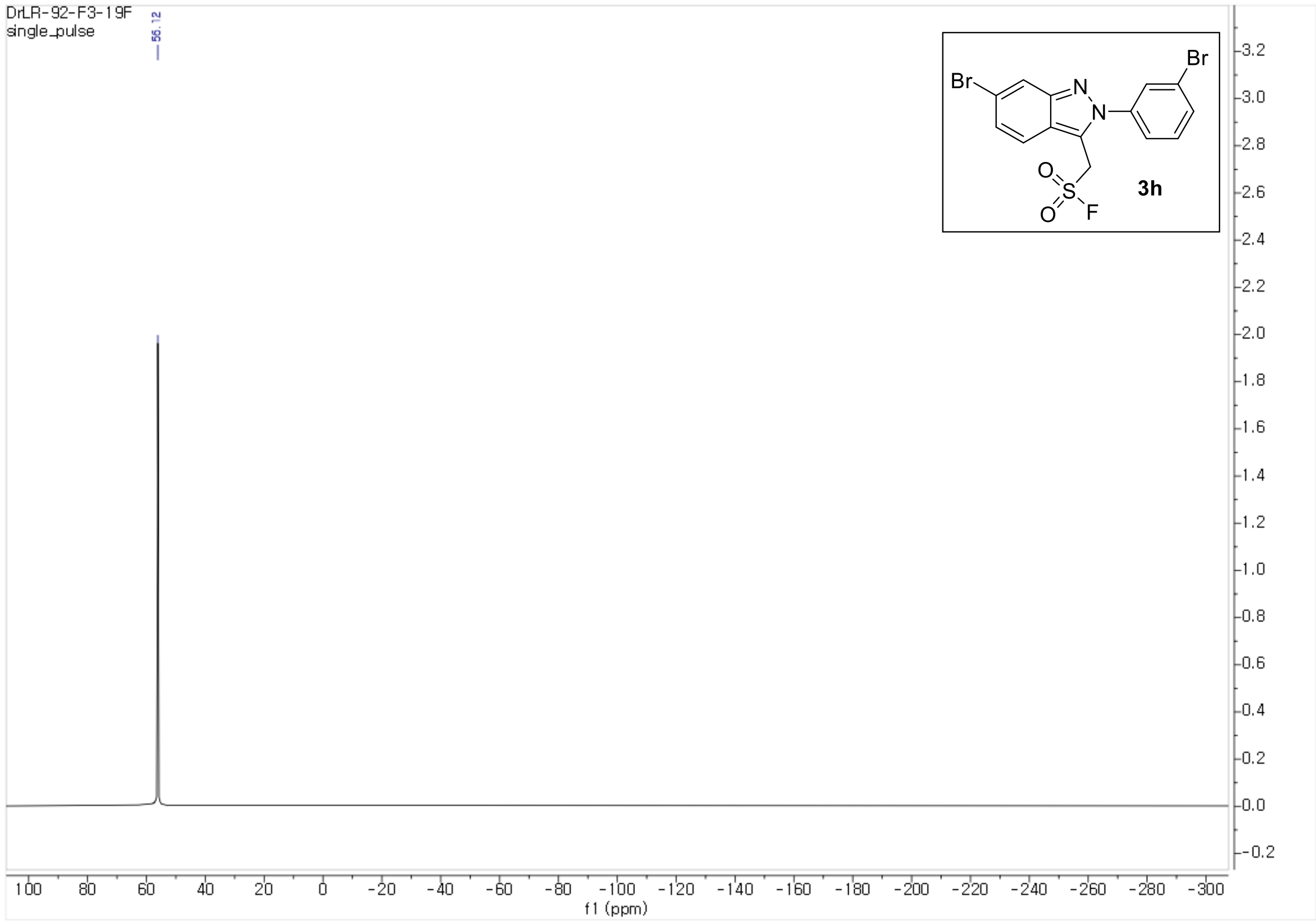
DrLR-92-F3-19F
single_pulse
55.12
Br
N
N
Br
O
S
O
F
3h
f1 (ppm)

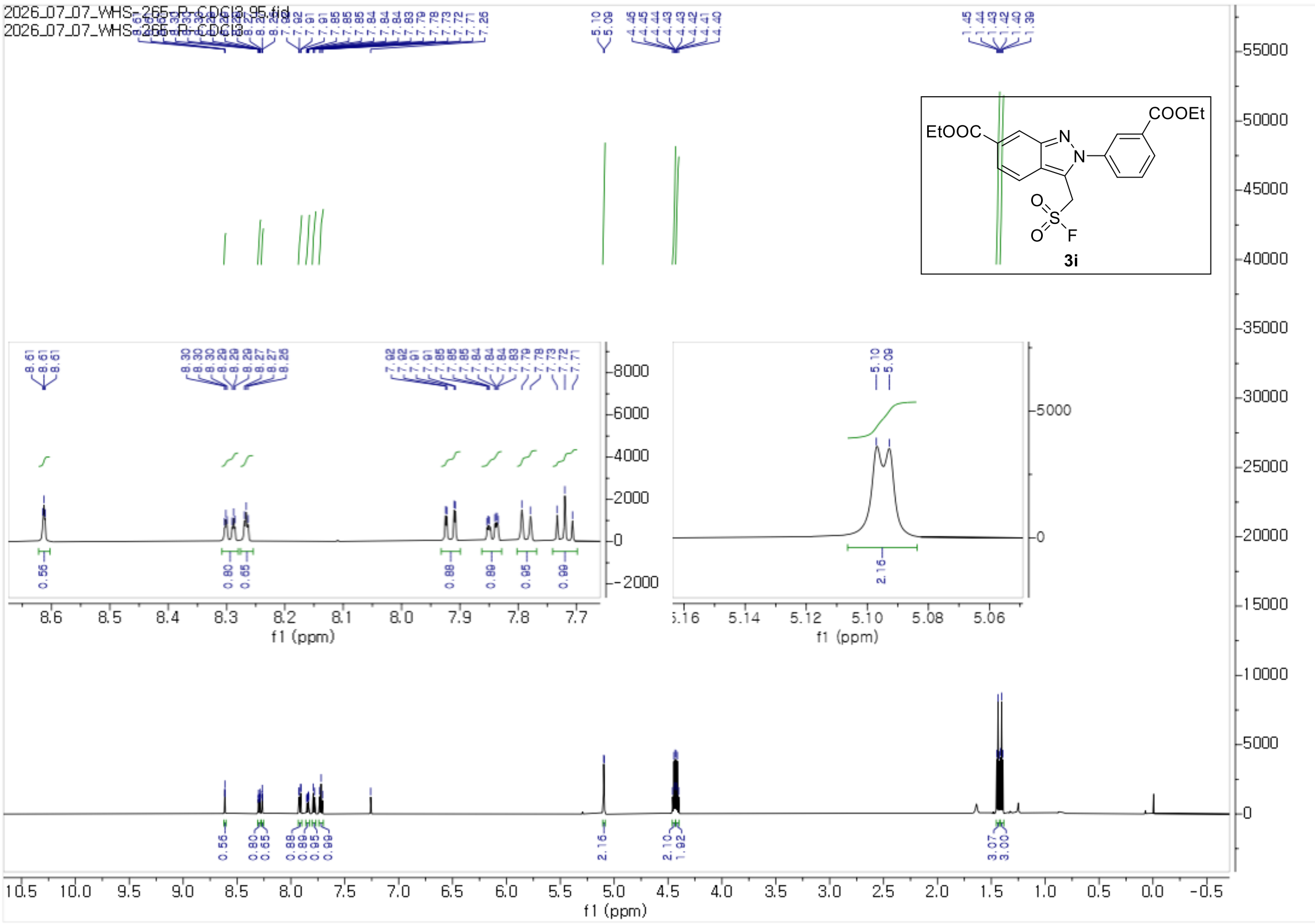
2026_07_07_WHS-265-P-CDCl3.95.fid
2026_07_07_WHS-265-P-CDCl3
EtOOC
COOEt
3i
f1 (ppm)

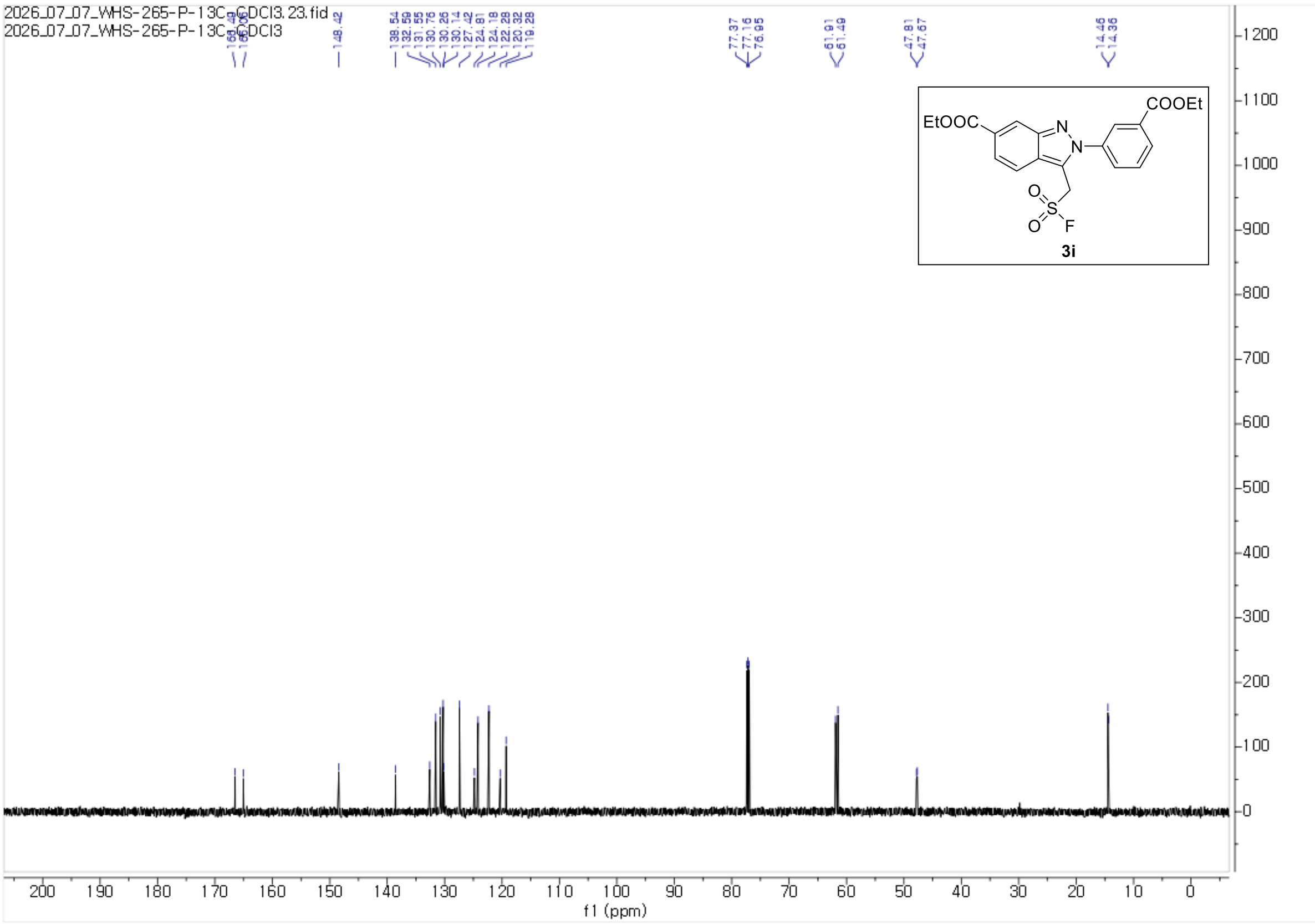
2026_07_07_WHS-265-P-13C-CDCl3.23.fid
2026_07_07_WHS-265-P-13C-CDCl3
EtOOC
COOEt
3i
f1 (ppm)

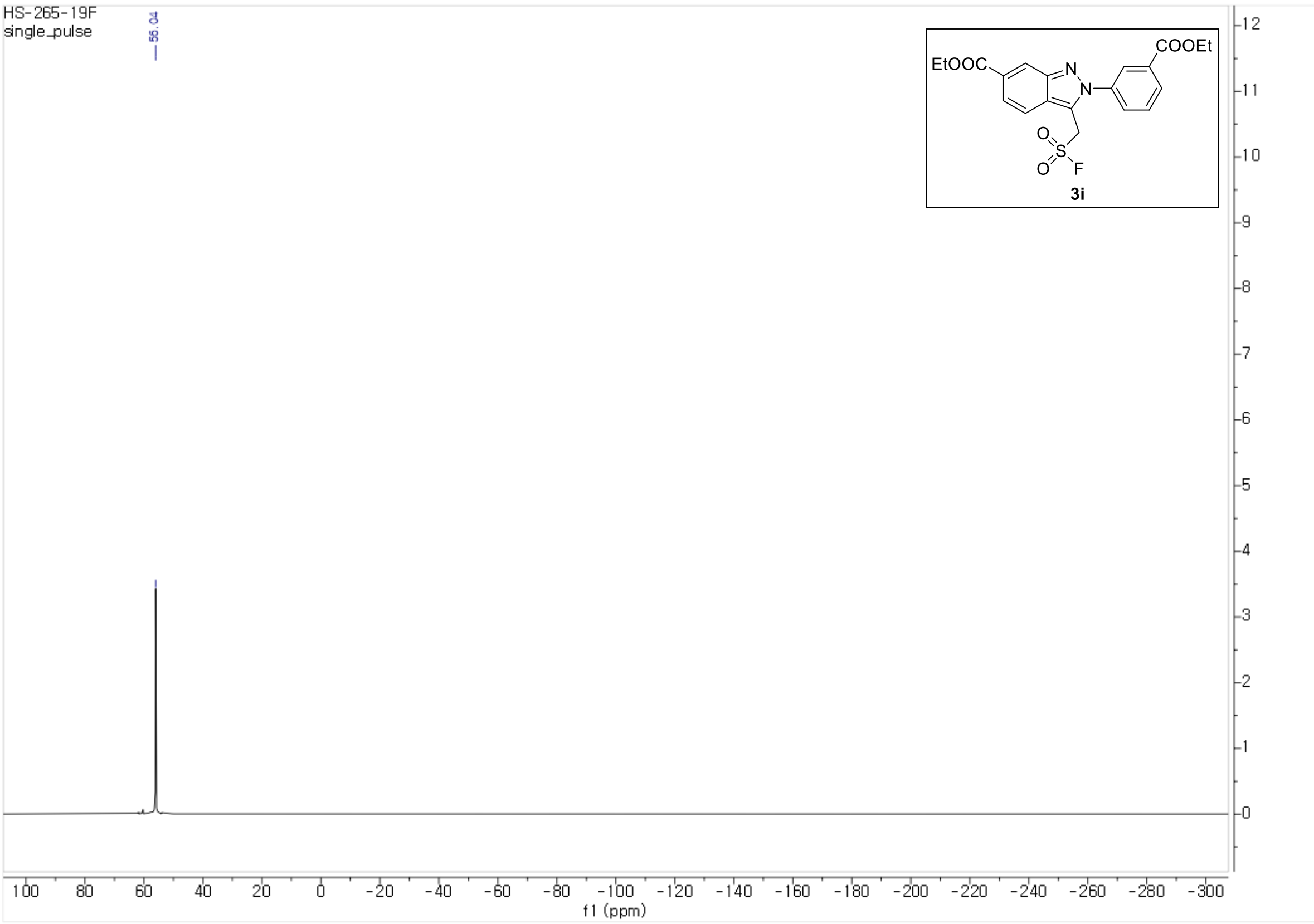
HS-265-19F
single_pulse
56.04
EtOOC
COOEt
N
N
O
S
O
F
3i
12
11
10
9
8
7
6
5
4
3
2
1
0
100
80
60
40
20
0
-20
-40
-60
-80
-100
-120
-140
-160
-180
-200
-220
-240
-260
-280
-300
f1 (ppm)

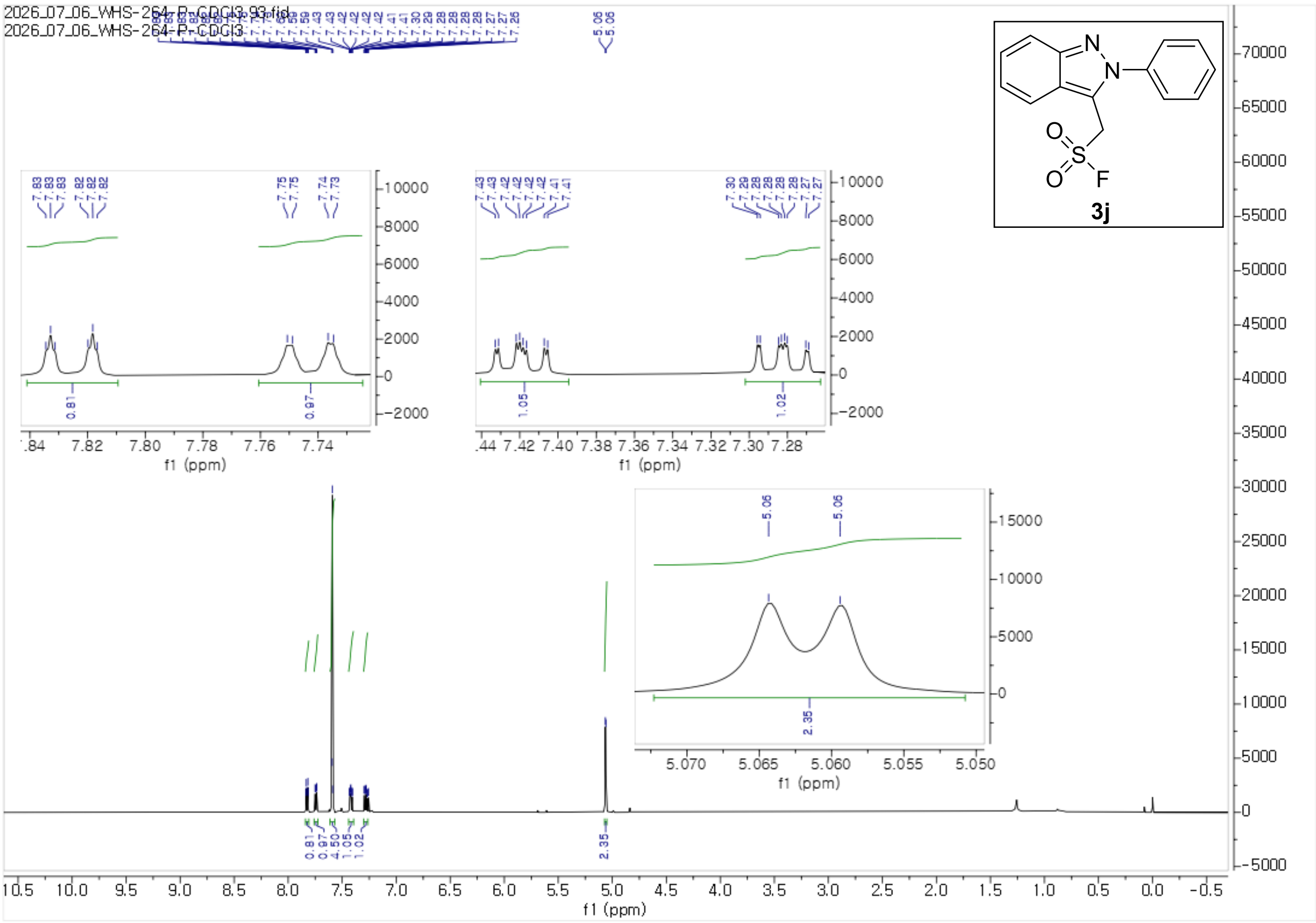
2026_07_06_WHS-264-P-CDCl3.93.fid
2026_07_06_WHS-264-P-CDCl3
3j
f1 (ppm)

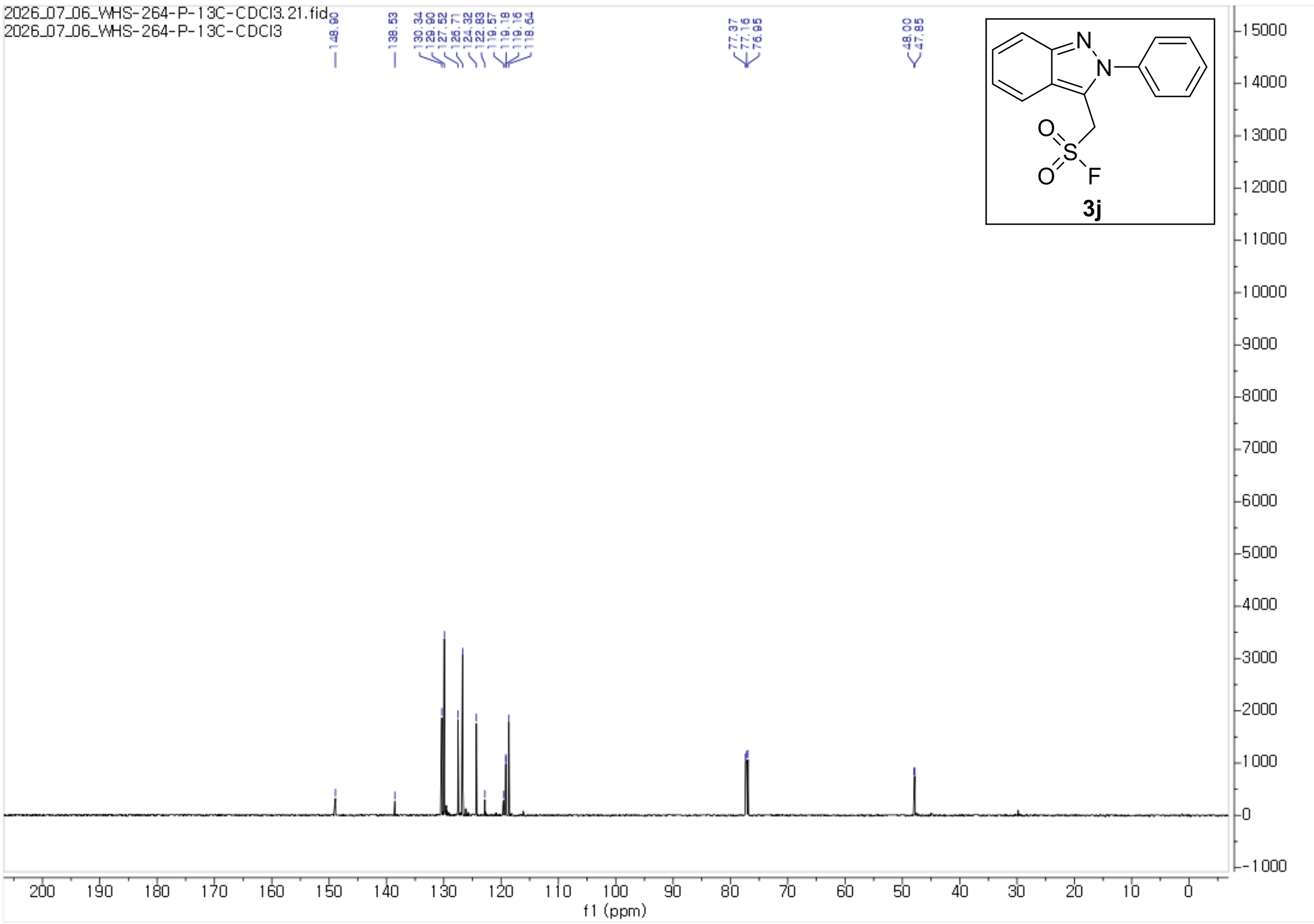
2026_07_06_WHS-264-P-13C-CDCl3.21.fid
2026_07_06_WHS-264-P-13C-CDCl3
148.90
138.53
130.34
129.90
127.52
126.71
124.32
122.83
119.57
119.18
119.16
118.64
77.37
77.16
76.95
48.00
47.85
3j
f1 (ppm)

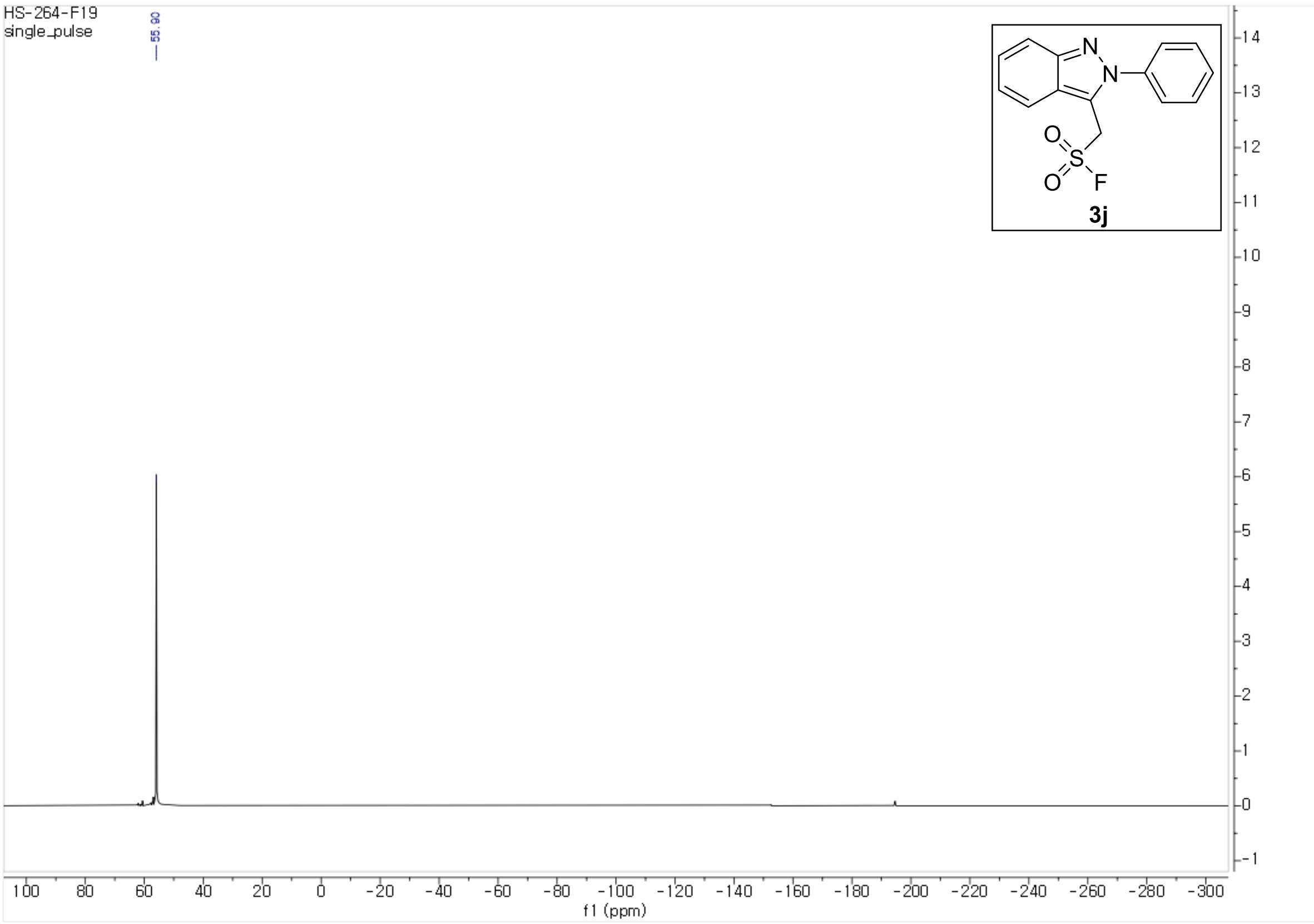
HS-264-F19
single_pulse
55.90
3j
f1 (ppm)

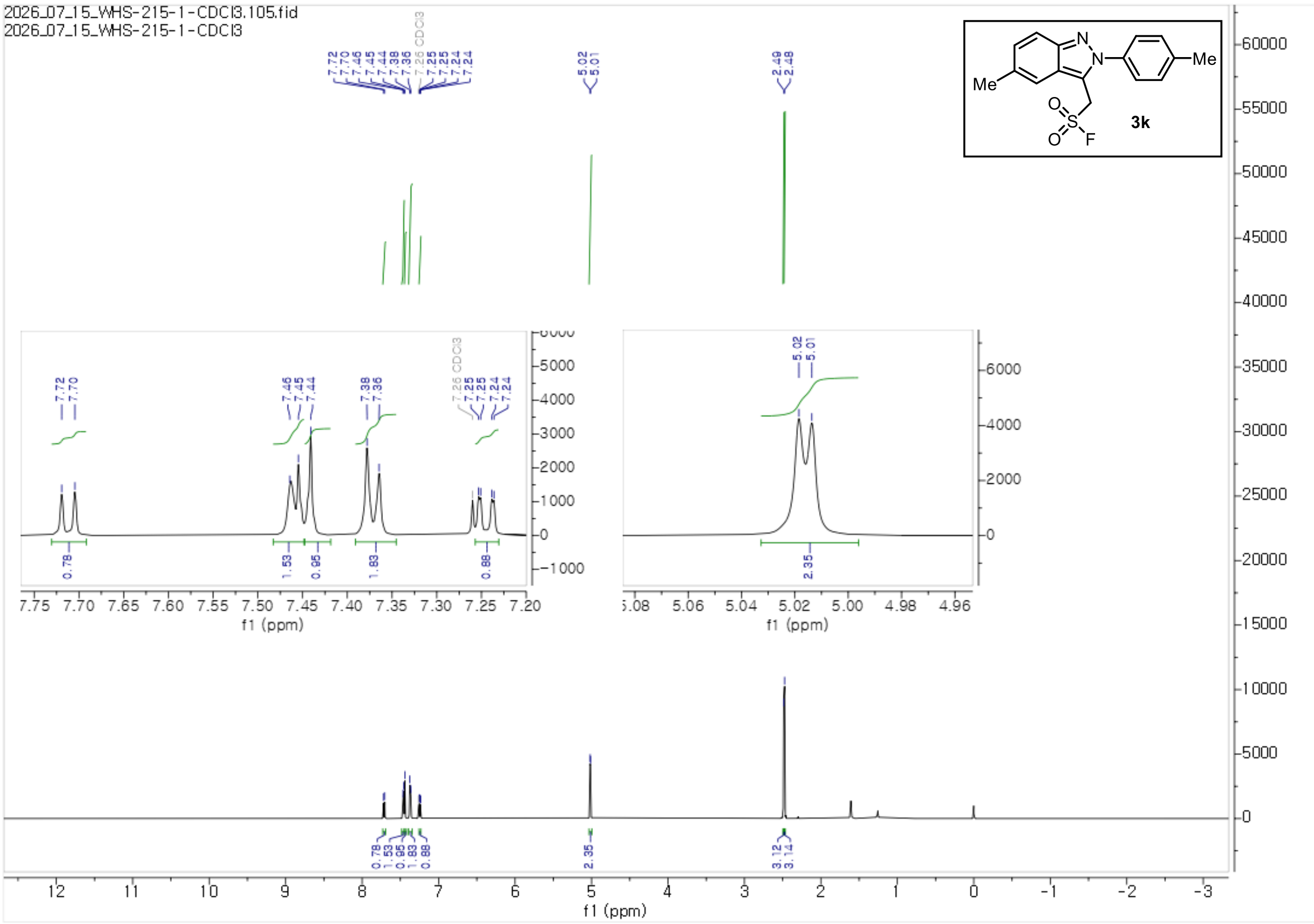
2026_07_15_WHS-215-1-CDCl3.105.fid
2026_07_15_WHS-215-1-CDCl3
3k
f1 (ppm)

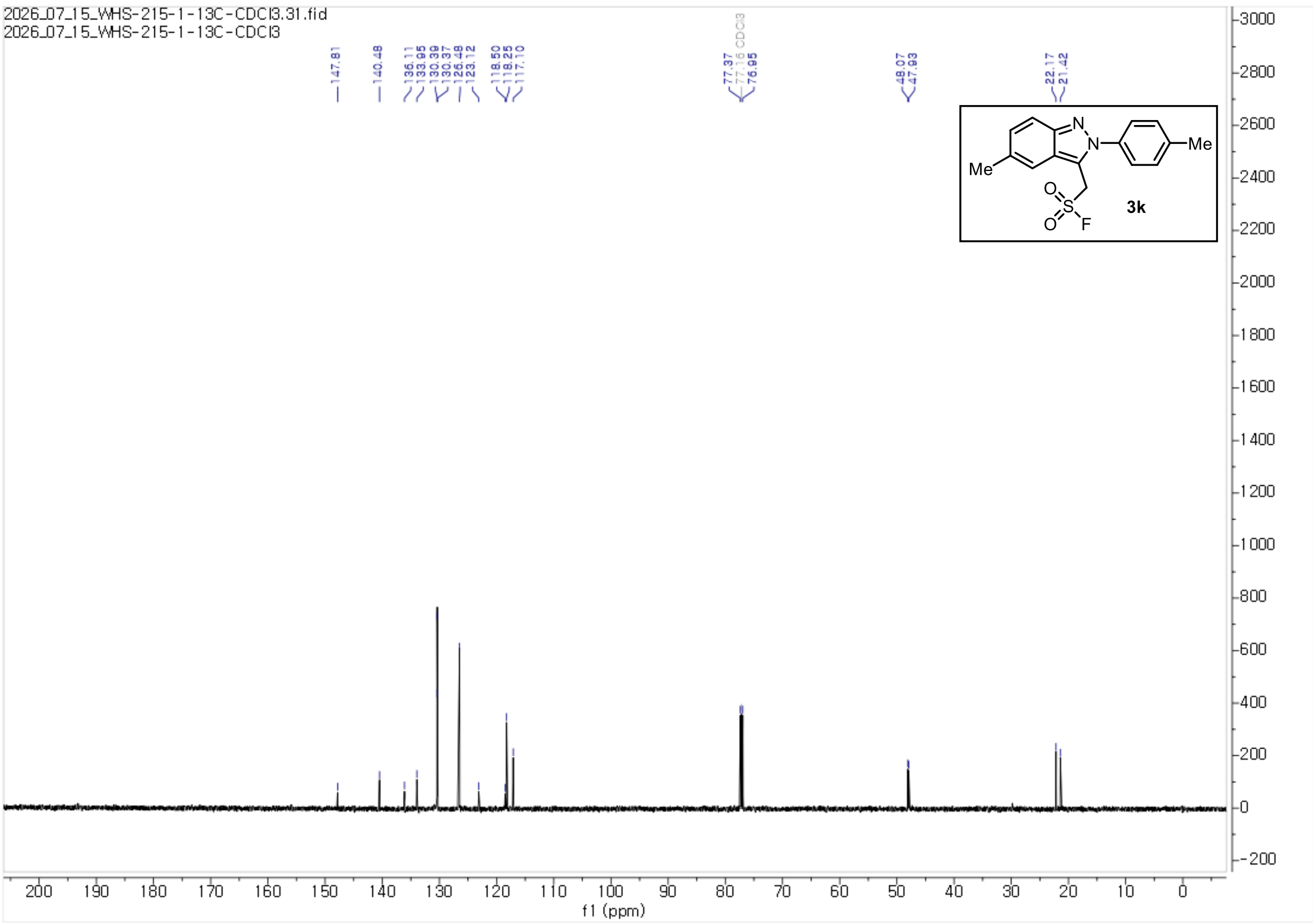
2026_07_15_WHS-215-1-13C-CDCl3.31.fid
2026_07_15_WHS-215-1-13C-CDCl3
147.81
140.48
136.11
133.95
130.39
130.37
126.48
123.12
118.50
118.25
117.10
77.37
77.16 CDCl3
76.95
48.07
47.93
22.17
21.42
3k
f1 (ppm)

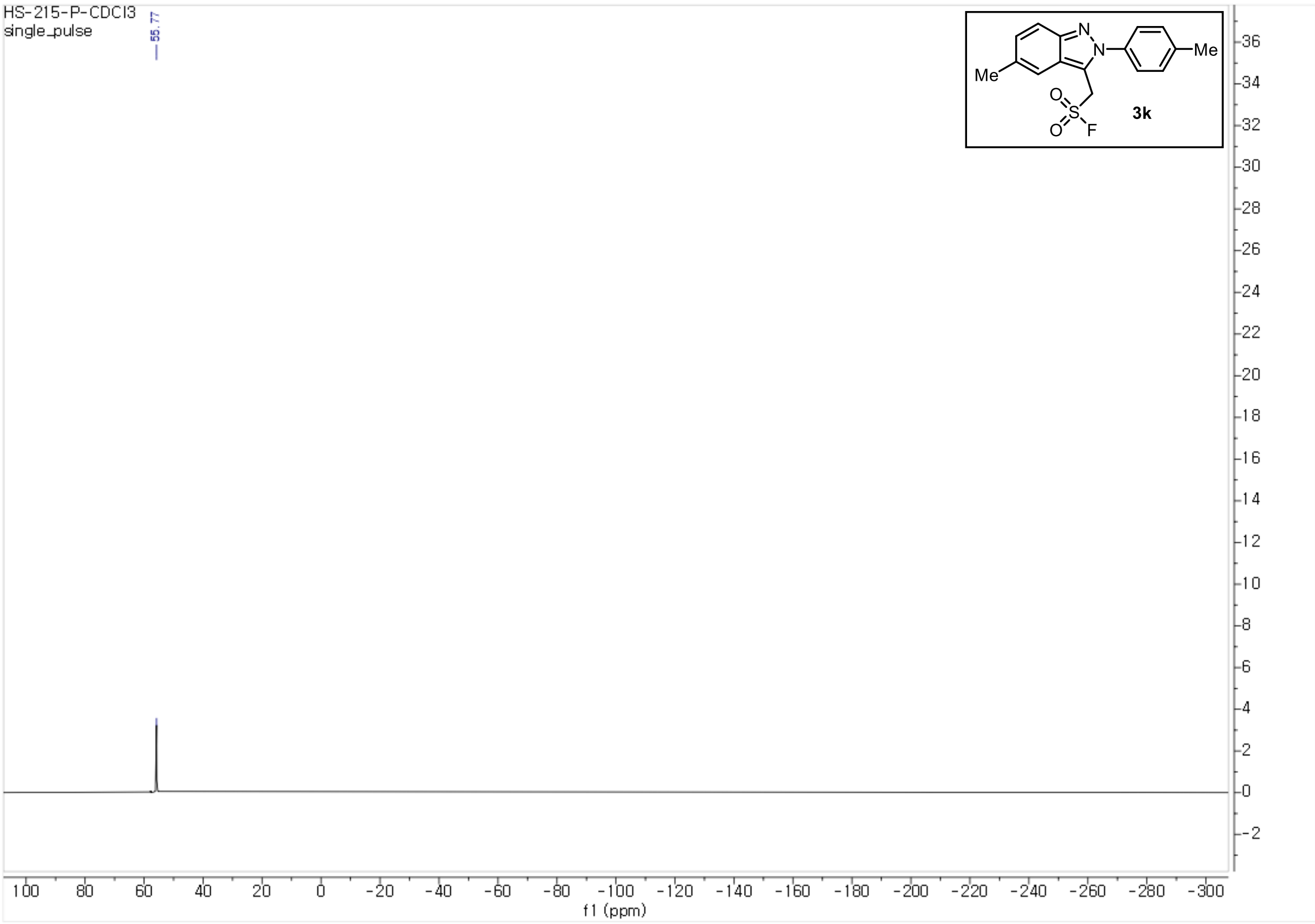
HS-215-P-CDCl3
single_pulse
55.77
Me
N
N
Me
O
S
O
F
3k
f1 (ppm)

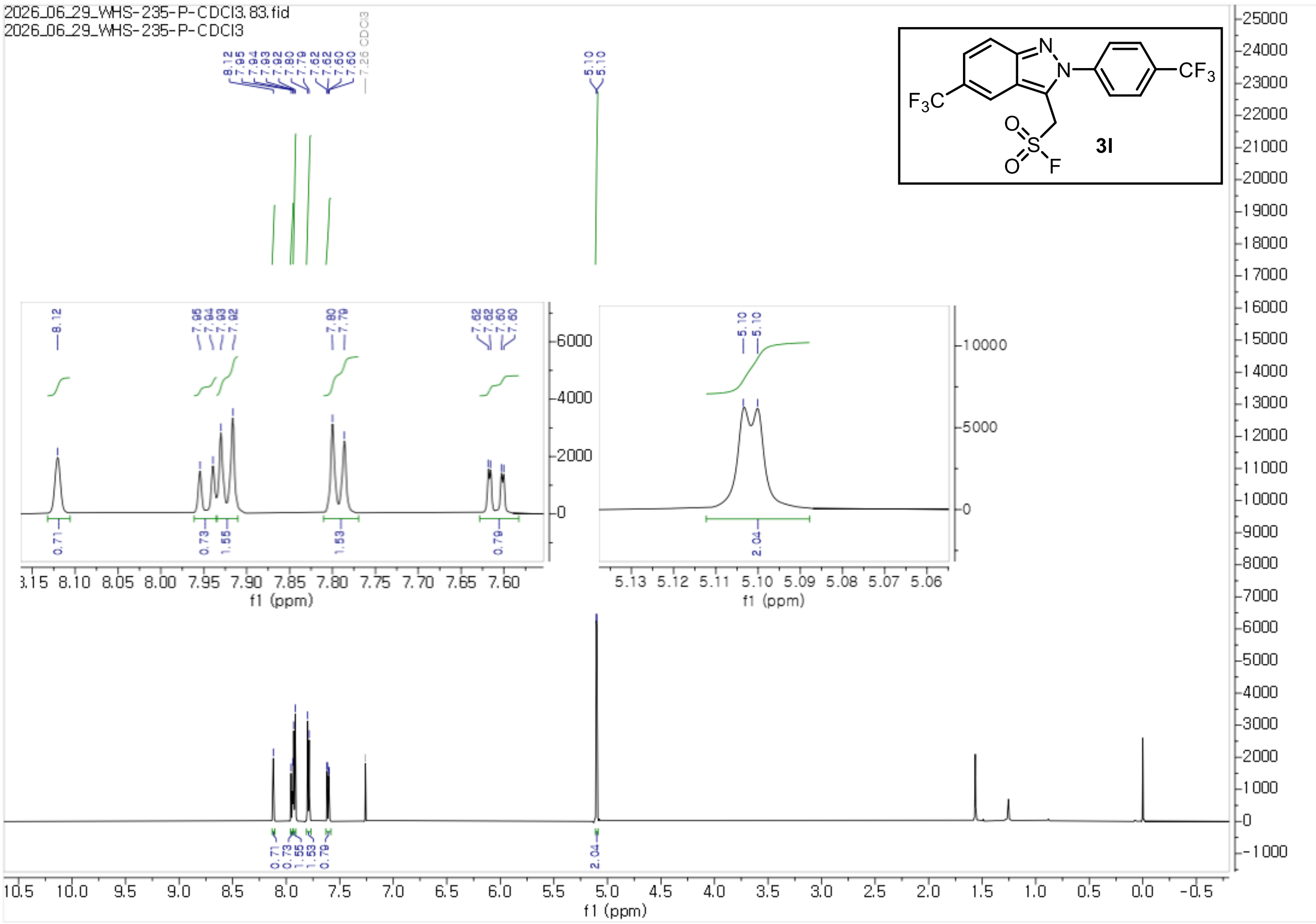
2026_06_29_WHS-235-P-CDCl3.83.fid
2026_06_29_WHS-235-P-CDCl3
7.26 CDCl3
N
N
CF3
F3C
O
S
O
F
3l
f1 (ppm)

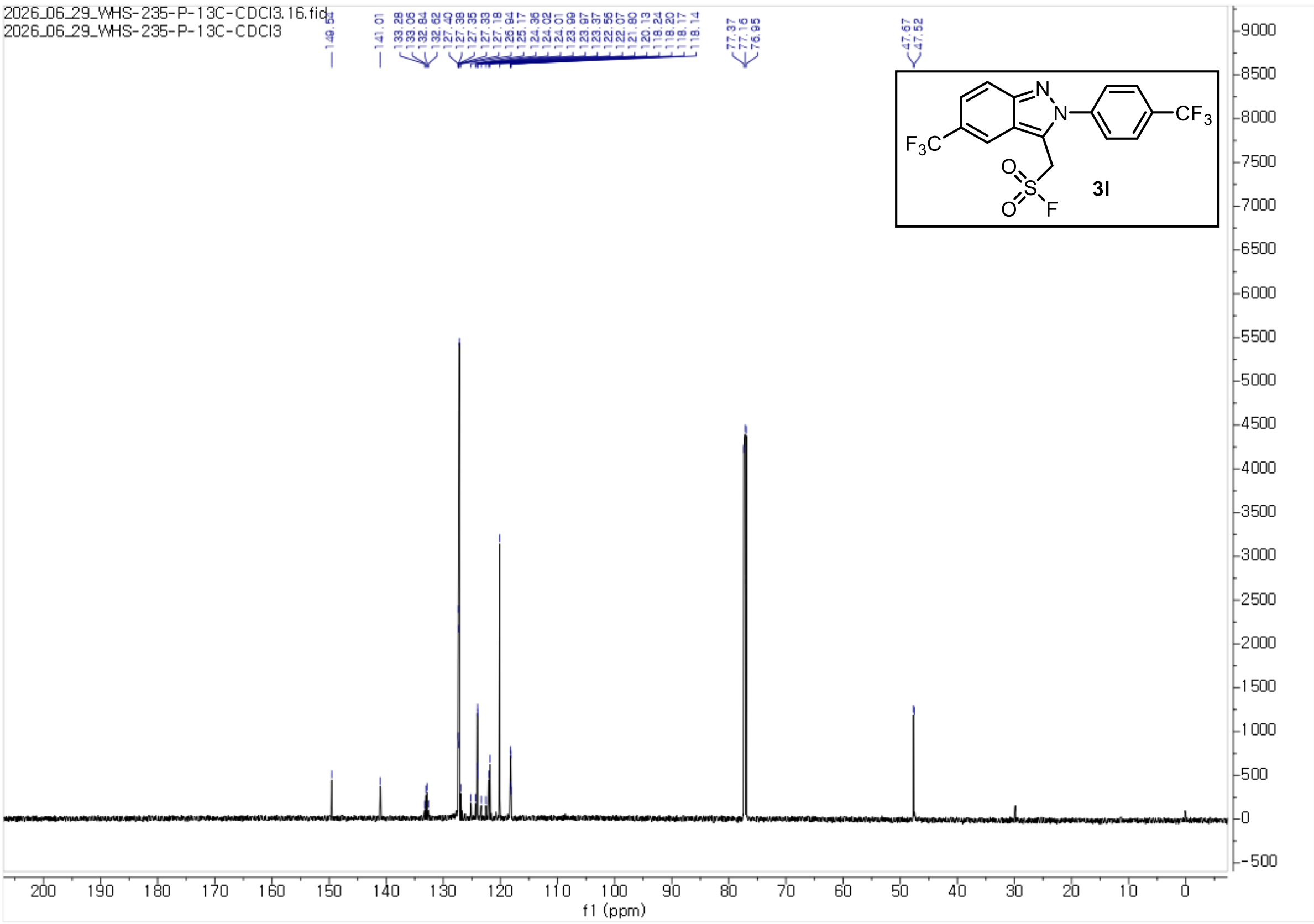
2026_06_29_WHS-235-P-13C-CDCl3.16.fid
2026_06_29_WHS-235-P-13C-CDCl3
N
N
CF3
F3C
O
S
O
F
3l
f1 (ppm)

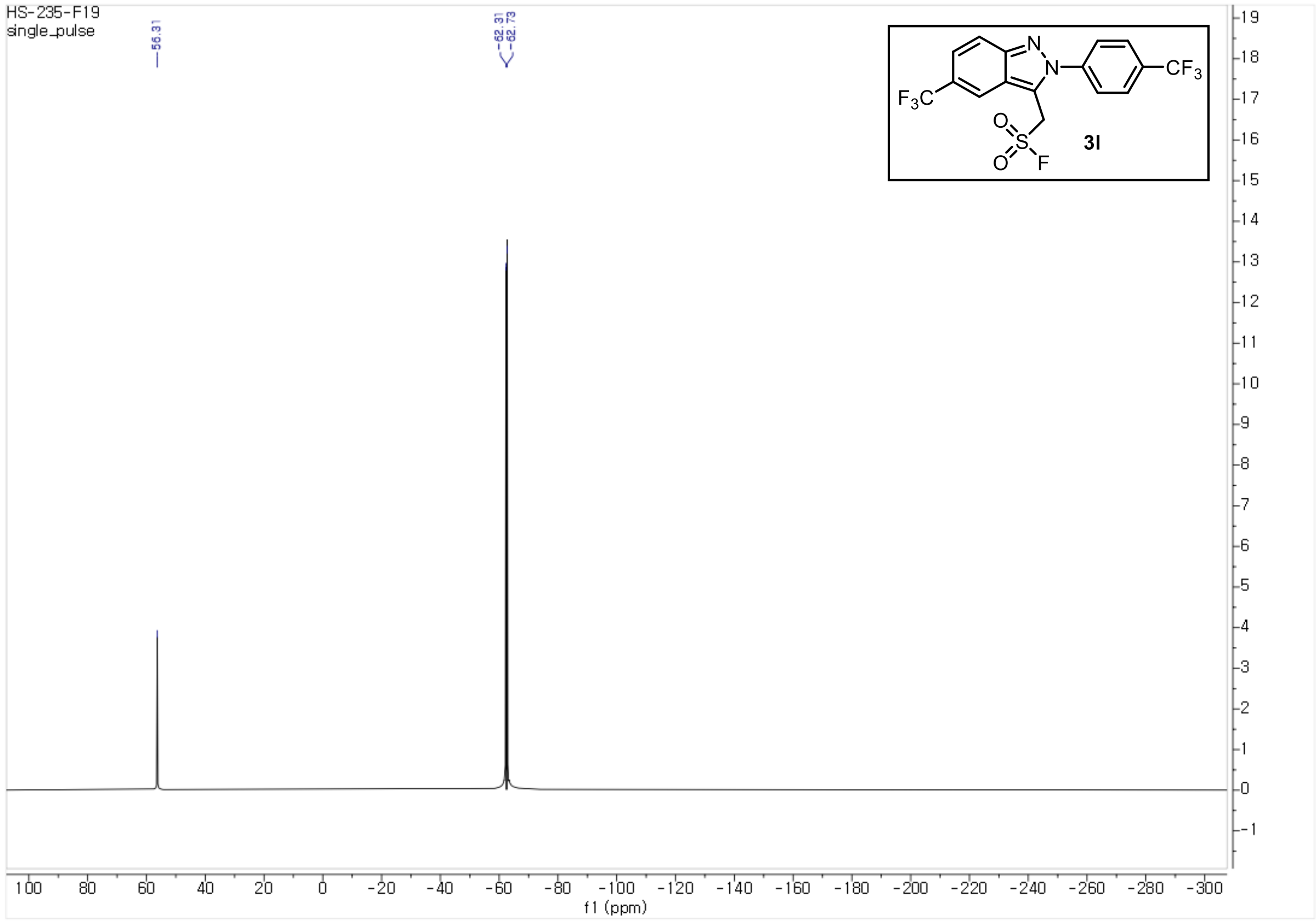
HS-235-F19
single_pulse
—56.31
-62.31
-62.73
N
N
CF3
F3C
O
S
O
F
3l
f1 (ppm)

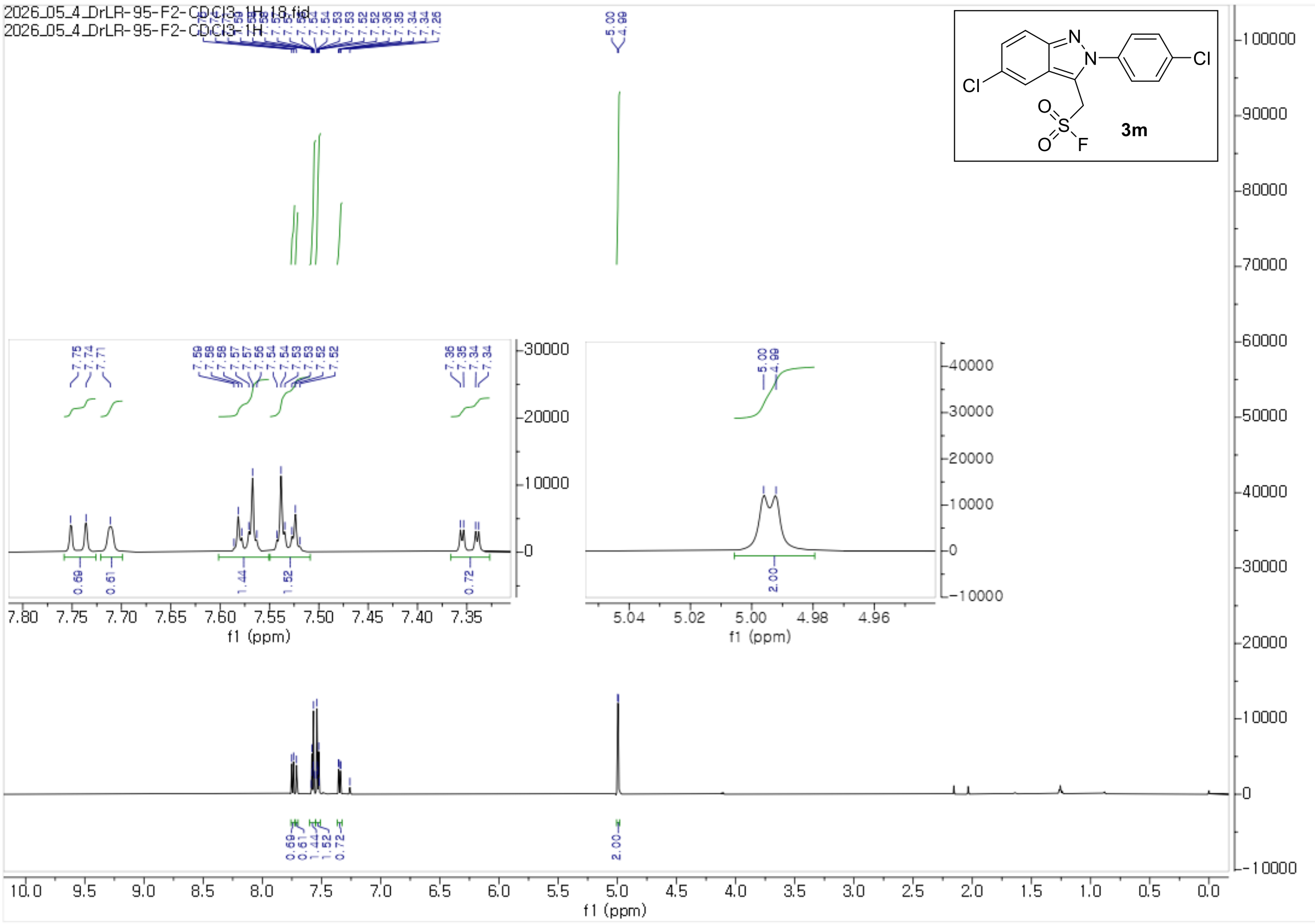
2026_05_4_DrLR-95-F2-CDCl3-1H.18.fid
2026_05_4_DrLR-95-F2-CDCl3-1H
3m
f1 (ppm)

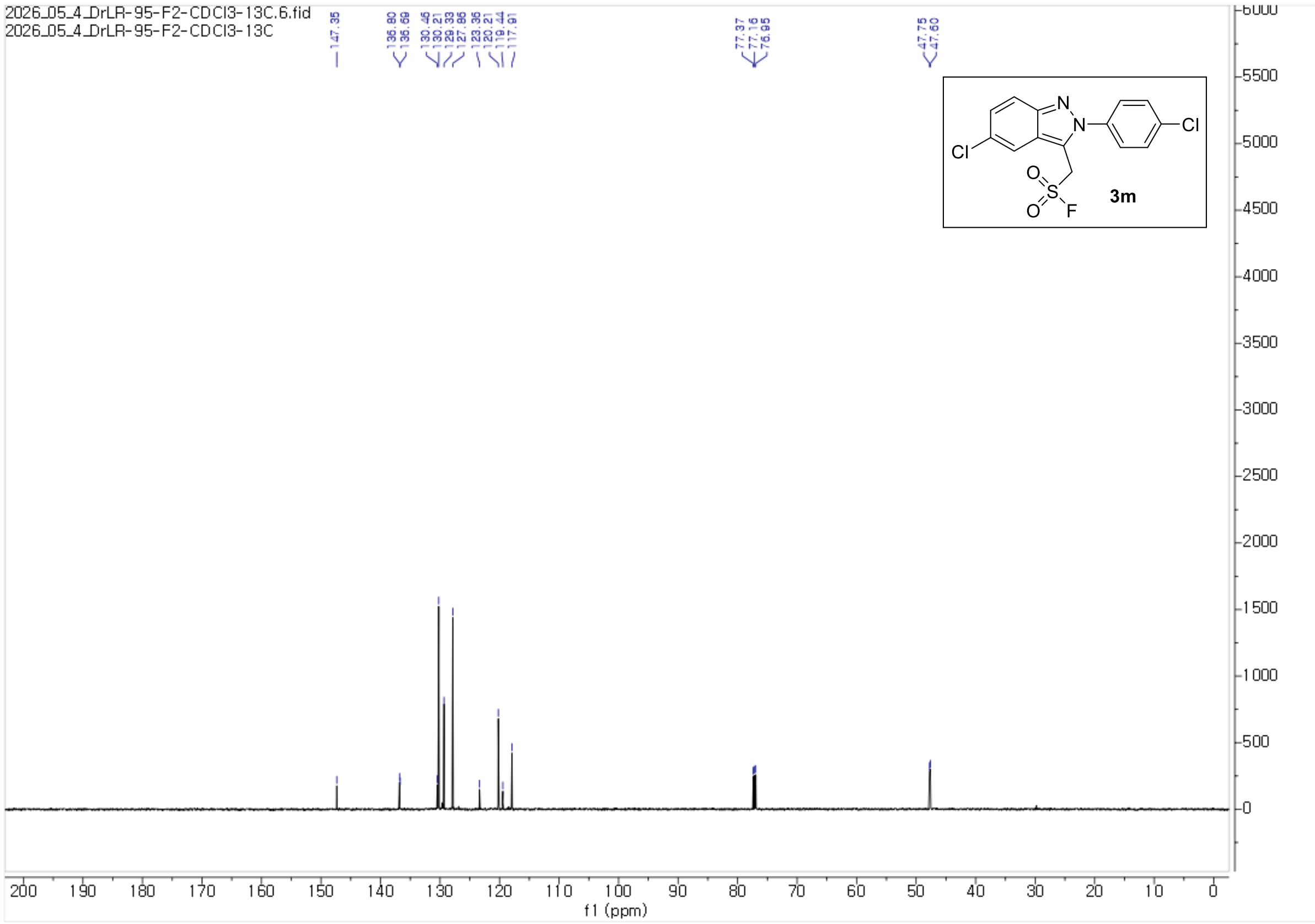
2026_05_4_DrLR-95-F2-CDCl3-13C.6.fid
2026_05_4_DrLR-95-F2-CDCl3-13C
147.35
136.80
136.69
130.45
130.21
129.33
127.86
123.36
120.21
119.44
117.91
77.37
77.16
76.95
47.75
47.60
3m
f1 (ppm)

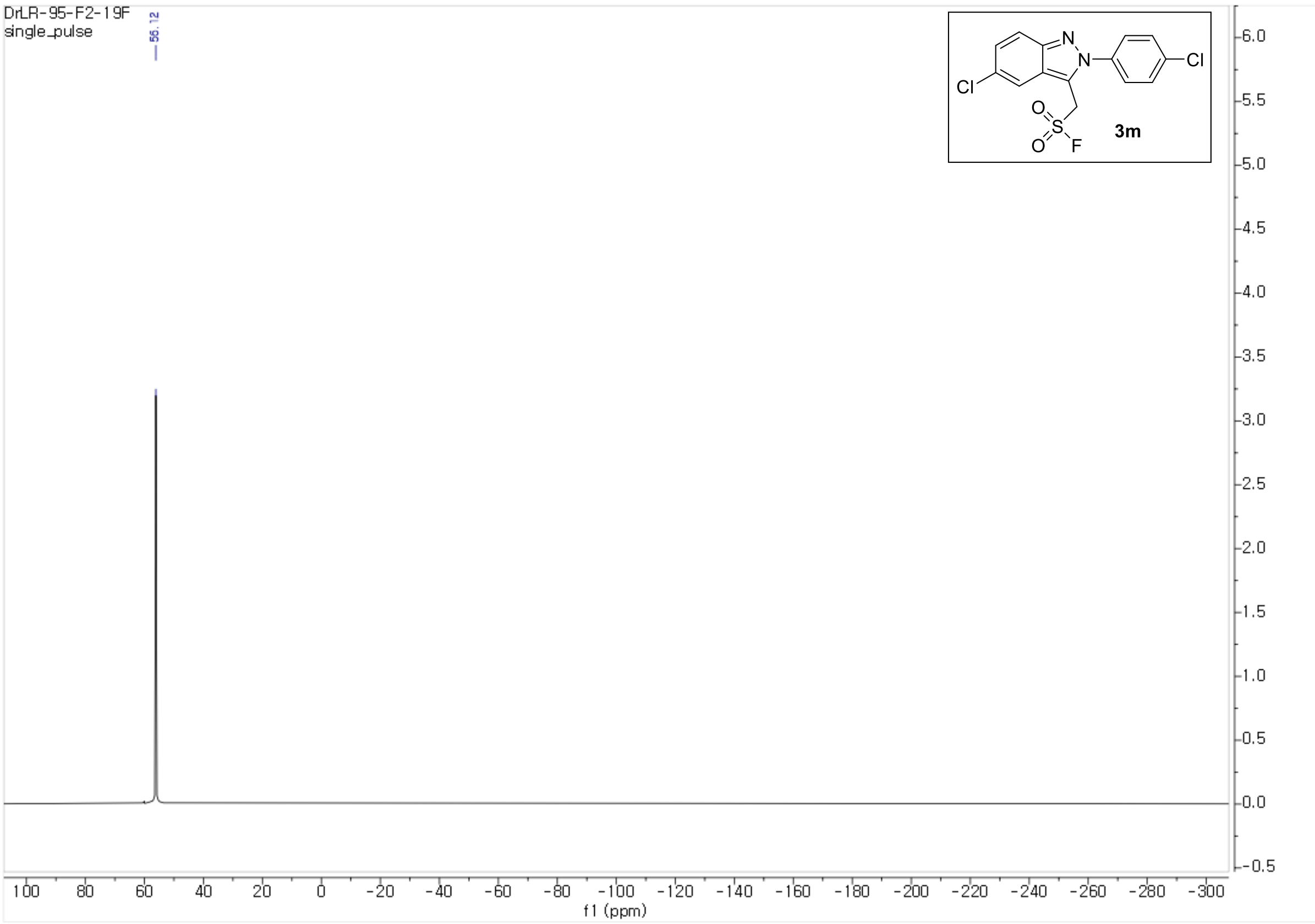
DrLR-95-F2-19F
single_pulse
56.12
Cl
N
N
Cl
O
S
O
F
3m
f1 (ppm)

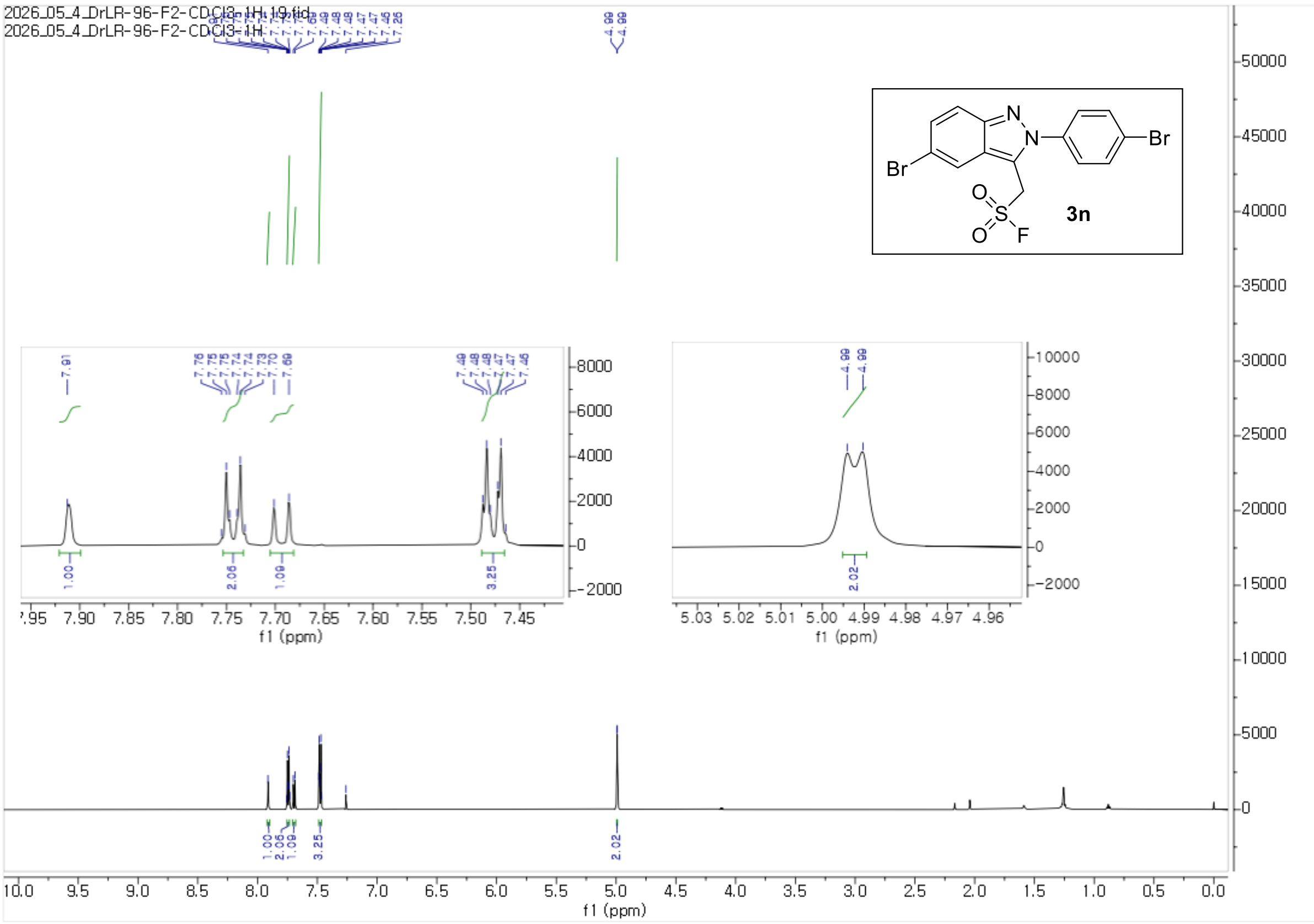
2026_05_4_DrLR-96-F2-CDCl3-1H.19.fid
2026_05_4_DrLR-96-F2-CDCl3-1H
3n
f1 (ppm)

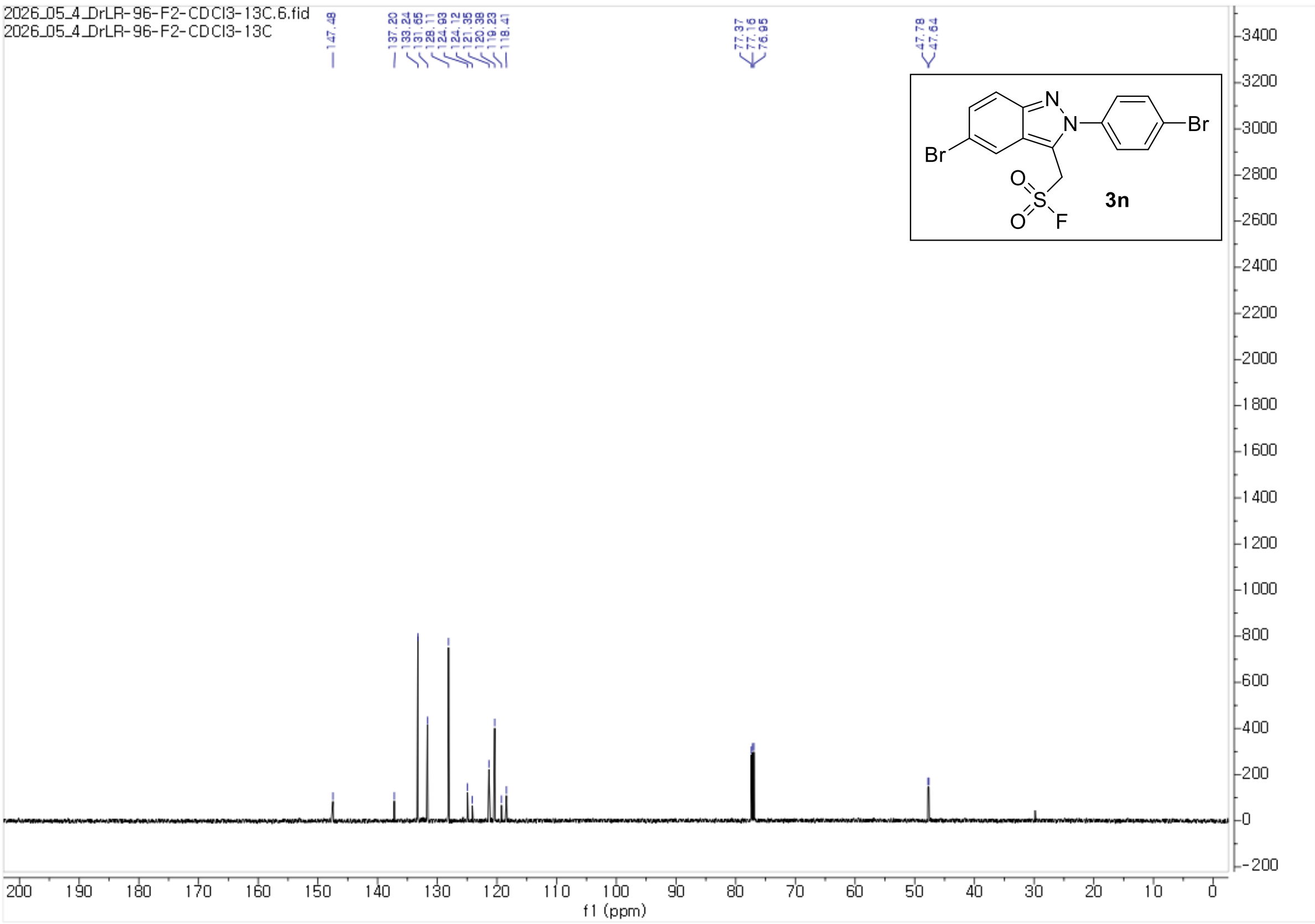
2026_05_4_DrLR-96-F2-CDCl3-13C.6.fid
2026_05_4_DrLR-96-F2-CDCl3-13C
147.48
137.20
133.24
131.65
128.11
124.93
124.12
121.35
120.38
119.23
118.41
77.37
77.16
76.95
47.78
47.64
3n
f1 (ppm)

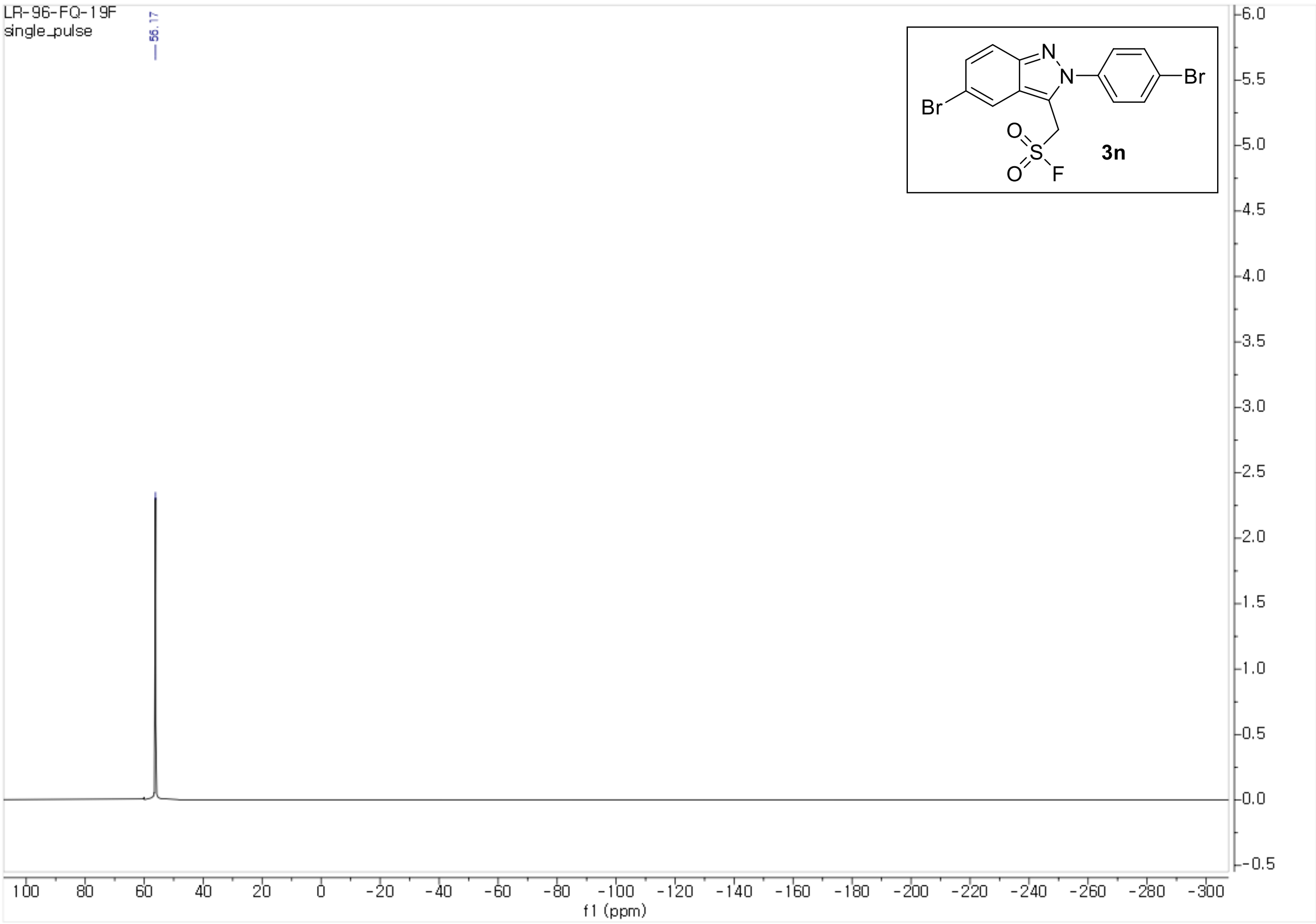
LR-96-FQ-19F
single_pulse
56.17
Br
N
N
Br
O
S
O
F
3n
f1 (ppm)

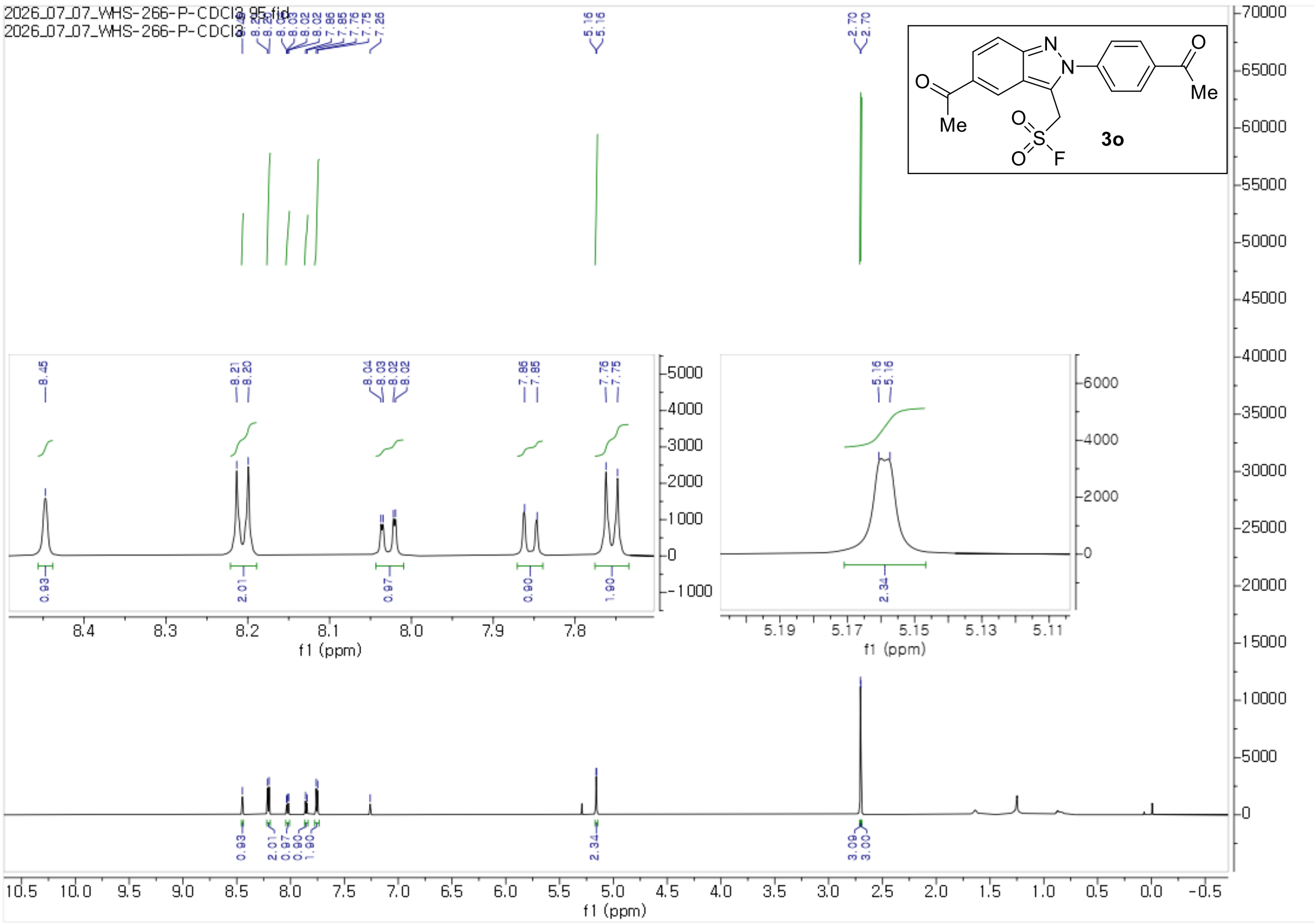
2026_07_07_WHS-266-P-CDCl3.95.fid
2026_07_07_WHS-266-P-CDCl3
3o
f1 (ppm)

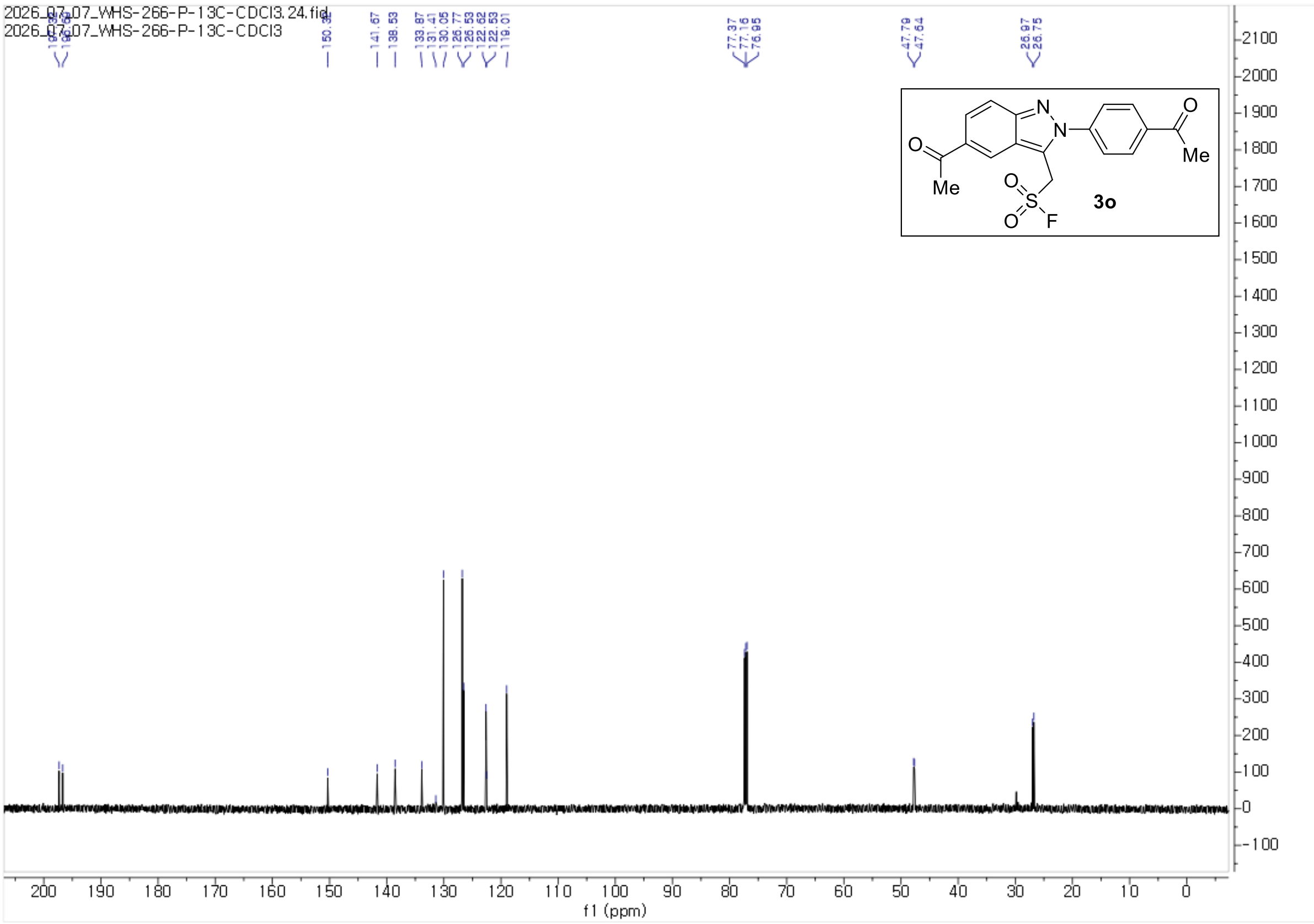
2026_07_07_WHS-266-P-13C-CDCl3.24.fid
2026_07_07_WHS-266-P-13C-CDCl3
3o
f1 (ppm)

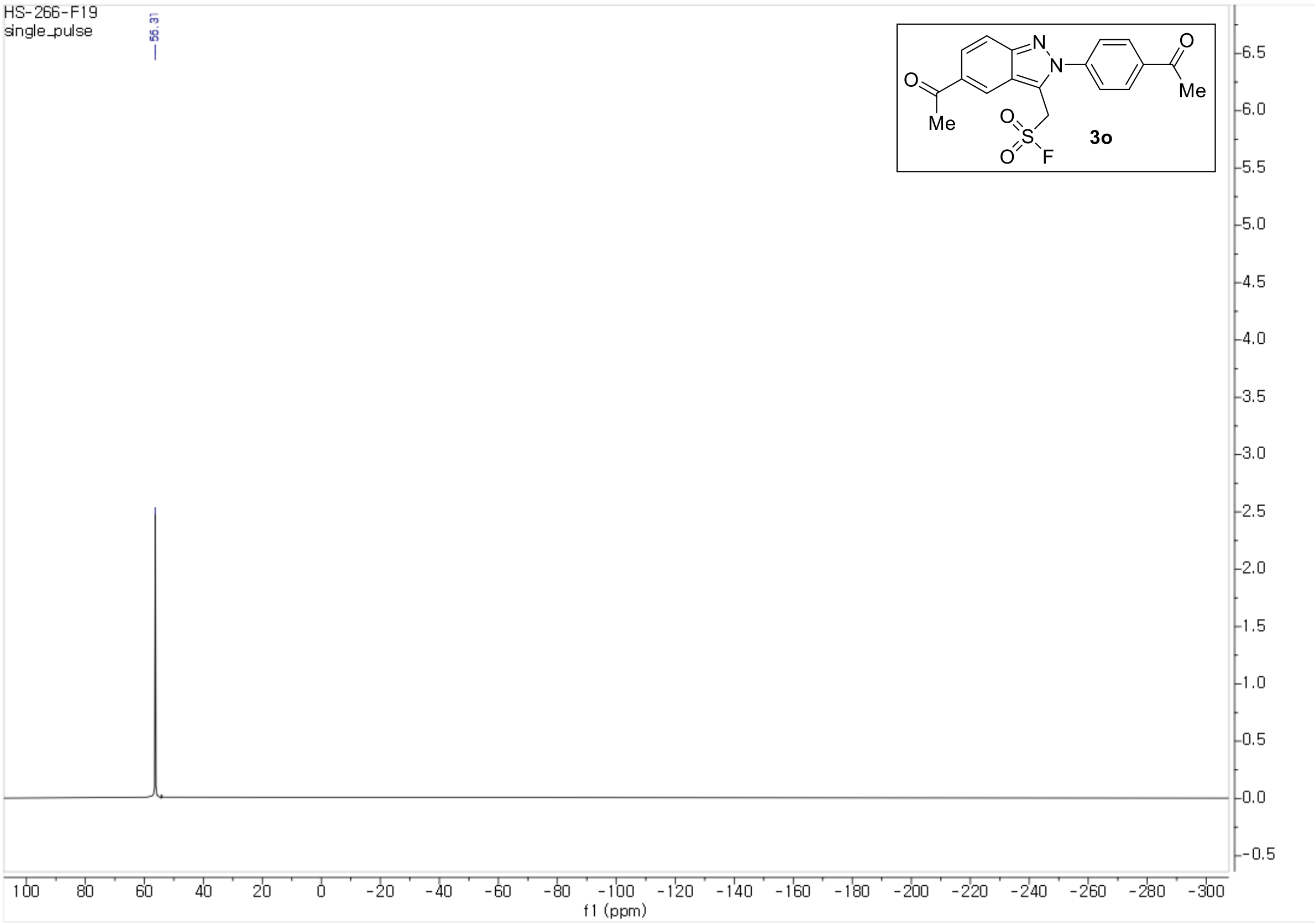

HS-266-F19
single_pulse
55.31
O
Me
N
N
O
Me
O
S
O
F
3o
6.5
6.0
5.5
5.0
4.5
4.0
3.5
3.0
2.5
2.0
1.5
1.0
0.5
0.0
-0.5
100 80 60 40 20 0 -20 -40 -60 -80 -100 -120 -140 -160 -180 -200 -220 -240 -260 -280 -300
f1 (ppm)

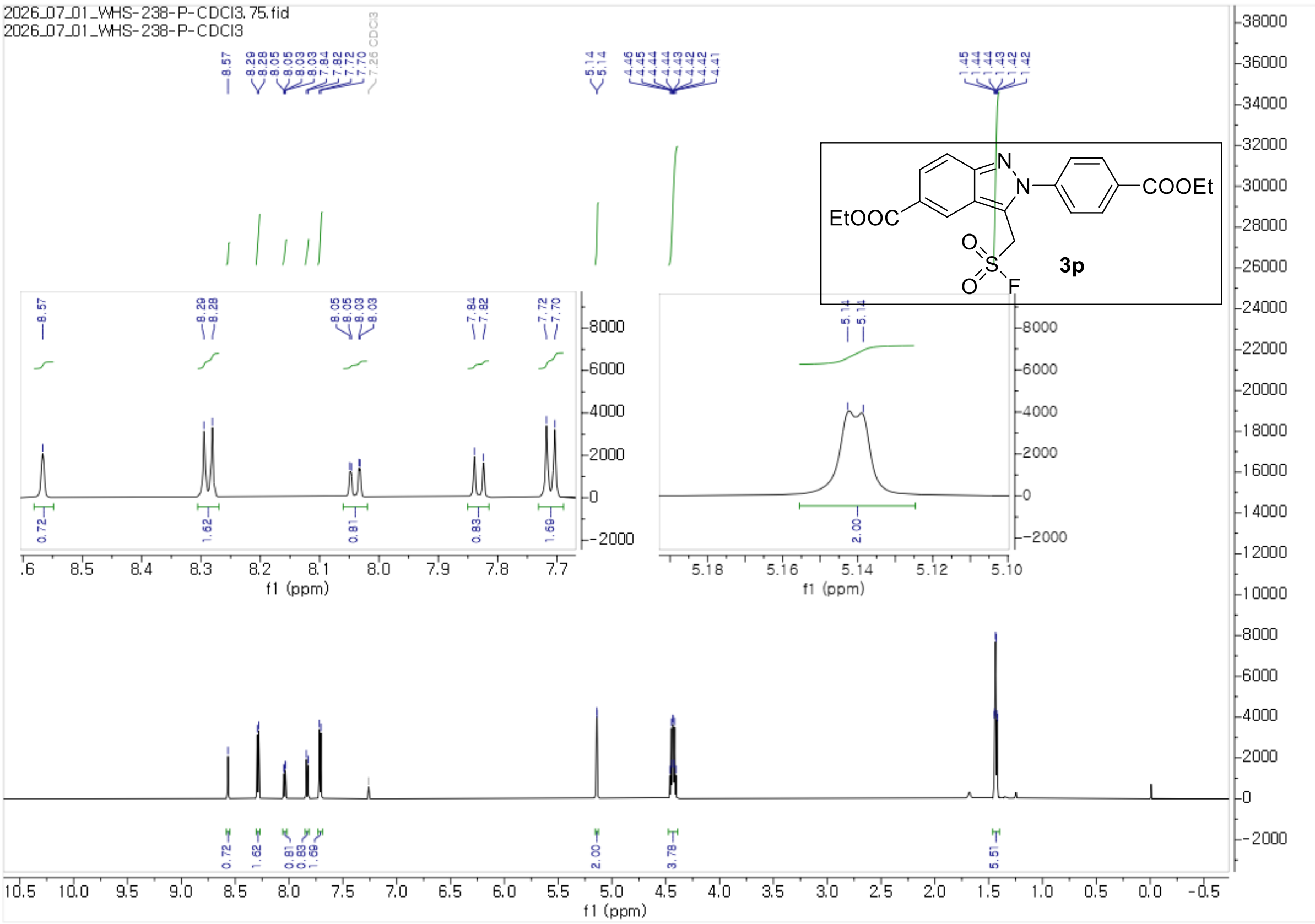
2026_07_01_WHS-238-P-CDCl3.75.fid
2026_07_01_WHS-238-P-CDCl3
EtOOC
COOEt
3p
f1 (ppm)

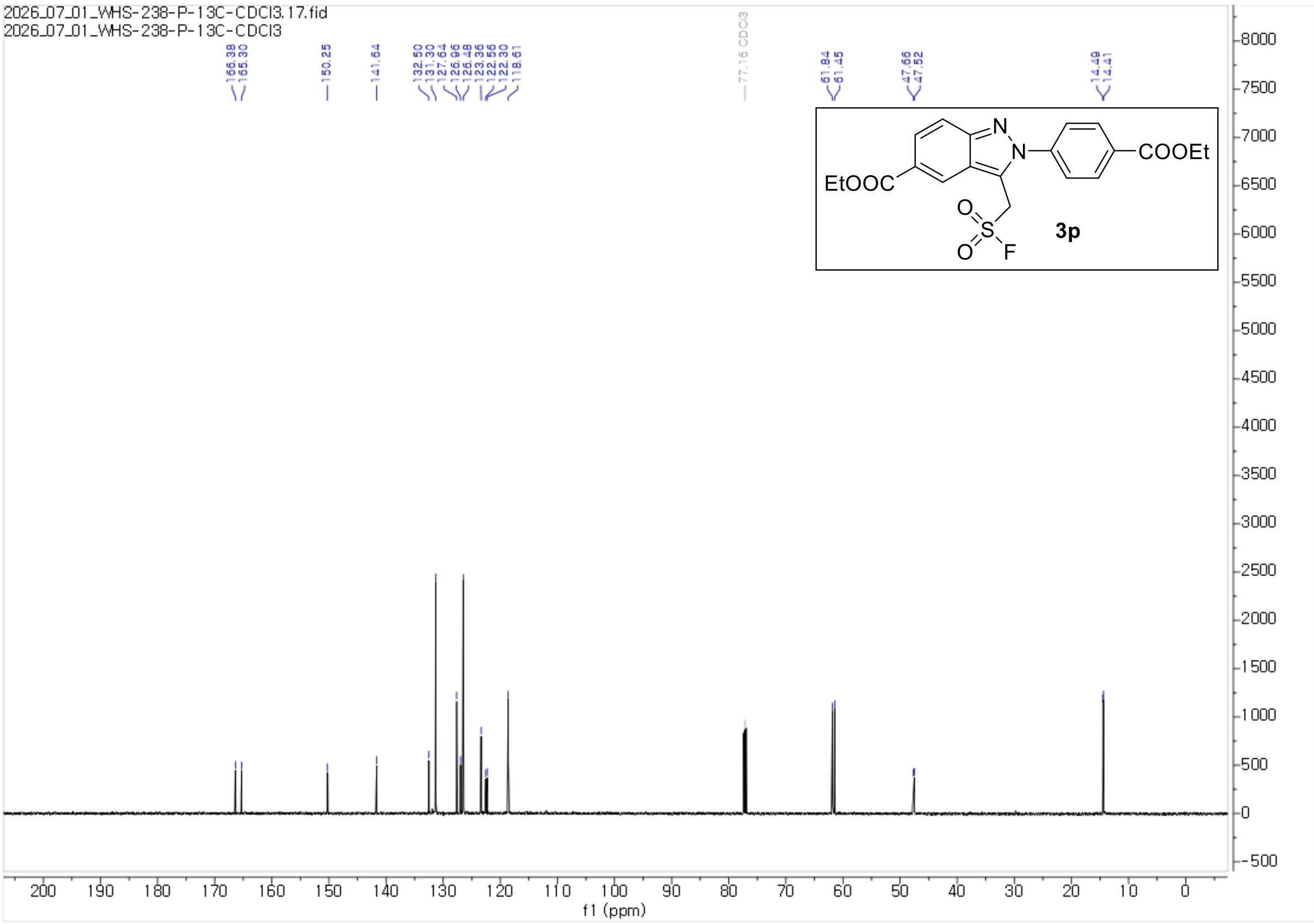
2026_07_01_WHS-238-P-13C-CDCl3.17.fid
2026_07_01_WHS-238-P-13C-CDCl3
EtOOC
COOEt
3p
f1 (ppm)

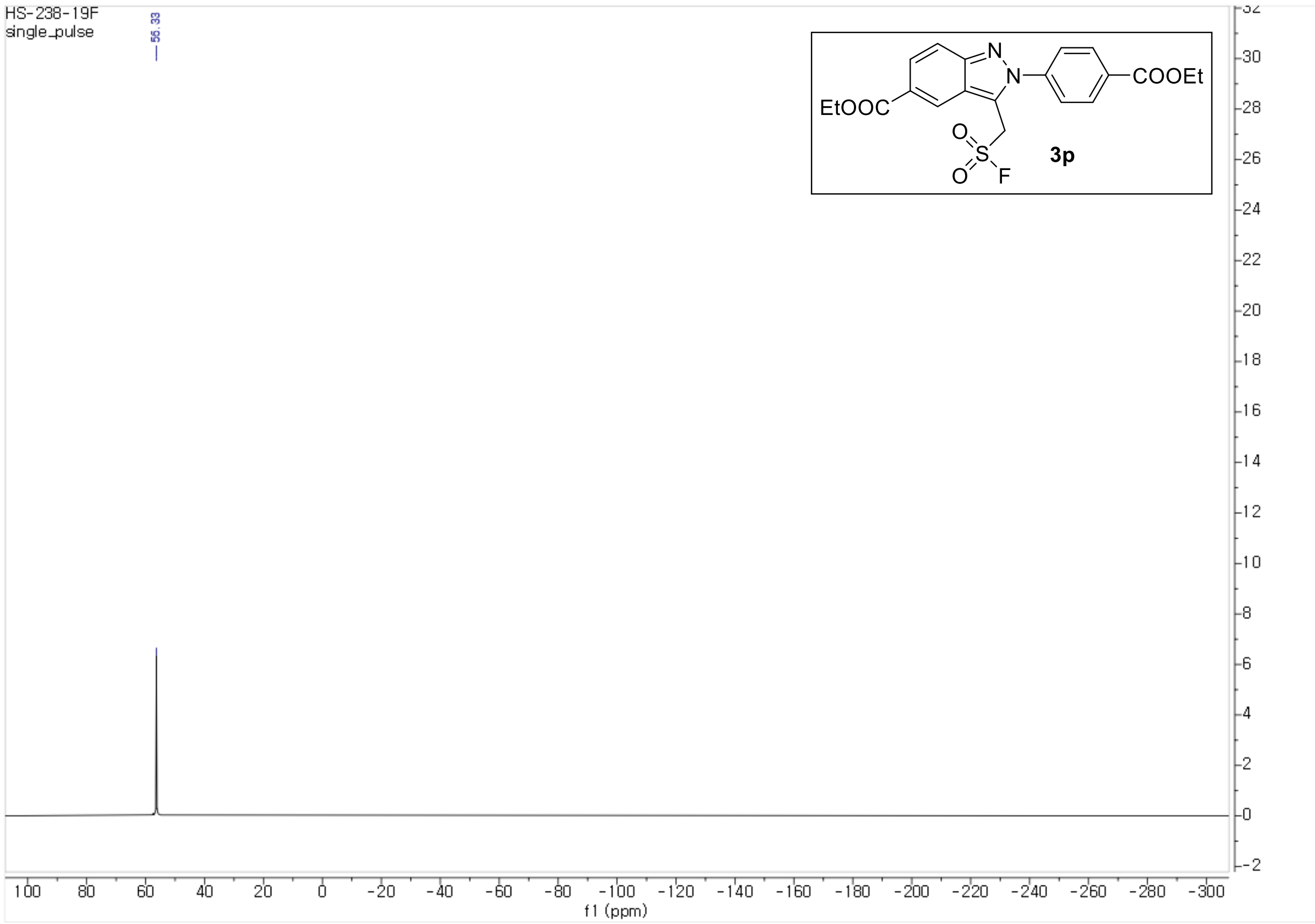
HS-238-19F
single_pulse
55.33
EtOOC
N
N
COOEt
O
S
O
F
3p
f1 (ppm)

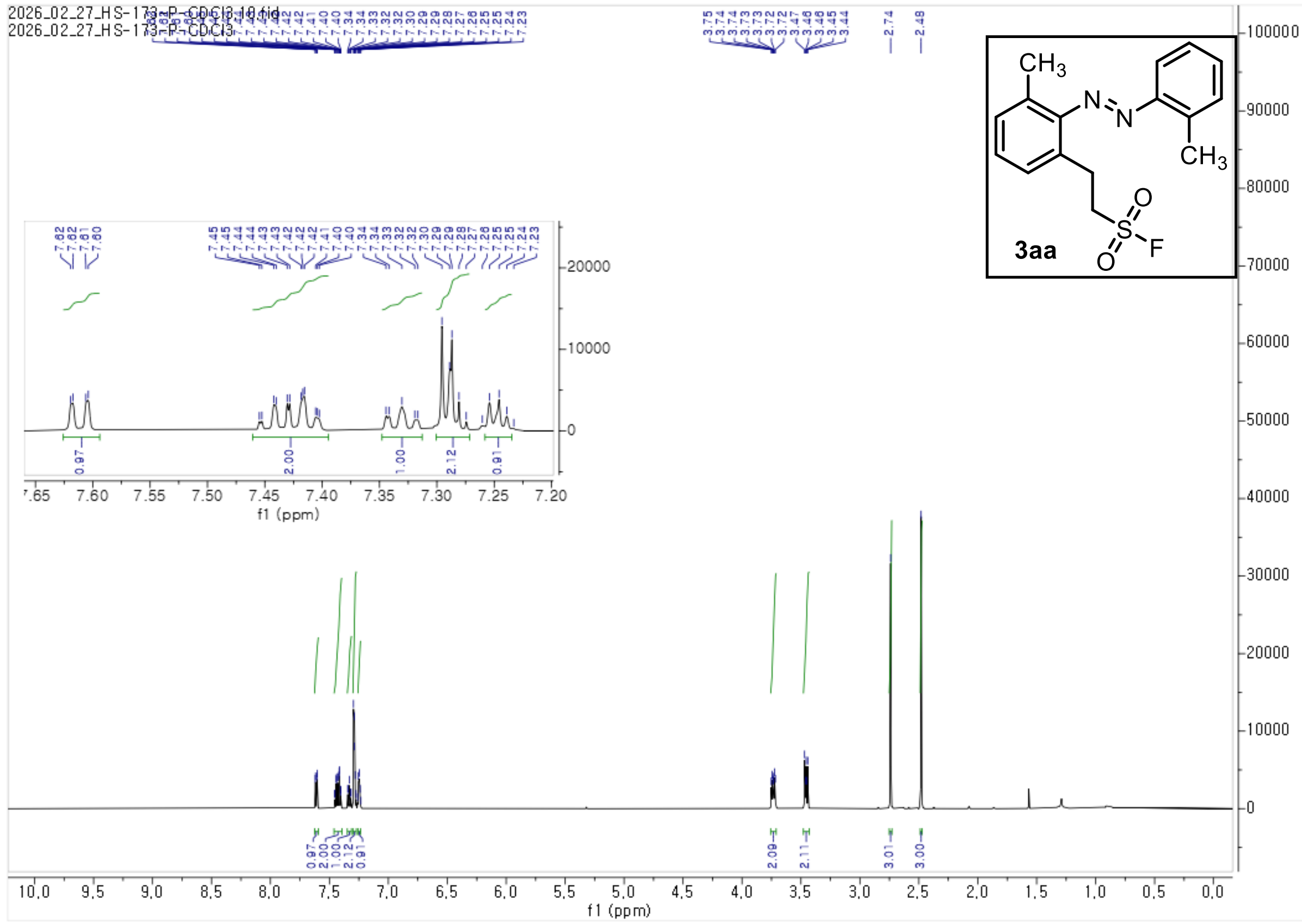
2026_02_27_HS-173-P-CDCl3.10.fid
2026_02_27_HS-173-P-CDCl3
7.62 7.62 7.61 7.60 7.45 7.44 7.44 7.43 7.43 7.42 7.42 7.41 7.40 7.40 7.34 7.34 7.33 7.32 7.32 7.30 7.29 7.29 7.28 7.27 7.26 7.25 7.25 7.24 7.23
3.75 3.74 3.73 3.73 3.72 3.47 3.46 3.46 3.45 3.44
2.74
2.48
0.97 2.00 1.00 2.12 0.91
2.09 2.11 3.01 3.00
f1 (ppm)
CH3
N
N
CH3
O
S
F
O
3aa

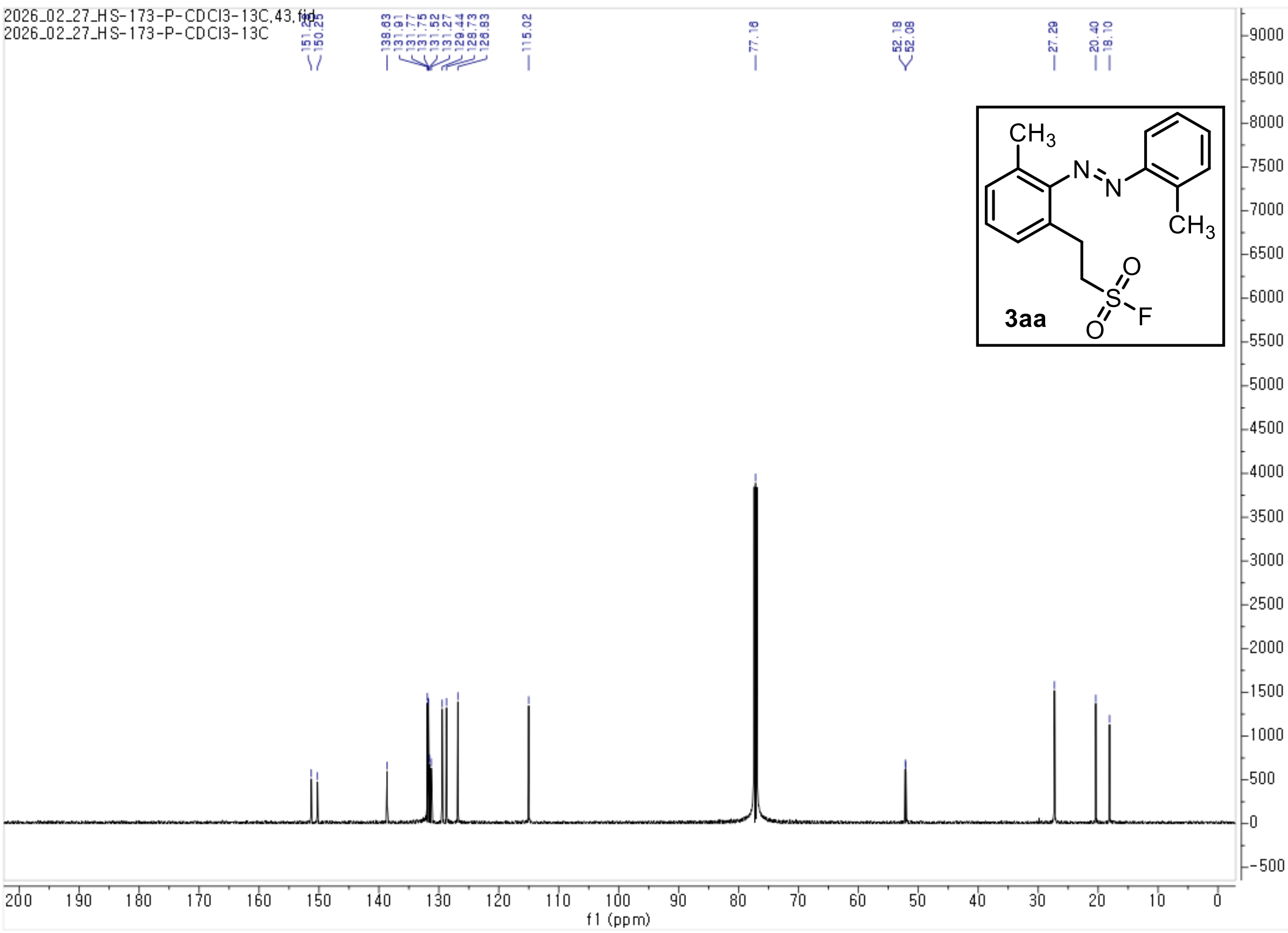
2026_02_27_HS-173-P-CDCl3-13C.43.fid
2026_02_27_HS-173-P-CDCl3-13C
151.26 150.25
138.63 131.91 131.75 131.52 131.27 129.44 128.73 126.83
115.02
77.16
52.18 52.08
27.29
20.40 18.10
f1 (ppm)
CH3
N
N
CH3
O
S
F
O
3aa

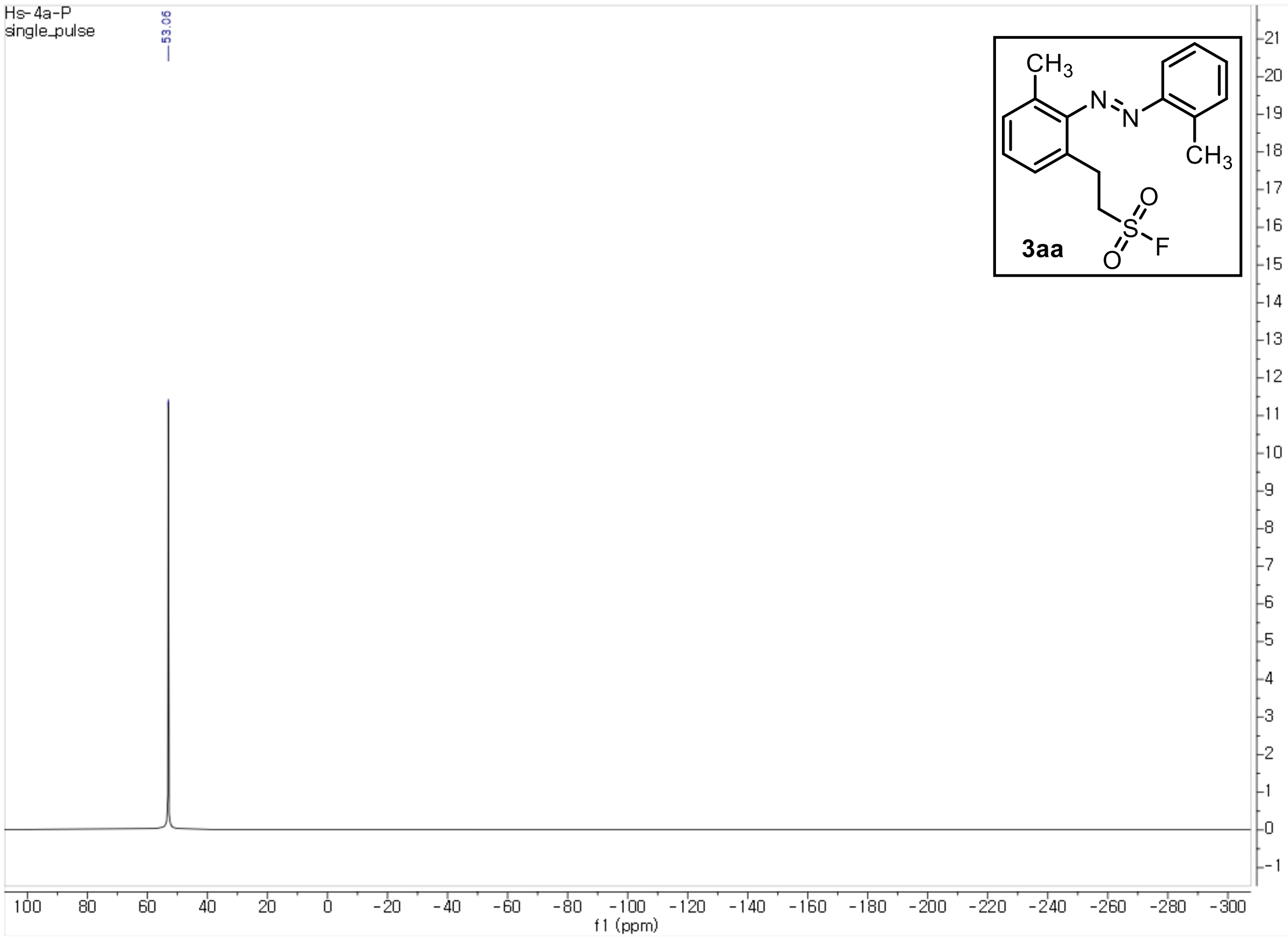
Hs-4a-P
single_pulse
53.05
3aa
f1 (ppm)

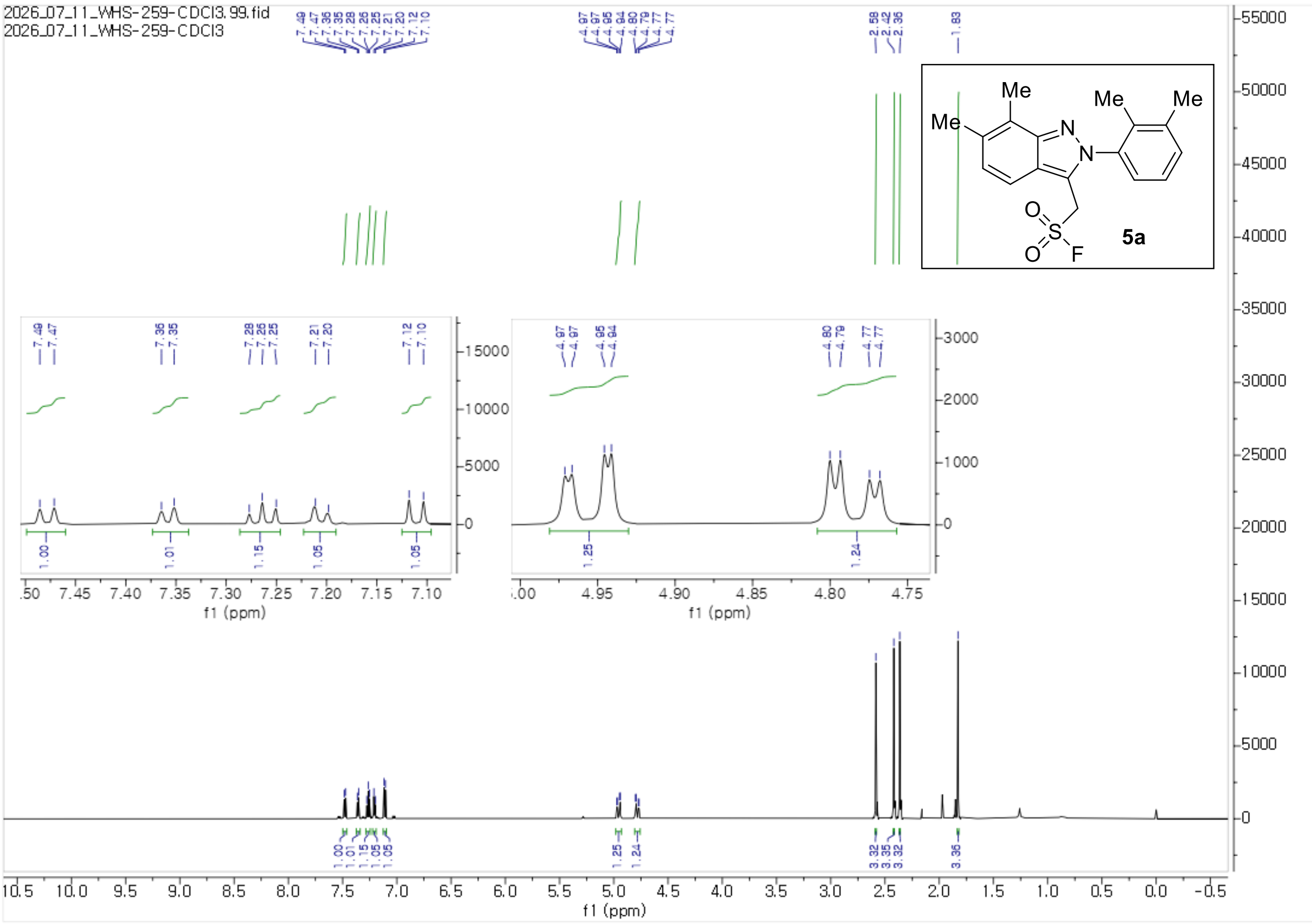
2026_07_11_WHS-259-CDCl3.99.fid
2026_07_11_WHS-259-CDCl3
7.49 7.47 7.36 7.35 7.28 7.26 7.25 7.21 7.20 7.12 7.10
4.97 4.97 4.95 4.94 4.80 4.79 4.77 4.77
2.59 2.42 2.36
1.83
Me
Me
Me
Me
N
N
O
S
O
F
5a
1.00 1.01 1.15 1.05 1.05
1.25 1.24
3.32 3.35 3.32
3.36
f1 (ppm)

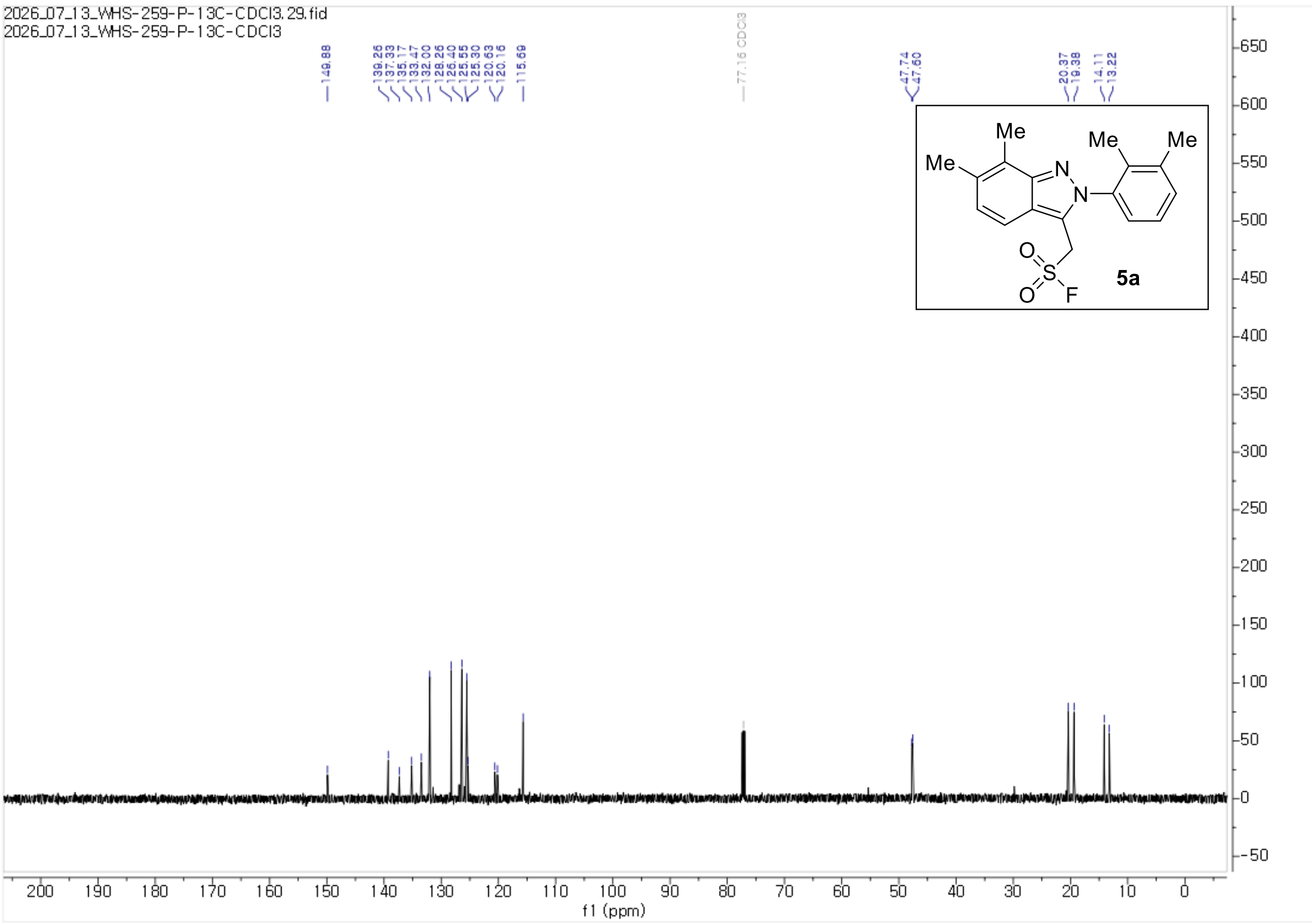
2026_07_13_WHS-259-P-13C-CDCl3.29.fid
2026_07_13_WHS-259-P-13C-CDCl3
149.88
139.26 137.33 135.17 133.47 132.00 128.26 126.40 125.55 125.30 120.63 120.16 115.69
77.16 CDCl3
47.74 47.60
20.37 19.38 14.11 13.22
Me
Me
Me
Me
N
N
O
S
O
F
5a
f1 (ppm)

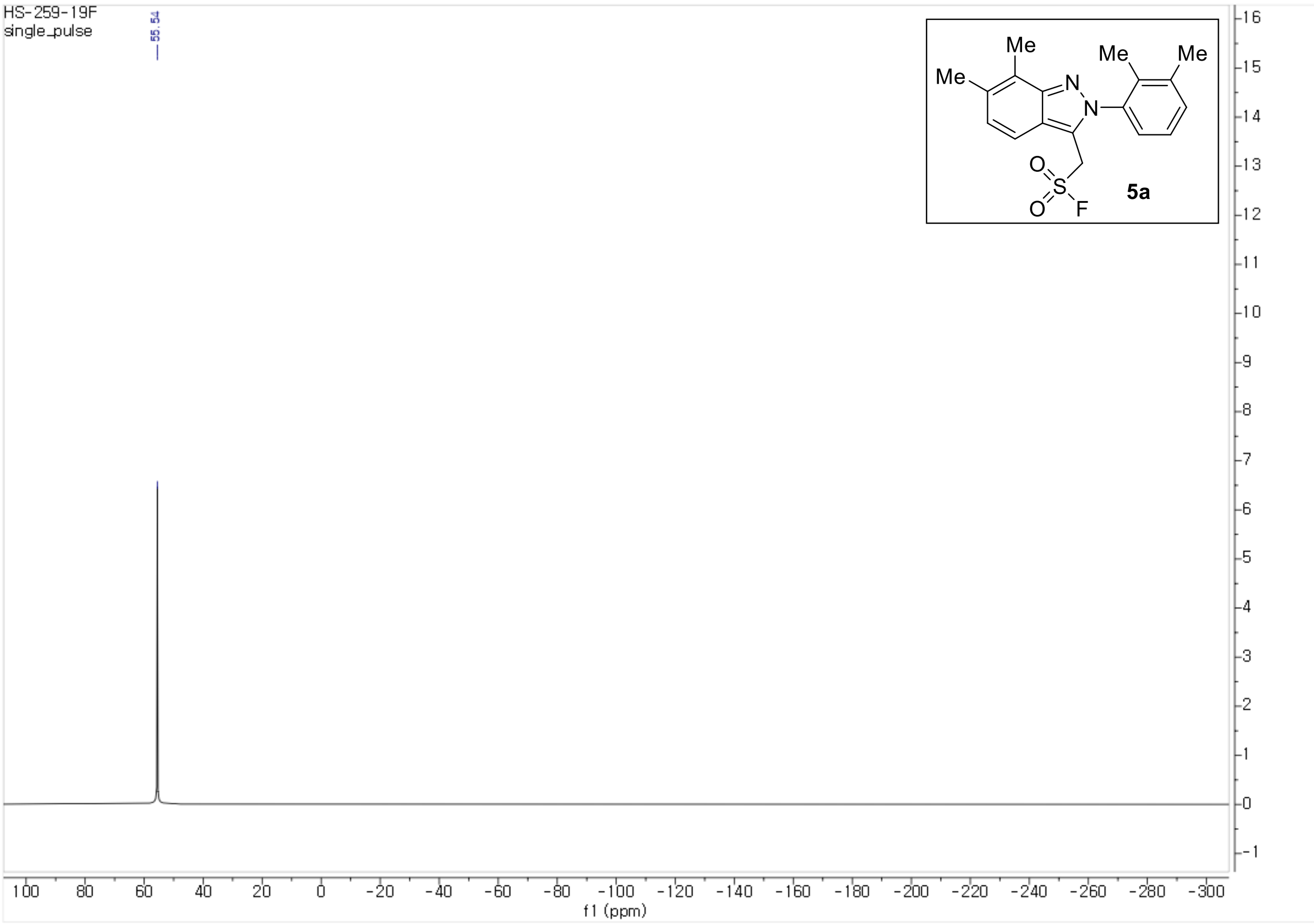

HS-259-19F
single_pulse
55.54
Me
Me
Me
Me
N
N
O
S
O
F
5a
f1 (ppm)

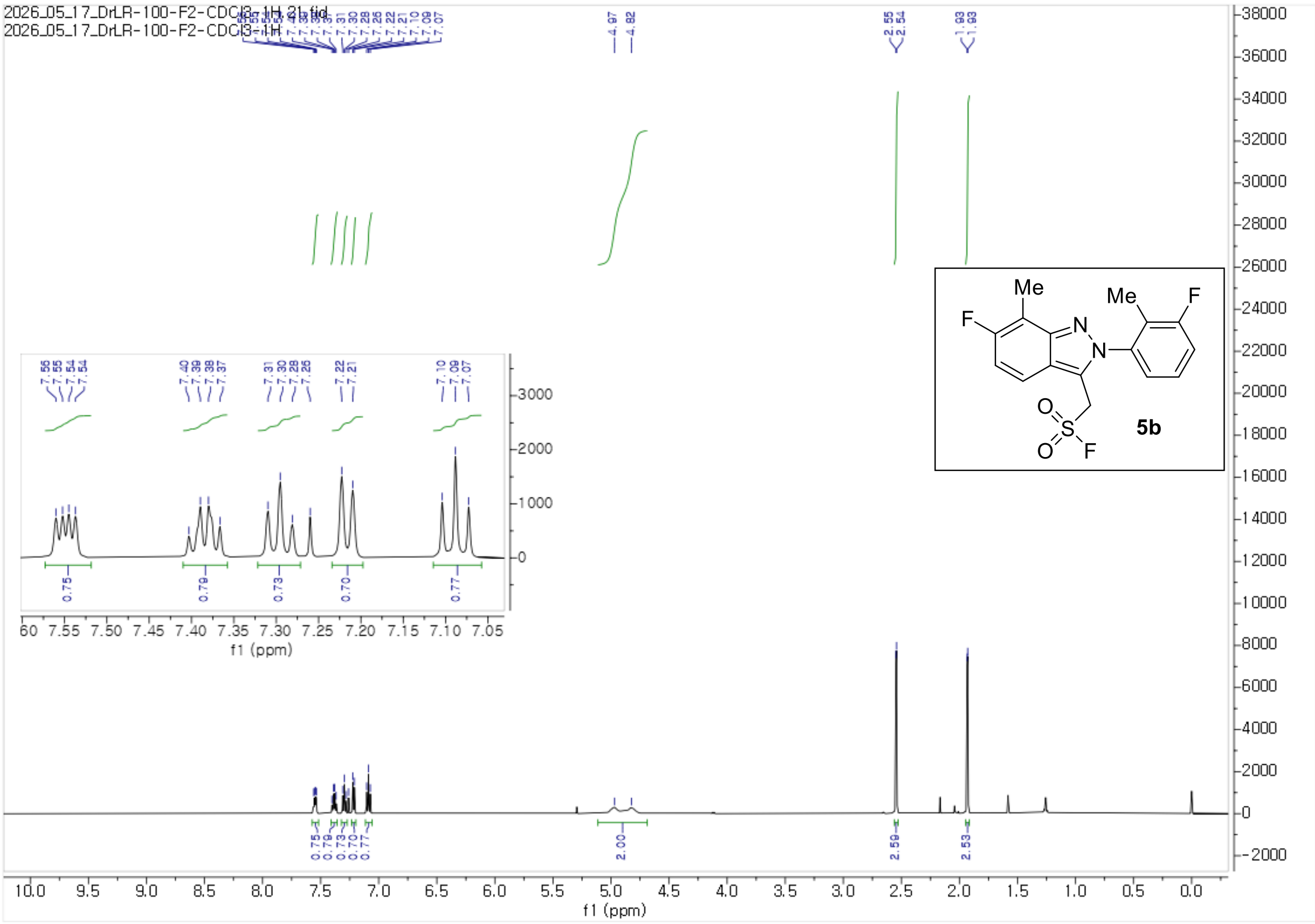
2026_05_17_DrLR-100-F2-CDCl3-1H.21.fid
2026_05_17_DrLR-100-F2-CDCl3-1H
Me
Me
F
F
N
N
O
S
O
F
5b
f1 (ppm)

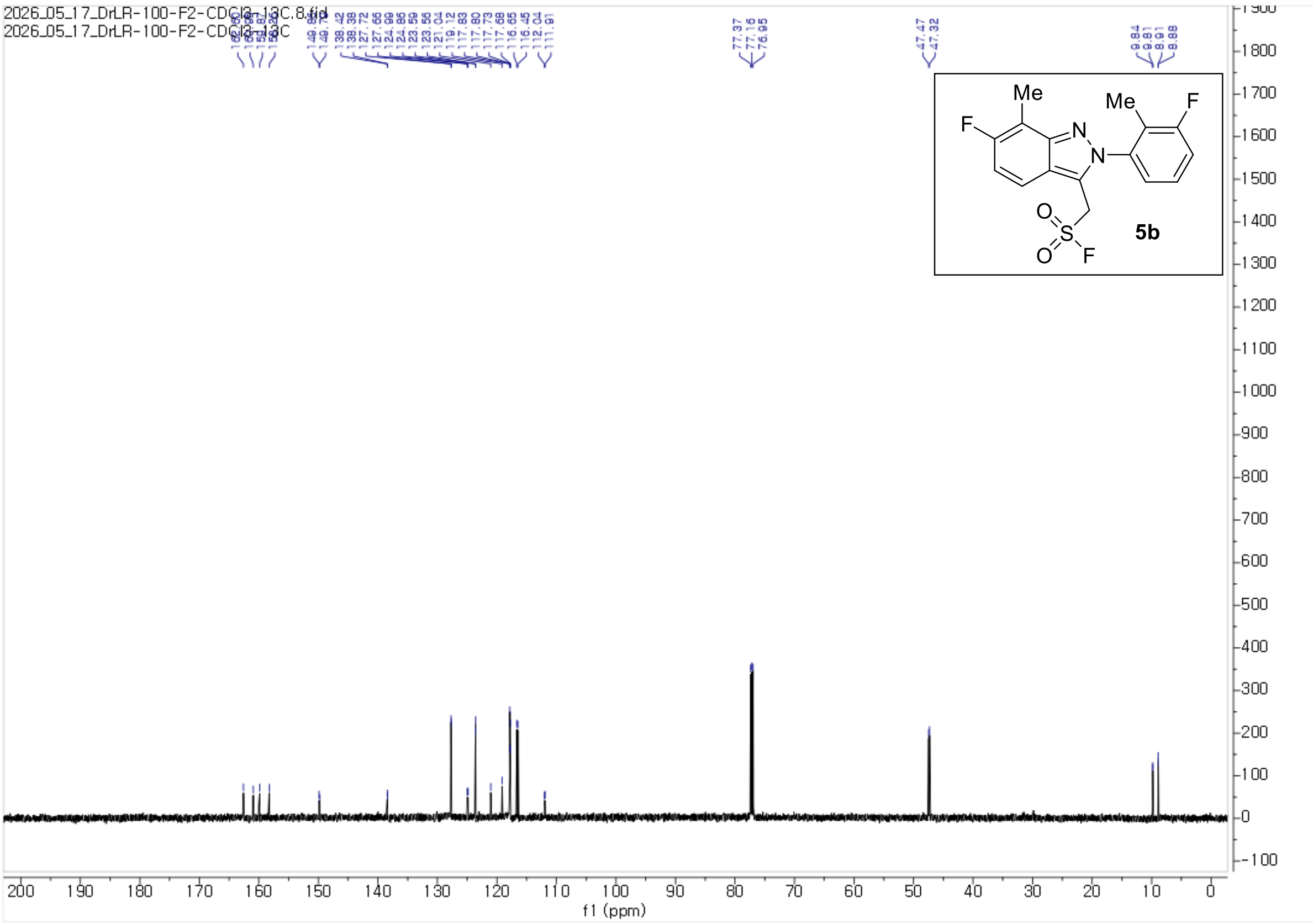
2026_05_17_DrLR-100-F2-CDCl3-13C.8.fid
2026_05_17_DrLR-100-F2-CDCl3-13C
Me
Me
F
F
N
N
O
S
O
F
5b
f1 (ppm)

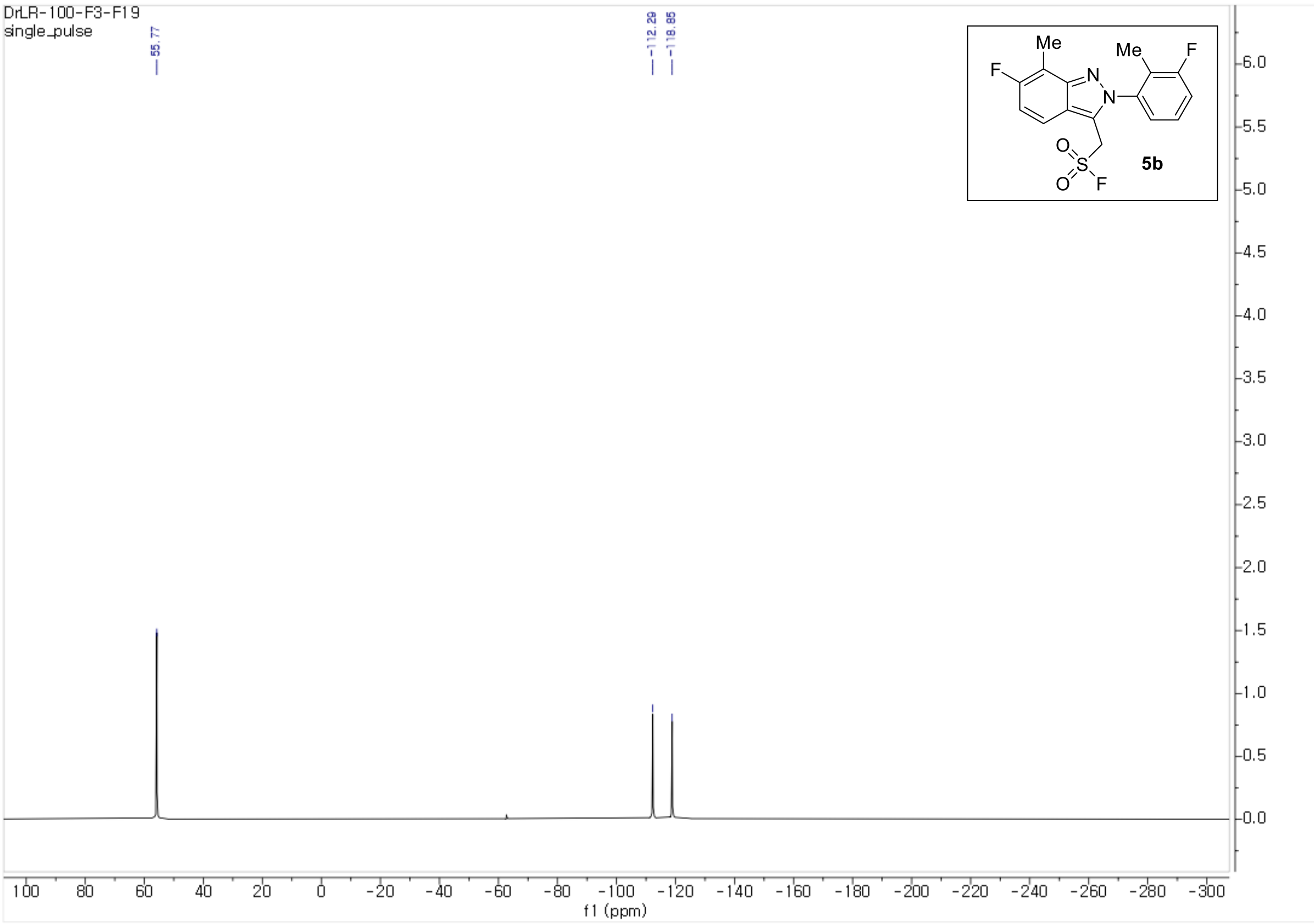

DrLR-100-F3-F19
single_pulse
55.77
-112.29
-118.85
Me
F
N
N
Me
F
O
S
O
F
5b
f1 (ppm)

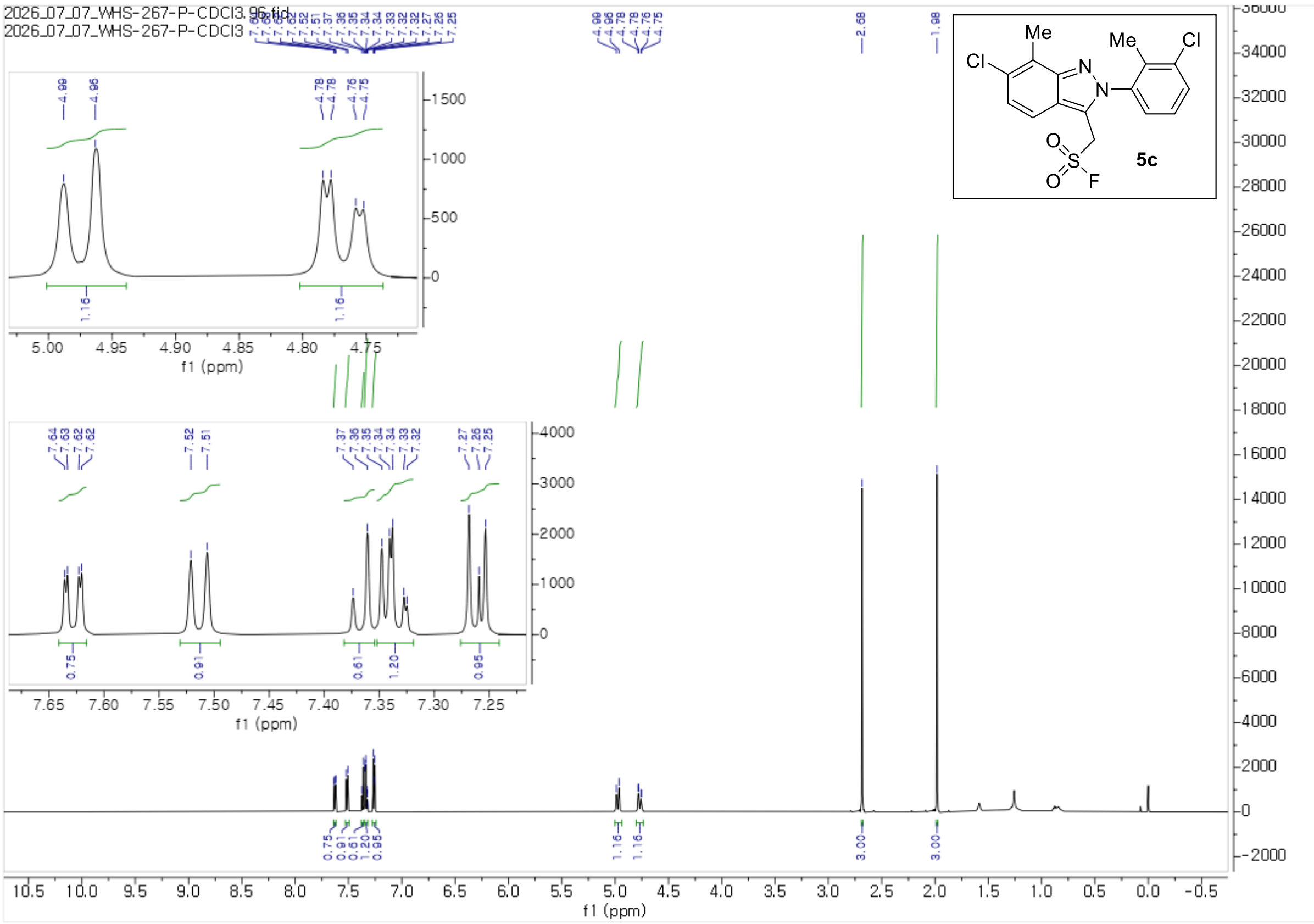
2026_07_07_WHS-267-P-CDCl3.96.fid
2026_07_07_WHS-267-P-CDCl3
Me
Cl
N
N
Me
Cl
O
S
O
F
5c
f1 (ppm)

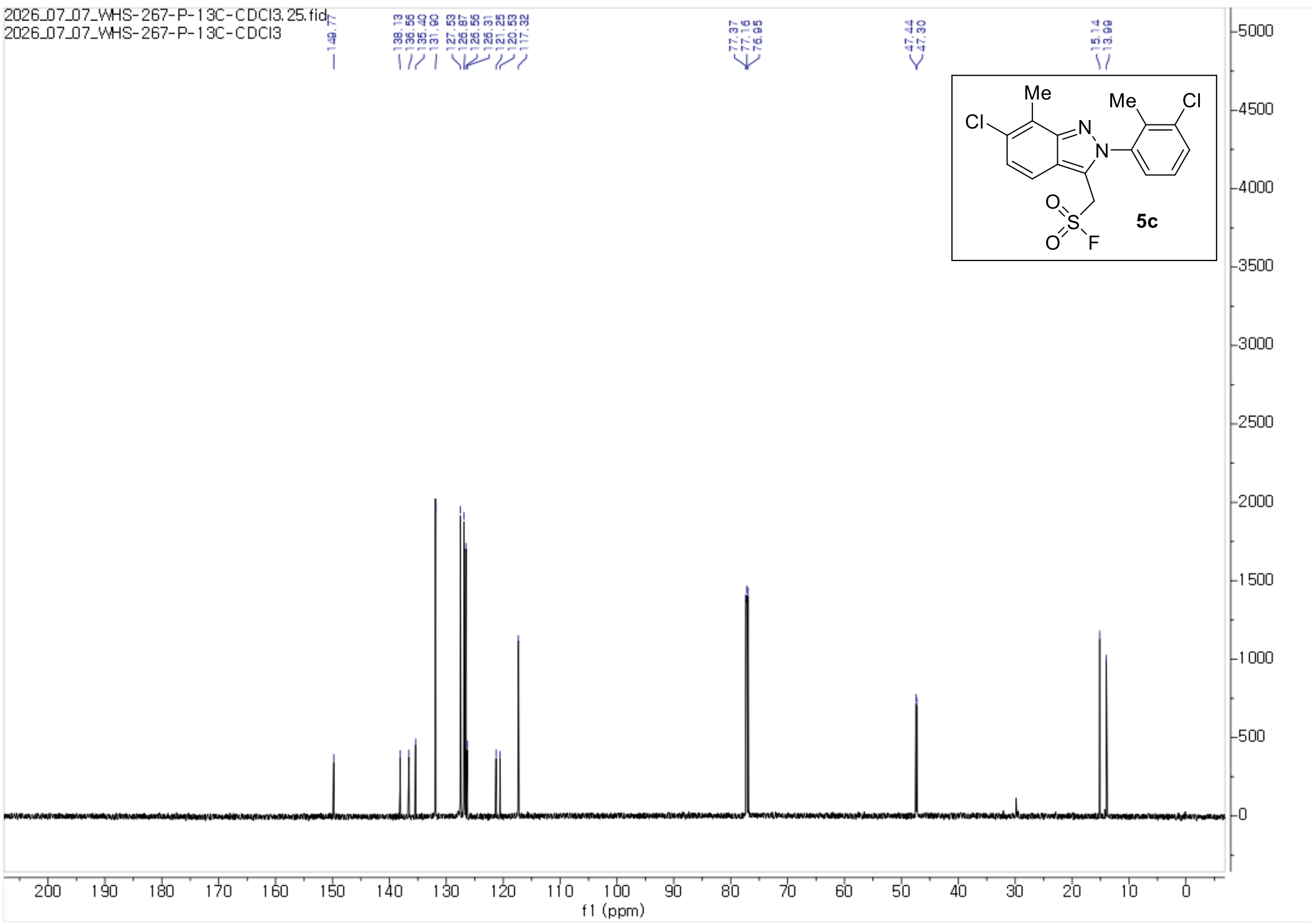
2026_07_07_WHS-267-P-13C-CDCl3.25.fid
2026_07_07_WHS-267-P-13C-CDCl3
149.77
138.13
136.56
135.40
131.90
127.53
126.87
126.56
126.31
121.25
120.53
117.32
77.37
77.16
76.95
47.44
47.30
15.14
13.99
Me
Cl
N
N
Me
Cl
O
S
O
F
5c
f1 (ppm)

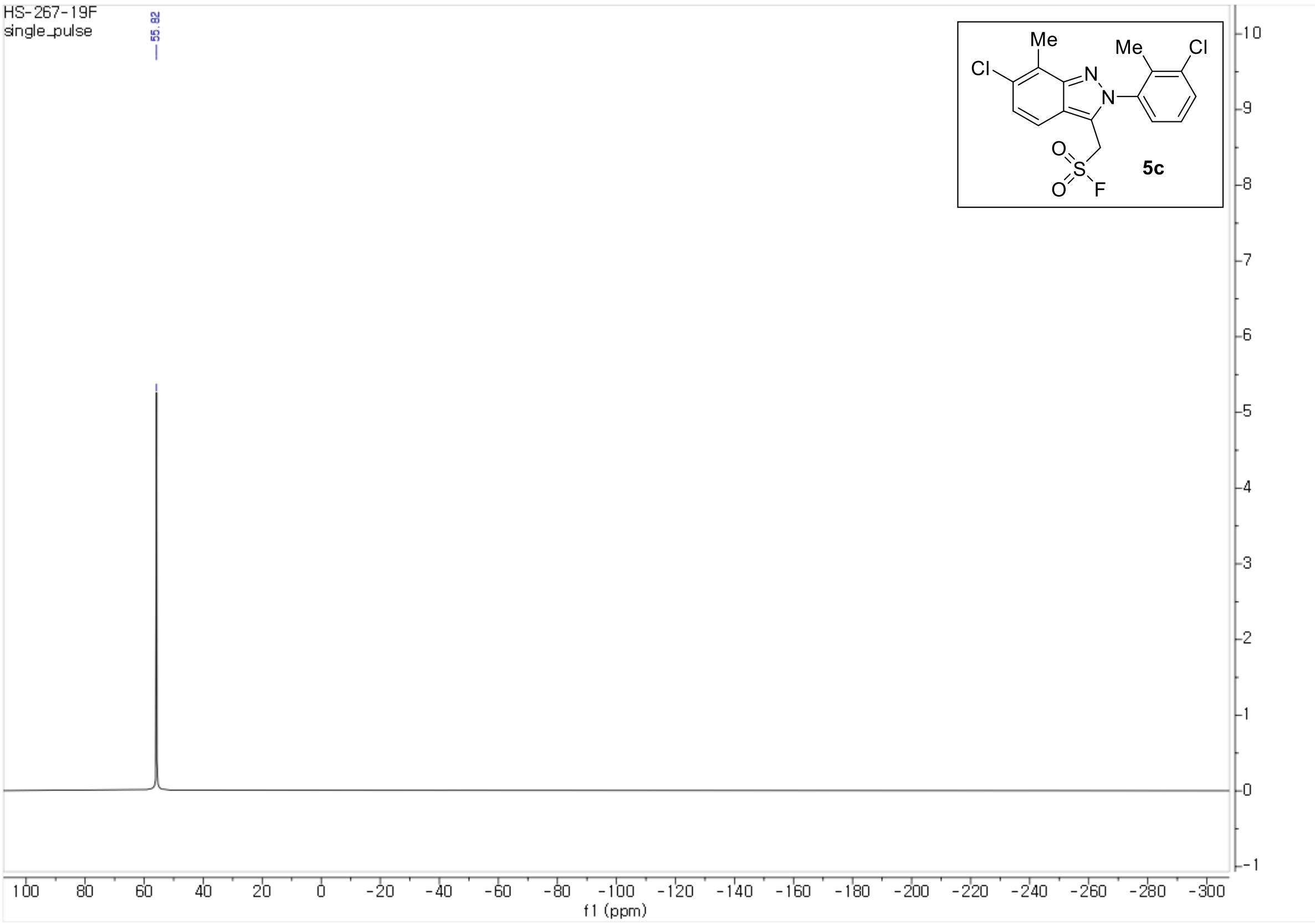
HS-267-19F
single_pulse
55.82
Me
Cl
N
N
Me
Cl
O
S
O
F
5c
f1 (ppm)

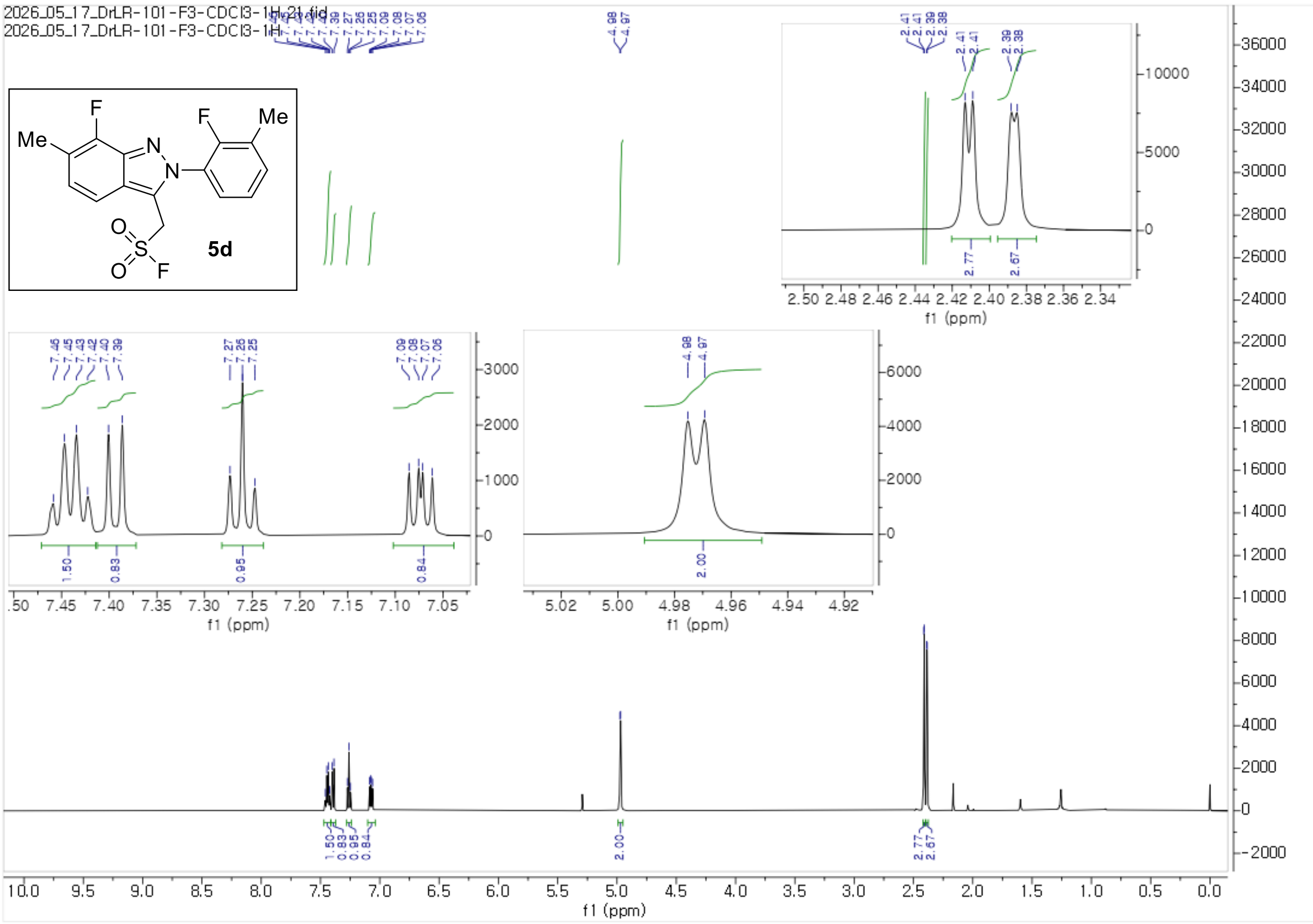
2026_05_17_DrLR-101-F3-CDCl3-1H.21.fid
2026_05_17_DrLR-101-F3-CDCl3-1H
F
Me
F
Me
N
N
O
S
O
F
5d
7.45
7.45
7.43
7.42
7.40
7.39
7.27
7.25
7.25
7.09
7.08
7.07
7.06
4.98
4.97
2.41
2.41
2.39
2.38
1.50
0.83
0.95
0.84
2.00
2.77
2.67
f1 (ppm)

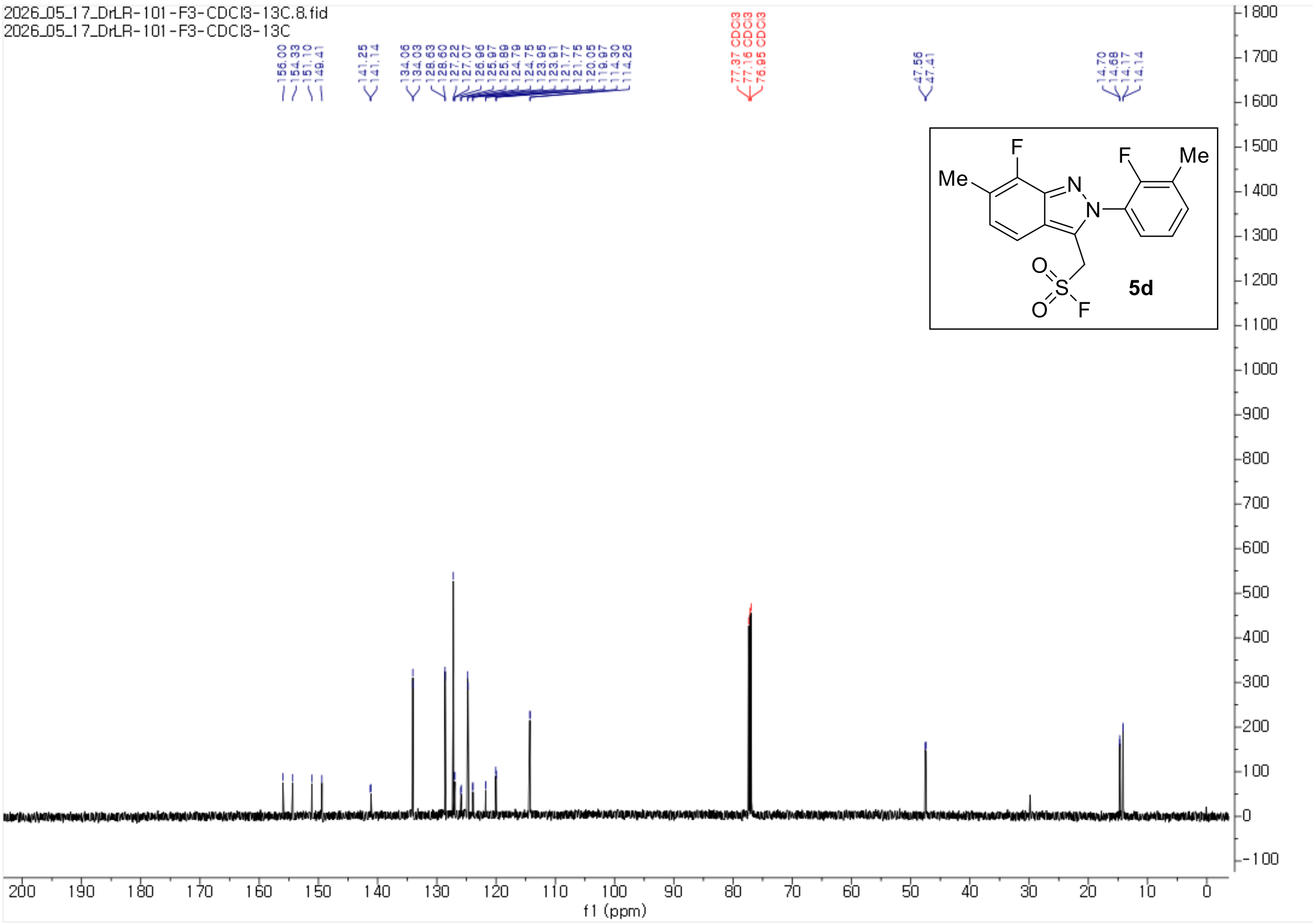
2026_05_17_DrLR-101-F3-CDCl3-13C.8.fid
2026_05_17_DrLR-101-F3-CDCl3-13C
155.00
154.33
151.10
149.41
141.25
141.14
134.06
134.03
128.63
128.60
127.22
127.07
126.96
125.97
125.89
124.79
124.75
123.95
123.91
121.75
120.05
119.97
114.30
114.26
77.37 CDCl3
77.16 CDCl3
76.95 CDCl3
47.56
47.41
14.70
14.67
14.17
14.14
F
Me
F
Me
N
N
O
S
O
F
5d
f1 (ppm)

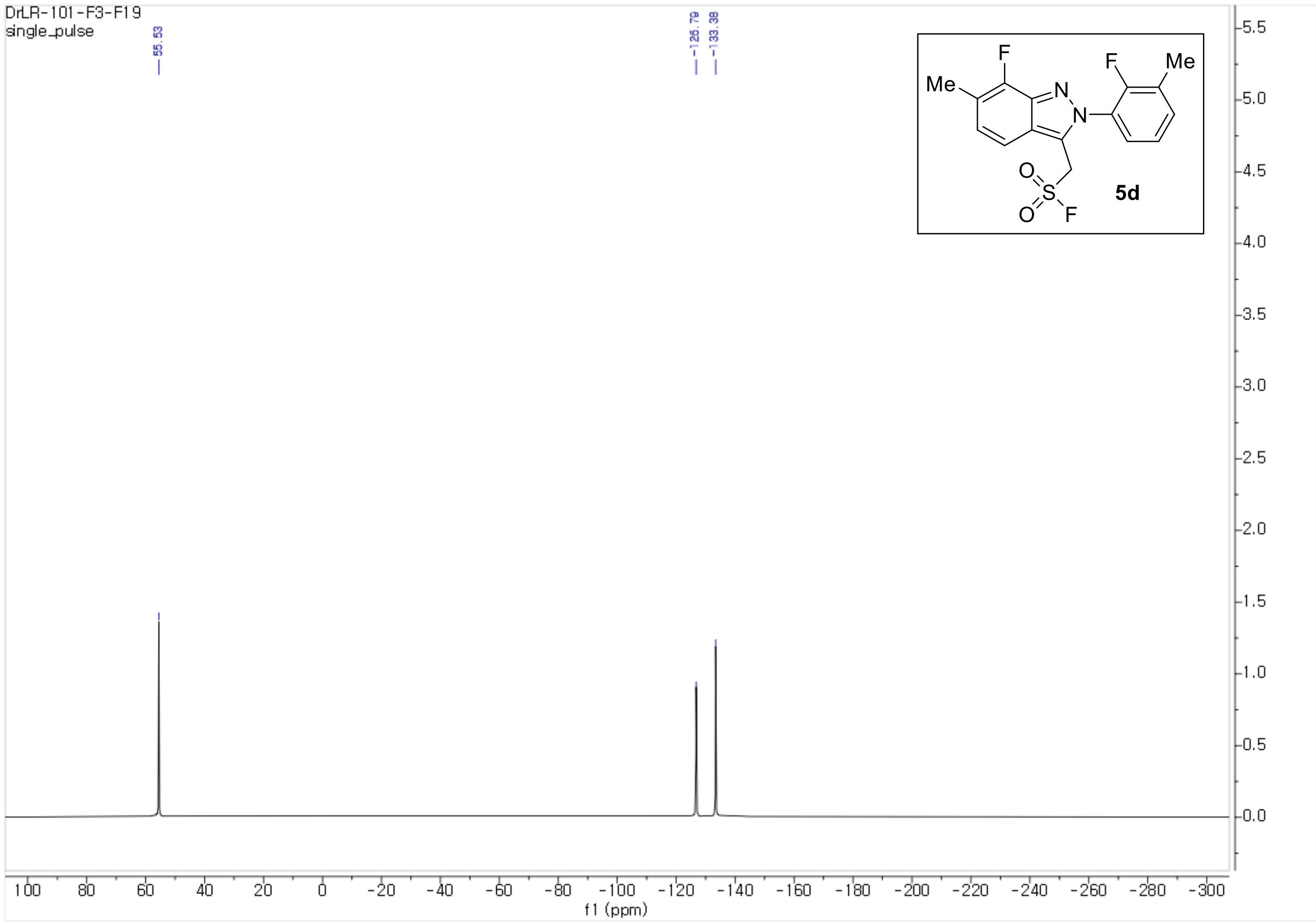
DrLR-101-F3-F19
single_pulse
55.53
-126.79
-133.38
F
Me
N
N
F
Me
O
S
O
F
5d
f1 (ppm)

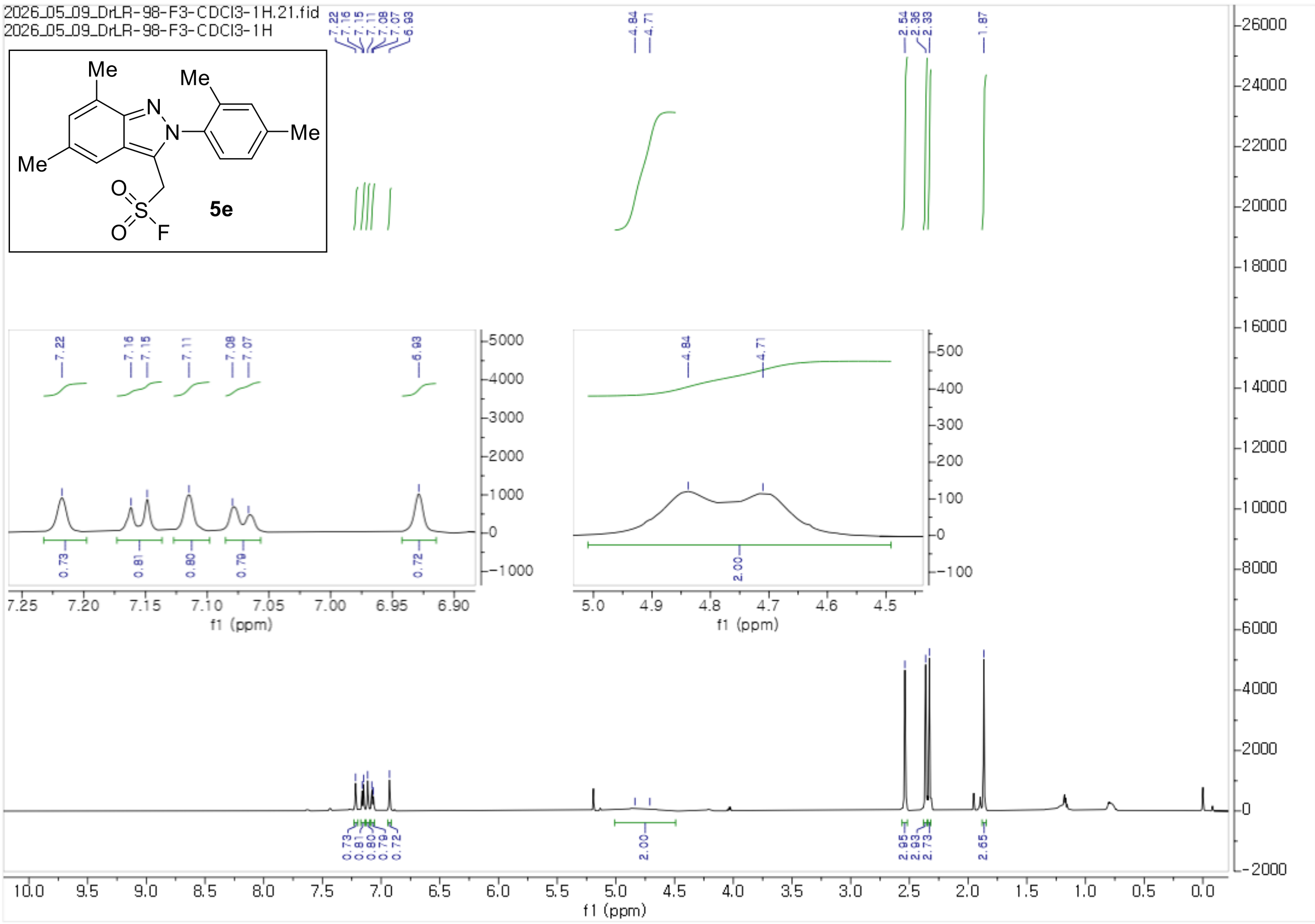
2026_05_09_DrLR-98-F3-CDCl3-1H.21.fid
2026_05_09_DrLR-98-F3-CDCl3-1H
7.22 7.16 7.15 7.11 7.08 7.07 6.93
4.84 4.71
2.54 2.36 2.33
1.87
Me
Me
Me
Me
N
N
O
S
O
F
5e
f1 (ppm)

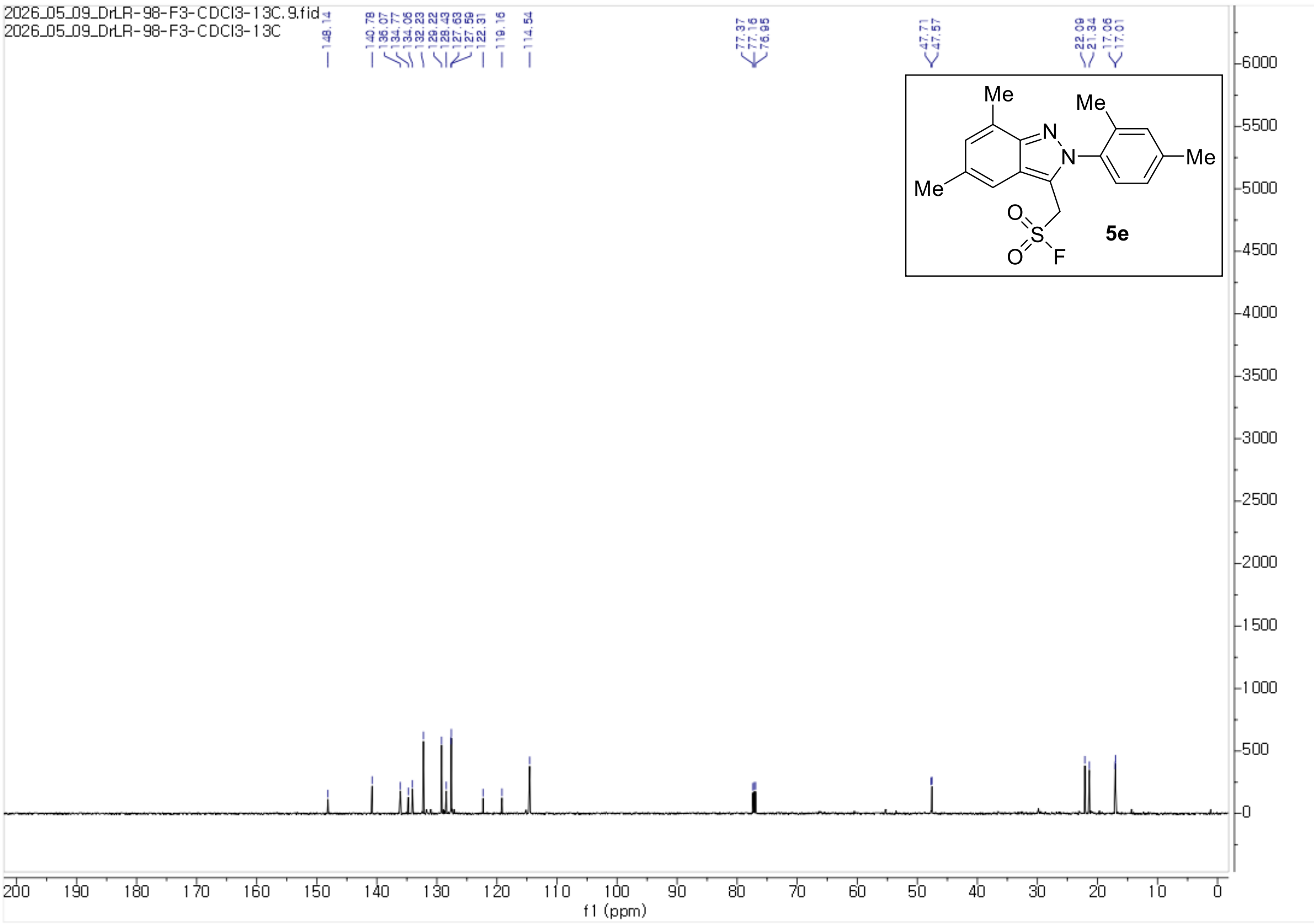
2026_05_09_DrLR-98-F3-CDCl3-13C.9.fid
2026_05_09_DrLR-98-F3-CDCl3-13C
148.14
140.78 136.07 134.77 134.06 132.23 129.22 128.43 127.63 127.59 122.31 119.16
114.54
77.37 77.16 76.95
47.71 47.57
22.09 21.34 17.06 17.01
Me
Me
Me
Me
N
N
O
S
O
F
5e
f1 (ppm)

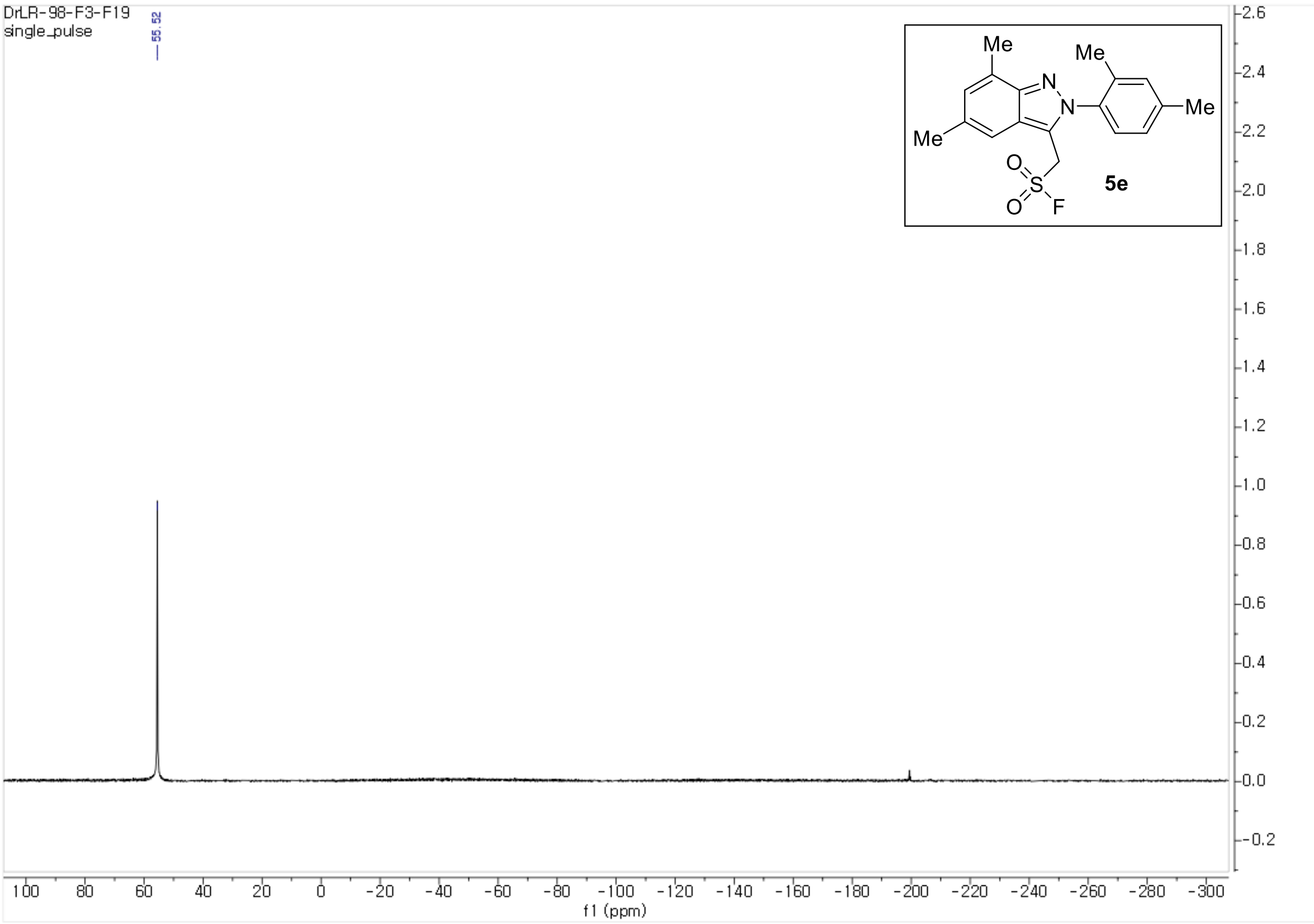

DrLR-98-F3-F19
single_pulse
55.52
5e
f1 (ppm)

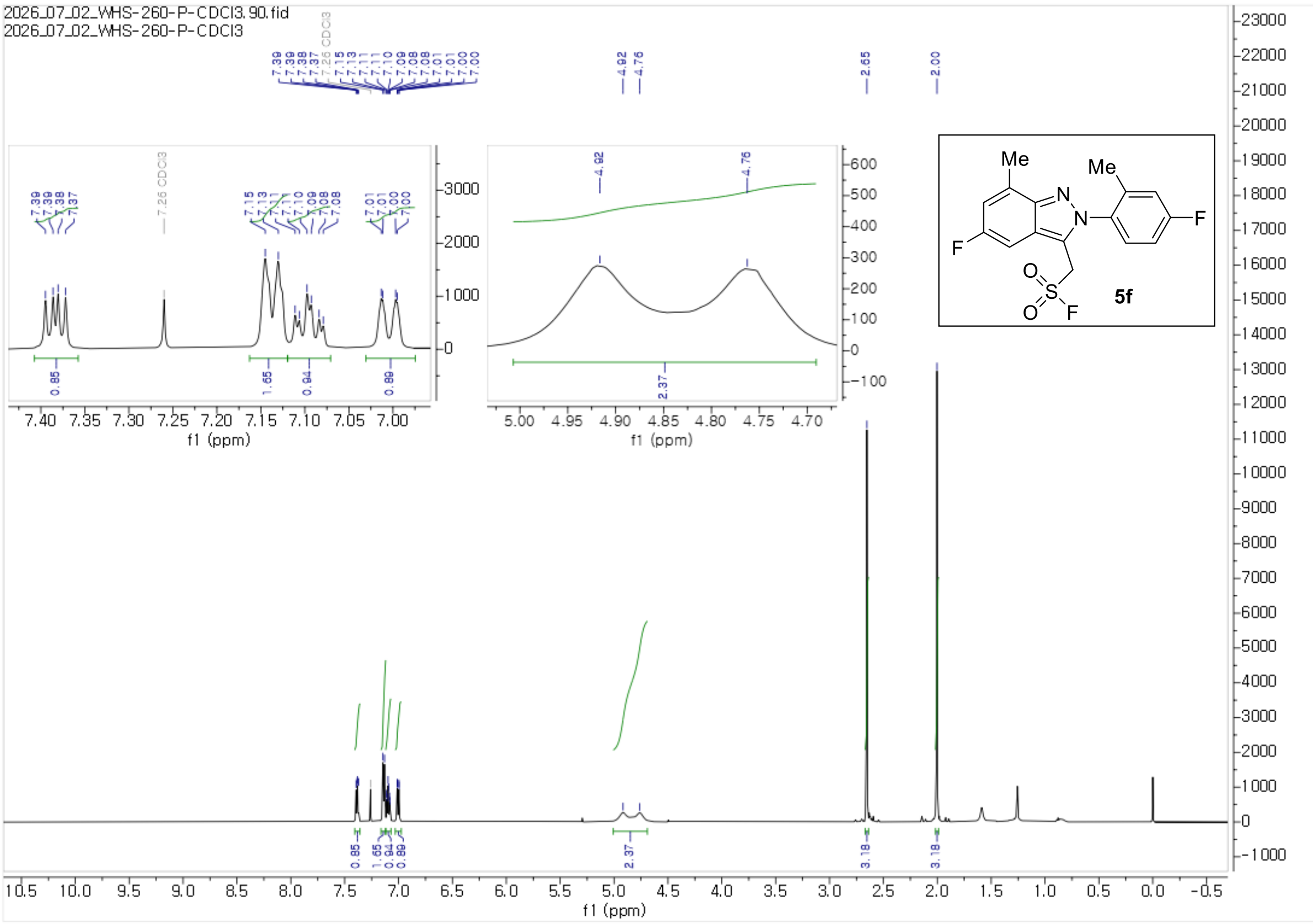
2026_07_02_WHS-260-P-CDCl3.90.fid
2026_07_02_WHS-260-P-CDCl3
7.39 7.39 7.38 7.37 7.26 CDCl3 7.15 7.13 7.11 7.11 7.10 7.09 7.08 7.08 7.01 7.01 7.00 7.00
4.92 4.76
2.65
2.00
0.85 1.65 0.94 0.89
2.37
3.18 3.18
Me
Me
N
N
F
F
O
S
O
F
5f
f1 (ppm)

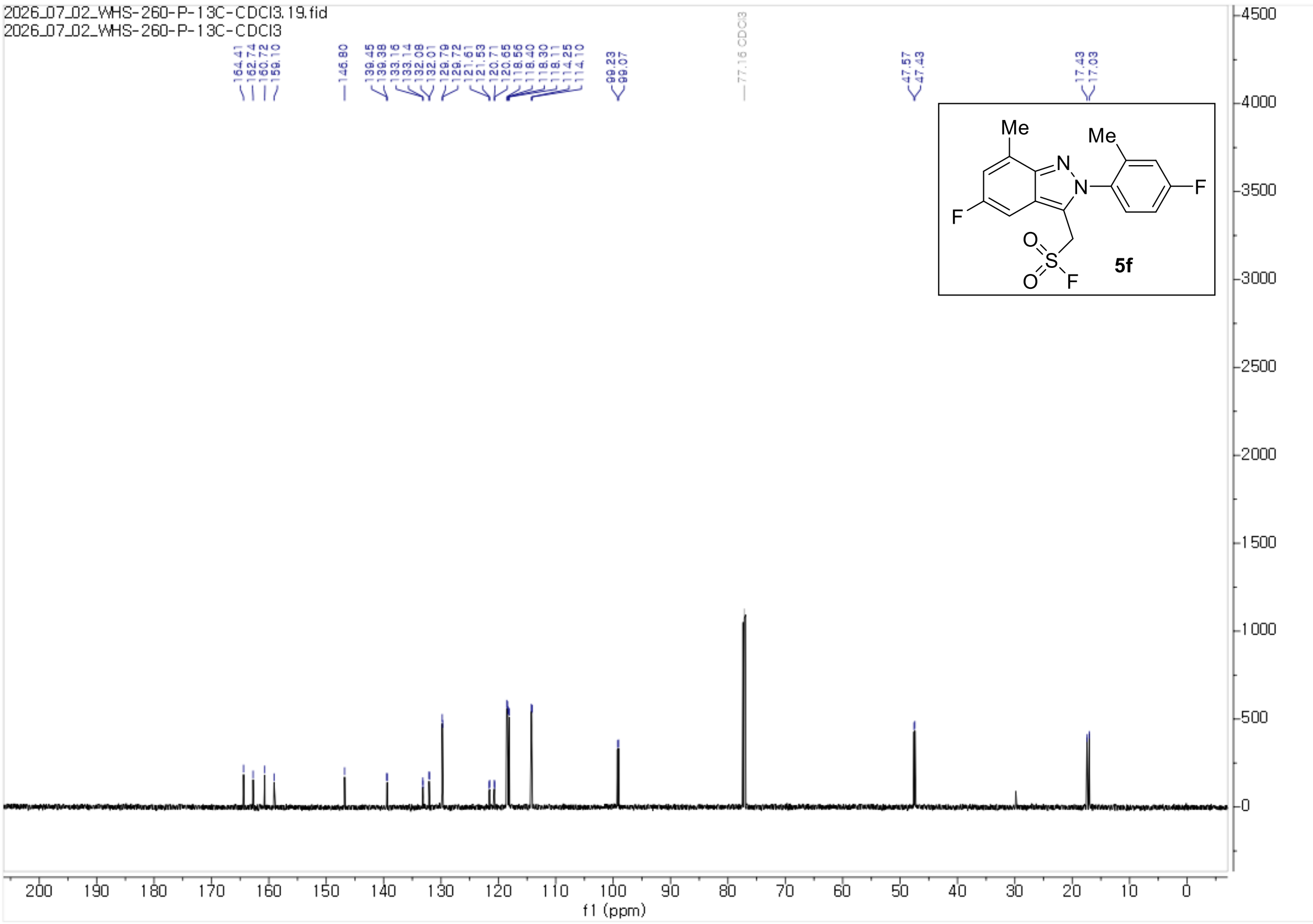
2026_07_02_WHS-260-P-13C-CDCl3.19.fid
2026_07_02_WHS-260-P-13C-CDCl3
164.41 162.74 160.72 159.10
146.80
139.45 139.38 133.16 133.14 132.08 132.01 129.79 129.72 121.81 121.53 120.71 120.65 118.55 118.40 118.30 118.11 114.25 114.10
99.23 99.07
77.16 CDCl3
47.57 47.43
17.43 17.03
Me
Me
N
N
F
F
O
S
O
F
5f
f1 (ppm)

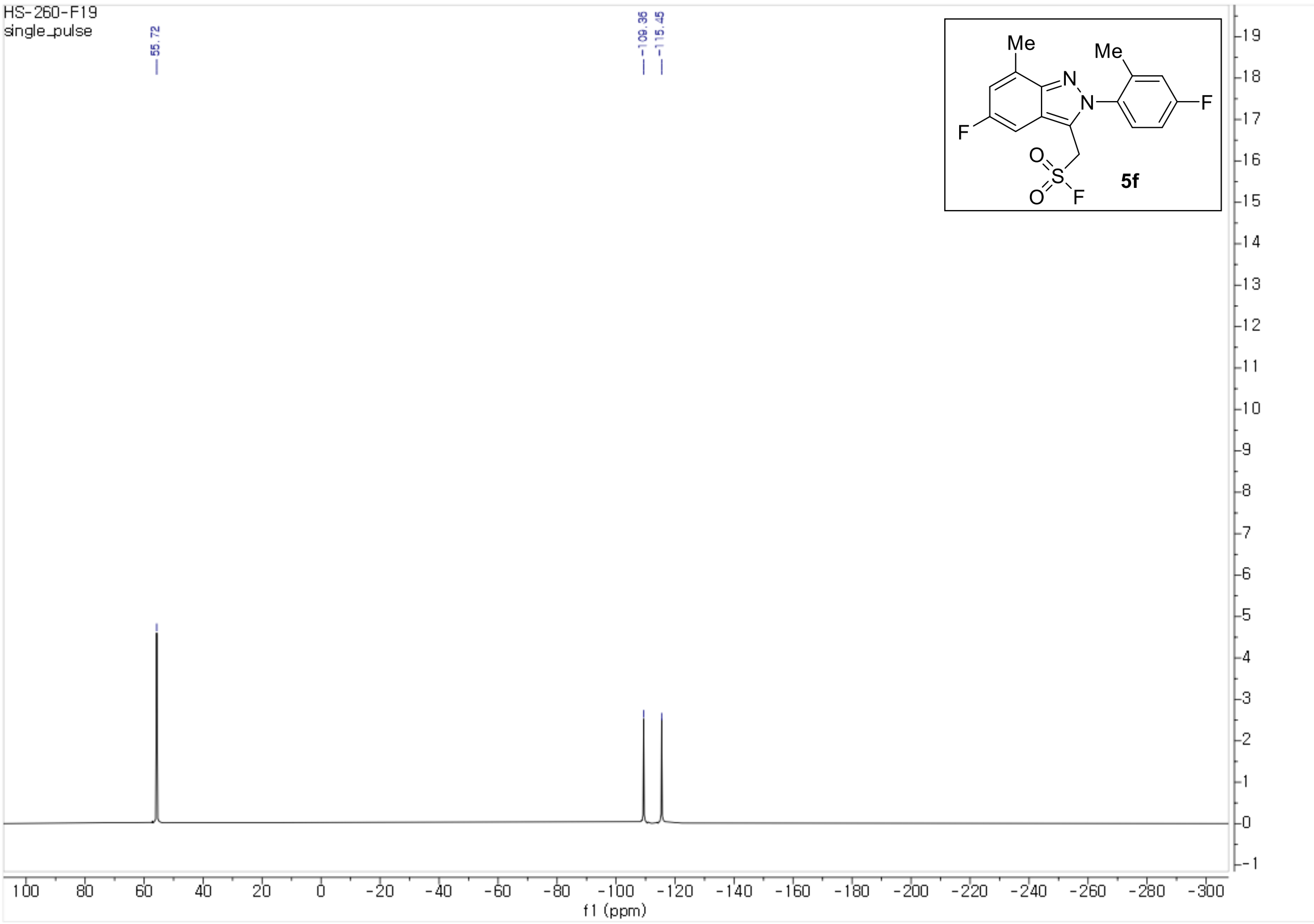
HS-260-F19
single_pulse
55.72
-109.36
-115.45
Me
Me
N
N
F
F
O
S
O
F
5f
f1 (ppm)

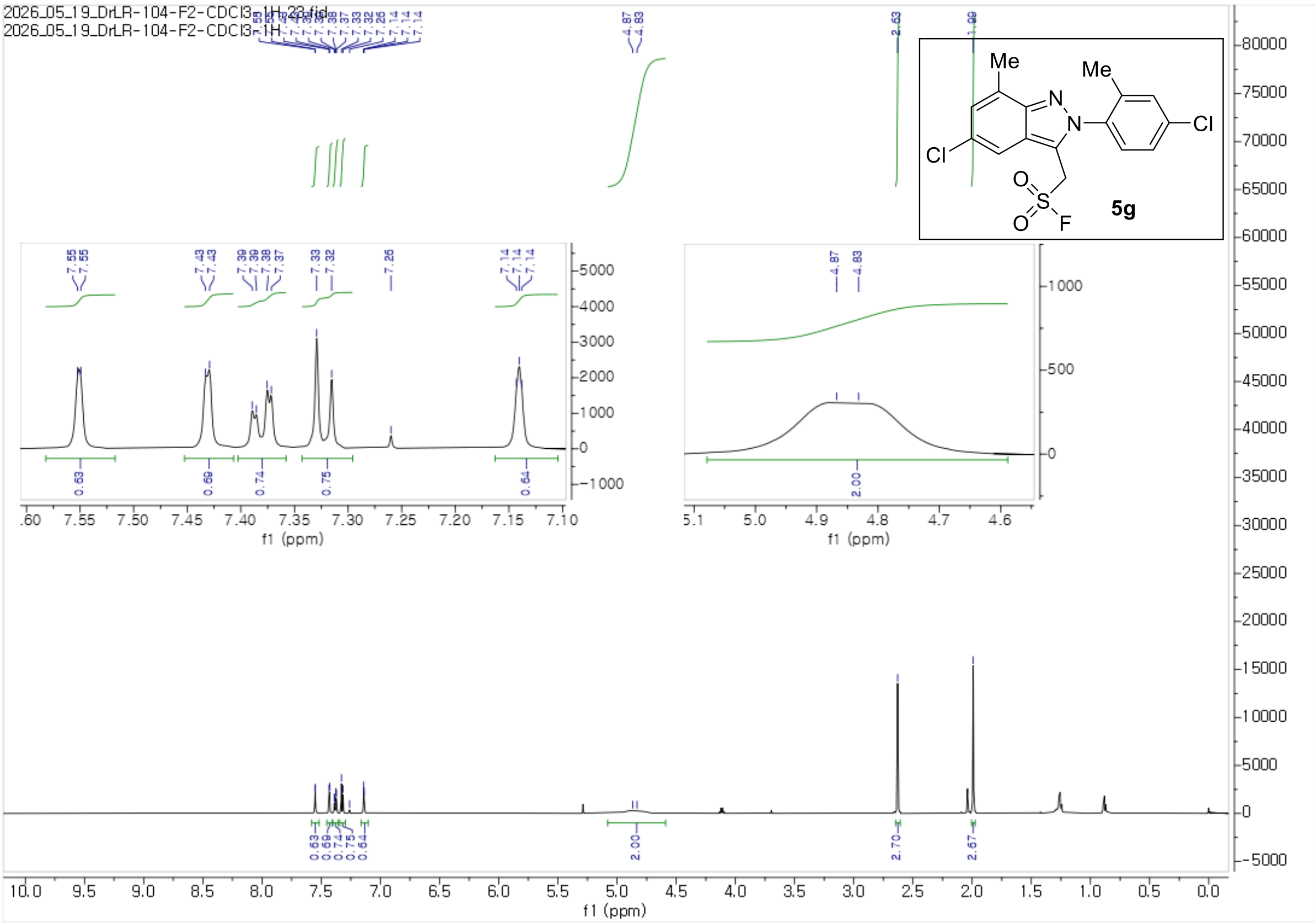
2026_05_19_DrLR-104-F2-CDCl3-1H.23.fid
2026_05_19_DrLR-104-F2-CDCl3-1H
5g
f1 (ppm)

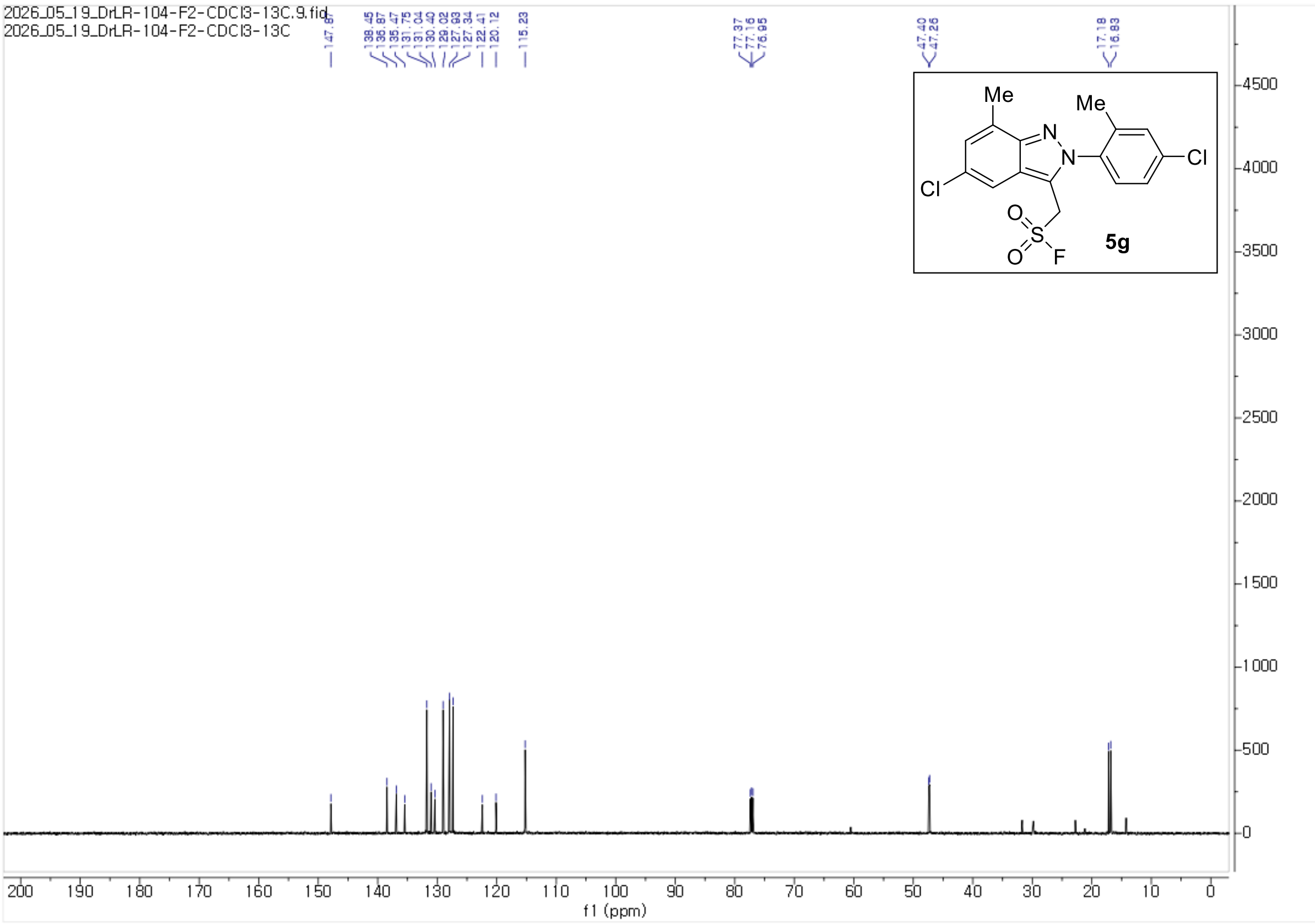
2026_05_19_DrLR-104-F2-CDCl3-13C.9.fid
2026_05_19_DrLR-104-F2-CDCl3-13C
147.87
115.23
77.37
77.16
76.95
47.40
47.26
17.18
16.83
5g
f1 (ppm)

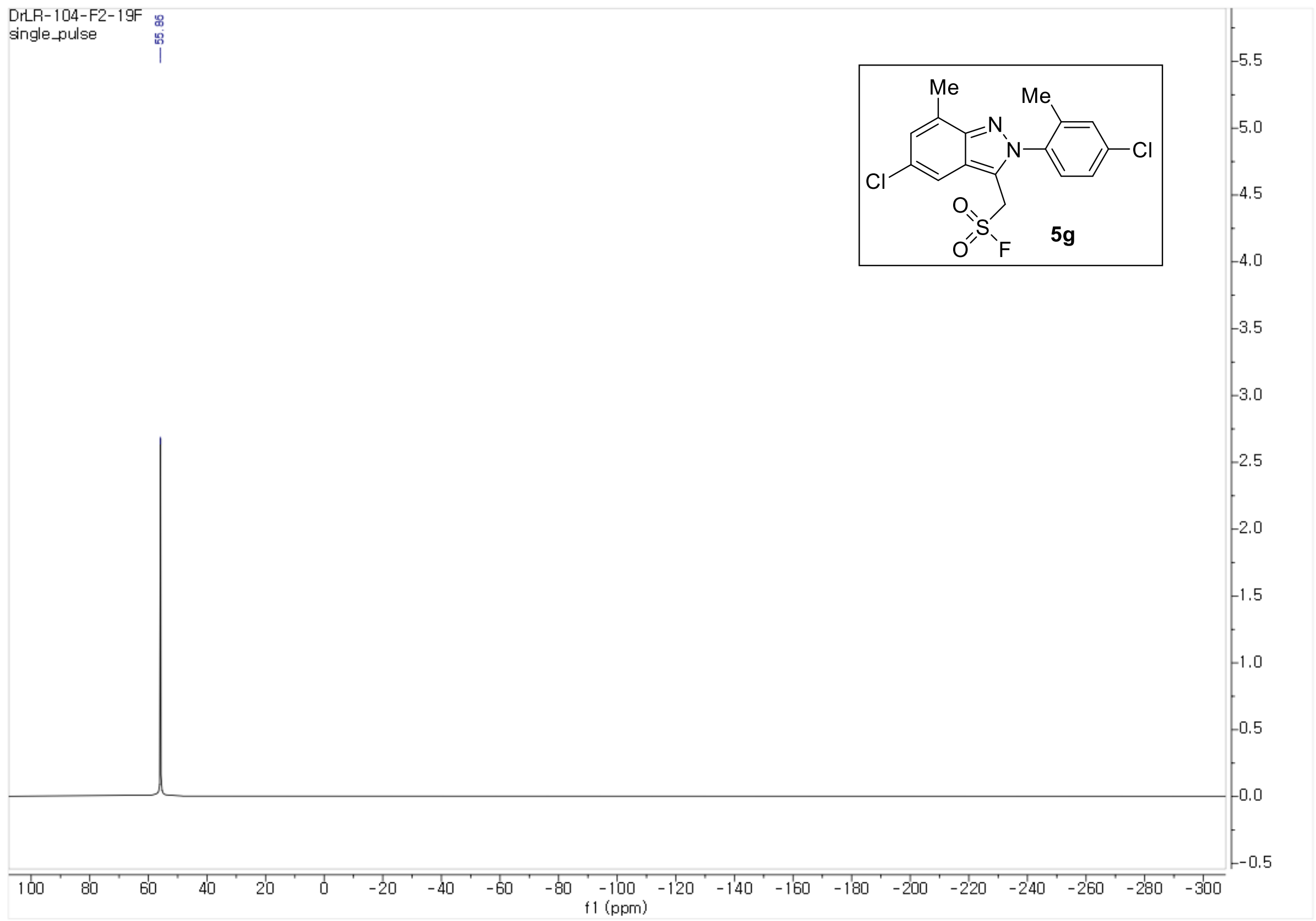

DrLR-104-F2-19F
single_pulse
55.86
Me
Me
N
N
Cl
Cl
O
S
O
F
5g
f1 (ppm)

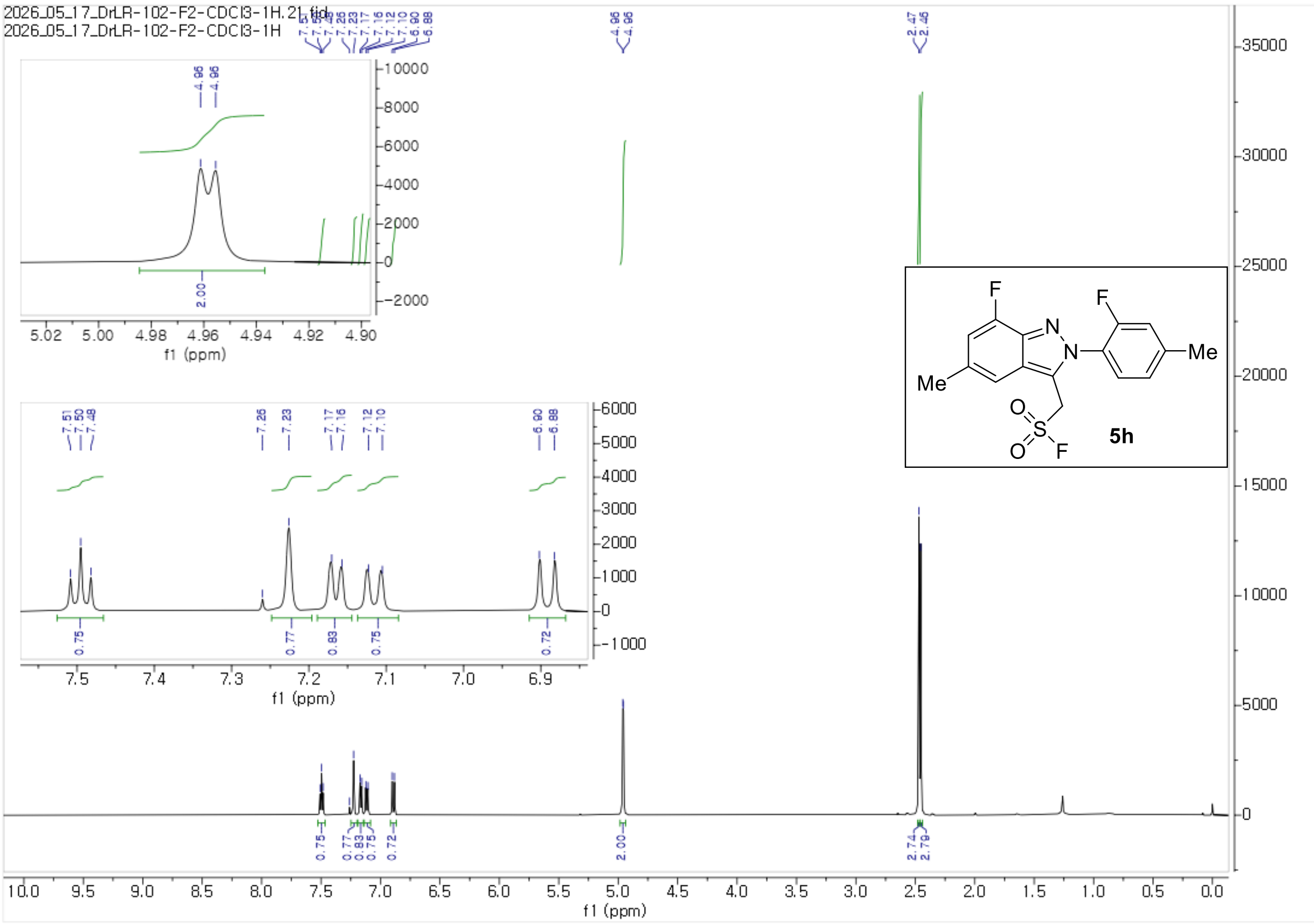
2026_05_17_DrLR-102-F2-CDCl3-1H.21.fid
2026_05_17_DrLR-102-F2-CDCl3-1H
5h
f1 (ppm)

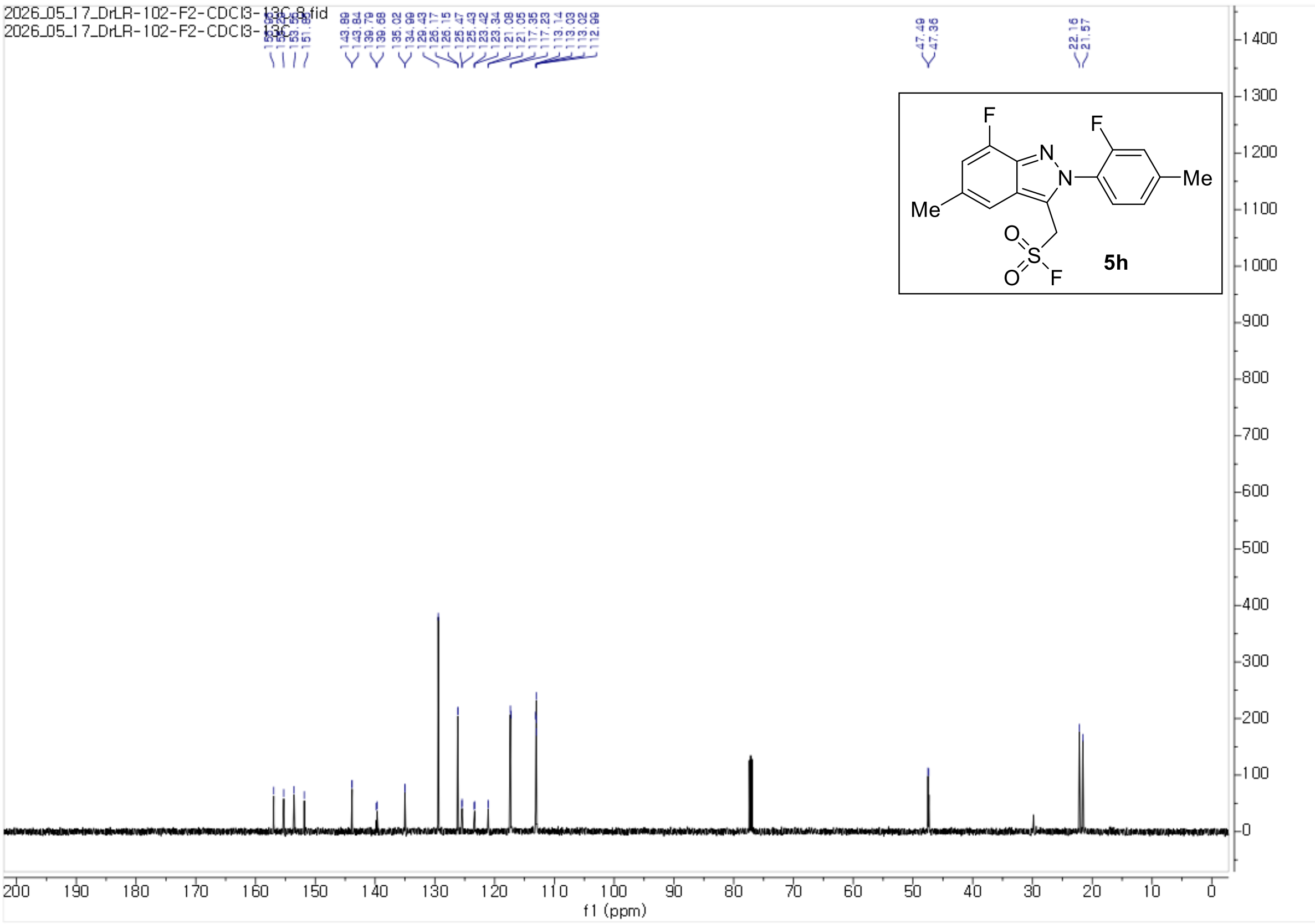
2026_05_17_DrLR-102-F2-CDCl3-13C.8.fid
2026_05_17_DrLR-102-F2-CDCl3-13C
5h
f1 (ppm)

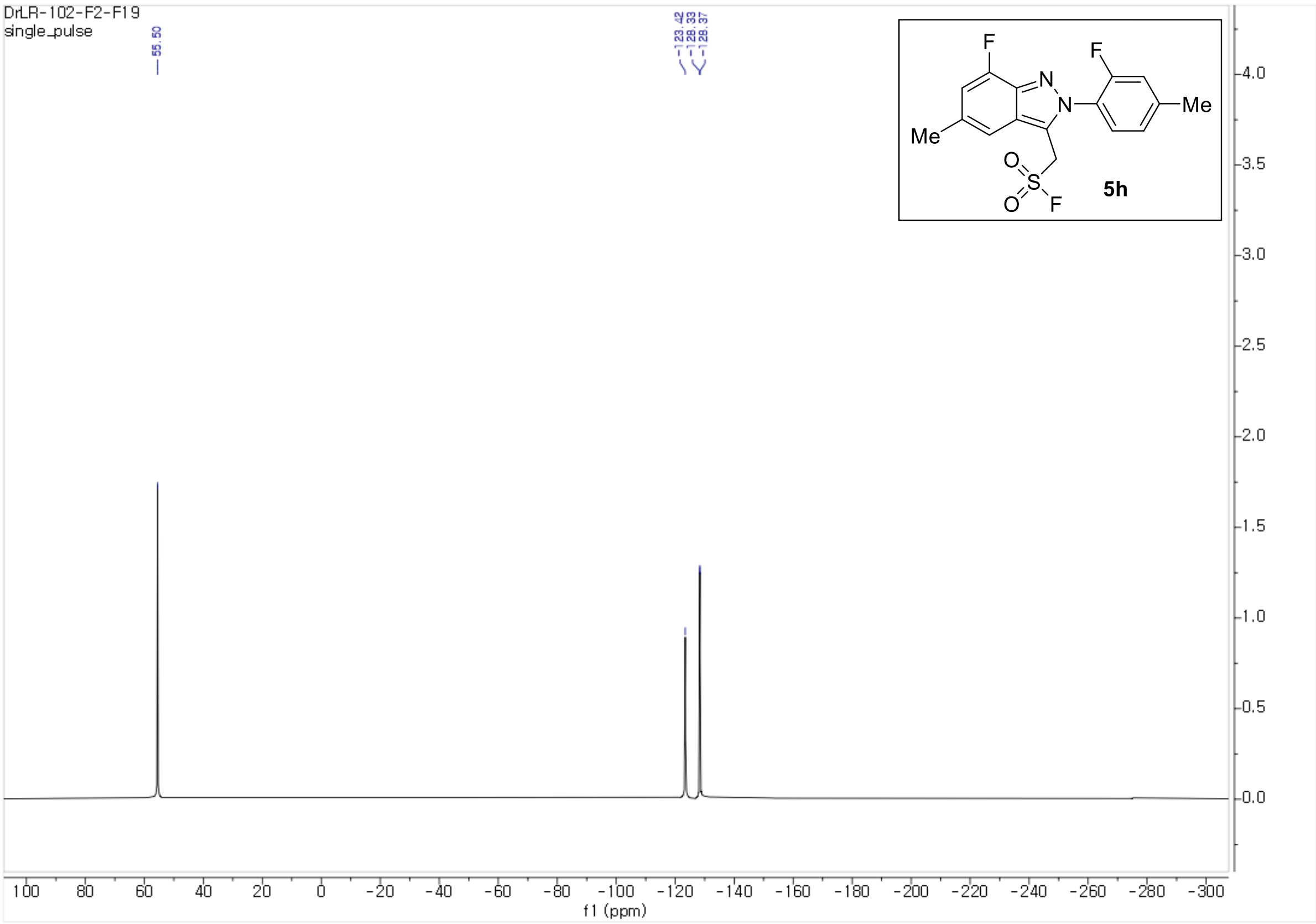
DrLR-102-F2-F19
single_pulse
55.50
-123.42
-128.33
-128.37
F
F
N
N
Me
Me
O
S
O
F
5h
4.0
3.5
3.0
2.5
2.0
1.5
1.0
0.5
0.0
100
80
60
40
20
0
-20
-40
-60
-80
-100
-120
-140
-160
-180
-200
-220
-240
-260
-280
-300
f1 (ppm)

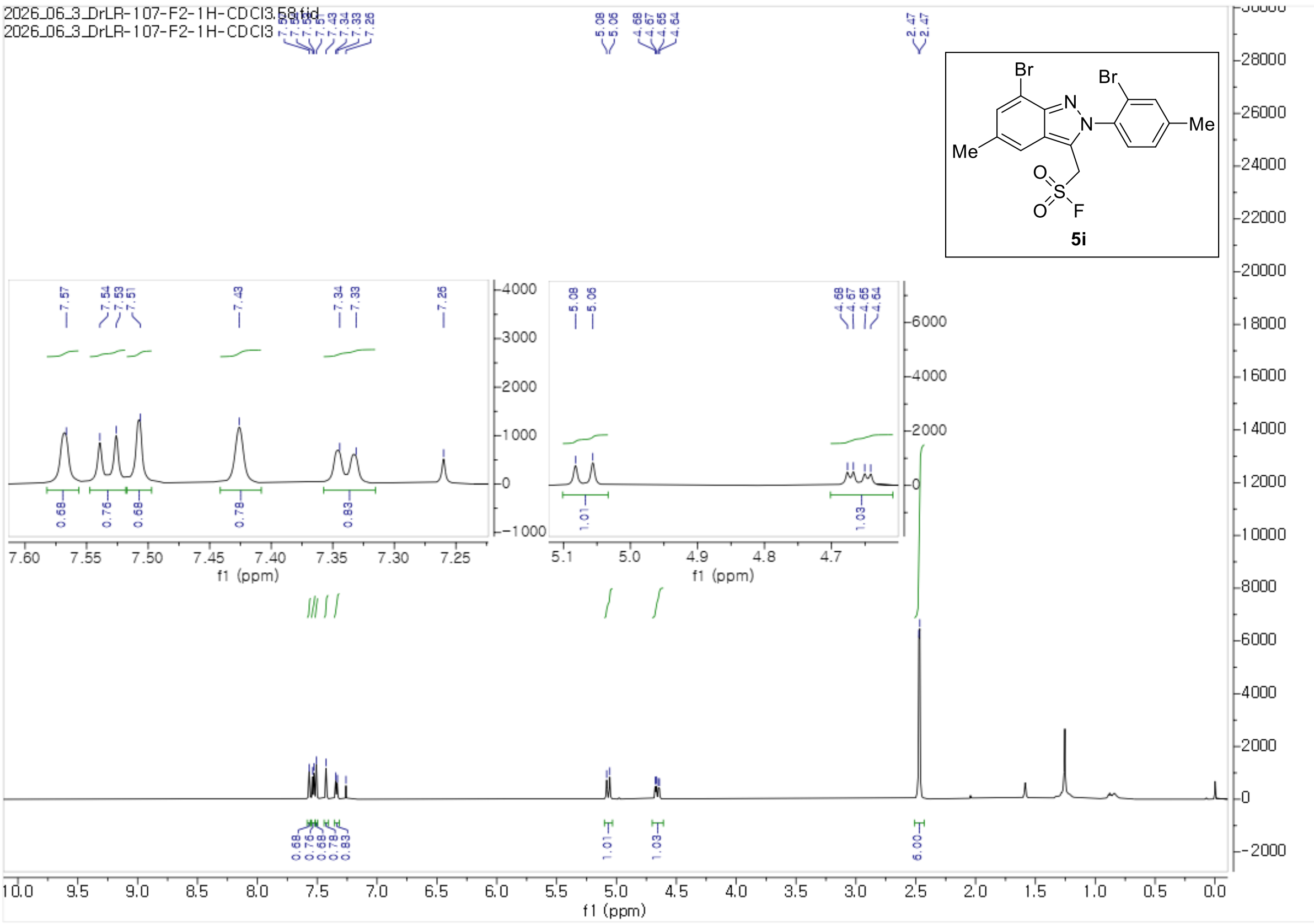
2026_06_3_DrLR-107-F2-1H-CDCl3.58.fid
2026_06_3_DrLR-107-F2-1H-CDCl3
Br
Br
N
N
Me
Me
O
S
O
F
5i
f1 (ppm)

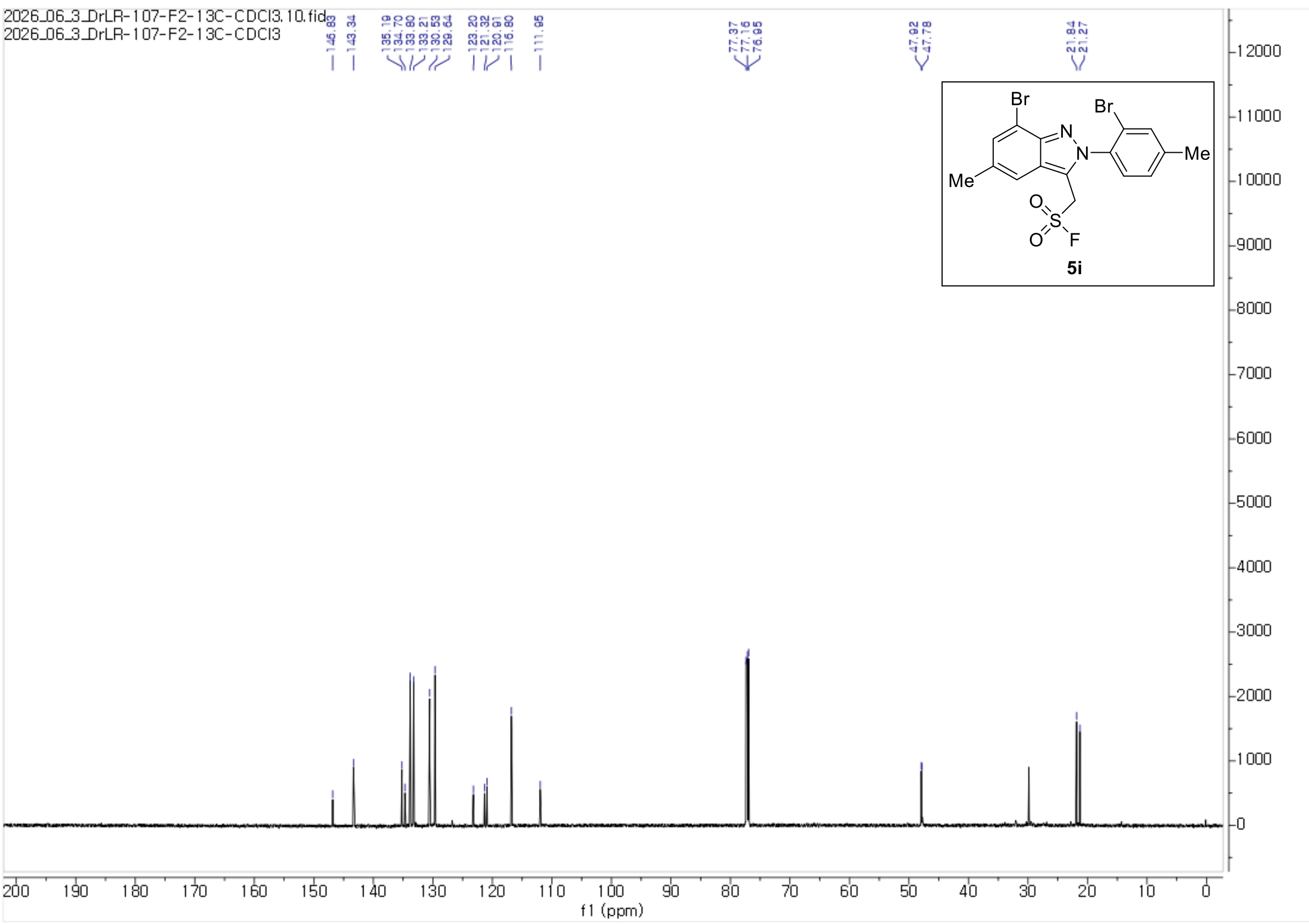
2026_06_3_DrLR-107-F2-13C-CDCl3.10.fid
2026_06_3_DrLR-107-F2-13C-CDCl3
146.83
143.34
135.19
134.70
133.80
133.21
130.53
129.64
123.20
121.32
120.91
116.80
111.95
77.37
77.16
76.95
47.92
47.78
21.84
21.27
Br
Br
N
N
Me
Me
O
S
O
F
5i
f1 (ppm)

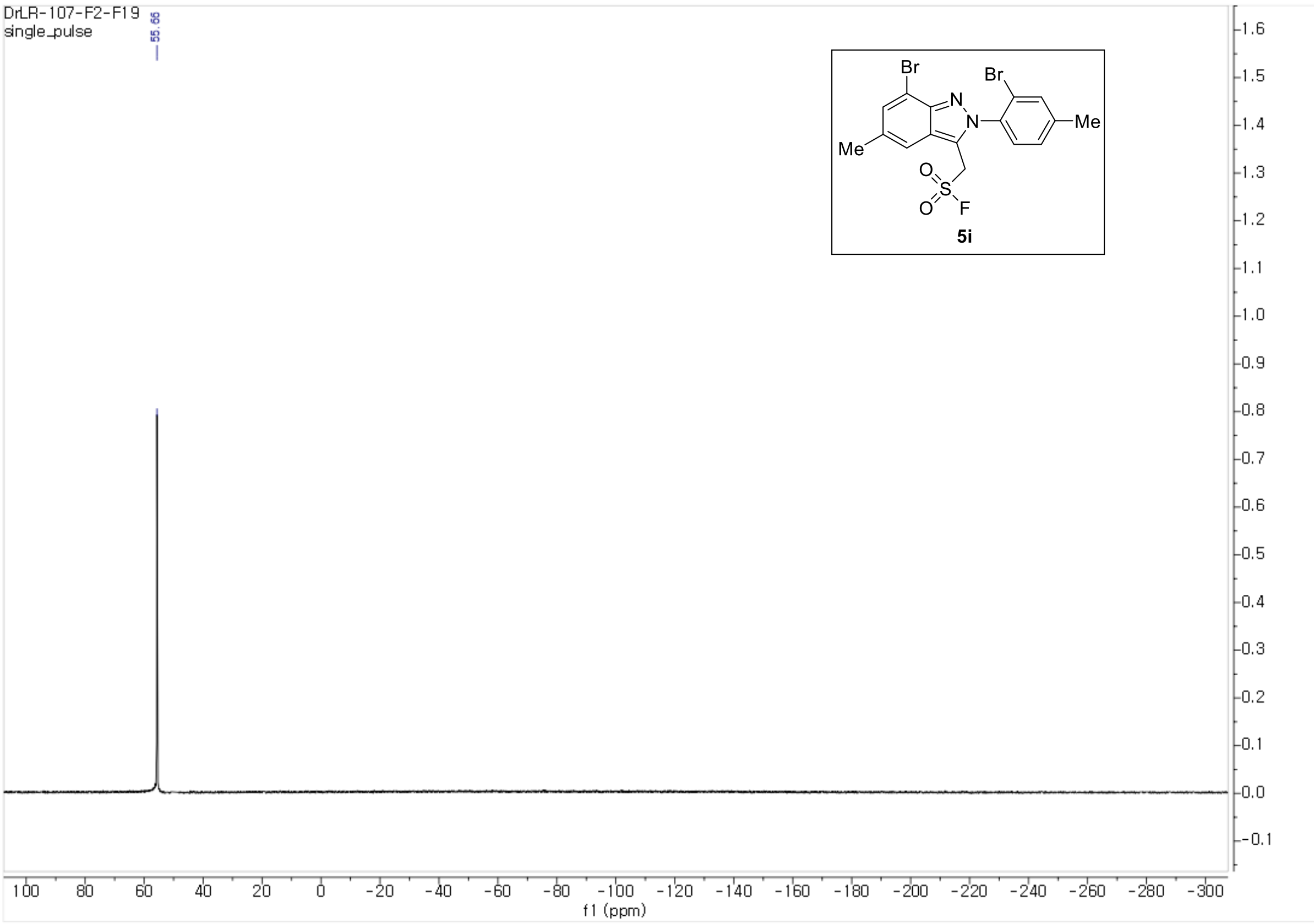
DrLR-107-F2-F19
single_pulse
55.66
Br
Br
N
N
Me
Me
O
S
O
F
5i
f1 (ppm)

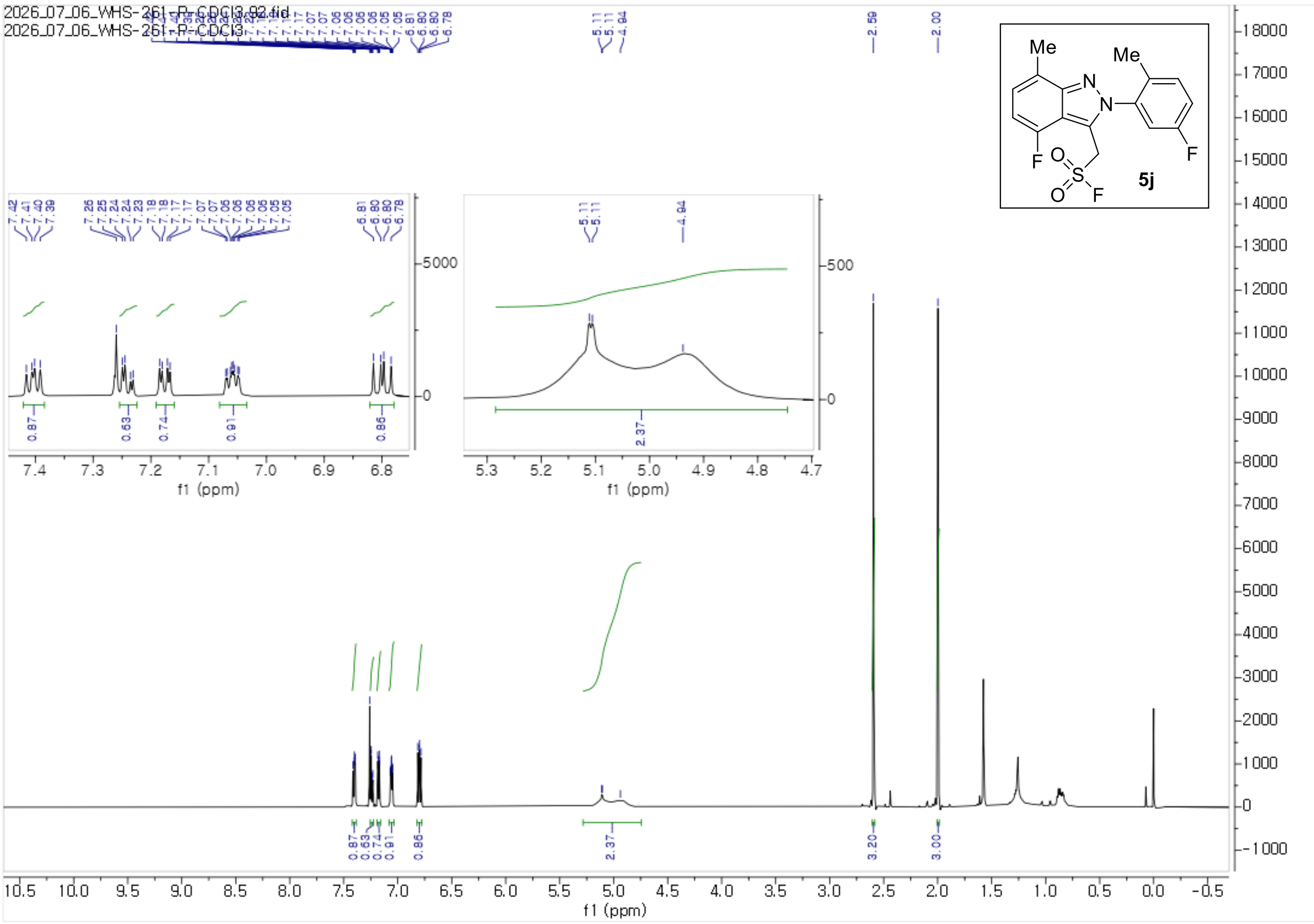
2026_07_06_WHS-261-P-CDCl3.92.fid
2026_07_06_WHS-261-P-CDCl3
5.11
5.11
4.94
2.59
2.00
Me
Me
N
N
F
O
S
O
F
F
5j
f1 (ppm)

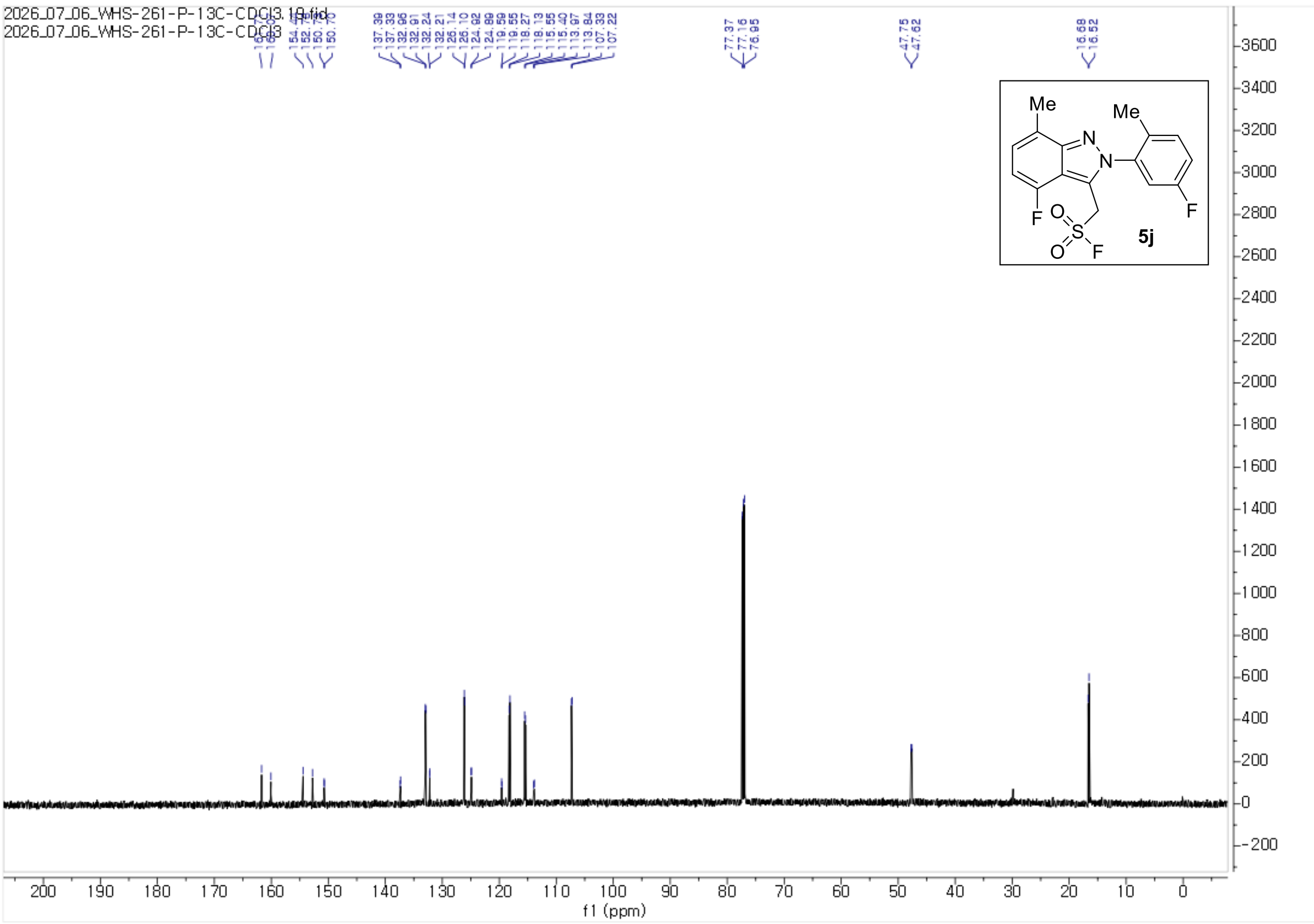
2026_07_06_WHS-261-P-13C-CDCl3.19.fid
2026_07_06_WHS-261-P-13C-CDCl3
137.39
137.33
132.96
132.91
132.24
132.21
125.10
124.92
124.89
119.59
119.55
118.27
118.13
115.55
115.40
113.97
113.84
107.33
107.22
77.37
77.16
76.95
47.75
47.62
16.58
16.52
Me
Me
N
N
F
O
S
O
F
F
5j
f1 (ppm)

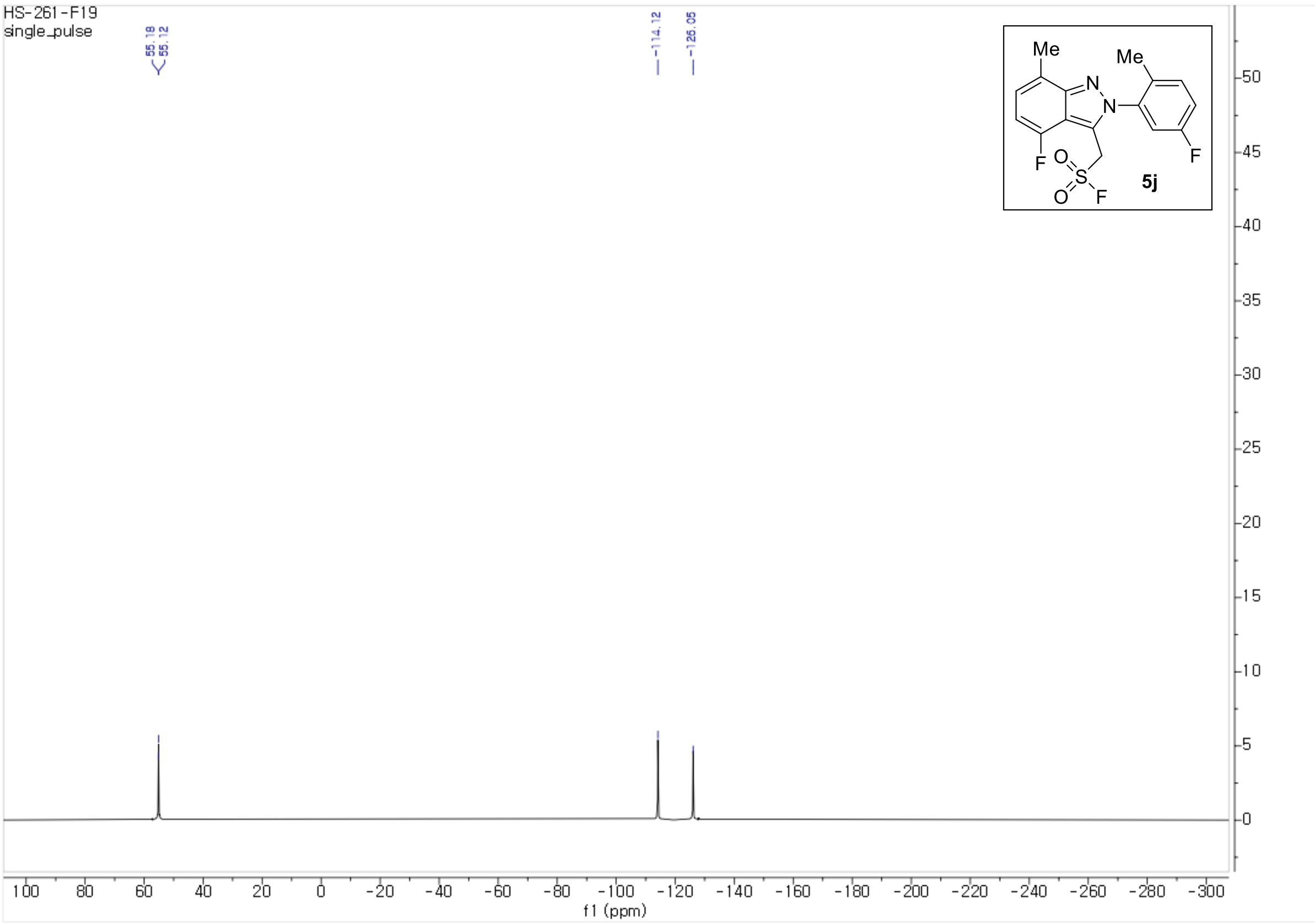
HS-261-F19
single_pulse
55.18
55.12
-114.12
-126.05
Me
Me
N
N
F
O
S
O
F
F
5j
f1 (ppm)

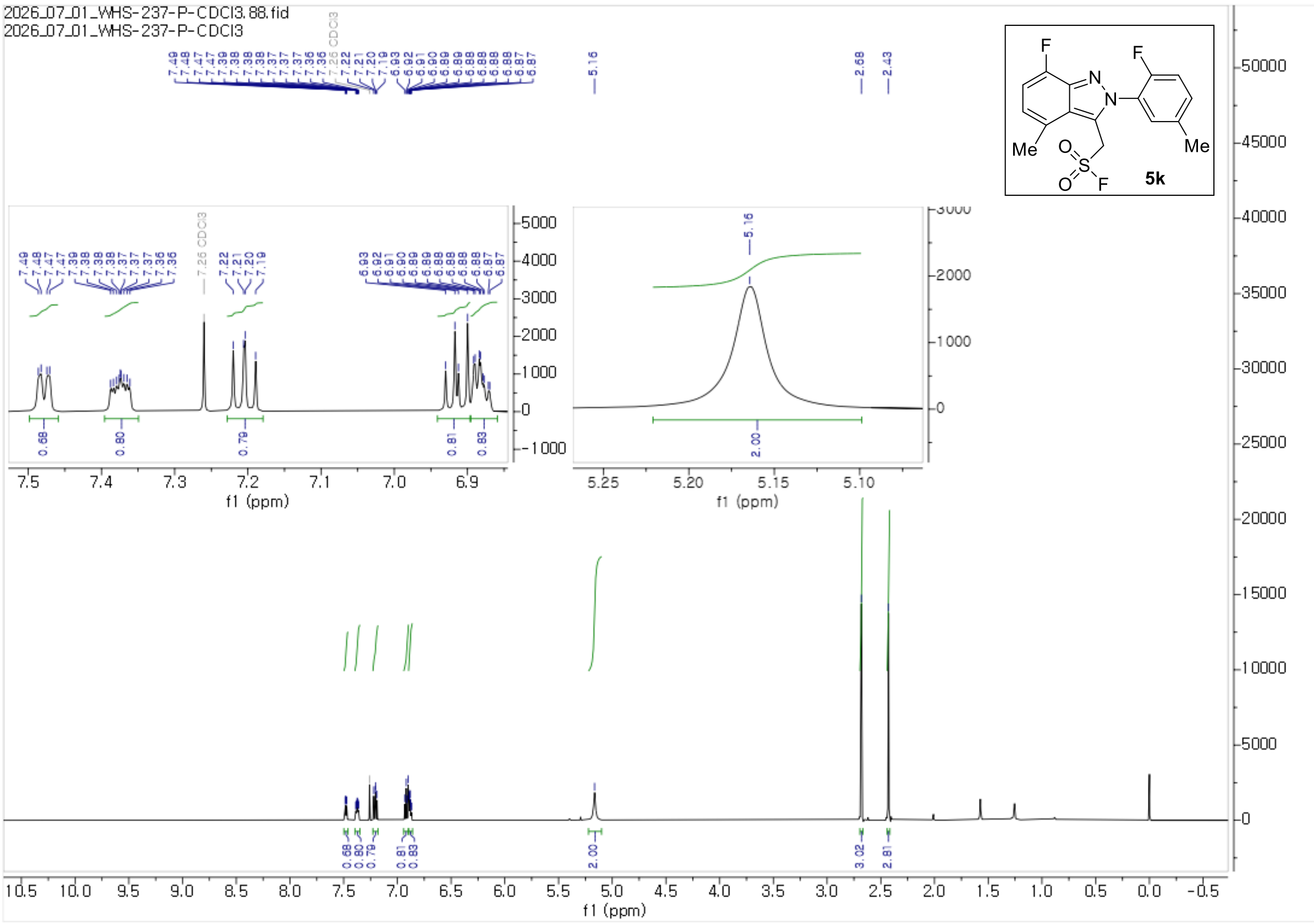
2026_07_01_WHS-237-P-CDCl3.88.fid
2026_07_01_WHS-237-P-CDCl3
5k
f1 (ppm)

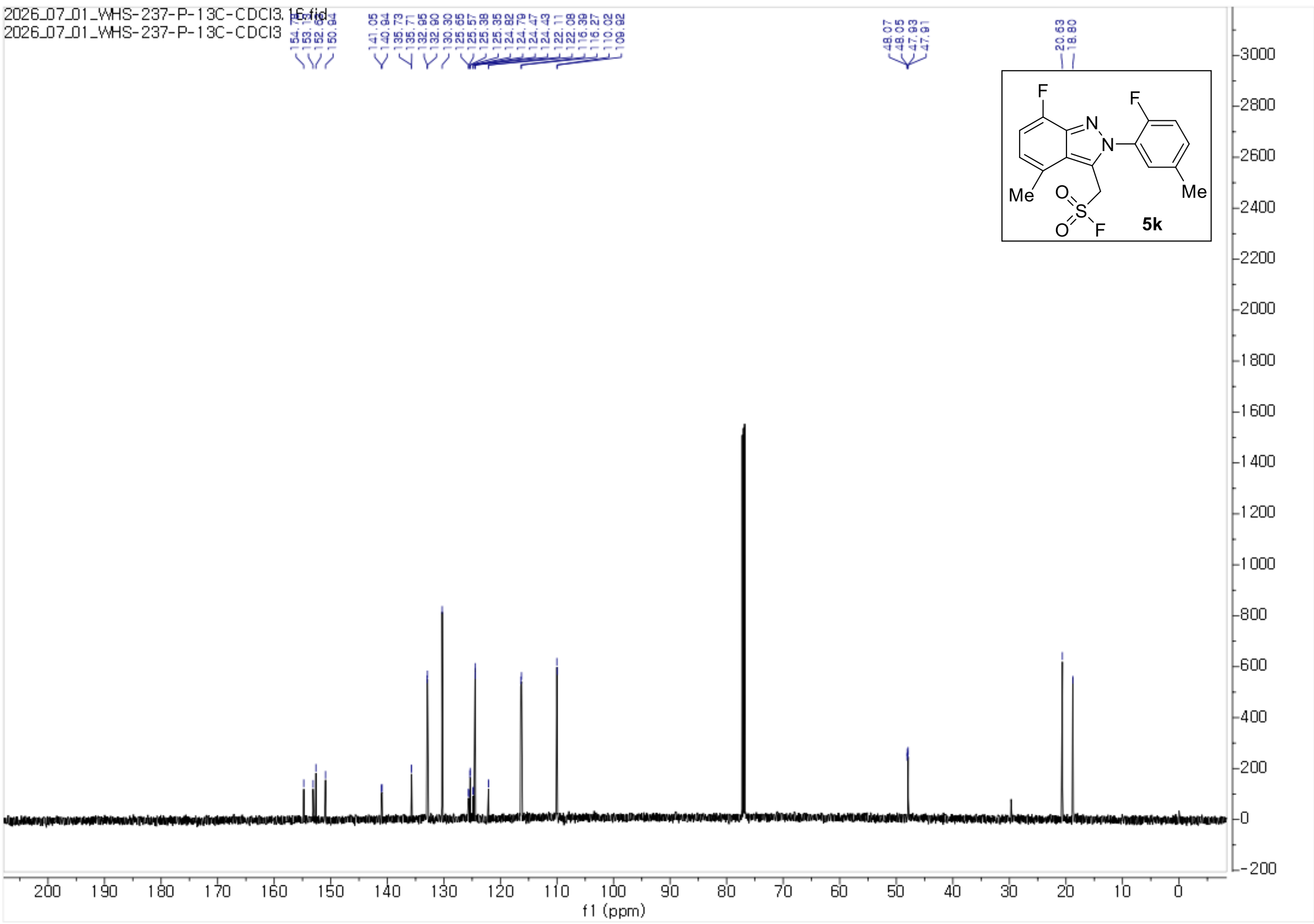
2026_07_01_WHS-237-P-13C-CDCl3.16.fid
2026_07_01_WHS-237-P-13C-CDCl3
5k
f1 (ppm)

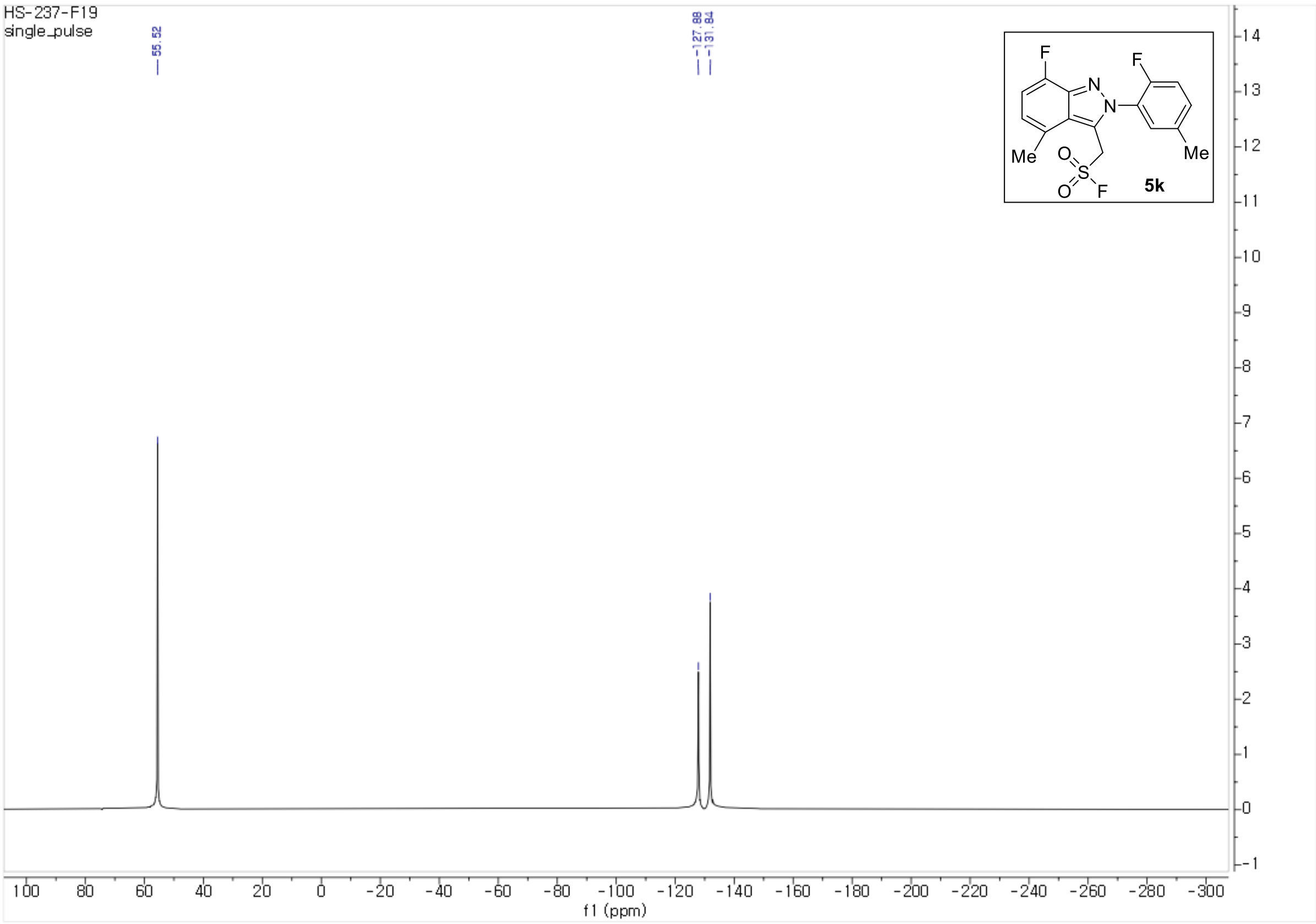
HS-237-F19
single_pulse
55.52
-127.88
-131.84
F
N
N
F
Me
Me
O
S
O
F
5k
f1 (ppm)

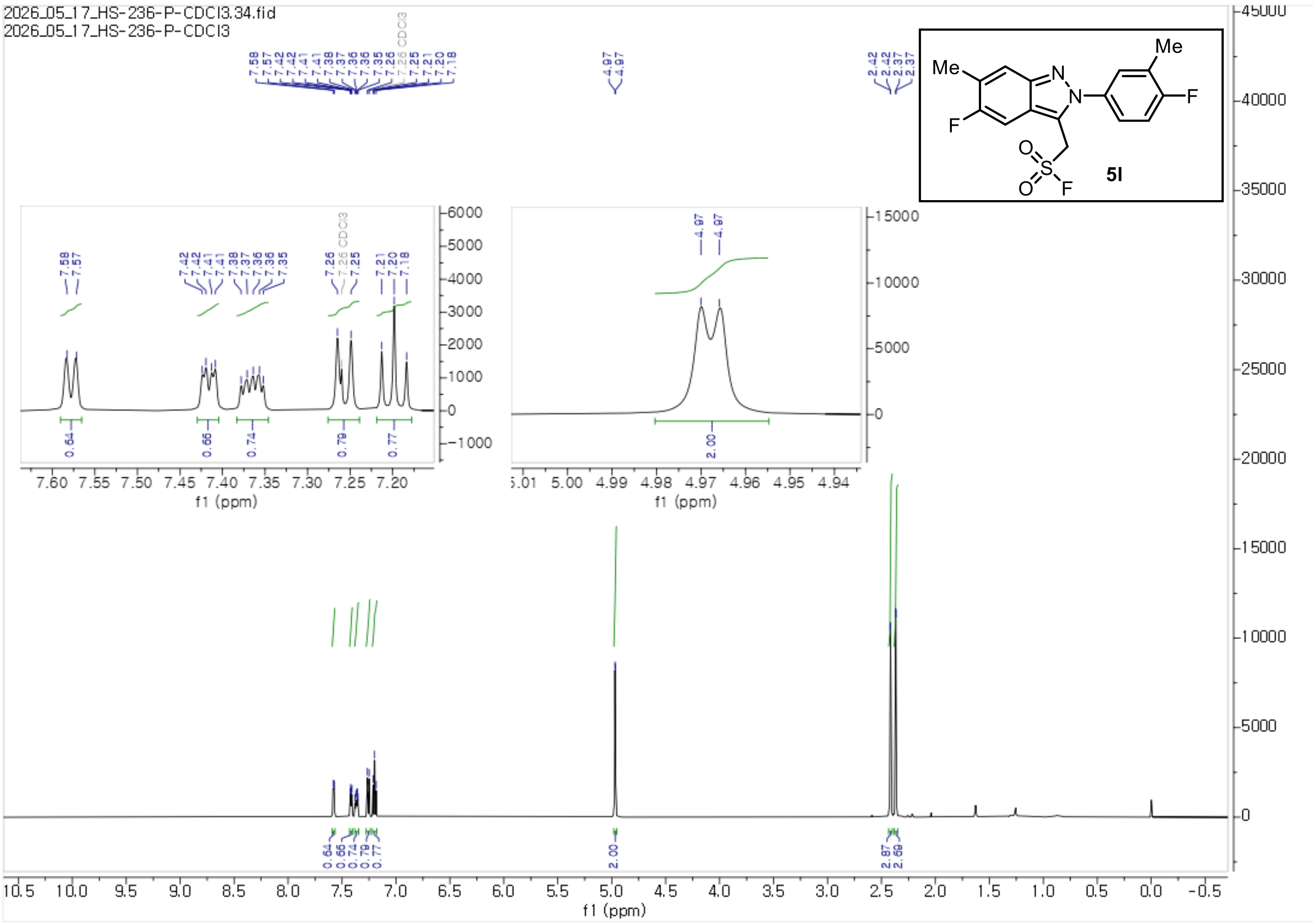
2026_05_17_HS-236-P-CDCl3.34.fid
2026_05_17_HS-236-P-CDCl3
Me
N
N
Me
F
F
O
S
O
F
5l
f1 (ppm)

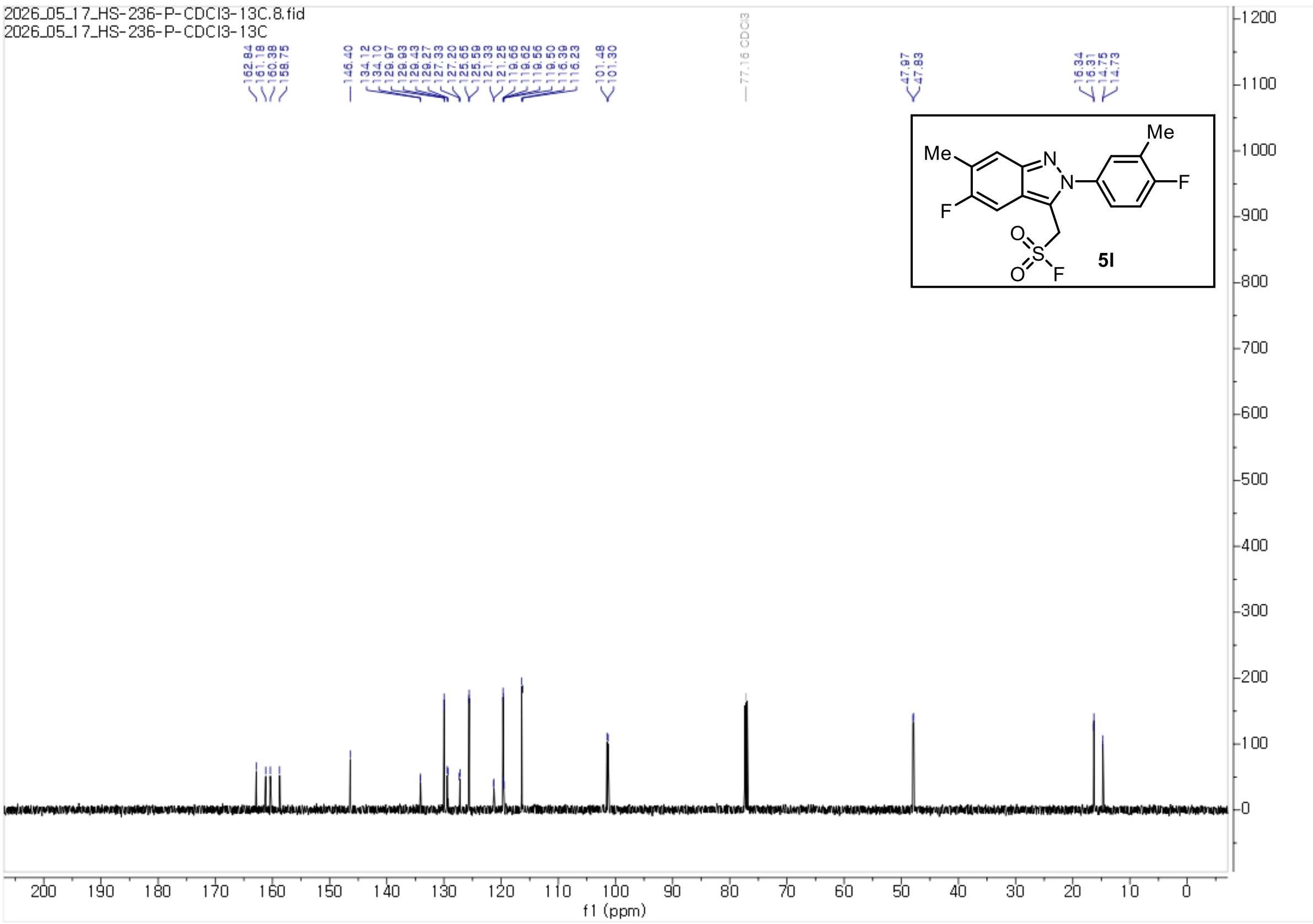
2026_05_17_HS-236-P-CDCl3-13C.8.fid
2026_05_17_HS-236-P-CDCl3-13C
Me
N
N
Me
F
F
O
S
O
F
5l
f1 (ppm)

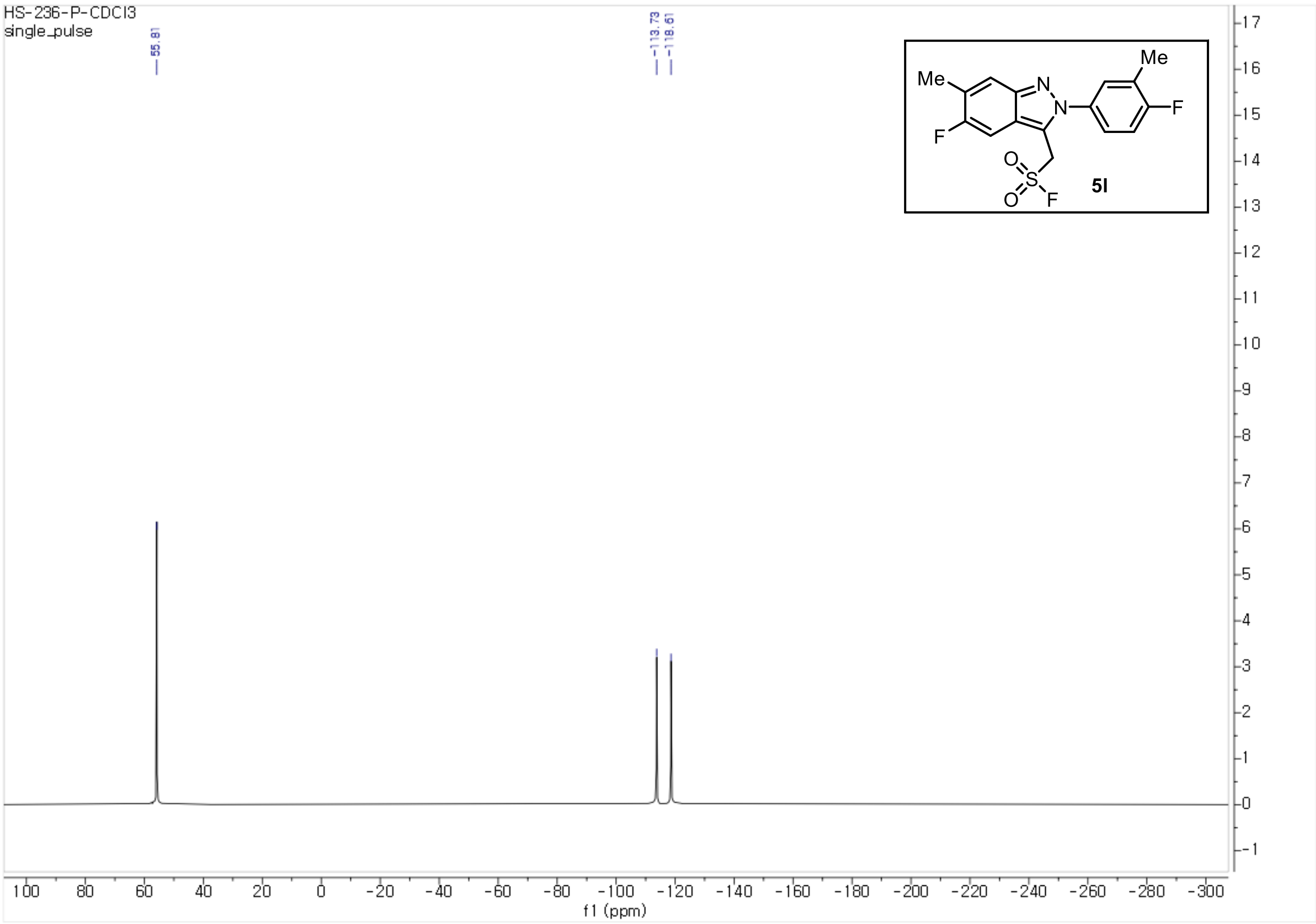

HS-236-P-CDCl3
single_pulse
55.81
-113.73
-118.61
Me
N
N
Me
F
F
O
S
O
F
5l
f1 (ppm)

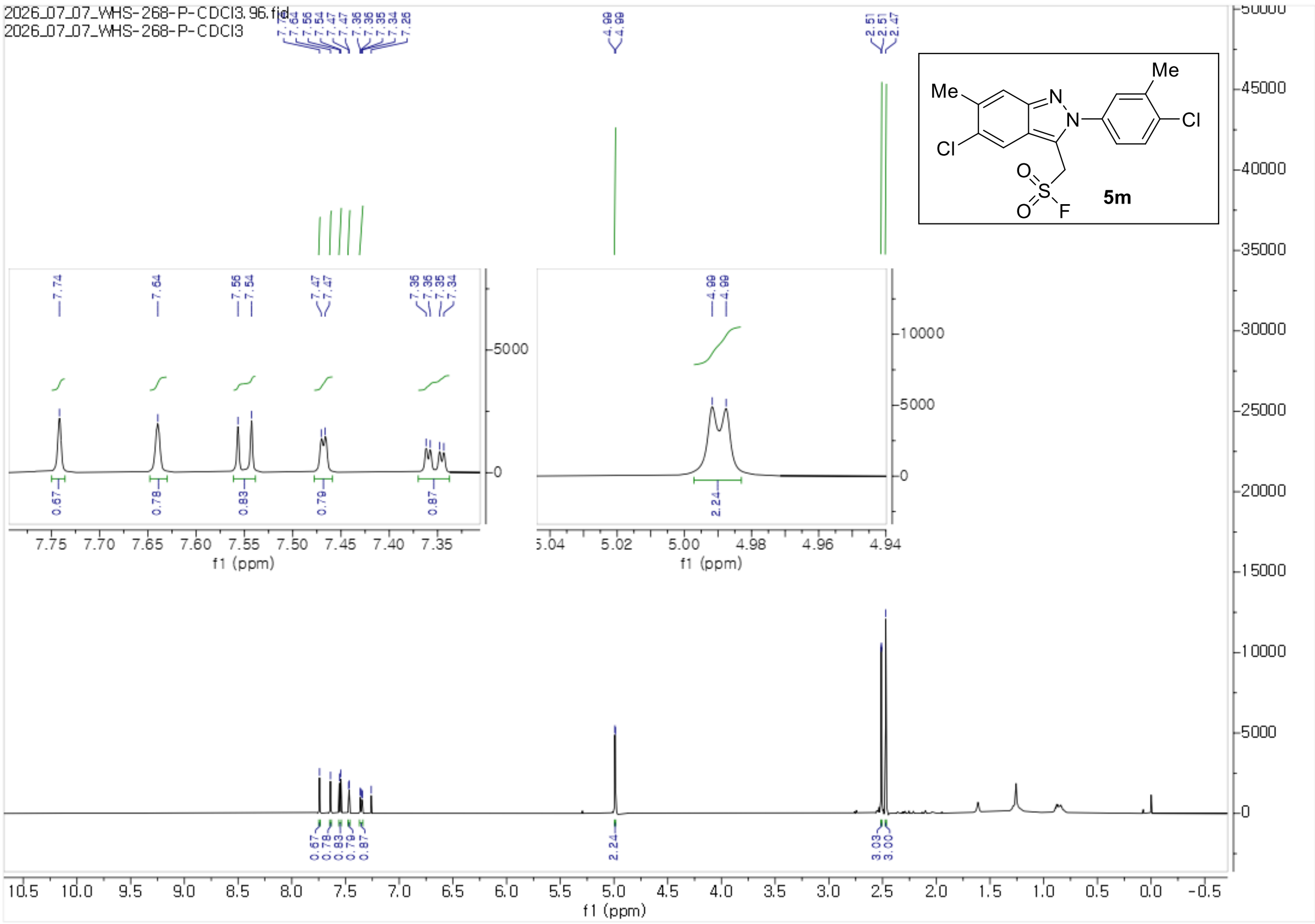

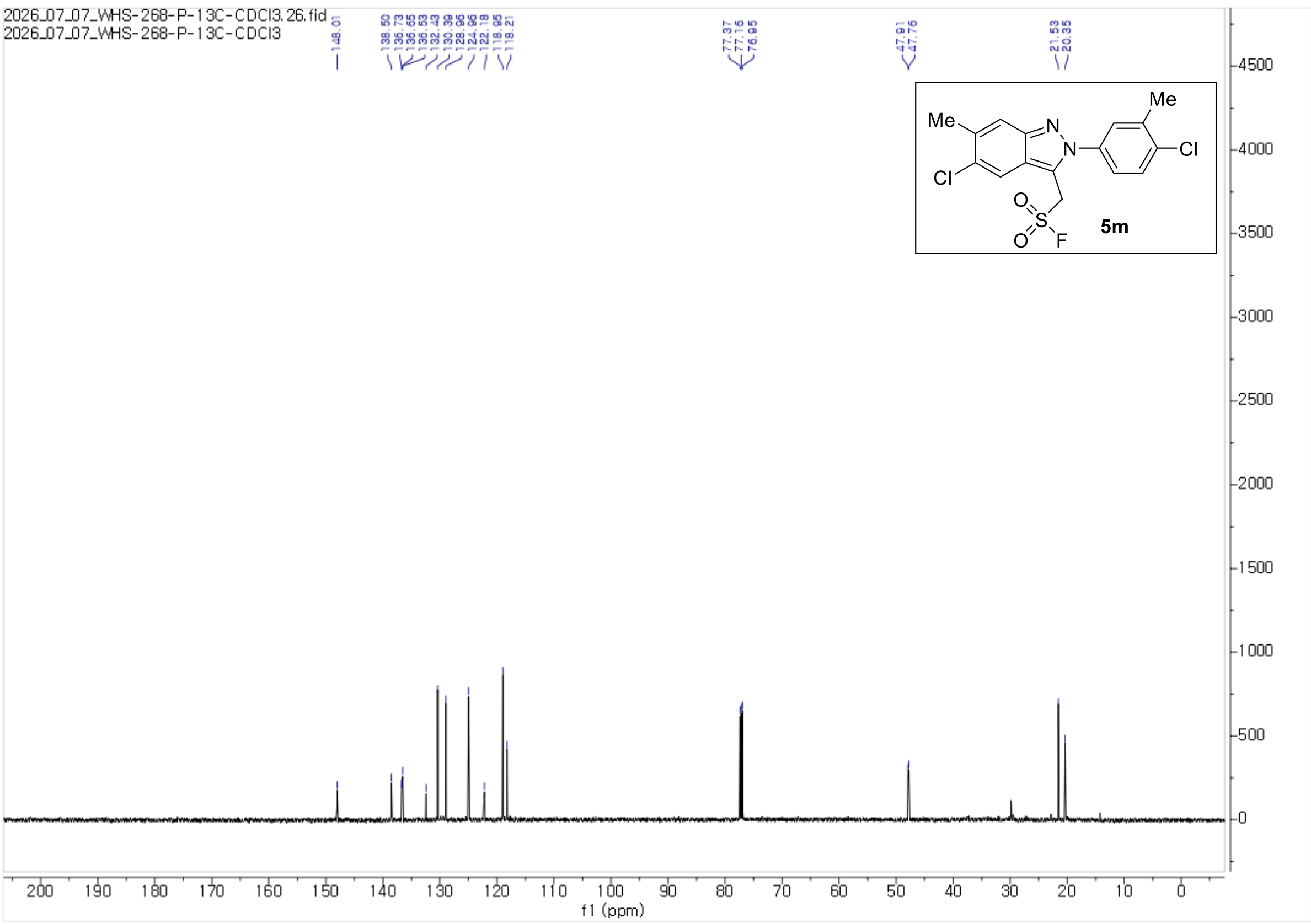

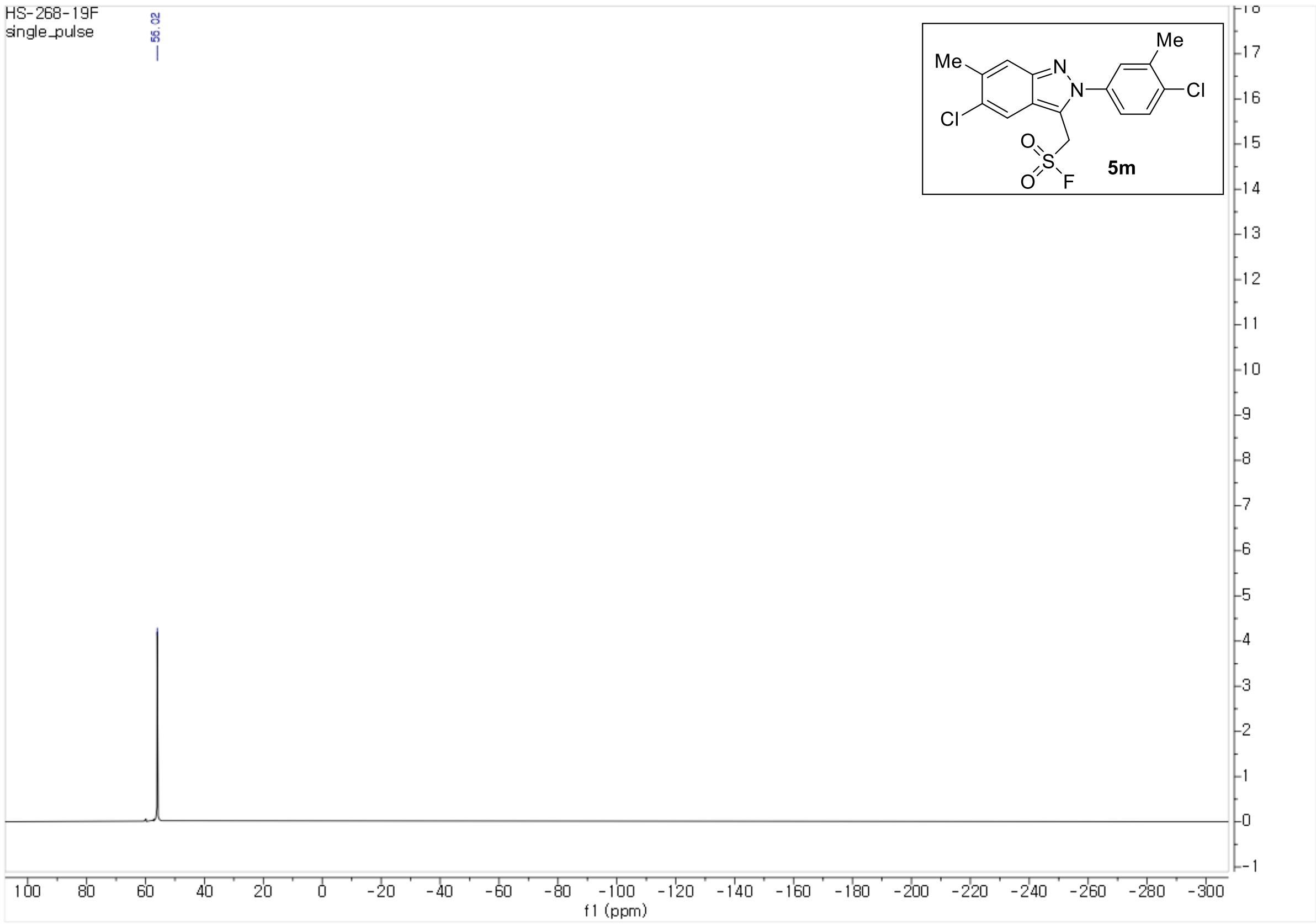
HS-268-19F
single_pulse
55.02
Me
N
N
Me
Cl
Cl
O
S
O
F
5m
f1 (ppm)

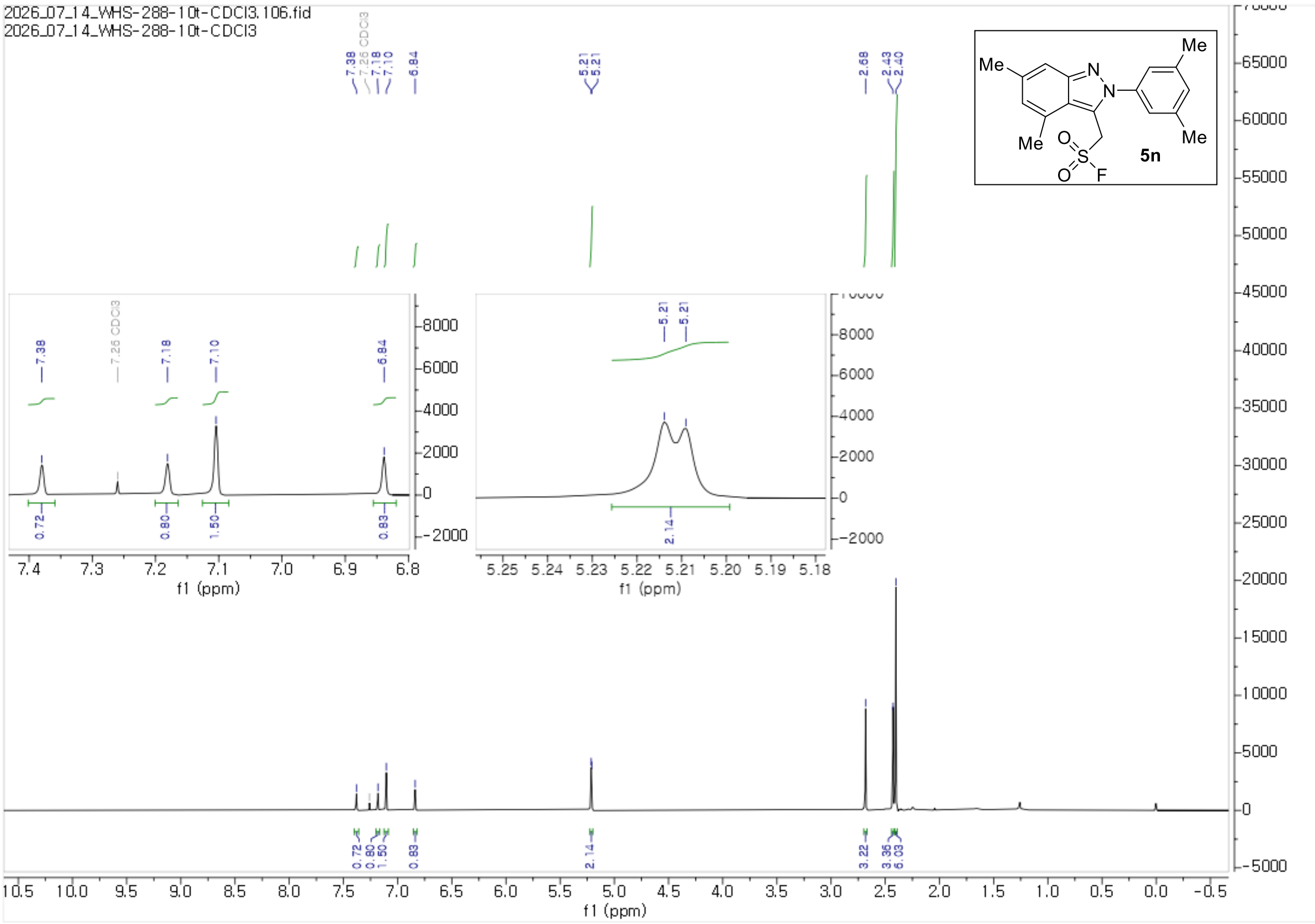
2026_07_14_WHS-288-10t-CDCl3.106.fid
2026_07_14_WHS-288-10t-CDCl3
5n
f1 (ppm)

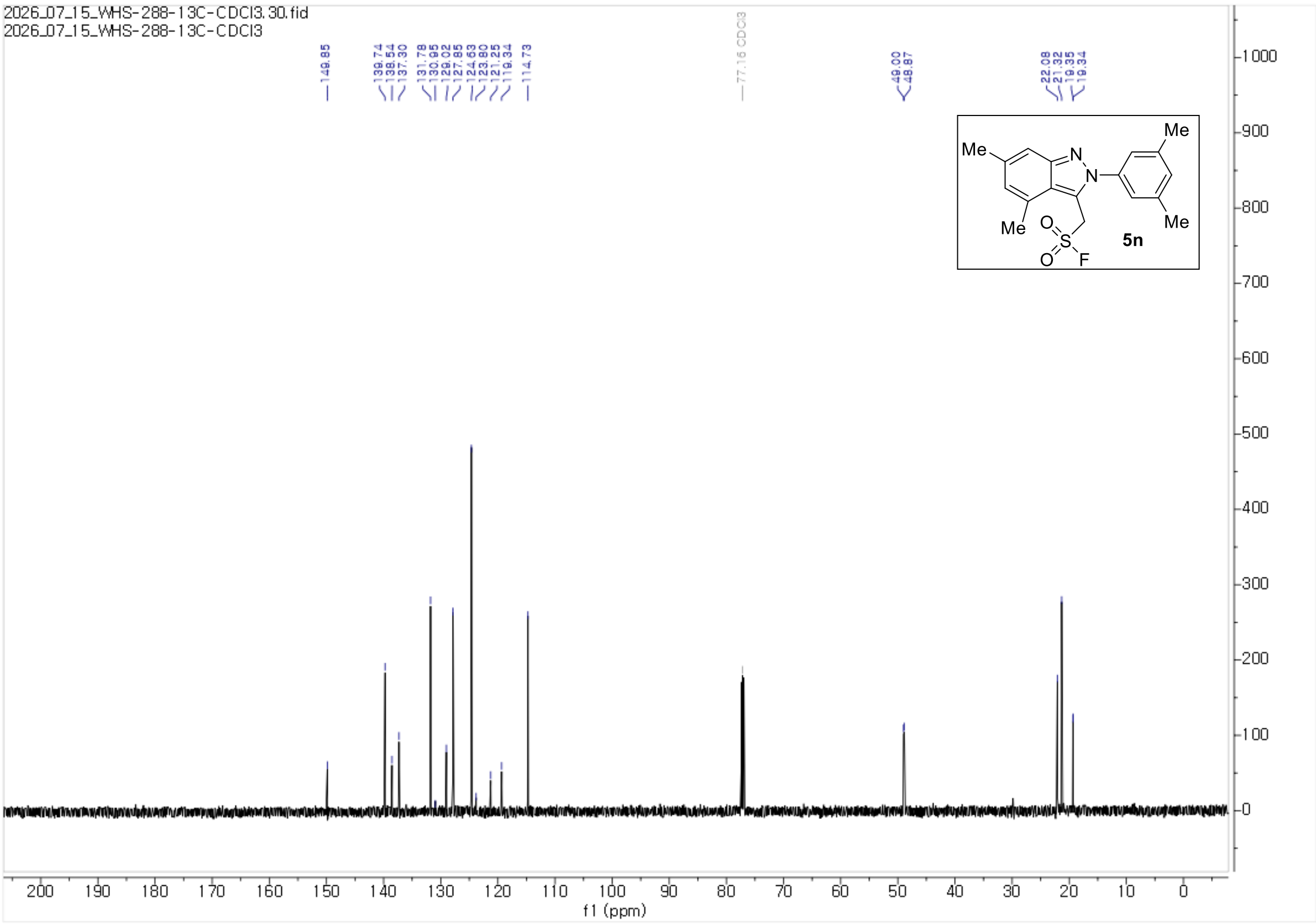
2026_07_15_WHS-288-13C-CDCl3.30.fid
2026_07_15_WHS-288-13C-CDCl3
5n
f1 (ppm)

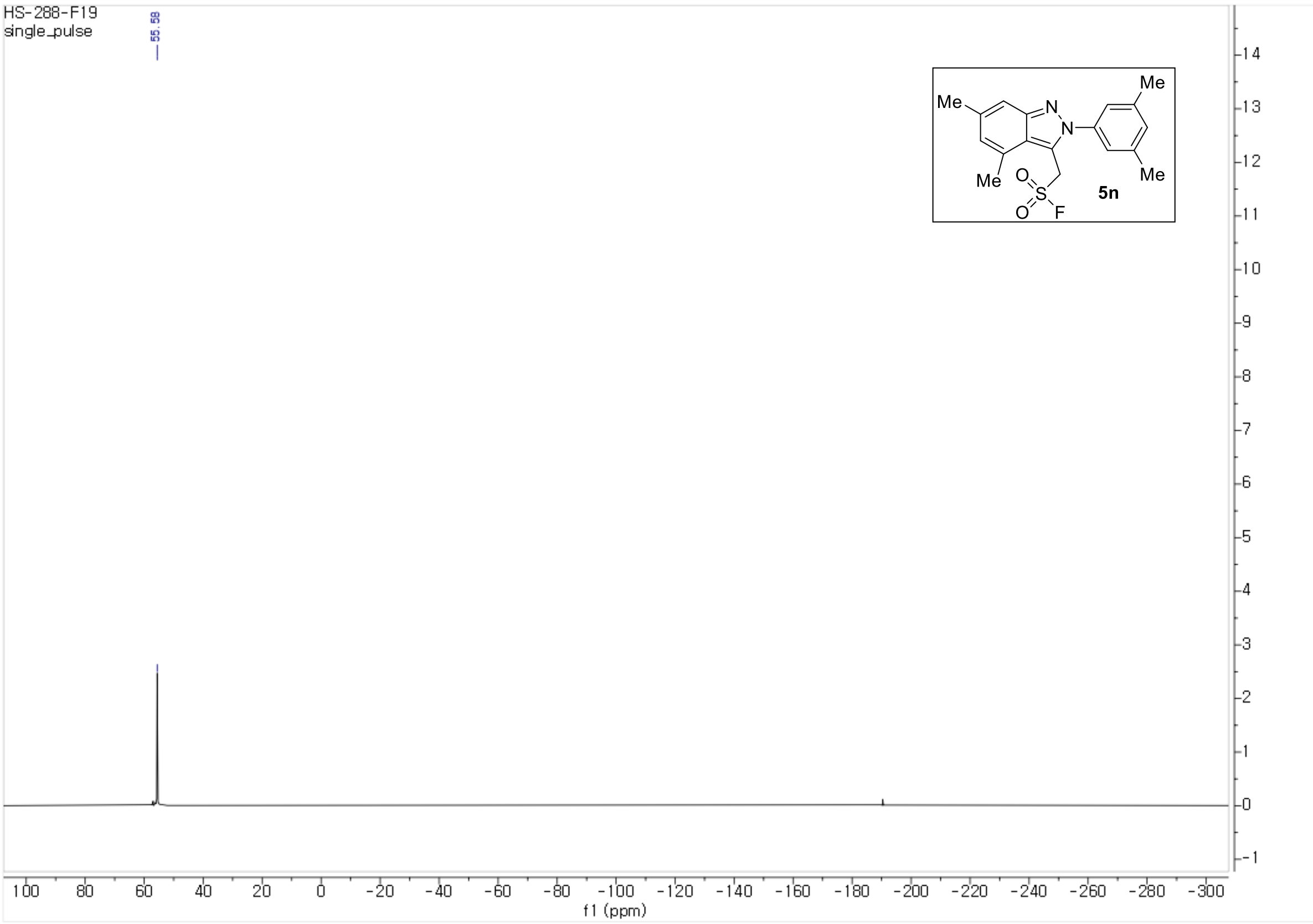
HS-288-F19
single_pulse
55.59
Me
N
N
Me
Me
O
S
O
F
Me
5n
f1 (ppm)

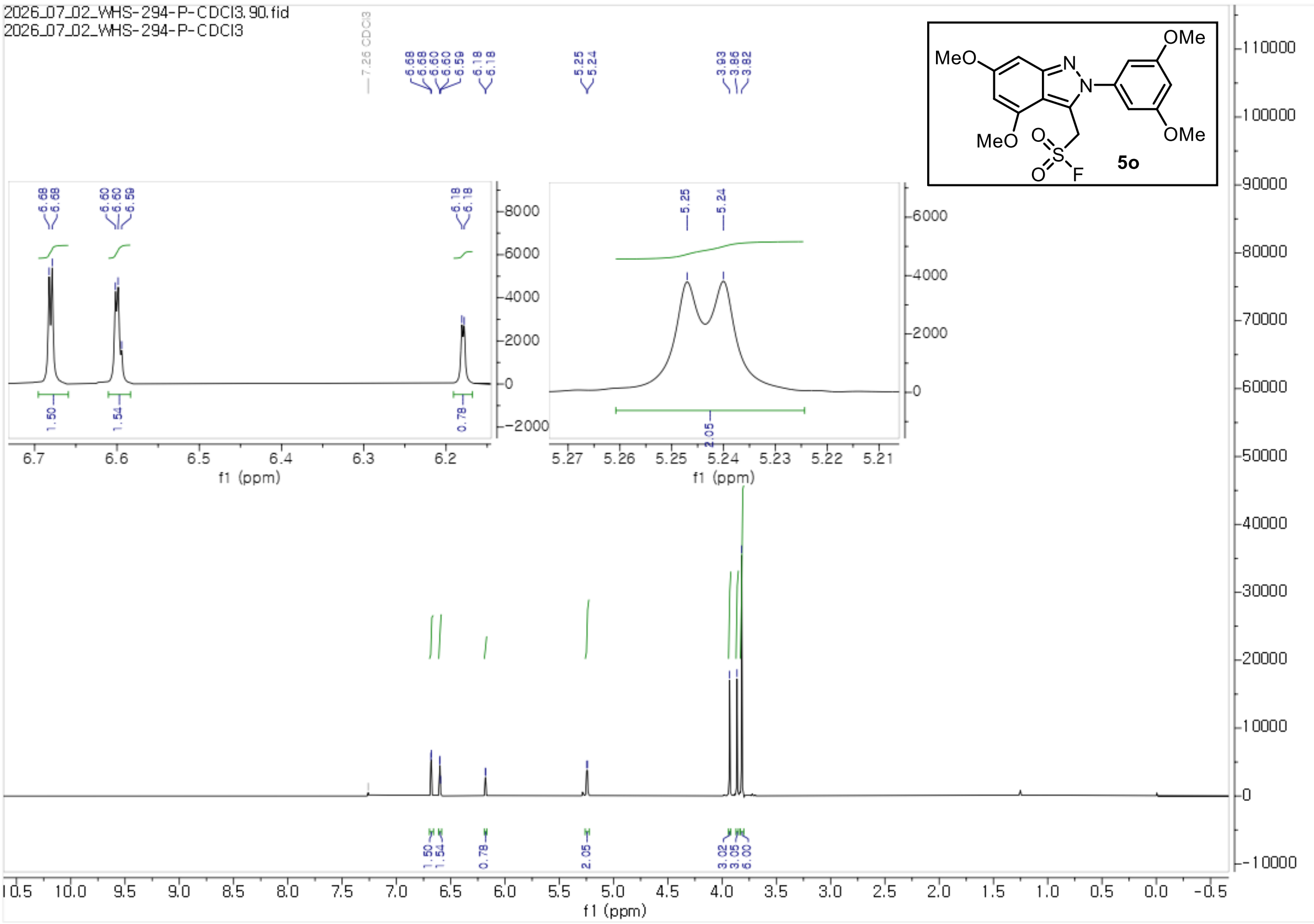
2026_07_02_WHS-294-P-CDCl3.90.fid
2026_07_02_WHS-294-P-CDCl3
7.26 CDCl3
6.68
6.68
6.60
6.60
6.59
6.18
6.18
5.25
5.24
3.93
3.86
3.82
MeO
N
N
OMe
MeO
O
S
O
F
OMe
5o
1.50
1.54
0.78
2.05
3.02
3.05
6.00
f1 (ppm)

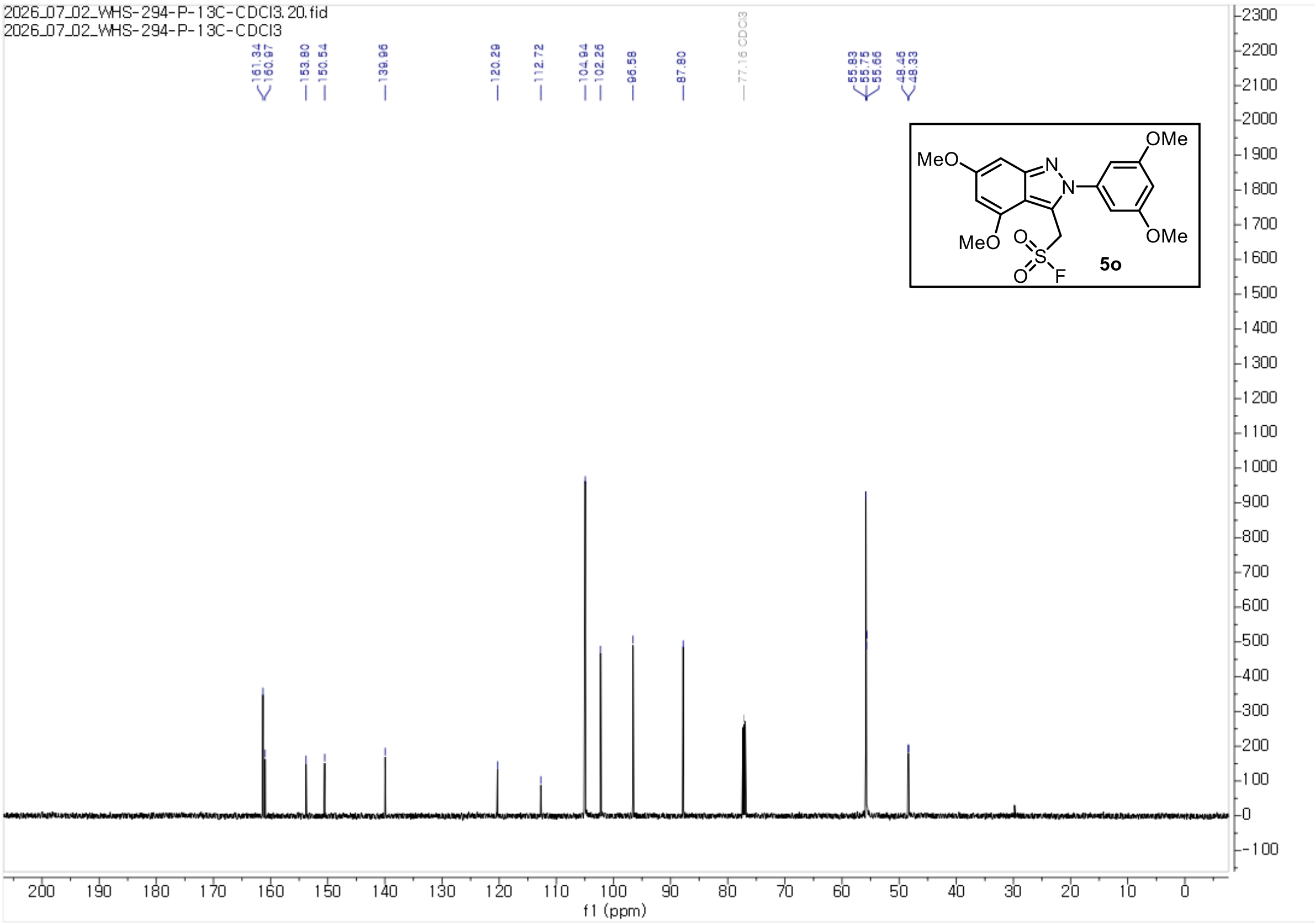
2026_07_02_WHS-294-P-13C-CDCl3.20.fid
2026_07_02_WHS-294-P-13C-CDCl3
161.34
160.97
153.80
150.54
139.96
120.29
112.72
104.94
102.26
96.58
87.80
77.16 CDCl3
55.83
55.75
55.66
48.46
48.33
MeO
N
N
OMe
MeO
O
S
O
F
OMe
5o
f1 (ppm)

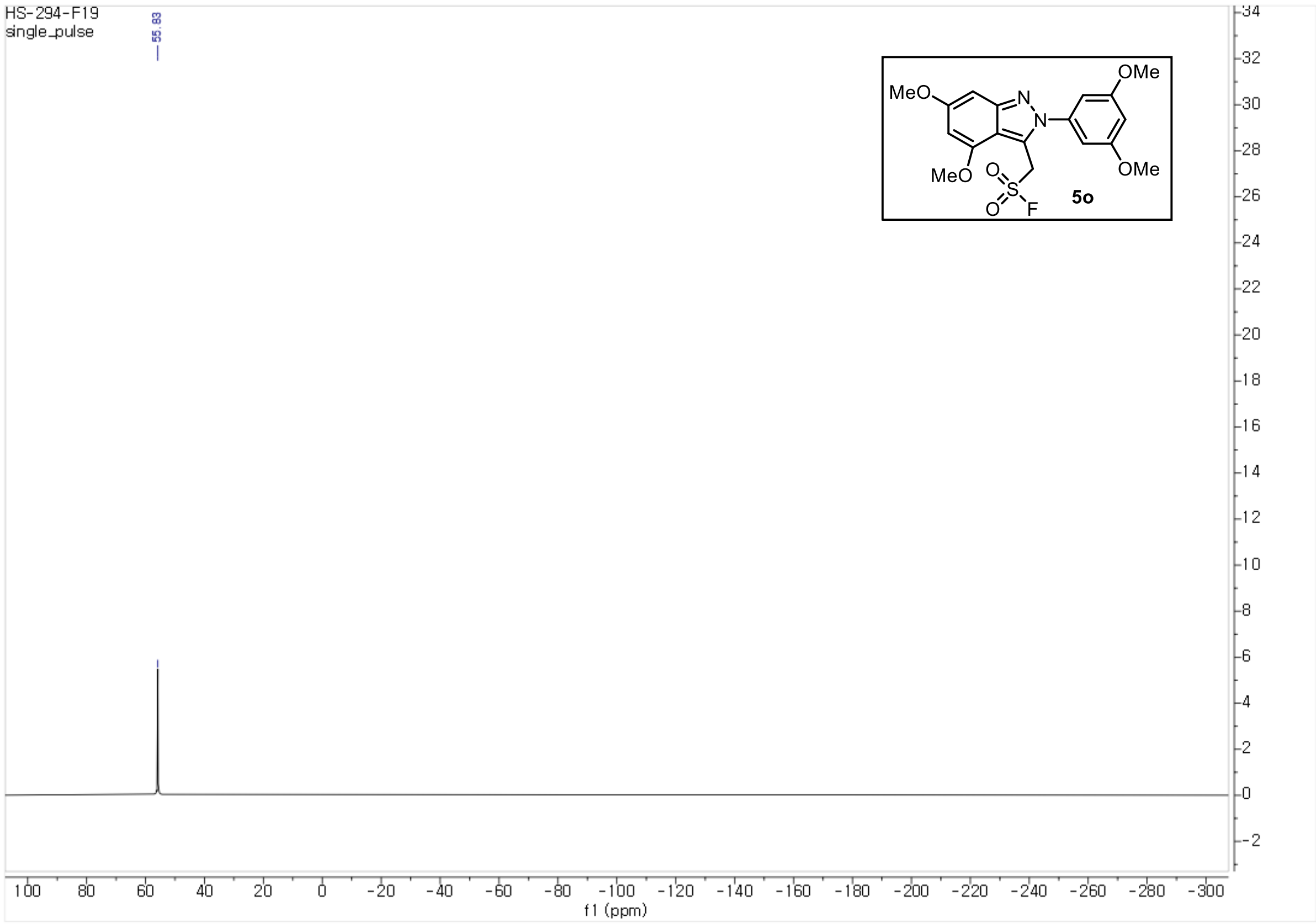
HS-294-F19
single_pulse
55.83
MeO
N
N
OMe
MeO
O
S
O
F
OMe
5o
f1 (ppm)

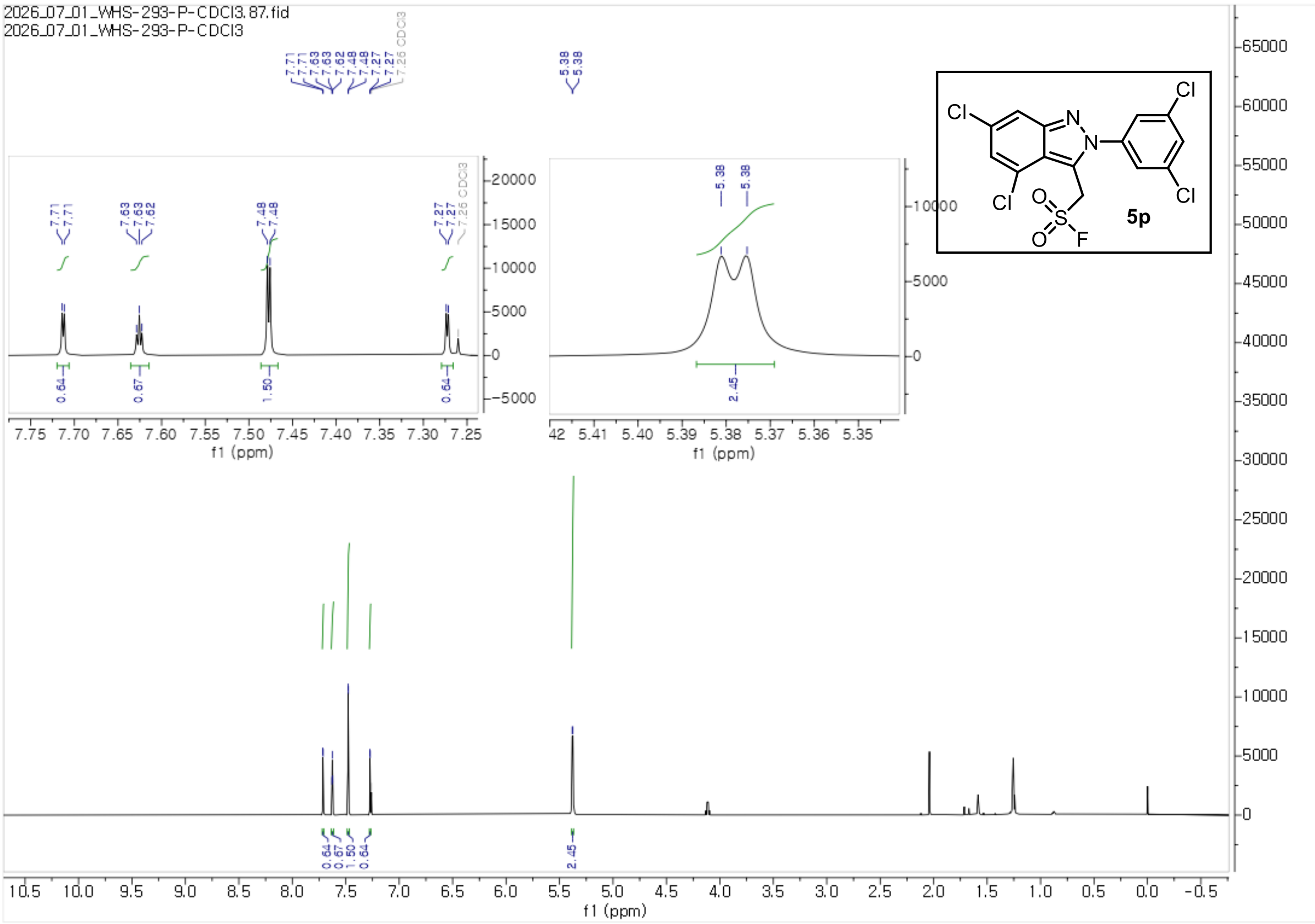
2026_07_01_WHS-293-P-CDCl3.87.fid
2026_07_01_WHS-293-P-CDCl3
7.71 7.71 7.63 7.63 7.62 7.48 7.48 7.27 7.27 7.26 CDCl3
5.38 5.38
5p
f1 (ppm)

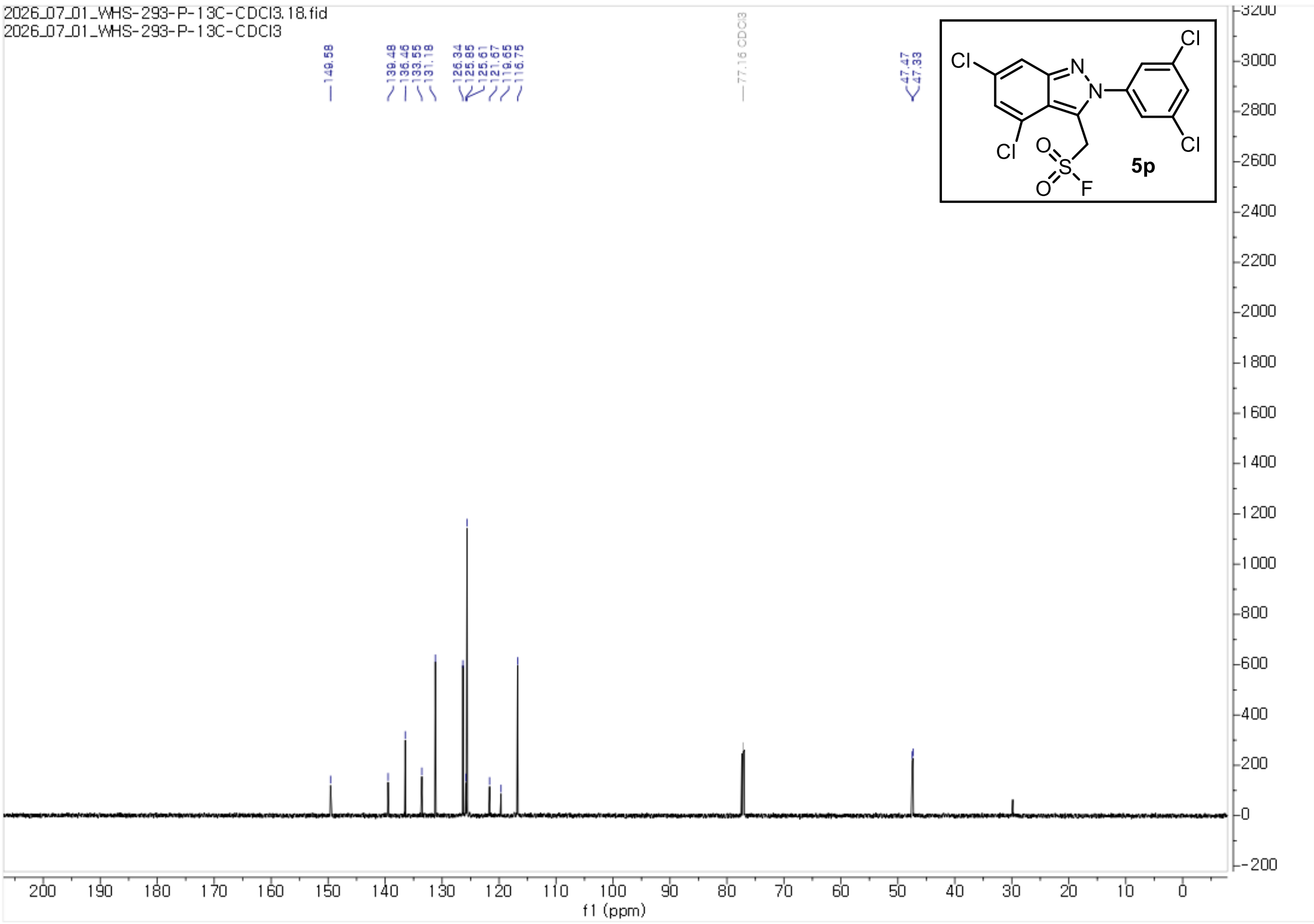
2026_07_01_WHS-293-P-13C-CDCl3.18.fid
2026_07_01_WHS-293-P-13C-CDCl3
149.58
139.48 136.46 133.55 131.18
126.34 125.85 125.61 121.67 119.65 116.75
77.16 CDCl3
47.47 47.33
5p
f1 (ppm)

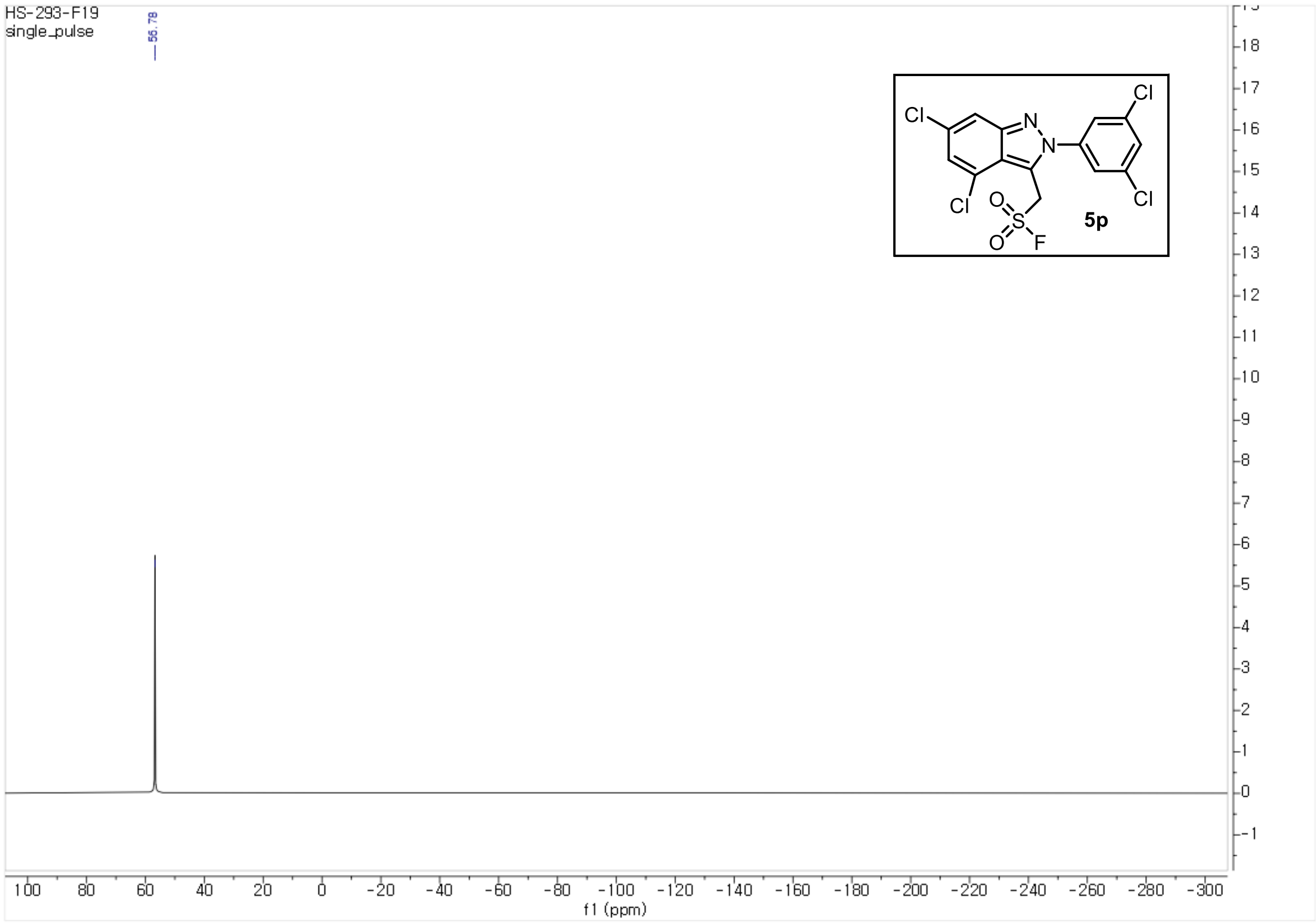
HS-293-F19
single_pulse
55.78
5p
f1 (ppm)

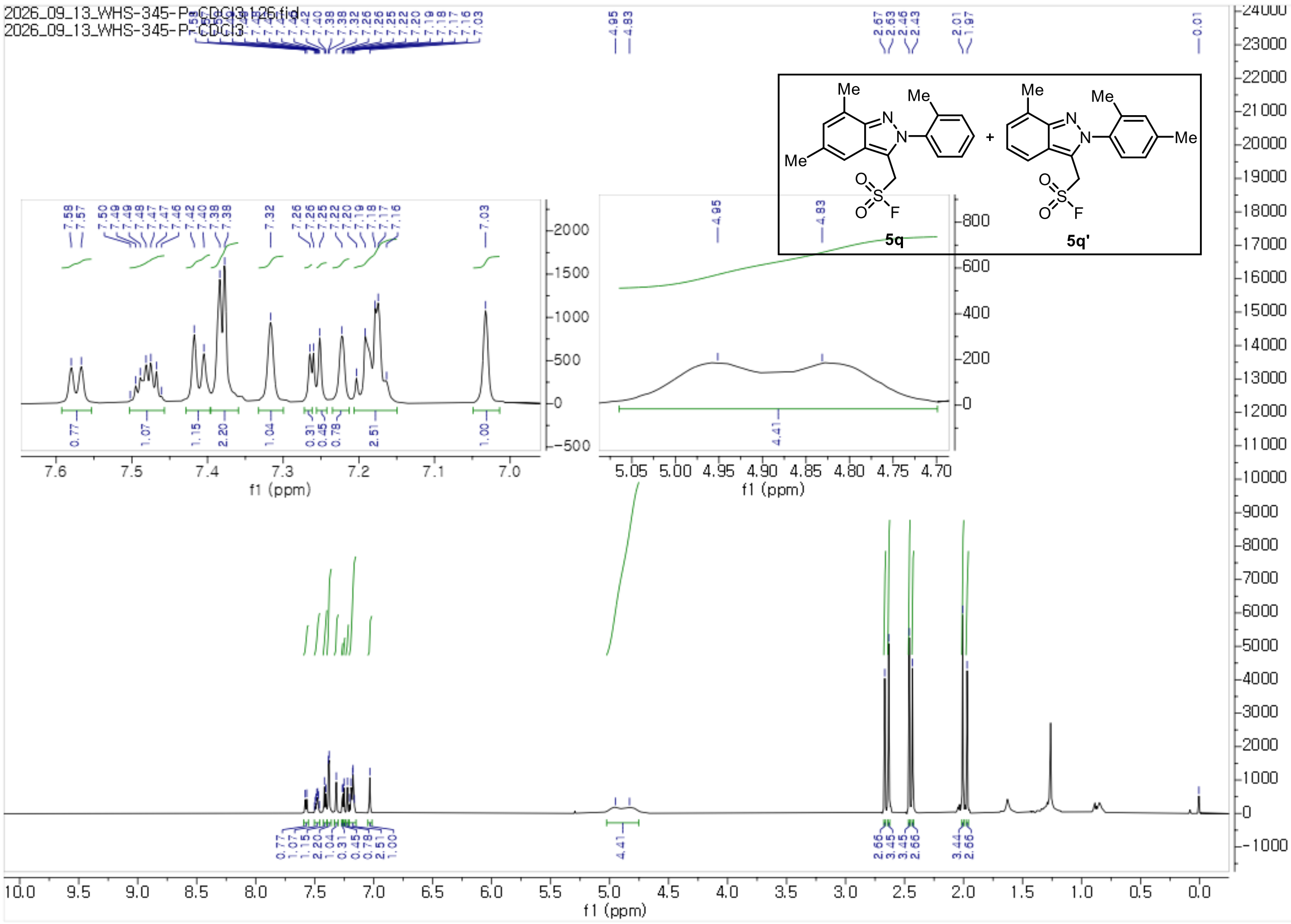
2026_09_13_WHS-345-P-CDCl3-1H.26.fid
2026_09_13_WHS-345-P-CDCl3
5q
5q'
f1 (ppm)

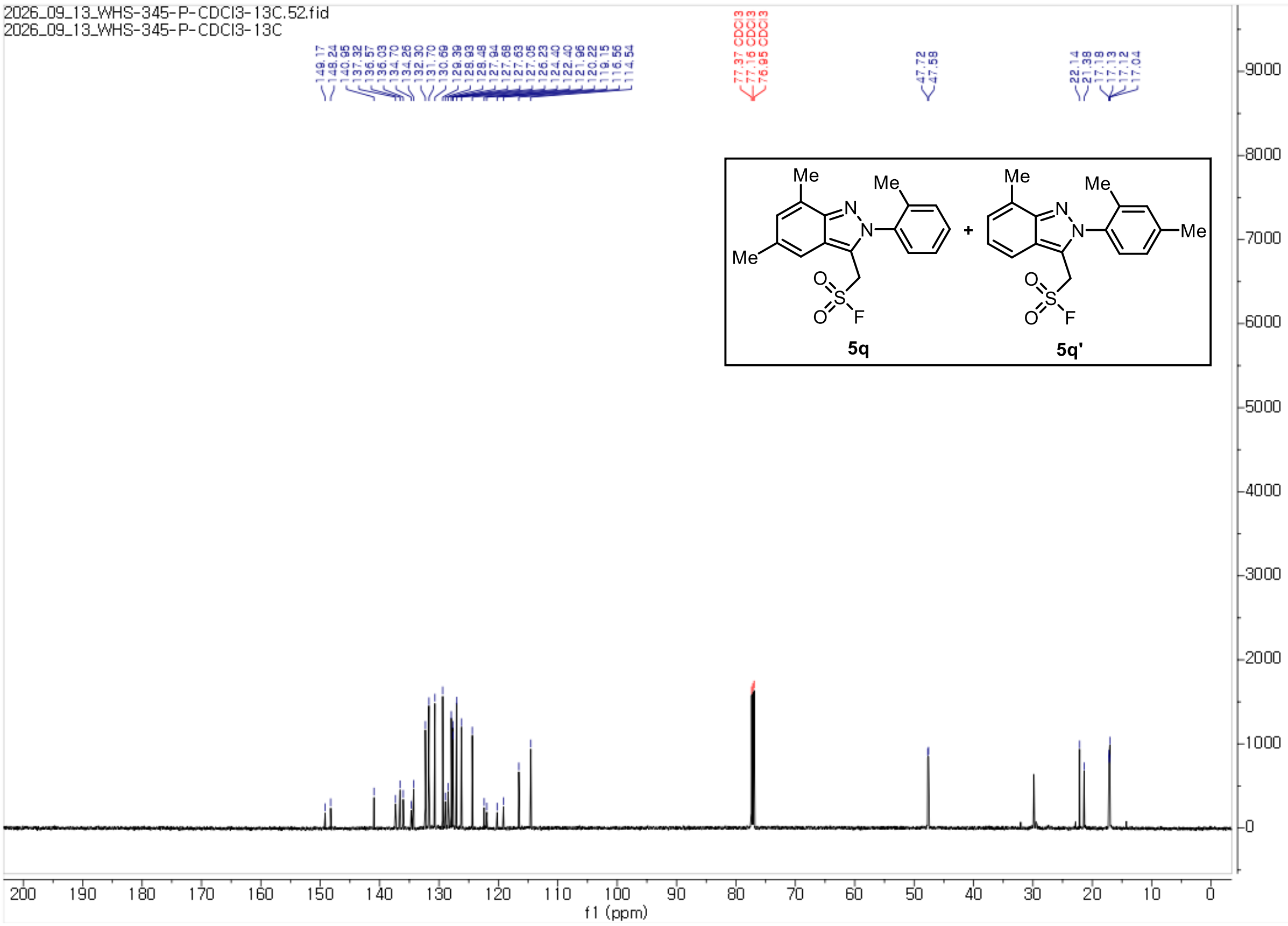
2026_09_13_WHS-345-P-CDCl3-13C.52.fid
2026_09_13_WHS-345-P-CDCl3-13C
5q
5q'
f1 (ppm)

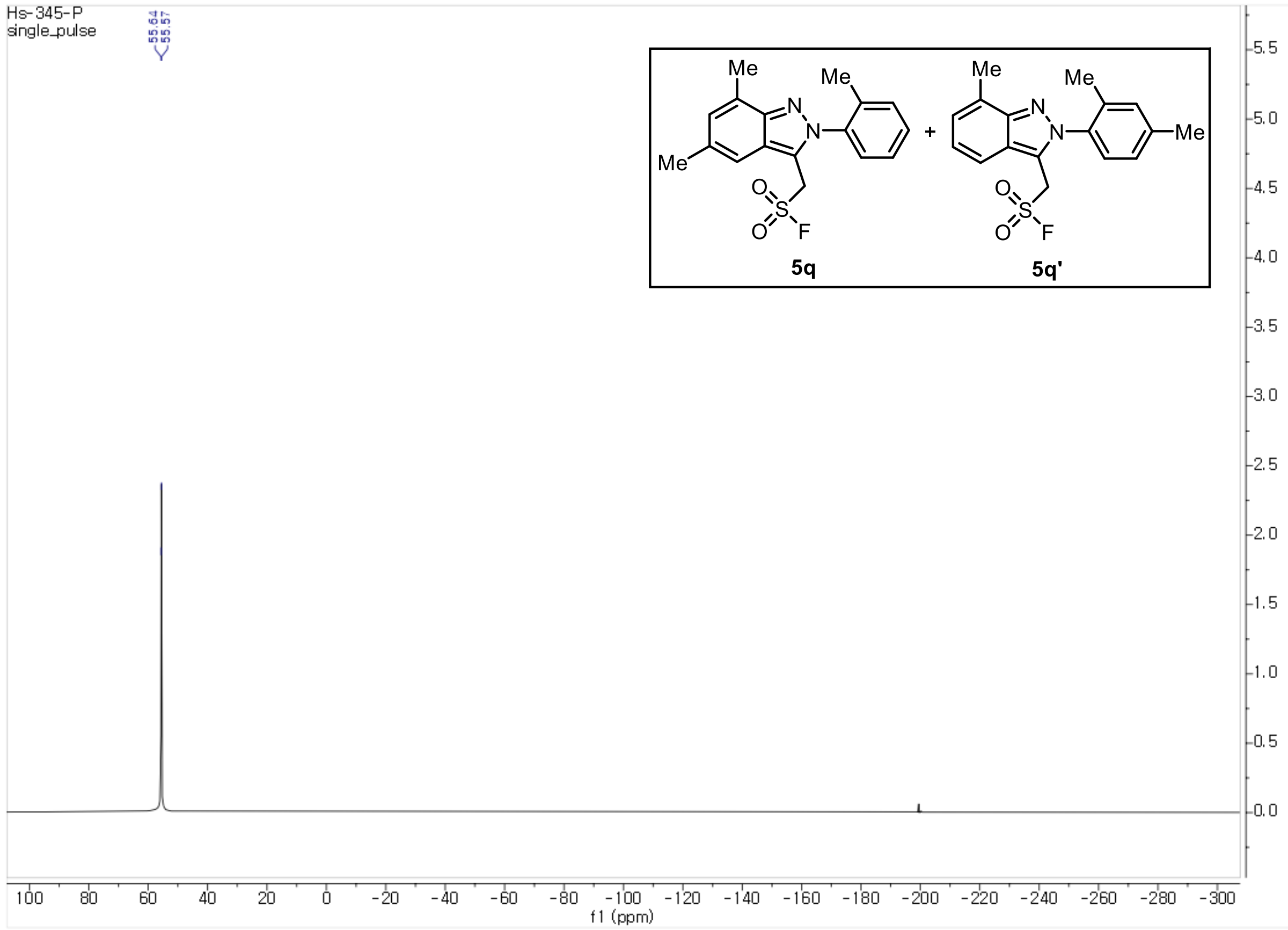
Hs-345-P
single_pulse
55.64
55.57
5q
5q'
f1 (ppm)

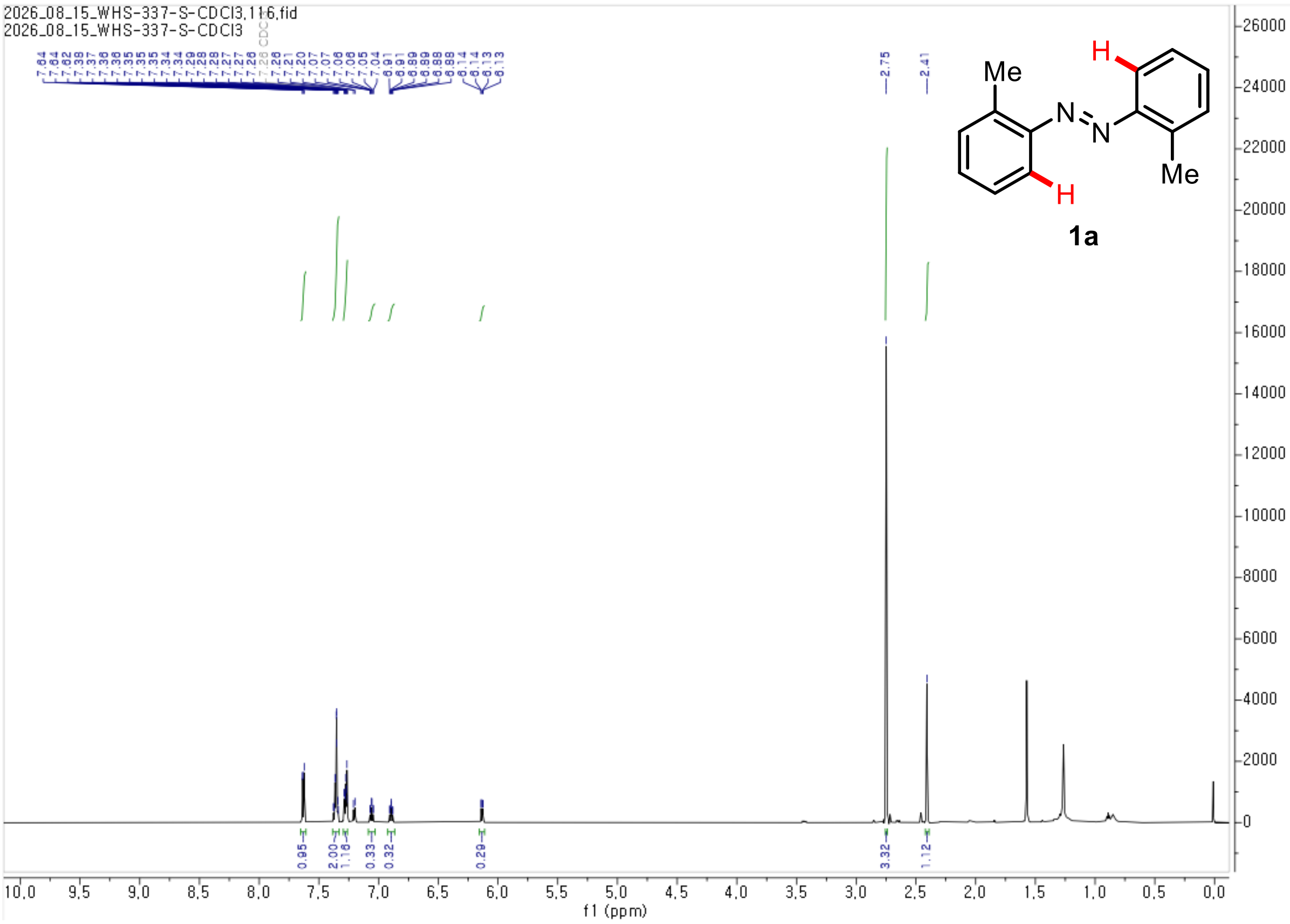
2026_08_15_WHS-337-S-CDCl3,1,fid
2026_08_15_WHS-337-S-CDCl3
Me
H
N
N
H
Me
1a
f1 (ppm)

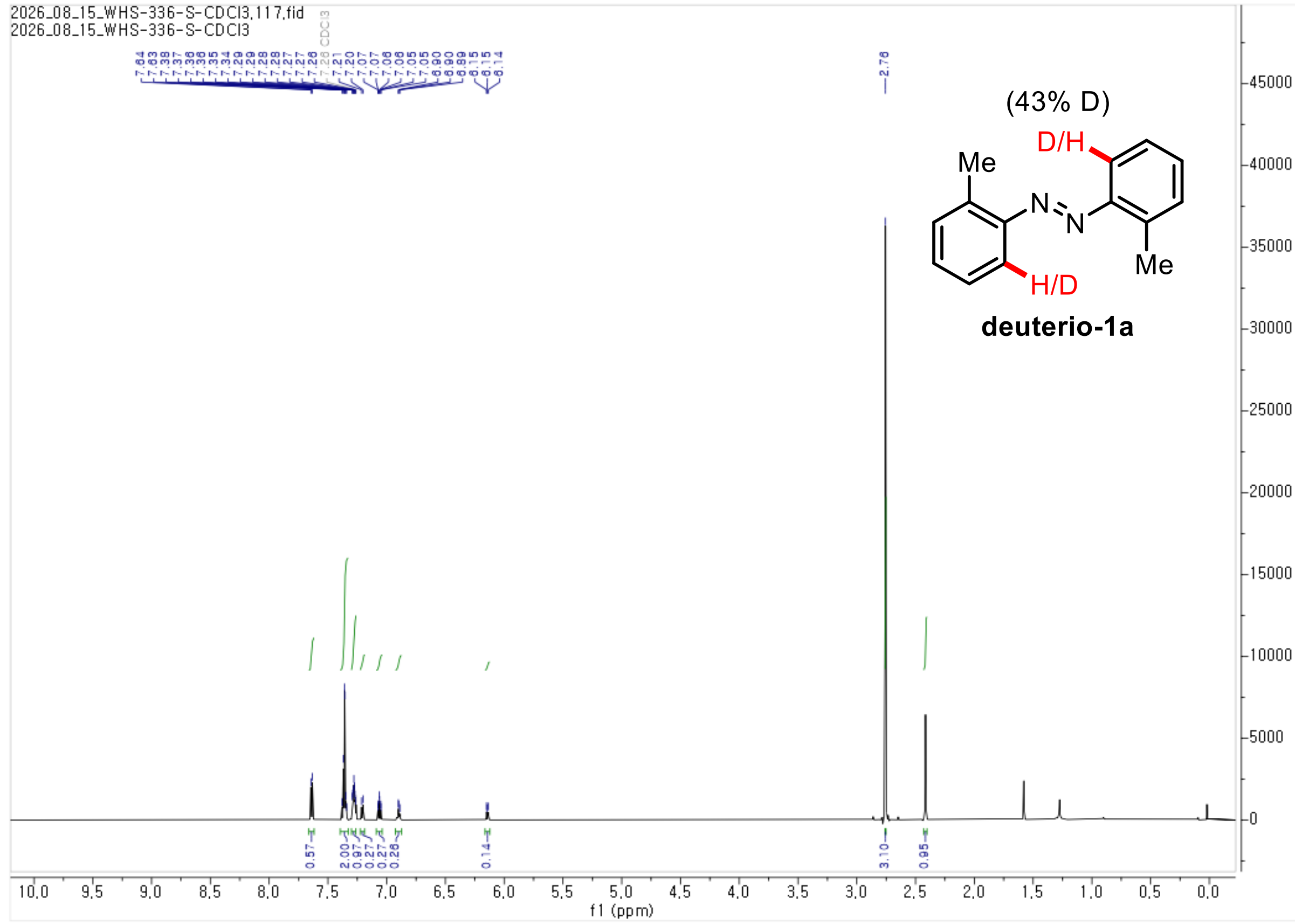
2026_08_15_WHS-336-S-CDCl3.117.fid
2026_08_15_WHS-336-S-CDCl3
(43% D)
D/H
Me
N
N
H/D
Me
deuterio-1a
f1 (ppm)

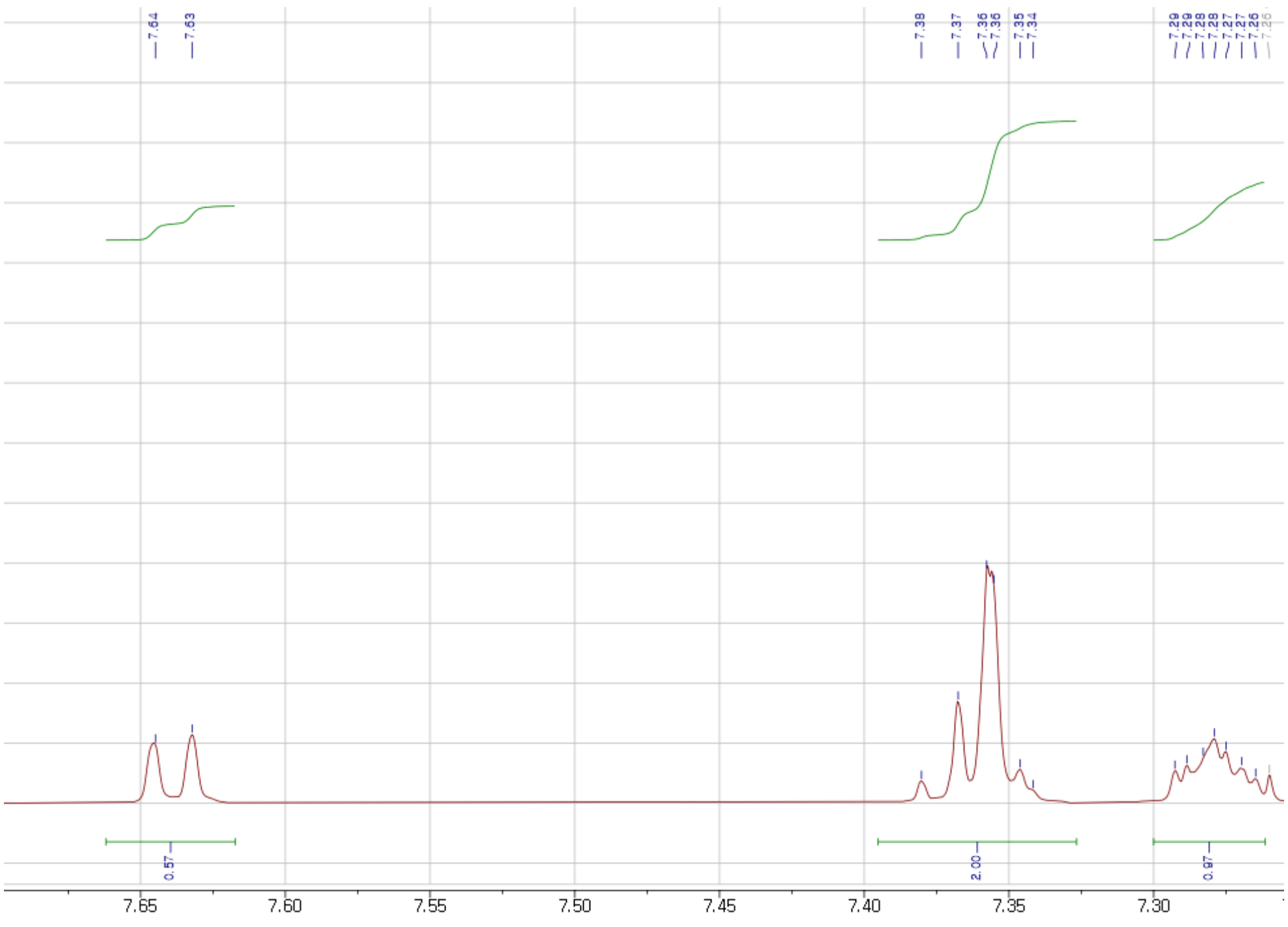

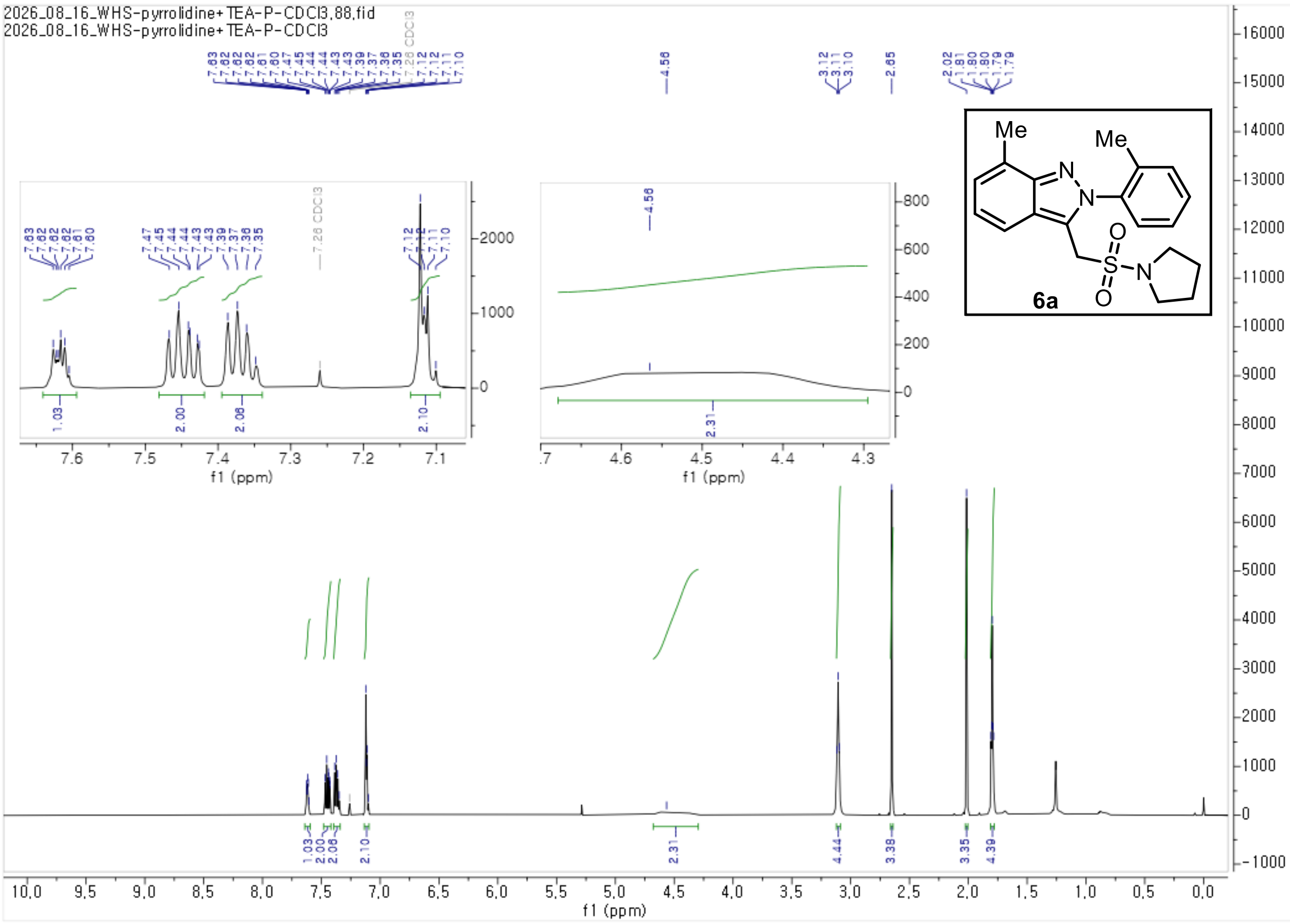
2026_08_16_WHS-pyrrolidine+TEA-P-CDCl3.88.fid
2026_08_16_WHS-pyrrolidine+TEA-P-CDCl3
Me
Me
N
N
O
S
N
O
6a
f1 (ppm)

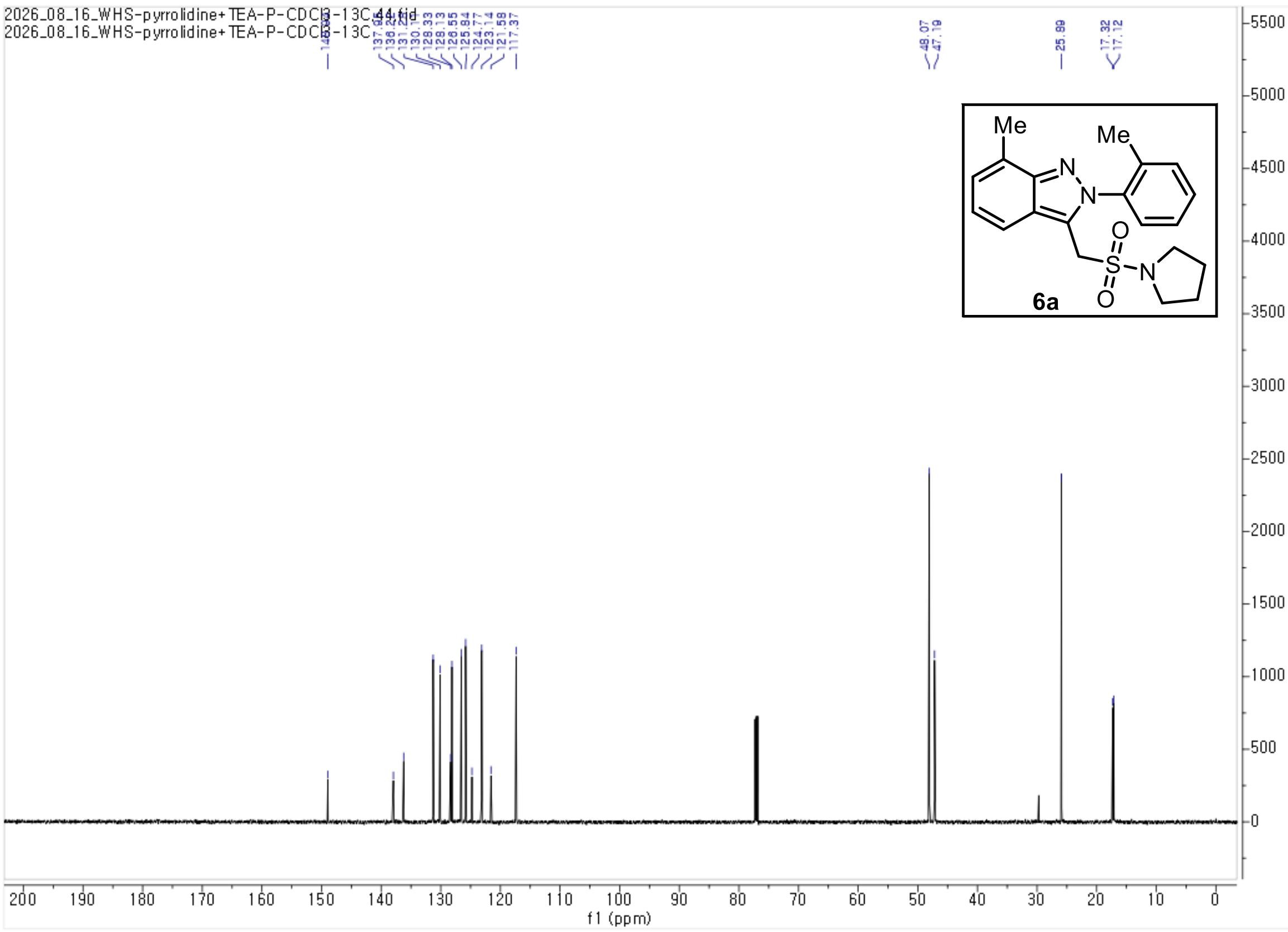
2026_08_16_WHS-pyrrolidine+TEA-P-CDCl3-13C.44.fid
2026_08_16_WHS-pyrrolidine+TEA-P-CDCl3-13C
Me
Me
N
N
O
S
N
O
6a
f1 (ppm)

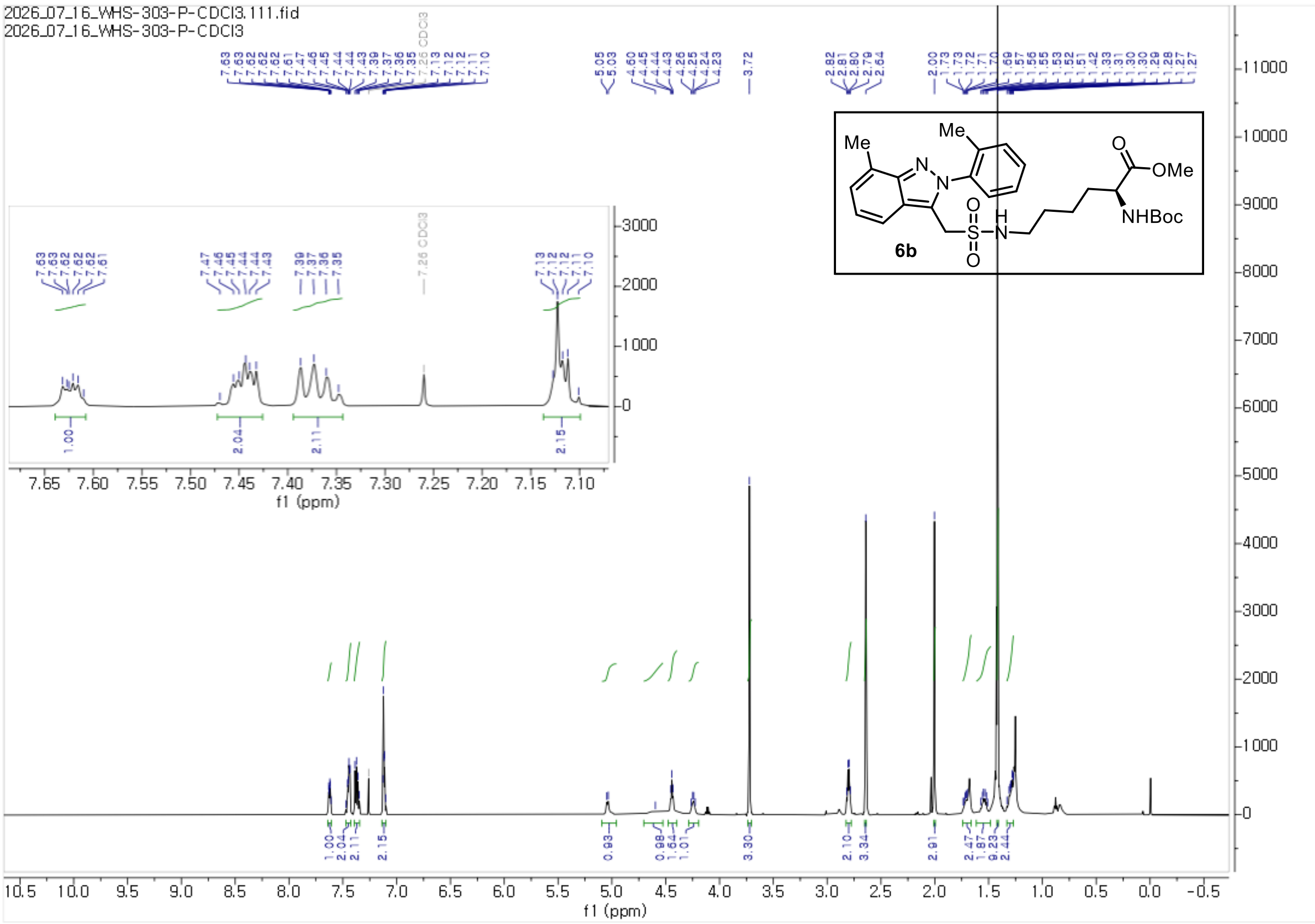
2026_07_16_WHS-303-P-CDCl3.111.fid
2026_07_16_WHS-303-P-CDCl3
6b
f1 (ppm)

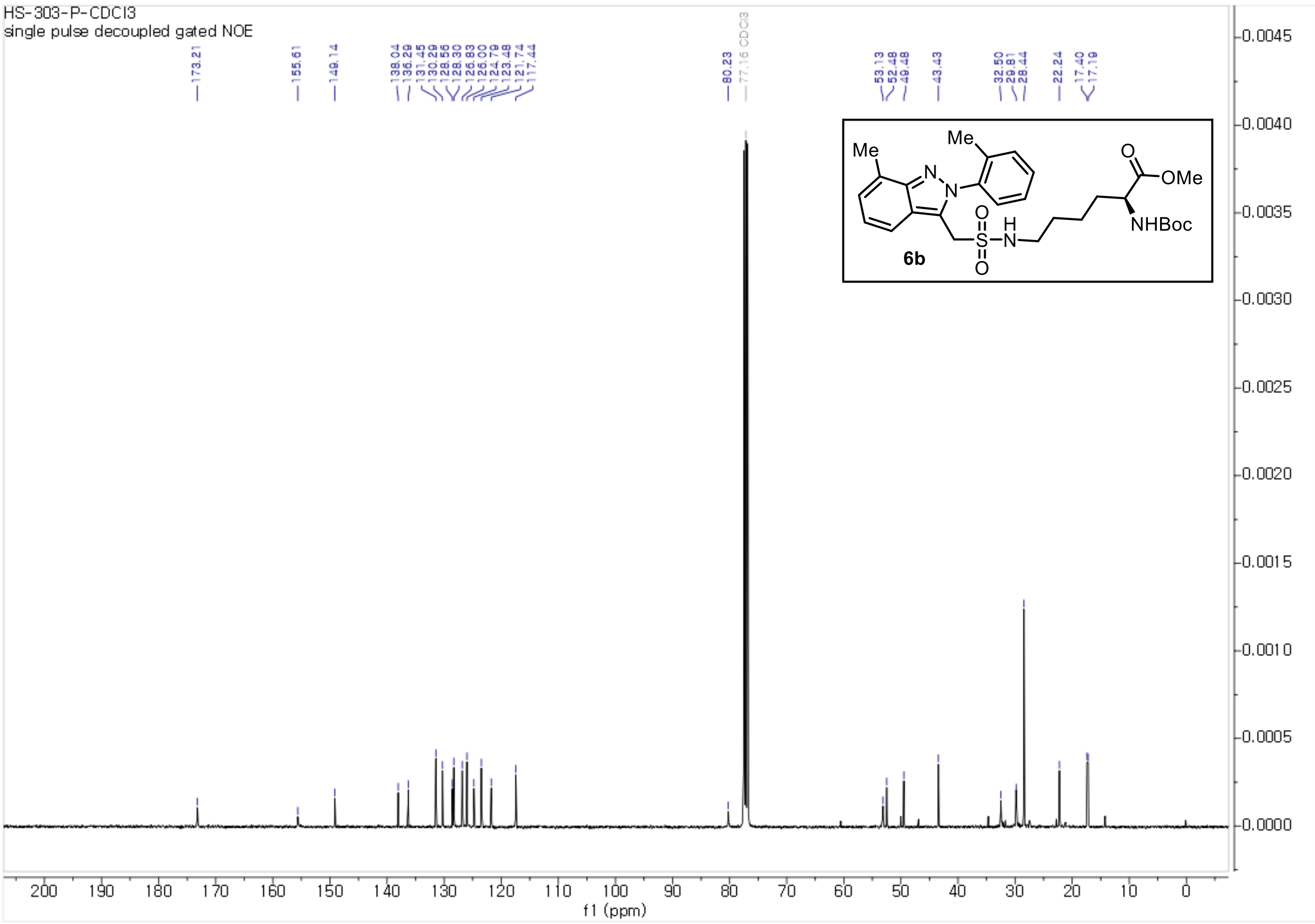
HS-303-P-CDCl3
single pulse decoupled gated NOE
6b
f1 (ppm)

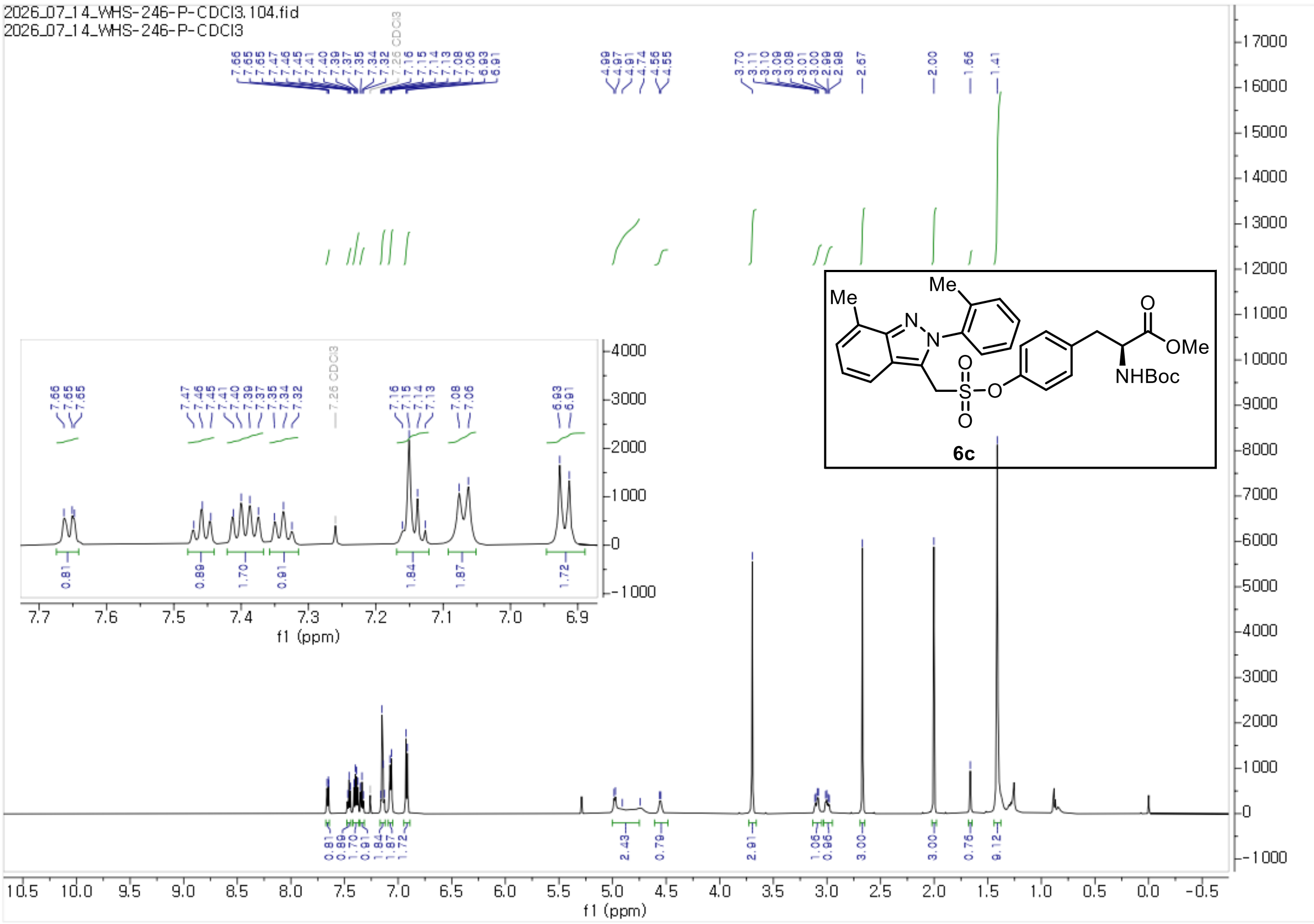
2026_07_14_WHS-246-P-CDCl3.104.fid
2026_07_14_WHS-246-P-CDCl3
f1 (ppm)
6c

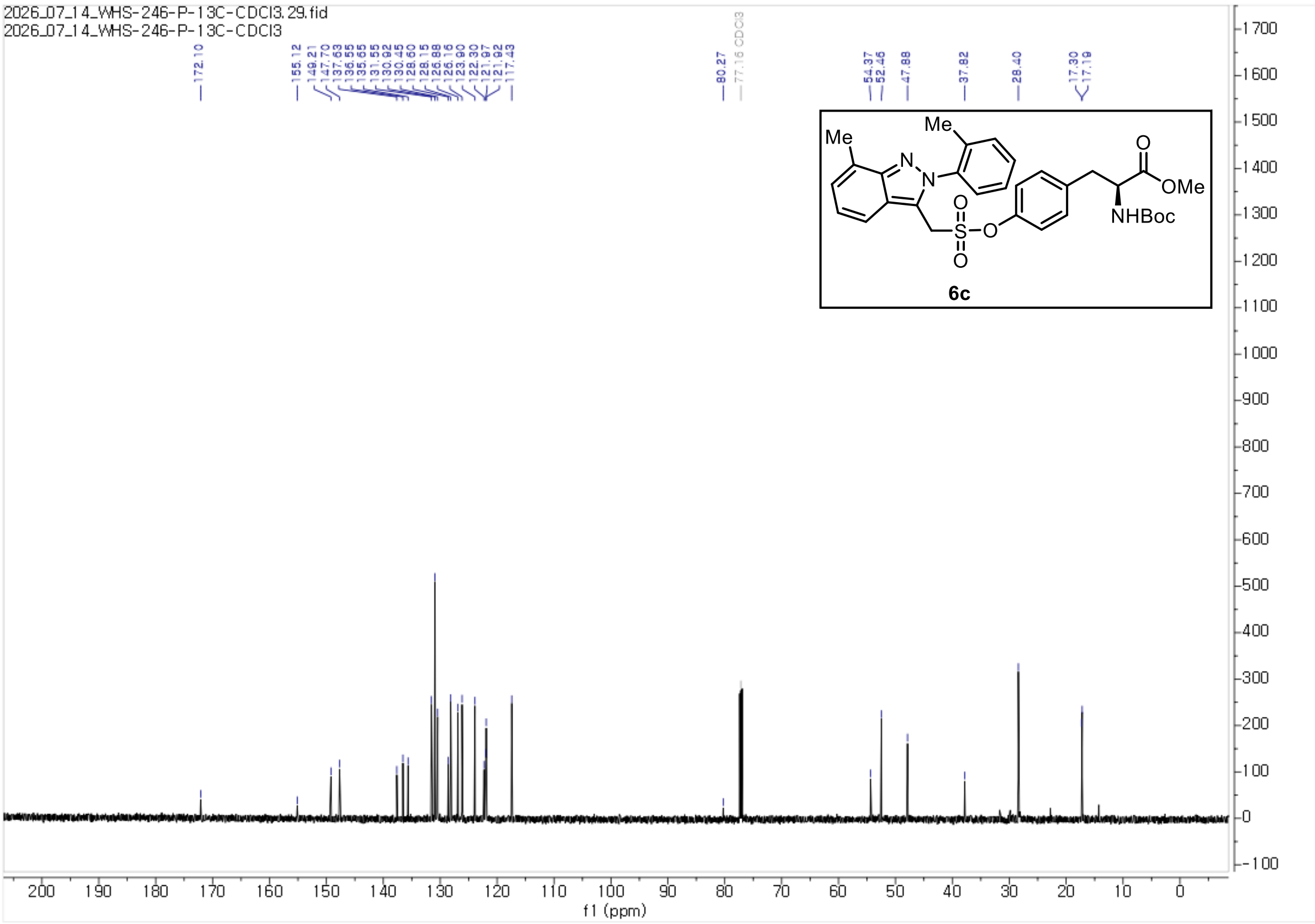
2026_07_14_WHS-246-P-13C-CDCl3.29.fid
2026_07_14_WHS-246-P-13C-CDCl3
f1 (ppm)
6c

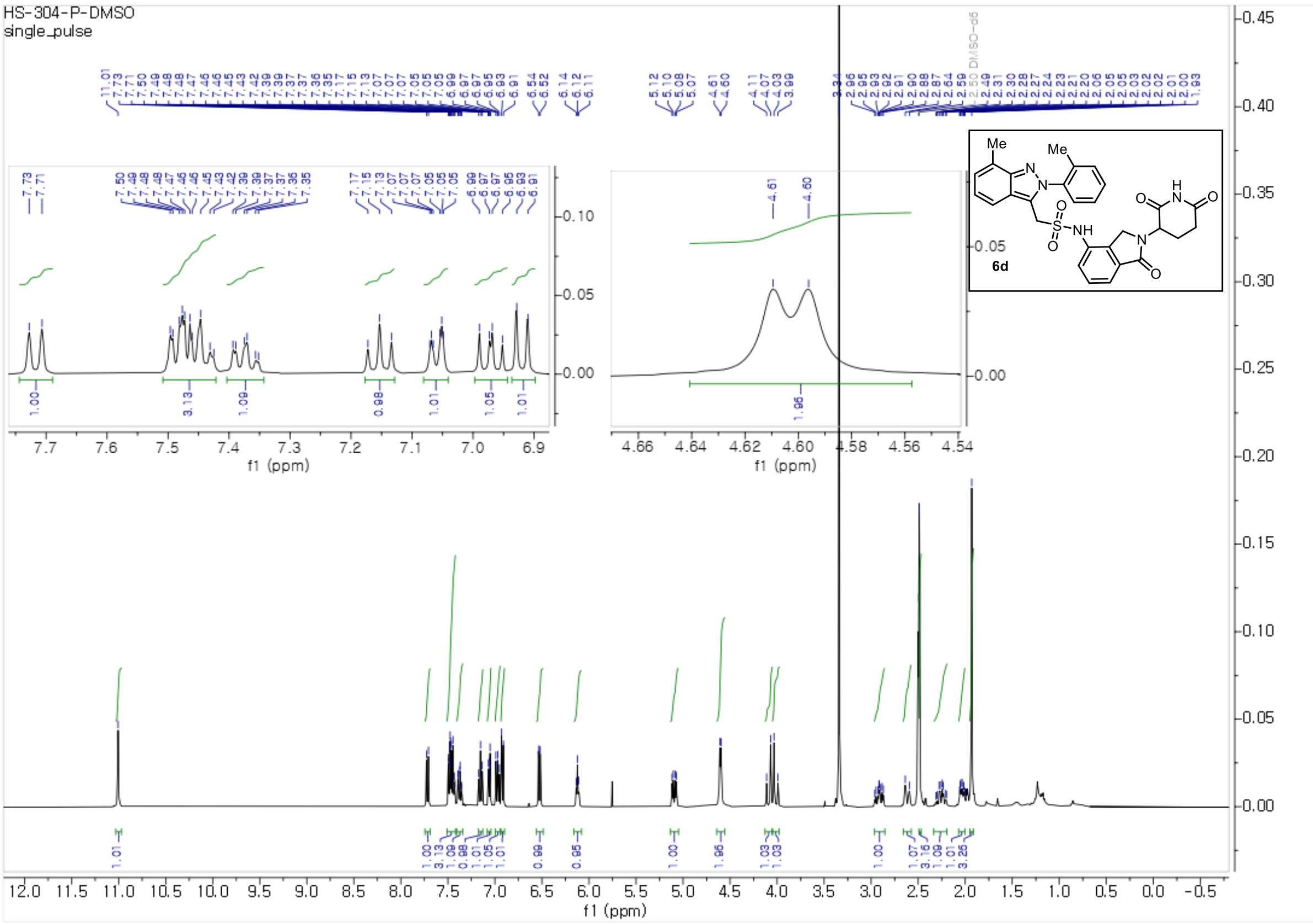

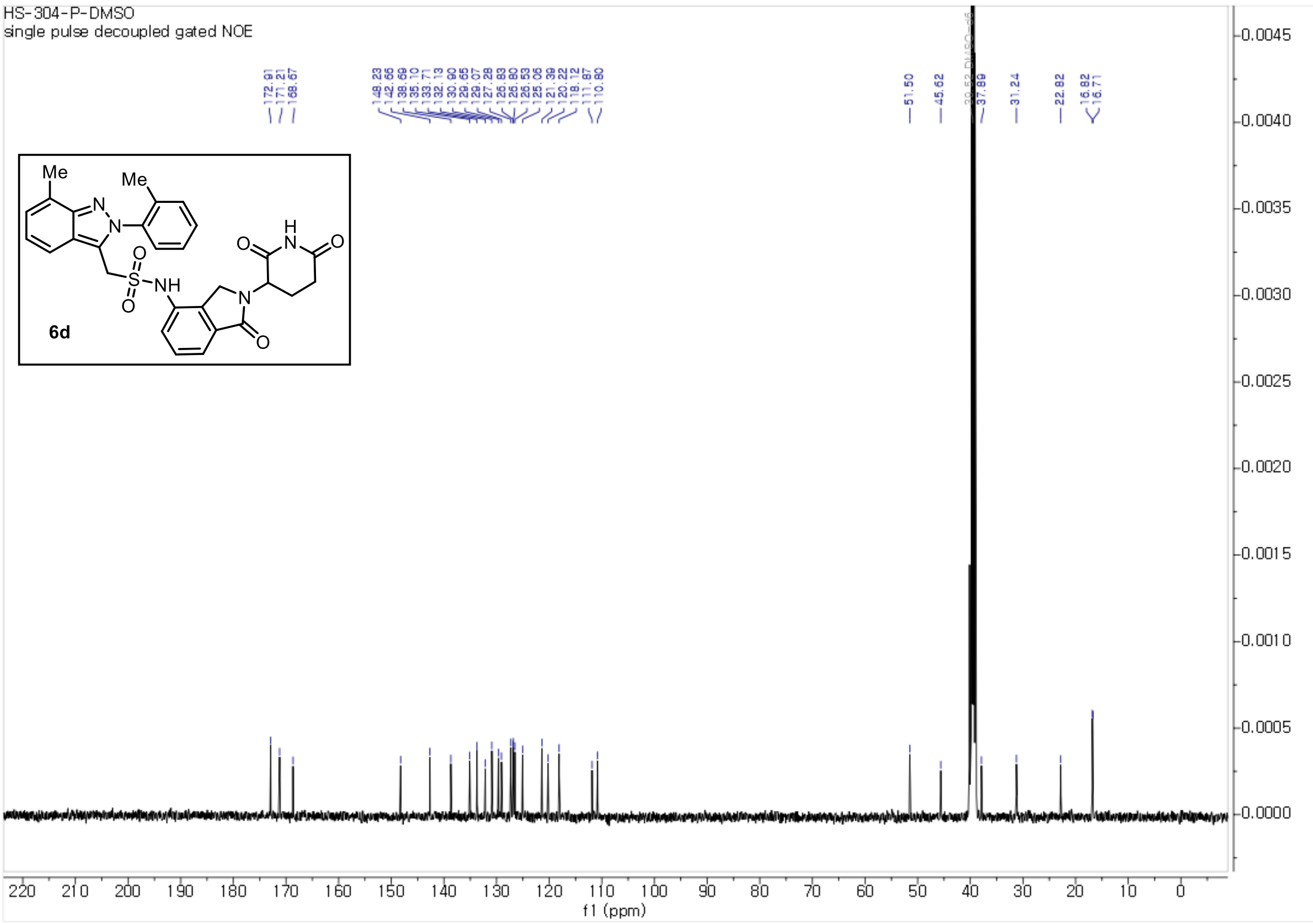

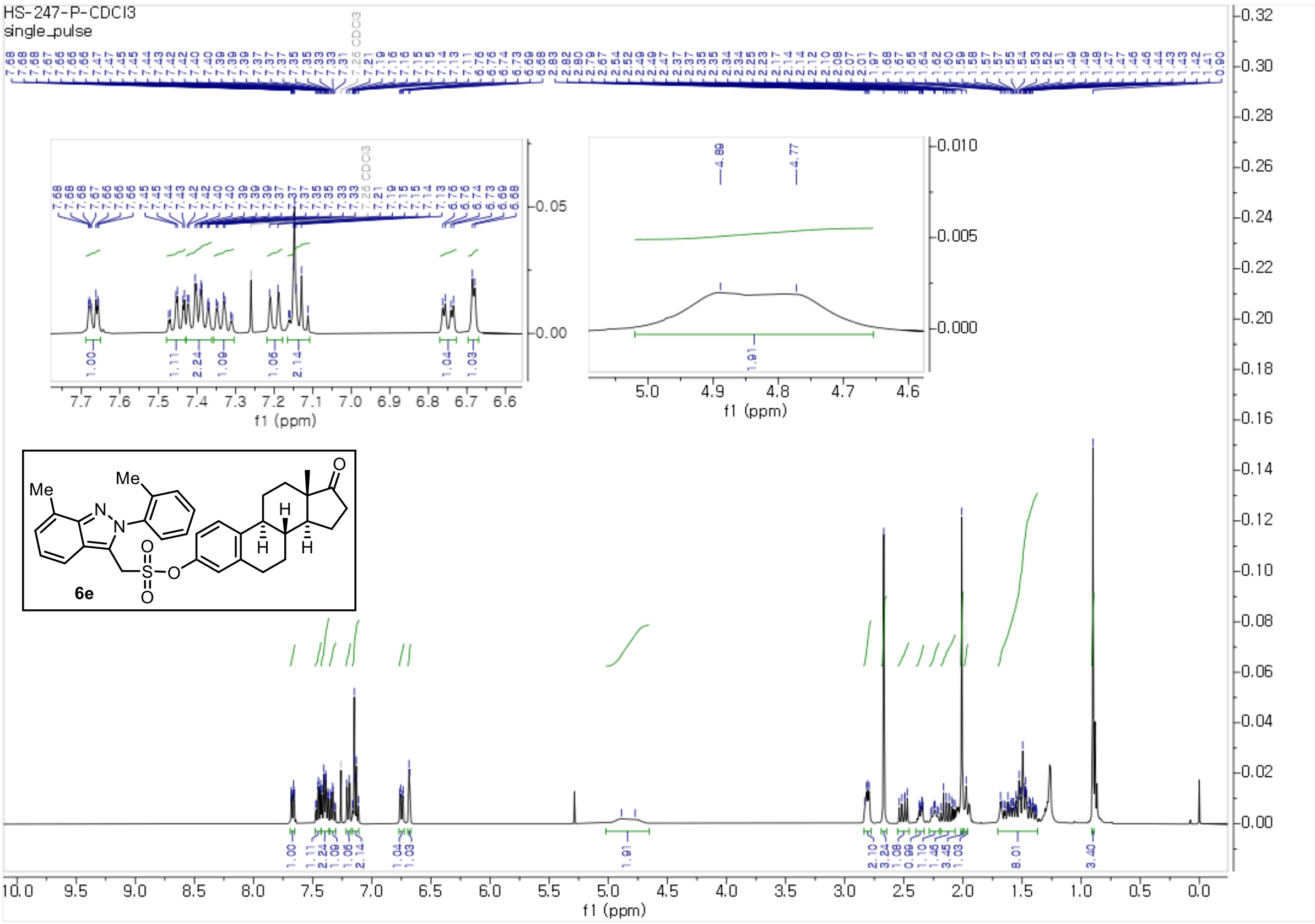

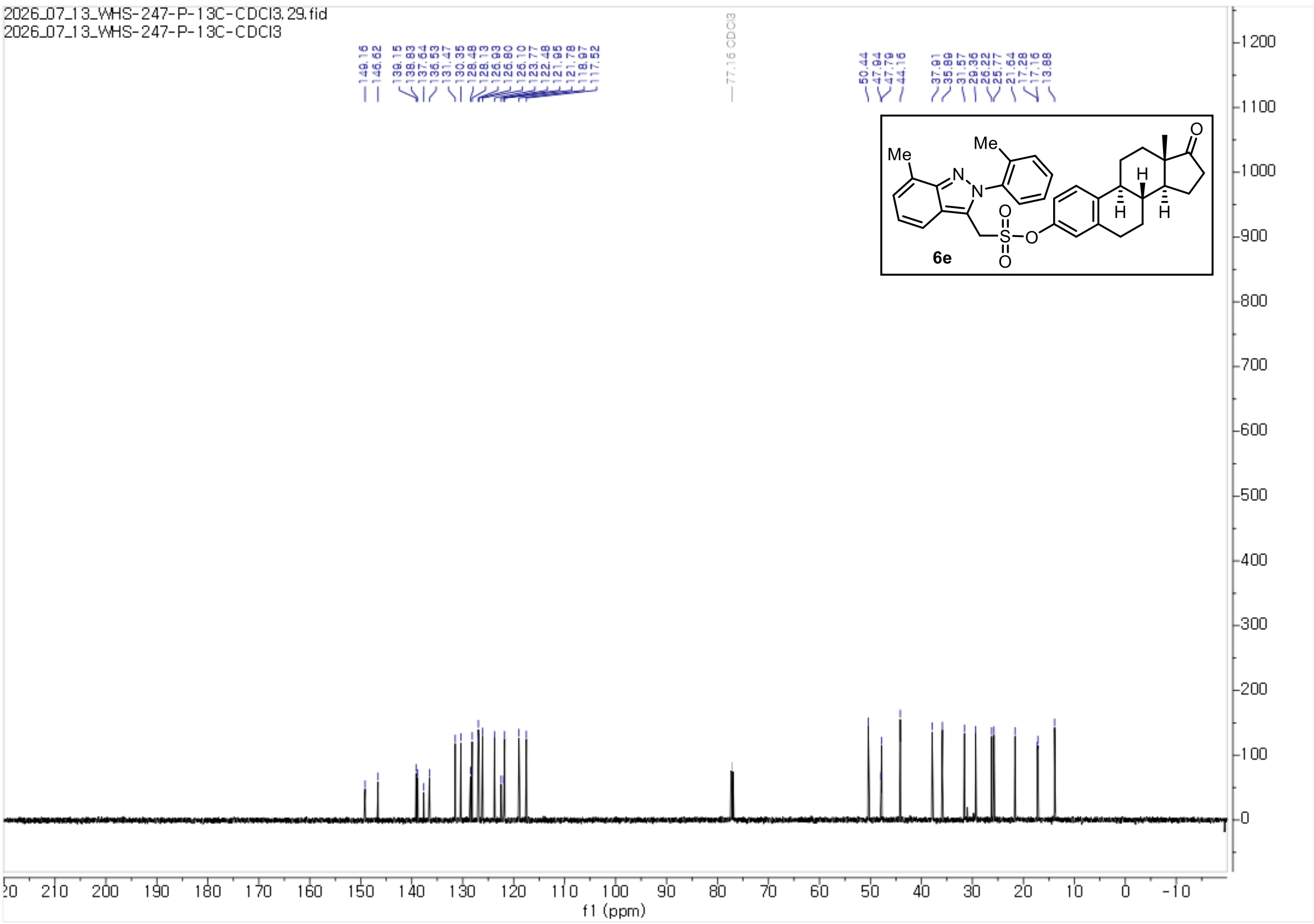

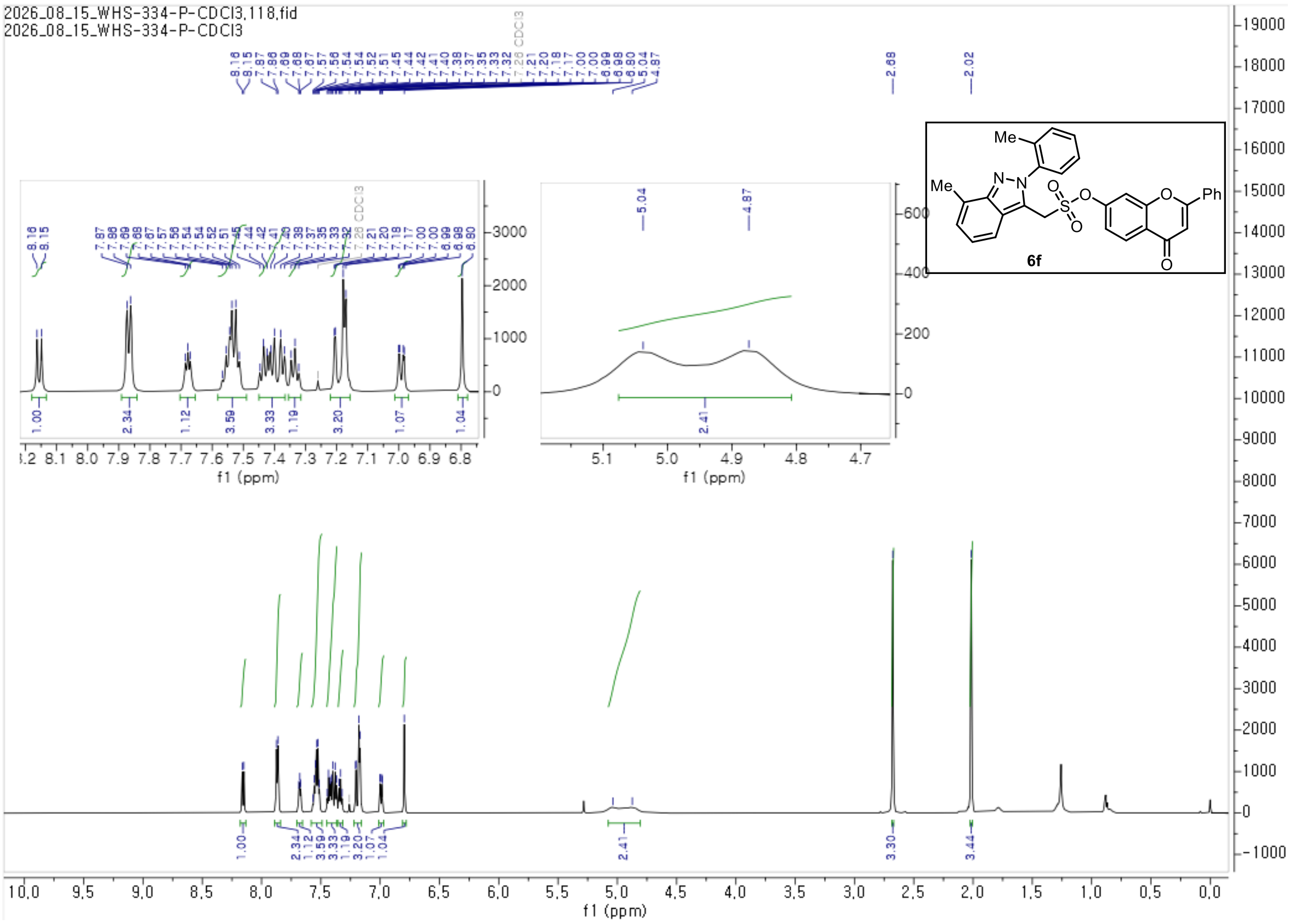
2026_08_15_WHS-334-P-CDCl3,118,fid
2026_08_15_WHS-334-P-CDCl3
6f
f1 (ppm)

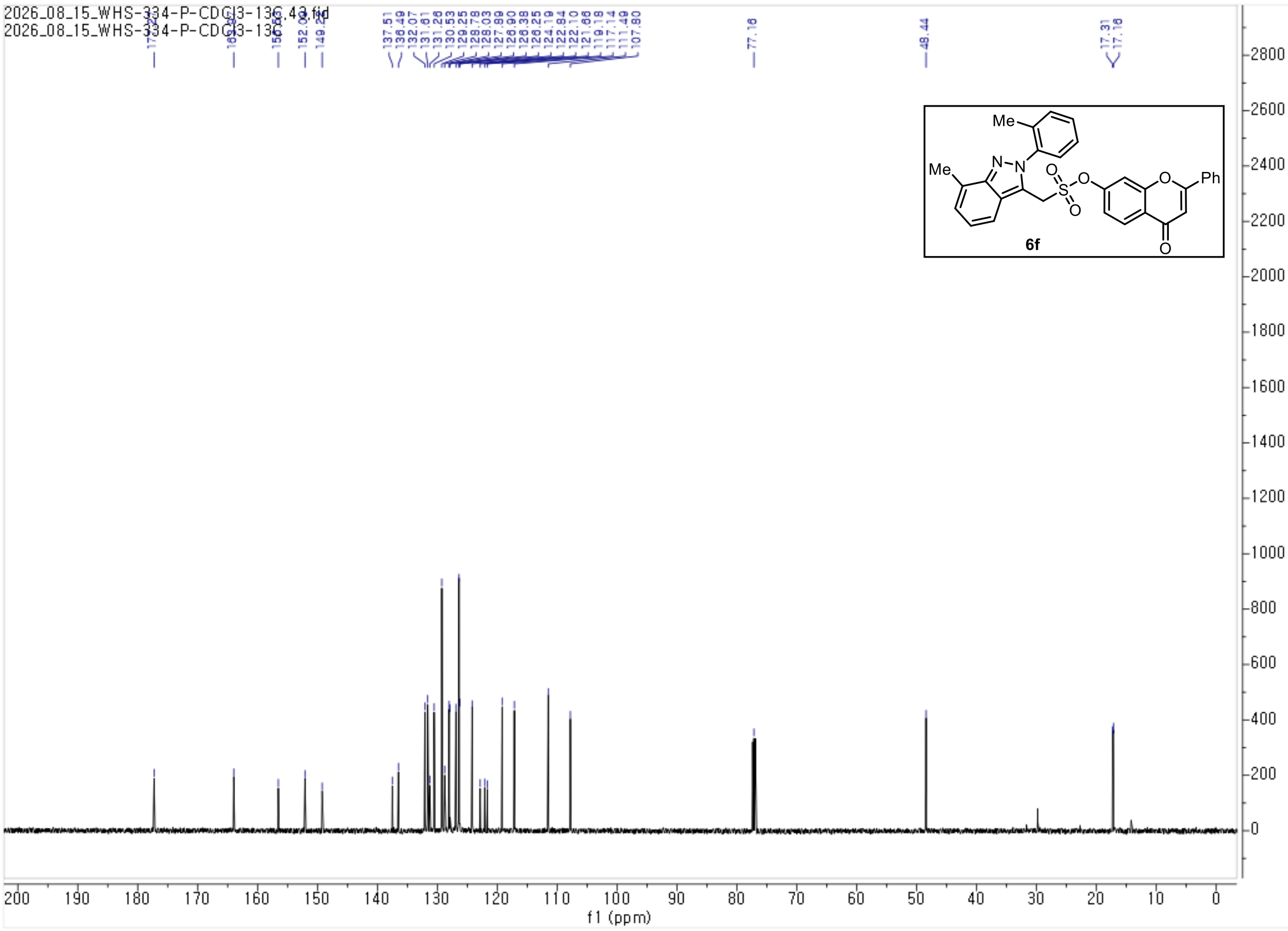
2026_08_15_WHS-334-P-CDCl3-13C,43,fid
2026_08_15_WHS-334-P-CDCl3-13C
6f
f1 (ppm)

9. HR-MS analytical data of the products

Compound 3a

```
[ Elemental Composition ]                                          Page: 1
Data   : EI-1-HS-209-002                  Date : 03-Jul-2026 15:23
Sample: High resolution GC Mass (JMS-700)
Note : SMLab-Mass Analysis & Research Center
Inlet : Direct                            Ion Mode : EI+
RT : 0.29 min                             Scan#: (9,10)+12
Elements : C 17/0, H 16/0, F 1/0, N 2/0, O 3/0, S 2/0
Mass Tolerance      : 1000ppm, 3mmu if m/z < 3, 5mmu if m/z > 5
Unsaturation (U.S.) : -0.5 - 80.0

Observed m/z Int%   Err[ppm / mmu]    U.S.  Composition
  318.0834   100.0     -1.4 /  -0.5   11.0  C 16 H 15 F N 2 O 2 S
```

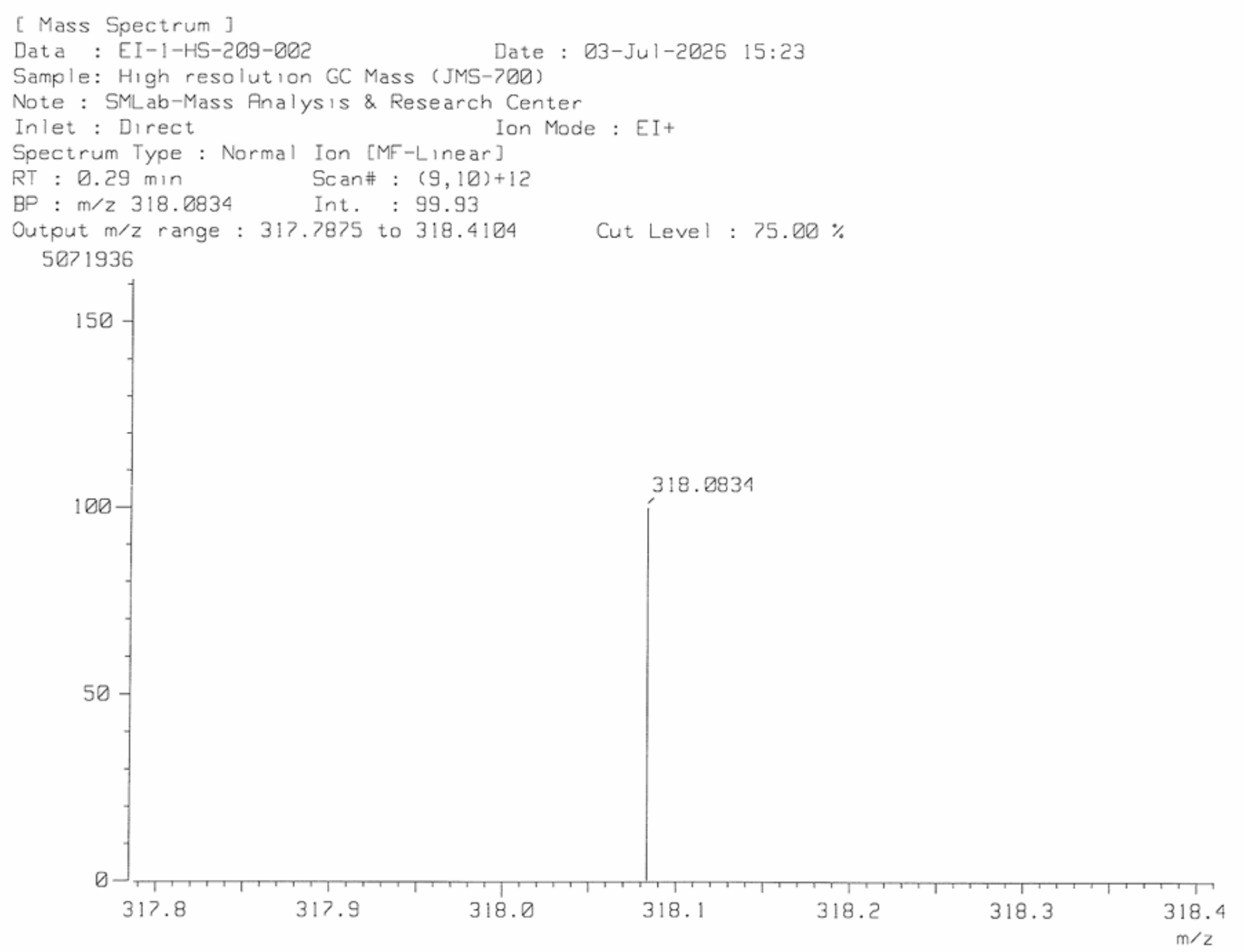

Compound 3b

[Elemental Composition] Page: 1
Data : EI-HS-214-002 Date : 08-Jul-2026 17:54
Sample: High resolution GC Mass (JMS-700)
Note : SMLab-Mass Analysis & Research Center
Inlet : Direct Ion Mode : EI+
RT : 0.47 min Scan#: 15
Elements : C 20/0, H 20/0, F 1/0, N 4/0, O 2/0, S 1/0
Mass Tolerance : 1000ppm, 3mmu if m/z < 3, 5mmu if m/z > 5
Unsaturation (U.S.) : -0.5 - 80.0

Observed m/z	Int%	Err[ppm / mmu]	U.S.	Composition
346.1146	100.0	-1.5 / -0.5	11.0	C 18 H 19 F N 2 O 2 S

[Mass Spectrum]
Data : EI-HS-214-002 Date : 08-Jul-2026 17:54
Sample: High resolution GC Mass (JMS-700)
Note : SMLab-Mass Analysis & Research Center
Inlet : Direct Ion Mode : EI+
Spectrum Type : Normal Ion [MF-Linear]
RT : 0.47 min Scan# : 15
BP : m/z 346.1146 Int. : 50.05
Output m/z range : 345.8036 to 346.4200 Cut Level : 73.53 %

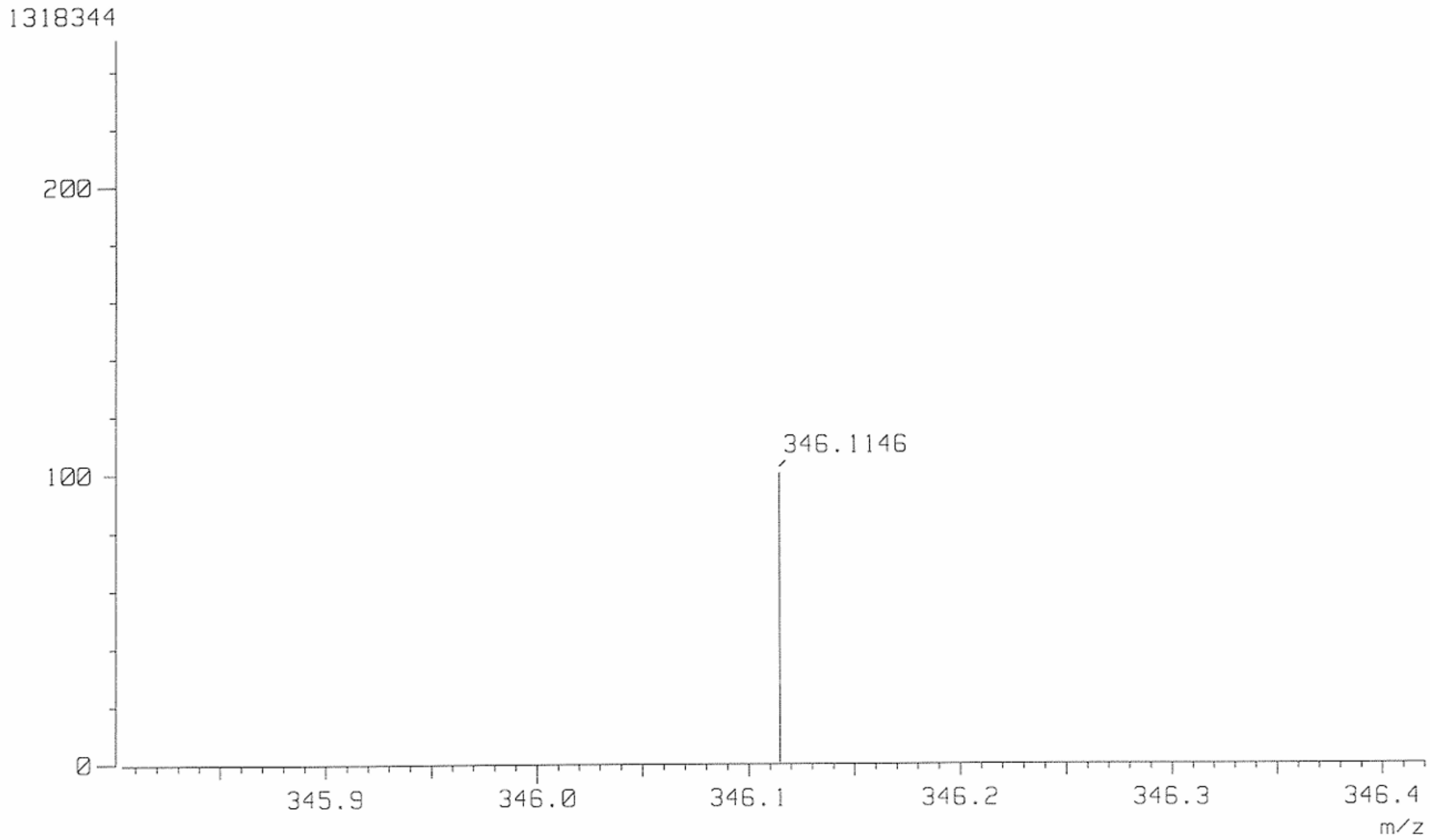

Compound 3c

[Elemental Composition]
Data : EI-28-Code-LR-38-F3-019   Date : 04-Jul-2026 09:57   Page: 1
Sample: High resolution GC Mass (JMS-700)
Note : SMLab-Mass Analysis & Research Center
Inlet : Direct   Ion Mode : EI+
RT : 0.60 min   Scan#: 19
Elements : C 15/0, H 14/0, F 3/0, N 2/0, O 3/0, S 1/0
Mass Tolerance : 1000ppm, 3mmu if m/z < 3, 5mmu if m/z > 5
Unsaturation (U.S.) : -0.5 - 80.0

Observed m/z	Int%	Err[ppm / mmu]	U.S.	Composition
326.0336	100.0	-0.4 / -0.1	11.0	C 14 H 9 F 3 N 2 O 2 S

[Mass Spectrum]
Data : EI-28-Code-LR-38-F3-019   Date : 04-Jul-2026 09:57
Sample: High resolution GC Mass (JMS-700)
Note : SMLab-Mass Analysis & Research Center
Inlet : Direct   Ion Mode : EI+
Spectrum Type : Normal Ion [MF-Linear]
RT : 0.60 min   Scan# : 19
BP : m/z 326.0336   Int. : 35.99
Output m/z range : 325.7115 to 326.3140   Cut Level : 78.68 %

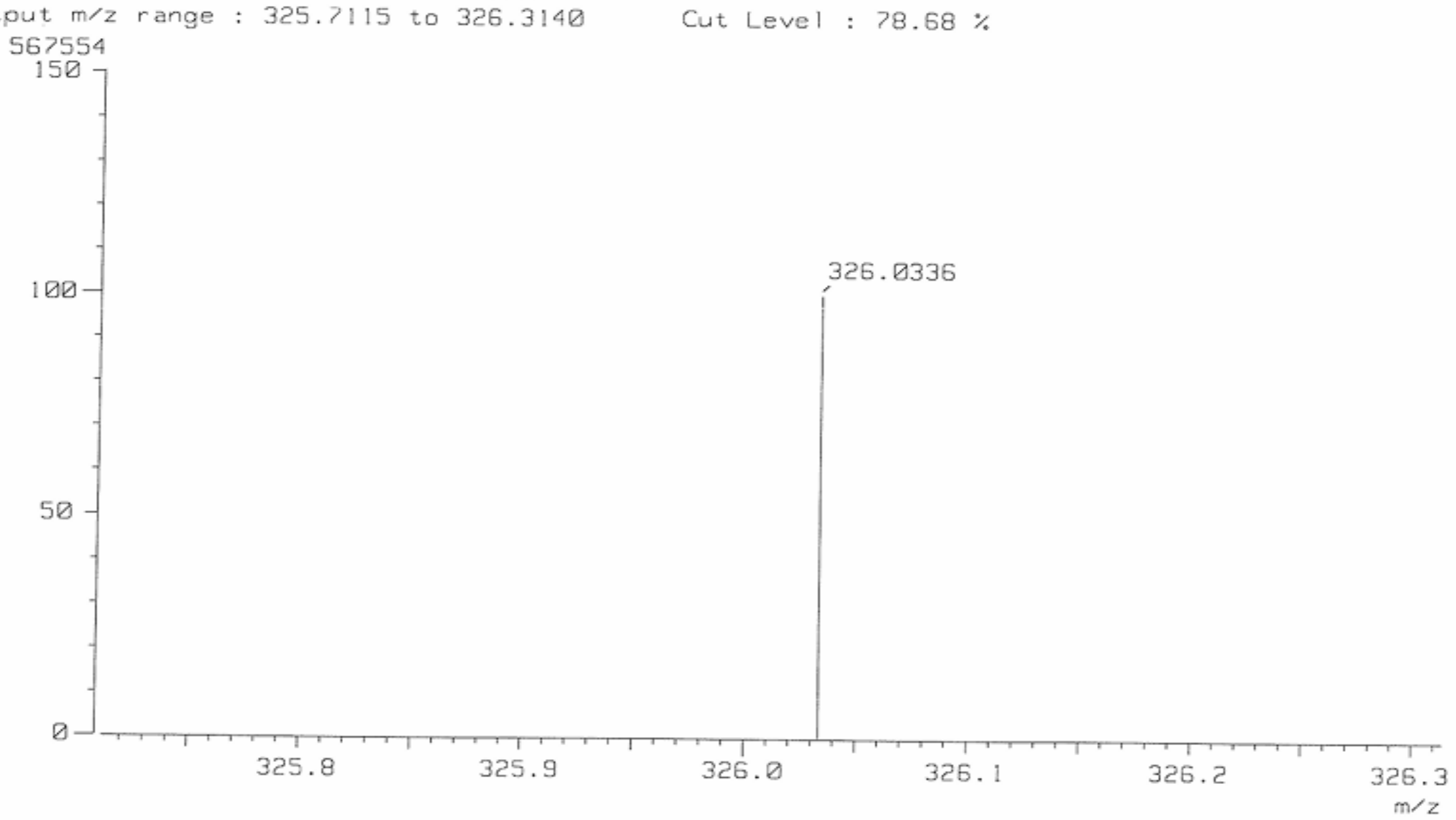

Compound 3d

```
[ Elemental Composition ]                                          Page: 1
Data   : EI-36-Code-LR-103-F3-005      Date : 04-Jul-2026 09:35
Sample: High resolution GC Mass (JMS-700)
Note : SMLab-Mass Analysis & Research Center
Inlet : Direct                          Ion Mode : EI+
RT : 1.17 min                           Scan#: (28,44)
Elements : C 15/0, H 10/0, Cl 2/0, F 1/0, N 2/0, O 3/0, S 1/0
Mass Tolerance        : 1000ppm, 3mmu if m/z < 3, 5mmu if m/z > 5
Unsaturation (U.S.) : -0.5 - 80.0

Observed m/z Int%   Err[ppm / mmu]    U.S.  Composition
  357.9742   100.0     -0.9 /  -0.3   11.0  C 14 H 9 Cl 2 F N 2 O 2 S
```

```
[ Mass Spectrum ]
Data   : EI-36-Code-LR-103-F3-005      Date : 04-Jul-2026 09:35
Sample: High resolution GC Mass (JMS-700)
Note : SMLab-Mass Analysis & Research Center
Inlet : Direct                          Ion Mode : EI+
Spectrum Type : Normal Ion [MF-Linear]
RT : 1.17 min          Scan# : (28,44)
BP : m/z 357.9742      Int.  : 8.45
Output m/z range : 357.6682 to 358.2556      Cut Level : 47.84 %
```

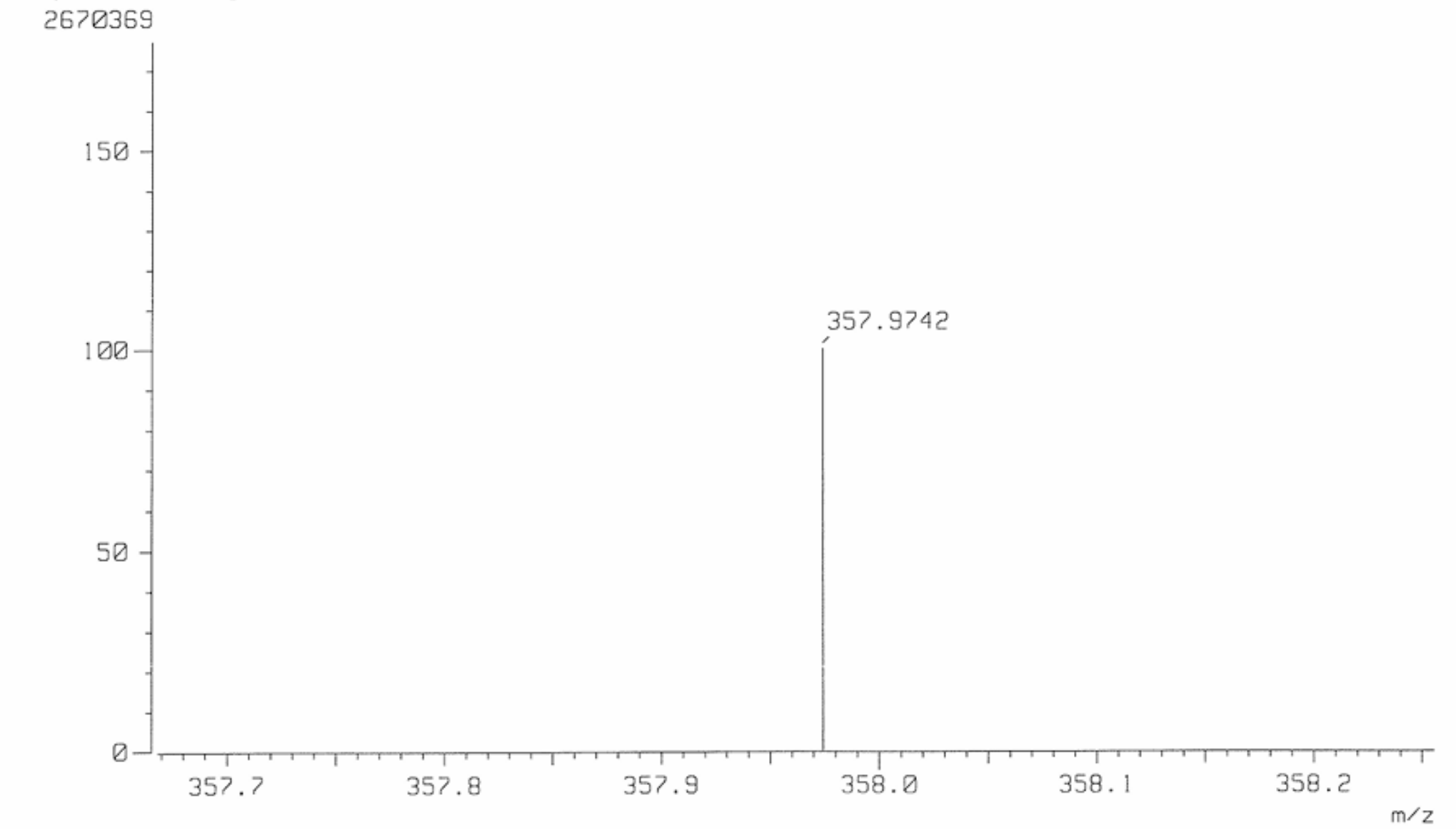

Compound 3e

[Elemental Composition]　　Page: 1

Data : EI-12-HS-250-025　　Date : 03-Jul-2026 16:04
Sample: High resolution GC Mass (JMS-700)
Note : SMLab-Mass Analysis & Research Center
Inlet : Direct　　Ion Mode : EI+
RT : 2.10 min　　Scan#: 64
Elements : C 20/0, H 20/0, F 1/0, N 2/0, O 3/0, S 1/0
Mass Tolerance : 1000ppm, 3mmu if m/z < 3, 5mmu if m/z > 5
Unsaturation (U.S.) : -0.5 - 80.0

Observed m/z	Int%	Err[ppm / mmu]	U.S.	Composition
346.1152	100.0	+0.1 / +0.0	11.0	C 18 H 19 F N 2 O 2 S

[Mass Spectrum]
Data : EI-12-HS-250-025　　Date : 03-Jul-2026 16:04
Sample: High resolution GC Mass (JMS-700)
Note : SMLab-Mass Analysis & Research Center
Inlet : Direct　　Ion Mode : EI+
Spectrum Type : Normal Ion [MF-Linear]
RT : 2.10 min　　Scan# : 64
BP : m/z 346.1152　　Int. : 71.61
Output m/z range : 345.7537 to 346.4530　　Cut Level : 71.32 %

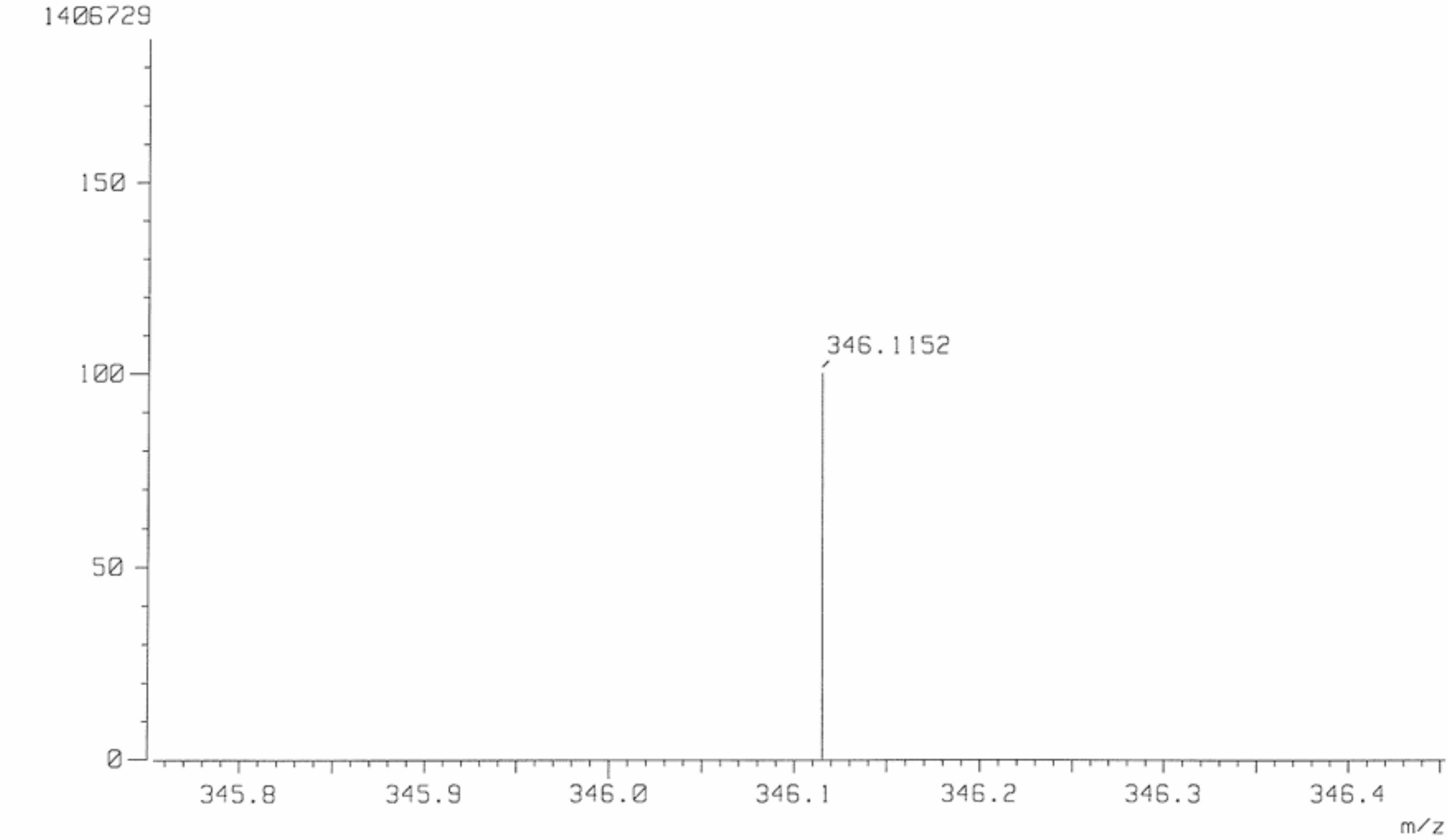

Compound 3f

[Elemental Composition] Page: 1
Data : EI-5-HS-220-008 Date : 03-Jul-2026 15:34
Sample: High resolution GC Mass (JMS-700)
Note : SMLab-Mass Analysis & Research Center
Inlet : Direct Ion Mode : EI+
RT : 1.04 min Scan#: (31,33)
Elements : C 20/0, H 25/0, F 1/0, N 2/0, O 2/0, S 1/0
Mass Tolerance : 1000ppm, 3mmu if m/z < 3, 5mmu if m/z > 5
Unsaturation (U.S.) : -0.5 - 80.0

Observed m/z	Int%	Err [ppm / mmu]	U.S.	Composition
374.1465	23.8	+0.2 / +0.1	11.0	C 20 H 23 F N 2 O 2 S

[Mass Spectrum]
Data : EI-5-HS-220-008 Date : 03-Jul-2026 15:34
Sample: High resolution GC Mass (JMS-700)
Note : SMLab-Mass Analysis & Research Center
Inlet : Direct Ion Mode : EI+
Spectrum Type : Normal Ion [MF-Linear]
RT : 1.04 min Scan# : (31,33)
BP : m/z 317.9681 Int. : 96.88
Output m/z range : 373.8229 to 374.4768 Cut Level : 12.50 %

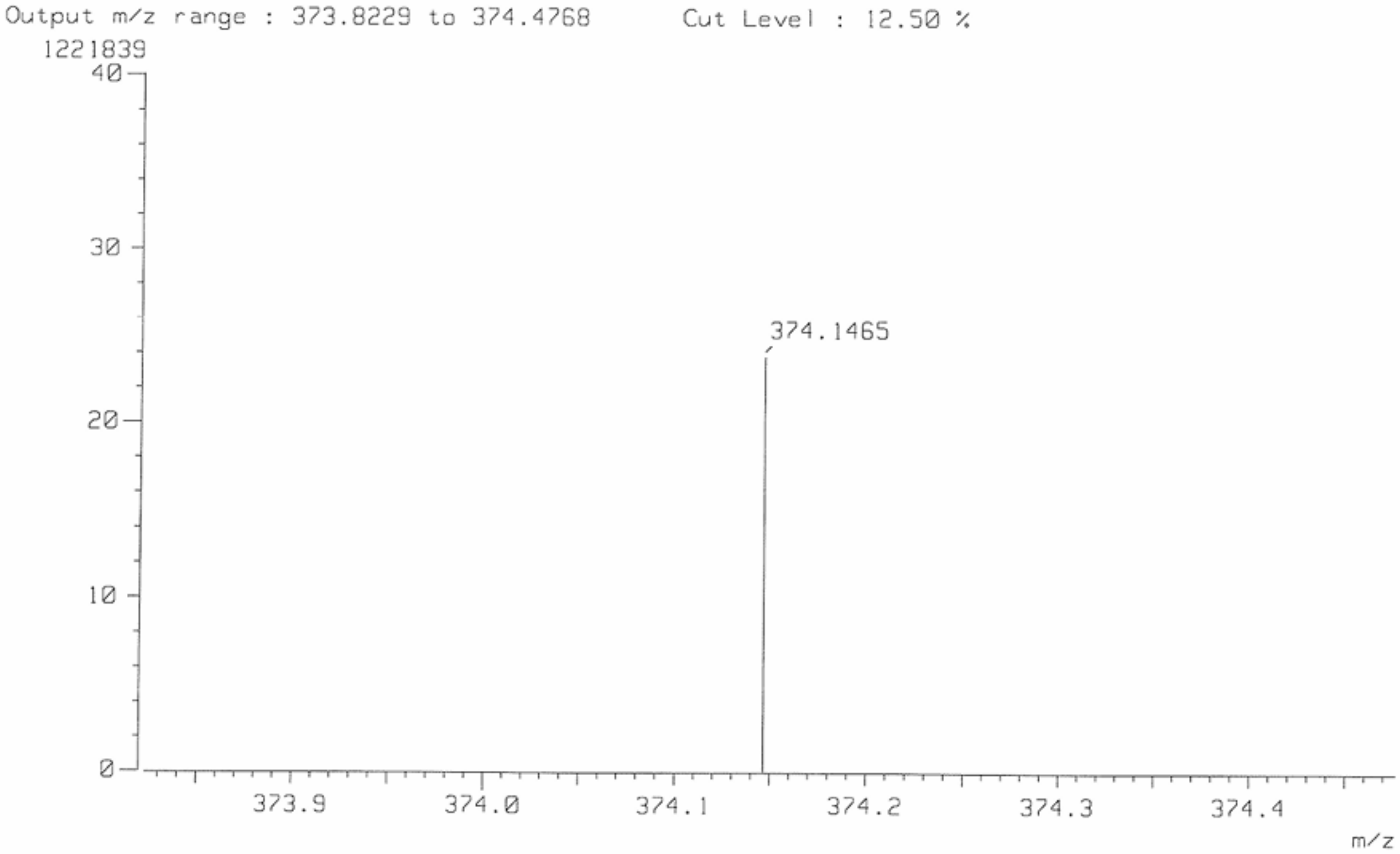

Compound 3g

[Elemental Composition]   Page: 1

Data : EI-39-Code-LR-112-F2-020   Date : 04-Jul-2026 10:00
Sample: High resolution GC Mass (JMS-700)
Note : SMLab-Mass Analysis & Research Center
Inlet : Direct   Ion Mode : EI+
RT : 1.57 min   Scan#: 48
Elements : C 14/0, H 14/0, F 3/0, N 2/0, O 3/0, S 1/0
Mass Tolerance : 1000ppm, 3mmu if m/z < 3, 5mmu if m/z > 5
Unsaturation (U.S.) : -0.5 - 80.0

Observed m/z	Int%	Err[ppm / mmu]	U.S.	Composition
326.0333	100.0	-1.2 / -0.4	11.0	C 14 H 9 F 3 N 2 O 2 S

[Mass Spectrum]
Data : EI-39-Code-LR-112-F2-020   Date : 04-Jul-2026 10:00
Sample: High resolution GC Mass (JMS-700)
Note : SMLab-Mass Analysis & Research Center
Inlet : Direct   Ion Mode : EI+
Spectrum Type : Normal Ion [MF-Linear]
RT : 1.57 min   Scan# : 48
BP : m/z 326.0333   Int. : 51.85
Output m/z range : 325.6868 to 326.3103   Cut Level : 69.10 %

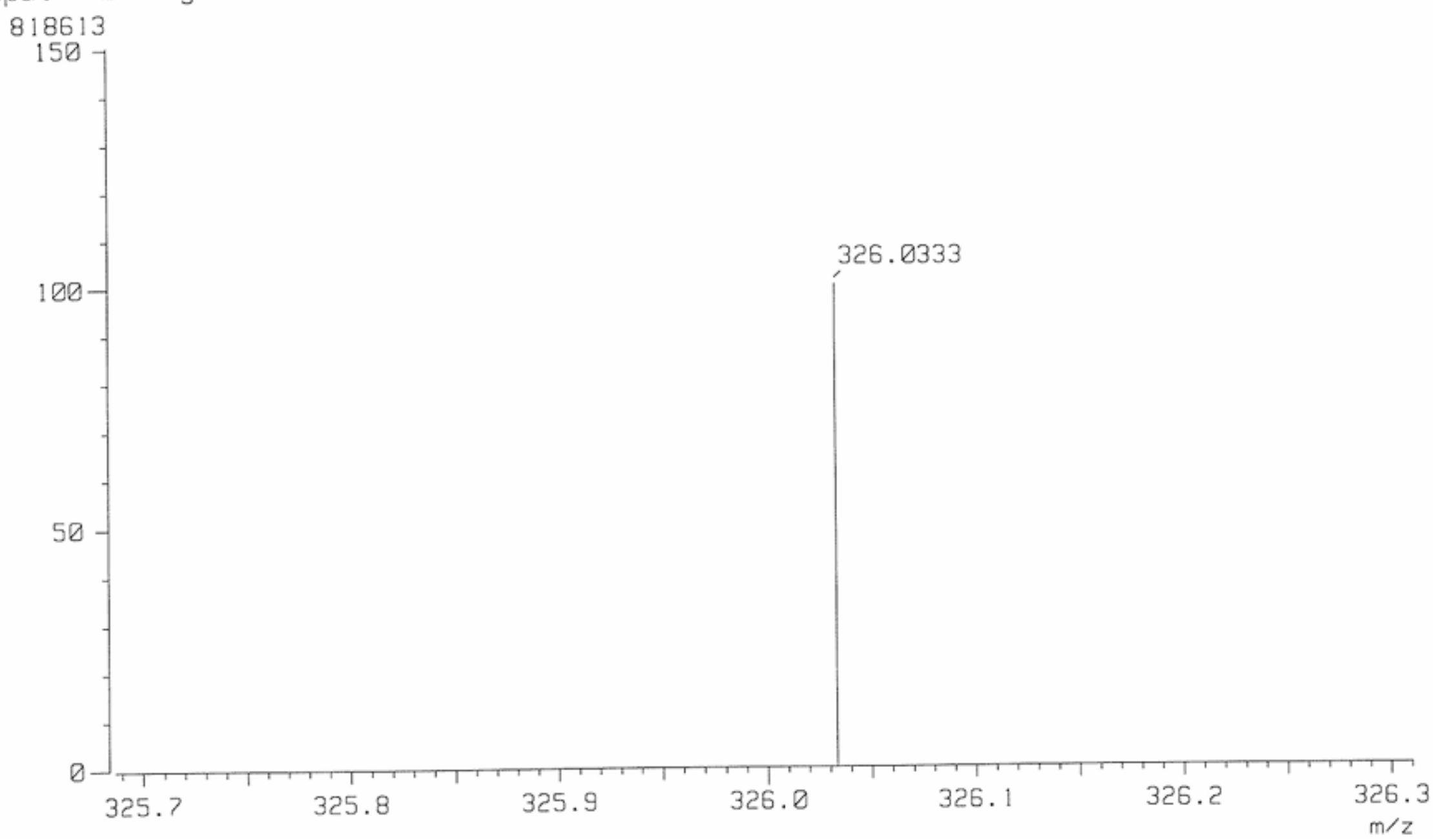

Compound 3h

[Elemental Composition]   Page: 1
Data : EI-29-Code-LR-92-F3-023   Date : 04-Jul-2026 10:05
Sample: High resolution GC Mass (JMS-700)
Note : SMLab-Mass Analysis & Research Center
Inlet : Direct   Ion Mode : EI+
RT : 1.27 min   Scan#: (38,40)
Elements : C 15/0, H 14/0, Br 2/0, F 1/0, N 2/0, O 3/0, S 1/0
Mass Tolerance : 1000ppm, 3mmu if m/z < 3, 5mmu if m/z > 5
Unsaturation (U.S.) : -0.5 - 80.0

Observed m/z	Int%	Err[ppm / mmu]	U.S.	Composition
445.8737	49.0	+0.4 / +0.2	11.0	C 14 H 9 Br 2 F N 2 O 2 S

[Mass Spectrum]
Data : EI-29-Code-LR-92-F3-023   Date : 04-Jul-2026 10:05
Sample: High resolution GC Mass (JMS-700)
Note : SMLab-Mass Analysis & Research Center
Inlet : Direct   Ion Mode : EI+
Spectrum Type : Normal Ion [MF-Linear]
RT : 1.27 min   Scan# : (38,40)
BP : m/z 447.8712   Int. : 52.94
Output m/z range : 445.4921 to 446.1763   Cut Level : 28.68 %

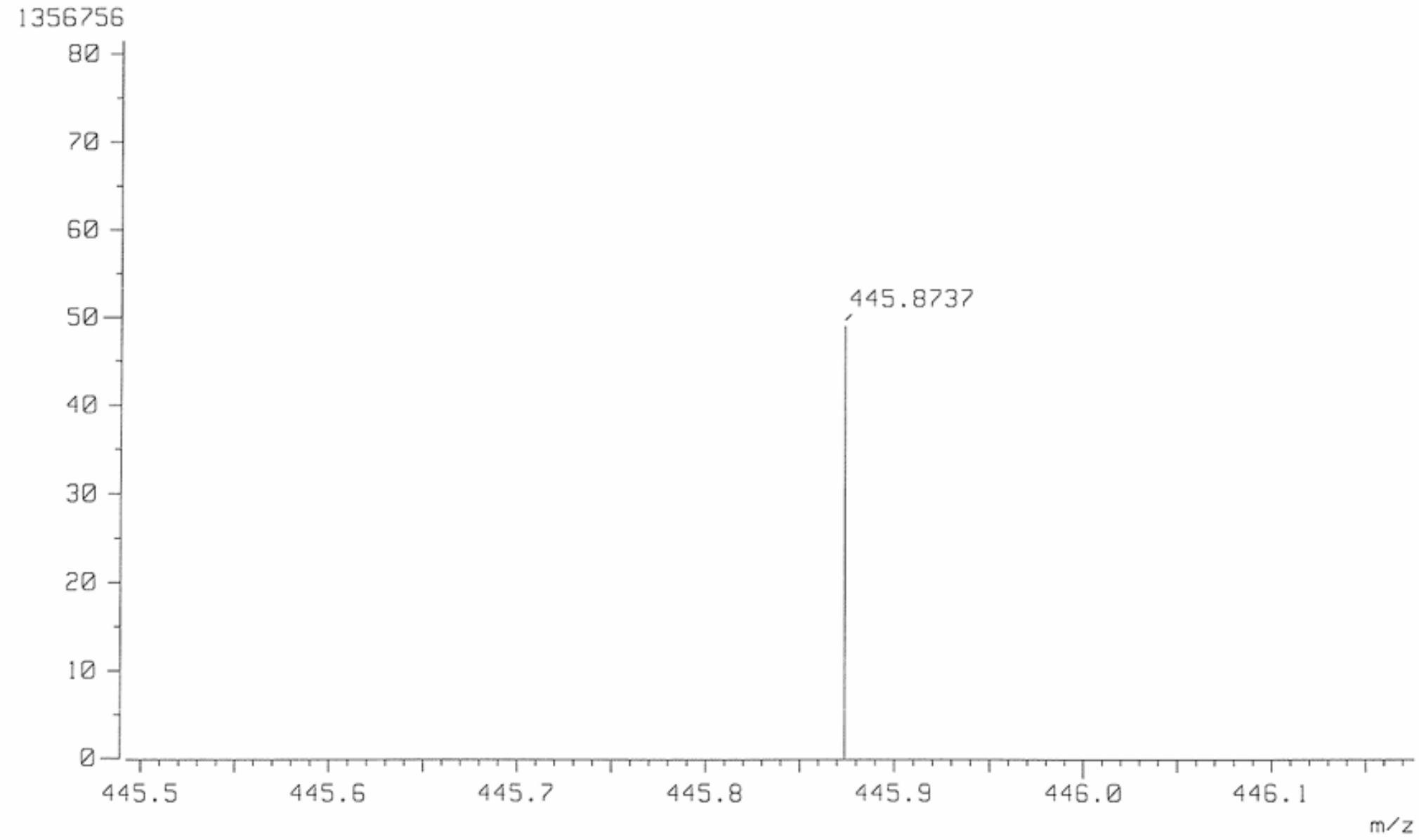

Compound 3i

```
[ Elemental Composition ]                                              Page: 1
Data   : EI-18-HS-265-014              Date : 03-Jul-2026 16:53
Sample: High resolution GC Mass (JMS-700)
Note : SMLab-Mass Analysis & Research Center
Inlet : Direct                          Ion Mode : EI+
RT : 0.25 min                           Scan#: (8,9)+12+16+20+23
Elements : C 22/0, H 20/0, F 1/0, N 2/0, O 7/0, S 1/0
Mass Tolerance        : 1000ppm, 3mmu if m/z < 3, 5mmu if m/z > 5
Unsaturation (U.S.) : -0.5 - 80.0

Observed m/z Int%   Err[ppm / mmu]    U.S.  Composition
  434.0950   100.0     +0.4 /  +0.2    13.0  C 20 H 19 F N 2 O 6 S
```

```
[ Mass Spectrum ]
Data   : EI-18-HS-265-014              Date : 03-Jul-2026 16:53
Sample: High resolution GC Mass (JMS-700)
Note : SMLab-Mass Analysis & Research Center
Inlet : Direct                          Ion Mode : EI+
Spectrum Type : Normal Ion [MF-Linear]
RT : 0.25 min          Scan# : (8,9)+12+16+20+23
BP : m/z 434.0950      Int.   : 34.76
Output m/z range : 433.8213 to 434.4085      Cut Level : 75.74 %
```

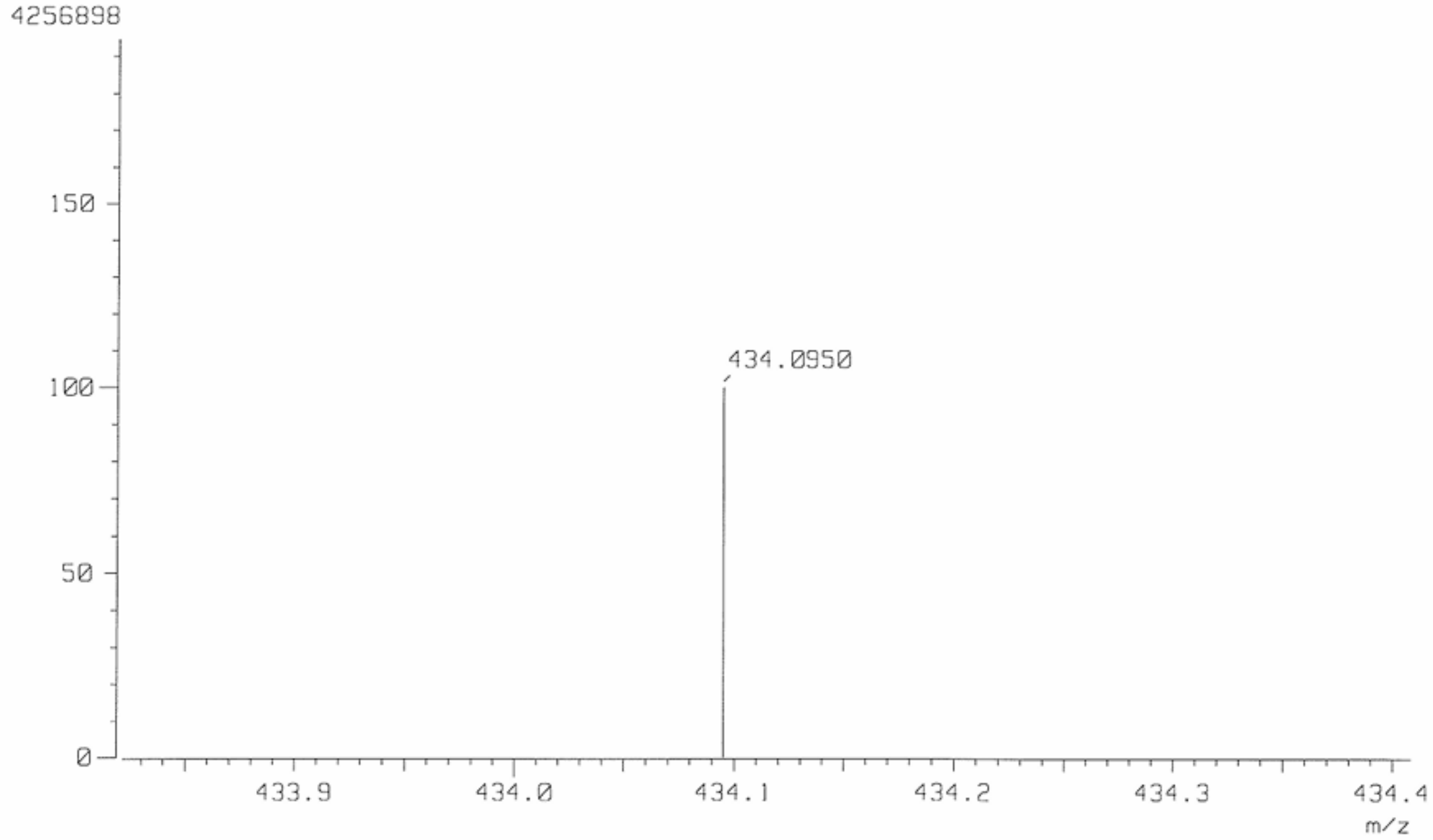

Compound 3j

[Elemental Composition] Page: 1
Data : EI-17-HS-264-009 Date : 03-Jul-2026 16:43
Sample: High resolution GC Mass (JMS-700)
Note : SMLab-Mass Analysis & Research Center
Inlet : Direct Ion Mode : EI+
RT : 0.80 min Scan#: 25
Elements : C 15/0, H 12/0, F 1/0, N 2/0, O 3/0, S 1/0
Mass Tolerance : 1000ppm, 3mmu if m/z < 3, 5mmu if m/z > 5
Unsaturation (U.S.) : -0.5 - 80.0

Observed m/z	Int%	Err[ppm / mmu]	U.S.	Composition
290.0525	72.6	-0.1 / +0.0	11.0	C 14 H 11 F N 2 O 2 S

[Mass Spectrum]
Data : EI-17-HS-264-009 Date : 03-Jul-2026 16:43
Sample: High resolution GC Mass (JMS-700)
Note : SMLab-Mass Analysis & Research Center
Inlet : Direct Ion Mode : EI+
Spectrum Type : Normal Ion [MF-Linear]
RT : 0.80 min Scan# : 25
BP : m/z 280.9864 Int. : 88.13
Output m/z range : 289.7019 to 290.3839 Cut Level : 57.35 %

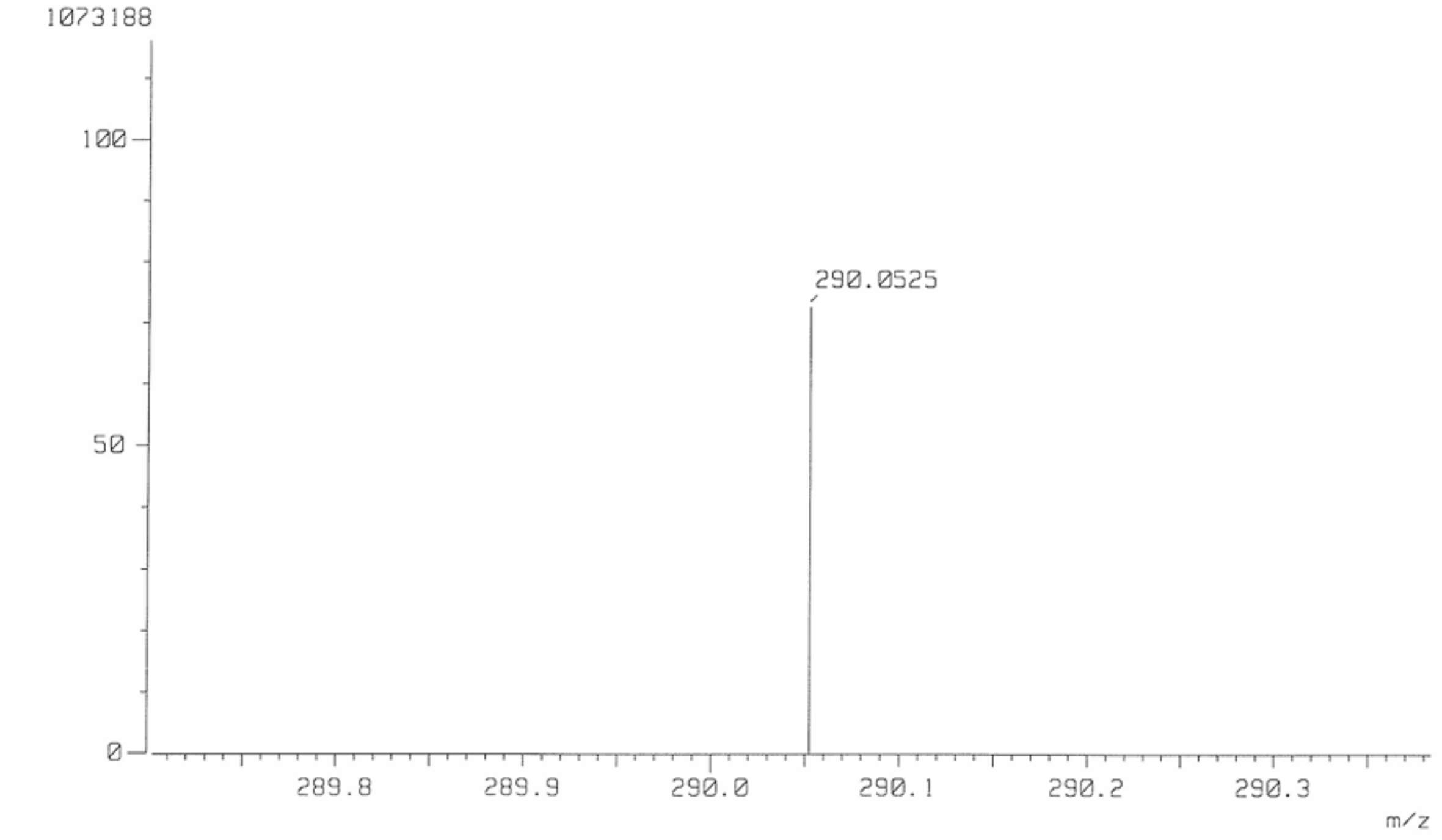

Compound 3k

[Elemental Composition]   Page: 1
Data : EI-2-HS-215-004   Date : 03-Jul-2026 15:26
Sample: High resolution GC Mass (JMS-700)
Note : SMLab-Mass Analysis & Research Center
Inlet : Direct   Ion Mode : EI+
RT : 0.90 min   Scan#: (23,33)
Elements : C 17/0, H 16/0, F 1/0, N 2/0, O 3/0, S 2/0
Mass Tolerance : 1000ppm, 3mmu if m/z < 3, 5mmu if m/z > 5
Unsaturation (U.S.) : -0.5 - 80.0

Observed m/z	Int%	Err[ppm / mmu]	U.S.	Composition
318.0834	100.0	-1.4 / -0.5	11.0	C 16 H 15 F N 2 O 2 S

[Mass Spectrum]
Data : EI-2-HS-215-004   Date : 03-Jul-2026 15:26
Sample: High resolution GC Mass (JMS-700)
Note : SMLab-Mass Analysis & Research Center
Inlet : Direct   Ion Mode : EI+
Spectrum Type : Normal Ion [MF-Linear]
RT : 0.90 min   Scan# : (23,33)
BP : m/z 318.0834   Int. : 36.23
Output m/z range : 317.7204 to 318.4415   Cut Level : 75.94 %

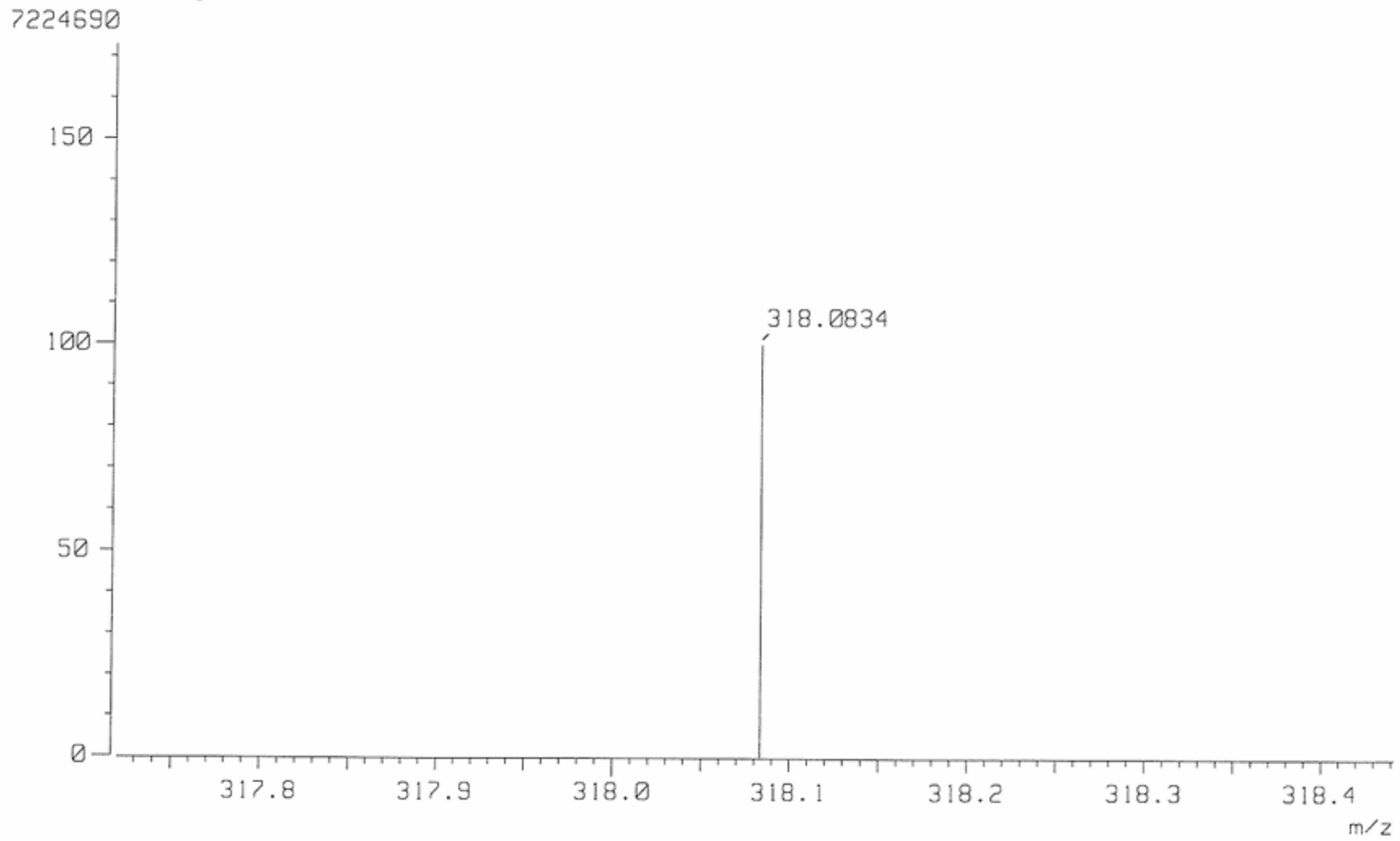

Compound 3l

[Elemental Composition] Page: 1

Data : EI-6-HS-235-011 Date : 03-Jul-2026 15:38
Sample: High resolution GC Mass (JMS-700)
Note : SMLab-Mass Analysis & Research Center
Inlet : Direct Ion Mode : EI+
RT : 0.77 min Scan#: 24
Elements : C 18/0, H 10/0, F 7/0, N 2/0, O 2/0, S 1/0
Mass Tolerance : 1000ppm, 3mmu if m/z < 3, 5mmu if m/z > 5
Unsaturation (U.S.) : -0.5 - 80.0

Observed m/z	Int%	Err[ppm / mmu]	U.S.	Composition
426.0270	100.0	-0.6 / -0.3	11.0	C 16 H 9 F 7 N 2 O 2 S

[Mass Spectrum]
Data : EI-6-HS-235-011 Date : 03-Jul-2026 15:38
Sample: High resolution GC Mass (JMS-700)
Note : SMLab-Mass Analysis & Research Center
Inlet : Direct Ion Mode : EI+
Spectrum Type : Normal Ion [MF-Linear]
RT : 0.77 min Scan# : 24
BP : m/z 426.0270 Int. : 58.65
Output m/z range : 425.6586 to 426.3903 Cut Level : 63.97 %

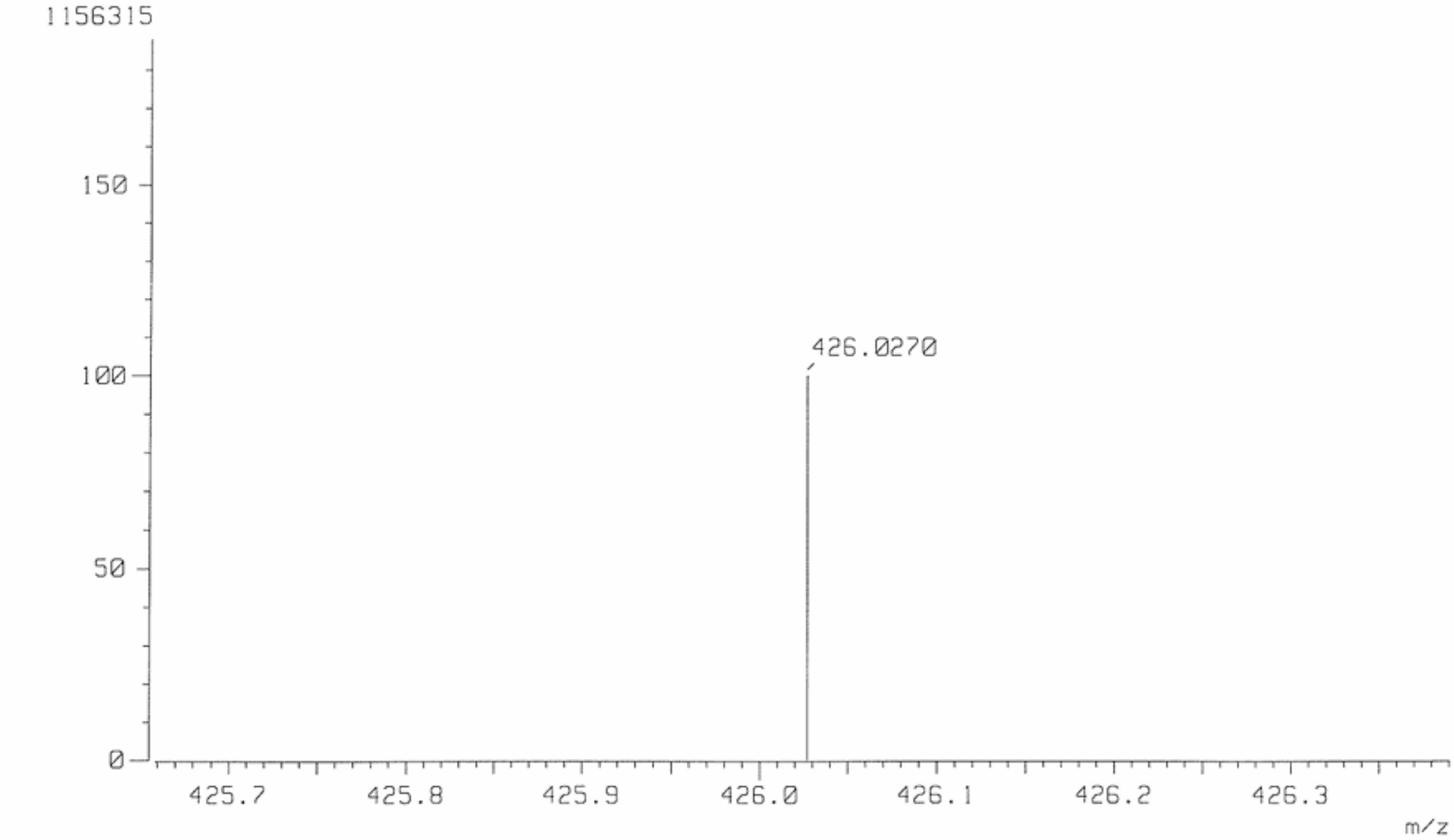

Compound 3m

[Elemental Composition] Page: 1
Data : EI-30-Code-LR-95-F2-004 Date : 04-Jul-2026 09:33
Sample: High resolution GC Mass (JMS-700)
Note : SMLab-Mass Analysis & Research Center
Inlet : Direct Ion Mode : EI+
RT : 0.45 min Scan#: (14,15)
Elements : C 14/0, H 14/0, Cl 2/0, F 1/0, N 2/0, O 3/0, S 1/0
Mass Tolerance : 1000ppm, 3mmu if m/z < 3, 5mmu if m/z > 5
Unsaturation (U.S.) : -0.5 - 80.0

Observed m/z	Int%	Err[ppm / mmu]	U.S.	Composition
357.9748	74.0	+0.6 / +0.2	11.0	C 14 H 9 Cl 2 F N 2 O 2 S

[Mass Spectrum]
Data : EI-30-Code-LR-95-F2-004 Date : 04-Jul-2026 09:33
Sample: High resolution GC Mass (JMS-700)
Note : SMLab-Mass Analysis & Research Center
Inlet : Direct Ion Mode : EI+
Spectrum Type : Normal Ion [MF-Linear]
RT : 0.45 min Scan# : (14,15)
BP : m/z 330.9792 Int. : 56.55
Output m/z range : 357.5989 to 358.3101 Cut Level : 59.56 %

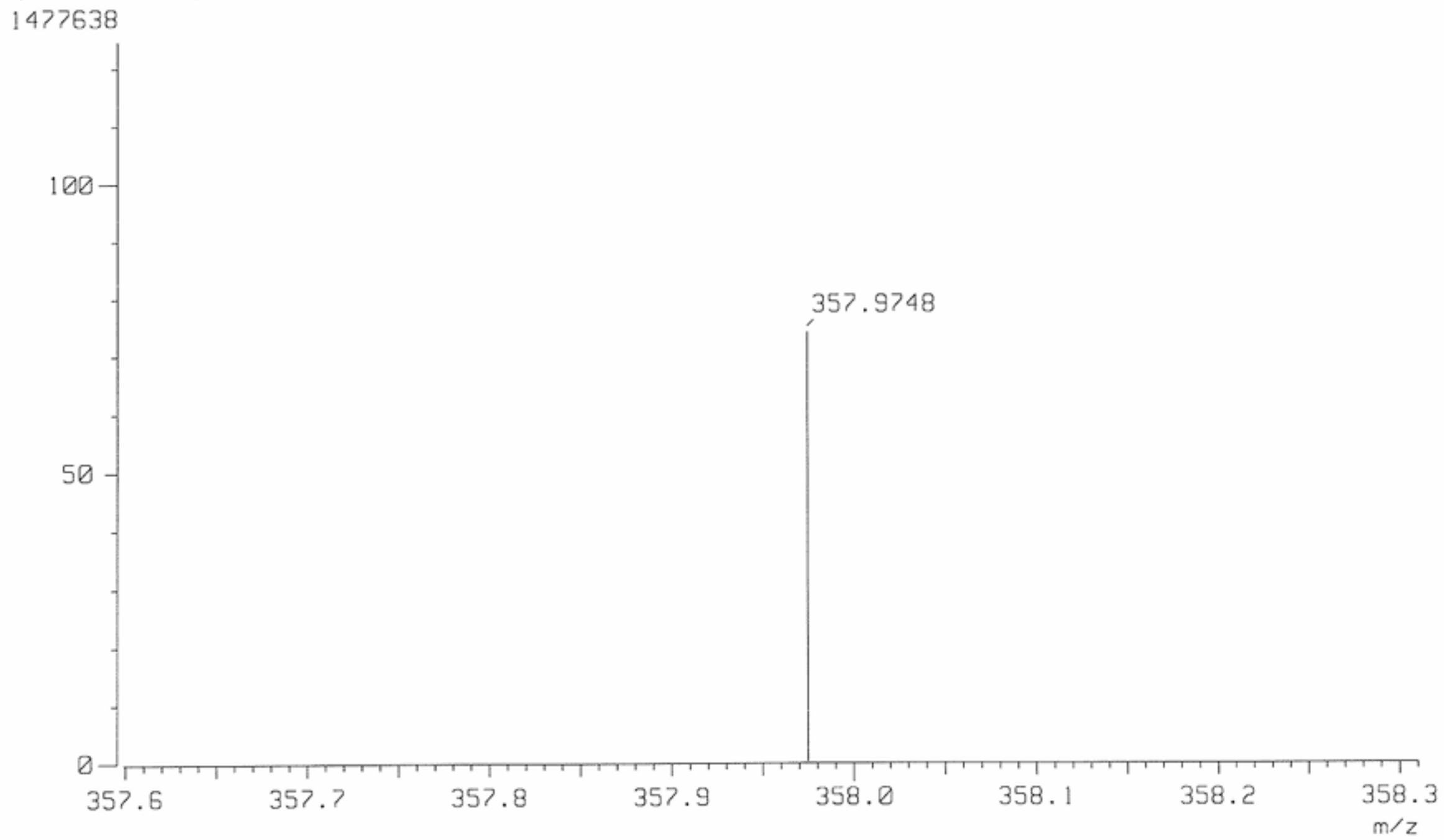

Compound 3n

```
[ Elemental Composition ]                                          Page: 1
Data   : EI-31-Code-LR-96-F2-024      Date : 04-Jul-2026 10:08
Sample: High resolution GC Mass (JMS-700)
Note : SMLab-Mass Analysis & Research Center
Inlet : Direct                        Ion Mode : EI+
RT : 1.20 min                         Scan#: (31,43)
Elements : C 15/0, H 14/0, Br 2/0, F 1/0, N 2/0, O 3/0, S 1/0
Mass Tolerance      : 1000ppm, 3mmu if m/z < 3, 5mmu if m/z > 5
Unsaturation (U.S.) : -0.5 - 80.0

Observed m/z Int%   Err[ppm / mmu]    U.S.  Composition
  445.8737    49.9     +0.4 /  +0.2   11.0  C 14 H 9 Br 2 F N 2 O 2 S
```

```
[ Mass Spectrum ]
Data   : EI-31-Code-LR-96-F2-024      Date : 04-Jul-2026 10:08
Sample: High resolution GC Mass (JMS-700)
Note : SMLab-Mass Analysis & Research Center
Inlet : Direct                        Ion Mode : EI+
Spectrum Type : Normal Ion [MF-Linear]
RT : 1.20 min            Scan# : (31,43)
BP : m/z 447.8712        Int.  : 61.12
Output m/z range : 445.5535 to 446.1893      Cut Level : 34.56 %
```

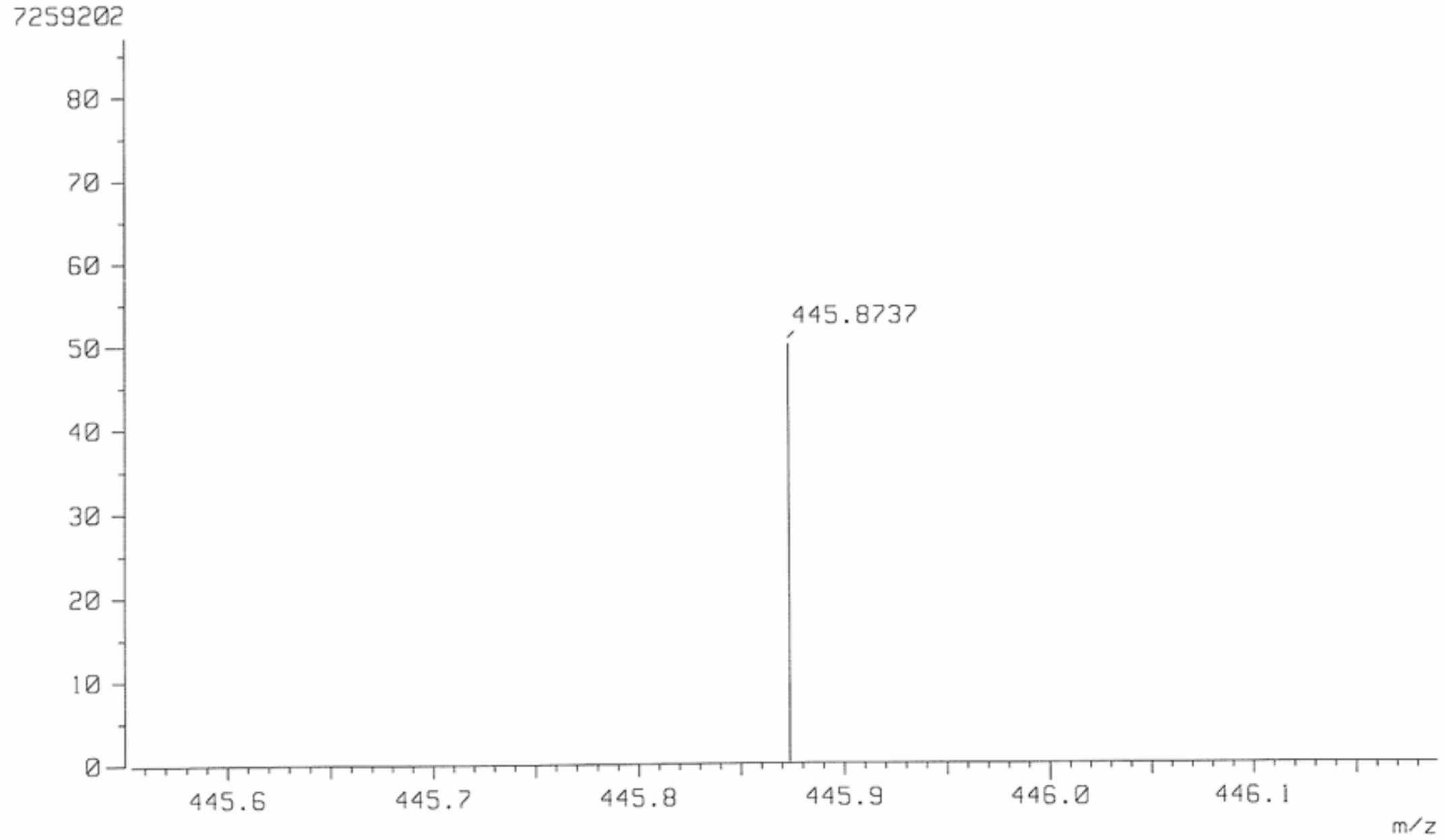

Compound 3o

[Elemental Composition] Page: 1
Data : EI-19-HS-266-018 Date : 03-Jul-2026 17:00
Sample: High resolution GC Mass (JMS-700)
Note : SMLab-Mass Analysis & Research Center
Inlet : Direct Ion Mode : EI+
RT : 0.44 min Scan#: 14+16
Elements : C 20/0, H 18/0, F 1/0, N 2/0, O 5/0, S 1/0
Mass Tolerance : 1000ppm, 3mmu if m/z < 3, 5mmu if m/z > 5
Unsaturation (U.S.) : -0.5 - 80.0

Observed m/z	Int%	Err[ppm / mmu]	U.S.	Composition
374.0737	100.0	+0.2 / +0.1	13.0	C 18 H 15 F N 2 O 4 S

[Mass Spectrum]
Data : EI-19-HS-266-018 Date : 03-Jul-2026 17:00
Sample: High resolution GC Mass (JMS-700)
Note : SMLab-Mass Analysis & Research Center
Inlet : Direct Ion Mode : EI+
Spectrum Type : Normal Ion [MF-Linear]
RT : 0.44 min Scan# : 14+16
BP : m/z 374.0737 Int. : 59.79
Output m/z range : 373.6907 to 374.3827 Cut Level : 74.26 %

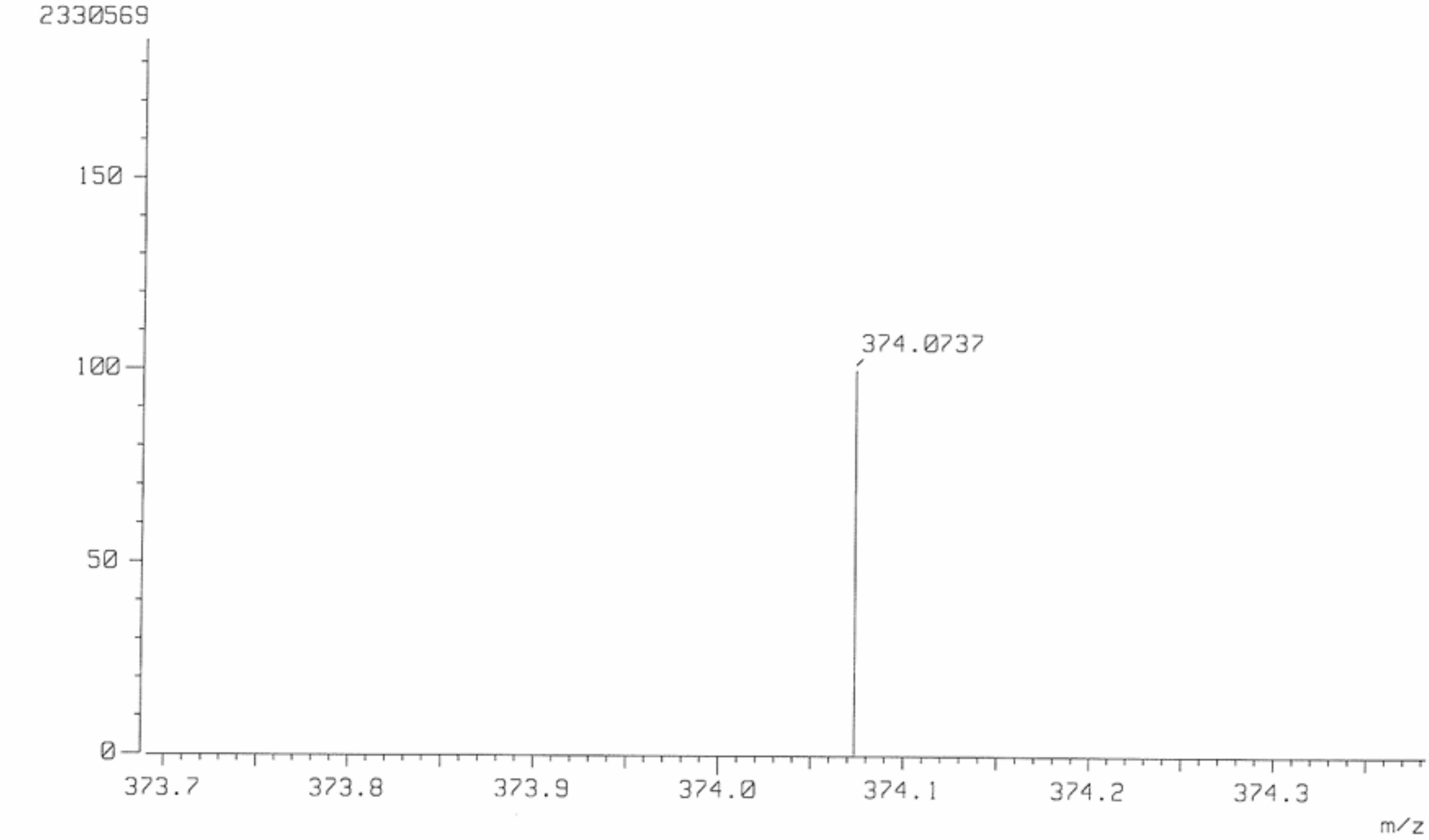

Compound 3p

```
[ Elemental Composition ]                                              Page: 1
Data   : EI-9-HS-238-013               Date : 03-Jul-2026 15:42
Sample: High resolution GC Mass (JMS-700)
Note : SMLab-Mass Analysis & Research Center
Inlet : Direct                         Ion Mode : EI+
RT : 0.94 min                          Scan#: 29+25+23+22
Elements : C 21/0, H 20/0, F 1/0, N 2/0, O 7/0, S 1/0
Mass Tolerance      : 1000ppm, 3mmu if m/z < 3, 5mmu if m/z > 5
Unsaturation (U.S.) : -0.5 - 80.0
```

Observed m/z	Int%	Err[ppm / mmu]	U.S.	Composition
434.0944	33.9	-1.0 / -0.4	13.0	C 20 H 19 F N 2 O 6 S

```
[ Mass Spectrum ]
Data   : EI-9-HS-238-013             Date : 03-Jul-2026 15:42
Sample: High resolution GC Mass (JMS-700)
Note : SMLab-Mass Analysis & Research Center
Inlet : Direct                       Ion Mode : EI+
Spectrum Type : Normal Ion [MF-Linear]
RT : 0.94 min          Scan# : 29+25+23+22
BP : m/z 374.1496      Int.   : 90.05
Output m/z range : 433.7915 to 434.3655      Cut Level : 8.09 %
```

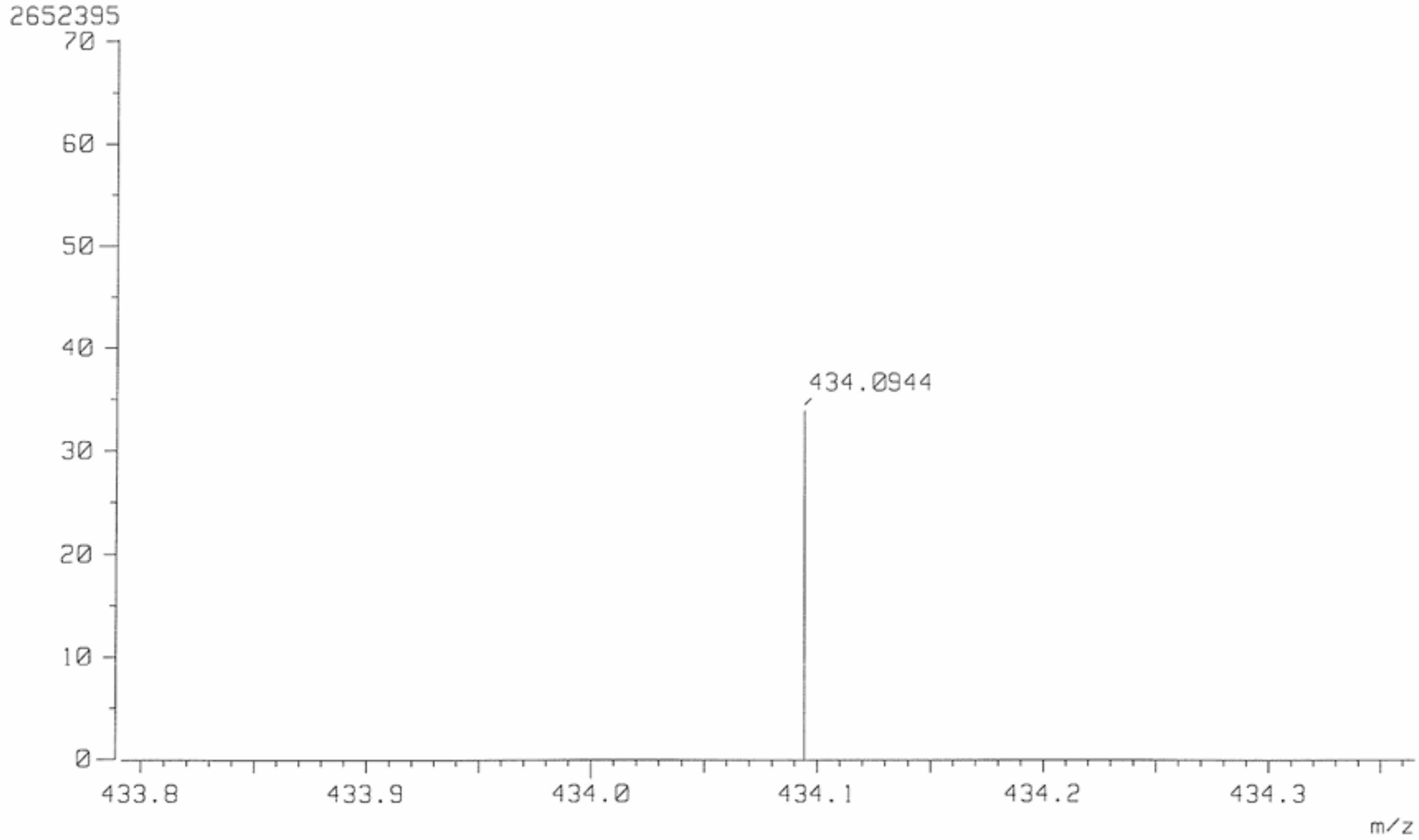

Compound 3aa

[Elemental Composition]
Data : EI-1-HS-4a-007 Date : 16-Sep-2026 15:19 Page: 1
Sample: High resolution GC Mass (JMS-700)
Note : SMLab-Mass Analysis & Research Center
Inlet : Direct Ion Mode : EI+
RT : 0.20 min Scan#: 7
Elements : C 17/0, H 20/0, F 1/0, N 2/0, O 2/0, S 1/0
Mass Tolerance : 1000ppm, 3mmu if m/z < 3, 5mmu if m/z > 5
Unsaturation (U.S.) : -0.5 - 80.0

Observed m/z	Int%	Err[ppm / mmu]	U.S.	Composition
320.0990	100.0	-1.5 / -0.5	10.0	C 16 H 17 F N 2 O 2 S

[Mass Spectrum]
Data : EI-1-HS-4a-007 Date : 16-Sep-2026 15:19
Sample: High resolution GC Mass (JMS-700)
Note : SMLab-Mass Analysis & Research Center
Inlet : Direct Ion Mode : EI+
Spectrum Type : Normal Ion [MF-Linear]
RT : 0.20 min Scan# : 7
BP : m/z 320.0990 Int. : 399.92
Output m/z range : 319.8122 to 320.3468 Cut Level : 0.00 %

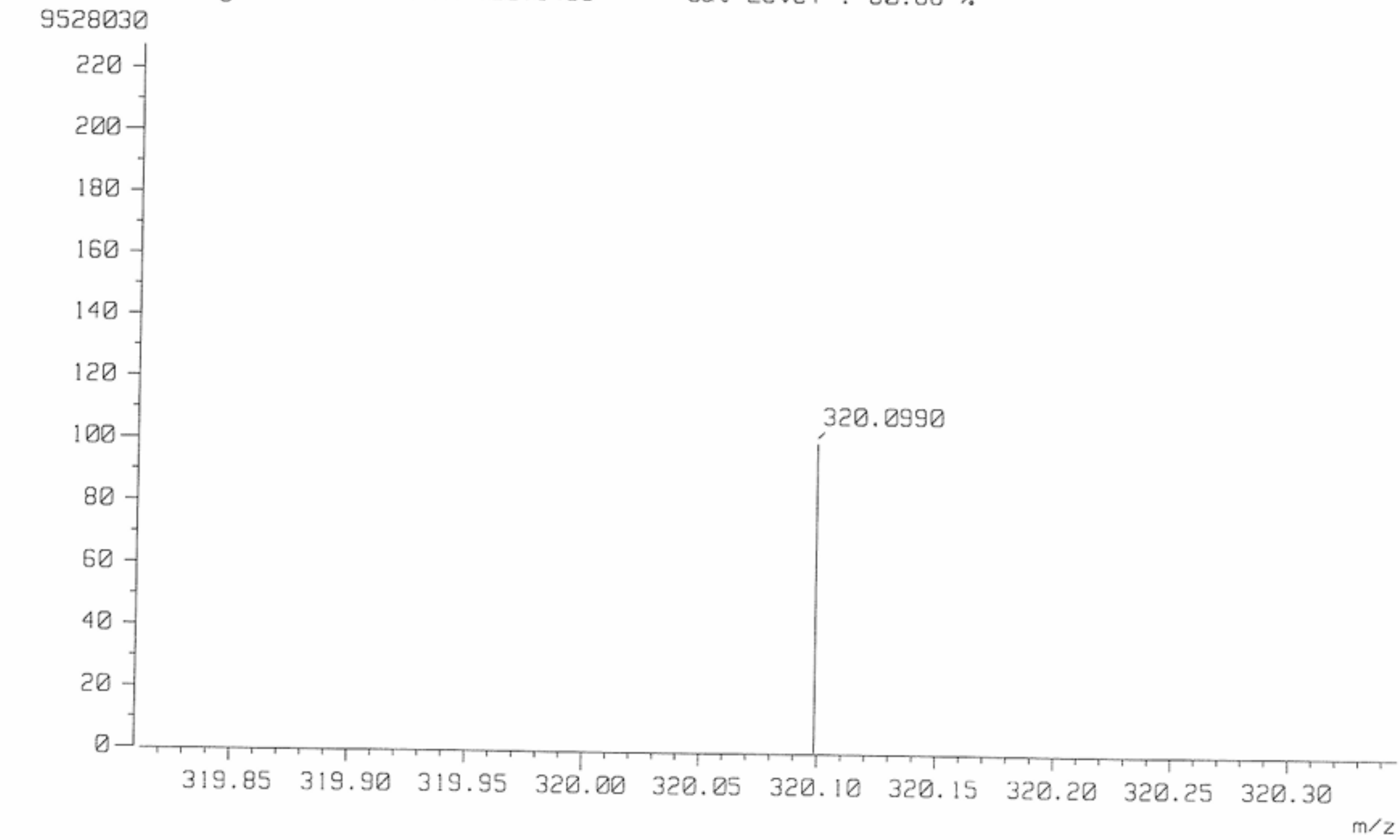

Compound 5a

[Elemental Composition] Page: 1
Data : EI-13-HS-259-002 Date : 03-Jul-2026 16:11
Sample: High resolution GC Mass (JMS-700)
Note : SMLab-Mass Analysis & Research Center
Inlet : Direct Ion Mode : EI+
RT : 0.94 min Scan#: 29
Elements : C 20/0, H 20/0, F 1/0, N 2/0, O 3/0, S 1/0
Mass Tolerance : 1000ppm, 3mmu if m/z < 3, 5mmu if m/z > 5
Unsaturation (U.S.) : -0.5 - 80.0

Observed m/z	Int%	Err[ppm / mmu]	U.S.	Composition
346.1147	99.0	-1.1 / -0.4	11.0	C 18 H 19 F N 2 O 2 S

[Mass Spectrum]
Data : EI-13-HS-259-002 Date : 03-Jul-2026 16:11
Sample: High resolution GC Mass (JMS-700)
Note : SMLab-Mass Analysis & Research Center
Inlet : Direct Ion Mode : EI+
Spectrum Type : Normal Ion [MF-Linear]
RT : 0.94 min Scan# : 29
BP : m/z 330.9792 Int. : 49.14
Output m/z range : 345.7822 to 346.4178 Cut Level : 72.79 %

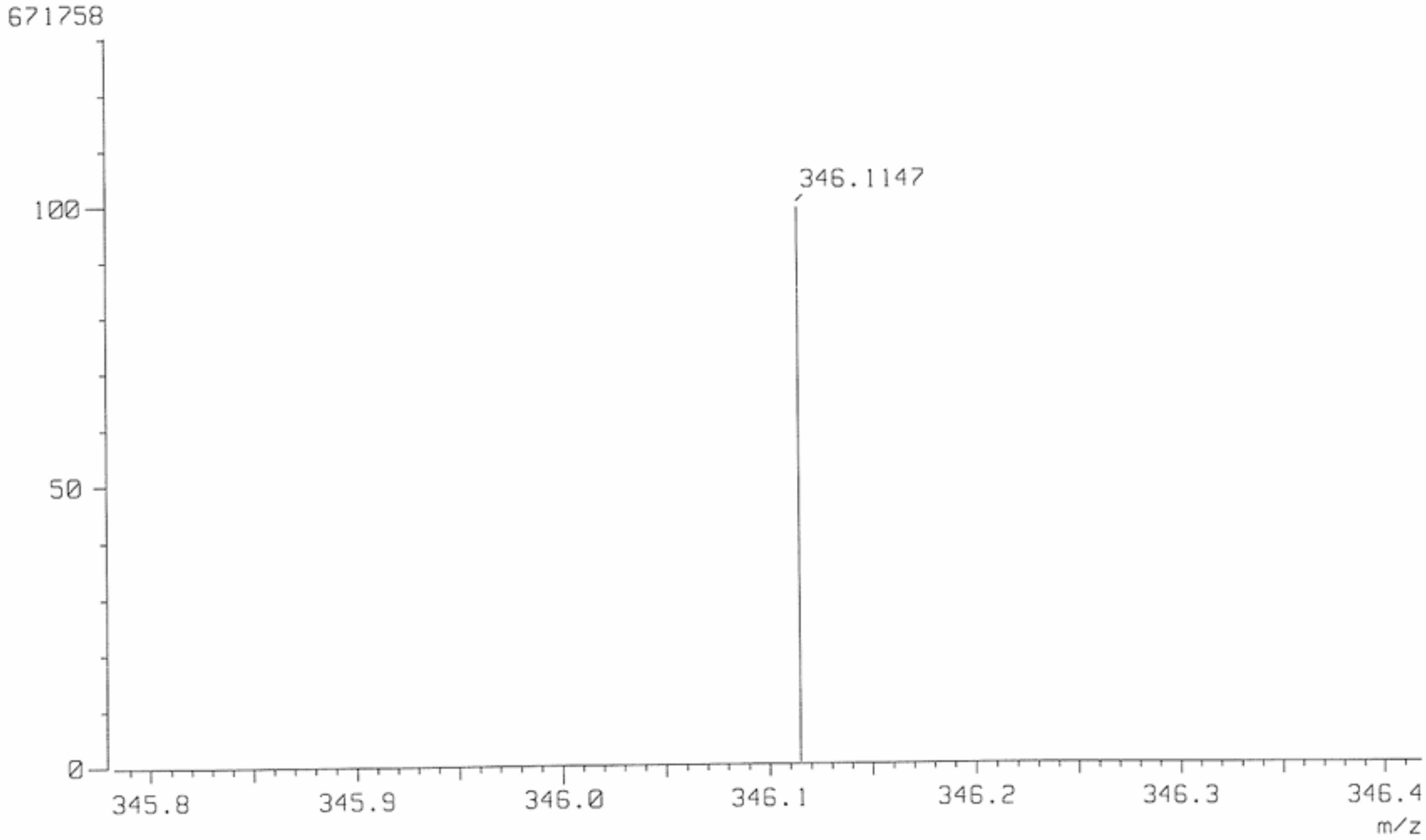

Compound 5b

[Elemental Composition] Page: 1
Data : EI-33-Code-LR-100-F2-010 Date : 04-Jul-2026 09:44
Sample: High resolution GC Mass (JMS-700)
Note : SMLab-Mass Analysis & Research Center
Inlet : Direct Ion Mode : EI+
RT : 0.55 min Scan#: (16,19)+21+22
Elements : C 17/0, H 14/0, F 3/0, N 2/0, O 3/0, S 1/0
Mass Tolerance : 1000ppm, 3mmu if m/z < 3, 5mmu if m/z > 5
Unsaturation (U.S.) : -0.5 - 80.0

Observed m/z	Int%	Err[ppm / mmu]	U.S.	Composition
354.0651	100.0	+0.2 / +0.1	11.0	C 16 H 13 F 3 N 2 O 2 S

[Mass Spectrum]
Data : EI-33-Code-LR-100-F2-010 Date : 04-Jul-2026 09:44
Sample: High resolution GC Mass (JMS-700)
Note : SMLab-Mass Analysis & Research Center
Inlet : Direct Ion Mode : EI+
Spectrum Type : Normal Ion [MF-Linear]
RT : 0.55 min Scan# : (16,19)+21+22
BP : m/z 354.0651 Int. : 28.62
Output m/z range : 353.7304 to 354.3839 Cut Level : 77.21 %

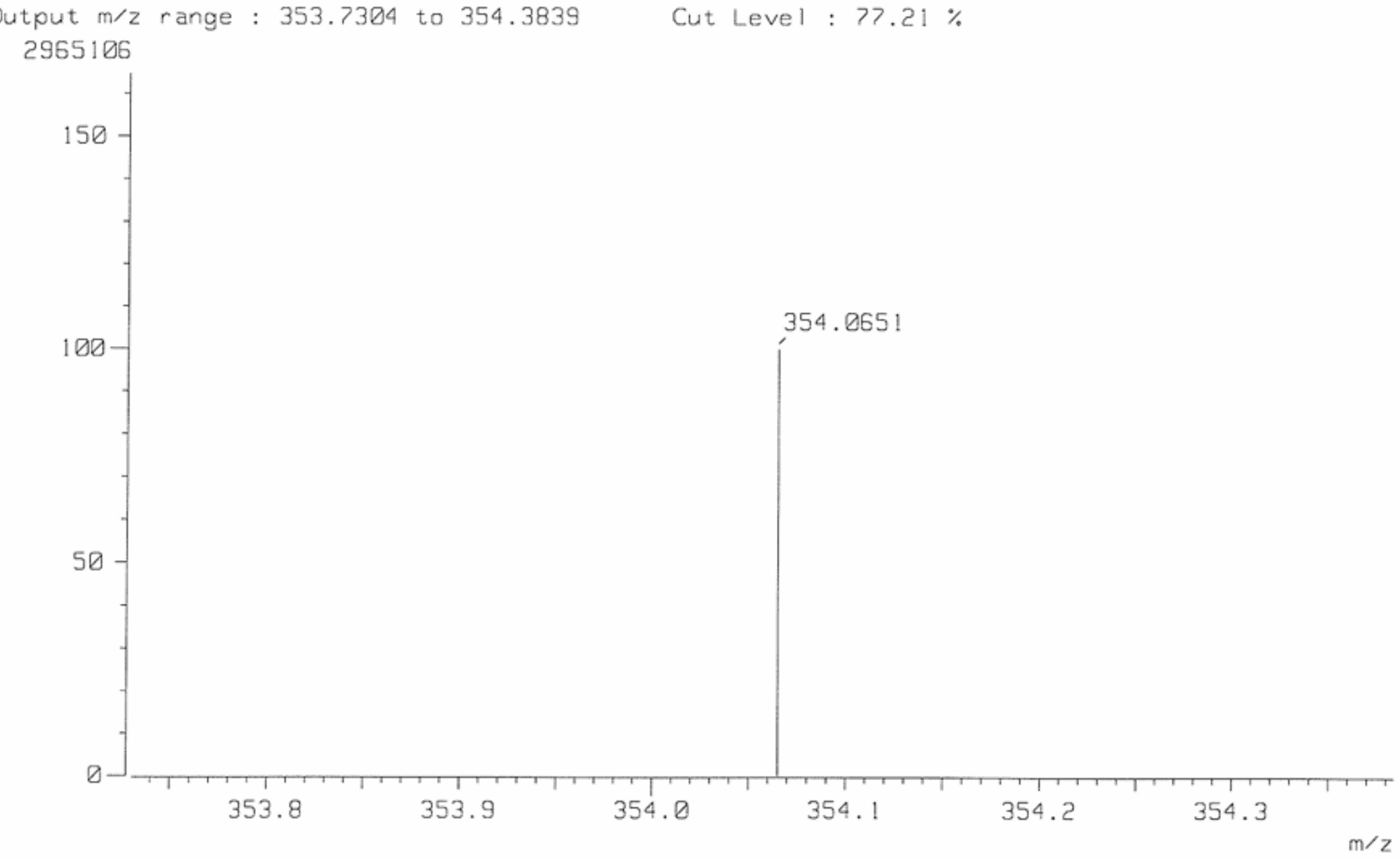

Compound 5c

[Elemental Composition]　　　　　　　　　　　　　　　　　　　Page: 1
Data : EI-20-HS-267-019　　　　　　　Date : 03-Jul-2026 17:03
Sample: High resolution GC Mass (JMS-700)
Note : SMLab-Mass Analysis & Research Center
Inlet : Direct　　　　　　　　　　　　Ion Mode : EI+
RT : 0.57 min　　　　　　　　　　　　Scan#: (17,19)+26
Elements : C 17/0, H 14/0, Cl 2/0, F 1/0, N 2/0, O 3/0, S 1/0
Mass Tolerance : 1000ppm, 3mmu if m/z < 3, 5mmu if m/z > 5
Unsaturation (U.S.) : -0.5 - 80.0

Observed m/z	Int%	Err[ppm / mmu]	U.S.	Composition
386.0059	100.0	-0.1 / +0.0	11.0	C 16 H 13 Cl 2 F N 2 O 2 S

[Mass Spectrum]
Data : EI-20-HS-267-019　　　　Date : 03-Jul-2026 17:03
Sample: High resolution GC Mass (JMS-700)
Note : SMLab-Mass Analysis & Research Center
Inlet : Direct　　　　　　　　　Ion Mode : EI+
Spectrum Type : Normal Ion [MF-Linear]
RT : 0.57 min　　　　Scan# : (17,19)+26
BP : m/z 386.0059　　Int. : 37.78
Output m/z range : 385.7055 to 386.2889　　Cut Level : 47.92 %

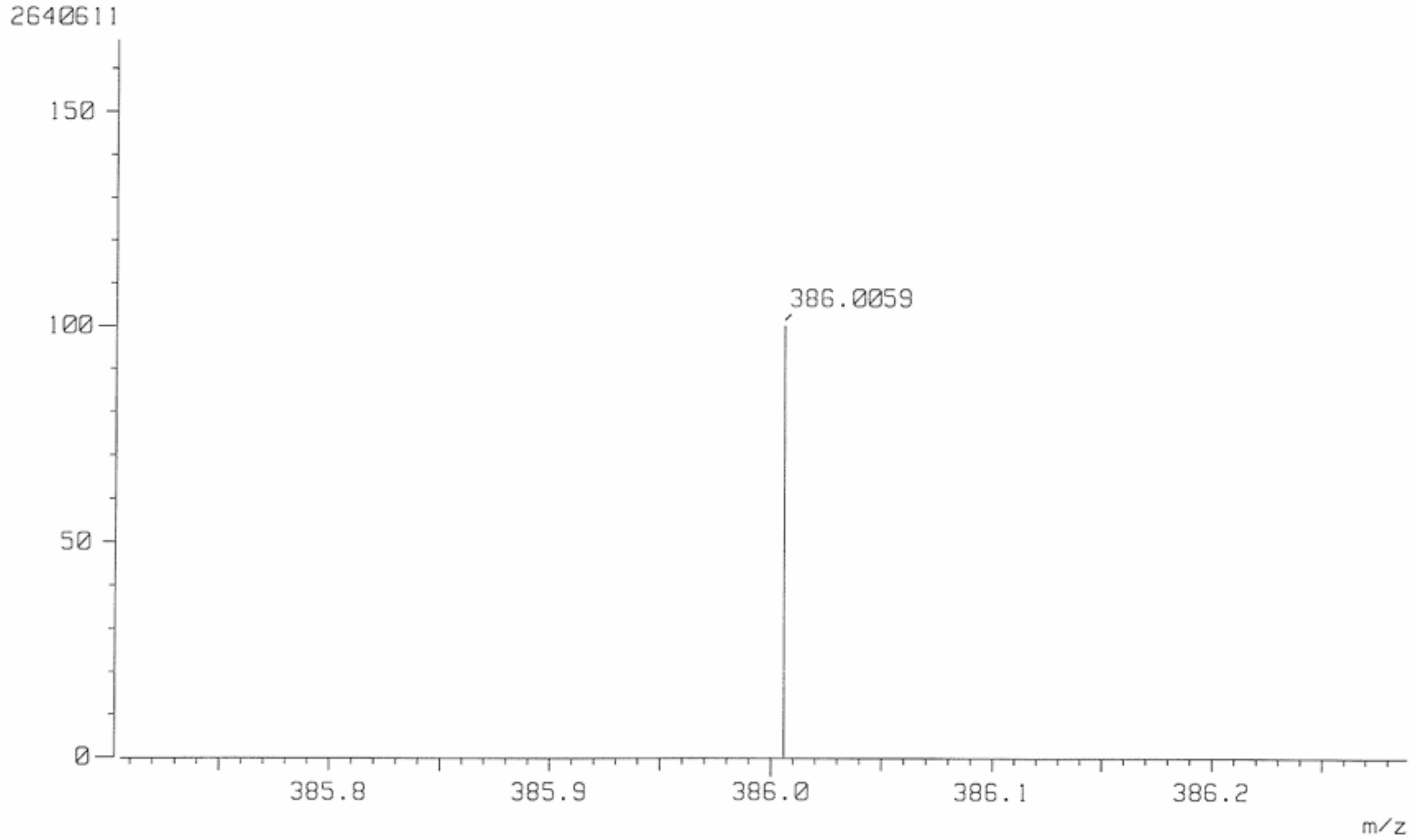

Compound 5d

[Elemental Composition] Page: 1
Data : EI-34-Code-LR-101-F3-008 Date : 04-Jul-2026 09:39
Sample: High resolution GC Mass (JMS-700)
Note : SMLab-Mass Analysis & Research Center
Inlet : Direct Ion Mode : EI+
RT : 0.44 min Scan#: (13,15)
Elements : C 17/0, H 14/0, F 3/0, N 2/0, O 3/0, S 1/0
Mass Tolerance : 1000ppm, 3mmu if m/z < 3, 5mmu if m/z > 5
Unsaturation (U.S.) : -0.5 - 80.0

Observed m/z	Int%	Err[ppm / mmu]	U.S.	Composition
354.0648	100.0	-0.6 / -0.2	11.0	C 16 H 13 F 3 N 2 O 2 S

[Mass Spectrum]
Data : EI-34-Code-LR-101-F3-008 Date : 04-Jul-2026 09:39
Sample: High resolution GC Mass (JMS-700)
Note : SMLab-Mass Analysis & Research Center
Inlet : Direct Ion Mode : EI+
Spectrum Type : Normal Ion [MF-Linear]
RT : 0.44 min Scan# : (13,15)
BP : m/z 354.0648 Int. : 67.41
Output m/z range : 353.7226 to 354.3914 Cut Level : 78.68 %

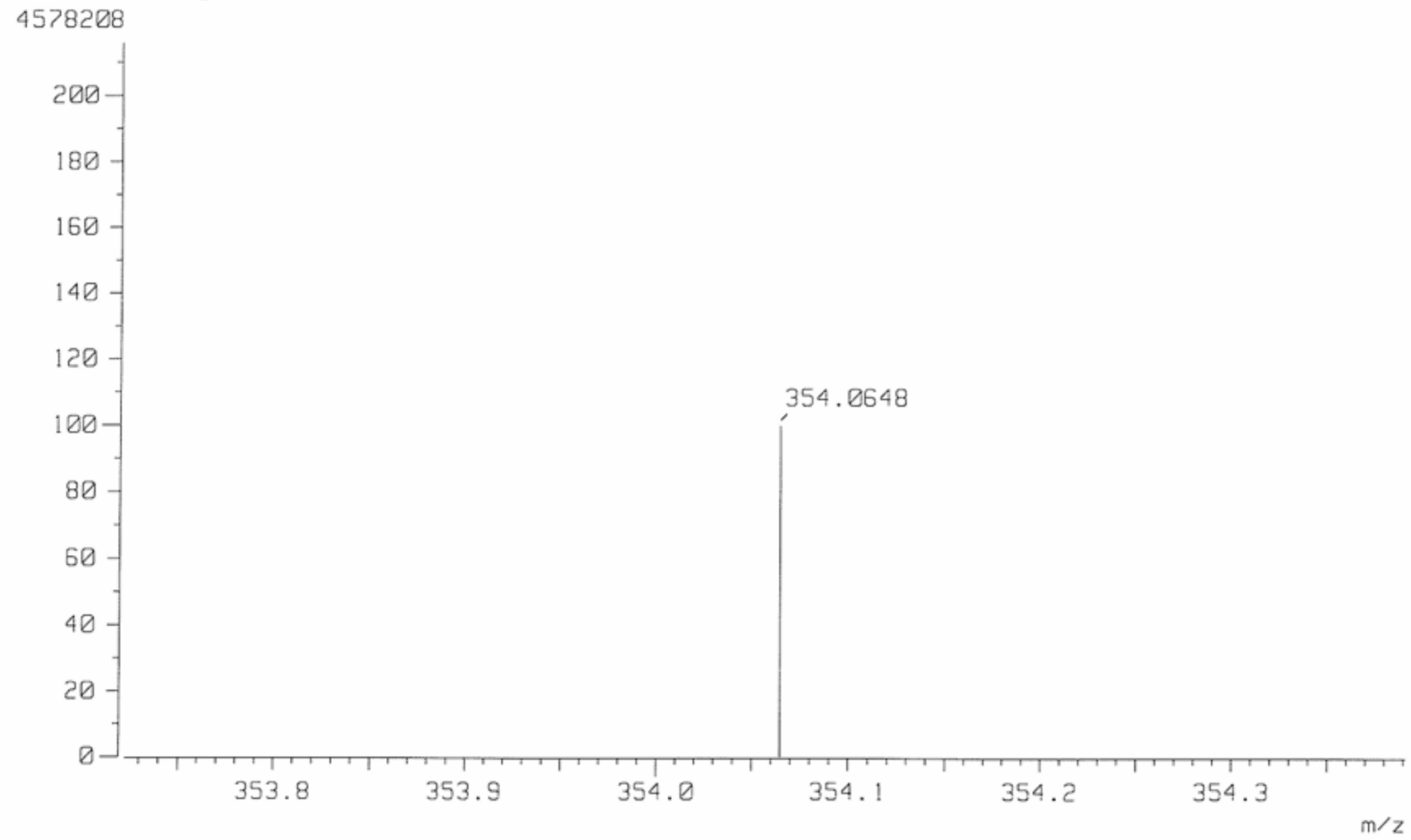

Compound 5e

[Elemental Composition] Page: 1
Data : EI-32-Code-LR-98-F3-002 Date : 04-Jul-2026 09:29
Sample: High resolution GC Mass (JMS-700)
Note : SMLab-Mass Analysis & Research Center
Inlet : Direct Ion Mode : EI+
RT : 0.62 min Scan#: (19,20)
Elements : C 20/0, H 20/0, F 1/0, N 2/0, O 3/0, S 1/0
Mass Tolerance : 1000ppm, 3mmu if m/z < 3, 5mmu if m/z > 5
Unsaturation (U.S.) : -0.5 - 80.0

Observed m/z	Int%	Err[ppm / mmu]	U.S.	Composition
346.1150	100.0	-0.3 / -0.1	11.0	C 18 H 19 F N 2 O 2 S

[Mass Spectrum]
Data : EI-32-Code-LR-98-F3-002 Date : 04-Jul-2026 09:29
Sample: High resolution GC Mass (JMS-700)
Note : SMLab-Mass Analysis & Research Center
Inlet : Direct Ion Mode : EI+
Spectrum Type : Normal Ion [MF-Linear]
RT : 0.62 min Scan# : (19,20)
BP : m/z 346.1150 Int. : 77.80
Output m/z range : 345.8177 to 346.3625 Cut Level : 74.26 %

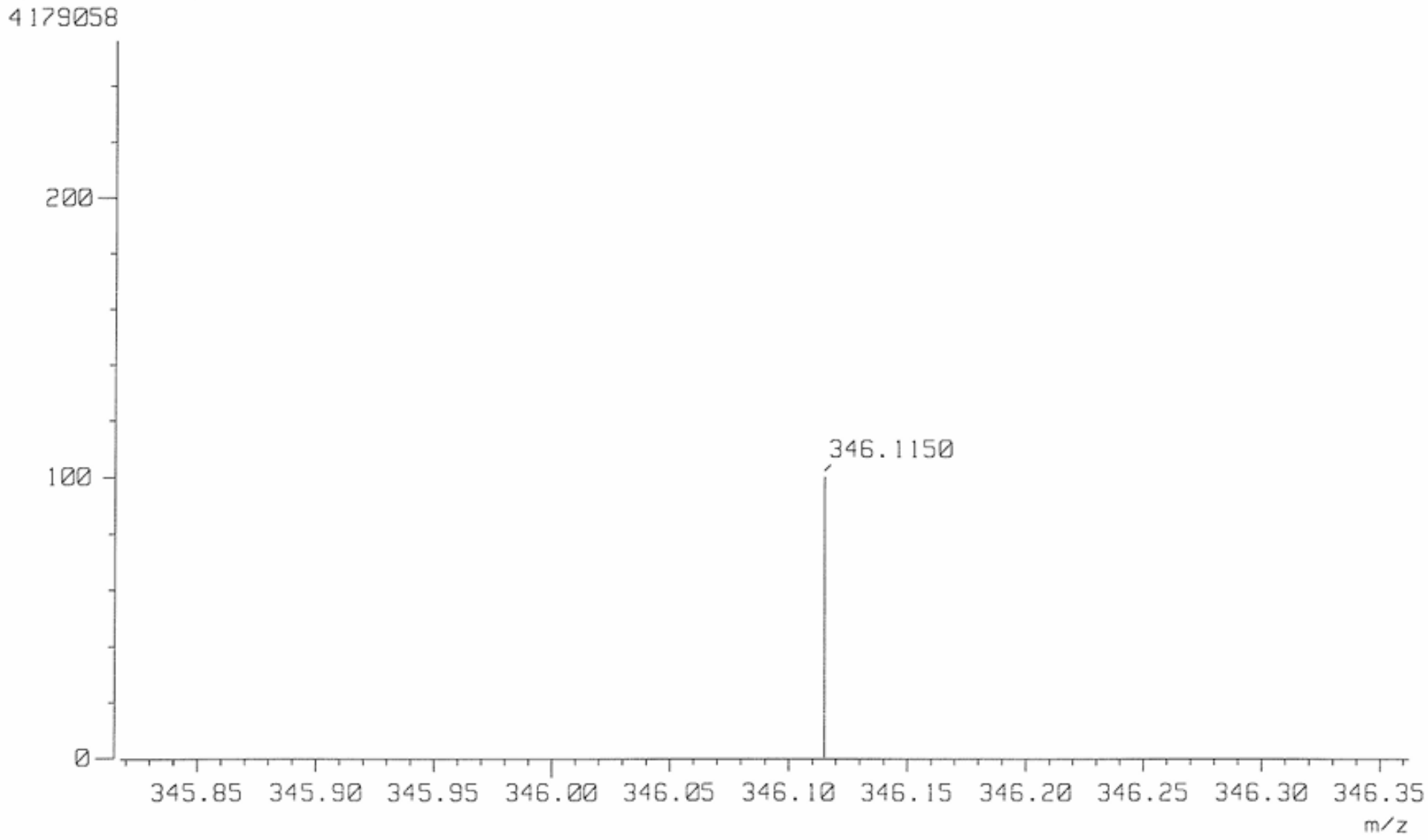

Compound 5f

[Elemental Composition] Page: 1
Data : EI-14-HS-260-021 Date : 03-Jul-2026 15:57
Sample: High resolution GC Mass (JMS-700)
Note : SMLab-Mass Analysis & Research Center
Inlet : Direct Ion Mode : EI+
RT : 0.74 min Scan#: 23
Elements : C 18/0, H 15/0, F 3/0, N 2/0, O 3/0, S 1/0
Mass Tolerance : 1000ppm, 3mmu if m/z < 3, 5mmu if m/z > 5
Unsaturation (U.S.) : -0.5 - 80.0

Observed m/z	Int%	Err[ppm / mmu]	U.S.	Composition
354.0646	95.0	-1.2 / -0.4	11.0	C 16 H 13 F 3 N 2 O 2 S

[Mass Spectrum]
Data : EI-14-HS-260-021 Date : 03-Jul-2026 15:57
Sample: High resolution GC Mass (JMS-700)
Note : SMLab-Mass Analysis & Research Center
Inlet : Direct Ion Mode : EI+
Spectrum Type : Normal Ion [MF-Linear]
RT : 0.74 min Scan# : 23
BP : m/z 330.9750 Int. : 65.81
Output m/z range : 353.7357 to 354.3895 Cut Level : 64.71 %

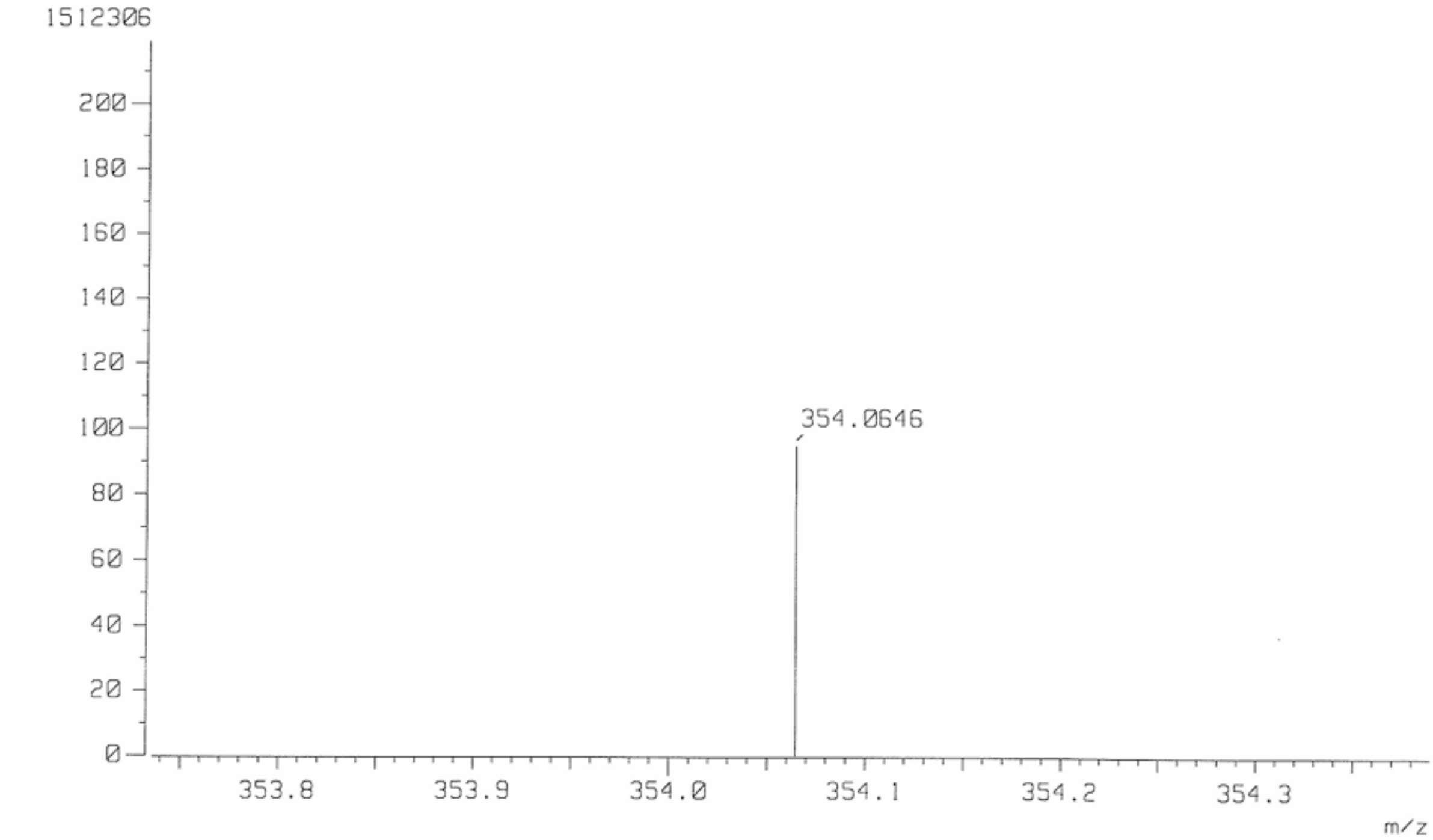

Compound 5g

```
[ Elemental Composition ]                                                Page: 1
Data   : EI-37-Code-LR-104-F2-016      Date : 04-Jul-2026 09:53
Sample: High resolution GC Mass (JMS-700)
Note : SMLab-Mass Analysis & Research Center
Inlet : Direct                         Ion Mode : EI+
RT : 1.17 min                          Scan#: (35,37)
Elements : C 17/0, H 14/0, Cl 2/0, F 1/0, N 2/0, O 3/0, S 1/0
Mass Tolerance      : 1000ppm, 3mmu if m/z < 3, 5mmu if m/z > 5
Unsaturation (U.S.) : -0.5 - 80.0

Observed m/z  Int%   Err[ppm / mmu]   U.S.  Composition
  386.0054    89.5     -1.3 /  -0.5   11.0  C 16 H 13 Cl 2 F N 2 O 2 S
```

```
[ Mass Spectrum ]
Data   : EI-37-Code-LR-104-F2-016      Date : 04-Jul-2026 09:53
Sample: High resolution GC Mass (JMS-700)
Note : SMLab-Mass Analysis & Research Center
Inlet : Direct                         Ion Mode : EI+
Spectrum Type : Normal Ion [MF-Linear]
RT : 1.17 min            Scan# : (35,37)
BP : m/z 330.9807        Int.  : 62.03
Output m/z range : 385.6056 to 386.3035      Cut Level : 73.53 %
```

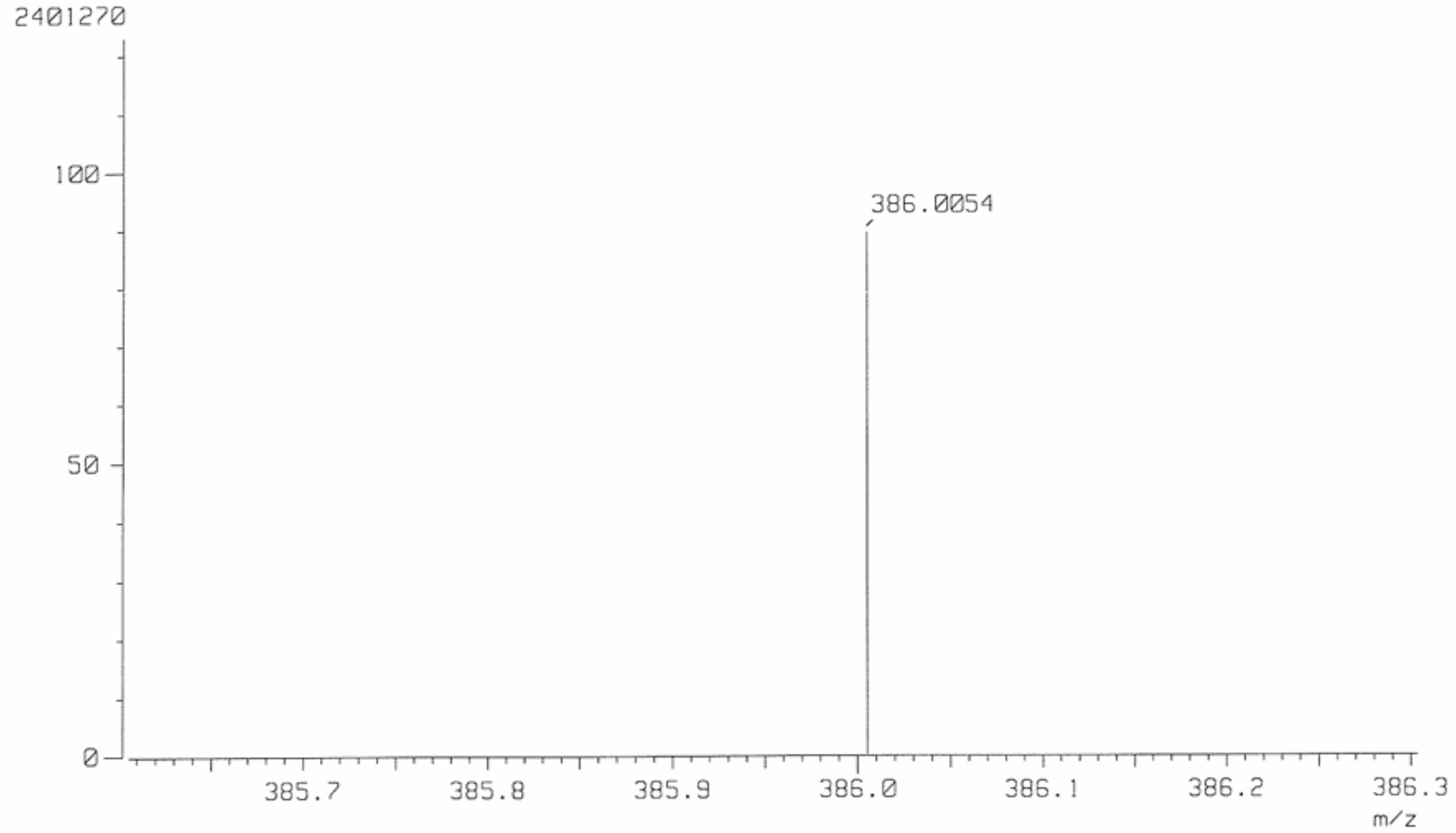

Compound 5h

[Elemental Composition] Page: 1
Data : EI-35-Code-LR-102-F2-009 Date : 04-Jul-2026 09:41
Sample: High resolution GC Mass (JMS-700)
Note : SMLab-Mass Analysis & Research Center
Inlet : Direct Ion Mode : EI+
RT : 0.84 min Scan#: 26
Elements : C 17/0, H 14/0, F 3/0, N 2/0, O 3/0, S 1/0
Mass Tolerance : 1000ppm, 3mmu if m/z < 3, 5mmu if m/z > 5
Unsaturation (U.S.) : -0.5 - 80.0

Observed m/z	Int%	Err[ppm / mmu]	U.S.	Composition
354.0650	64.4	+0.1 / +0.0	11.0	C 16 H 13 F 3 N 2 O 2 S

[Mass Spectrum]
Data : EI-35-Code-LR-102-F2-009 Date : 04-Jul-2026 09:41
Sample: High resolution GC Mass (JMS-700)
Note : SMLab-Mass Analysis & Research Center
Inlet : Direct Ion Mode : EI+
Spectrum Type : Normal Ion [MF-Linear]
RT : 0.84 min Scan# : 26
BP : m/z 330.9814 Int. : 49.04
Output m/z range : 353.7044 to 354.3787 Cut Level : 46.32 %

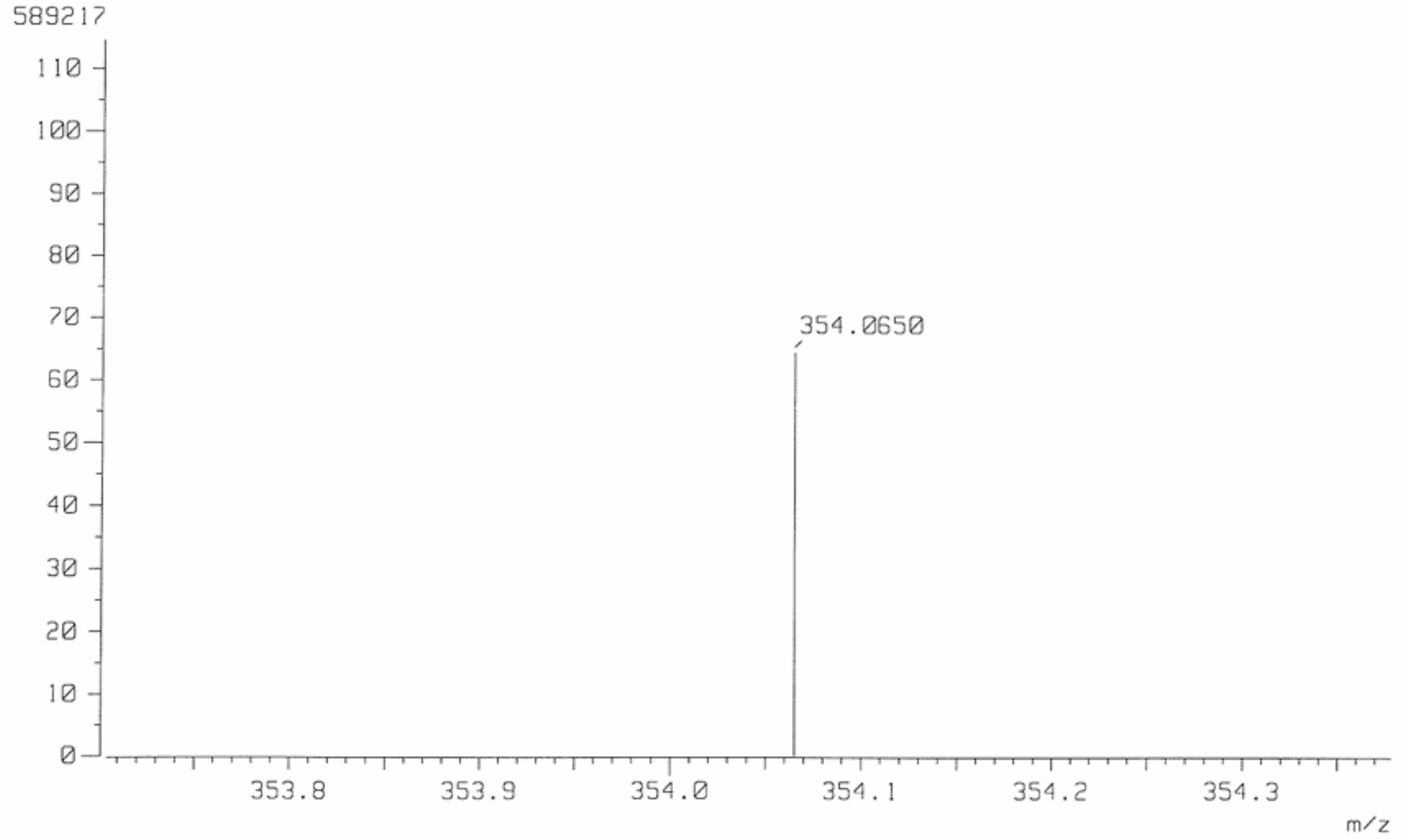

Compound 5i

```
[ Elemental Composition ]                                                    Page: 1
Data   : EI-38-Code-LR-107-F2-027      Date : 04-Jul-2026 10:12
Sample: High resolution GC Mass (JMS-700)
Note : SMLab-Mass Analysis & Research Center
Inlet : Direct                         Ion Mode : EI+
RT : 0.62 min                          Scan#: (19,20)
Elements : C 17/0, H 14/0, Br 2/0, F 1/0, N 2/0, O 3/0, S 1/0
Mass Tolerance      : 1000ppm, 3mmu if m/z < 3, 5mmu if m/z > 5
Unsaturation (U.S.) : -0.5 - 80.0

Observed m/z  Int%    Err[ppm / mmu]    U.S.  Composition
  473.9047    52.1      -0.3 /  -0.2    11.0  C 16 H 13 Br 2 F N 2 O 2 S
```

```
[ Mass Spectrum ]
Data   : EI-38-Code-LR-107-F2-027     Date : 04-Jul-2026 10:12
Sample: High resolution GC Mass (JMS-700)
Note : SMLab-Mass Analysis & Research Center
Inlet : Direct                         Ion Mode : EI+
Spectrum Type : Normal Ion [MF-Linear]
RT : 0.62 min           Scan# : (19,20)
BP : m/z 475.9027       Int.  : 40.35
Output m/z range : 473.5868 to 474.1896      Cut Level : 37.50 %
```

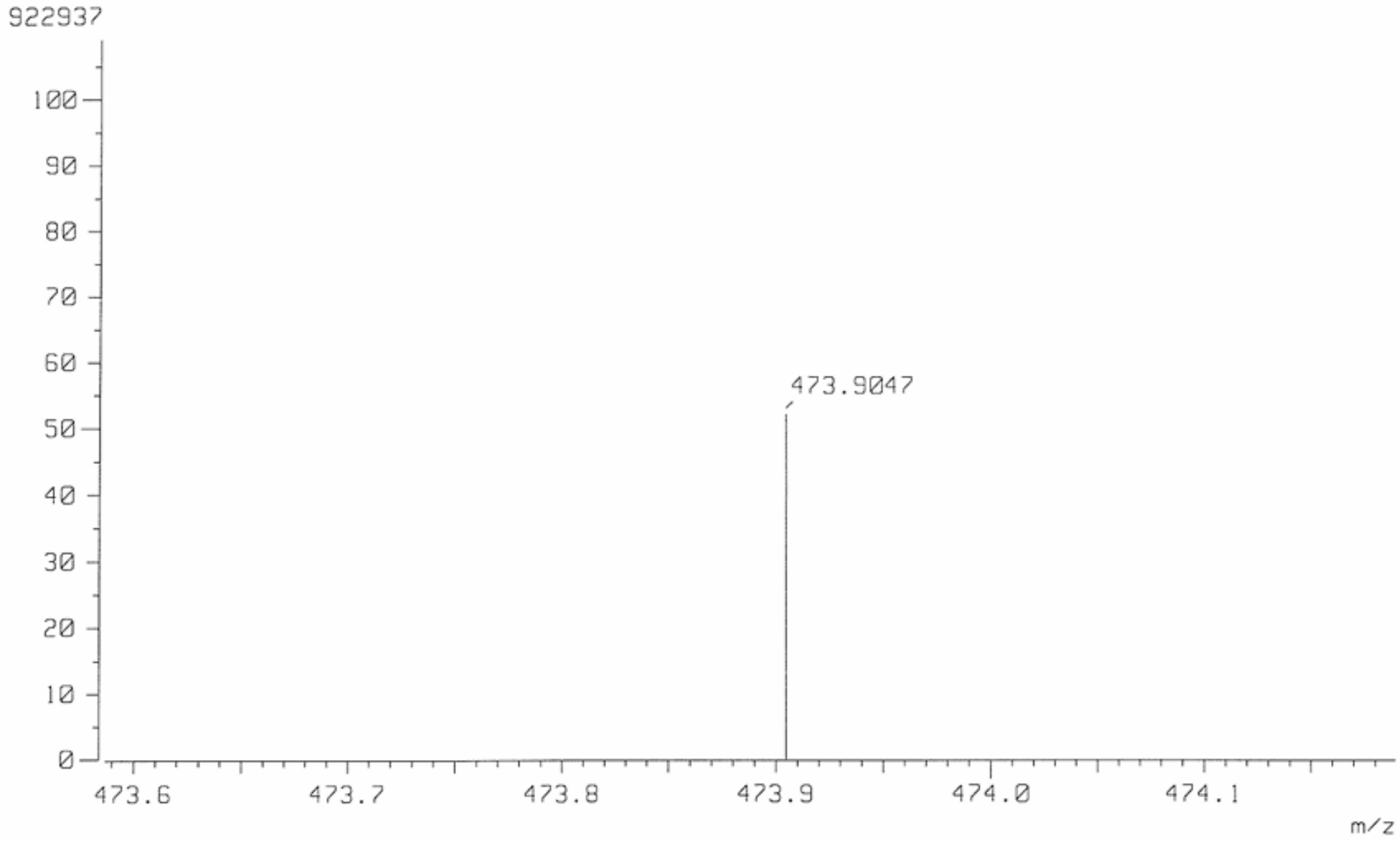

Compound 5j

[Elemental Composition] Page: 1
Data : EI-15-HS-261-024 Date : 03-Jul-2026 16:01
Sample: High resolution GC Mass (JMS-700)
Note : SMLab-Mass Analysis & Research Center
Inlet : Direct Ion Mode : EI+
RT : 1.27 min Scan#: 39+40+39
Elements : C 18/0, H 15/0, F 3/0, N 2/0, O 3/0, S 2/0
Mass Tolerance : 1000ppm, 3mmu if m/z < 3, 5mmu if m/z > 5
Unsaturation (U.S.) : -0.5 - 80.0

Observed m/z	Int%	Err[ppm / mmu]	U.S.	Composition
354.0650	96.6	+0.1 / +0.0	11.0	C 16 H 13 F 3 N 2 O 2 S

[Mass Spectrum]
Data : EI-15-HS-261-024 Date : 03-Jul-2026 16:01
Sample: High resolution GC Mass (JMS-700)
Note : SMLab-Mass Analysis & Research Center
Inlet : Direct Ion Mode : EI+
Spectrum Type : Normal Ion [MF-Linear]
RT : 1.27 min Scan# : 39+40+39
BP : m/z 330.9821 Int. : 40.23
Output m/z range : 353.6933 to 354.3838 Cut Level : 69.12 %

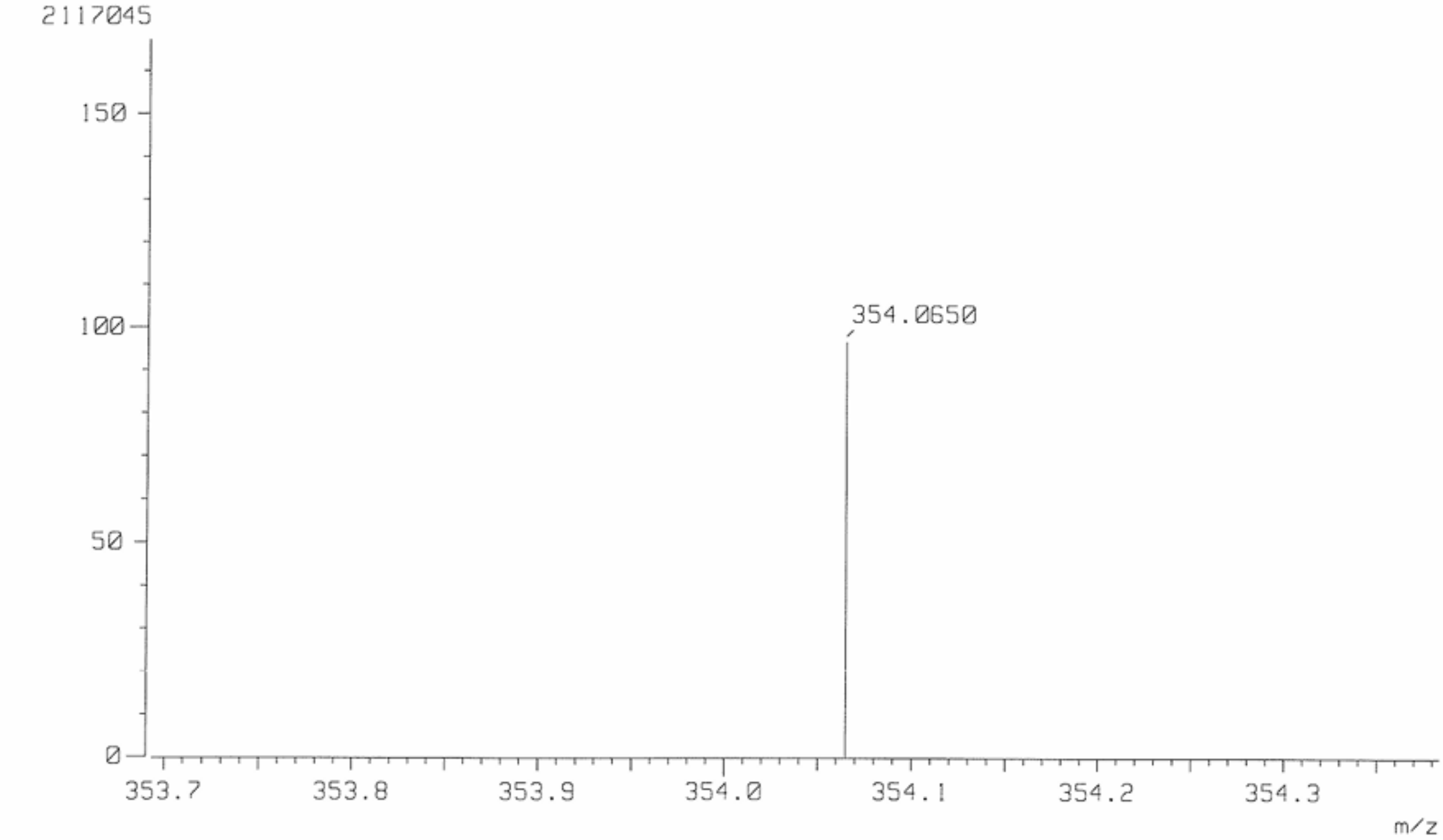

Compound 5k

```
[ Elemental Composition ]                                          Page: 1
Data   : EI-8-HS-237-019              Date : 03-Jul-2026 15:54
Sample: High resolution GC Mass (JMS-700)
Note : SMLab-Mass Analysis & Research Center
Inlet : Direct                        Ion Mode : EI+
RT : 0.57 min                         Scan#: 18
Elements : C 17/0, H 14/0, F 3/0, N 2/0, O 2/0, S 1/0
Mass Tolerance      : 1000ppm, 3mmu if m/z < 3, 5mmu if m/z > 5
Unsaturation (U.S.) : -0.5 - 80.0

Observed m/z  Int%   Err[ppm / mmu]   U.S.  Composition
  354.0647   100.0     -0.8 /  -0.3   11.0  C 16 H 13 F 3 N 2 O 2 S
```

```
[ Mass Spectrum ]
Data   : EI-8-HS-237-019              Date : 03-Jul-2026 15:54
Sample: High resolution GC Mass (JMS-700)
Note : SMLab-Mass Analysis & Research Center
Inlet : Direct                        Ion Mode : EI+
Spectrum Type : Normal Ion [MF-Linear]
RT : 0.57 min           Scan# : 18
BP : m/z 354.0647       Int.  : 65.15
Output m/z range : 353.7457 to 354.3908       Cut Level : 39.33 %
```

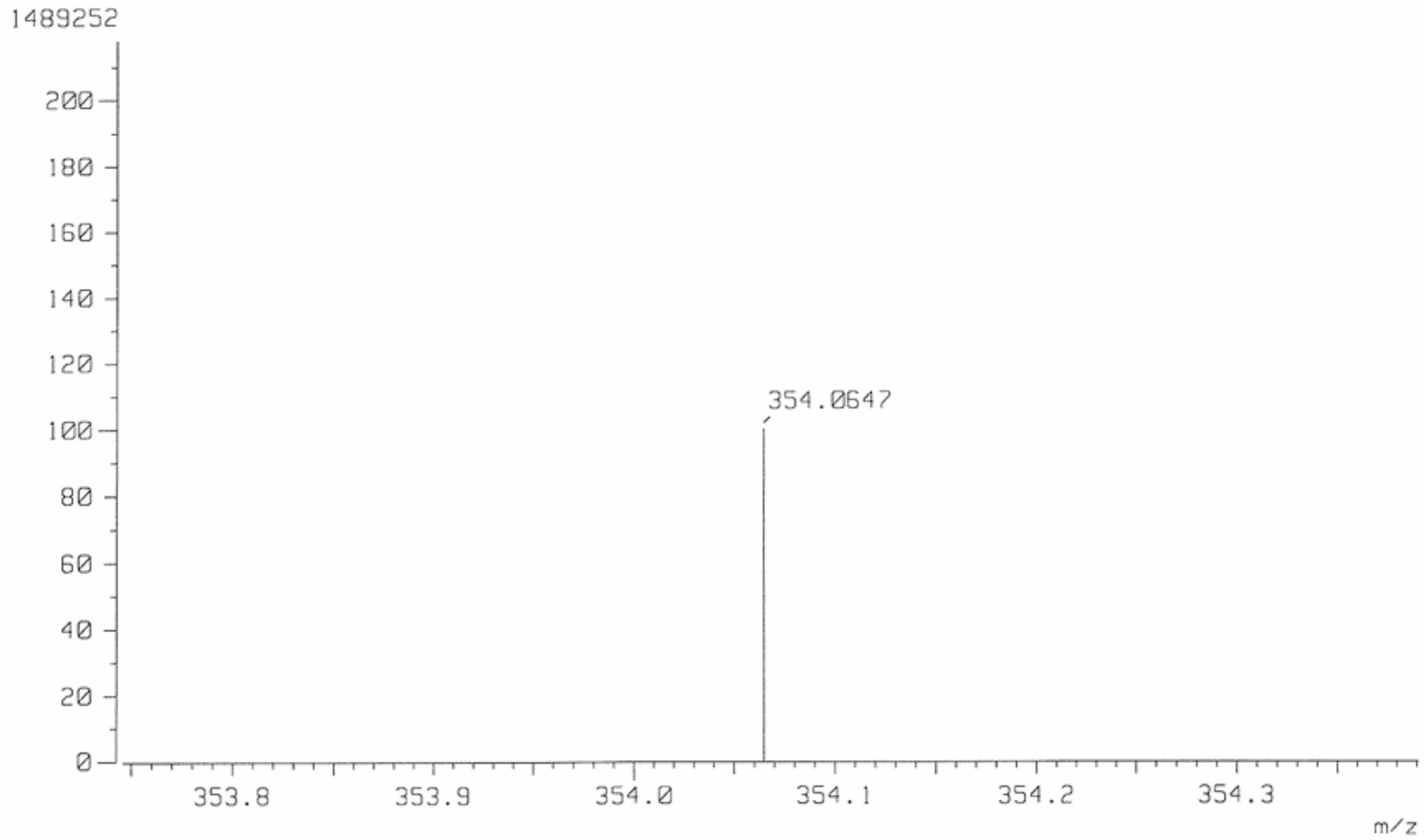

Compound 5l

[Elemental Composition]　　Page: 1
Data : EI-7-HS-236-017　　Date : 03-Jul-2026 15:50
Sample: High resolution GC Mass (JMS-700)
Note : SMLab-Mass Analysis & Research Center
Inlet : Direct　　Ion Mode : EI+
RT : 1.32 min　　Scan#: (38,43)
Elements : C 17/0, H 15/0, F 3/0, N 2/0, O 2/0, S 1/0
Mass Tolerance : 1000ppm, 3mmu if m/z < 3, 5mmu if m/z > 5
Unsaturation (U.S.) : -0.5 - 80.0

Observed m/z	Int%	Err[ppm / mmu]	U.S.	Composition
354.0650	100.0	+0.0 / +0.0	11.0	C 16 H 13 F 3 N 2 O 2 S

[Mass Spectrum]
Data : EI-7-HS-236-017　　Date : 03-Jul-2026 15:50
Sample: High resolution GC Mass (JMS-700)
Note : SMLab-Mass Analysis & Research Center
Inlet : Direct　　Ion Mode : EI+
Spectrum Type : Normal Ion [MF-Linear]
RT : 1.32 min　　Scan# : (38,43)
BP : m/z 354.0650　　Int. : 20.58
Output m/z range : 353.6934 to 354.3286　　Cut Level : 62.76 %

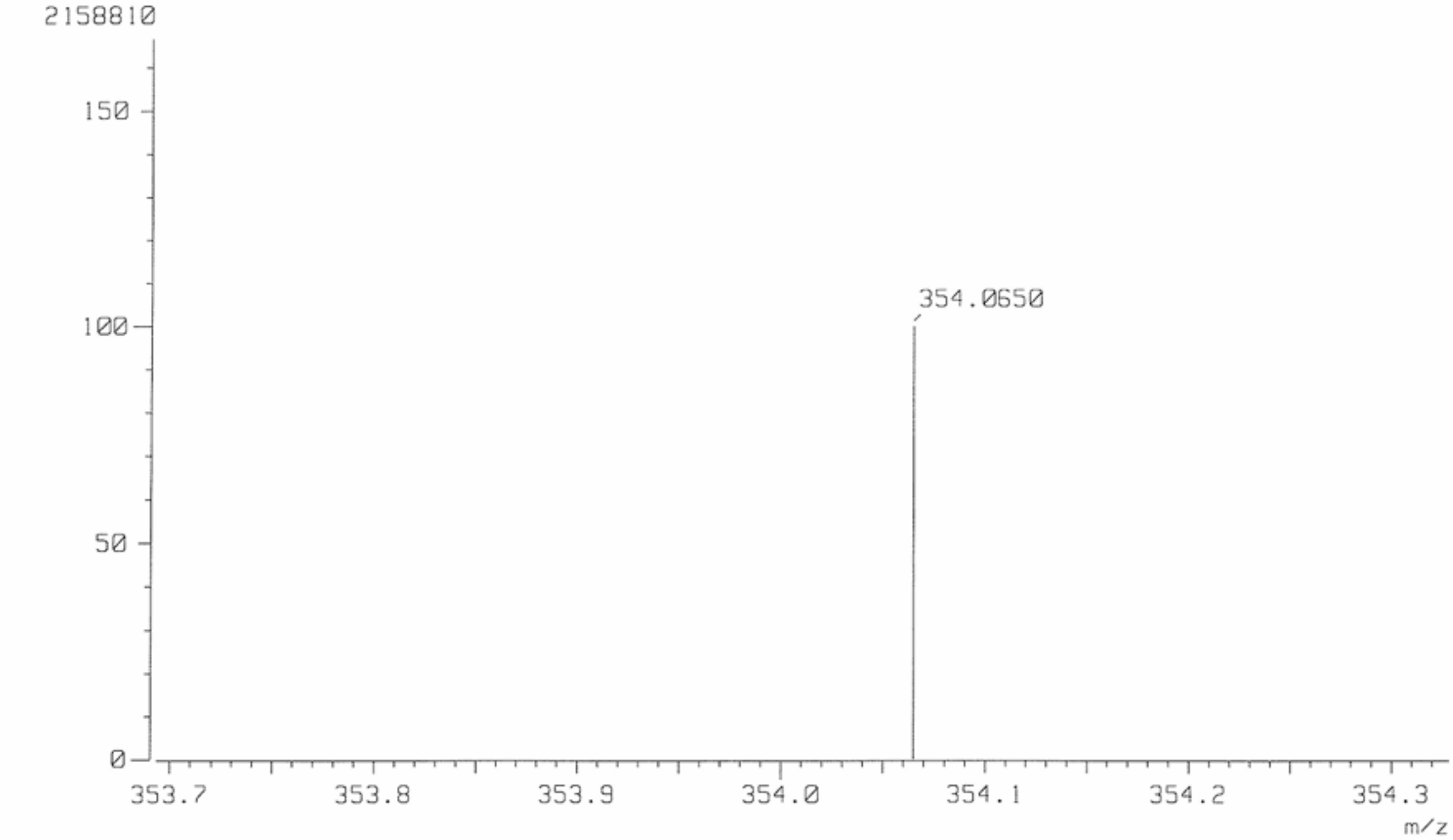

Compound 5m

[Elemental Composition] Page: 1
Data : EI-21-HS-268-001 Date : 04-Jul-2026 08:52
Sample: High resolution GC Mass (JMS-700)
Note : SMLab-Mass Analysis & Research Center
Inlet : Direct Ion Mode : EI+
RT : 1.74 min Scan#: 53
Elements : C 17/0, H 14/0, Cl 2/0, F 1/0, N 2/0, O 3/0, S 1/0
Mass Tolerance : 1000ppm, 3mmu if m/z < 3, 5mmu if m/z > 5
Unsaturation (U.S.) : -0.5 - 80.0

Observed m/z	Int%	Err[ppm / mmu]	U.S.	Composition
386.0057	100.0	-0.5 / -0.2	11.0	C 16 H 13 Cl 2 F N 2 O 2 S

[Mass Spectrum]
Data : EI-21-HS-268-001 Date : 04-Jul-2026 08:52
Sample: High resolution GC Mass (JMS-700)
Note : SMLab-Mass Analysis & Research Center
Inlet : Direct Ion Mode : EI+
Spectrum Type : Normal Ion [MF-Linear]
RT : 1.74 min Scan# : 53
BP : m/z 386.0057 Int. : 86.93
Output m/z range : 385.6309 to 386.3767 Cut Level : 80.15 %

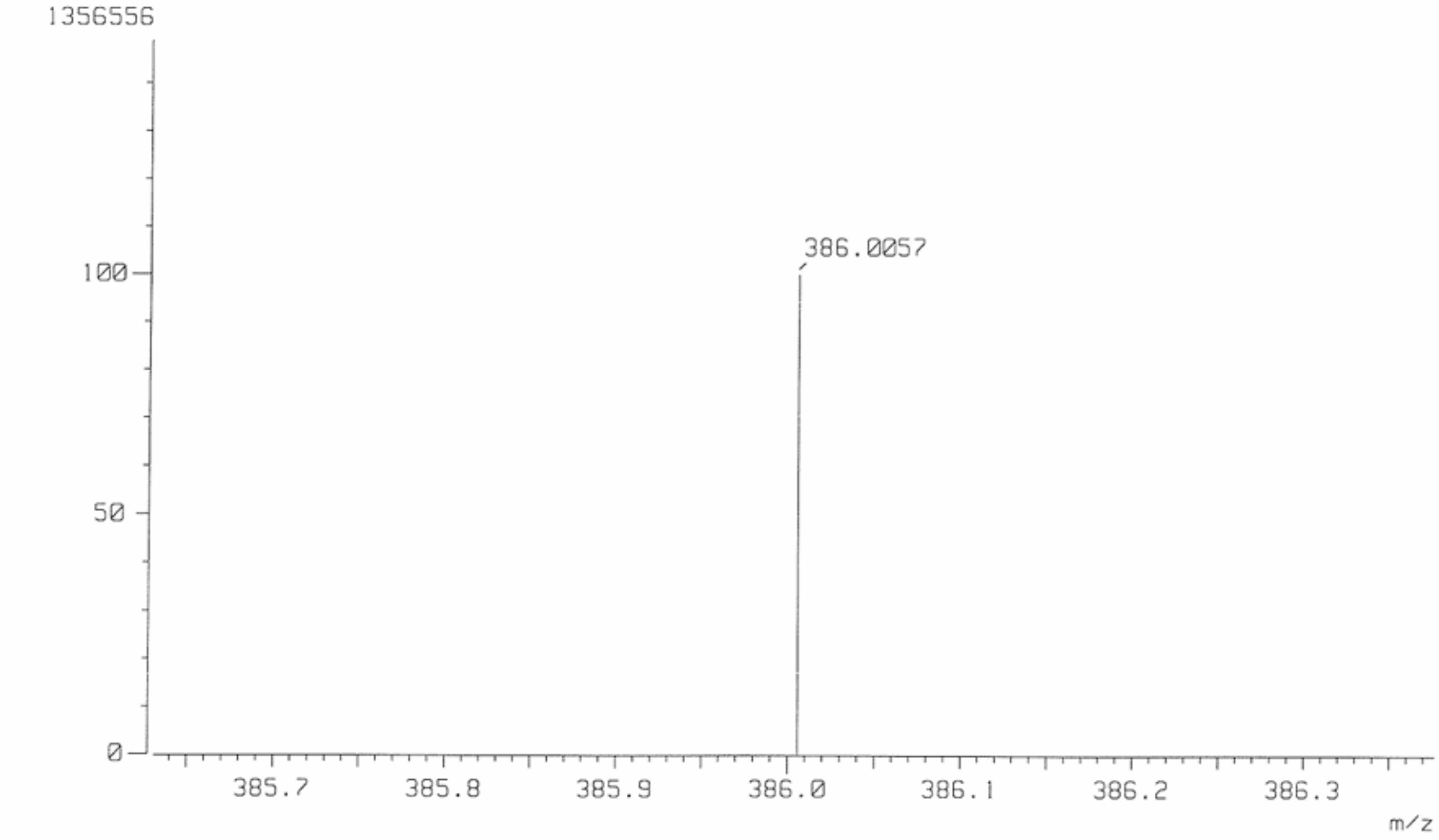

Compound 5n

[Elemental Composition]   Page: 1
Data   : EI-HS288-HR006   Date : 04-Jul-2026 10:46
Sample: High resolution GC Mass (JMS-700)
Note : SMLab-Mass Analysis & Research Center
Inlet : Direct   Ion Mode : EI+
RT : 1.22 min   Scan#: (37,38)
Elements : C 20/0, H 20/0, F 1/0, N 2/0, O 3/0, S 1/0
Mass Tolerance   : 1000ppm, 3mmu if m/z < 3, 5mmu if m/z > 5
Unsaturation (U.S.) : -0.5 - 80.0

Observed m/z	Int%	Err[ppm / mmu]	U.S.	Composition
346.1153	7.4	+0.5 / +0.2	11.0	C 18 H 19 F N 2 O 2 S

[Mass Spectrum]
Data   : EI-HS288-HR006   Date : 04-Jul-2026 10:46
Sample: High resolution GC Mass (JMS-700)
Note : SMLab-Mass Analysis & Research Center
Inlet : Direct   Ion Mode : EI+
Spectrum Type : Normal Ion [MF-Linear]
RT : 1.22 min   Scan# : (37,38)
BP : m/z 322.1659   Int.   : 44.68
Output m/z range : 345.8145 to 346.4016   Cut Level : 7.35 %

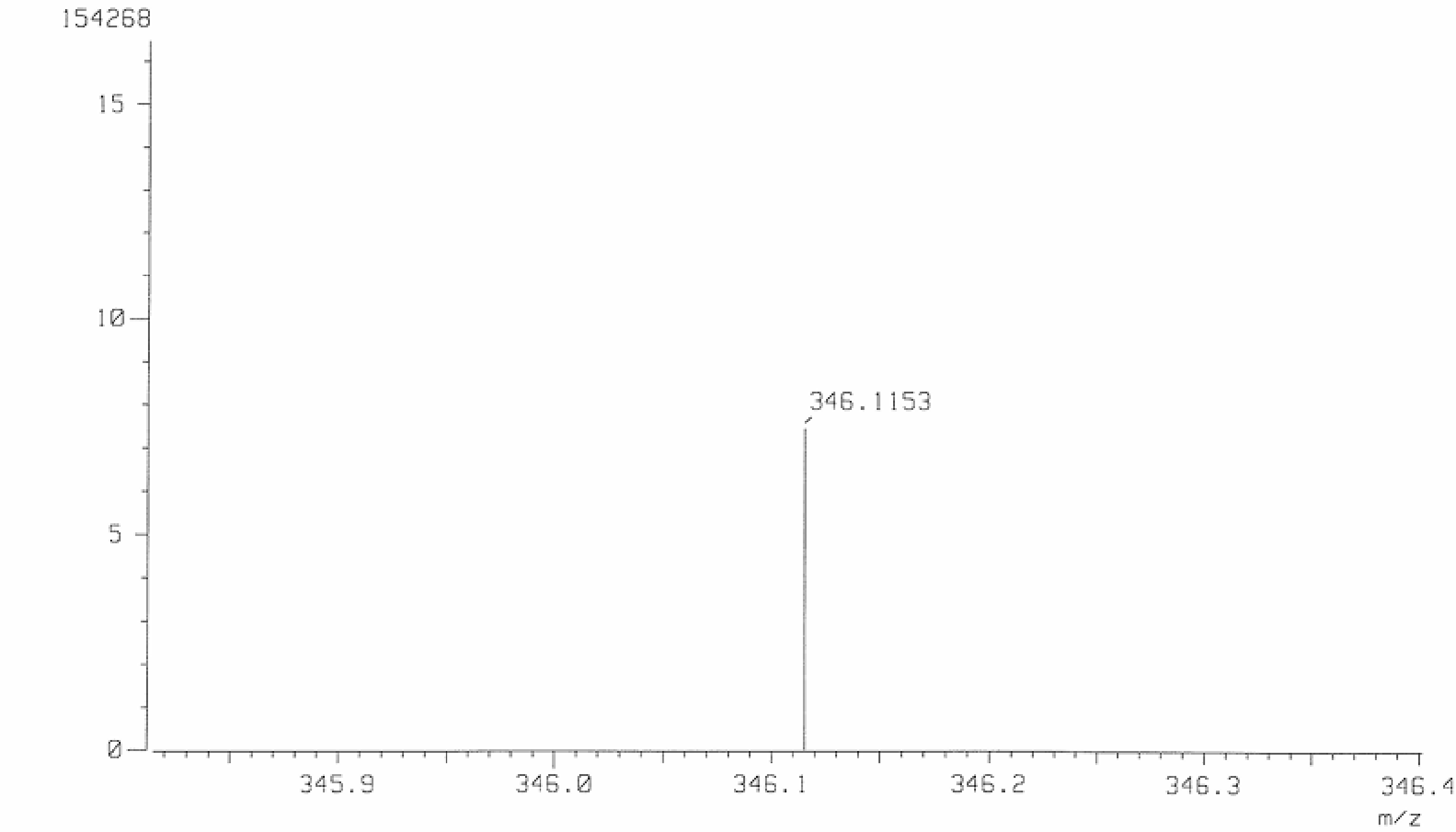

Compound 5o

[Elemental Composition] Page: 1

Data : EI-27-HS-294-004 Date : 04-Jul-2026 10:18
Sample: High resolution GC Mass (JMS-700)
Note : SMLab-Mass Analysis & Research Center
Inlet : Direct Ion Mode : EI+
RT : 1.02 min Scan#: (30,33)
Elements : C 20/0, H 20/0, F 1/0, N 2/0, O 6/0, S 1/0
Mass Tolerance : 1000ppm, 3mmu if m/z < 3, 5mmu if m/z > 5
Unsaturation (U.S.) : -0.5 - 80.0

Observed m/z	Int%	Err[ppm / mmu]	U.S.	Composition
410.0946	100.0	-0.4 / -0.2	11.0	C 18 H 19 F N 2 O 6 S

[Mass Spectrum]
Data : EI-27-HS-294-004 Date : 04-Jul-2026 10:18
Sample: High resolution GC Mass (JMS-700)
Note : SMLab-Mass Analysis & Research Center
Inlet : Direct Ion Mode : EI+
Spectrum Type : Normal Ion [MF-Linear]
RT : 1.02 min Scan# : (30,33)
BP : m/z 410.0946 Int. : 74.04
Output m/z range : 409.7812 to 410.3832 Cut Level : 72.28 %

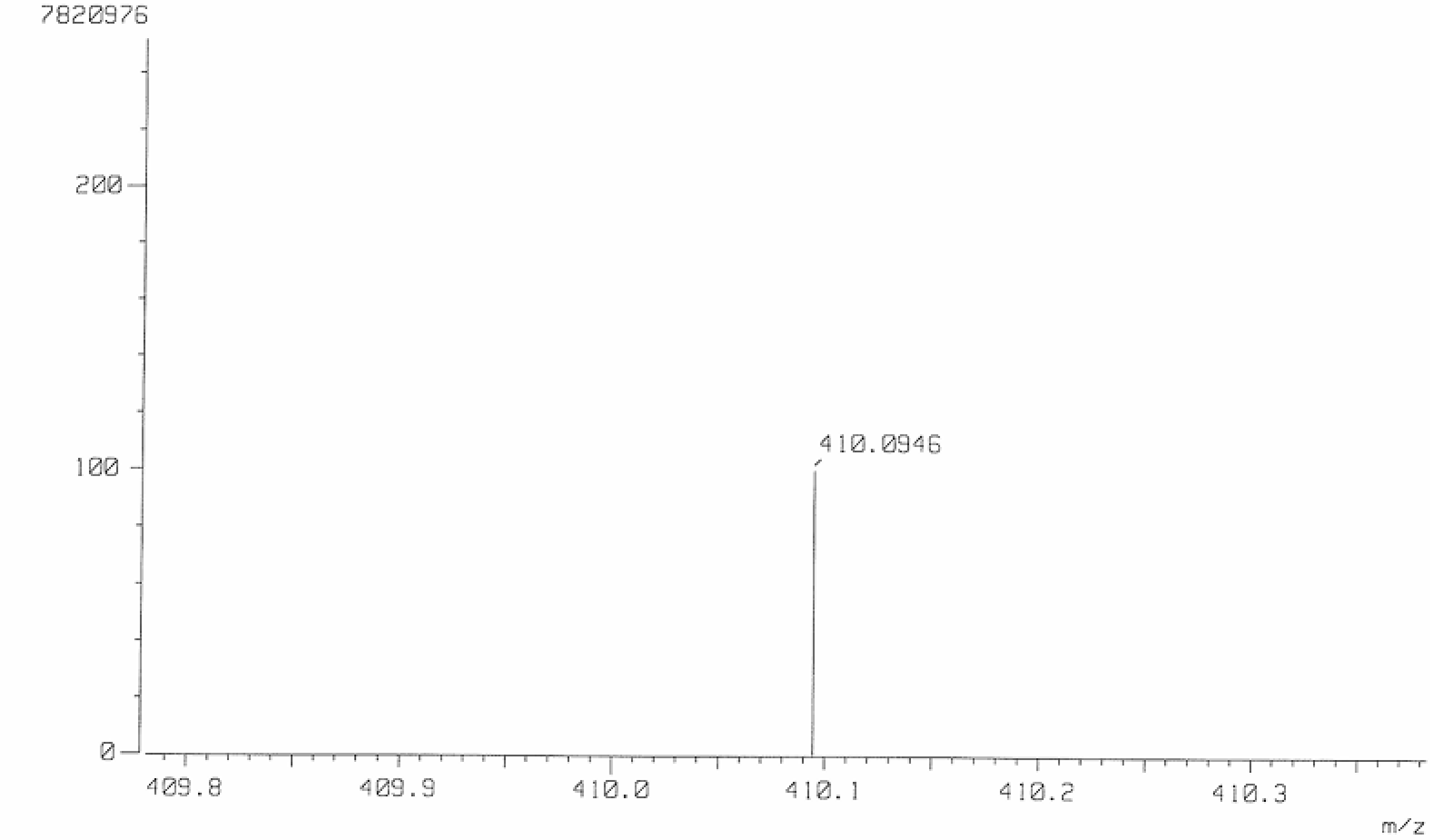

Compound 5p

[Elemental Composition]

Page: 1

Data : EI-26-HS-293-003 Date : 04-Jul-2026 10:16
Sample: High resolution GC Mass (JMS-700)
Note : SMLab-Mass Analysis & Research Center
Inlet : Direct Ion Mode : EI+
RT : 0.35 min Scan#: (11,12)+10+7
Elements : C 15/0, H 9/0, Cl 4/0, F 1/0, N 2/0, O 3/0, S 1/0
Mass Tolerance : 1000ppm, 3mmu if m/z < 3, 5mmu if m/z > 5
Unsaturation (U.S.) : -0.5 - 80.0

Observed m/z	Int%	Err[ppm / mmu]	U.S.	Composition
425.8961	75.0	-1.4 / -0.6	11.0	C 14 H 7 Cl 4 F N 2 O 2 S

[Mass Spectrum]
Data : EI-26-HS-293-003 Date : 04-Jul-2026 10:16
Sample: High resolution GC Mass (JMS-700)
Note : SMLab-Mass Analysis & Research Center
Inlet : Direct Ion Mode : EI+
Spectrum Type : Normal Ion [MF-Linear]
RT : 0.35 min Scan# : (11,12)+10+7
BP : m/z 427.8942 Int. : 72.72
Output m/z range : 425.4651 to 426.2671 Cut Level : 51.47 %

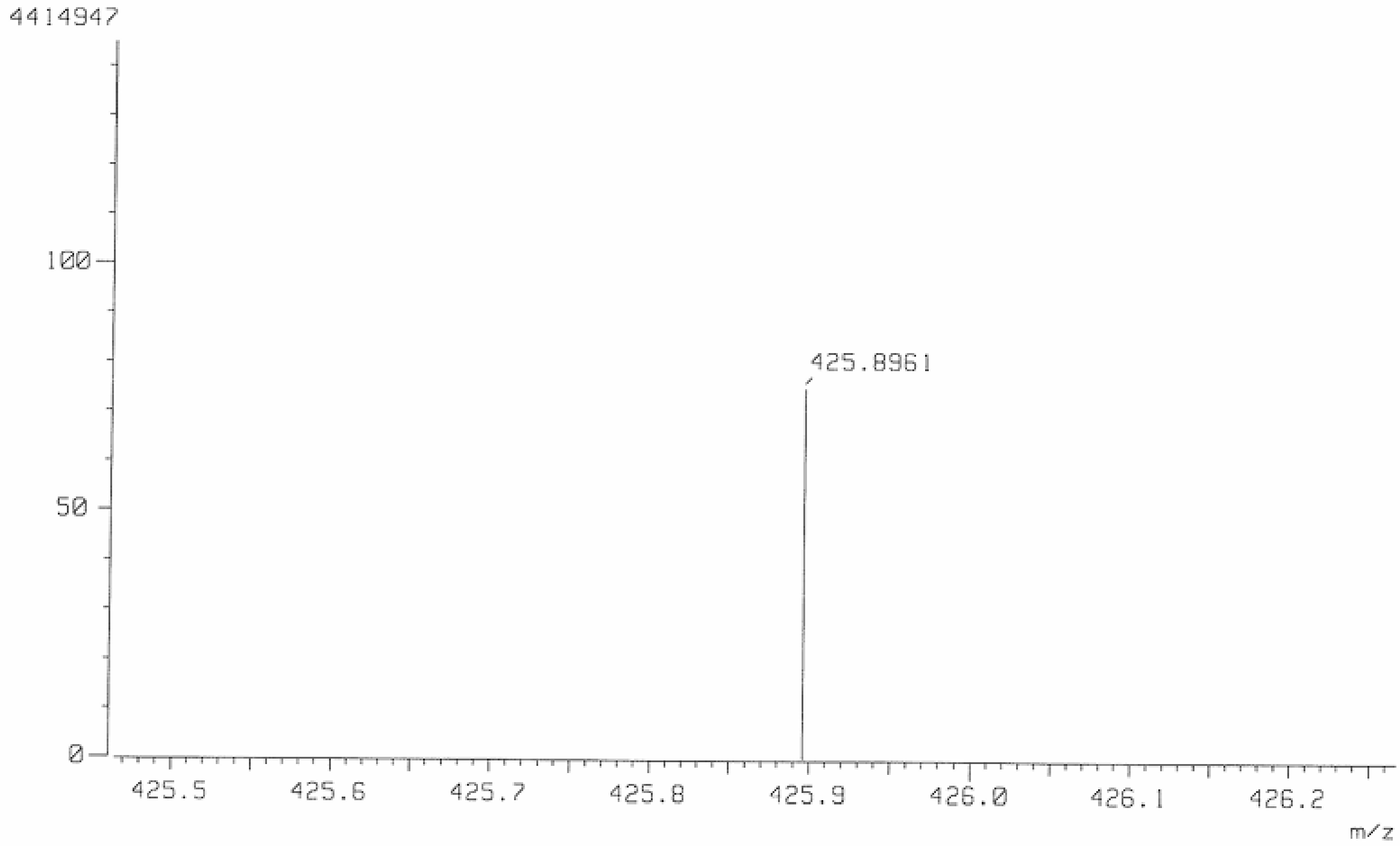

Compound 5q and 5q'

[Elemental Composition]
Page: 1
Data : EI-4-HS-345-P-010 Date : 16-Sep-2026 15:26
Sample: High resolution GC Mass (JMS-700)
Note : SMLab-Mass Analysis & Research Center
Inlet : Direct Ion Mode : EI+
RT : 0.14 min Scan#: 5+8
Elements : C 18/0, H 20/0, F 1/0, N 2/0, O 2/0, S 1/0
Mass Tolerance : 1000ppm, 3mmu if m/z < 3, 5mmu if m/z > 5
Unsaturation (U.S.) : -0.5 - 80.0

Observed m/z	Int%	Err[ppm / mmu]	U.S.	Composition
332.0991	100.0	-1.3 / -0.4	11.0	C 17 H 17 F N 2 O 2 S

[Mass Spectrum]
Data : EI-4-HS-345-P-010 Date : 16-Sep-2026 15:26
Sample: High resolution GC Mass (JMS-700)
Note : SMLab-Mass Analysis & Research Center
Inlet : Direct Ion Mode : EI+
Spectrum Type : Normal Ion [MF-Linear]
RT : 0.14 min Scan# : 5+8
BP : m/z 332.0991 Int. : 247.23
Output m/z range : 331.7353 to 332.4460 Cut Level : 72.79 %

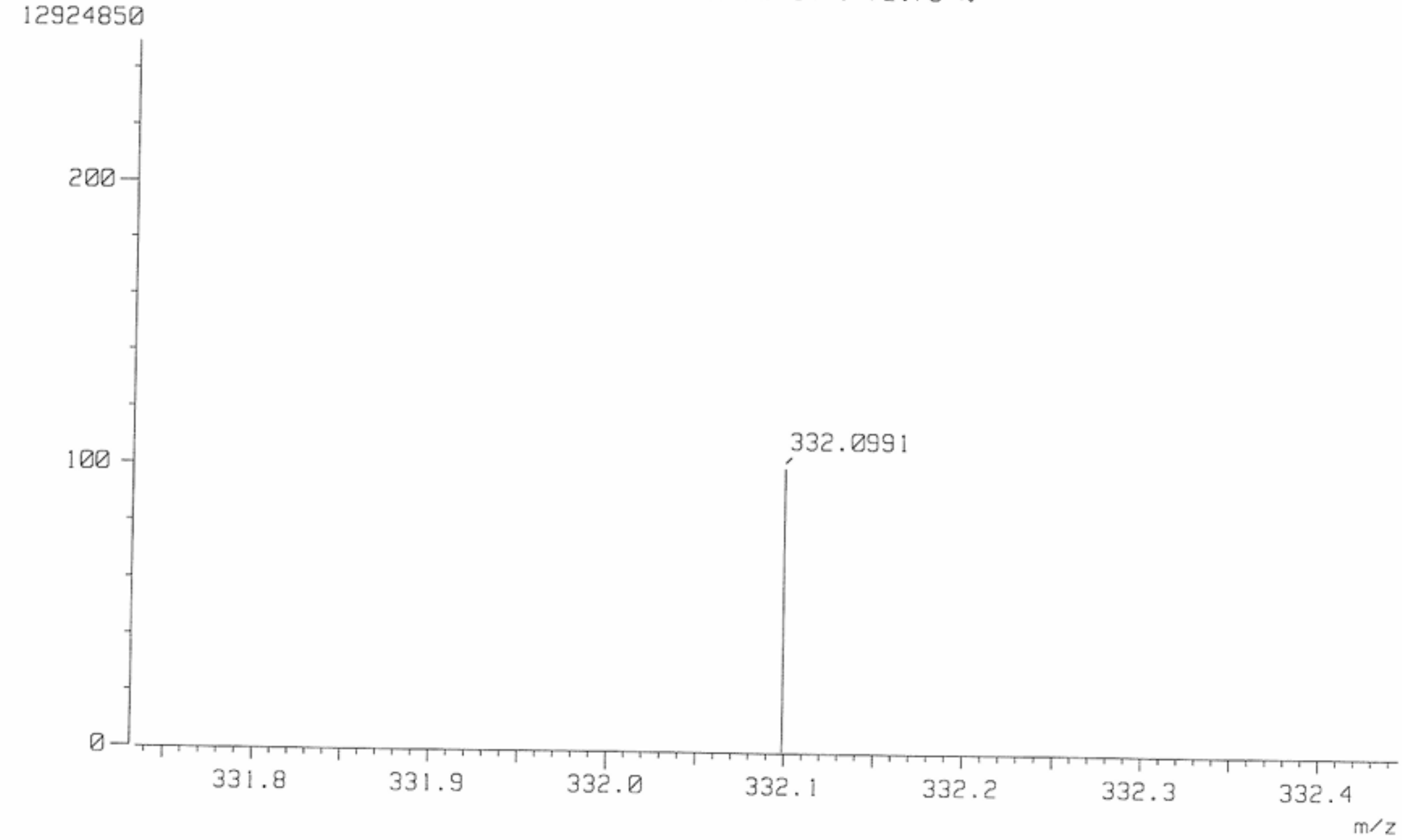

Compound 6a

[Elemental Composition]   Page: 1

Data : EI-3-HS-335-P-014   Date : 16-Sep-2026 15:45

Sample: High resolution GC Mass (JMS-700)

Note : SMLab-Mass Analysis & Research Center

Inlet : Direct   Ion Mode : EI+

RT : 0.30 min   Scan#: 10

Elements : C 22/0, H 24/0, N 3/0, O 3/0, S 1/0

Mass Tolerance : 1000ppm, 3mmu if m/z < 3, 5mmu if m/z > 5

Unsaturation (U.S.) : -0.5 - 80.0

Observed m/z	Int%	Err[ppm / mmu]	U.S.	Composition
369.1507	100.0	-1.0 / -0.4	12.0	C 20 H 23 N 3 O 2 S

[Mass Spectrum]

Data : EI-3-HS-335-P-014   Date : 16-Sep-2026 15:45

Sample: High resolution GC Mass (JMS-700)

Note : SMLab-Mass Analysis & Research Center

Inlet : Direct   Ion Mode : EI+

Spectrum Type : Normal Ion [MF-Linear]

RT : 0.30 min   Scan# : 10

BP : m/z 369.1507   Int. : 125.47

Output m/z range : 368.8396 to 369.4700   Cut Level : 70.59 %

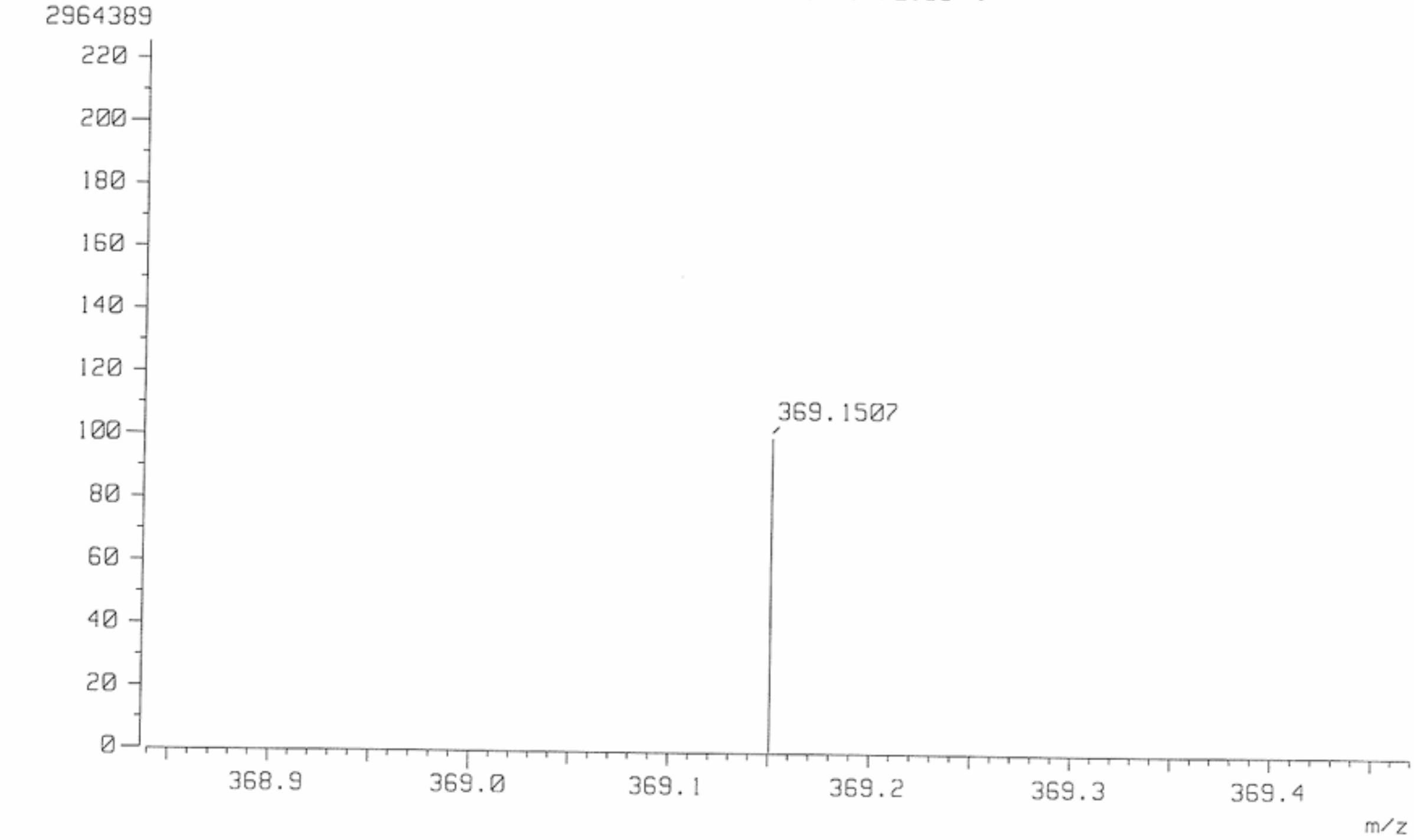

Compound 6b

[Elemental Composition]　　　　　　　　　　　　　　　　　　　　Page: 1
Data : EI-HS272-re-001　　　　　　Date : 20-Jul-2026 12:56
Sample: High resolution GC Mass (JMS-700)
Note : SMLab-Mass Analysis & Research Center
Inlet : Direct　　　　　　　　　　Ion Mode : EI+
RT : 2.70 min　　　　　　　　　　Scan#: 82
Elements : C 29/0, H 40/0, N 4/0, O 7/0, S 1/0
Mass Tolerance : 1000ppm, 3mmu if m/z < 3, 5mmu if m/z > 5
Unsaturation (U.S.) : -0.5 - 80.0

Observed m/z	Int%	Err[ppm / mmu]	U.S.	Composition
558.2509	42.7	-0.6 / -0.4	13.0	C 28 H 38 N 4 O 6 S

[Mass Spectrum]
Data : EI-HS272-re-001　　　　　　Date : 20-Jul-2026 12:56
Sample: High resolution GC Mass (JMS-700)
Note : SMLab-Mass Analysis & Research Center
Inlet : Direct　　　　　　　　　　Ion Mode : EI+
Spectrum Type : Normal Ion [MF-Linear]
RT : 2.70 min　　　　Scan# : 82
BP : m/z 508.2835　　　Int. : 69.90
Output m/z range : 557.9486 to 558.5346　　　Cut Level : 30.88 %

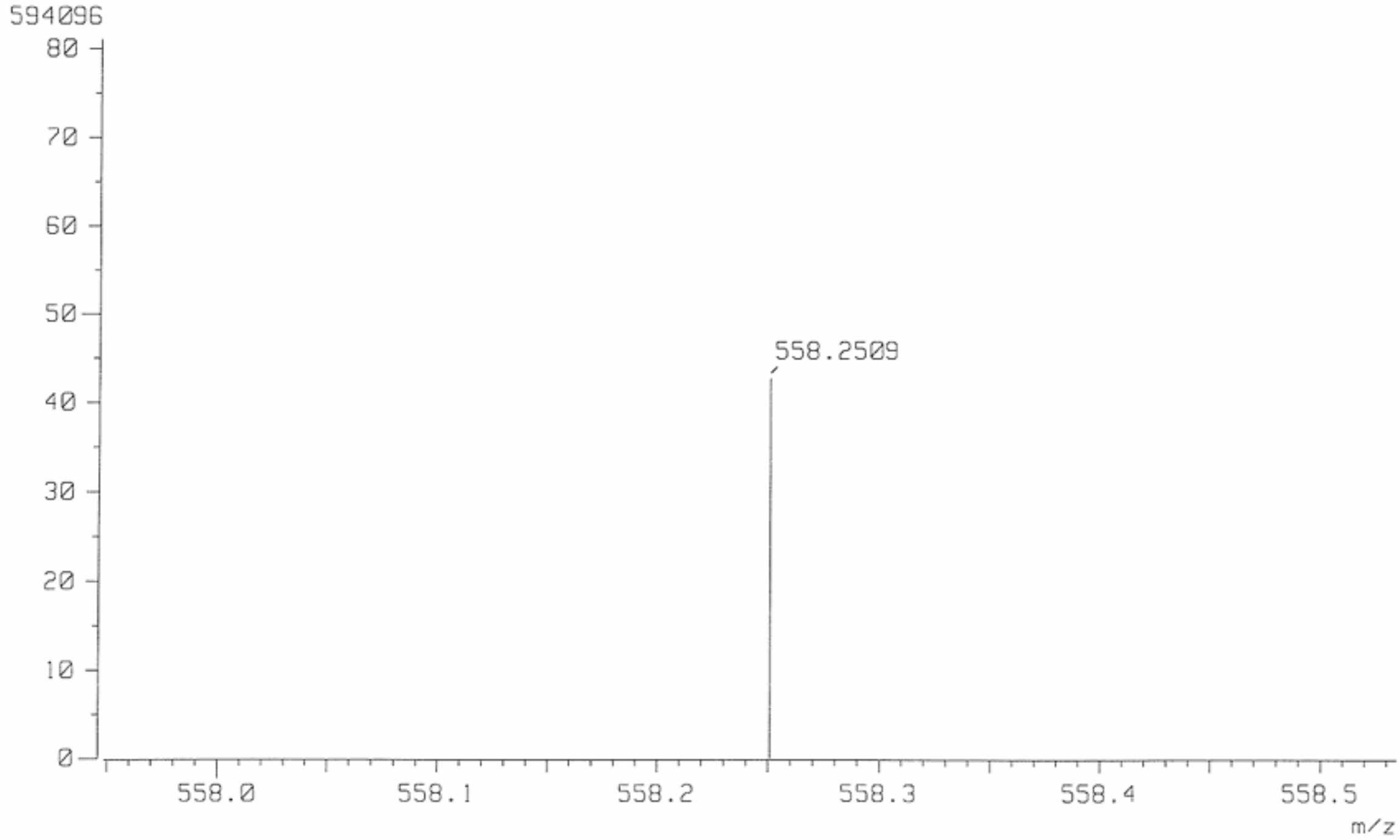

Compound 6c

[Elemental Composition]
Page: 1
Data : EI-10-HS-246-004
Date : 03-Jul-2026 16:30
Sample: High resolution GC Mass (JMS-700)
Note : SMLab-Mass Analysis & Research Center
Inlet : Direct
Ion Mode : EI+
RT : 0.29 min
Scan#: (9,10)+18
Elements : C 33/0, H 36/0, N 3/0, O 8/0, S 1/0
Mass Tolerance : 1000ppm, 3mmu if m/z < 3, 5mmu if m/z > 5
Unsaturation (U.S.) : -0.5 - 80.0

Observed m/z	Int%	Err[ppm / mmu]	U.S.	Composition
593.2191	100.0	-0.8 / -0.5	17.0	C 31 H 35 N 3 O 7 S

[Mass Spectrum]
Data : EI-10-HS-246-004
Date : 03-Jul-2026 16:30
Sample: High resolution GC Mass (JMS-700)
Note : SMLab-Mass Analysis & Research Center
Inlet : Direct
Ion Mode : EI+
Spectrum Type : Normal Ion [MF-Linear]
RT : 0.29 min
Scan# : (9,10)+18
BP : m/z 593.2191
Int. : 24.80
Output m/z range : 592.8818 to 593.5088
Cut Level : 48.55 %

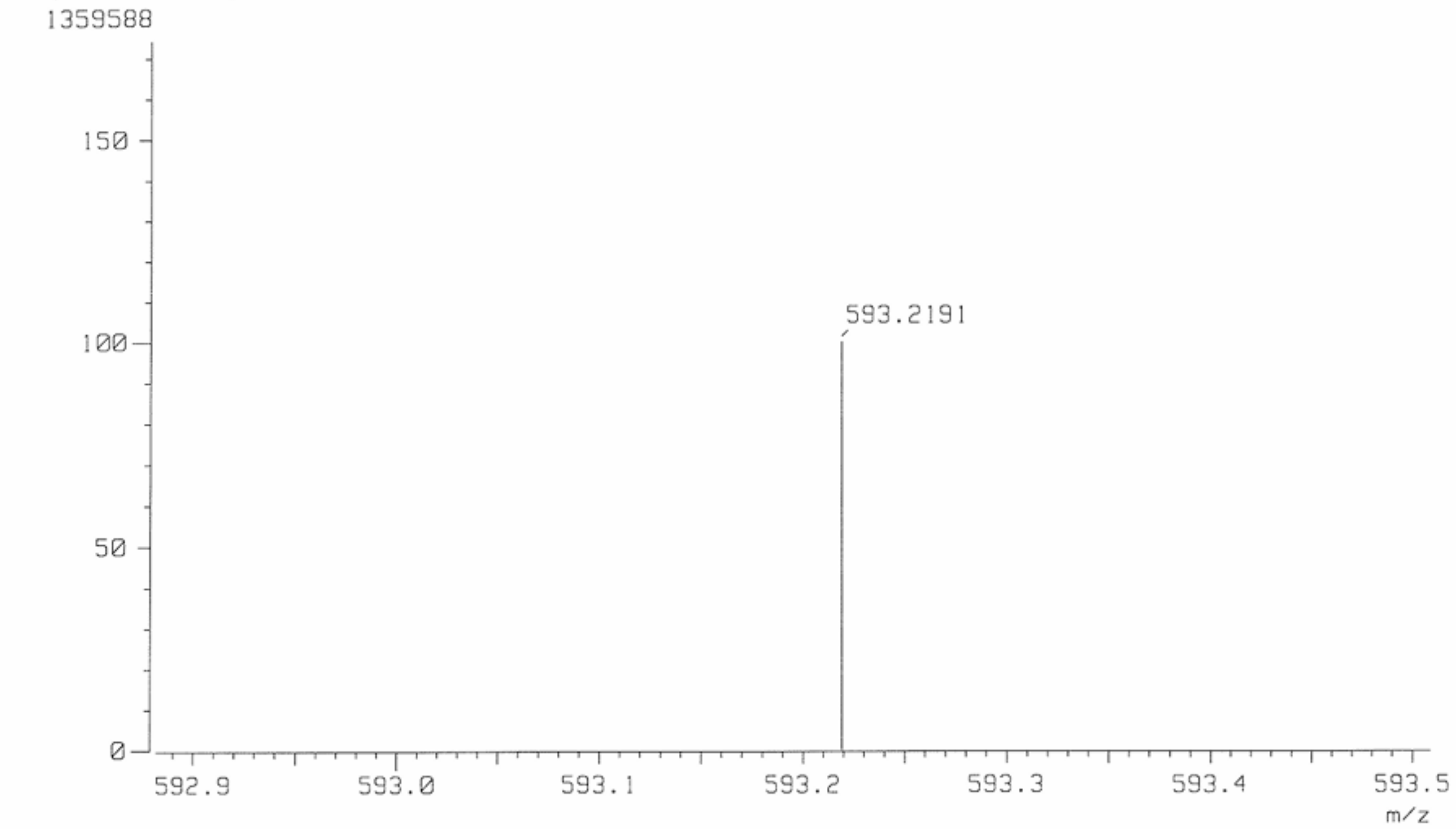

Compound 6d

```
[ Elemental Composition ]                                              Page: 1
Data   : FAB-HS303-R-020                 Date : 19-Jul-2026 11:57
Sample: -
Note : -
Inlet : Direct                           Ion Mode : FAB-
RT : 4.20 min                            Scan#: (84,86)
Elements : C 30/0, H 27/0, N 5/0, O 5/0, S 1/0
Mass Tolerance      : 1000ppm, 3mmu if m/z < 3, 5mmu if m/z > 5
Unsaturation (U.S.) : -0.5 - 80.0

Observed m/z Int%   Err[ppm / mmu]   U.S.  Composition
  556.1662    14.9     +1.3 /  +0.7   20.5  C 29 H 26 N 5 O 5 S
```

```
[ Mass Spectrum ]
Data  : FAB-HS303-R-020              Date : 19-Jul-2026 11:57
Sample: -
Note : -
Inlet : Direct                       Ion Mode : FAB-
Spectrum Type : Normal Ion [MF-Linear]
RT : 4.20 min           Scan# : (84,86)
BP : m/z 459.1310       Int.  : 6.60
Output m/z range : 555.8041 to 556.4866      Cut Level : 9.56 %
```

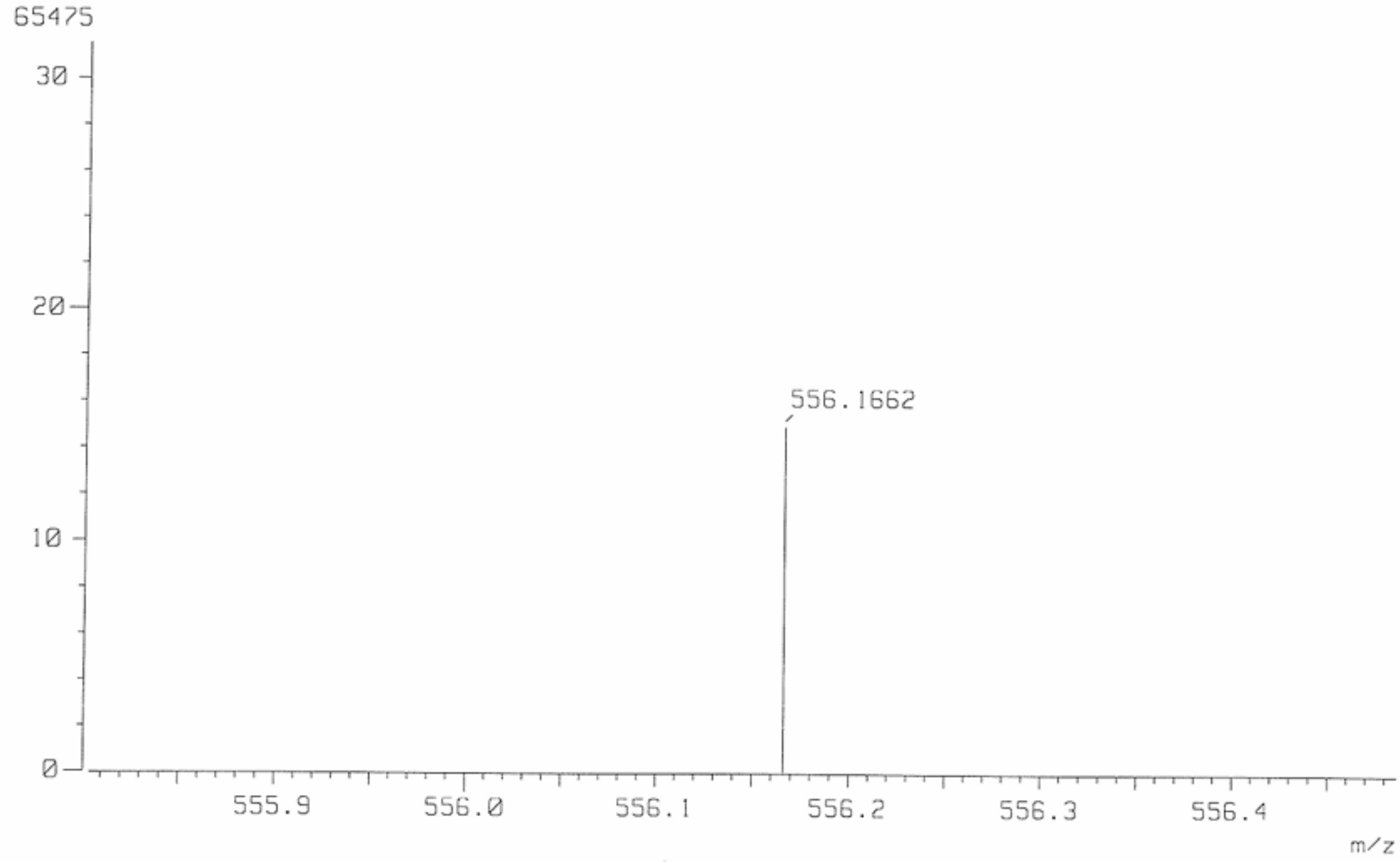

Compound 6e

[Elemental Composition]　　　　　　　　　　　　　　　　Page: 1
Data : EI-11-HS-247-006　　　　Date : 03-Jul-2026 16:36
Sample: High resolution GC Mass (JMS-700)
Note : SMLab-Mass Analysis & Research Center
Inlet : Direct　　　　　　　　Ion Mode : EI+
RT : 0.30 min　　　　　　　　Scan#: 10+10+11
Elements : C 35/0, H 37/0, N 2/0, O 5/0, S 1/0
Mass Tolerance : 1000ppm, 3mmu if m/z < 3, 5mmu if m/z > 5
Unsaturation (U.S.) : -0.5 - 80.0

Observed m/z	Int%	Err[ppm / mmu]	U.S.	Composition
568.2395	100.0	-0.1 / -0.1	19.0	C 34 H 36 N 2 O 4 S

[Mass Spectrum]
Data : EI-11-HS-247-006　　　　Date : 03-Jul-2026 16:36
Sample: High resolution GC Mass (JMS-700)
Note : SMLab-Mass Analysis & Research Center
Inlet : Direct　　　　　　　　Ion Mode : EI+
Spectrum Type : Normal Ion [MF-Linear]
RT : 0.30 min　　　　Scan# : 10+10+11
BP : m/z 568.2395　　　Int. : 71.42
Output m/z range : 567.9261 to 568.5631　　　Cut Level : 71.32 %

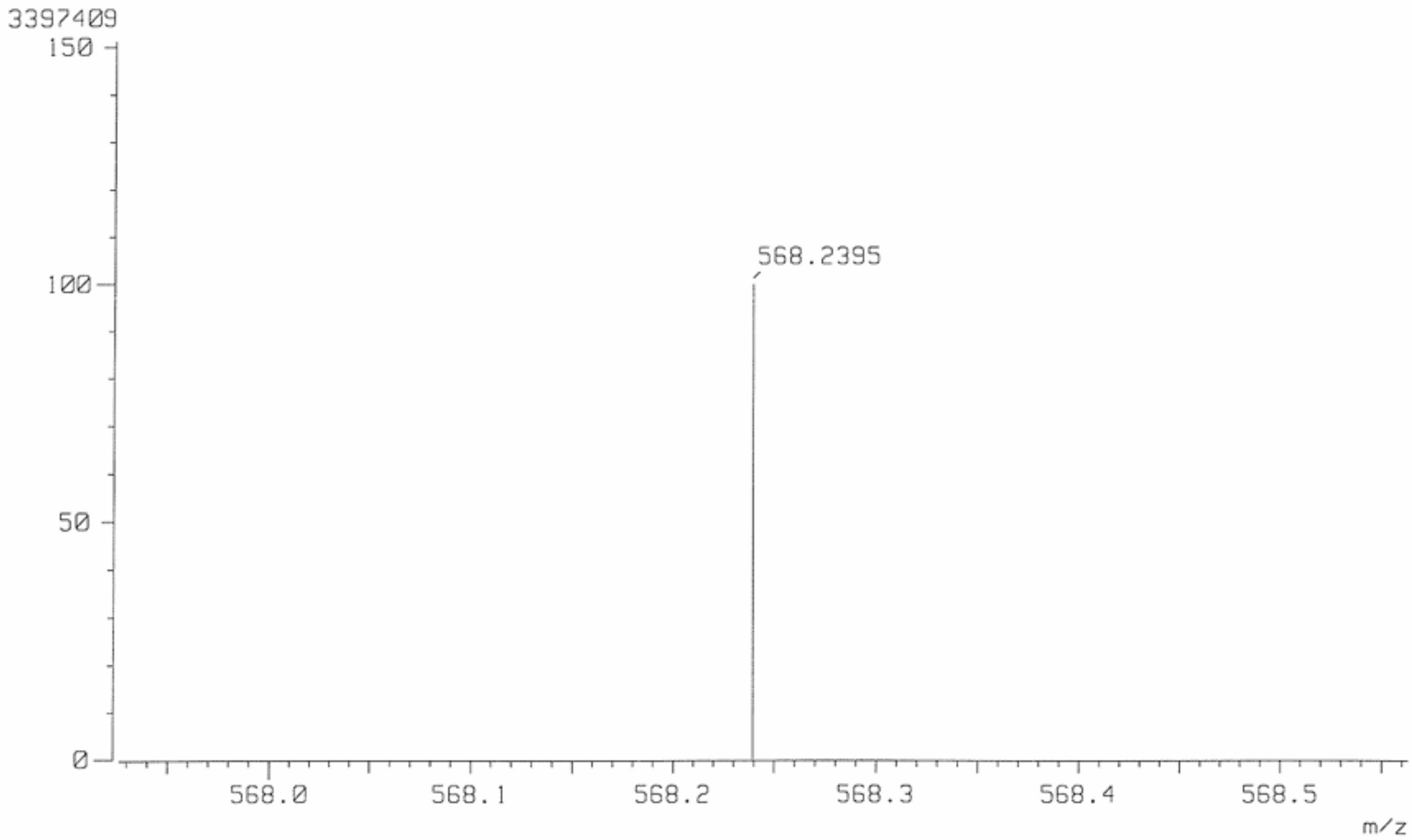

Compound 6f

```
[ Elemental Composition ]                                          Page: 1
Data   : FAB-HS-334-P-007               Date : 16-Sep-2026 16:56
Sample: High resolution GC Mass(JMS-700)
Note : SMlab-Mass Analysis & Research Center
Inlet : Direct                          Ion Mode : FAB+
RT : 1.44 min                           Scan#: (42,46)+14+6
Elements : C 32/0, H 25/0, N 2/0, O 6/0, S 1/0
Mass Tolerance       : 1000ppm, 3mmu if m/z < 3, 5mmu if m/z > 5
Unsaturation (U.S.) : -0.5 - 80.0

Observed m/z Int%   Err[ppm / mmu]    U.S.  Composition
  537.1486    96.8     +0.3 /  +0.1   21.5  C 31 H 25 N 2 O 5 S
```

```
[ Mass Spectrum ]
Data   : FAB-HS-334-P-007              Date : 16-Sep-2026 16:56
Sample: High resolution GC Mass(JMS-700)
Note : SMlab-Mass Analysis & Research Center
Inlet : Direct                         Ion Mode : FAB+
Spectrum Type : Normal Ion [MF-Linear]
RT : 1.44 min           Scan# : (42,46)+14+6
BP : m/z 473.1785       Int.   : 16.07
Output m/z range : 536.9069 to 537.4038      Cut Level : 55.01 %
```

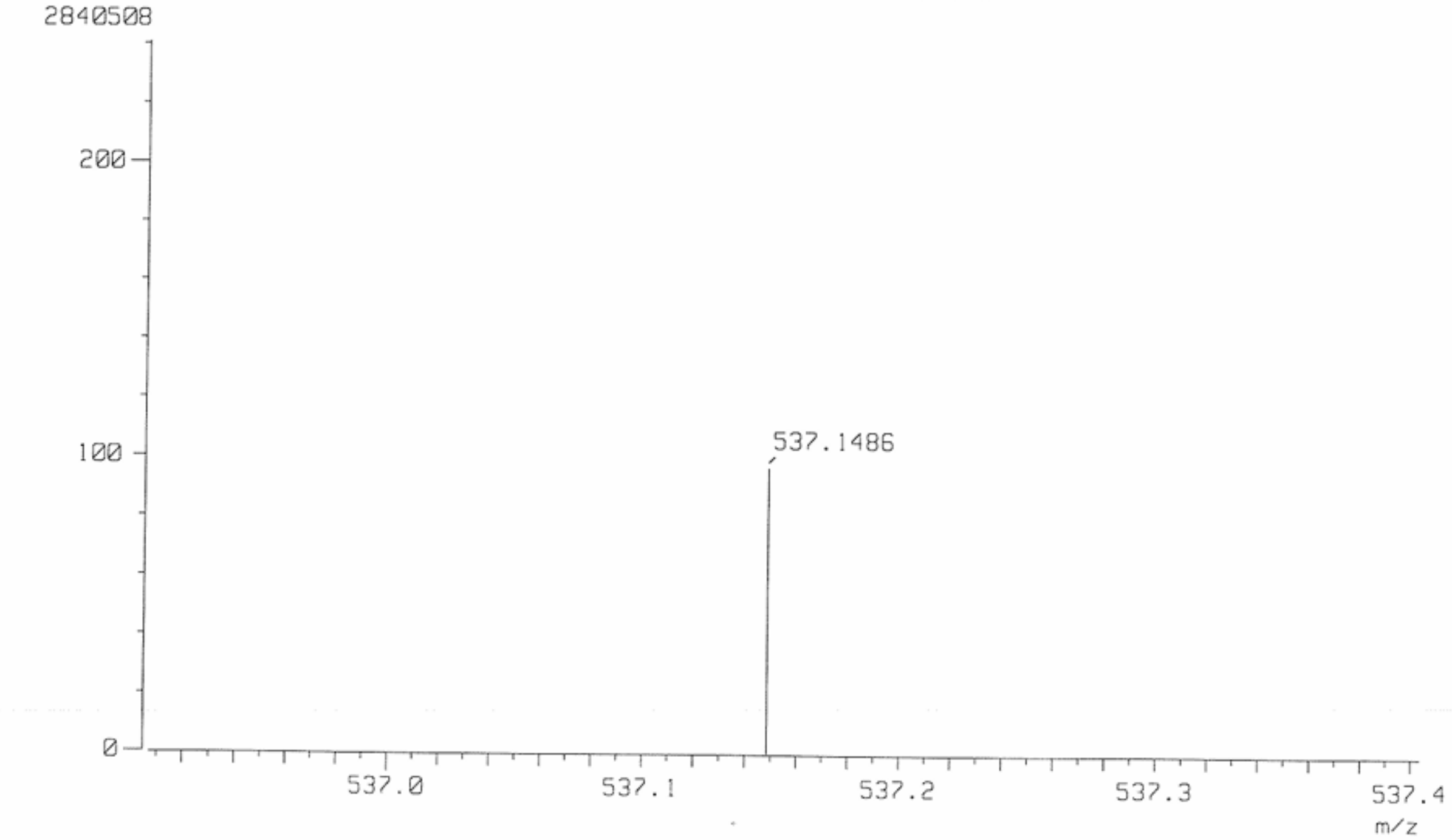

Supplement: Supplementary file 1 [file RA-016-D6RA09632H-s001.pdf]
