# Supplementary material for: Synthesis and diversification of SuFEx-active 2H-indazoles from azobenzenes and ethenesulfonyl fluoride
Source: RSC Adv. 2026 Oct 19. Online ahead of print. doi: 10.1039/d6ra09632h (PMC13678690; doi:10.1039/d6ra09632h)

Supplementary Information (SI) for RSC Advances.
This journal is © The Royal Society of Chemistry

Supporting Information

Synthesis and Diversification of SuFEx-Active 2*H*-Indazoles from Azobenzenes and Ethenesulfonyl Fluoride

Lingayya Rajaka,[a,b†] Hyosik Woo,[a,†] Myunghwa Jung,[a] Chanhee Jang,[c] Seungwan Kim,[a] Jihyo Lee,[a] Saegun Kim,[d] Sangil Han,[c,*] Han Yong Bae,[e,*] and Sang Hoon Han [a,*]

[a] *College of Pharmacy, Korea University, Sejong 30019, Republic of Korea*

[b] *Institute of Pharmaceutical Science and Translational Research, Korea University, Sejong 30019, Republic of Korea*

[c] *Department of Innovative Pharmaceutical Sciences, Kyungpook National University, Daegu 41566, Republic of Korea*

[d] *College of Pharmacy and Research Institute of Pharmaceutical Sciences, Sookmyung Women's University, Seoul 04310, Republic of Korea*

[e] *Department of Chemistry, Sungkyunkwan University, Suwon 16419, Republic of Korea*

* Corresponding authors E-mails: hans3@Korea.ac.kr (S. H. H.), sangil.han@knu.ac.kr (S. H.), hybae@skku.edu (H. Y. B)

[†] L.R. and H.W. equally contributed.

List of the Contents

1. General information and methods -- S3

2. Typical procedure for the synthesis of 2*H*-indazoles -- S4

3. Competition experiments --- S5

4. Deuterium-labelling experiments ---S6

5. Experimental procedure for the gram scale reaction -------------------------------------- S7

6. Experimental procedure for the applications -- S8

7. Characterization data for all products (3a–3p, 4a, 5a–5p, 6a-6f) ----------------------- S11

8. ^{1}H NMR, ^{13}C NMR and ^{19}F NMR spectra of products ----------------------------------- S32

9. HR-MS analytical data of the products -- S108

1. General information and methods

All the experiments were carried out in a well-ventilated fume hood under ambient reaction conditions. Commercially available reagents were used without additional purification, unless otherwise stated. Ethenesulfonyl fluoride (**2a**) was purchased from TCI. Azobenzenes **1a−1p** and **4a−4p**[1, 2] were prepared according to the reported literature. Sealed tubes (13 × 100 mm) were purchased from Fisher Scientific and dried in an oven overnight and cooled at room temperature before use. Thin-layer chromatography was carried out using plates coated with Kieselgel 60 F_{254} (Merck). Nuclear magnetic resonance spectra (^{1}H, ^{13}C and ^{19}F NMR) were recorded on a Jeol 400 and a Bruker 600 MHz NMR instrument in $CDCl_3$ solution and chemical shifts are reported as parts per million (ppm). Resonance patterns are reported with the notations s (singlet), d (doublet), t (triplet), q (quartet), m (multiplet) and br (broad). Coupling constants (*J*) are reported in hertz (Hz). IR spectra were recorded on a JASCO FT/IR4600 spectrophotometer and are reported as cm^{-1}. High resolution mass spectra (HRMS) were recorded on a JEOL JMS-700 spectrometer.

References

[1] Zhang, C.; Jiao, N. *Angew. Chem. Int. Ed.* **2010**, *49*, 6174−6177.
[2] Zhao, R.; Tan, C.; Xie, Y.; Gao, C.; Liu, H.; Jiang, Y. *Tetrahedron Lett.* **2011**, *52*, 3805−3809.

2. Typical procedure for the synthesis of 2*H*-indazoles

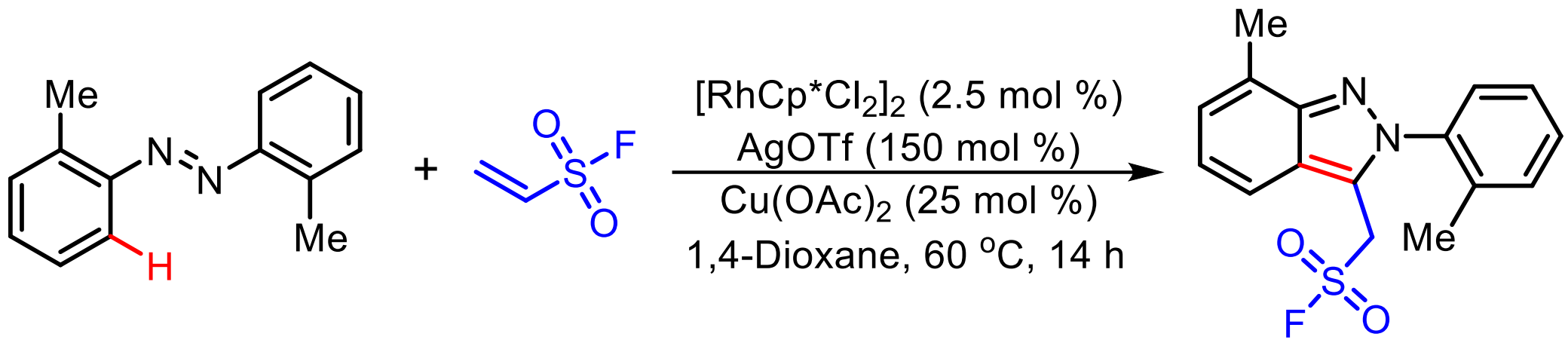

1a **2a** **3a**

To an oven-dried sealed tube charged with 1,2-di-*o*-tolyldiazene (**1a**) (42.0 mg, 0.2 mmol, 100 mol %), $[RhCp^*Cl_2]_2$ (3.1 mg, 0.005 mmol, 2.5 mol %), $Cu(OAc)_2$ (9.0 mg, 0.05 mmol, 25 mol %), AgOTf (77.0 mg, 0.3 mmol, 150 mol %), 1,4-dioxane (1 mL), was added ethenesulfonyl fluoride (**2a**) (66.0 mg, 0.6 mmol, 300 mol %). The reaction mixture was allowed to stir for 14 h at 60 °C. After completion of reaction, cool down reaction mass to room temperature. The reaction mass quenched with H_2O, diluted with EtOAc (10 mL) and did workup. EtOAc layer dried with $MgSO_4$, filtered, concentrated in vacuo. The residue was purified by flash column chromatography (*n*-hexanes/EtOAc = 9:1) to afford **3a** (50.9 mg) in 80% yield.

3. Competition experiments

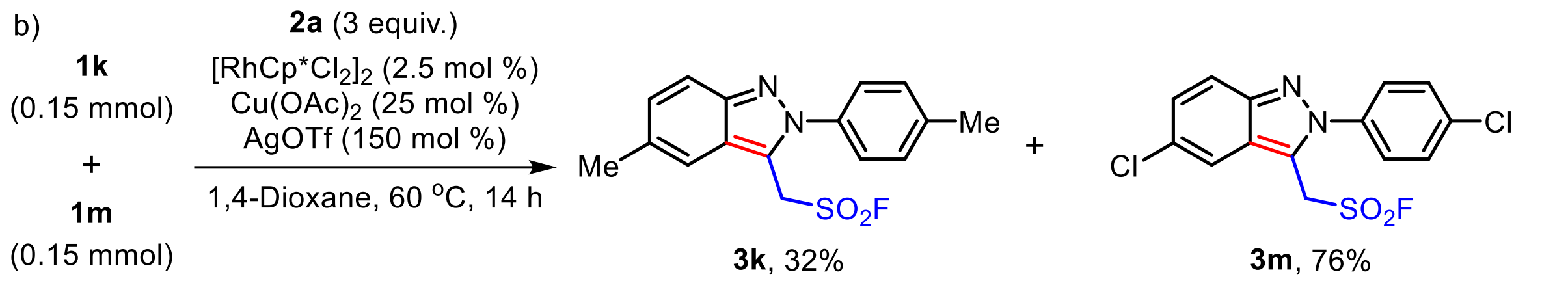

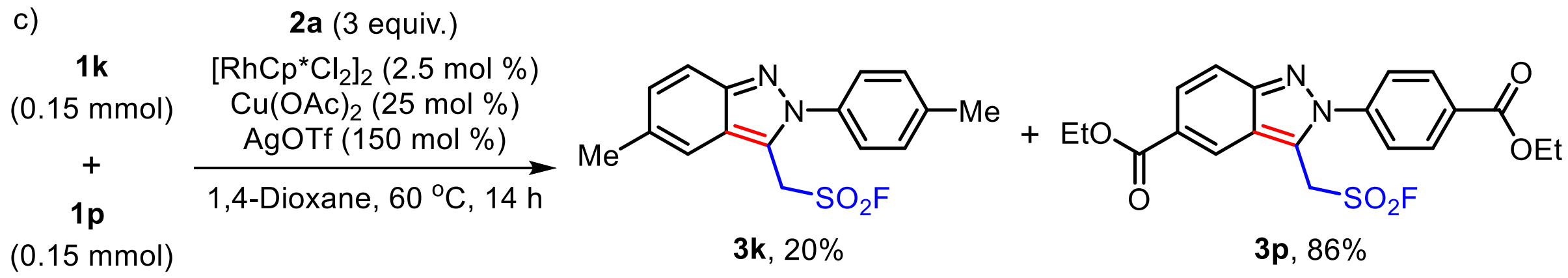

b) To an oven-dried sealed tube charged with 1,2-di-*p*-tolyldiazene (**1k**) (31.5 mg, 0.15 mmol, 50 mol%), 1,2-bis(4-chlorophenyl)diazene (**1m**) (37.6 mg, 0.15 mmol, 50 mol%), $[RhCp^*Cl_2]_2$ (4.6 mg, 0.0075 mmol, 2.5 mol %), $Cu(OAc)_2$ (13.6 mg, 0.075 mmol, 25 mol %), AgOTf (115.6 mg, 0.45 mmol, 150 mol %), 1,4-dioxane (1 mL), was added ethenesulfonyl fluoride (**2a**) (99.1 mg, 0.9 mmol, 300 mol %). The reaction mixture was allowed to stir for 14 h at 60 ºC. After completion of reaction, cool down reaction mass to room temperature. The reaction mass quenched with H_2O, diluted with EtOAc (10 mL) and did workup. EtOAc layer dried with $MgSO_4$, filtered, concentrated in vacuo. The residue was purified by flash column chromatography (*n*-hexanes/EtOAc = 9:1) to afford **3k** (15.2 mg) in 32% yield and **3m** (41.2 mg) in 76% yield.

c) To an oven-dried sealed tube charged with 1,2-di-*p*-tolyldiazene (**1k**) (31.5 mg, 0.15 mmol, 50 mol%), diethyl 4,4'-(diazene-1,2-diyl)dibenzoate (**1p**) (48.9 mg, 0.15 mmol, 50 mol%), $[RhCp^*Cl_2]_2$ (4.6 mg, 0.0075 mmol, 2.5 mol %), $Cu(OAc)_2$ (13.6 mg, 0.075 mmol, 25 mol %), AgOTf (115.6 mg, 0.45 mmol, 150 mol %), 1,4-dioxane (1 mL), was added ethenesulfonyl fluoride (**2a**) (99.1 mg, 0.9 mmol, 300 mol %). The reaction mixture was allowed to stir for 14 h at 60 ºC. After completion of reaction, cool down reaction mass to room temperature. The reaction mass quenched with H_2O, diluted with EtOAc (10 mL) and did workup. EtOAc layer dried with $MgSO_4$, filtered, concentrated in vacuo. The residue was purified by flash column chromatography (*n*-hexanes/EtOAc = 9:1) to afford **3k** (9.6 mg) in 20% yield and **3p** (56.0 mg) in 86% yield.

4. Deuterium-labelling experiments

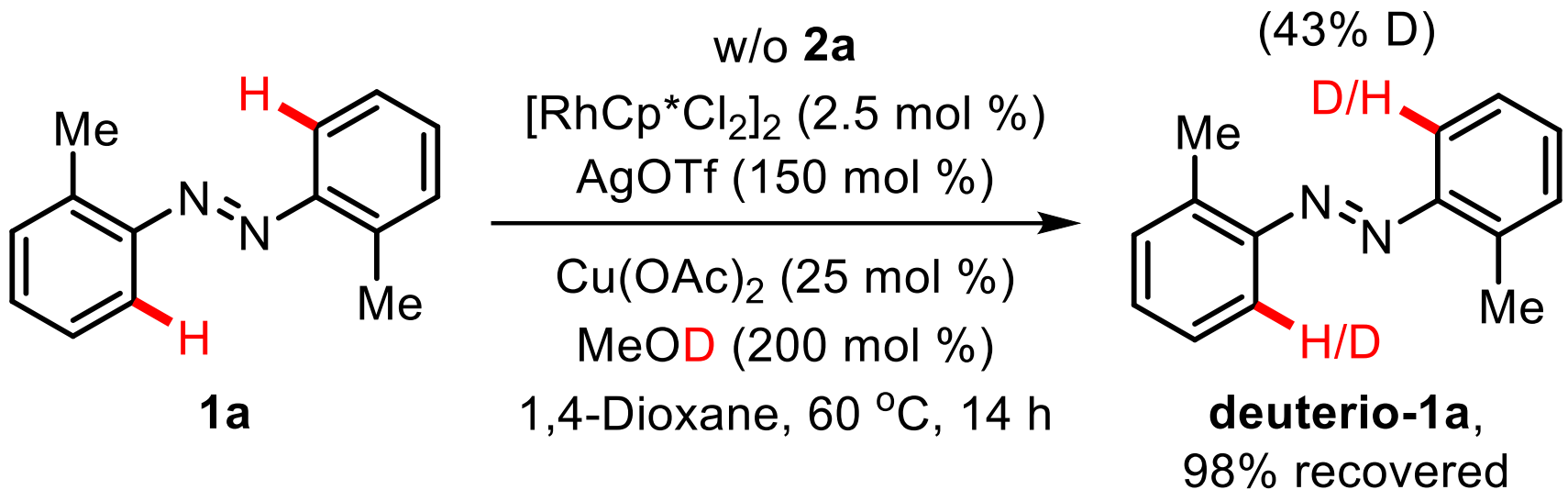

To an oven-dried sealed tube charged with 1,2-di-*o*-tolyldiazene (**1a**) (42.0 mg, 0.2 mmol, 100 mol %), $[RhCp^*Cl_2]_2$ (3.1 mg, 0.005 mmol, 2.5 mol %), $Cu(OAc)_2$ (9.0 mg, 0.05 mmol, 25 mol %), AgOTf (77.0 mg, 0.3 mmol, 150 mol %), 1,4-dioxane (1 mL), was added MeOD (14.4 mg, 0.4 mmol, 200 mol %). The reaction mixture was allowed to stir for 14 h at 60 °C. After completion of reaction, cool down reaction mass to room temperature. The reaction mass quenched with H_2O, diluted with EtOAc (10 mL) and did workup. EtOAc layer dried with $MgSO_4$, filtered, concentrated in vacuo. The residue was purified by flash column chromatography (*n*-hexanes/EtOAc = 9:1) to afford **deuterio-1a** (41.3 mg) in 98% yield.

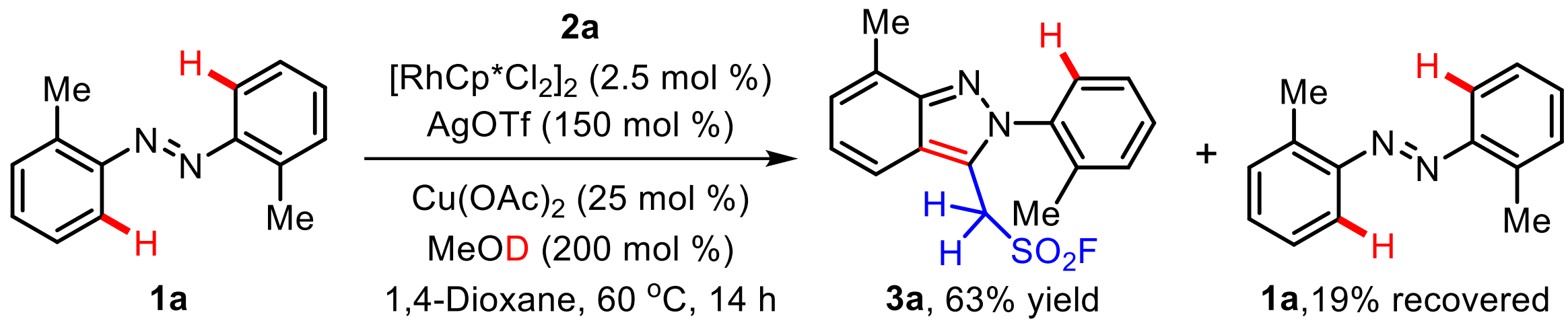

To an oven-dried sealed tube charged with 1,2-di-*o*-tolyldiazene (**1a**) (42.0 mg, 0.2 mmol, 100 mol %), $[RhCp^*Cl_2]_2$ (3.1 mg, 0.005 mmol, 2.5 mol %), $Cu(OAc)_2$ (9.0 mg, 0.05 mmol, 25 mol %), AgOTf (77.0 mg, 0.3 mmol, 150 mol %), 1,4-dioxane (1 mL), MeOD (14.4 mg, 0.4 mmol, 200 mol %), was added ethenesulfonyl fluoride (**2a**) (66.0 mg, 0.6 mmol, 300 mol %). The reaction mixture was allowed to stir for 14 h at 60 °C. After completion of reaction, cool down reaction mass to room temperature. The reaction mass quenched with H_2O, diluted with EtOAc (10 mL) and did workup. EtOAc layer dried with $MgSO_4$, filtered, concentrated in vacuo. The residue was purified by flash column chromatography (*n*-hexanes/EtOAc = 9:1) to afford **3a** (39.9 mg) in 63% yield and recovered **1a** (8.1 mg) in 19% yield.

5. Experimental procedure for the gram scale reaction

To an oven-dried sealed tube charged with 1,2-di-*o*-tolyldiazene (**1a**) (1051.4 mg, 5.0 mmol, 100 mol %), [RhCp*Cl_2]$_2$ (77.2 mg, 0.125 mmol, 2.5 mol %), Cu(OAc)$_2$ (227.0 mg, 1.25 mmol, 25 mol %), AgOTf (1927.0 mg, 7.5 mmol, 150 mol %), 1,4-dioxane (10 mL), was added ethenesulfonyl fluoride (**2a**) (1651.5 mg, 15.0 mmol, 300 mol %). The reaction mixture was allowed to stir for 14 h at 60 ºC. After completion of reaction, cool down reaction mass to room temperature. The reaction mass quenched with H_2O, diluted with EtOAc (25 mL) and did workup. EtOAc layer dried with $MgSO_4$, filtered, concentrated in vacuo. The residue was purified by flash column chromatography (*n*-hexanes/EtOAc = 9:1) to afford **3a** (1110 mg) in 70% yield.

6. Experimental procedure for the applications

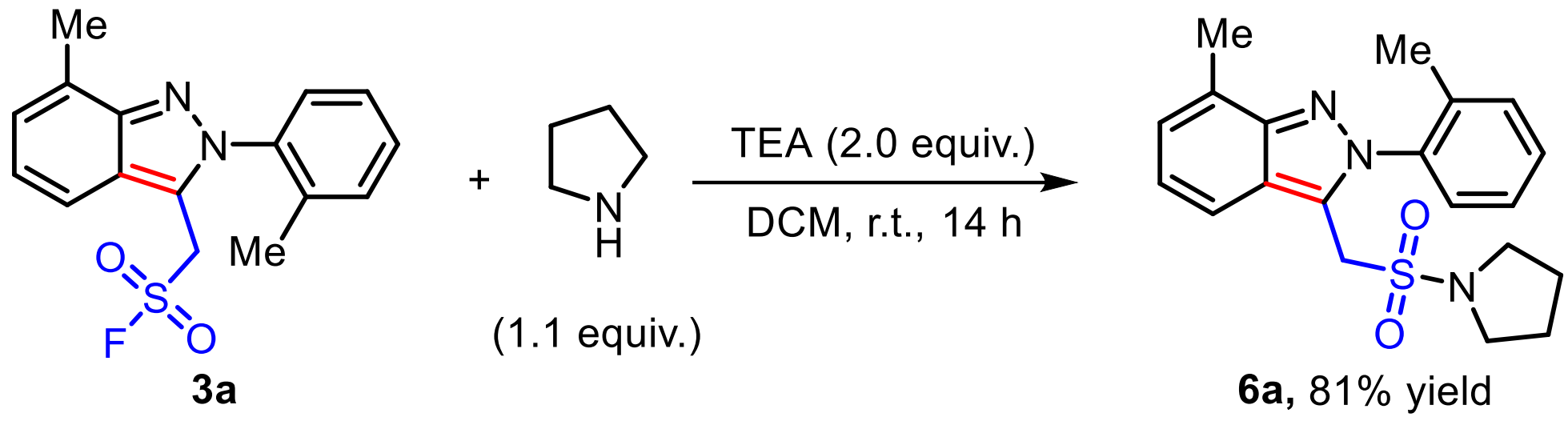

To an oven-dried sealed tube charged with (7-methyl-2-(*o*-tolyl)-2*H*-indazol-3-yl)methanesulfonyl fluoride (**3a**) (31.8 mg, 0.1 mmol, 100 mol %), pyrrolidine (7.8 mg, 0.11 mmol, 110 mol %), TEA (20.2 mg, 0.2 mmol, 200 mol %), DCM (1 mL). The reaction mixture was allowed to stir for 14 h at 25 °C. After completion of reaction as indicated by TLC analysis, reaction mass quenched with H_2O, diluted with EtOAc (10 mL) and did workup. EtOAc layer dried with $MgSO_4$, filtered, concentrated in vacuo. The residue was purified by flash column chromatography (*n*-hexanes/EtOAc = 2:1) to afford **6a** (30.0 mg) in 81% yield.

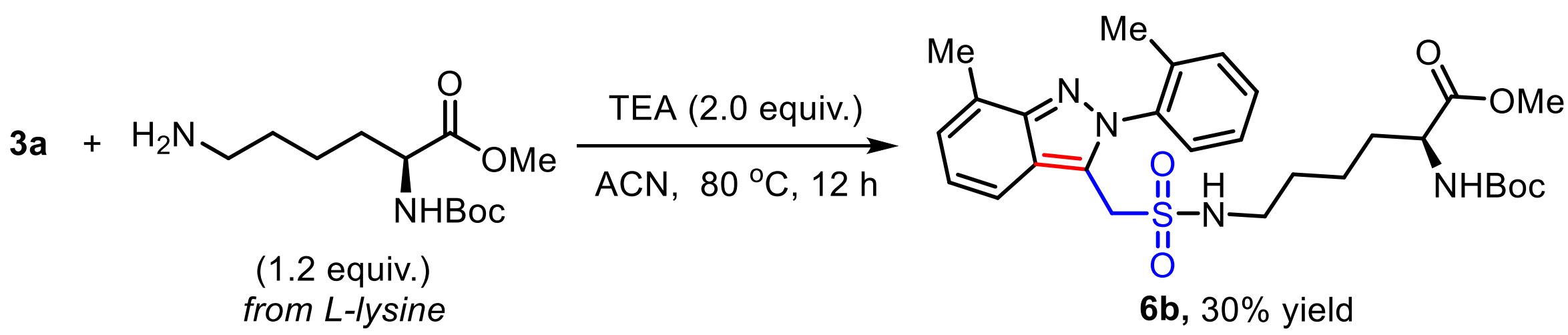

To an oven-dried sealed tube charged with (7-methyl-2-(*o*-tolyl)-2*H*-indazol-3-yl)methanesulfonyl fluoride (**3a**) (31.8 mg, 0.1 mmol, 100 mol %), protected lysine amino acid derivative (31.2 mg, 0.12 mmol, 120 mol %), TEA (20.2 mg, 0.2 mmol, 200 mol %), acetonitrile (1 mL). The reaction mixture was allowed to stir for 12 h at 80 °C. After completion of reaction as indicated by TLC analysis, reaction mass quenched with H_2O, diluted with EtOAc (10 mL) and did workup. EtOAc layer dried with $MgSO_4$, filtered, concentrated in vacuo. The residue was purified by flash column chromatography (*n*-hexanes/EtOAc = 1:1) to afford **6b** (16.8 mg) in 30% yield.

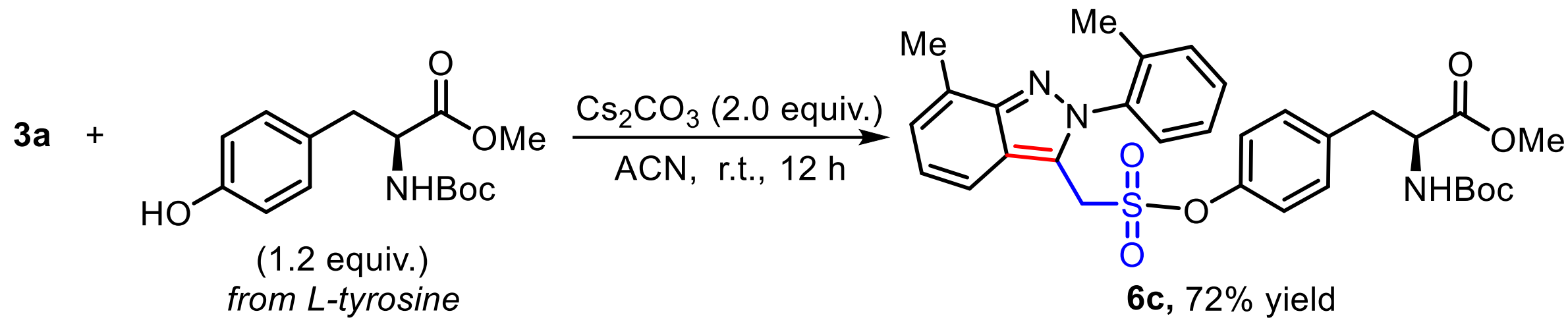

To an oven-dried sealed tube charged with (7-methyl-2-(*o*-tolyl)-2*H*-indazol-3-yl)methanesulfonyl fluoride (**3a**) (31.8 mg, 0.1 mmol, 100 mol %), protected tyrosine amino acid derivative (35.4 mg, 0.12 mmol, 120 mol %), Cs_2CO_3 (65.1 mg, 0.2 mmol, 200 mol %), acetonitrile (1 mL). The reaction mixture was allowed to stir for 12 h at 25 °C. After completion of reaction as indicated by TLC analysis, reaction mass quenched with H_2O, diluted with EtOAc (10 mL) and did workup. EtOAc layer dried with $MgSO_4$, filtered, concentrated in vacuo. The residue was purified by flash column chromatography (*n*-hexanes/EtOAc = 3:1) to afford **6c** (42.8 mg) in 72% yield.

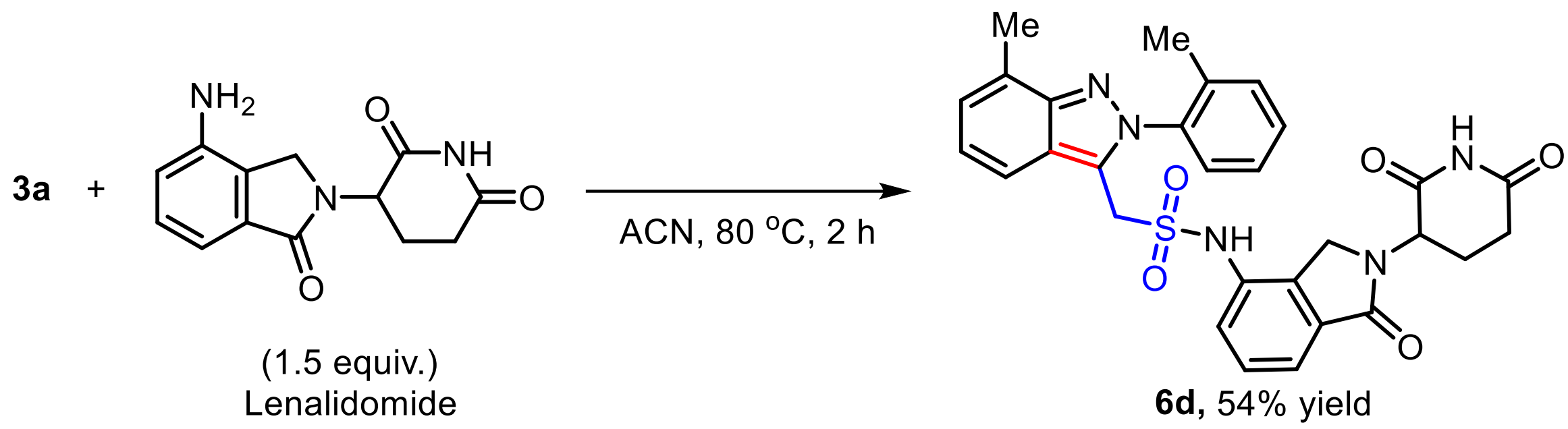

To an oven-dried sealed tube charged with (7-methyl-2-(*o*-tolyl)-2*H*-indazol-3-yl)methanesulfonyl fluoride (**3a**) (31.8 mg, 0.1 mmol, 100 mol %), lenalidomide (38.9 mg, 0.15 mmol, 150 mol %), acetonitrile (1 mL). The reaction mixture was allowed to stir for 2 h at 80 °C. After completion of reaction as indicated by TLC analysis, reaction mass quenched with H_2O, diluted with EtOAc (10 mL) and did workup. EtOAc layer dried with $MgSO_4$, filtered, concentrated in vacuo. The residue was purified by flash column chromatography (*n*-hexanes/EtOAc = 1:4) to afford **6d** (30.2 mg) in 54% yield.

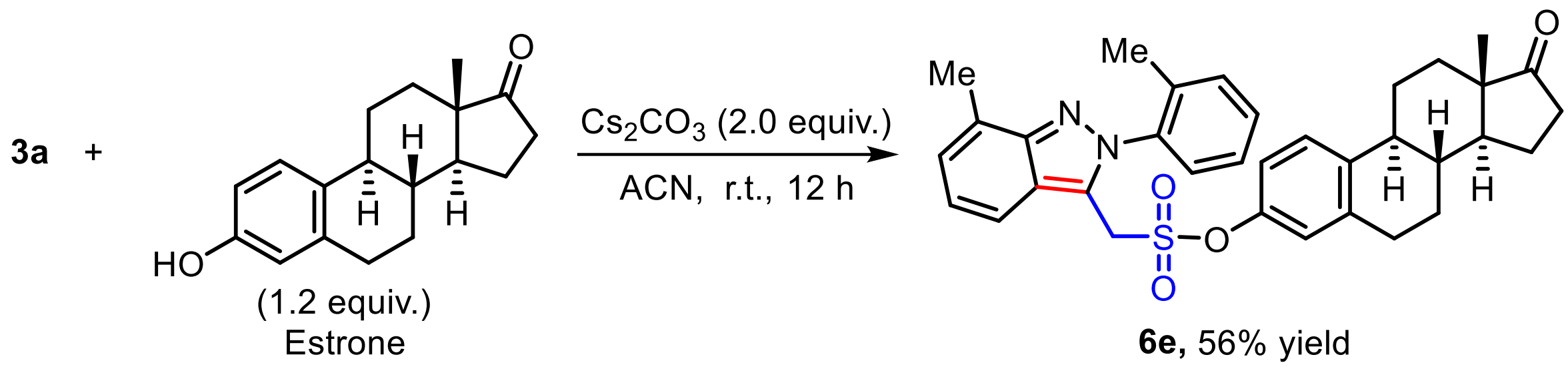

To an oven-dried sealed tube charged with (7-methyl-2-(*o*-tolyl)-2*H*-indazol-3-yl)methanesulfonyl fluoride (**3a**) (31.8 mg, 0.1 mmol, 100 mol %), estrone (32.4 mg, 0.12 mmol, 120 mol %), Cs_2CO_3 (65.1 mg, 0.2 mmol, 200 mol %), acetonitrile (1 mL). The reaction mixture was allowed to stir for 12 h at 25 °C. After completion of reaction as indicated by TLC analysis, reaction mass quenched with H_2O, diluted with EtOAc (10 mL) and did workup. EtOAc layer dried with $MgSO_4$, filtered, concentrated in vacuo. The residue was purified by flash column chromatography (*n*-hexanes/EtOAc = 2:1) to afford **6e** (31.9 mg) in 56% yield.

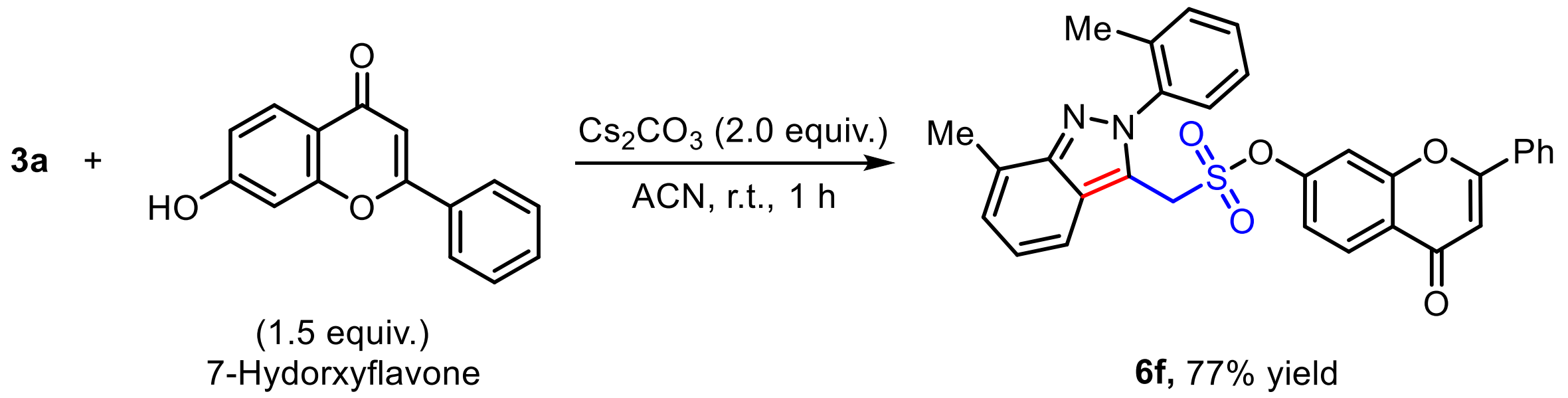

To an oven-dried sealed tube charged with (7-methyl-2-(*o*-tolyl)-2*H*-indazol-3-yl)methanesulfonyl fluoride (**3a**) (31.8 mg, 0.1 mmol, 100 mol %), 7-hydroxyflavone (35.7 mg, 0.15 mmol, 150 mol %), Cs_2CO_3 (65.1 mg, 0.2 mmol, 200 mol %), acetonitrile (1 mL). The reaction mixture was allowed to stir for 1 h at 25 °C. After completion of reaction as indicated by TLC analysis, reaction mass quenched with H_2O, diluted with EtOAc (10 mL) and did workup. EtOAc layer dried with $MgSO_4$, filtered, concentrated in vacuo. The residue was purified by flash column chromatography (*n*-hexanes/EtOAc = 3:1) to afford **6f** (41.5 mg) in 77% yield.

7. Characterization data for all products (3a–3p, 3aa, 5a–5q, 5q', 6a-6f)

(7-Methyl-2-(*o*-tolyl)-2*H*-indazol-3-yl)methanesulfonyl fluoride (3a)

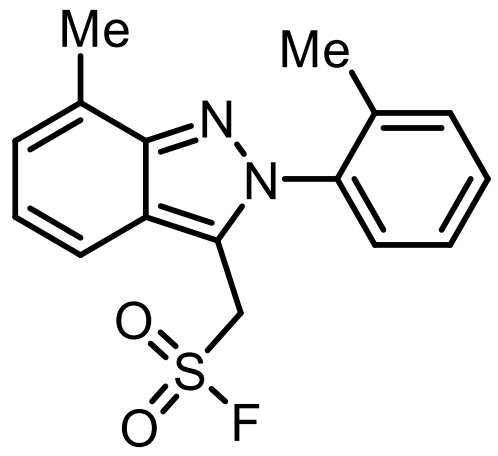

3a

50.9 mg (80%); Pale violet solid; ^{1}H NMR (600 MHz, $CDCl_3$) δ 7.58 (d, J = 8.2 Hz, 1H), 7.51 – 7.48 (m, 1H), 7.43 – 7.41 (m, 1H), 7.40 – 7.38 (m, 2H), 7.21 (d, J = 6.7 Hz, 1H), 7.19 – 7.17 (m, 1H), 4.91 (d, J = 89.5 Hz, 2H), 2.67 (s, 3H), 2.02 (s, 3H) ppm; ^{13}C NMR (150 MHz, $CDCl_3$) δ 149.23, 137.24, 136.50, 131.74, 130.78, 128.93, 127.89, 127.09, 126.34, 124.51, 122.00, 120.19, 116.57, 47.61 (d, J = 20.8 Hz), 17.20, 17.12 ppm; ^{19}F NMR (376 MHz, $CDCl_3$) δ 55.66 ppm; FT-IR ($CHCl_3$, film) υ 3028, 2919, 1503, 1409, 1204, 869 cm^{-1}; HRMS (EI+) calcd for $C_{16}H_{15}FN_2O_2S$ $[M]^+$ 318.0838, found 318.0834.

(7-Ethyl-2-(2-ethylphenyl)-2*H*-indazol-3-yl)methanesulfonyl fluoride (3b)

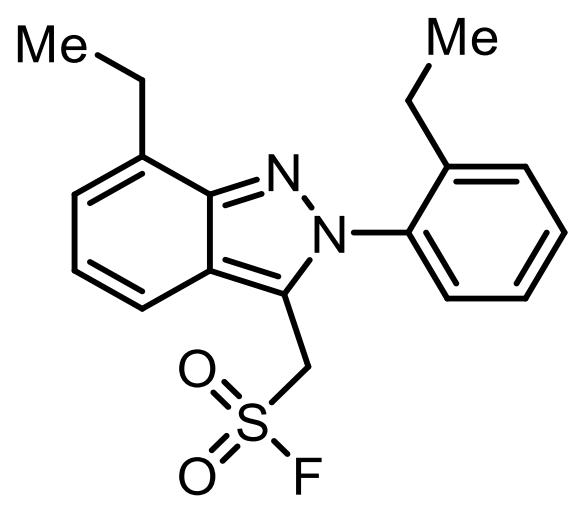

3b

50.3 mg (73%); Yellow solid; ^{1}H NMR (600 MHz, $CDCl_3$) δ 7.51 (d, J = 8.3 Hz, 1H), 7.48 – 7.45 (m, 1H), 7.40 (d, J = 7.4 Hz, 1H), 7.33 – 7.31 (m, 2H), 7.18 – 7.11 (m, 2H), 4.82 (d, J = 109.5 Hz, 2H), 3.02 (d, J = 20.6 Hz, 2H), 2.15 (d, J = 7.2 Hz, 2H), 1.33 (t, J = 7.6 Hz, 3H), 1.04 (t, J = 7.6 Hz, 3H) ppm; ^{13}C NMR (150 MHz, $CDCl_3$) δ 148.40, 142.26, 136.73, 135.01, 130.99, 129.93, 127.99, 126.97, 124.58, 124.33, 122.15, 120.31, 116.58 (d, J = 1.1 Hz), 47.72 (d, J = 21.4 Hz), 24.47, 23.82, 14.74, 14.20 ppm; ^{19}F NMR (376 MHz, $CDCl_3$) δ 55.80 ppm; FT-IR ($CHCl_3$, film) υ 2968, 2930, 1534, 1498, 1460, 1411, 1236, 1206, 1089, 889 cm^{-1}; HRMS

(EI+) calcd for $C_{18}H_{19}FN_2O_2S$ $[M]^+$ 346.1151, found 346.1146.

(7-Fluoro-2-(2-fluorophenyl)-2*H*-indazol-3-yl)methanesulfonyl fluoride (3c)

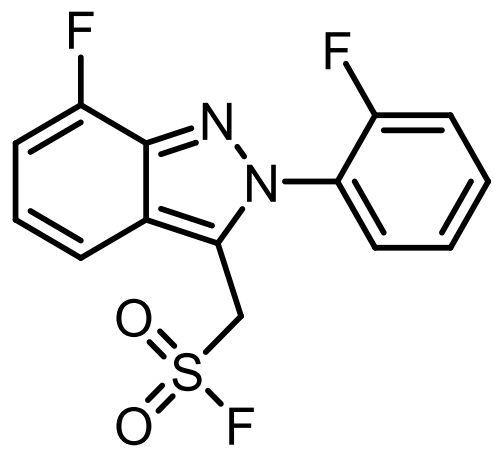

3c

51.0 mg (78%); Brown solid; ^{1}H NMR (600 MHz, $CDCl_3$) δ 7.67 (td, *J* = 7.6, 1.7 Hz, 1H), 7.64 – 7.60 (m, 1H), 7.51 (d, *J* = 8.5 Hz, 1H), 7.41 (t, *J* = 8.0 Hz, 1H), 7.35 (t, *J* = 8.5 Hz, 1H), 7.20 (td, *J* = 8.0, 4.3 Hz, 1H), 7.06 (dd, *J* = 11.0, 7.5 Hz, 1H), 5.01 (d, *J* = 3.5 Hz, 2H) ppm; ^{13}C NMR (150 MHz, $CDCl_3$) δ 157.25, 155.58, 154.13, 152.42, 140.96 (d, *J* = 17.0 Hz), 132.80 (d, *J* = 7.7 Hz), 130.06, 126.10 (d, *J* = 11.5 Hz), 125.66 (d, *J* = 3.9 Hz), 125.44 (d, *J* = 5.5 Hz), 124.73 (d, *J* = 6.0 Hz), 122.20 (d, *J* = 3.3 Hz), 117.08 (d, *J* = 18.7 Hz), 115.09 (d, *J* = 4.9 Hz), 110.57 (d, *J* = 15.9 Hz), 47.44 (dd, *J* = 21.7, 3.0 Hz) ppm; ^{19}F NMR (376 MHz, $CDCl_3$) δ 55.66, -122.37, -127.36 ppm; FT-IR ($CHCl_3$, film) υ 2947, 2921, 1507, 1405, 1267, 1240, 1206, 1090, 875 cm^{-1}; HRMS (EI+) calcd for $C_{14}H_9F_3N_2O_2S$ $[M]^+$ 326.0337, found 326.0336.

(7-Chloro-2-(2-chlorophenyl)-2*H*-indazol-3-yl)methanesulfonyl fluoride (3d)

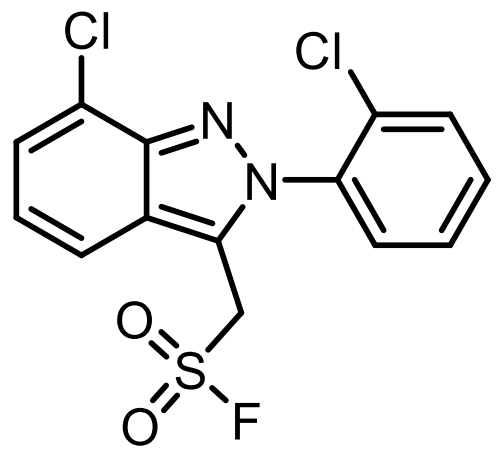

3d

51.8 mg (72%); Pale green solid; ^{1}H NMR (600 MHz, $CDCl_3$) δ 7.70 – 7.65 (m, 2H), 7.63 – 7.57 (m, 2H), 7.54 (td, *J* = 7.6, 1.7 Hz, 1H), 7.47 (d, *J* = 7.2 Hz, 1H), 7.23 (dd, *J* = 8.5, 7.2 Hz, 1H), 5.14 (d, *J* = 15.6 Hz, 1H), 4.73 (dd, *J* = 15.6, 5.4 Hz, 1H) ppm; ^{13}C NMR (150 MHz, $CDCl_3$) δ 146.98, 135.65, 132.38, 131.42, 130.80, 130.50, 128.42, 127.07, 124.79, 124.14, 123.41, 122.50, 118.09, 47.57 (d, *J* = 22.0 Hz) ppm; ^{19}F NMR (376 MHz, $CDCl_3$) δ 55.75 ppm; FT-IR ($CHCl_3$, film) υ 2923, 2851, 1490, 1407, 1264, 1200, 1052, 869 cm^{-1}; HRMS (EI+) calcd

for $C_{14}H_9Cl_2FN_2O_2S$ $[M]^+$ 357.9746, found 357.9742.

(6-Ethyl-2-(3-ethylphenyl)-2H-indazol-3-yl)methanesulfonyl fluoride (3e)

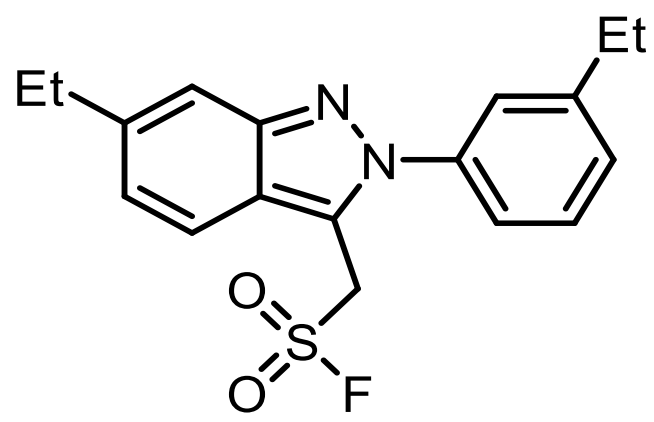

3e

32.8 mg (47%); Yellow solid; ^{1}H NMR (600 MHz, $CDCl_3$) δ 7.66 (d, *J* = 8.6 Hz, 1H), 7.58 (s, 1H), 7.49 (t, *J* = 7.7 Hz, 1H), 7.42 (d, *J* = 6.6 Hz, 2H), 7.39 (t, *J* = 8.1 Hz, 1H), 7.16 (dd, *J* = 8.7, 1.5 Hz, 1H), 5.04 (d, *J* = 2.9 Hz, 2H), 2.81 – 2.78 (m, 2H), 2.78 – 2.74 (m, 2H), 1.32 (t, *J* = 7.5 Hz, 3H), 1.29 (t, *J* = 7.6 Hz, 3H) ppm; ^{13}C NMR (150 MHz, $CDCl_3$) δ 149.51, 146.52, 143.89, 138.58, 129.83, 129.64, 126.33, 126.25, 123.79, 121.43, 119.27, 118.81, 115.49, 48.09 (d, *J* = 21.4 Hz), 29.55, 28.77, 15.40, 15.32 ppm; ^{19}F NMR (376 MHz, $CDCl_3$) δ 55.82 ppm; FT-IR ($CHCl_3$, film) υ 2962, 2929, 2873, 1495, 1408, 1206, 1054, 862 cm^{-1}; HRMS (EI+) calcd for $C_{18}H_{19}FN_2O_2S$ $[M]^+$ 346.1151, found 346.1152.

(6-Isopropyl-2-(3-isopropylphenyl)-2H-indazol-3-yl)methanesulfonyl fluoride (3f)

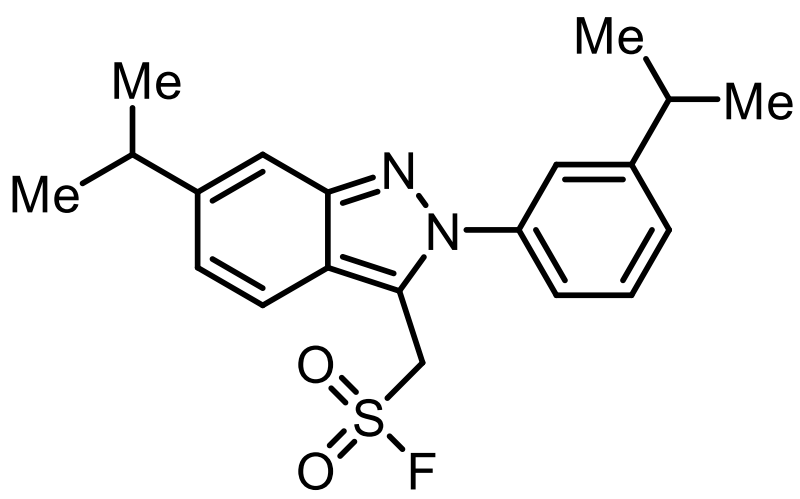

3f

50.2 mg (67%); Yellow solid; ^{1}H NMR (600 MHz, $CDCl_3$) δ 7.67 (d, *J* = 8.8 Hz, 1H), 7.62 (s, 1H), 7.49 (t, *J* = 7.7 Hz, 1H), 7.46 – 7.43 (m, 2H), 7.38 – 7.36 (m, 1H), 7.21 (dd, *J* = 8.8, 1.4 Hz, 1H), 5.02 (d, *J* = 2.9 Hz, 2H), 3.08 – 3.04 (m, 1H), 3.03 – 2.98 (m, 1H), 1.34 (d, *J* = 6.9 Hz, 6H), 1.30 (d, *J* = 6.9 Hz, 6H) ppm; ^{13}C NMR (150 MHz, $CDCl_3$) δ 151.19, 149.45, 148.47, 138.56, 129.63, 128.50, 125.10, 124.90, 123.89, 121.51, 119.25, 118.87, 114.01, 48.09 (d, *J* = 20.8 Hz), 34.65, 34.16, 23.90, 23.79 ppm; ^{19}F NMR (376 MHz, $CDCl_3$) δ 55.86 ppm; FT-IR ($CHCl_3$, film) υ 2964, 2874, 1497, 1375, 1040, 866 cm^{-1}; HRMS (EI+) calcd for $C_{20}H_{23}FN_2O_2S$

$[M]^+$ 374.1464, found 374.1465.

(6-Fluoro-2-(3-fluorophenyl)-2*H*-indazol-3-yl)methanesulfonyl fluoride (3g)

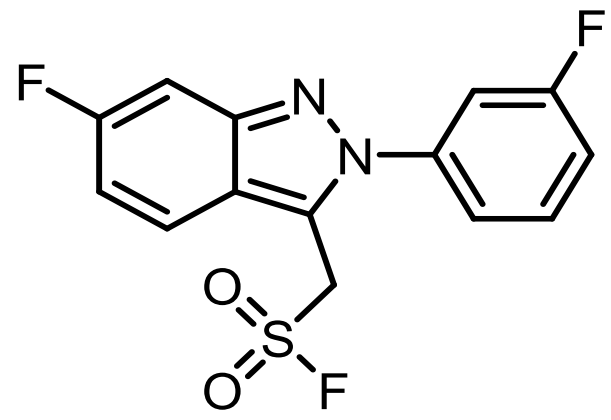

3g

37.2 mg (57%); Pale yellow solid; ^{1}H NMR (600 MHz, $CDCl_3$) δ 7.60 (d, *J* = 5.1 Hz, 1H), 7.58 (d, *J* = 8.2 Hz, 1H), 7.40 (d, *J* = 7.9 Hz, 1H), 7.35 – 7.33 (m, 2H), 7.32 (s, 1H), 6.89 (dd, *J* = 10.7, 7.5 Hz, 1H), 5.18 (d, *J* = 4.0 Hz, 2H) ppm; ^{13}C NMR (150 MHz, $CDCl_3$) δ 162.87 (d, *J* = 249.5 Hz), 155.18 (d, *J* = 252.2 Hz), 150.89 (d, *J* = 5.0 Hz), 139.29 (d, *J* = 9.8 Hz), 131.33 (d, *J* = 8.8 Hz), 127.92 (d, *J* = 6.6 Hz), 122.42 (d, *J* = 3.3 Hz), 118.98 (d, *J* = 5.5 Hz), 117.88 (d, *J* = 20.8 Hz), 114.84 (d, *J* = 2.7 Hz), 114.81, 114.67, 107.35 (d, *J* = 17.6 Hz), 47.91 (dd, *J* = 20.8, 2.2 Hz) ppm; ^{19}F NMR (376 MHz, $CDCl_3$) δ 55.23 (d, *J* = 22.6 Hz), -108.89, -121.54 ppm; FT-IR ($CHCl_3$, film) υ 2920, 2847, 1602, 1567, 1408, 1236, 1201, 1088, 871, 849, 811 cm^{-1}; HRMS (EI+) calcd for $C_{14}H_9F_3N_2O_2S$ $[M]^+$ 326.0337, found 326.0333.

(6-Bromo-2-(3-bromophenyl)-2*H*-indazol-3-yl)methanesulfonyl fluoride (3h)

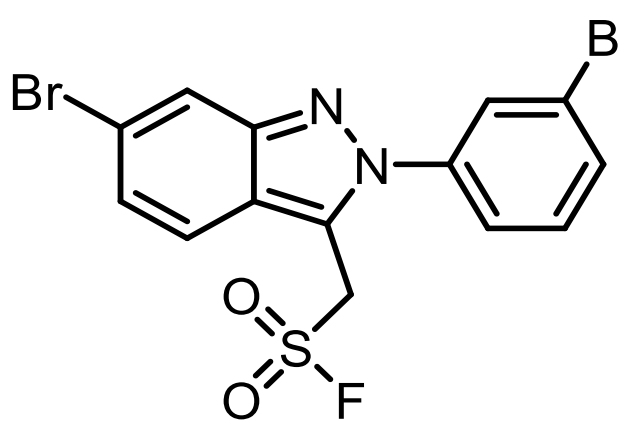

3h

54.7 mg (61%); Brown solid; ^{1}H NMR (600 MHz, $CDCl_3$) δ 7.92 (d, *J* = 1.7 Hz, 1H), 7.69 – 7.67 (m, 1H), 7.55 (d, *J* = 9.0 Hz, 1H), 7.50 – 7.49 (m, 1H), 7.41 (t, *J* = 8.0 Hz, 1H), 7.29 (dd, *J* = 9.0, 1.6 Hz, 1H), 7.19 (s, 1H), 4.97 (d, *J* = 2.3 Hz, 2H) ppm; ^{13}C NMR (150 MHz, $CDCl_3$) δ 149.67, 139.27, 133.81, 131.19, 129.96, 128.55, 125.17, 123.53, 121.97, 121.52, 121.03, 120.62, 120.50, 47.68 (d, *J* = 22.0 Hz) ppm; ^{19}F NMR (376 MHz, $CDCl_3$) δ 56.12 ppm; FT-IR ($CHCl_3$, film) υ 2924, 2853, 1581, 1476, 1259, 1196, 1035, 862 cm^{-1}; HRMS (EI+) calcd for

$C_{14}H_9Br_2FN_2O_2S$ $[M]^+$ 445.8736, found 445.8737.

Ethyl 2-(3-(ethoxycarbonyl)phenyl)-3-((fluorosulfonyl)methyl)-2*H*-indazole-6-carboxylate (3i)

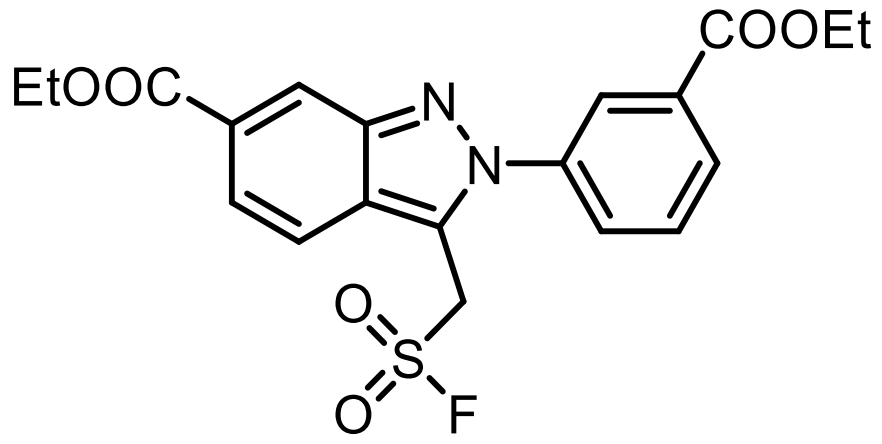

3i

63.9 mg (74%); Yellow solid; ^{1}H NMR (600 MHz, $CDCl_3$) δ 8.61 (t, *J* = 1.2 Hz, 1H), 8.29 (dt, *J* = 7.9, 1.3 Hz, 1H), 8.27 (t, *J* = 1.8 Hz, 1H), 7.92 (dd, *J* = 8.9, 1.3 Hz, 1H), 7.85 – 7.83 (m, 1H), 7.79 (d, *J* = 9.0 Hz, 1H), 7.72 (t, *J* = 7.9 Hz, 1H), 5.09 (d, *J* = 2.8 Hz, 2H), 4.46 – 4.43 (m, 2H), 4.43 – 4.40 (m, 2H), 1.44 (t, *J* = 7.1 Hz, 3H), 1.40 (t, *J* = 7.1 Hz, 3H) ppm; ^{13}C NMR (150 MHz, $CDCl_3$) δ 166.49, 165.06, 148.42, 138.54, 132.59, 131.55, 130.76, 130.26, 130.14, 127.42, 124.81, 124.18, 122.28, 120.32, 119.28, 61.91, 61.49, 47.74 (d, *J* = 21.4 Hz), 14.46, 14.36 ppm; ^{19}F NMR (376 MHz, $CDCl_3$) δ 56.04 ppm; FT-IR ($CHCl_3$, film) υ 2960, 2923, 2855, 1701, 1408, 1368, 1322, 1206, 1080, 865 cm^{-1}; HRMS (EI+) calcd for $C_{20}H_{19}FN_2O_6S$ $[M]^+$ 434.0948, found 434.0950.

(2-Phenyl-2*H*-indazol-3-yl)methanesulfonyl fluoride (3j)

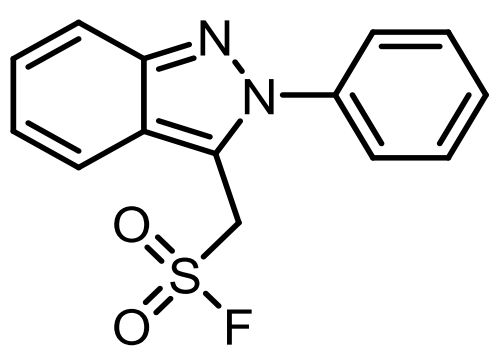

3j

32.0 mg (55%); Yellow solid; ^{1}H NMR (600 MHz, $CDCl_3$) δ 7.83 (dt, *J* = 8.8, 1.0 Hz, 1H), 7.74 (dd, *J* = 8.6, 1.1 Hz, 1H), 7.60 – 7.59 (m, 5H), 7.42 (ddd, *J* = 8.8, 6.7, 1.1 Hz, 1H), 7.28 (ddd, *J* = 8.5, 6.7, 1.0 Hz, 1H), 5.06 (d, *J* = 3.0 Hz, 2H) ppm; ^{13}C NMR (150 MHz, $CDCl_3$) δ 148.90, 138.53, 130.34, 129.90, 127.52, 126.71, 124.32, 122.83, 119.57, 119.17 (d, *J* = 1.7 Hz), 118.64, 47.92 (d, *J* = 21.4 Hz) ppm; ^{19}F NMR (376 MHz, $CDCl_3$) δ 55.90 ppm; FT-IR ($CHCl_3$, film) υ 2922, 2853, 1498, 1408, 1204, 812 cm^{-1}; HRMS (EI+) calcd for $C_{14}H_{11}FN_2O_2S$ $[M]^+$

290.0525, found 290.0525.

(5-Methyl-2-(*p*-tolyl)-2*H*-indazol-3-yl)methanesulfonyl fluoride (3k)

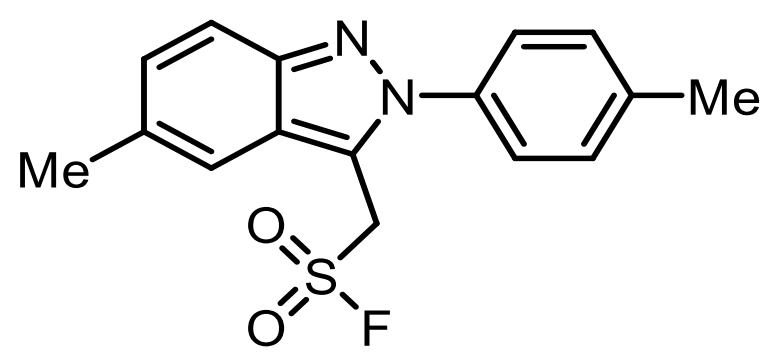

3k

39.6 mg (62%); Red solid; ^{1}H NMR (600 MHz, $CDCl_3$) δ 7.71 (d, *J* = 9.0 Hz, 1H), 7.46 (d, *J* = 5.1 Hz, 2H), 7.44 (s, 1H), 7.37 (d, *J* = 8.3 Hz, 2H), 7.24 (dd, *J* = 8.9, 1.7 Hz, 1H), 5.02 (d, *J* = 3.0 Hz, 2H), 2.49 (s, 3H), 2.48 (s, 3H) ppm; ^{13}C NMR (150 MHz, $CDCl_3$) δ 147.81, 140.48, 136.11, 133.95, 130.39, 130.37, 126.48, 123.12, 118.50, 118.25, 117.10, 48.00 (d, *J* = 20.9 Hz), 22.17, 21.42 ppm; ^{19}F NMR (376 MHz, $CDCl_3$) δ 55.77 ppm; FT-IR ($CHCl_3$, film) υ 2962, 2921, 2859, 1604, 1511, 1406, 1204, 1037, 865 cm^{-1}; HRMS (EI+) calcd for $C_{16}H_{15}FN_2O_2S$ $[M]^+$ 318.0838, found 318.0834.

(5-(Trifluoromethyl)-2-(4-(trifluoromethyl)phenyl)-2*H*-indazol-3-yl)methanesulfonyl fluoride (3l)

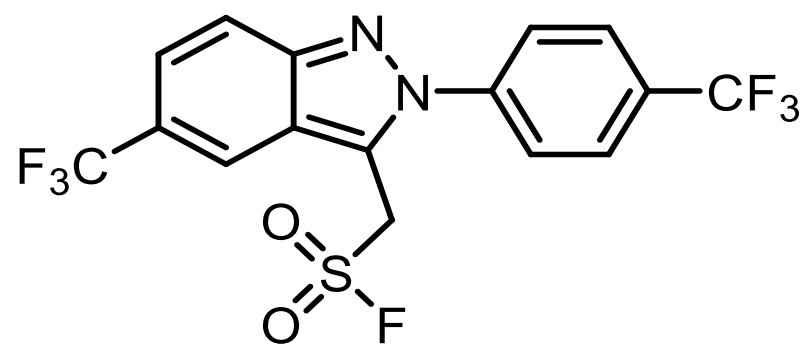

3l

36.3 mg (43%); White solid; ^{1}H NMR (600 MHz, $CDCl_3$) δ 8.12 (s, 1H), 7.95 (d, *J* = 9.1 Hz, 1H), 7.92 (d, *J* = 8.5 Hz, 2H), 7.79 (d, *J* = 8.3 Hz, 2H), 7.61 (dd, *J* = 9.1, 1.7 Hz, 1H), 5.10 (d, *J* = 2.2 Hz, 2H) ppm; ^{13}C NMR (150 MHz, $CDCl_3$) δ 149.54, 141.01, 132.95 (q, *J* = 33.2 Hz), 127.36 (q, *J* = 3.7 Hz), 127.18, 126.94, 125.17, 124.36, 124.00 (q, *J* = 2.9 Hz), 123.37, 122.56, 122.07, 121.80, 120.13, 118.19 (q, *J* = 5.1 Hz), 47.60 (d, *J* = 22.5 Hz) ppm; ^{19}F NMR (376 MHz, $CDCl_3$) δ 56.31, -62.31, -62.73 ppm; FT-IR ($CHCl_3$, film) υ 2922, 1614, 1423, 1390, 1319, 1202, 1156, 1115, 1063, 891, 856, 804 cm^{-1}; HRMS (EI+) calcd for $C_{16}H_9F_7N_2O_2S$ $[M]^+$ 426.0273, found 426.0270.

(5-Chloro-2-(4-chlorophenyl)-2*H*-indazol-3-yl)methanesulfonyl fluoride (3m)

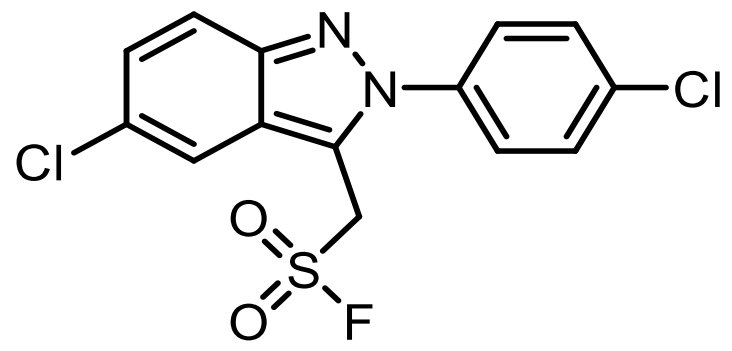

3m

51.0 mg (71%); Yellow solid; ^{1}H NMR (600 MHz, $CDCl_3$) δ 7.74 (d, *J* = 9.5 Hz, 1H), 7.71 (s, 1H), 7.59 – 7.56 (m, 2H), 7.54 – 7.52 (m, 2H), 7.35 (dd, *J* = 9.2, 1.9 Hz, 1H), 4.99 (d, *J* = 2.4 Hz, 2H) ppm; ^{13}C NMR (150 MHz, $CDCl_3$) δ 147.35, 136.80, 136.69, 130.46, 130.21, 129.33, 127.86, 123.36, 120.21, 119.44, 117.91, 47.68 (d, *J* = 22.0 Hz) ppm; ^{19}F NMR (376 MHz, $CDCl_3$) δ 56.12 ppm; FT-IR ($CHCl_3$, film) υ 2919, 2845, 1496, 1405, 1202, 1092, 1013, 840 cm^{-1}; HRMS (EI+) calcd for $C_{14}H_9Cl_2FN_2O_2S$ $[M]^+$ 357.9746, found 357.9748.

(5-Bromo-2-(4-bromophenyl)-2*H*-indazol-3-yl)methanesulfonyl fluoride (3n)

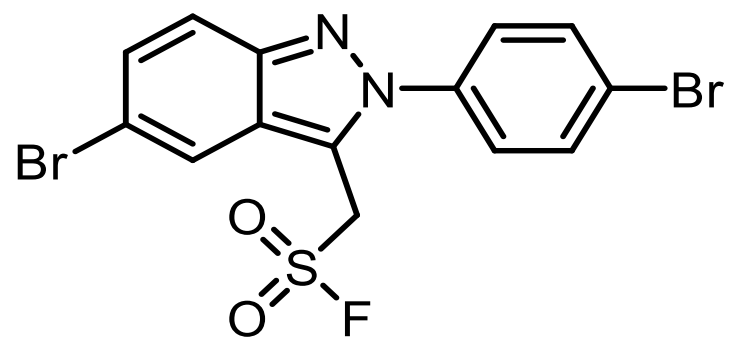

3n

56.5 mg (63%); Yellow solid; ^{1}H NMR (600 MHz, $CDCl_3$) δ 7.91 (s, 1H), 7.76 – 7.73 (m, 2H), 7.69 (d, *J* = 9.1 Hz, 1H), 7.49 – 7.46 (m, 3H), 4.99 (d, *J* = 2.3 Hz, 2H) ppm; ^{13}C NMR (150 MHz, $CDCl_3$) δ 147.48, 137.20, 133.24, 131.65, 128.11, 124.93, 124.12, 121.35, 120.38, 119.23, 118.41, 47.71 (d, *J* = 22.0 Hz) ppm; ^{19}F NMR (376 MHz, $CDCl_3$) δ 56.17 ppm; FT-IR ($CHCl_3$, film) υ 2921, 2847, 1488, 1409, 1256, 1200, 1075, 1009, 835 cm^{-1}; HRMS (EI+) calcd for $C_{14}H_9Br_2FN_2O_2S$ $[M]^+$ 445.8736, found 445.8737.

(5-Acetyl-2-(4-acetylphenyl)-2*H*-indazol-3-yl)methanesulfonyl fluoride (3o)

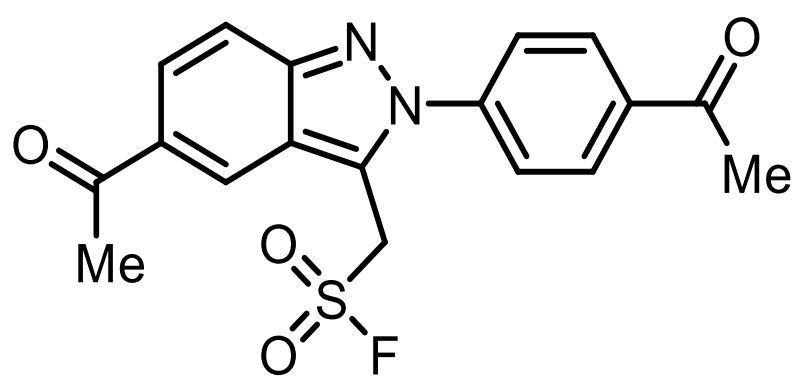

3o

61.6 mg (82%); White solid; ^{1}H NMR (600 MHz, $CDCl_3$) δ 8.45 (s, 1H), 8.21 (d, J = 8.5 Hz, 2H), 8.03 (dd, J = 9.1, 1.6 Hz, 1H), 7.85 (d, J = 9.2 Hz, 1H), 7.76 (d, J = 8.5 Hz, 2H), 5.16 (d, J = 2.0 Hz, 2H), 2.70 (s, 3H), 2.70 (s, 3H) ppm; ^{13}C NMR (150 MHz, $CDCl_3$) δ 197.32, 196.69, 150.32, 141.67, 138.53, 133.87, 131.41, 130.05, 126.77, 126.53, 122.62, 122.53, 119.01, 47.72 (d, J = 22.0 Hz), 26.97, 26.75 ppm; ^{19}F NMR (376 MHz, $CDCl_3$) δ 56.31 ppm; FT-IR ($CHCl_3$, film) υ 2923, 2850, 1684, 1665, 1602, 1489, 1408, 1265, 1198, 809 cm^{-1}; HRMS (EI+) calcd for $C_{18}H_{15}FN_2O_4S$ $[M]^+$ 374.0737, found 374.0737.

Ethyl 2-(4-(ethoxycarbonyl)phenyl)-3-((fluorosulfonyl)methyl)-2*H*-indazole-5-carboxylate (3p)

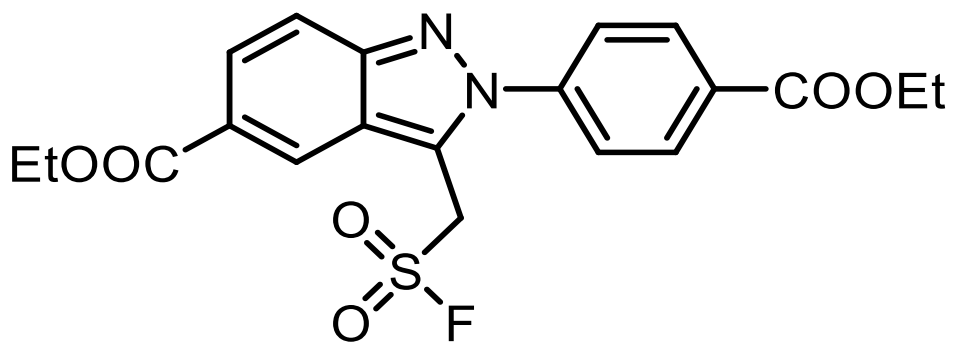

3p

57.3 mg (66%); White solid; ^{1}H NMR (600 MHz, $CDCl_3$) δ 8.57 (s, 1H), 8.29 (d, J = 8.5 Hz, 2H), 8.04 (dd, J = 9.1, 1.6 Hz, 1H), 7.83 (d, J = 9.1 Hz, 1H), 7.71 (d, J = 8.5 Hz, 2H), 5.14 (d, J = 2.6 Hz, 2H), 4.43 (dq, J = 10.8, 7.1 Hz, 4H), 1.43 (td, J = 7.2, 2.5 Hz, 6H) ppm; ^{13}C NMR (150 MHz, $CDCl_3$) δ 166.38, 165.30, 150.25, 141.64, 132.50, 131.30, 127.64, 126.96, 126.48, 123.36, 122.56, 122.30, 118.61, 61.84, 61.45, 47.59 (d, J = 22.0 Hz), 14.49, 14.41 ppm; ^{19}F NMR (376 MHz, $CDCl_3$) δ 56.33 ppm; FT-IR ($CHCl_3$, film) υ 2922, 1719, 1697, 1605, 1402, 1270, 1196, 1105, 815 cm^{-1}; HRMS (EI+) calcd for $C_{20}H_{19}FN_2O_6S$ $[M]^+$ 434.0948, found 434.0944.

(*E*)-2-(3-Methyl-2-(*o*-tolyldiazenyl)phenyl)ethane-1-sulfonyl fluoride (3aa)

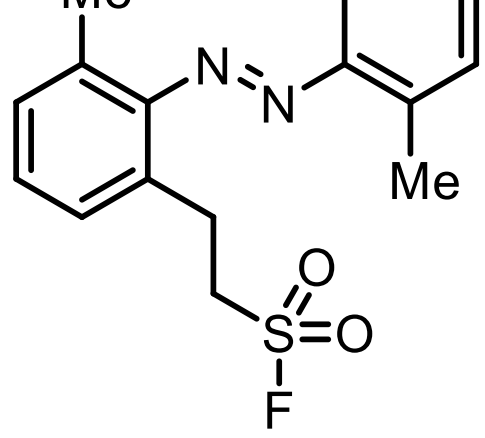

3aa

52.6 mg (82%); Red solid; ^{1}H NMR (600 MHz, $CDCl_3$) δ 7.61 (dd, *J* = 8.0, 1.6 Hz, 1H), 7.45 – 7.40 (m, 2H), 7.34 – 7.32 (m, 1H), 7.29 (dd, *J* = 5.1, 3.8 Hz, 2H), 7.25 (q, *J* = 3.4 Hz, 1H), 3.75 – 3.72 (m, 2H), 3.47 – 3.44 (m, 2H), 2.74 (s, 3H), 2.48 (s, 3H) ppm; ^{13}C NMR (150 MHz, $CDCl_3$) δ 151.28, 150.25, 138.63, 131.91, 131.77, 131.75, 131.52, 131.27, 129.44, 128.73, 126.83, 115.02, 52.13 (d, *J* = 14.8 Hz), 27.29, 20.40, 18.10 ppm; FT-IR ($CHCl_3$, film) υ 3023, 2912, 1502, 1408, 1204, 869 cm^{-1}; HRMS (ESI-) calcd for $C_{16}H_{16}FN_2O_2S$ $[M-H]^-$ 319.0917, found 319.0921.

(2-(2,3-Dimethylphenyl)-6,7-dimethyl-2*H*-indazol-3-yl)methanesulfonyl fluoride (5a)

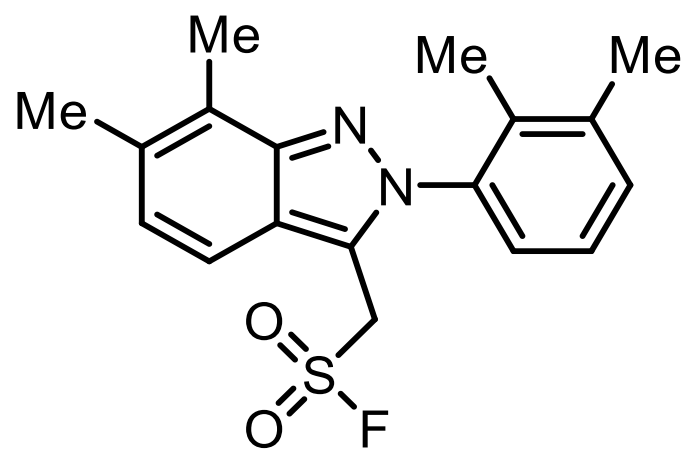

5a

44.2 mg (64%); Yellow solid; ^{1}H NMR (600 MHz, $CDCl_3$) δ 7.48 (d, *J* = 8.5 Hz, 1H), 7.36 (d, *J* = 7.5 Hz, 1H), 7.26 (t, *J* = 8.0 Hz, 1H), 7.20 (d, *J* = 7.7 Hz, 1H), 7.11 (d, *J* = 8.6 Hz, 1H), 4.96 (dd, *J* = 15.3, 2.9 Hz, 1H), 4.78 (dd, *J* = 15.3, 4.2 Hz, 1H), 2.58 (s, 3H), 2.42 (s, 3H), 2.36 (s, 3H), 1.83 (s, 3H) ppm; ^{13}C NMR (150 MHz, $CDCl_3$) δ 149.88, 139.26, 137.33, 135.17, 133.47, 132.00, 128.26, 126.40, 125.55, 125.30, 120.63, 120.16, 115.69, 47.67 (d, *J* = 20.9 Hz), 20.37, 19.38, 14.11, 13.22 ppm; ^{19}F NMR (376 MHz, $CDCl_3$) δ 55.54 ppm; FT-IR ($CHCl_3$, film) υ 2921, 1485, 1404, 1200, 1016, 842 cm^{-1}; HRMS (EI+) calcd for $C_{18}H_{19}FN_2O_2S$ $[M]^+$ 346.1151, found 346.1147.

(6-Fluoro-2-(3-fluoro-2-methylphenyl)-7-methyl-2*H*-indazol-3-yl)methanesulfonyl fluoride (5b)

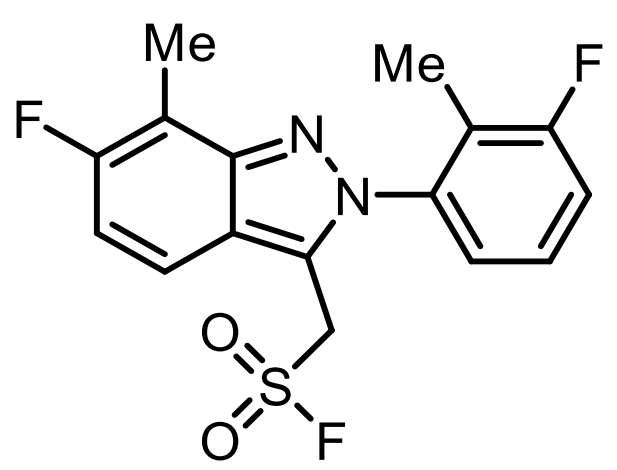

5b

51.3 mg (72%); Pale green solid; ^{1}H NMR (600 MHz, $CDCl_3$) δ 7.55 (dd, *J* = 9.2, 4.8 Hz, 1H), 7.40 – 7.37 (m, 1H), 7.30 (t, *J* = 8.7 Hz, 1H), 7.22 (d, *J* = 7.8 Hz, 1H), 7.09 (t, *J* = 9.4 Hz, 1H), 4.90 (d, *J* = 88.5 Hz, 2H), 2.54 (d, *J* = 2.0 Hz, 3H), 1.93 (d, *J* = 2.1 Hz, 3H) ppm; ^{13}C NMR (150 MHz, $CDCl_3$) δ 162.60, 160.95, 159.87, 158.26, 149.83 (d, *J* = 10.5 Hz), 138.40 (d, *J* = 6.4 Hz), 127.69 (d, *J* = 9.5 Hz), 124.93 (d, *J* = 19.4 Hz), 123.57 (d, *J* = 3.6 Hz), 117.82 (d, *J* = 5.0 Hz), 117.71 (d, *J* = 7.4 Hz), 116.55 (d, *J* = 29.8 Hz), 111.97 (d, *J* = 20.0 Hz), 47.39 (d, *J* = 22.0 Hz), 9.82 (d, *J* = 4.1 Hz), 8.89 (d, *J* = 3.8 Hz) ppm; ^{19}F NMR (376 MHz, $CDCl_3$) δ 55.77, -112.29, -118.85 ppm; FT-IR ($CHCl_3$, film) υ 2923, 2852, 1583, 1409, 1245, 1199, 792 cm^{-1}; HRMS (EI+) calcd for $C_{16}H_{13}F_3N_2O_2S$ $[M]^+$ 354.0650, found 354.0651.

(6-Chloro-2-(3-chloro-2-methylphenyl)-7-methyl-2*H*-indazol-3-yl)methanesulfonyl fluoride (5c)

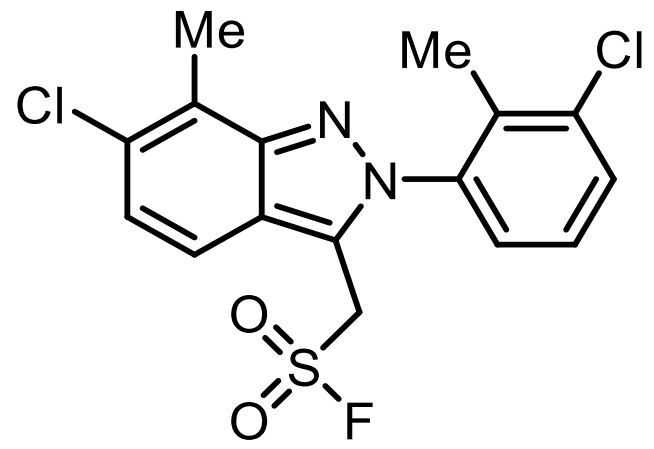

5c

42.5 mg (55%); Pale green solid; ^{1}H NMR (600 MHz, $CDCl_3$) δ 7.63 (dd, *J* = 7.7, 1.7 Hz, 1H), 7.51 (d, *J* = 8.9 Hz, 1H), 7.37 (d, *J* = 7.7 Hz, 1H), 7.35 – 7.32 (m, 1H), 7.27 – 7.25 (m, 1H), 4.98 (d, *J* = 15.2 Hz, 1H), 4.77 (dd, *J* = 15.4, 3.9 Hz, 1H), 2.68 (s, 3H), 1.98 (s, 3H) ppm; ^{13}C NMR (150 MHz, $CDCl_3$) δ 149.77, 138.13, 136.56, 135.40, 131.90, 127.53, 126.87, 126.56, 126.31, 121.25, 120.53, 117.32, 47.37 (d, *J* = 22.0 Hz), 15.14, 13.99 ppm; ^{19}F NMR (376 MHz, $CDCl_3$) δ 55.82 ppm; FT-IR ($CHCl_3$, film) υ 2922, 1484, 1414, 1384, 1207, 1100, 999, 886, 786, 754, 712 cm^{-1}; HRMS (EI+) calcd for $C_{16}H_{13}Cl_2FN_2O_2S$ $[M]^+$ 386.0059, found 386.0059.

(7-Fluoro-2-(2-fluoro-3-methylphenyl)-6-methyl-2*H*-indazol-3-yl)methanesulfonyl fluoride (5d)

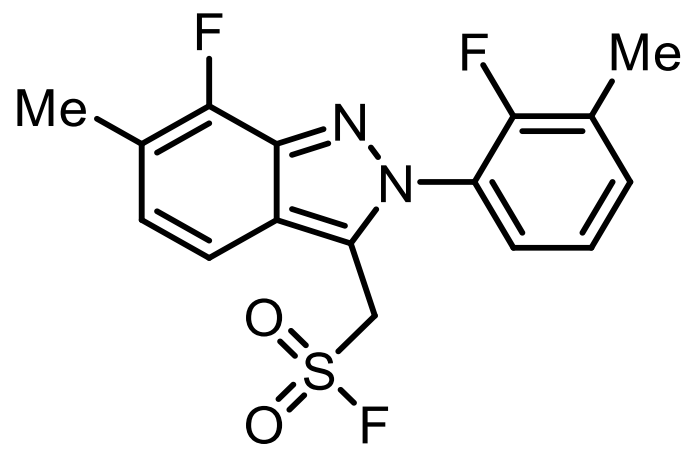

5d

49.7 mg (70%); Yellow solid; ^{1}H NMR (600 MHz, $CDCl_3$) δ 7.44 (q, J = 7.0 Hz, 2H), 7.39 (d, J = 8.6 Hz, 1H), 7.27 – 7.25 (m, 1H), 7.07 (dd, J = 8.6, 6.0 Hz, 1H), 4.97 (d, J = 3.6 Hz, 2H), 2.41 (d, J = 2.5 Hz, 3H), 2.39 (d, J = 2.0 Hz, 3H) ppm; ^{13}C NMR (150 MHz, $CDCl_3$) δ 156.00, 154.33, 151.10, 149.41, 141.19 (d, J = 17.5 Hz), 134.05 (d, J = 5.0 Hz), 128.62 (d, J = 3.3 Hz), 127.22, 127.01 (d, J = 15.6 Hz), 125.93 (d, J = 12.8 Hz), 124.77 (d, J = 4.7 Hz), 123.93 (d, J = 4.9 Hz), 121.76 (d, J = 3.1 Hz), 120.01 (d, J = 12.6 Hz), 114.28 (d, J = 5.4 Hz), 47.49 (d, J = 22.2 Hz), 14.69 (d, J = 3.5 Hz), 14.15 (d, J = 3.8 Hz) ppm; ^{19}F NMR (376 MHz, $CDCl_3$) δ 55.53, -126.77, -133.38 ppm; FT-IR ($CHCl_3$, film) υ 2924, 2850, 1545, 1499, 1406, 1221, 1208, 1068, 859 cm^{-1}; HRMS (EI+) calcd for $C_{16}H_{13}F_3N_2O_2S$ $[M]^+$ 354.0650, found 354.0648.

(2-(2,4-Dimethylphenyl)-5,7-dimethyl-2*H*-indazol-3-yl)methanesulfonyl fluoride (5e)

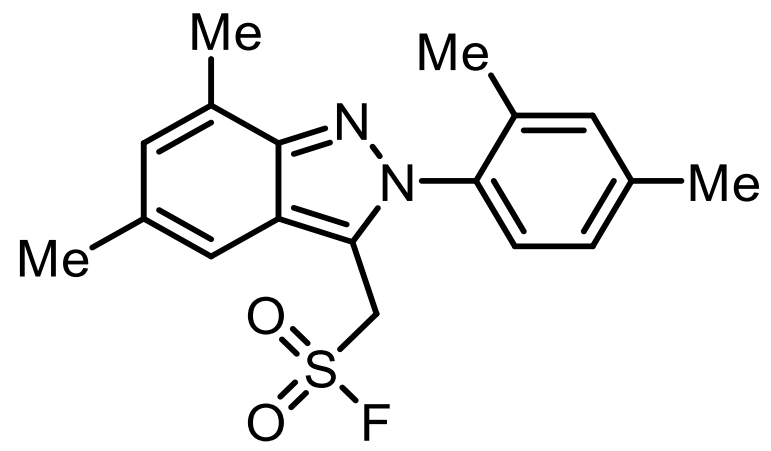

5e

59.0 mg (85%); Yellow gummy solid; ^{1}H NMR (600 MHz, $CDCl_3$) δ 7.22 (s, 1H), 7.16 (d, J = 7.9 Hz, 1H), 7.11 (s, 1H), 7.07 (d, J = 7.9 Hz, 1H), 6.93 (s, 1H), 4.77 (d, J = 76.7 Hz, 2H), 2.54 (s, 3H), 2.36 (s, 3H), 2.33 (s, 3H), 1.87 (s, 3H) ppm; ^{13}C NMR (150 MHz, $CDCl_3$) δ 148.14, 140.78, 136.07, 134.77, 134.06, 132.23, 129.22, 128.43, 127.63, 127.59, 122.31, 119.16, 114.54, 47.64 (d, J = 20.9 Hz), 22.09, 21.34, 17.06, 17.01 ppm; ^{19}F NMR (376 MHz, $CDCl_3$) δ 55.52 ppm; FT-IR ($CHCl_3$, film) υ 2960, 2917, 2856, 1508, 1403, 1268, 1209, 813 cm^{-1}; HRMS (EI+) calcd for $C_{18}H_{19}FN_2O_2S$ $[M]^+$ 346.1151, found 346.1150.

(5-Fluoro-2-(4-fluoro-2-methylphenyl)-7-methyl-2*H*-indazol-3-yl)methanesulfonyl

fluoride (5f)

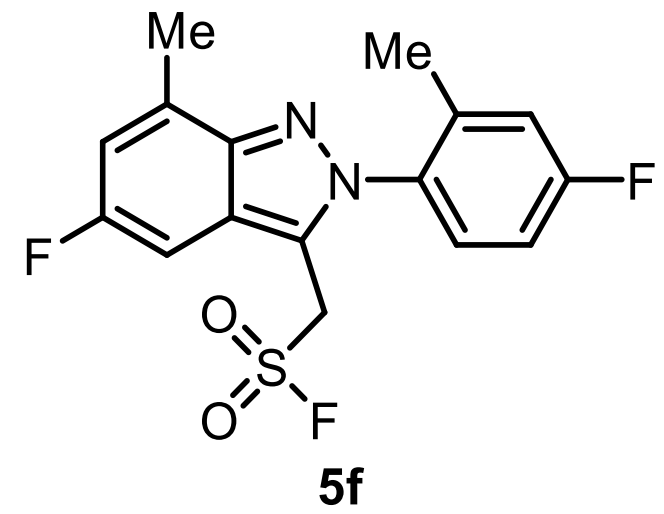

5f

46.9 mg (66%); Brown solid; ^{1}H NMR (600 MHz, $CDCl_3$) δ 7.38 (dd, *J* = 8.5, 5.1 Hz, 1H), 7.14 (d, *J* = 9.0 Hz, 2H), 7.10 (td, *J* = 8.1, 3.0 Hz, 1H), 7.00 (dd, *J* = 9.8, 1.1 Hz, 1H), 4.84 (d, *J* = 94.0 Hz, 2H), 2.65 (s, 3H), 2.00 (s, 3H) ppm; ^{13}C NMR (150 MHz, $CDCl_3$) δ 163.57 (d, *J* = 250.0 Hz), 159.91 (d, *J* = 242.8 Hz), 146.80, 139.42 (d, *J* = 9.3 Hz), 133.15 (d, *J* = 3.3 Hz), 132.05 (d, *J* = 9.9 Hz), 129.76 (d, *J* = 9.3 Hz), 121.57 (d, *J* = 12.1 Hz), 120.68 (d, *J* = 8.8 Hz), 118.48 (d, *J* = 22.0 Hz), 118.20 (d, *J* = 28.0 Hz), 114.18 (d, *J* = 22.5 Hz), 99.15 (d, *J* = 24.7 Hz), 47.50 (d, *J* = 21.4 Hz), 17.43, 17.03 ppm; ^{19}F NMR (376 MHz, $CDCl_3$) δ 55.72, -109.36, -115.45 ppm; FT-IR ($CHCl_3$, film) υ 2992, 2925, 2851, 1504, 1408, 1273, 1225, 882 cm^{-1}; HRMS (EI+) calcd for $C_{16}H_{13}F_3N_2O_2S$ $[M]^+$ 354.0650, found 354.0646.

(5-Chloro-2-(4-chloro-2-methylphenyl)-7-methyl-2*H*-indazol-3-yl)methanesulfonyl fluoride (5g)

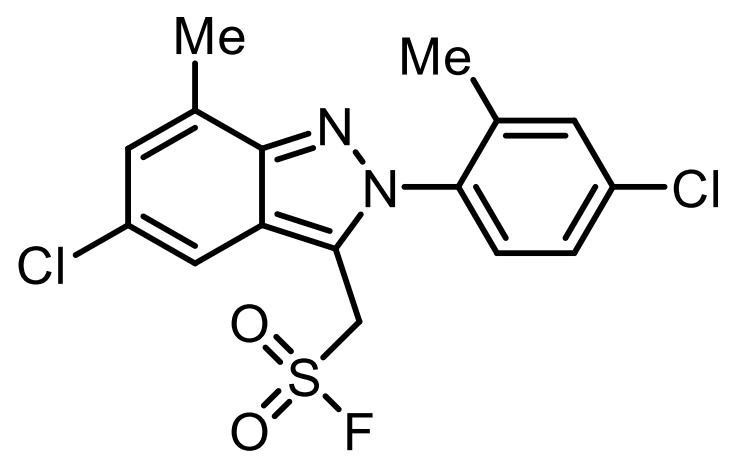

5g

64.0 mg (82%); Pale yellow solid; ^{1}H NMR (600 MHz, $CDCl_3$) δ 7.55 (d, *J* = 1.9 Hz, 1H), 7.43 (d, *J* = 2.3 Hz, 1H), 7.38 (dd, *J* = 8.3, 2.4 Hz, 1H), 7.32 (d, *J* = 8.4 Hz, 1H), 7.14 (t, *J* = 1.6 Hz, 1H), 4.87 (s, 2H), 2.63 (s, 3H), 1.99 (s, 3H) ppm; ^{13}C NMR (150 MHz, $CDCl_3$) δ 147.87, 138.45, 136.87, 135.47, 131.75, 131.04, 130.40, 129.02, 127.93, 127.34, 122.41, 120.12, 115.23, 47.33 (d, *J* = 21.9 Hz), 17.18, 16.83 ppm; ^{19}F NMR (376 MHz, $CDCl_3$) δ 55.86 ppm; FT-IR ($CHCl_3$, film) υ 2918, 2852, 1494, 1411, 1347, 1202, 888, 792 cm^{-1}; HRMS (EI+) calcd

for $C_{16}H_{13}Cl_2FN_2O_2S$ $[M]^+$ 386.0059, found 386.0054.

(7-Fluoro-2-(2-fluoro-4-methylphenyl)-5-methyl-2*H*-indazol-3-yl)methanesulfonyl fluoride (5h)

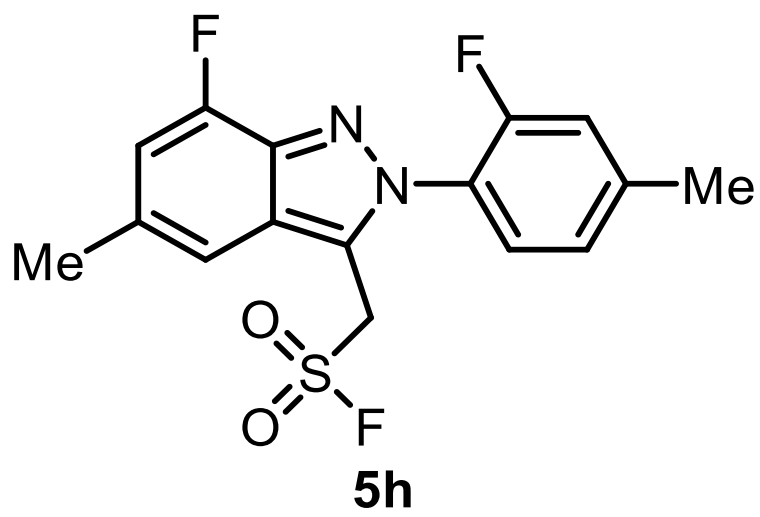

5h

48.5 mg (68%); Yellow solid; ^{1}H NMR (600 MHz, $CDCl_3$) δ 7.50 (t, *J* = 7.9 Hz, 1H), 7.23 (s, 1H), 7.16 (d, *J* = 8.1 Hz, 1H), 7.11 (d, *J* = 10.8 Hz, 1H), 6.89 (d, *J* = 11.7 Hz, 1H), 4.96 (d, *J* = 3.5 Hz, 2H), 2.47 (s, 3H), 2.46 (s, 3H) ppm; ^{13}C NMR (150 MHz, $CDCl_3$) δ 156.96, 155.29, 153.56, 151.85, 143.87 (d, *J* = 7.7 Hz), 139.74 (d, *J* = 17.0 Hz), 135.00 (d, *J* = 5.3 Hz), 129.43, 126.16 (d, *J* = 3.4 Hz), 125.45 (d, *J* = 5.5 Hz), 123.38 (d, *J* = 12.2 Hz), 121.07 (d, *J* = 3.5 Hz), 117.29 (d, *J* = 18.9 Hz), 113.14, 113.02 (d, *J* = 2.9 Hz), 112.99, 47.43 (d, *J* = 18.9 Hz), 22.16, 21.57 ppm; ^{19}F NMR (376 MHz, $CDCl_3$) δ 55.50, -123.43, -128.35 (d, *J* = 17.9 Hz) ppm; FT-IR ($CHCl_3$, film) υ 2923, 2852, 1557, 1521, 1410, 1208, 819 cm^{-1}; HRMS (EI+) calcd for $C_{16}H_{13}F_3N_2O_2S$ $[M]^+$ 354.0650, found 354.0650.

(7-Bromo-2-(2-bromo-4-methylphenyl)-5-methyl-2H-indazol-3-yl)methanesulfonyl fluoride (5i)

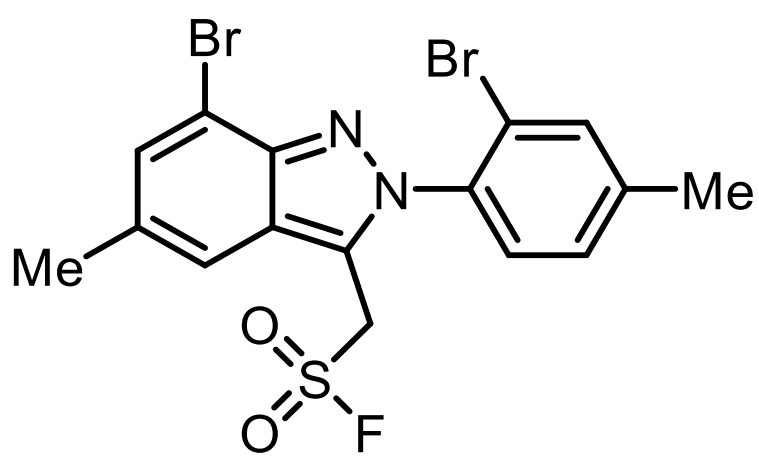

5i

20.4 mg (21%); Pale yellow solid; ^{1}H NMR (600 MHz, $CDCl_3$) δ 7.57 (s, 1H), 7.53 (d, *J* = 8.0 Hz, 1H), 7.51 (s, 1H), 7.43 (s, 1H), 7.34 (d, *J* = 8.0 Hz, 1H), 5.07 (d, *J* = 15.5 Hz, 1H), 4.66 (dd, *J* = 15.5, 5.4 Hz, 1H), 2.47 (d, *J* = 4.8 Hz, 6H) ppm; ^{13}C NMR (150 MHz, $CDCl_3$) δ 146.83, 143.34, 135.19, 134.70, 133.80, 133.21, 130.53, 129.64, 123.20, 121.32, 120.91, 116.80,

111.95, 47.85 (d, J = 21.4 Hz), 21.84, 21.27 ppm; ^{19}F NMR (376 MHz, $CDCl_3$) δ 55.66 ppm; FT-IR ($CHCl_3$, film) υ 2954, 2918, 2850, 1406, 1267, 1204, 1040, 816 cm^{-1}; HRMS (EI) calcd for $C_{16}H_{13}Br_2FN_2O_2S$ [M]$^+$ 473.9049, found 473.9047.

(4-Fluoro-2-(5-fluoro-2-methylphenyl)-7-methyl-2*H*-indazol-3-yl)methanesulfonyl fluoride (5j)

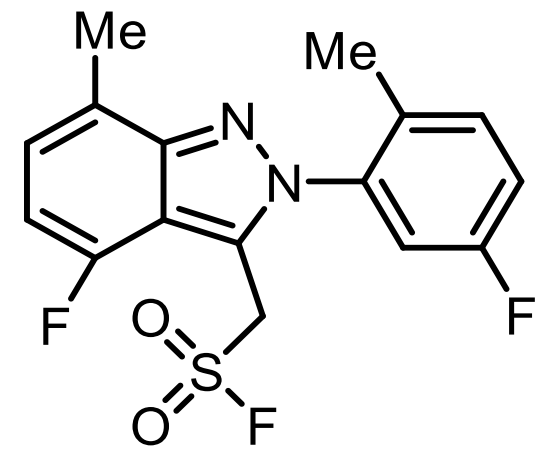

5j

14.2 mg (20%); Brown solid; ^{1}H NMR (600 MHz, $CDCl_3$) δ 7.40 (dd, J = 8.8, 5.7 Hz, 1H), 7.24 (dd, J = 8.3, 2.7 Hz, 1H), 7.18 (dd, J = 8.1, 2.7 Hz, 1H), 7.06 (ddd, J = 7.6, 4.9, 1.3 Hz, 1H), 6.80 (dd, J = 10.7, 7.6 Hz, 1H), 5.25 – 4.80 (m, 2H), 2.59 (s, 3H), 2.00 (s, 3H) ppm; ^{13}C NMR (150 MHz, $CDCl_3$) δ 160.89 (d, J = 246.7 Hz), 153.61 (d, J = 249.5 Hz), 150.71 (d, J = 5.0 Hz), 137.36 (d, J = 9.3 Hz), 132.94 (d, J = 8.2 Hz), 132.22 (d, J = 3.3 Hz), 126.12 (d, J = 6.6 Hz), 124.91 (d, J = 5.5 Hz), 119.57 (d, J = 6.0 Hz), 118.20 (d, J = 20.9 Hz), 115.48 (d, J = 24.2 Hz), 113.90 (d, J = 18.7 Hz), 107.27 (d, J = 17.0 Hz), 47.68 (d, J = 18.7 Hz), 16.68, 16.52 ppm; ^{19}F NMR (376 MHz, $CDCl_3$) δ 55.15 (d, J = 22.6 Hz), -114.12, -126.05 ppm; FT-IR ($CHCl_3$, film) υ 2923, 2854, 1573, 1508, 1409, 1389, 1246, 1206, 1160, 866, 804 cm^{-1}; HRMS (EI+) calcd for $C_{16}H_{13}F_3N_2O_2S$ [M]$^+$ 354.0650, found 354.0650.

(7-Fluoro-2-(2-fluoro-5-methylphenyl)-4-methyl-2*H*-indazol-3-yl)methanesulfonyl fluoride (5k)

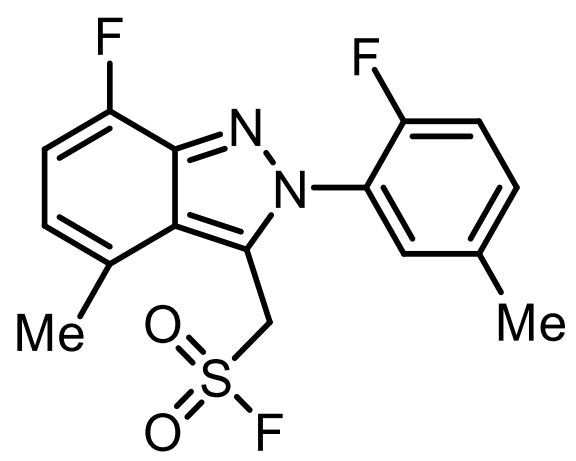

5k

24.8 mg (35%); White solid; ^{1}H NMR (600 MHz, $CDCl_3$) δ 7.48 (dd, J = 7.0, 2.6 Hz, 1H), 7.39

– 7.36 (m, 1H), 7.20 (dd, *J* = 9.8, 8.5 Hz, 1H), 6.93 – 6.91 (m, 1H), 6.89 – 6.86 (m, 1H), 5.16 (s, 2H), 2.68 (s, 3H), 2.43 (s, 3H) ppm; ^{13}C NMR (150 MHz, $CDCl_3$) δ 153.95 (d, *J* = 247.8 Hz), 151.78 (d, *J* = 253.9 Hz), 140.99 (d, *J* = 17.0 Hz), 135.72 (d, *J* = 3.8 Hz), 132.92 (d, *J* = 7.2 Hz), 130.30, 125.61 (d, *J* = 12.6 Hz), 125.37 (d, *J* = 5.0 Hz), 124.81 (d, *J* = 4.4 Hz), 124.45 (d, *J* = 5.5 Hz), 122.09 (d, *J* = 3.3 Hz), 116.33 (d, *J* = 19.2 Hz), 109.97 (d, *J* = 15.4 Hz), 47.99 (dd, *J* = 20.9, 3.8 Hz), 20.63, 18.80 ppm; ^{19}F NMR (376 MHz, $CDCl_3$) δ 55.52, -127.88, -131.84 ppm; FT-IR ($CHCl_3$, film) υ 2918, 2847, 1541, 1513, 1420, 1205, 1018, 869, 818 cm^{-1}; HRMS (EI+) calcd for $C_{16}H_{13}F_3N_2O_2S$ [M]$^+$ 354.0650, found 354.0647.

(5-Fluoro-2-(4-fluoro-3-methylphenyl)-6-methyl-2*H*-indazol-3-yl)methanesulfonyl fluoride (5l)

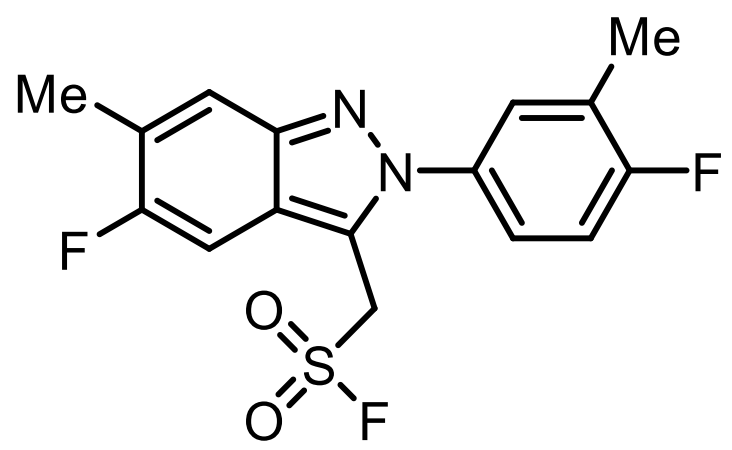

5l

43.3 mg (61%); Pale yellow solid; ^{1}H NMR (600 MHz, $CDCl_3$) δ 7.58 (d, *J* = 6.7 Hz, 1H), 7.42 (dd, *J* = 6.6, 2.9 Hz, 1H), 7.38 – 7.35 (m, 1H), 7.26 (d, *J* = 9.7 Hz, 1H), 7.20 (t, *J* = 8.8 Hz, 1H), 4.97 (d, *J* = 2.8 Hz, 2H), 2.42 (d, *J* = 1.4 Hz, 3H), 2.37 (d, *J* = 2.2 Hz, 3H) ppm; ^{13}C NMR (150 MHz, $CDCl_3$) δ 162.01 (d, *J* = 247.8 Hz), 159.56 (d, *J* = 244.5 Hz), 146.40, 134.11 (d, *J* = 3.3 Hz), 129.95 (d, *J* = 6.0 Hz), 129.35 (d, *J* = 23.6 Hz), 127.26 (d, *J* = 19.2 Hz), 125.62 (d, *J* = 8.8 Hz), 121.29 (d, *J* = 12.1 Hz), 119.64 (d, *J* = 5.5 Hz), 119.53 (d, *J* = 8.3 Hz), 116.31 (d, *J* = 24.2 Hz), 101.39 (d, *J* = 26.9 Hz), 47.90 (d, *J* = 21.4 Hz), 16.33 (d, *J* = 4.9 Hz), 14.74 (d, *J* = 3.3 Hz) ppm; ^{19}F NMR (376 MHz, $CDCl_3$) δ 55.81, -113.73, -118.61 ppm; FT-IR ($CHCl_3$, film) υ 2960, 2919, 2900, 1586, 1506, 1408, 1207, 1172, 854, 830, 794, 761 cm^{-1}; HRMS (EI+) calcd for $C_{16}H_{13}F_3N_2O_2S$ [M]$^+$ 354.0650, found 354.0650.

(5-Chloro-2-(4-chloro-3-methylphenyl)-6-methyl-2*H*-indazol-3-yl)methanesulfonyl fluoride (5m)

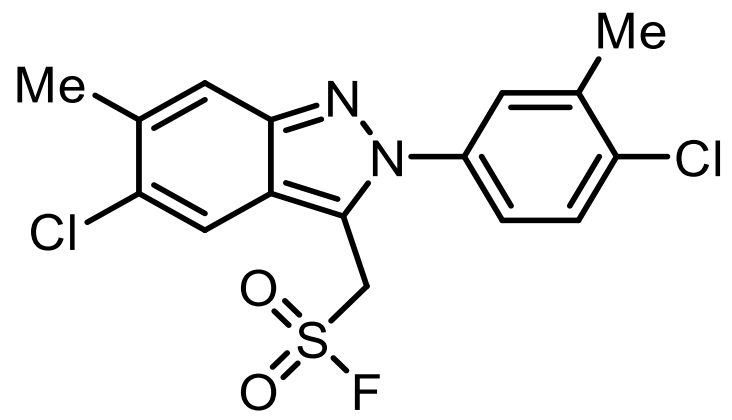

5m

53.6 mg (69%); Yellow solid; ^{1}H NMR (600 MHz, $CDCl_3$) δ 7.74 (s, 1H), 7.64 (s, 1H), 7.55 (d, J = 8.5 Hz, 1H), 7.47 (d, J = 2.6 Hz, 1H), 7.35 (dd, J = 8.4, 2.6 Hz, 1H), 4.99 (d, J = 2.6 Hz, 2H), 2.51 (d, J = 1.3 Hz, 3H), 2.47 (s, 3H) ppm; ^{13}C NMR (150 MHz, $CDCl_3$) δ 148.01, 138.50, 136.73, 136.65, 136.53, 132.43, 130.39, 128.96, 124.96, 122.18, 118.95, 118.22, 118.21, 47.83 (d, J = 21.9 Hz), 21.53, 20.35 ppm; ^{19}F NMR (376 MHz, $CDCl_3$) δ 56.02 ppm; FT-IR ($CHCl_3$, film) υ 2957, 2921, 2853, 1488, 1409, 1203, 1051, 826, 796 cm^{-1}; HRMS (EI+) calcd for $C_{16}H_{13}Cl_2FN_2O_2S$ [M]$^+$ 386.0059, found 386.0057.

(2-(3,5-Dimethylphenyl)-4,6-dimethyl-2*H*-indazol-3-yl)methanesulfonyl fluoride (5n)

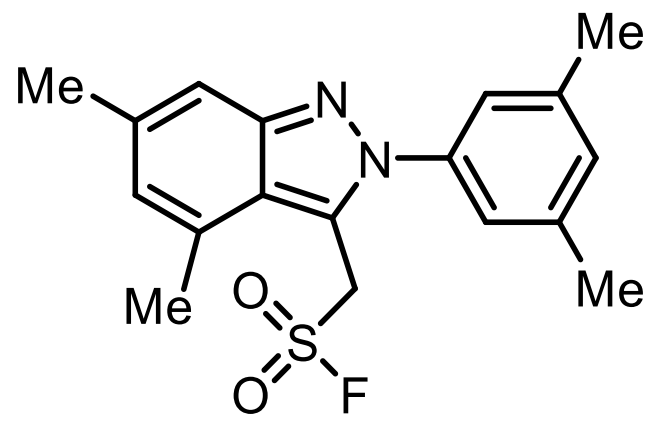

5n

19.9 mg (29%); Pale green solid; ^{1}H NMR (600 MHz, $CDCl_3$) δ 7.38 (s, 1H), 7.18 (s, 1H), 7.10 (s, 2H), 6.84 (s, 1H), 5.21 (d, J = 2.9 Hz, 2H), 2.68 (s, 3H), 2.43 (s, 3H), 2.40 (s, 6H) ppm; ^{13}C NMR (150 MHz, $CDCl_3$) δ 149.85, 139.74, 138.54, 137.30, 131.78, 130.95, 129.02, 127.85, 124.63, 123.80, 121.25, 119.34, 114.73, 48.93 (d, J = 19.8 Hz), 22.08, 21.32, 19.35 (d, J = 1.6 Hz) ppm; ^{19}F NMR (376 MHz, $CDCl_3$) δ 55.58 ppm; FT-IR ($CHCl_3$, film) υ 2918, 2852, 1595, 1483, 1407, 1205, 1166, 1083, 1032, 836, 805, 696 cm^{-1}; HRMS (EI+) calcd for $C_{18}H_{19}FN_2O_2S$ [M]$^+$ 346.1151, found 346.1153.

(2-(3,5-Dimethoxyphenyl)-4,6-dimethoxy-2*H*-indazol-3-yl)methanesulfonyl fluoride (5o)

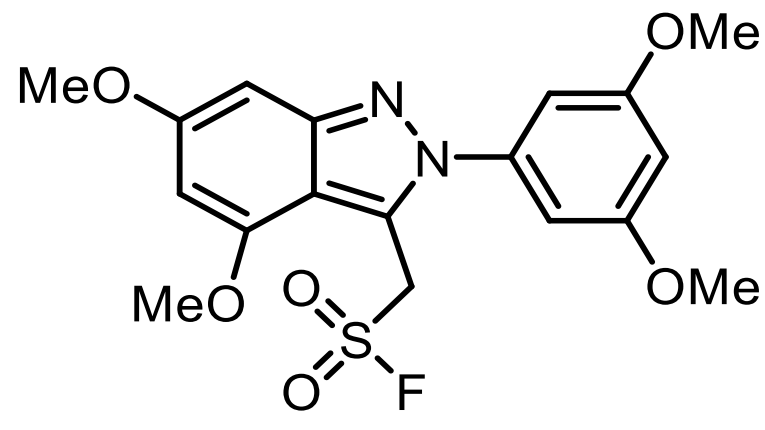

5o

35.5 mg (43%); White solid; ^{1}H NMR (600 MHz, $CDCl_3$) δ 6.68 (d, J = 2.2 Hz, 2H), 6.60 - 6.59 (m, 2H), 6.18 (d, J = 1.8 Hz, 1H), 5.24 (d, J = 4.4 Hz, 2H), 3.93 (s, 3H), 3.86 (s, 3H), 3.82 (s, 6H) ppm; ^{13}C NMR (150 MHz, $CDCl_3$) δ 161.34, 160.97, 153.80, 150.54, 139.96, 120.29, 112.72, 104.94, 102.26, 96.58, 87.80, 55.83, 55.75, 55.66, 48.39 (d, J = 19.8 Hz) ppm; ^{19}F NMR (376 MHz, $CDCl_3$) δ 55.83 ppm; FT-IR ($CHCl_3$, film) υ 3015, 2969, 2942, 2887, 2843, 1633, 1600, 1568, 1492, 1474, 1399, 1336, 1294, 1254, 1219, 1201, 1141, 1111, 1051, 1034, 820, 689 cm^{-1}; HRMS (EI+) calcd for $C_{18}H_{19}FN_2O_6S$ $[M]^+$ 410.0948, found 410.0946.

(4,6-Dichloro-2-(3,5-dichlorophenyl)-2*H*-indazol-3-yl)methanesulfonyl fluoride (5p)

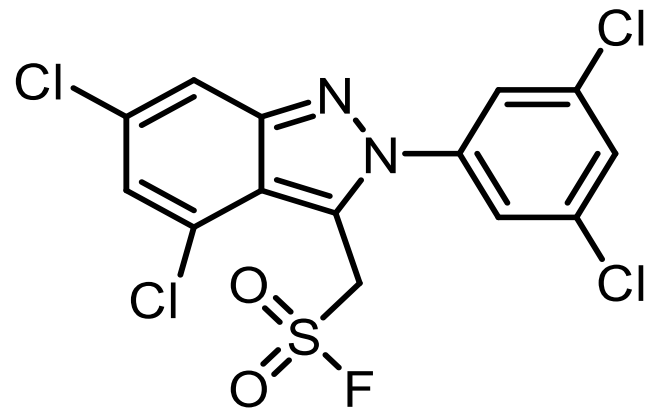

5p

31.2 mg (36%); Pale blue solid; ^{1}H NMR (600 MHz, $CDCl_3$) δ 7.71 (d, J = 1.6 Hz, 1H), 7.63 (t, J = 1.8 Hz, 1H), 7.48 (d, J = 1.9 Hz, 2H), 7.27 (d, J = 1.4 Hz, 1H), 5.38 (d, J = 3.6 Hz, 2H) ppm; ^{13}C NMR (150 MHz, $CDCl_3$) δ 149.58, 139.48, 136.46, 133.55, 131.18, 126.34, 125.85, 125.61, 121.67, 119.65, 116.75, 47.40 (d, J = 20.9 Hz) ppm; ^{19}F NMR (376 MHz, $CDCl_3$) δ 56.78 ppm; FT-IR ($CHCl_3$, film) υ 3092, 2919, 2847, 1623, 1579, 1535, 1470, 1437, 1407, 1207, 1099, 1068, 986, 862, 842, 806, 757 cm^{-1}; HRMS (EI+) calcd for $C_{14}H_7Cl_4FN_2O_2S$ $[M]^+$ 425.8966, found 425.8965.

(5,7-Dimethyl-2-(*o*-tolyl)-2*H*-indazol-3-yl)methanesulfonyl fluoride (**5q**) and (2-(2,4-dimethylphenyl)-7-methyl-2H-indazol-3-yl)methanesulfonyl fluoride (**5q'**): 56.0 mg (84%); **5q**:**5q'** = 1.3:1; Yellow solid; ^{1}H NMR (600 MHz, $CDCl_3$) δ 7.57 (d, J = 8.1 Hz, 1H), 7.50 - 7.46 (m, 1H), 7.41 (d, J = 7.5 Hz, 1H), 7.38 (d, J = 3.5 Hz, 2H), 7.32 (s, 1H), 7.26 (s, 1H), 7.25

(s, 1H), 7.22 (s, 1H), 7.22 – 7.16 (m, 2H), 7.03 (s, 1H), 4.89 (d, *J* = 74.5 Hz, 4H), 2.67 (s, 2H), 2.63 (s, 3H), 2.46 (s, 3H), 2.43 (s, 2H), 2.01 (s, 3H), 1.97 (s, 2H) ppm; ^{13}C NMR (150 MHz, $CDCl_3$) δ 149.17, 148.24, 140.95, 137.32, 136.57, 136.03, 134.70, 134.26, 132.30, 131.70, 130.69, 129.39, 128.93, 128.48, 127.94, 127.68, 127.63, 127.05, 126.23, 124.40, 122.40, 121.96, 120.22, 119.15, 116.56, 114.54, 47.72, 47.58, 22.14, 21.38, 17.18, 17.13, 17.12, 17.04 ppm; ^{19}F NMR (376 MHz, $CDCl_3$) δ 55.64, 55.57 ppm; HRMS (EI+) calcd for $C_{17}H_{17}FN_2O_2S$ $[M]^+$ 332.0995, found 332.0991.

7-Methyl-3-((pyrrolidin-1-ylsulfonyl)methyl)-2-(*o*-tolyl)-2*H*-indazole (6a)

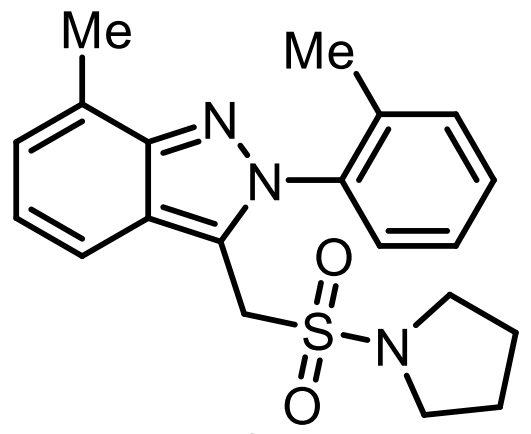

6a

30.0 mg (81%); White solid; ^{1}H NMR (600 MHz, $CDCl_3$) δ 7.63 – 7.60 (m, 1H), 7.47 – 7.43 (m, 2H), 7.39 – 7.35 (m, 2H), 7.12 – 7.10 (m, 2H), 4.56 (s, 2H), 3.12 – 3.10 (m, 4H), 2.65 (s, 3H), 2.02 (s, 3H), 1.81 – 1.79 (m, 4H) ppm; ^{13}C NMR (150 MHz, $CDCl_3$) δ 148.94, 137.95, 136.22, 131.28, 130.11, 128.33, 128.13, 126.55, 125.84, 124.77, 123.14, 121.58, 117.37, 48.07, 47.19, 25.89, 17.32, 17.12 ppm; FT-IR ($CHCl_3$, film) υ 3059, 2957, 2922, 2860, 1619, 1529, 1501, 1459, 1371, 1333, 1246, 1149, 1127, 1071, 1014, 945, 868, 766 cm^{-1}; HRMS (EI+) calcd for $C_{20}H_{23}N_3O_2S$ $[M]^+$ 369.1511, found 369.1507.

Methyl *N*^{2}-(tert-butoxycarbonyl)-*N*^{6}-(((7-methyl-2-(*o*-tolyl)-2*H*-indazol-3-yl)methyl)sulfonyl)-*L*-lysinate (6b)

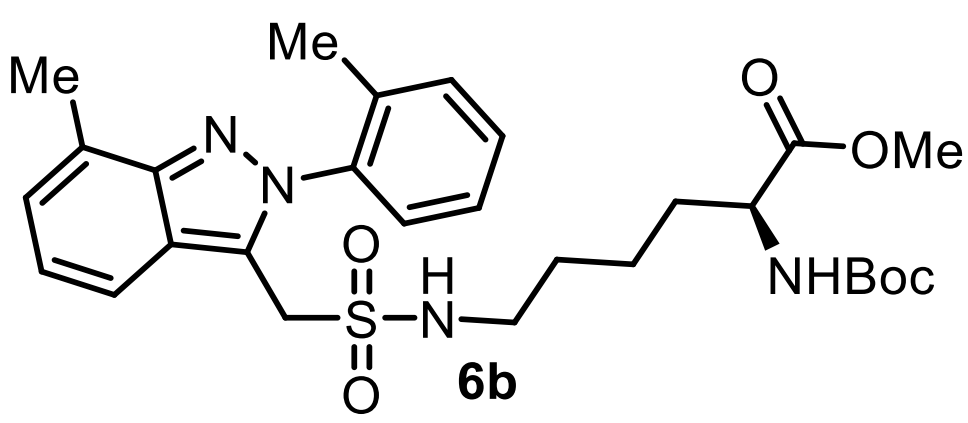

6b

10.6 mg (19%); White solid; ^{1}H NMR (600 MHz, $CDCl_3$) δ 7.62 (dt, *J* = 6.7, 3.0 Hz, 1H), 7.47 – 7.43 (m, 2H), 7.37 (dd, *J* = 15.6, 8.0 Hz, 2H), 7.13 - 7.10 (m, 2H), 5.04 (d, *J* = 8.3 Hz, 1H),

4.60 (s, 1H), 4.44 (t, J = 5.9 Hz, 2H), 4.24 (q, J = 7.7 Hz, 1H), 3.72 (s, 3H), 2.80 (q, J = 6.7 Hz, 2H), 2.64 (s, 3H), 2.00 (s, 3H), 1.74 – 1.68 (m, 2H), 1.54 (dq, J = 13.9, 7.6, 6.3 Hz, 2H), 1.42 (s, 9H), 1.33 – 1.27 (m, 2H) ppm; ^{13}C NMR (100 MHz, $CDCl_3$) δ 173.21, 155.61, 149.14, 138.04, 136.29, 131.45, 130.29, 128.56, 128.30, 126.83, 126.00, 124.79, 123.48, 121.74, 117.44, 80.23, 53.13, 52.48, 49.48, 43.43, 32.50, 29.81, 28.44, 22.24, 17.40, 17.19 ppm; FT-IR ($CHCl_3$, film) υ 2954, 2922, 2853, 1737, 1699, 1499, 1458, 1365, 1331, 1243, 1154, 1088, 1015, 865, 753, 722, 674 cm^{-1}; HRMS (EI+) calcd for $C_{28}H_{38}N_4O_6S$ $[M]^+$ 558.2512, found 558.2509.

Methyl (*S*)-2-((tert-butoxycarbonyl)amino)-3-(4-((((7-methyl-2-(*o*-tolyl)-2*H*-indazol-3-yl)methyl)sulfonyl)oxy)phenyl)propanoate (6c)

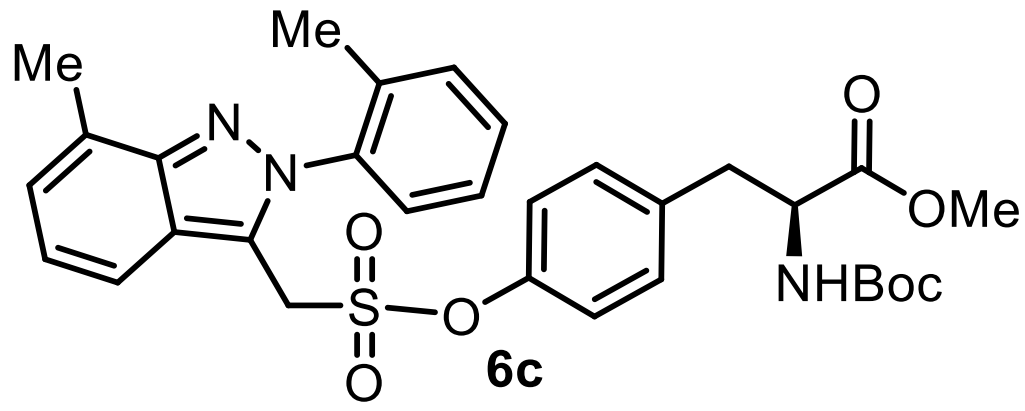

6c

85.4 mg (72%); White solid; ^{1}H NMR (600 MHz, $CDCl_3$) δ 7.66 – 7.65 (m, 1H), 7.46 (t, J = 7.6 Hz, 1H), 7.39 (q, J = 7.4 Hz, 2H), 7.34 (t, J = 7.0 Hz, 1H), 7.14 (d, J = 7.5 Hz, 2H), 7.07 (d, J = 8.5 Hz, 2H), 6.92 (d, J = 8.6 Hz, 2H), 5.00 – 4.74 (m, 2H), 4.55 (d, J = 7.8 Hz, 1H), 3.70 (s, 3H), 3.10 (dd, J = 14.0, 5.9 Hz, 1H), 3.00 (dd, J = 14.0, 6.5 Hz, 1H), 2.67 (s, 3H), 2.00 (s, 3H), 1.66 (s, 1H), 1.41 (s, 9H) ppm; ^{13}C NMR (150 MHz, $CDCl_3$) δ 172.10, 155.12, 149.21, 147.70, 137.63, 136.55, 135.65, 131.55, 130.92, 130.45, 128.60, 128.15, 126.88, 126.16, 123.90, 122.30, 121.97, 121.92, 117.43, 80.27, 54.37, 52.46, 47.88, 37.82, 28.40, 17.30, 17.19 ppm; FT-IR ($CHCl_3$, film) υ 3381, 2972, 2925, 2857, 1741, 1704, 1502, 1436, 1363, 1247, 1197, 1142, 1017, 862, 753 cm^{-1}; HRMS (EI+) calcd for $C_{31}H_{35}N_3O_7S$ $[M]^+$ 593.2196, found 593.2191.

***N*-(2-(2,6-Dioxopiperidin-3-yl)-1-oxoisoindolin-4-yl)-1-(7-methyl-2-(o-tolyl)-2H-indazol-3-yl)methanesulfonamide (6d)**

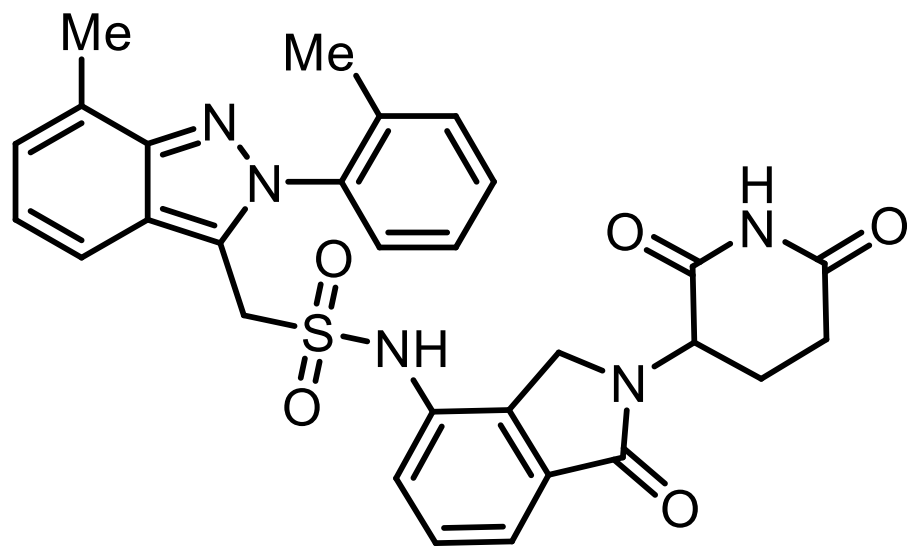

6d

30.0 mg (54%); White solid; ^{1}H NMR (400 MHz, DMSO-d_6) δ 11.01 (s, 1H), 7.72 (d, *J* = 8.4 Hz, 1H), 7.50 – 7.42 (m, 3H), 7.37 (td, *J* = 7.5, 1.9 Hz, 1H), 7.15 (t, *J* = 7.7 Hz, 1H), 7.06 (dt, *J* = 6.7, 1.1 Hz, 1H), 6.97 (dd, *J* = 8.5, 6.6 Hz, 1H), 6.92 (d, *J* = 6.8 Hz, 1H), 6.53 (d, *J* = 7.3 Hz, 1H), 6.12 (t, *J* = 5.3 Hz, 1H), 5.09 (dd, *J* = 13.2, 5.1 Hz, 1H), 4.60 (d, *J* = 5.6 Hz, 2H), 4.09 (d, *J* = 17.3 Hz, 1H), 4.01 (d, *J* = 17.2 Hz, 1H), 2.92 (ddd, *J* = 17.3, 13.5, 5.4 Hz, 1H), 2.62 (d, *J* = 17.3 Hz, 1H), 2.49 (s, 3H), 2.25 (qd, *J* = 13.2, 4.5 Hz, 1H), 2.07 – 2.00 (m, 1H), 1.93 (s, 3H) ppm; ^{13}C NMR (100 MHz, DMSO-D_6) δ 172.91, 171.21, 168.67, 148.23, 142.66, 138.69, 135.10, 133.71, 132.13, 130.90, 129.65, 129.07, 127.28, 126.83, 126.80, 126.53, 125.06, 121.39, 120.22, 118.12, 111.87, 110.80, 51.50, 45.62, 37.89, 31.24, 22.82, 16.82, 16.71 ppm; FT-IR ($CHCl_3$, film) υ 3384, 3194, 3084, 2919, 2852, 1729, 1707, 1671, 1612, 1526, 1493, 1458, 1421, 1375, 1348, 1328, 1233, 1190, 1148, 1006, 980, 916, 815, 790, 769, 747 cm^{-1}; HRMS (FAB-) calcd for $C_{29}H_{26}N_5O_5S$ [M-H]$^-$ 556.1654, found 556.1662.

(8*R*,9*S*,13*S*,14*S*)-13-methyl-17-oxo-7,8,9,11,12,13,14,15,16,17-decahydro-6*H*-cyclopenta[a]phenanthren-3-yl (7-methyl-2-(*o*-tolyl)-2*H*-indazol-3-yl)methanesulfonate (6e)

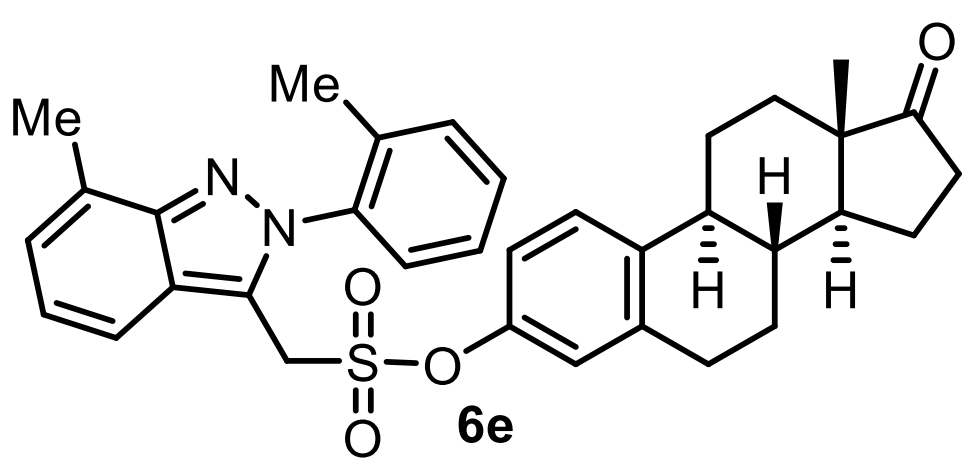

6e

63.7 mg (56%); White solid; ^{1}H NMR (400 MHz, $CDCl_3$) δ 7.69 – 7.65 (m, 1H), 7.45 (td, *J* = 7.4, 1.5 Hz, 1H), 7.43 – 7.36 (m, 2H), 7.36 – 7.30 (m, 1H), 7.20 (d, *J* = 8.7 Hz, 1H), 7.17 – 7.11 (m, 2H), 6.75 (dd, *J* = 8.6, 2.7 Hz, 1H), 6.68 (d, *J* = 2.6 Hz, 1H), 4.83 (d, *J* = 41.0 Hz, 2H),

2.81 (dd, J = 9.8, 5.2 Hz, 2H), 2.67 (s, 3H), 2.55 – 2.45 (m, 1H), 2.40 – 2.32 (m, 1H), 2.24 (td, J = 10.8, 7.1 Hz, 1H), 2.19 – 2.06 (m, 1H), 2.01 (s, 3H), 1.97 (s, 1H), 1.71 – 1.36 (m, 8H), 0.90 (s, 3H) ppm; ^{13}C NMR (150 MHz, $CDCl_3$) δ 149.16, 146.62, 139.15, 138.83, 137.64, 136.53, 131.47, 130.35, 128.48, 128.13, 126.93, 126.80, 126.10, 123.77, 122.48, 121.95, 121.78, 118.97, 117.52, 50.44, 47.94, 47.79, 44.16, 37.91, 35.89, 31.57, 29.36, 26.22, 25.77, 21.64, 17.28, 17.16, 13.88 ppm; FT-IR ($CHCl_3$, film) υ 2921, 2850, 1732, 1489, 1370, 1171, 1141, 916, 819, 774, 722 cm^{-1}; HRMS (EI+) calcd for $C_{34}H_{36}N_2O_4S$ $[M]^+$ 568.2396, found 568.2395.

4-Oxo-2-phenyl-4*H*-chromen-7-yl (7-methyl-2-(*o*-tolyl)-2*H*-indazol-3-yl)methanesulfonate (6f)

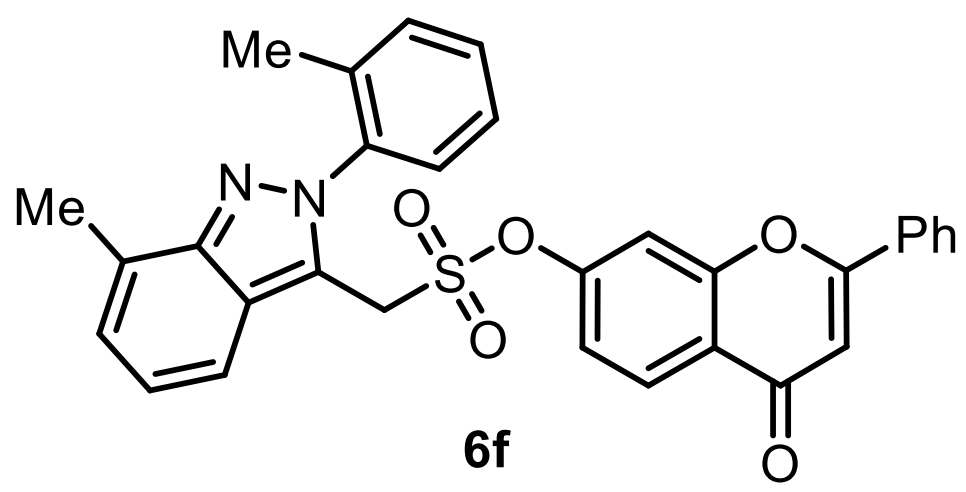

6f

41.5 mg (77%); Yellow solid; ^{1}H NMR (600 MHz, $CDCl_3$) δ 8.16 (d, J = 8.7 Hz, 1H), 7.87 (d, J = 6.8 Hz, 2H), 7.69 – 7.67 (m, 1H), 7.54 (dd, J = 11.6, 7.2 Hz, 4H), 7.45 – 7.37 (m, 3H), 7.33 (t, J = 7.6 Hz, 1H), 7.19 (dd, J = 18.6, 4.0 Hz, 3H), 6.99 (dd, J = 8.8, 2.3 Hz, 1H), 6.80 (s, 1H), 4.96 (d, J = 99.4 Hz, 2H), 2.68 (s, 3H), 2.02 (s, 3H) ppm; ^{13}C NMR (150 MHz, $CDCl_3$) δ 177.27, 163.97, 156.53, 152.09, 149.22, 137.51, 136.49, 132.07, 131.61, 131.26, 130.53, 129.25, 128.78, 128.03, 127.89, 126.90, 126.38, 126.25, 124.19, 122.84, 122.10, 121.66, 119.18, 117.14, 111.49, 107.80, 48.44, 17.31, 17.16 ppm; FT-IR ($CHCl_3$, film) υ 2922, 2850, 1734, 1489, 1377, 1177, 1147, 915, 819, 774, 722 cm^{-1}; HRMS (FAB+) calcd for $C_{31}H_{25}N_2O_5S$ $[M+H]^+$ 537.1484, found 537.1486.

8. ^{1}H NMR, ^{13}C NMR and ^{19}F NMR spectra of products

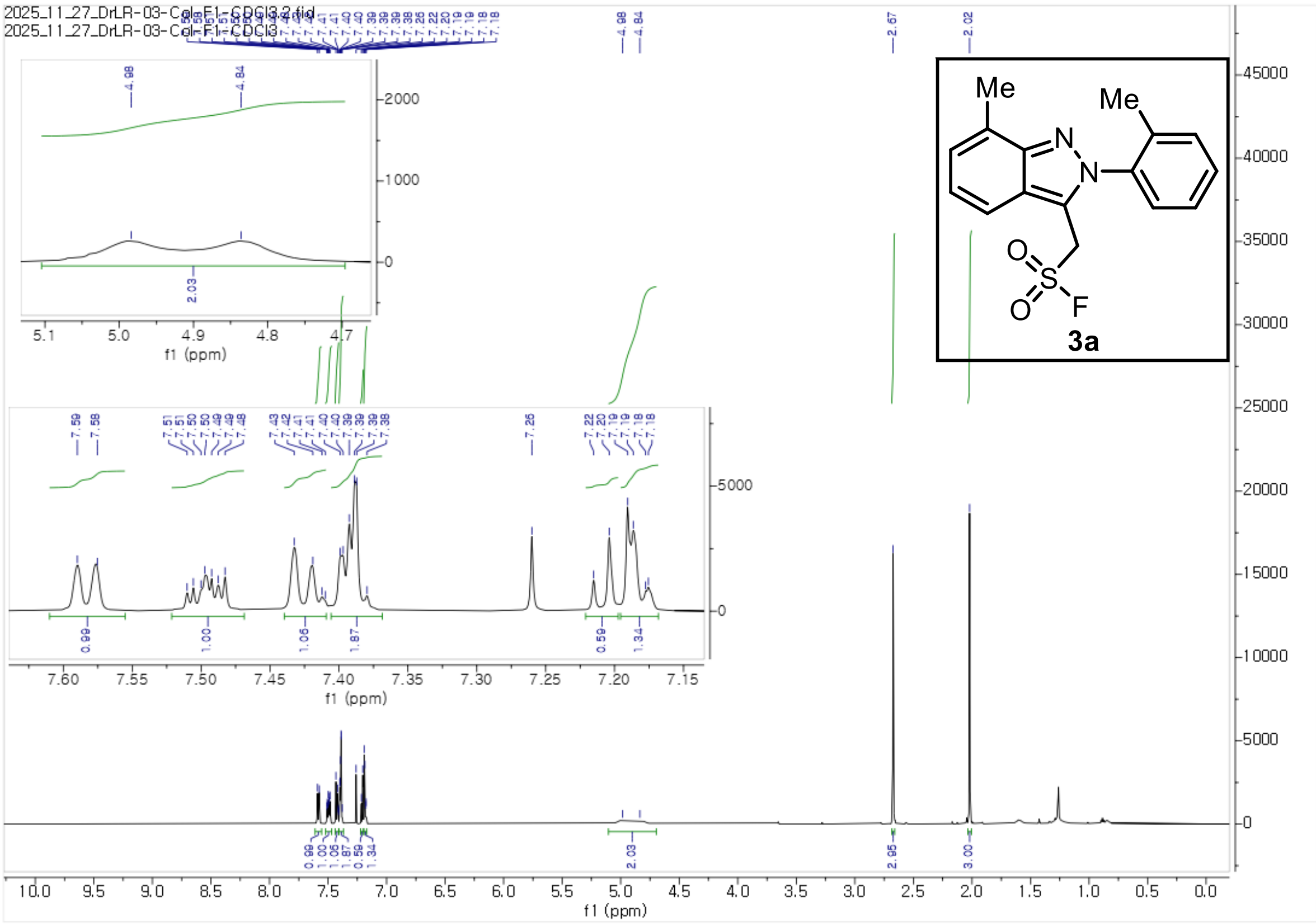

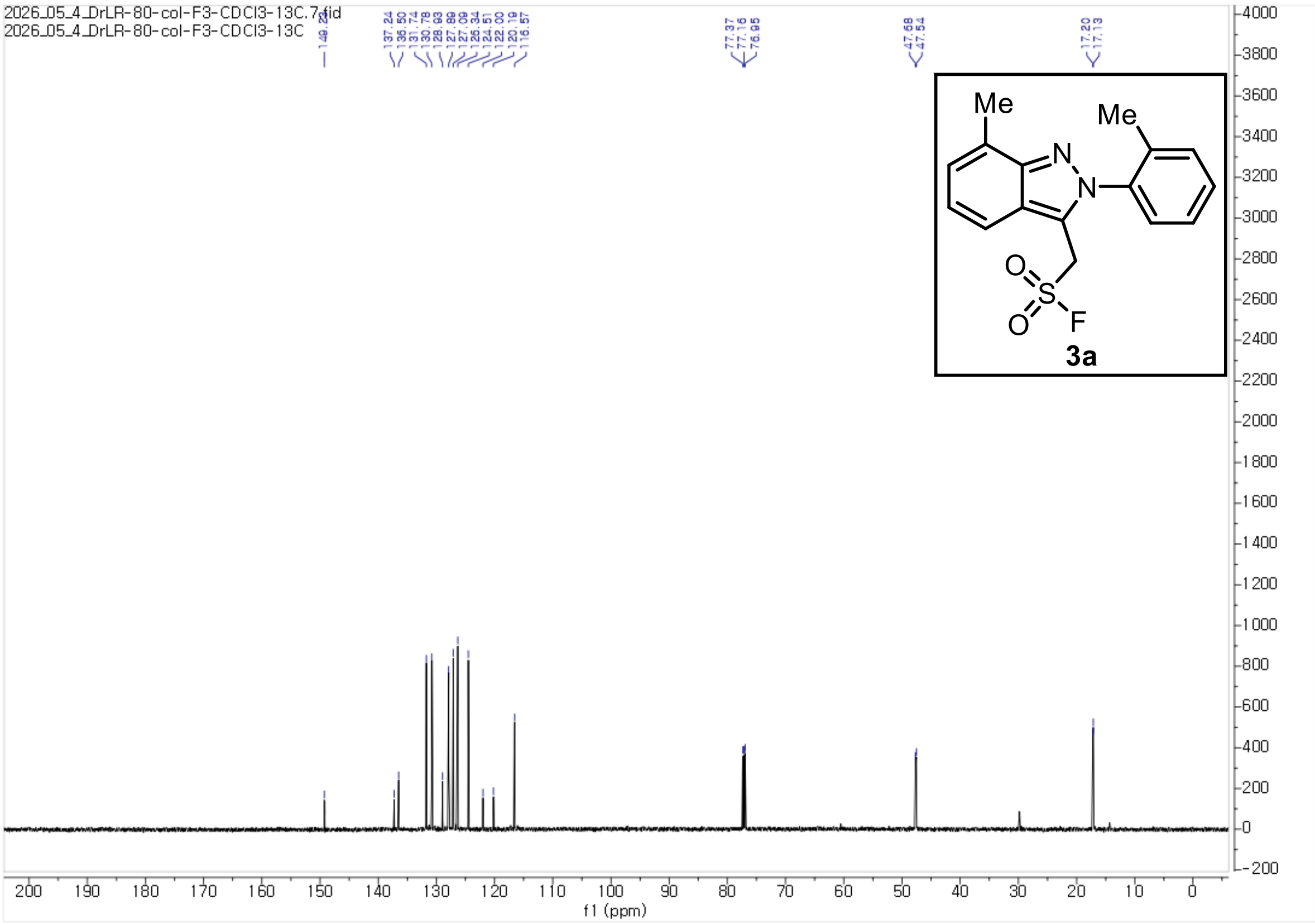

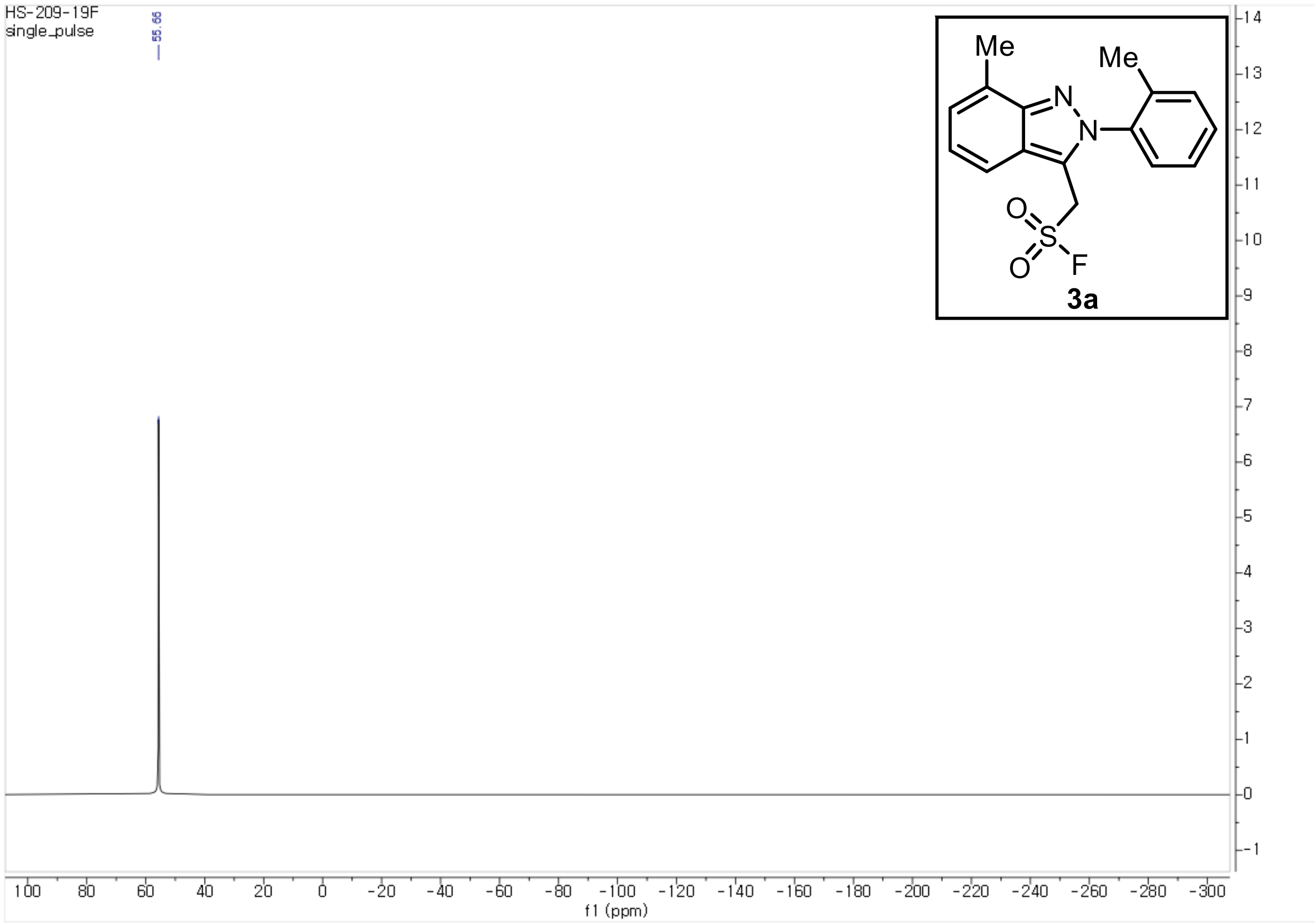

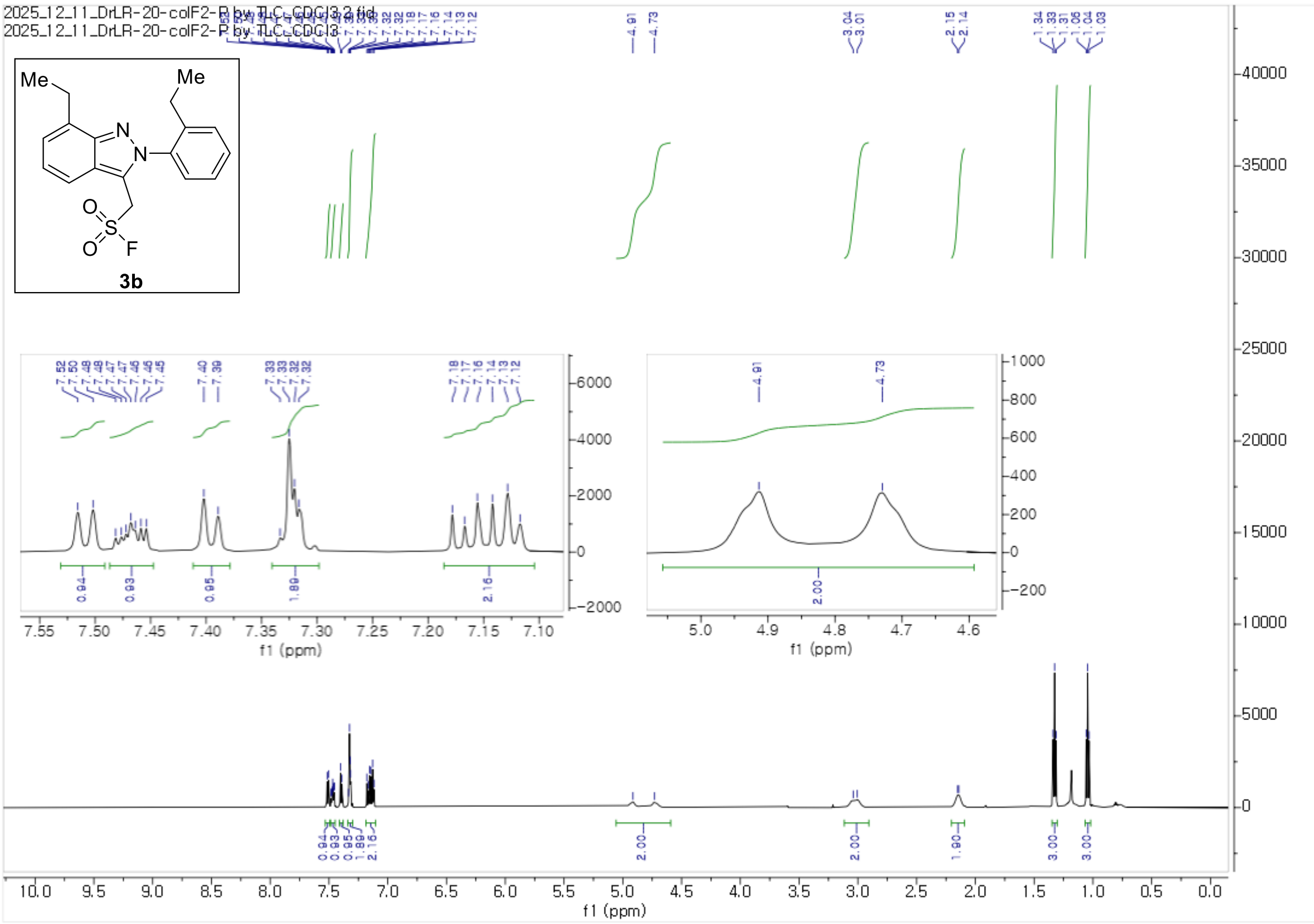

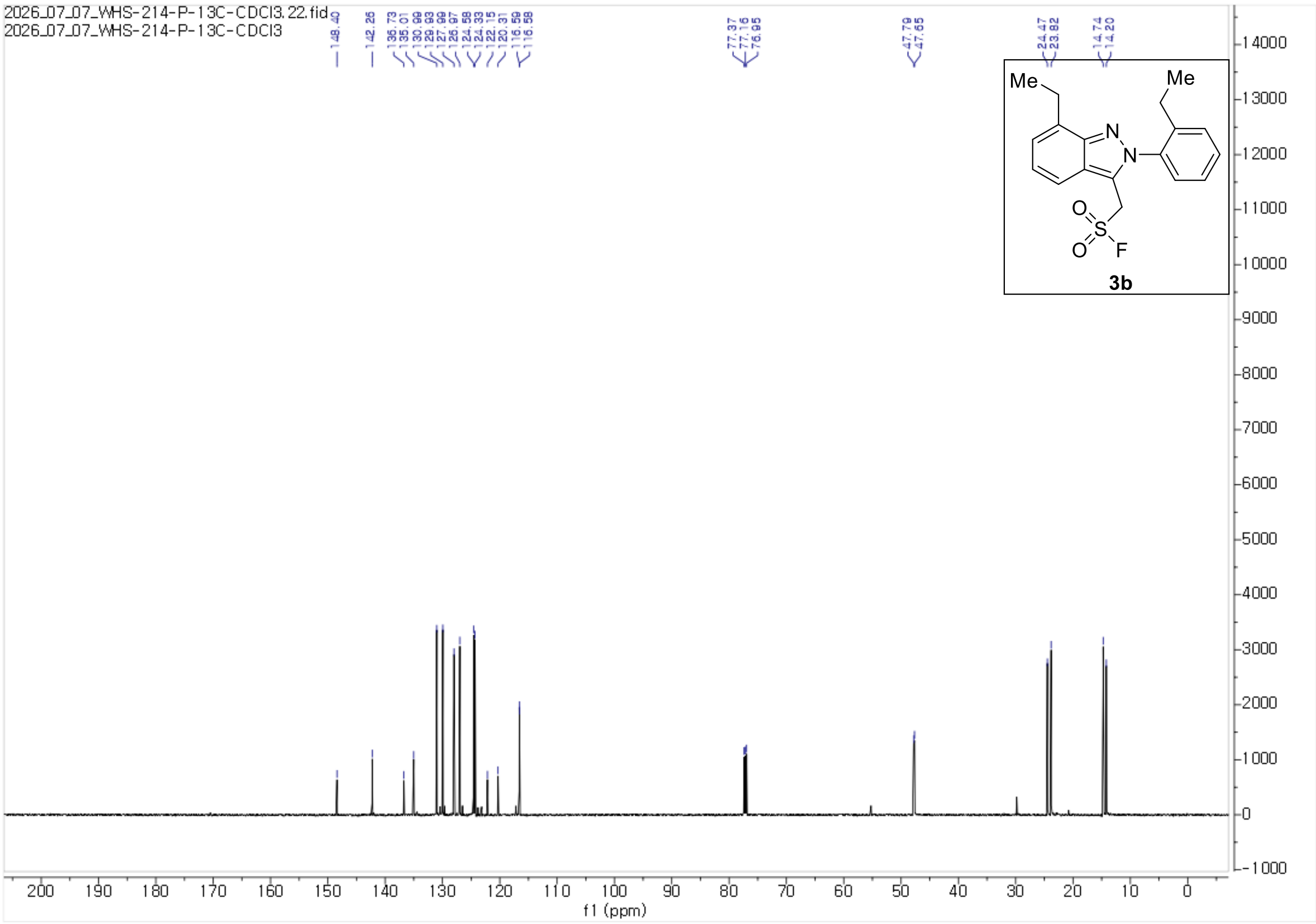

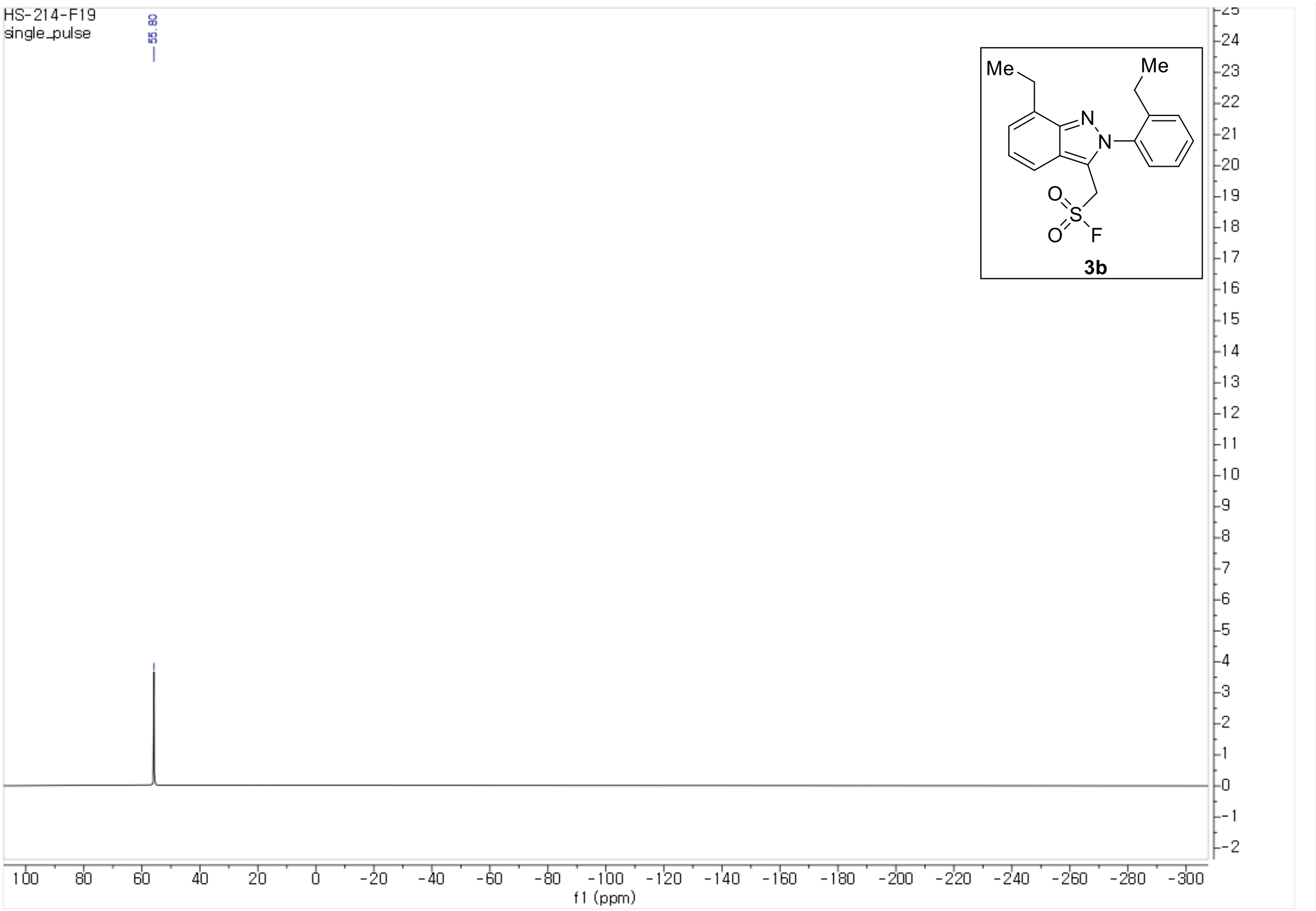

HS-214-F19
single_pulse
55.80
Me
Me
N
N
O
S
O
F
3b
f1 (ppm)

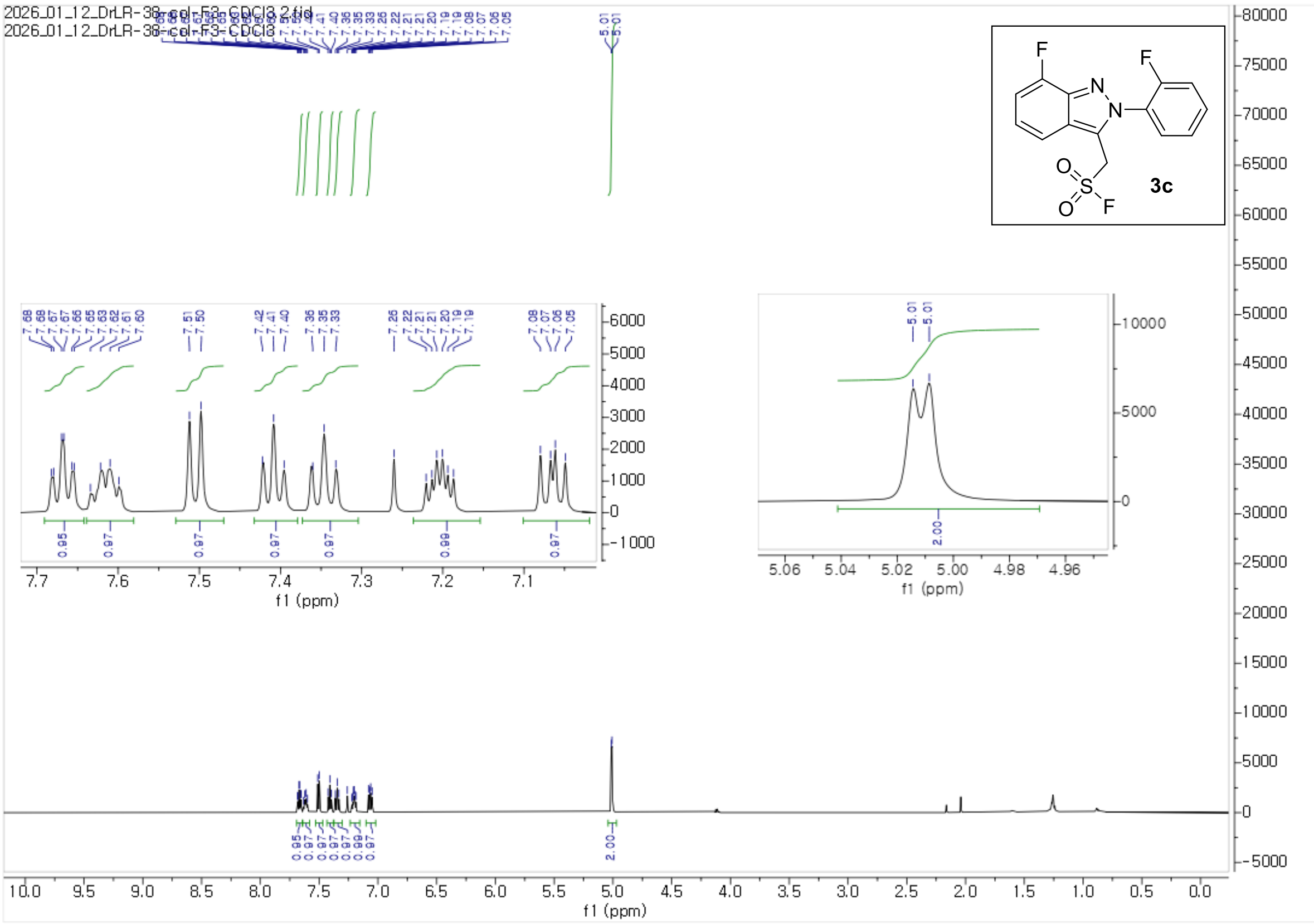
2026_01_12_DrLR-38-col-F3-CDCl3.2.fid
2026_01_12_DrLR-38-col-F3-CDCl3
3c
f1 (ppm)

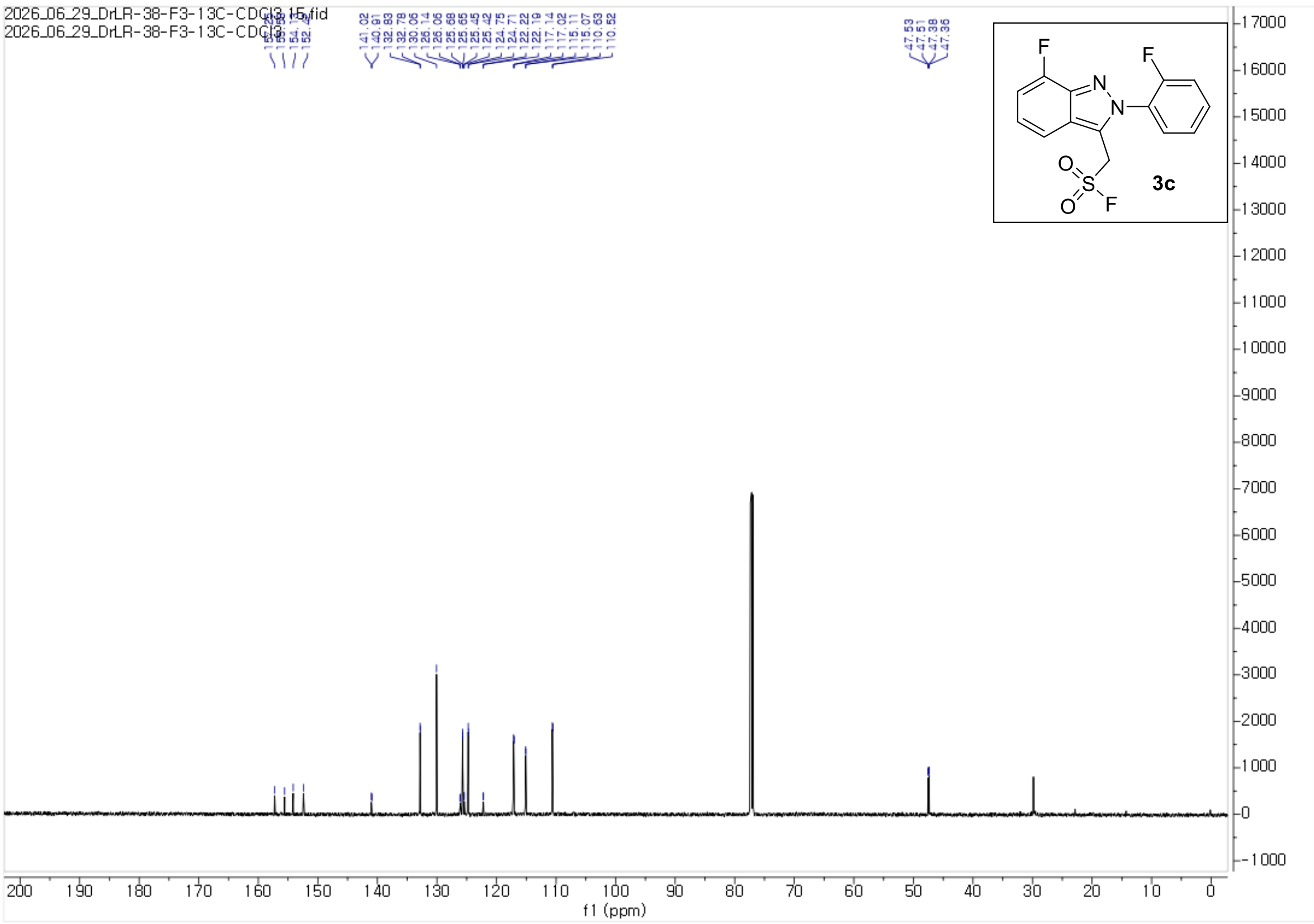
2026_06_29_DrLR-38-F3-13C-CDCl3.15.fid
2026_06_29_DrLR-38-F3-13C-CDCl3
3c
f1 (ppm)

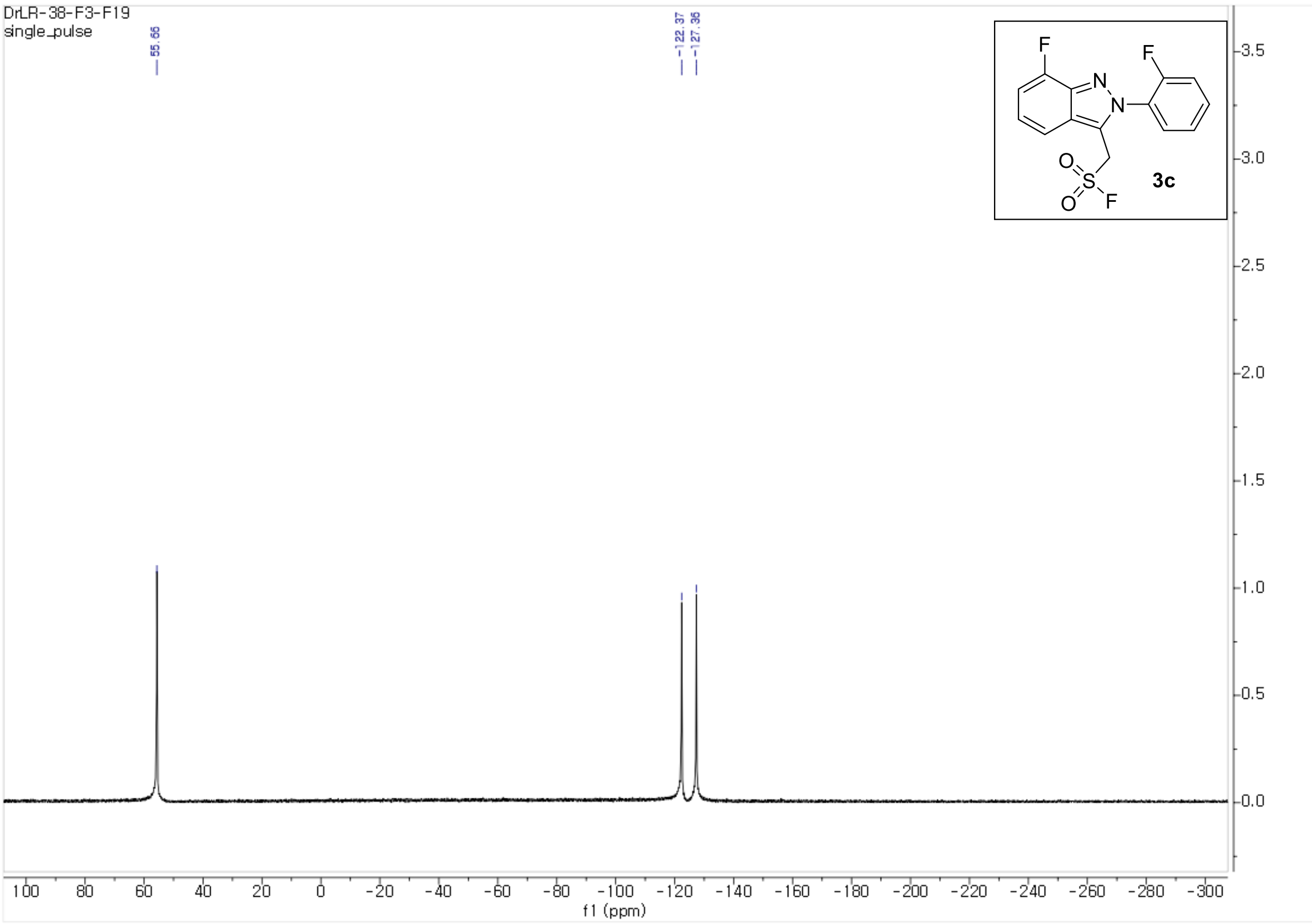
DrLR-38-F3-F19
single_pulse
55.66
-122.37
-127.36
3c
f1 (ppm)

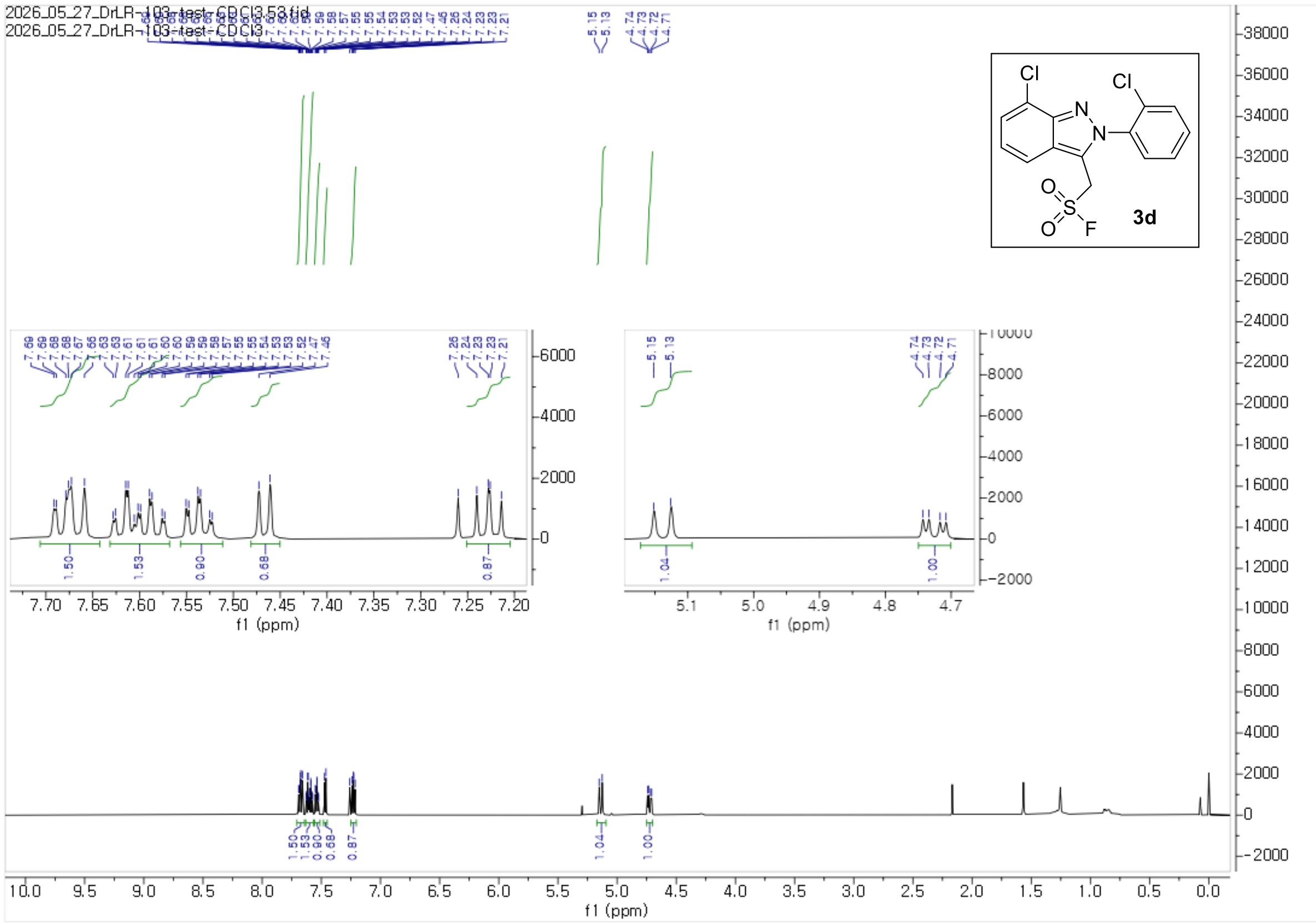
2026_05_27_DrLR-103-test-CDCl3.53.fid
2026_05_27_DrLR-103-test-CDCl3
3d
f1 (ppm)

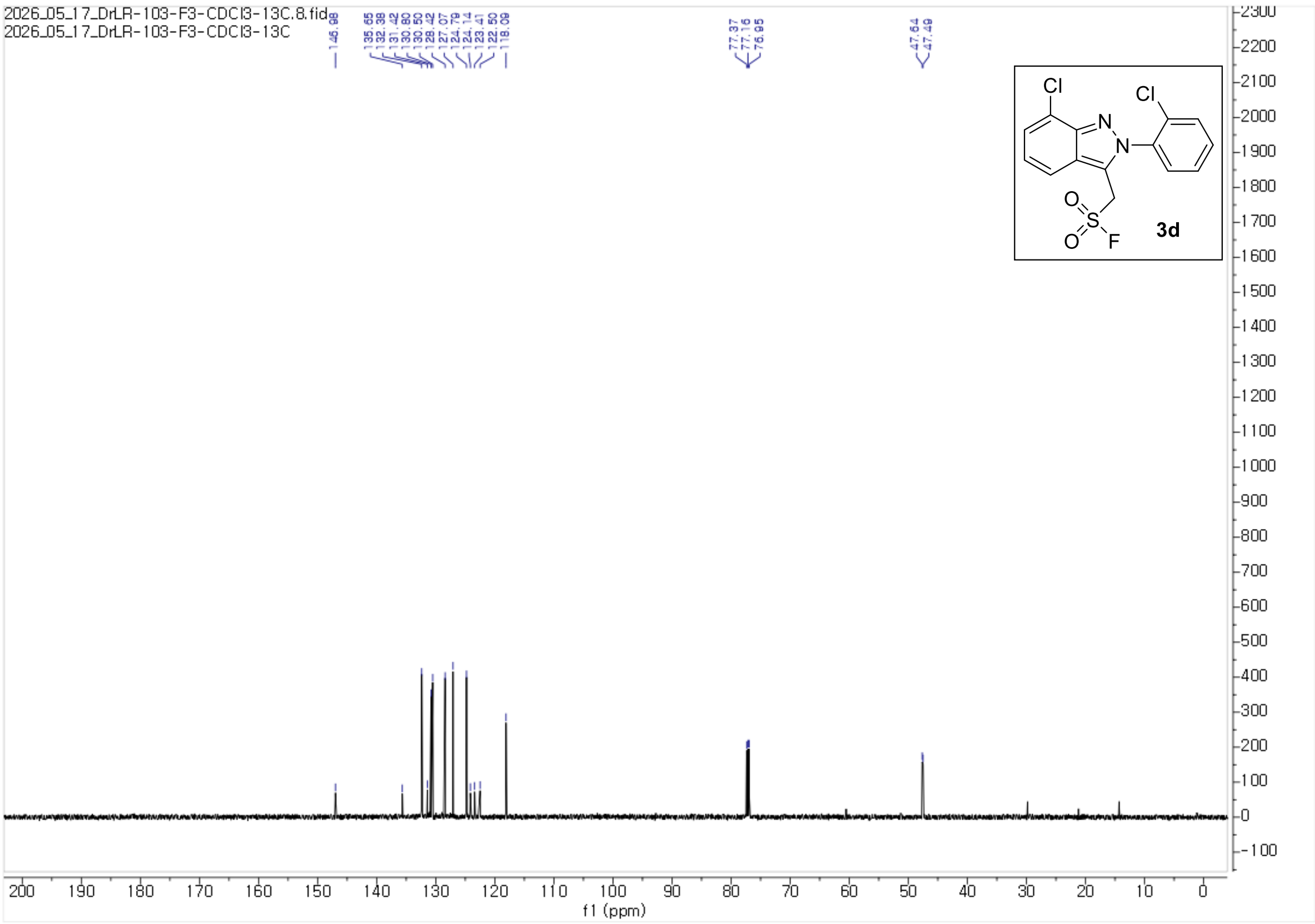
2026_05_17_DrLR-103-F3-CDCl3-13C.8.fid
2026_05_17_DrLR-103-F3-CDCl3-13C
3d
f1 (ppm)

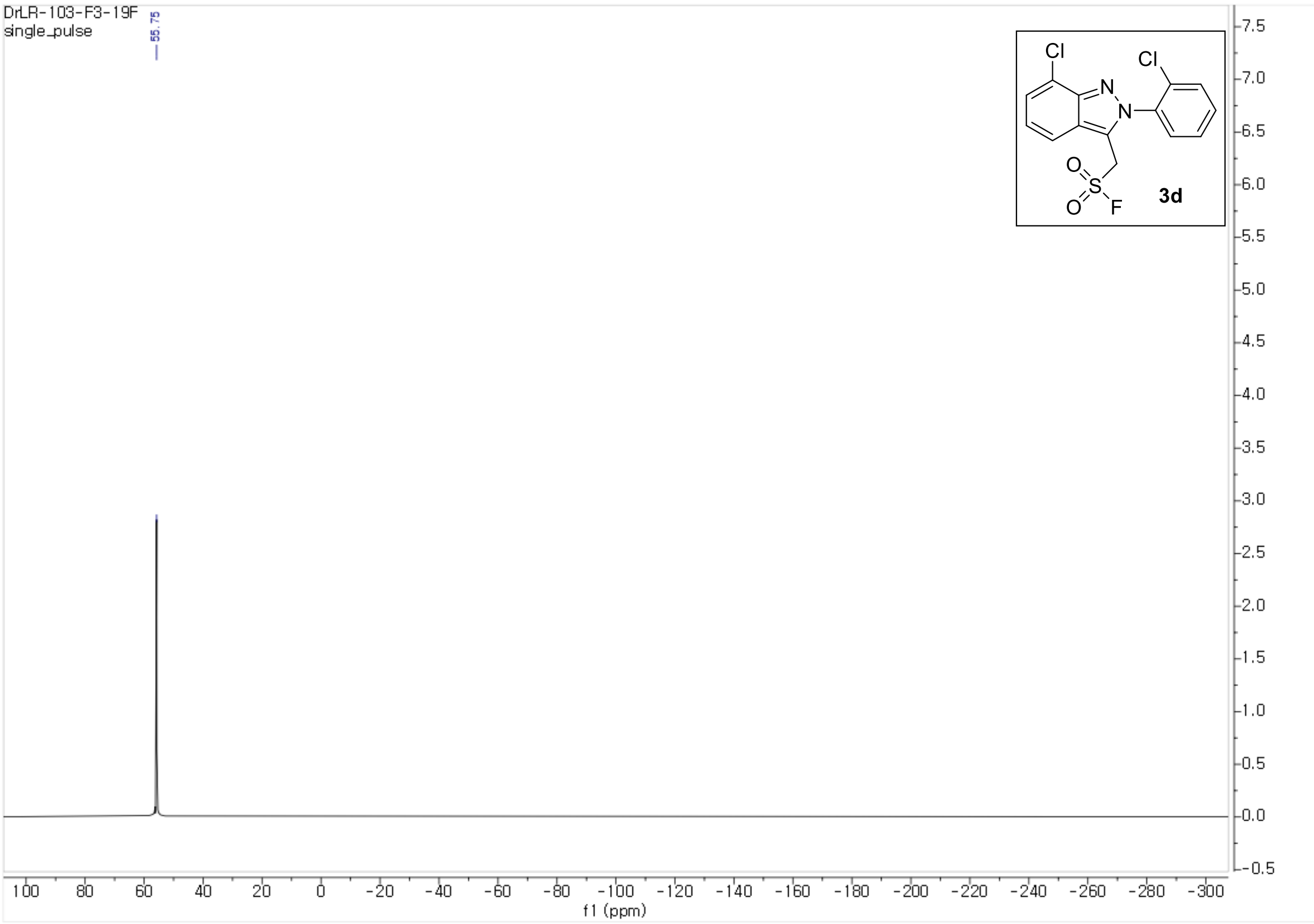

DrLR-103-F3-19F
single_pulse
55.75
3d
f1 (ppm)

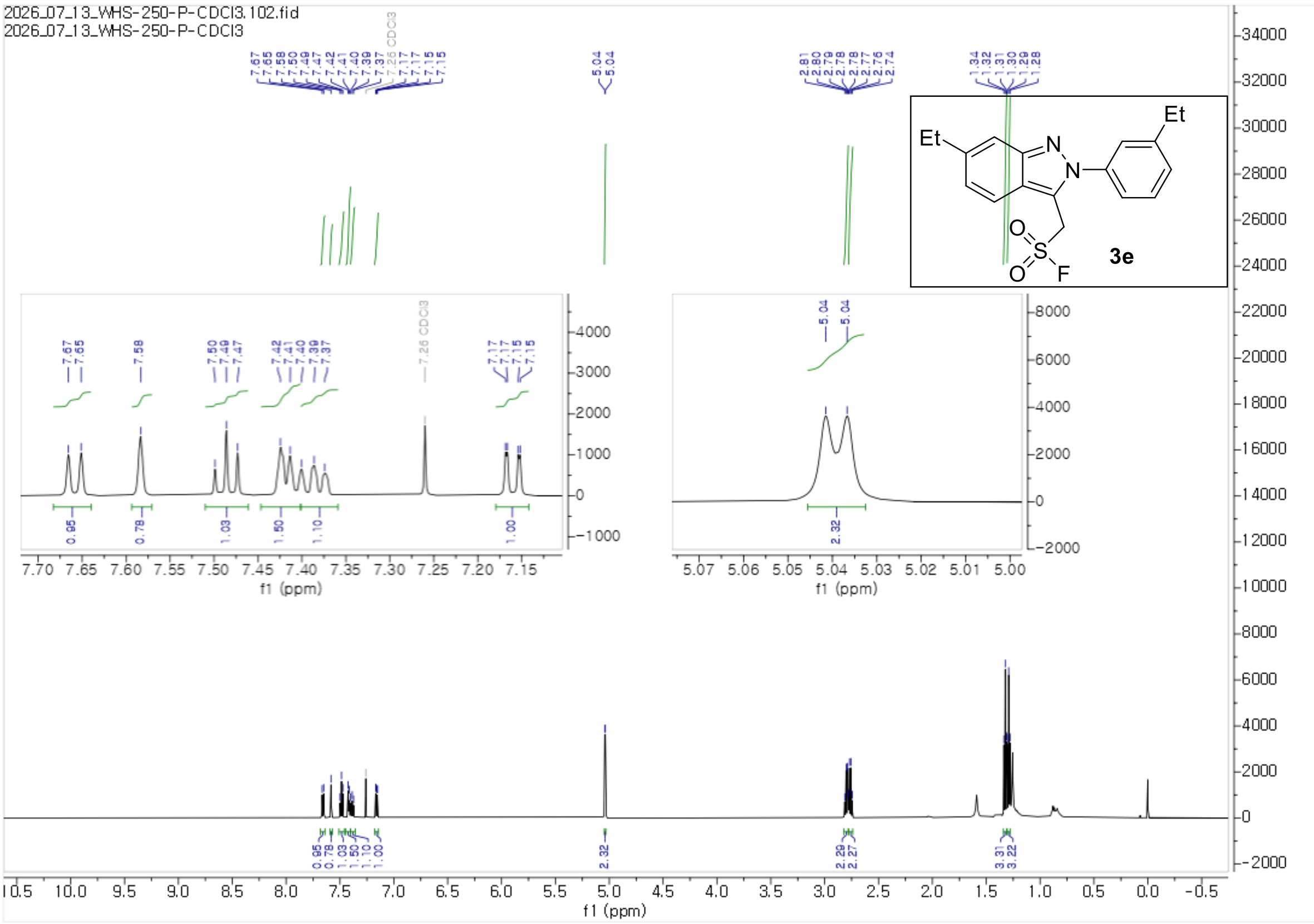
2026_07_13_WHS-250-P-CDCl3.102.fid
2026_07_13_WHS-250-P-CDCl3
Et
N
N
Et
O
S
O
F
3e
f1 (ppm)

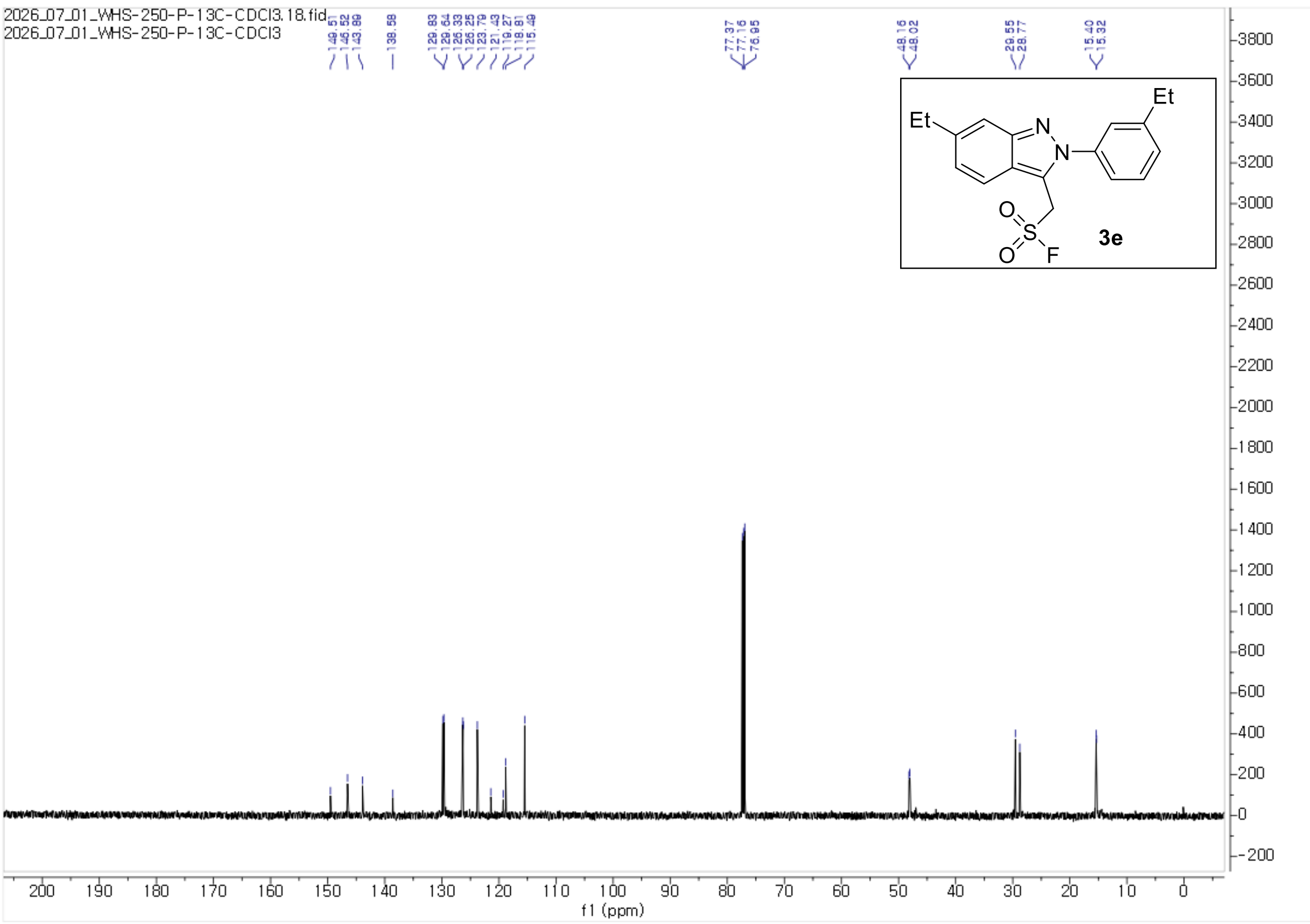
2026_07_01_WHS-250-P-13C-CDCl3.18.fid
2026_07_01_WHS-250-P-13C-CDCl3
Et
N
N
Et
O
S
O
F
3e
f1 (ppm)

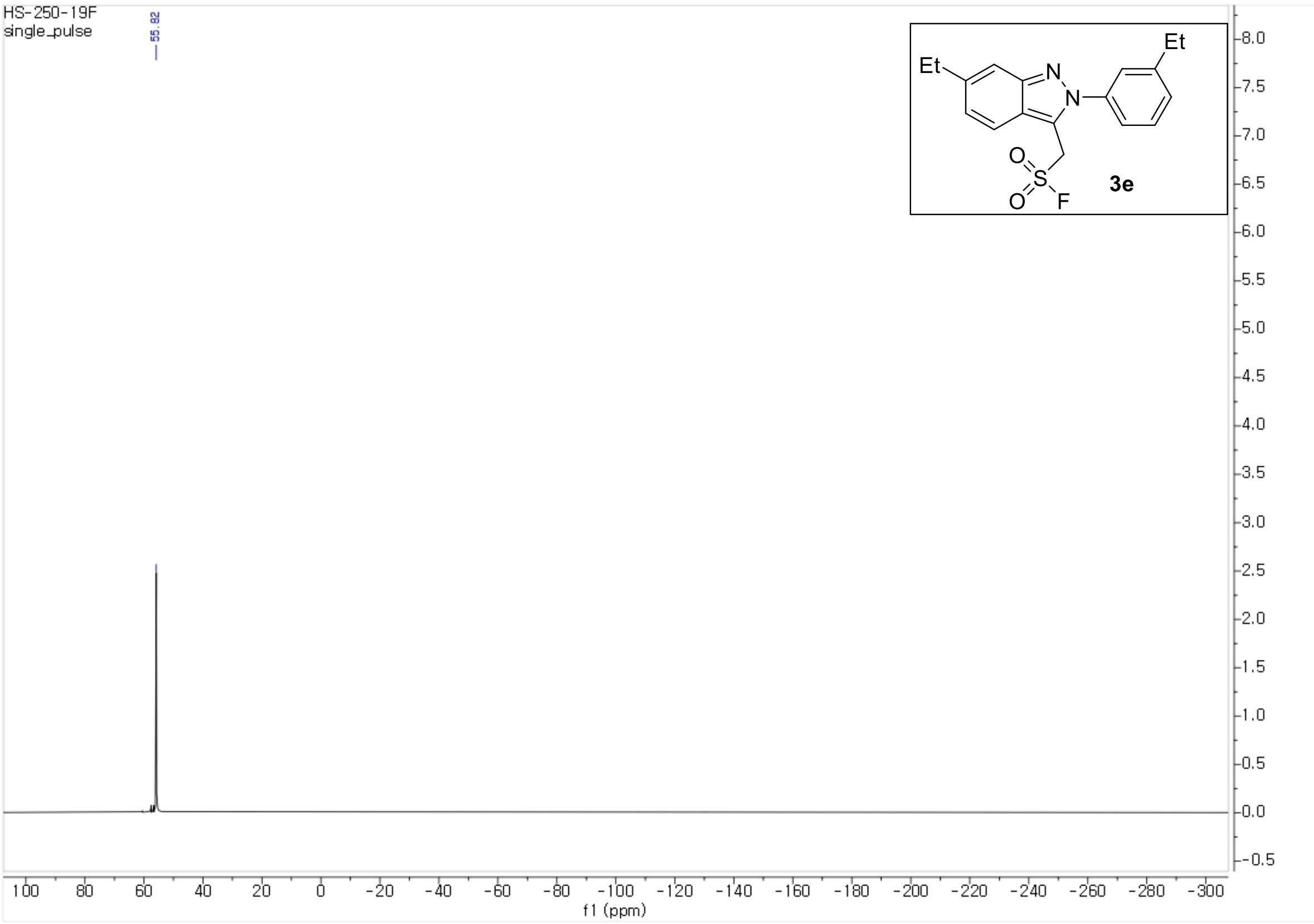
HS-250-19F
single_pulse
55.82
3e
f1 (ppm)

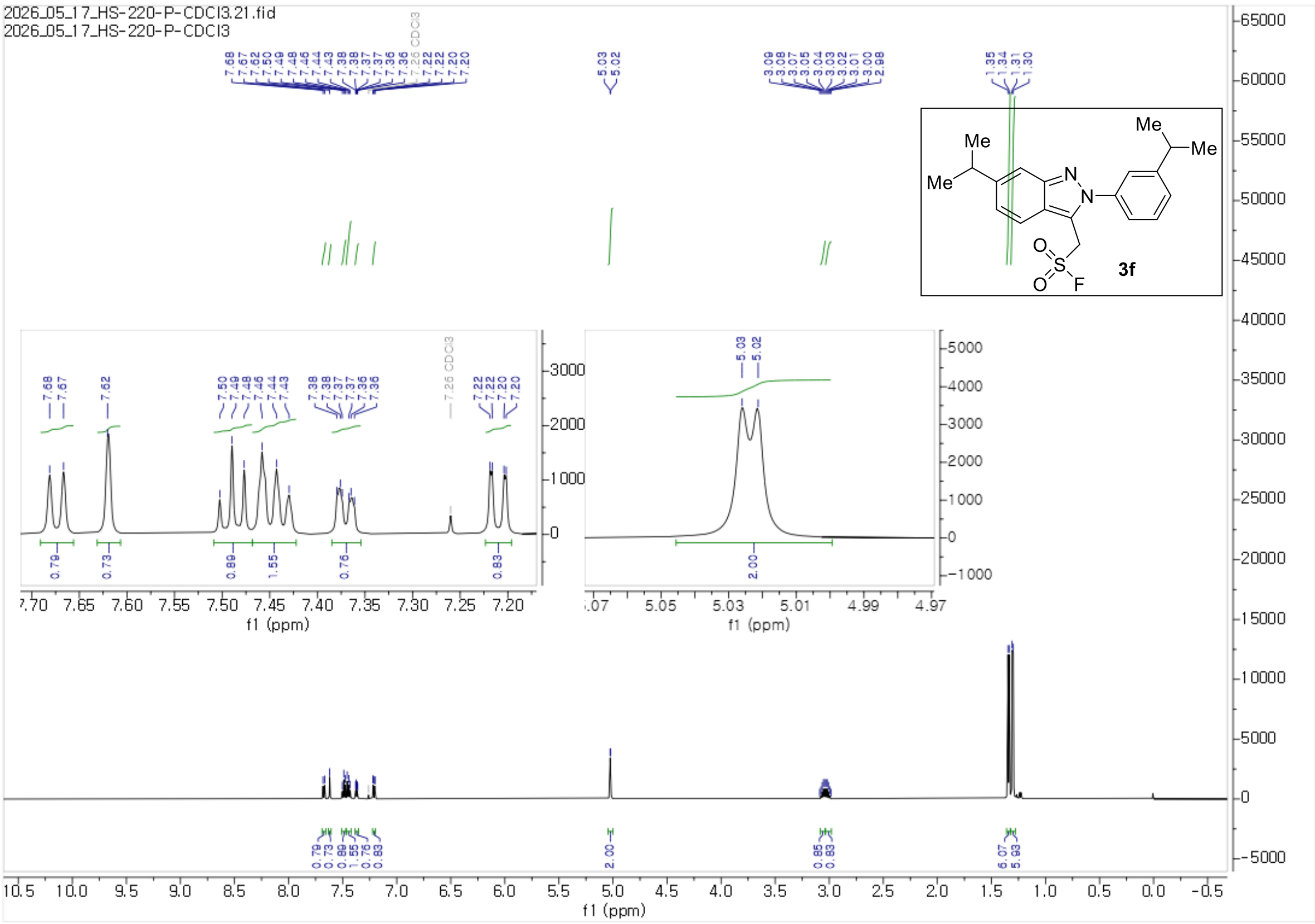
2026_05_17_HS-220-P-CDCl3.21.fid
2026_05_17_HS-220-P-CDCl3
3f
f1 (ppm)

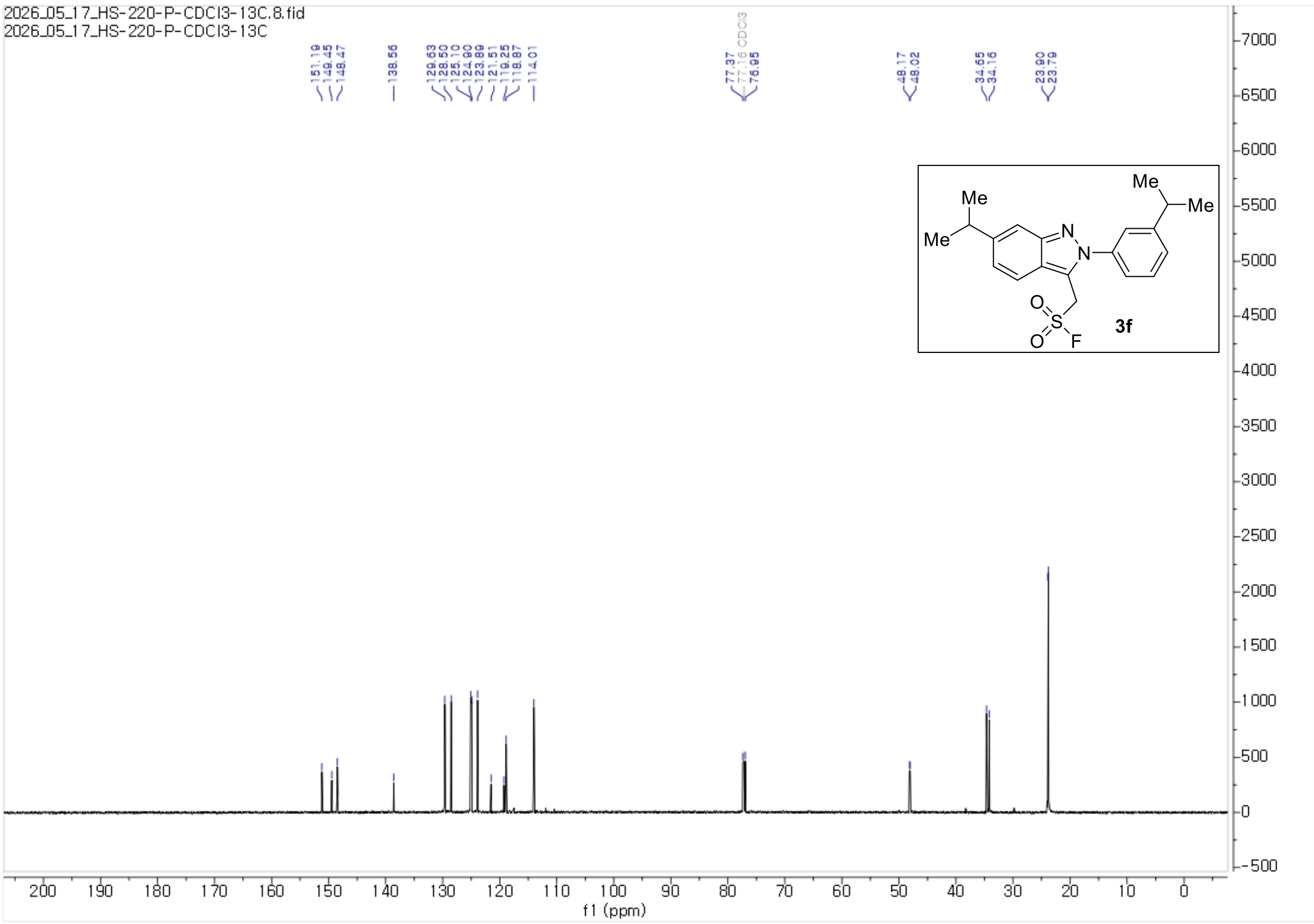
2026_05_17_HS-220-P-CDCl3-13C.8.fid
2026_05_17_HS-220-P-CDCl3-13C
3f
f1 (ppm)

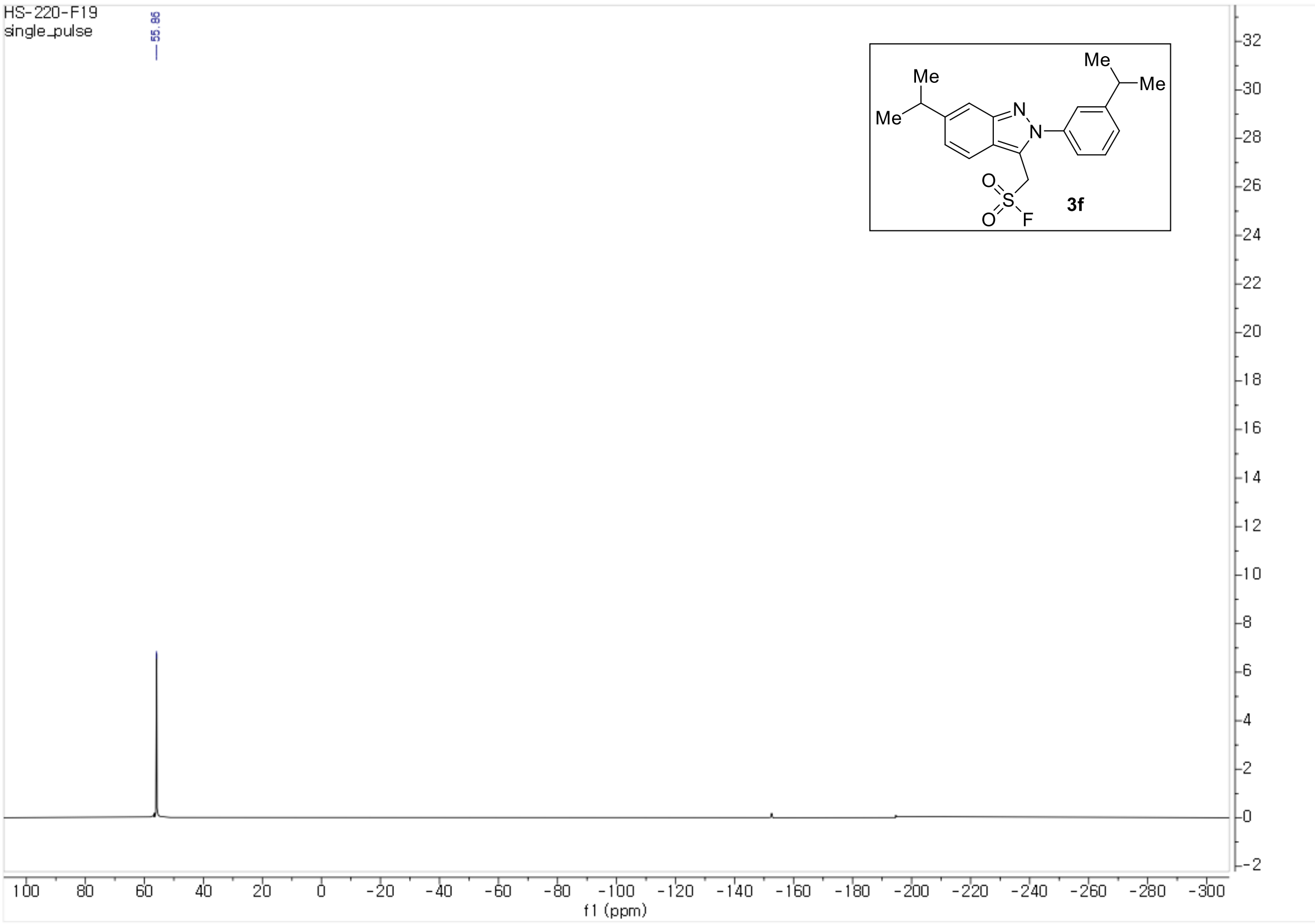
HS-220-F19
single_pulse
55.86
Me
Me
N
N
Me
Me
O
S
O
F
3f
f1 (ppm)

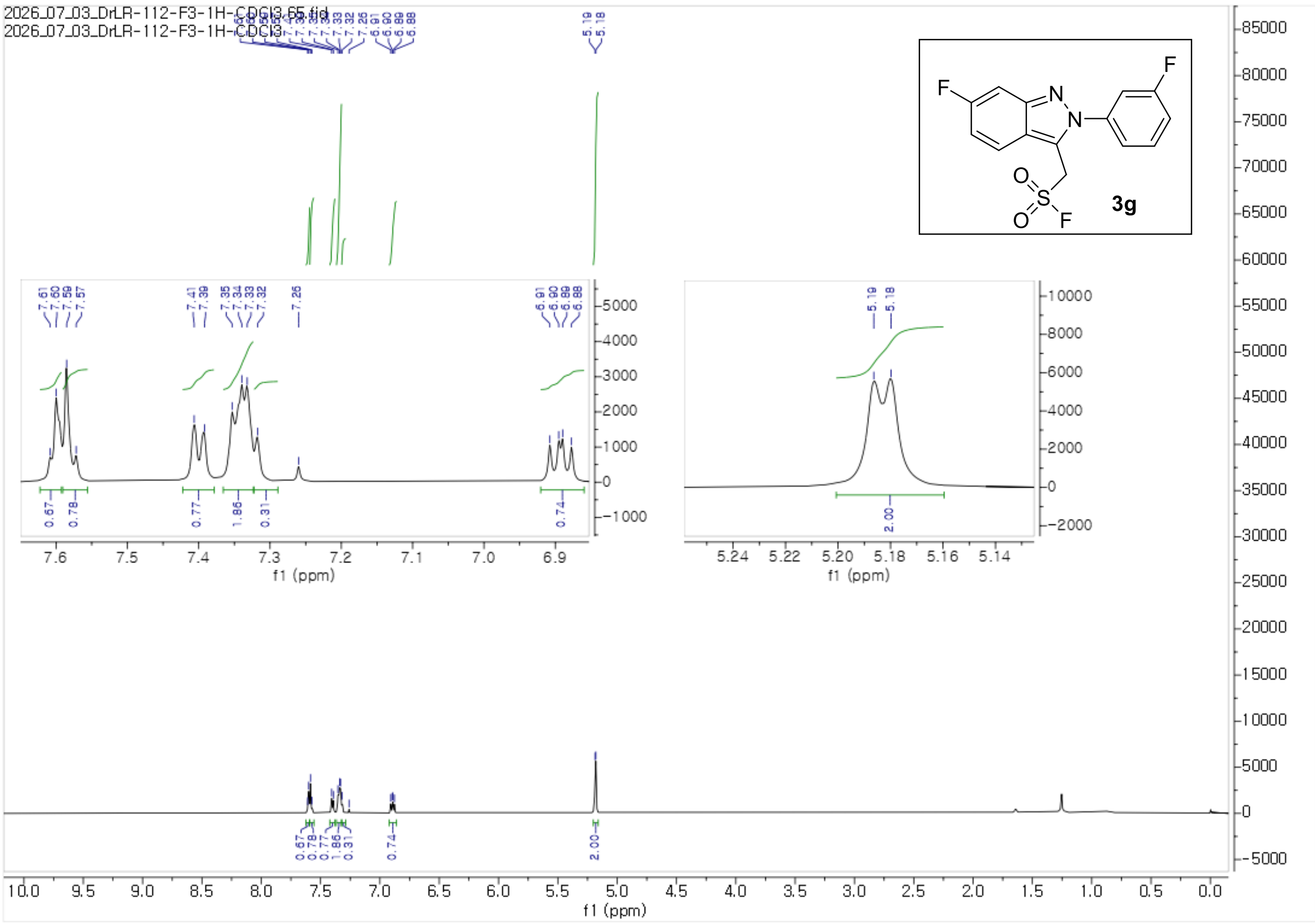
2026_07_03_DrLR-112-F3-1H-CDCl3.65.fid
2026_07_03_DrLR-112-F3-1H-CDCl3
3g
f1 (ppm)

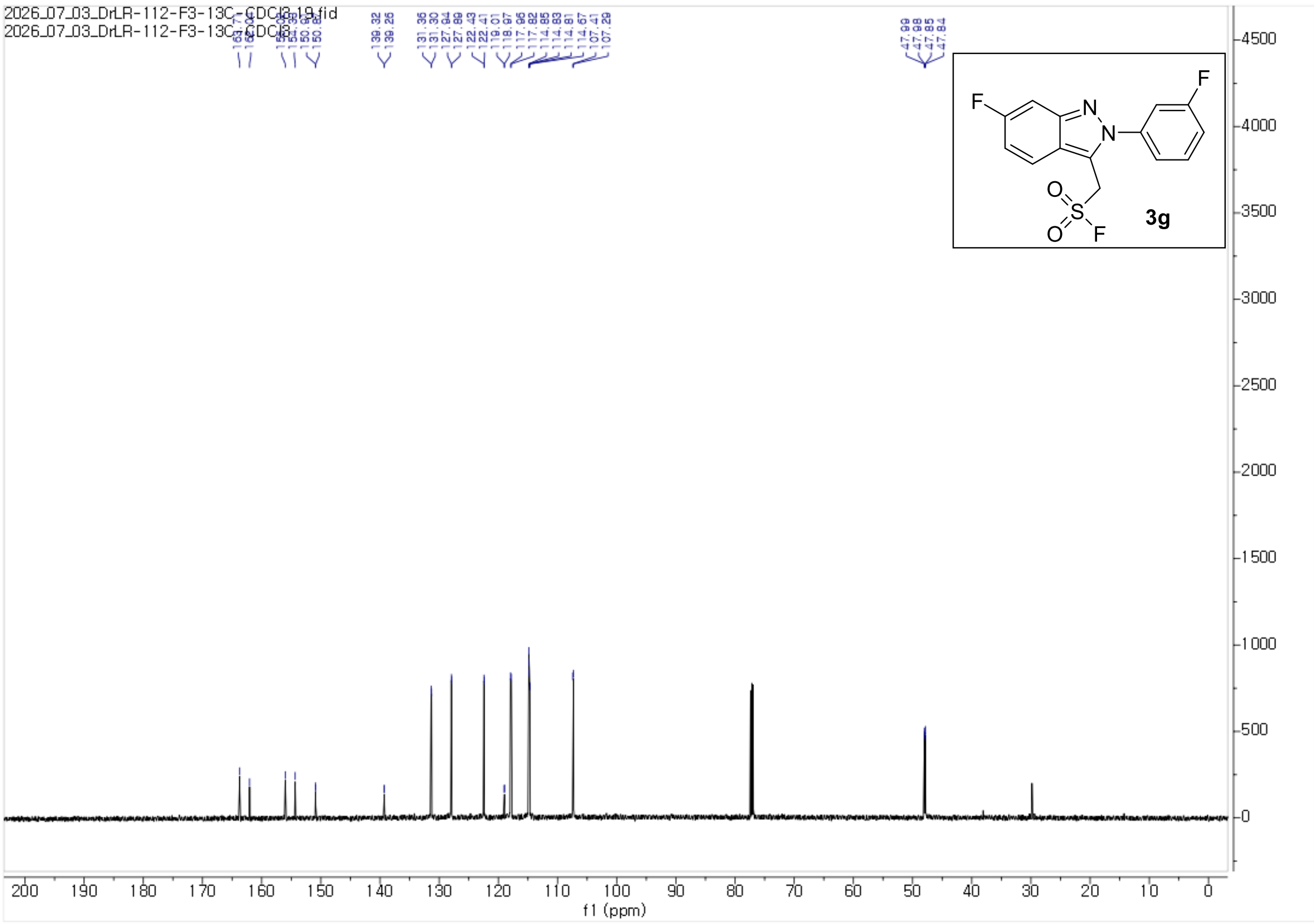
2026_07_03_DrLR-112-F3-13C-CDCl3.19.fid
2026_07_03_DrLR-112-F3-13C-CDCl3
3g
f1 (ppm)

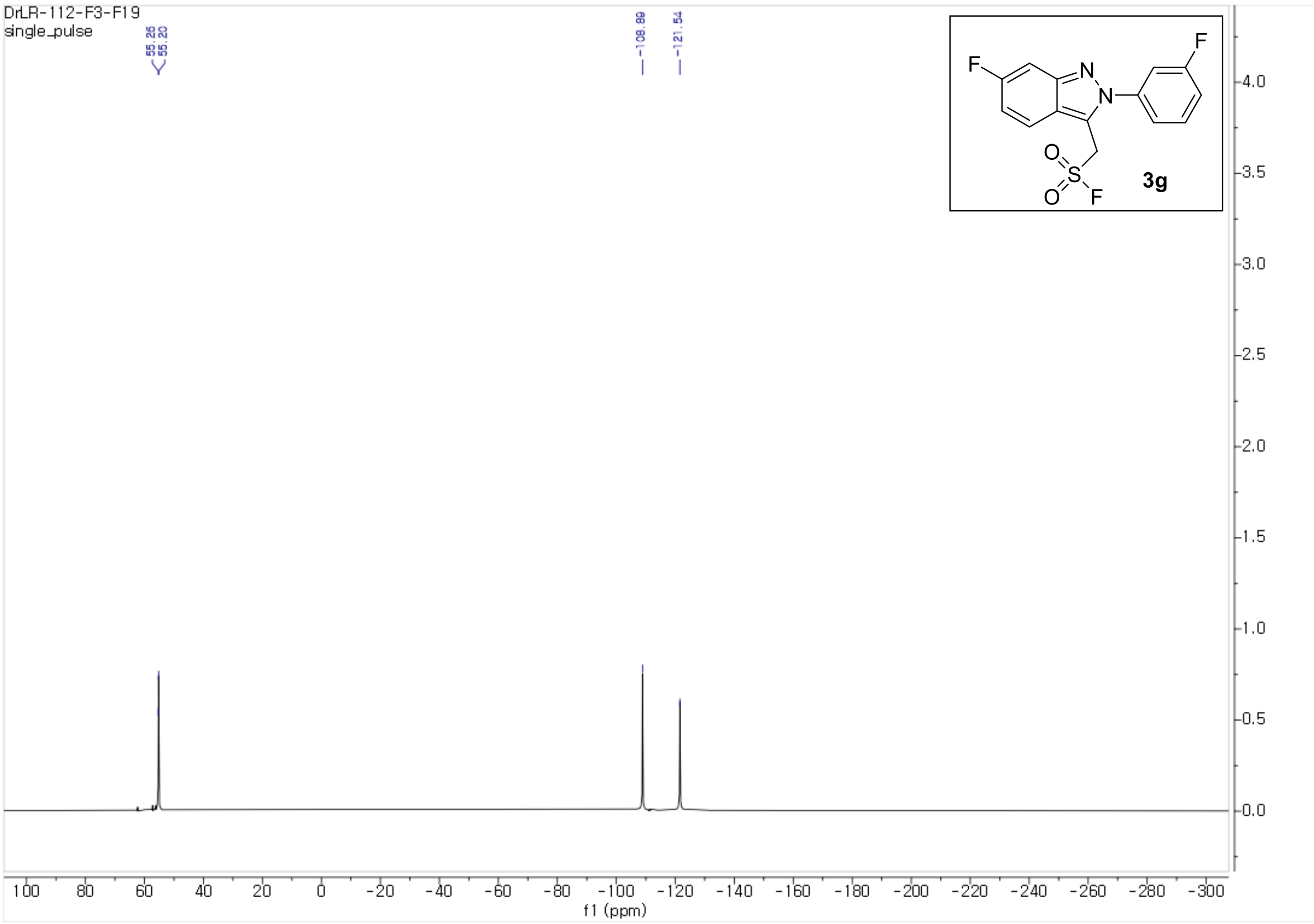
DrLR-112-F3-F19
single_pulse
55.26
55.20
-108.89
-121.54
3g
f1 (ppm)

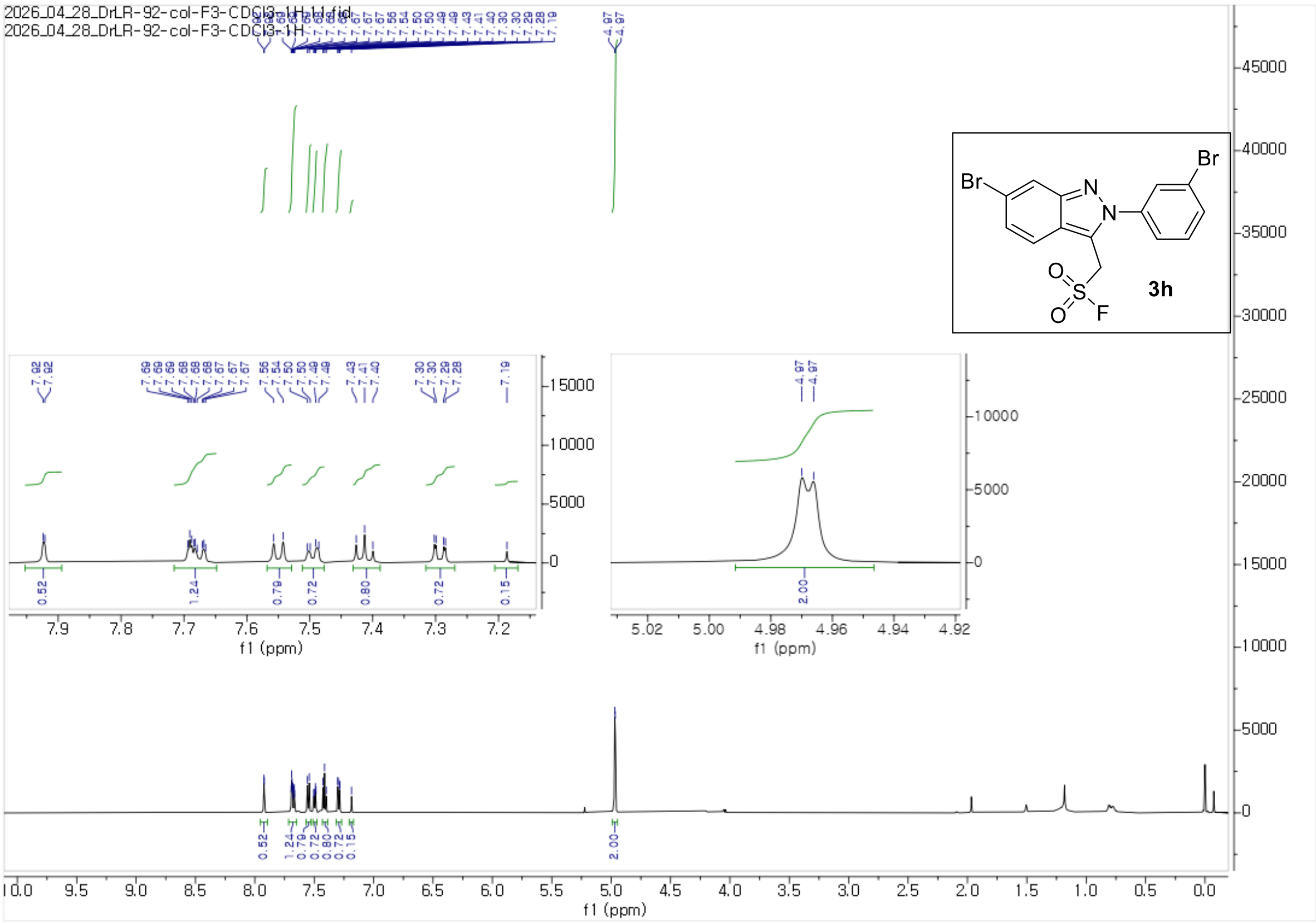

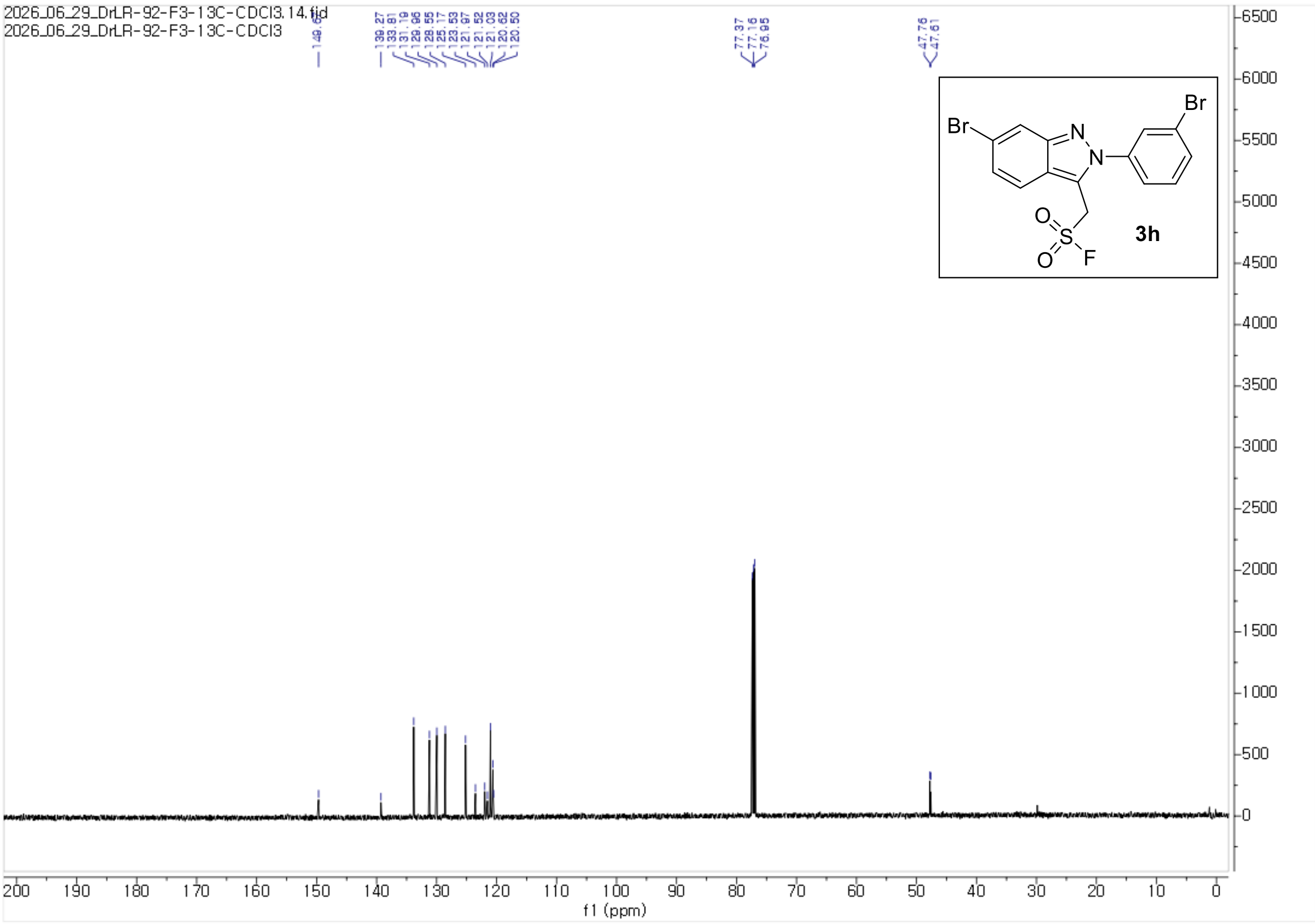

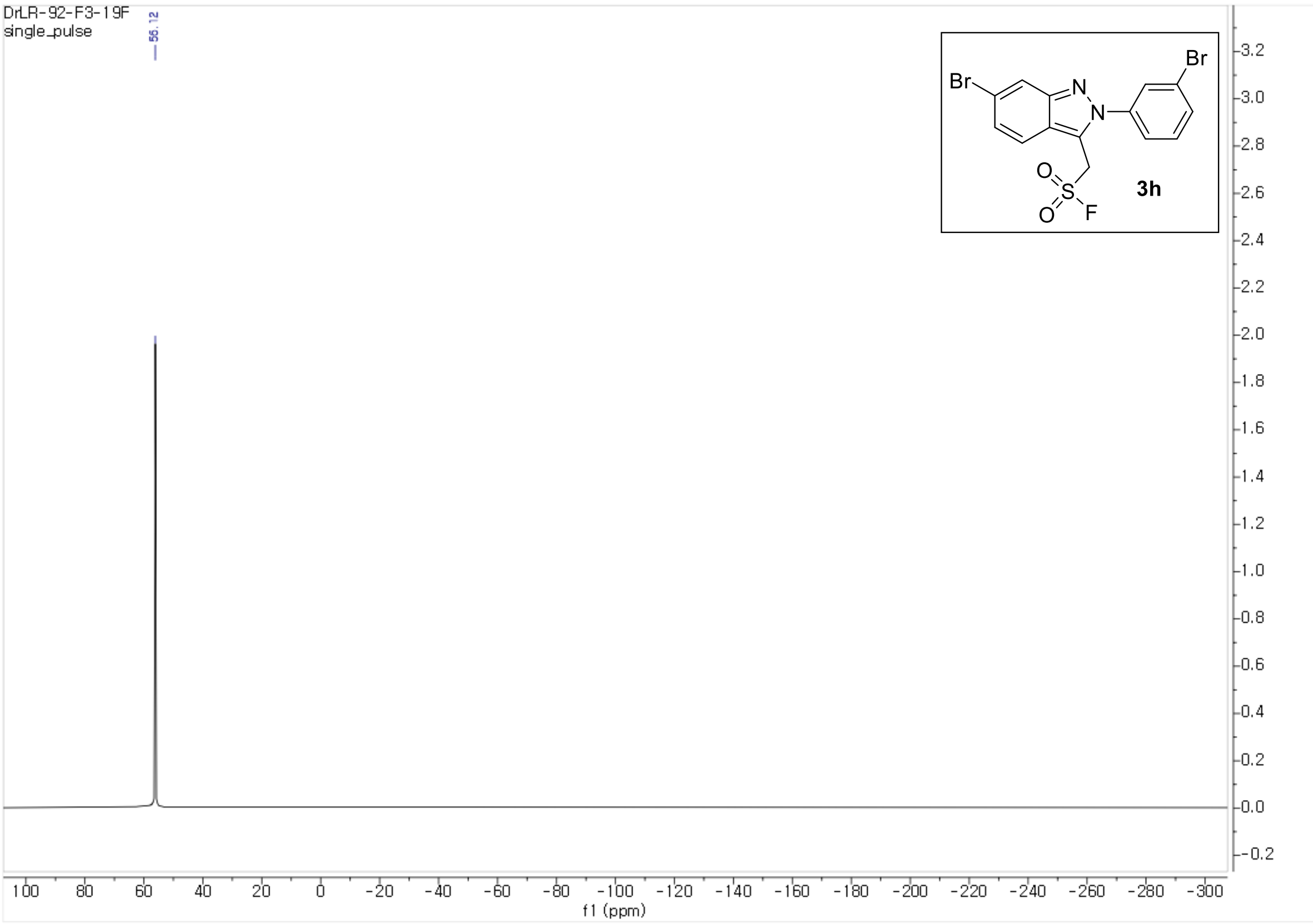
DrLR-92-F3-19F
single_pulse
56.12
3h
Br
Br
N
N
O
S
O
F
f1 (ppm)
100
80
60
40
20
0
-20
-40
-60
-80
-100
-120
-140
-160
-180
-200
-220
-240
-260
-280
-300
3.2
3.0
2.8
2.6
2.4
2.2
2.0
1.8
1.6
1.4
1.2
1.0
0.8
0.6
0.4
0.2
0.0
-0.2

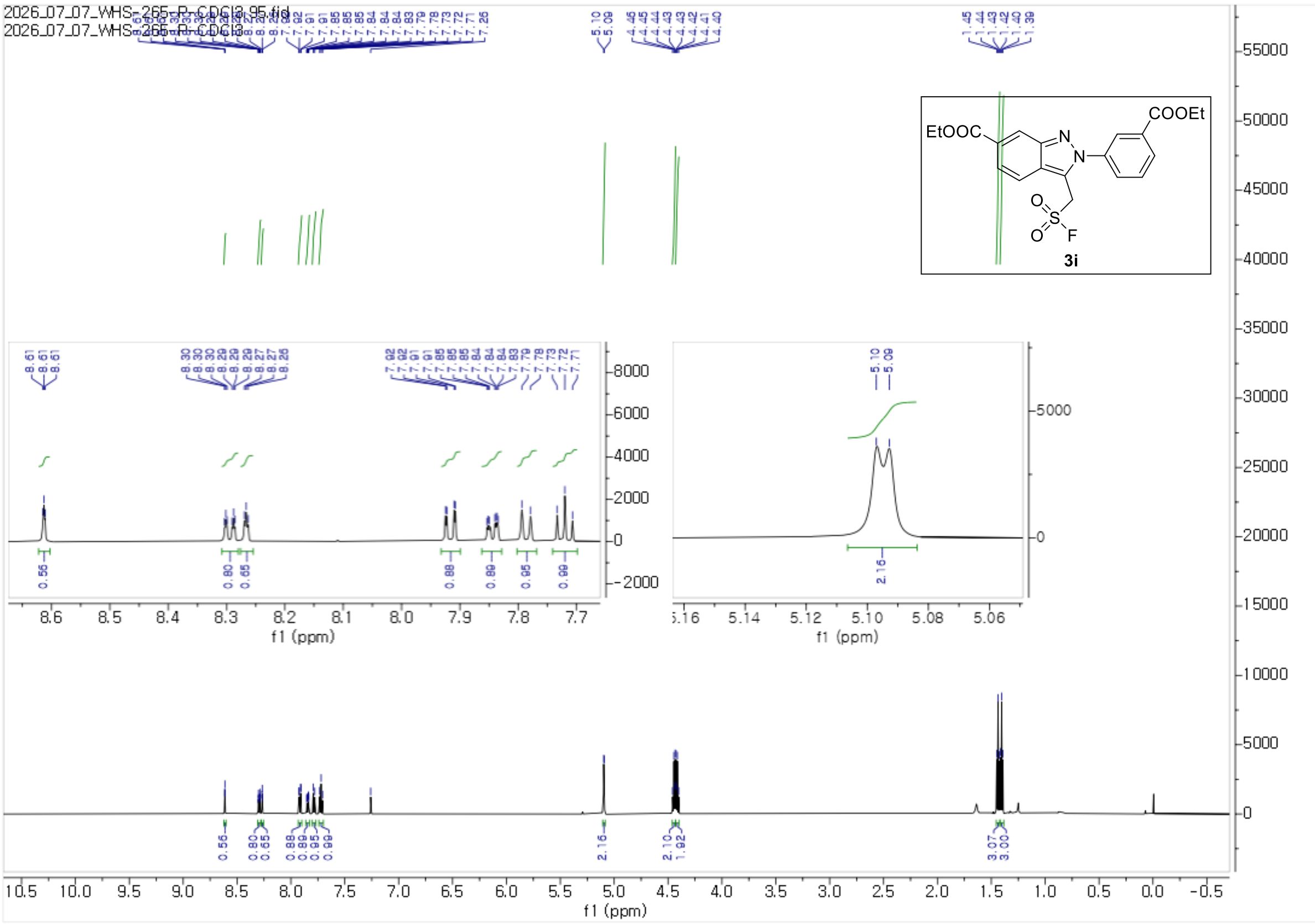

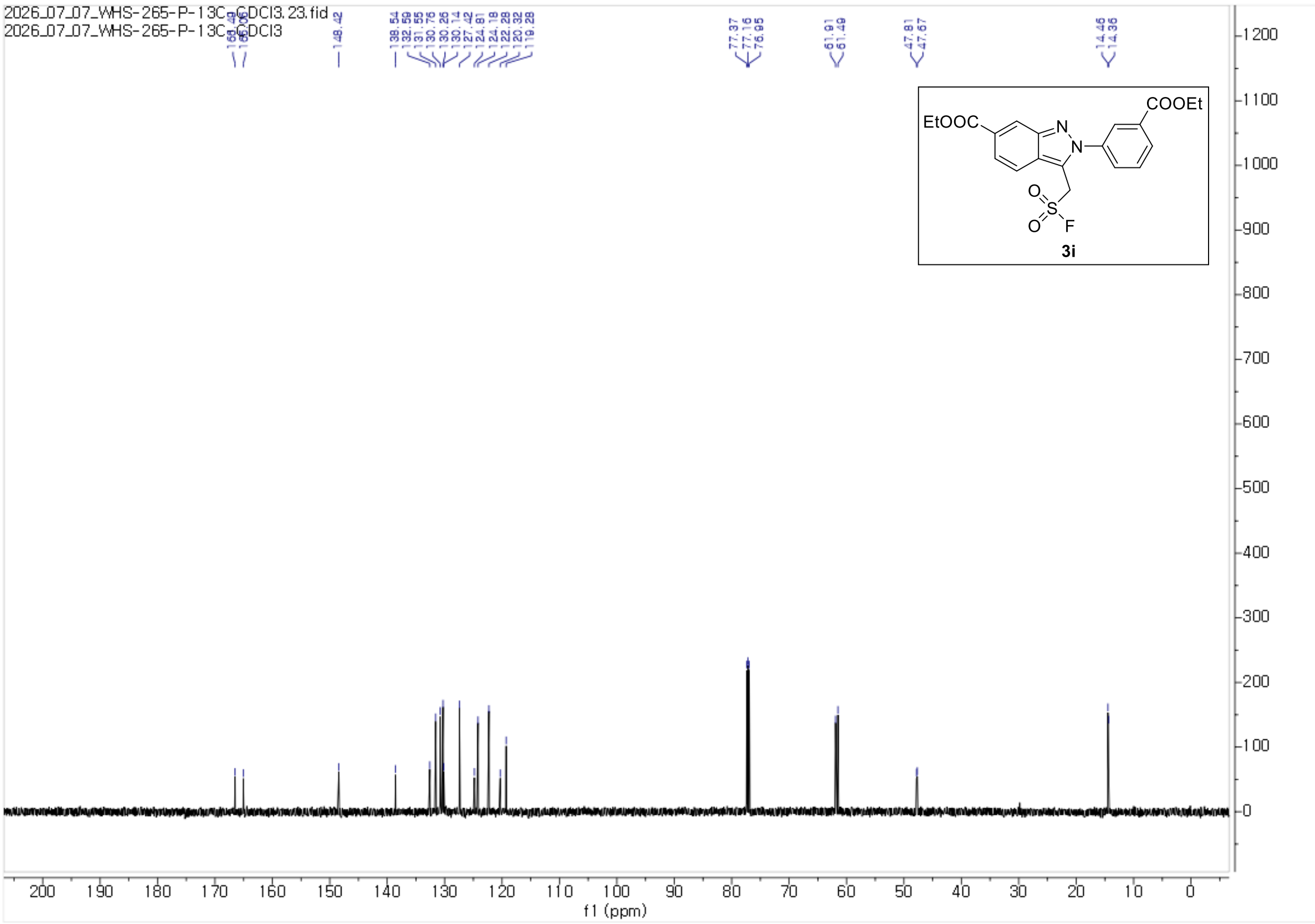

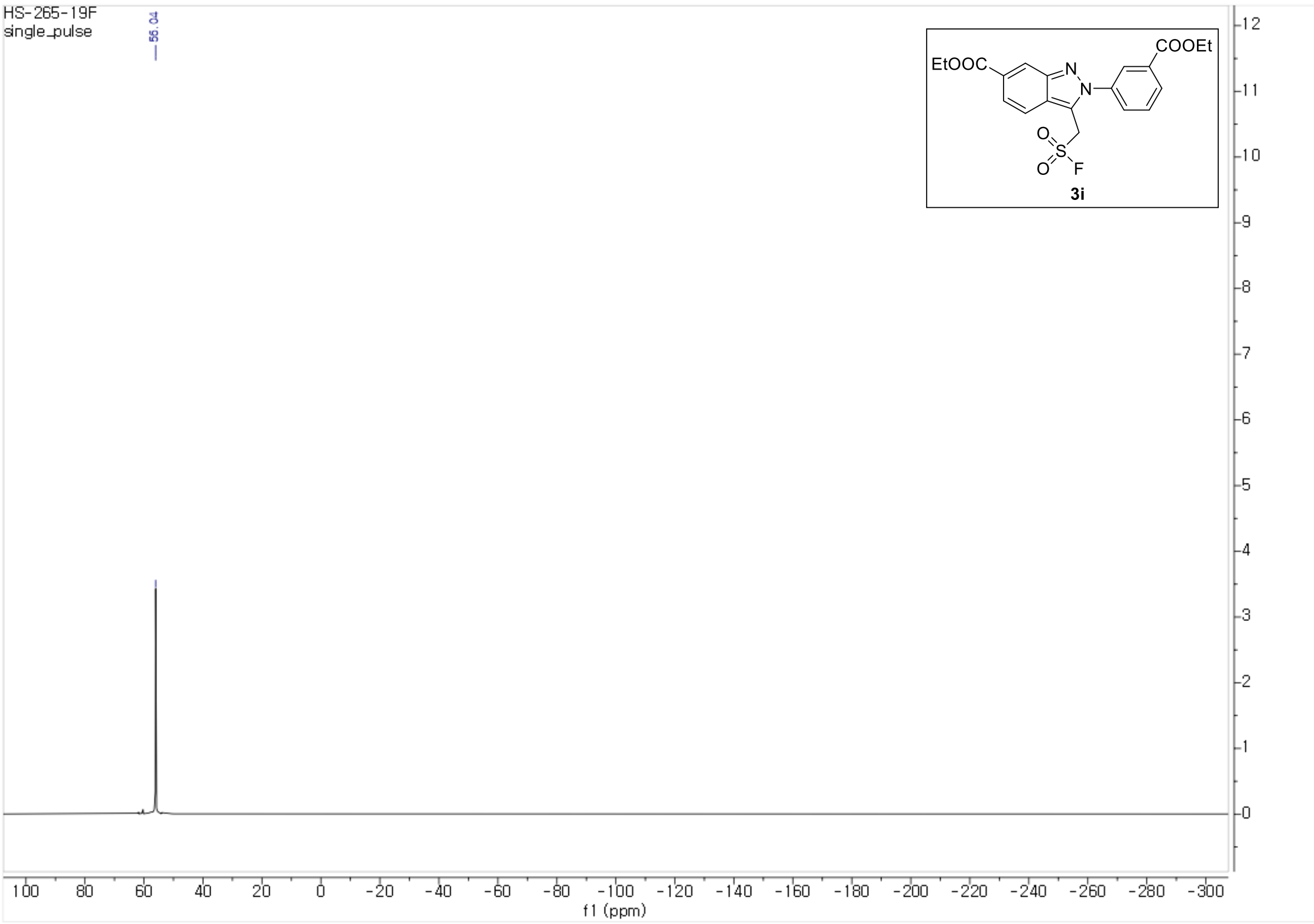
HS-265-19F
single_pulse
55.04
EtOOC
COOEt
N
N
O
S
O
F
3i
100
80
60
40
20
0
-20
-40
-60
-80
-100
-120
-140
-160
-180
-200
-220
-240
-260
-280
-300
f1 (ppm)
12
11
10
9
8
7
6
5
4
3
2
1
0

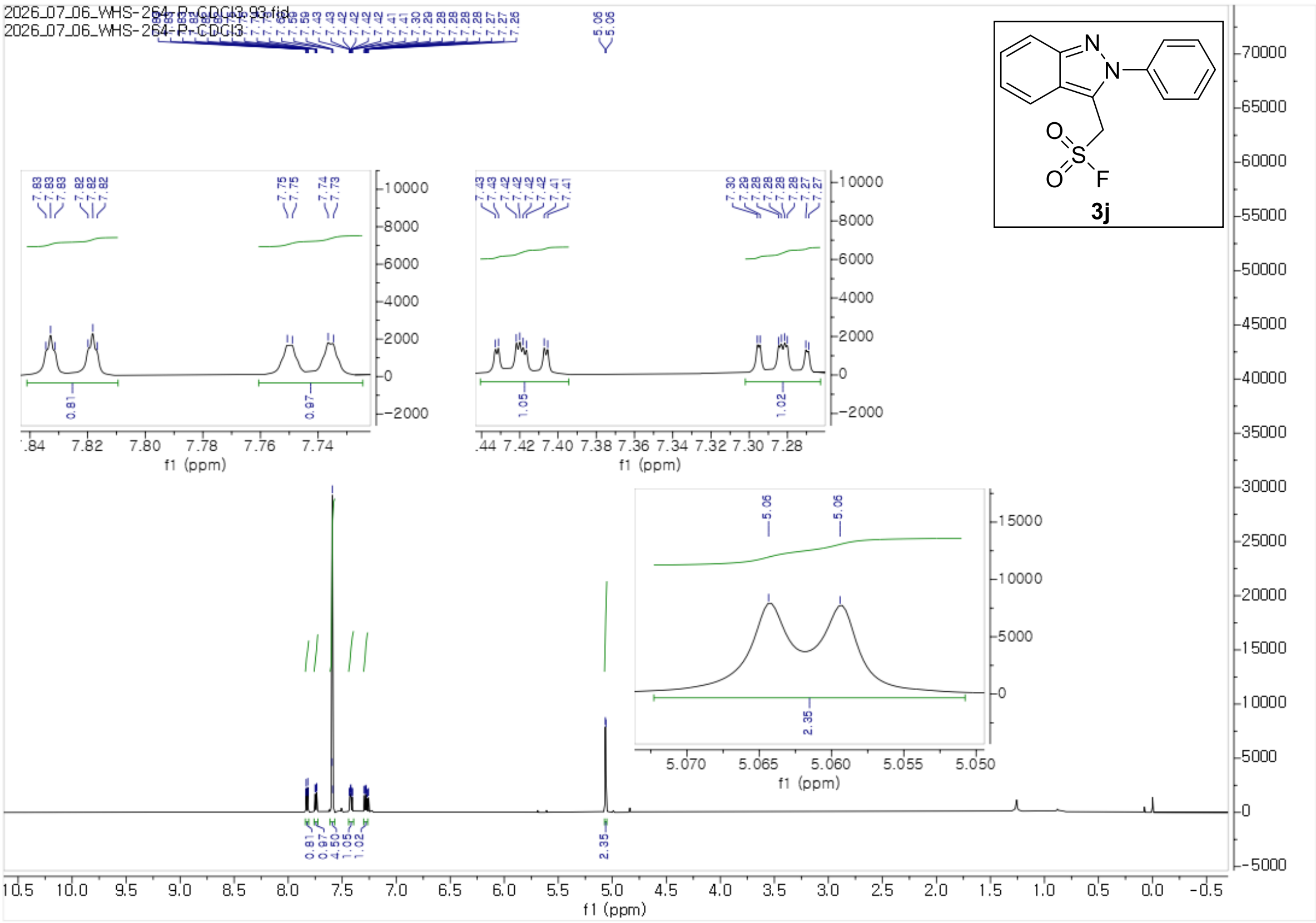

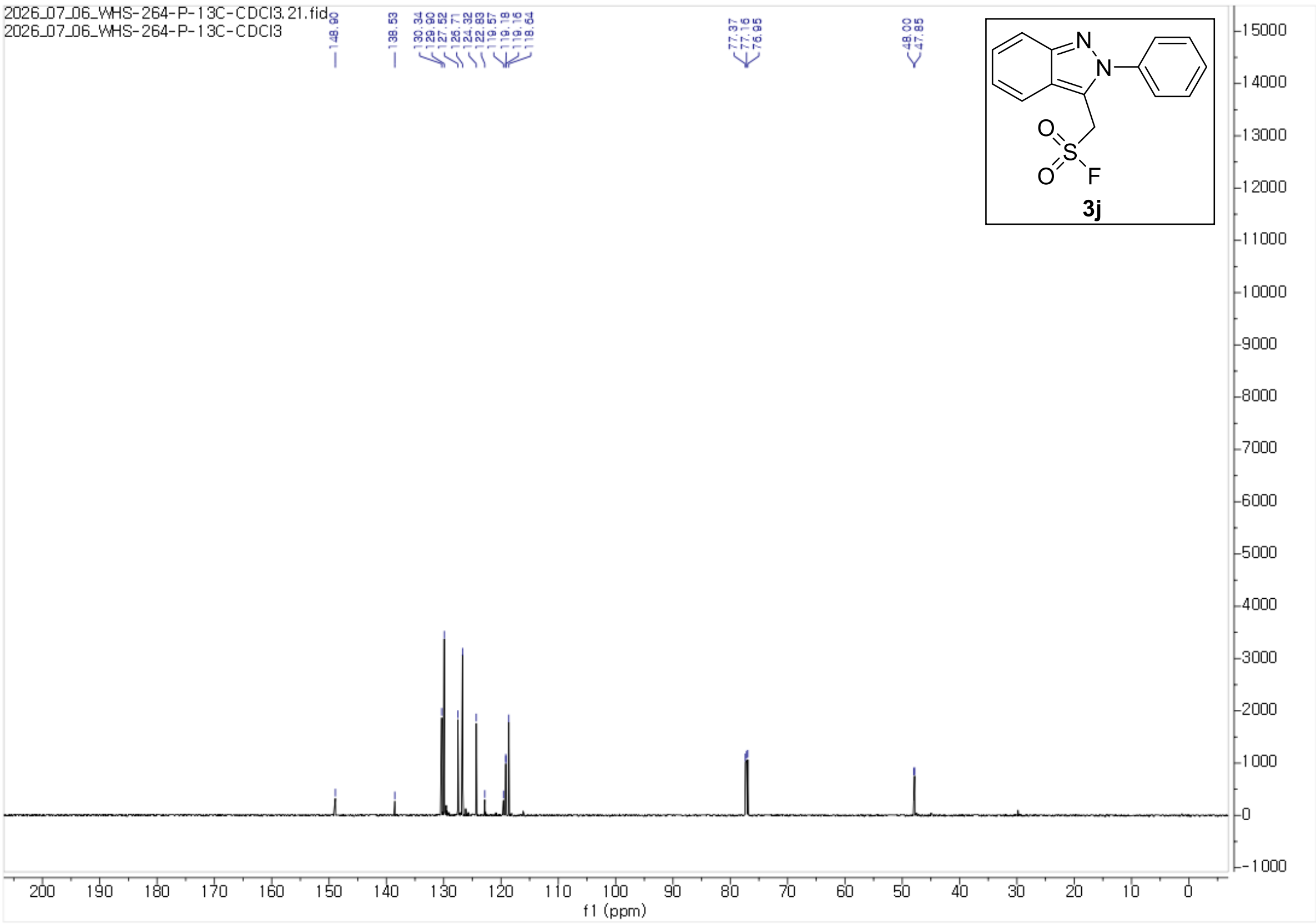

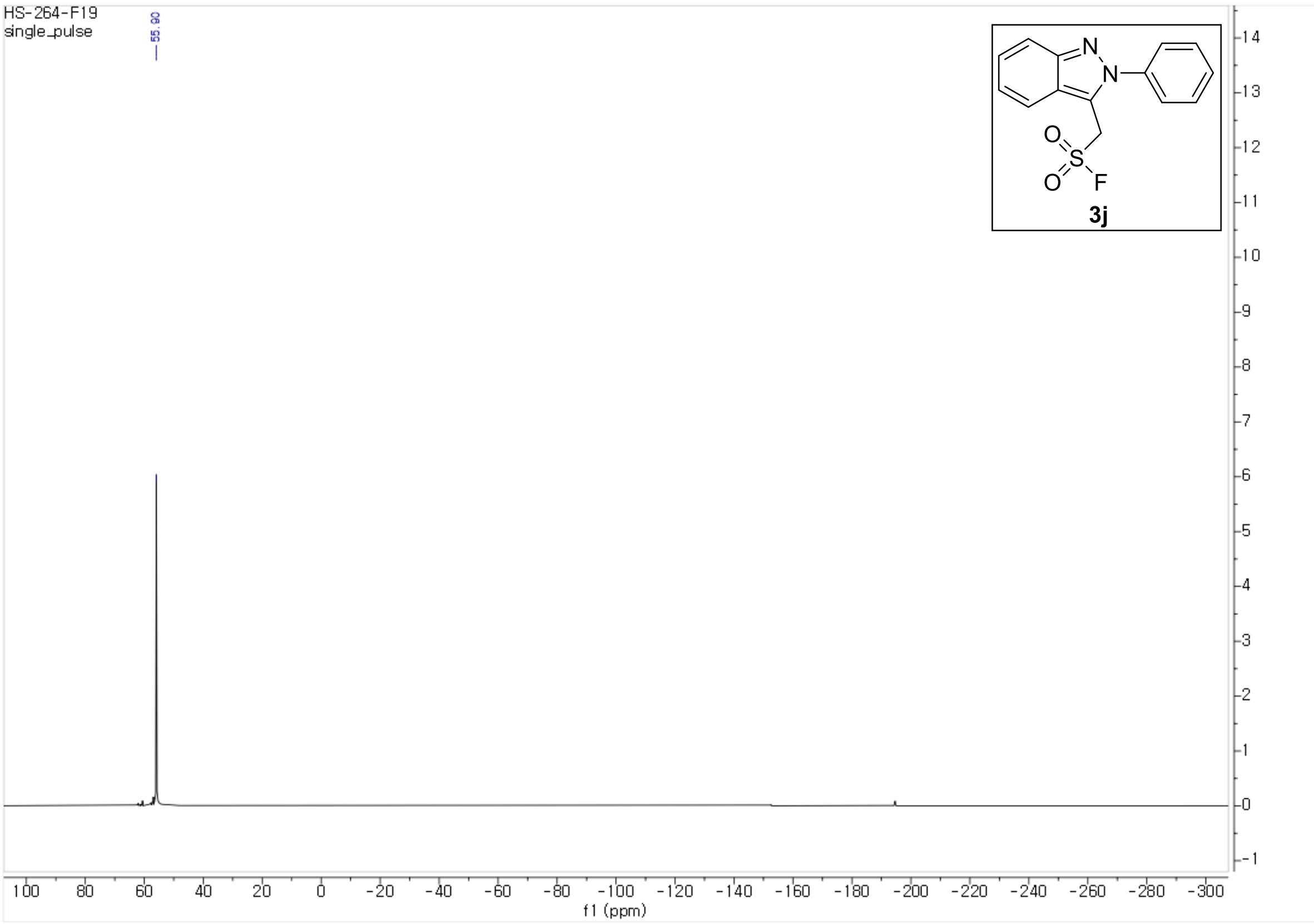

HS-264-F19
single_pulse
55.90
3j
f1 (ppm)

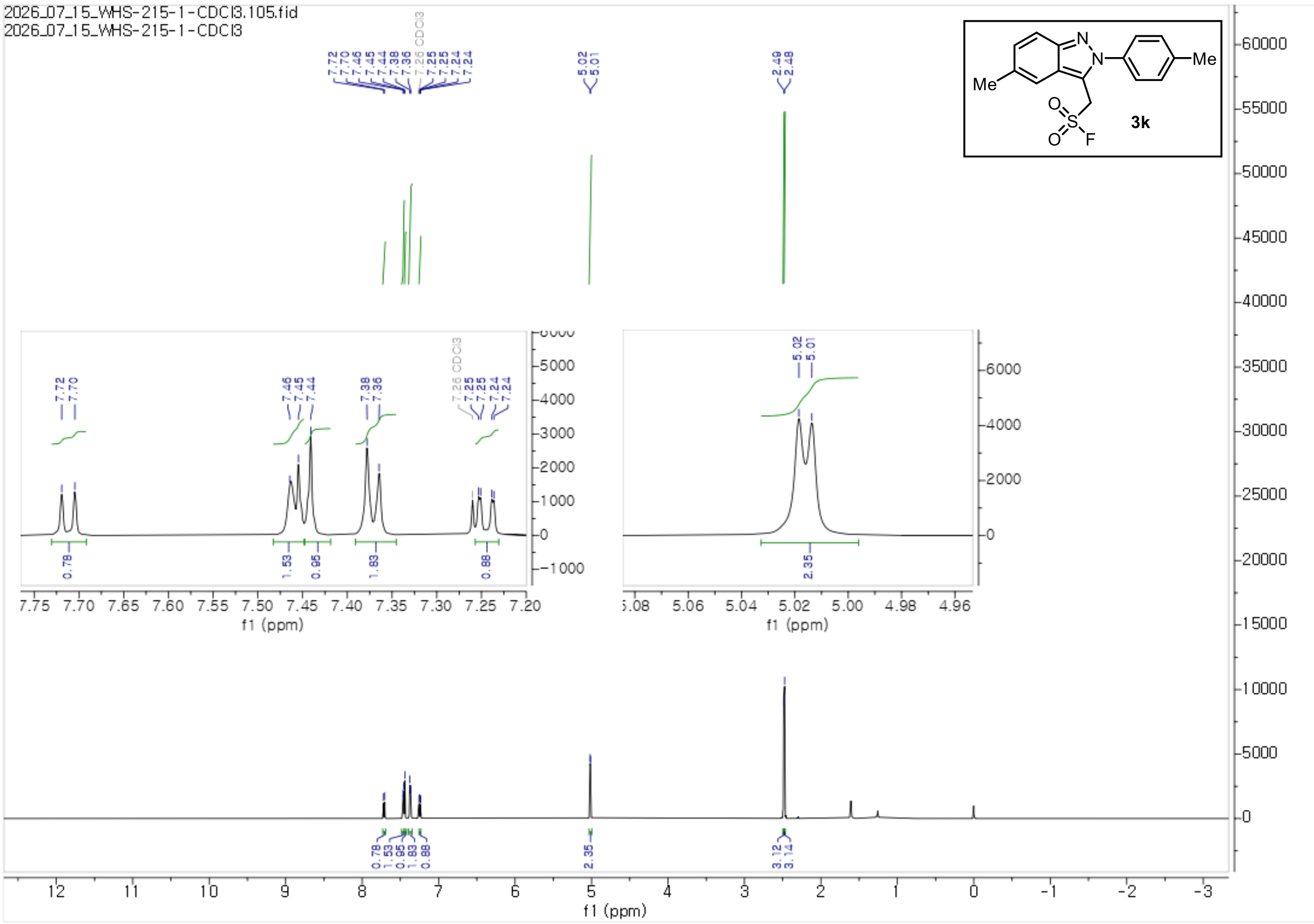
2026_07_15_WHS-215-1-CDCl3.105.fid
2026_07_15_WHS-215-1-CDCl3
3k
f1 (ppm)

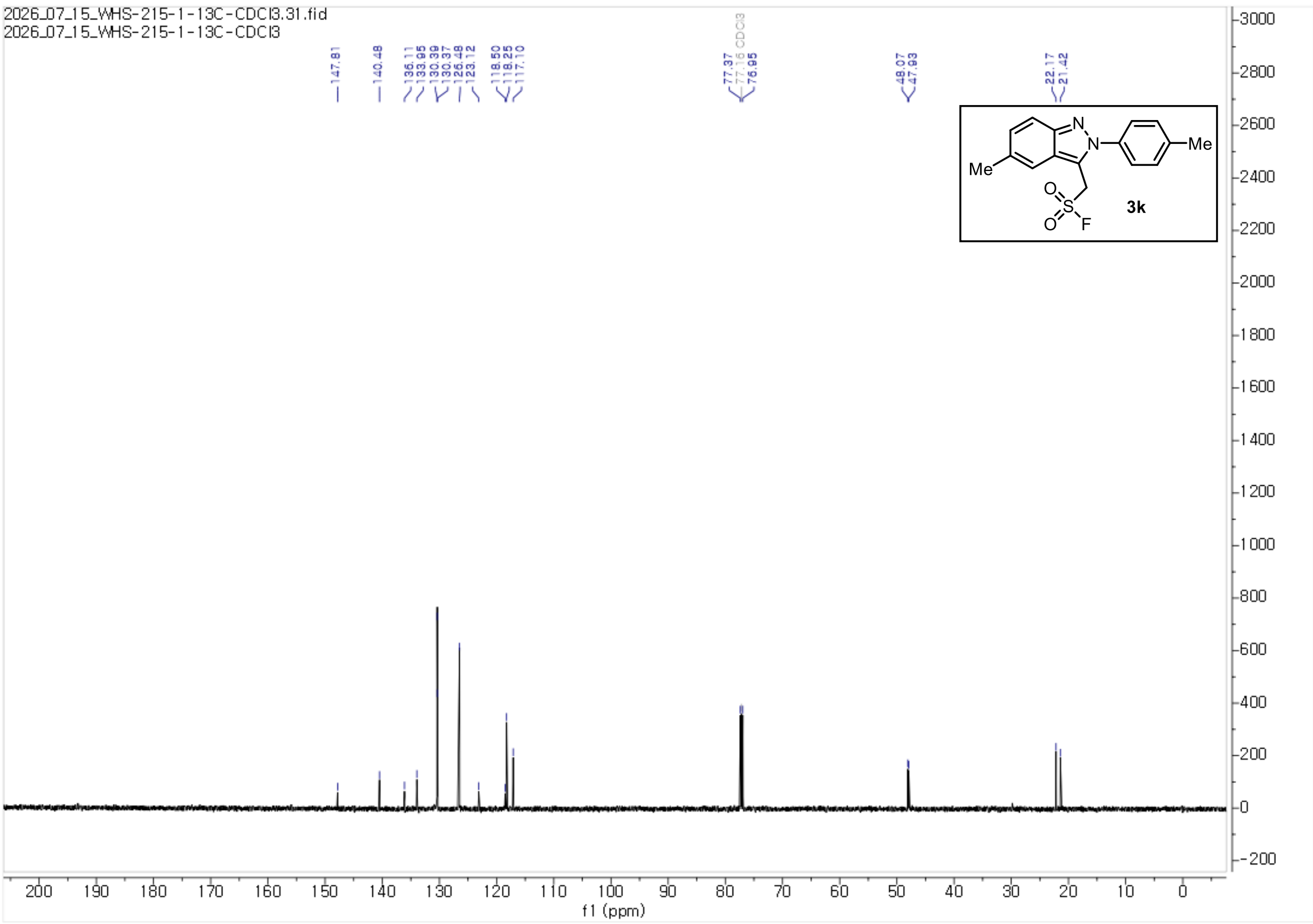
2026_07_15_WHS-215-1-13C-CDCl3.31.fid
2026_07_15_WHS-215-1-13C-CDCl3
3k
f1 (ppm)

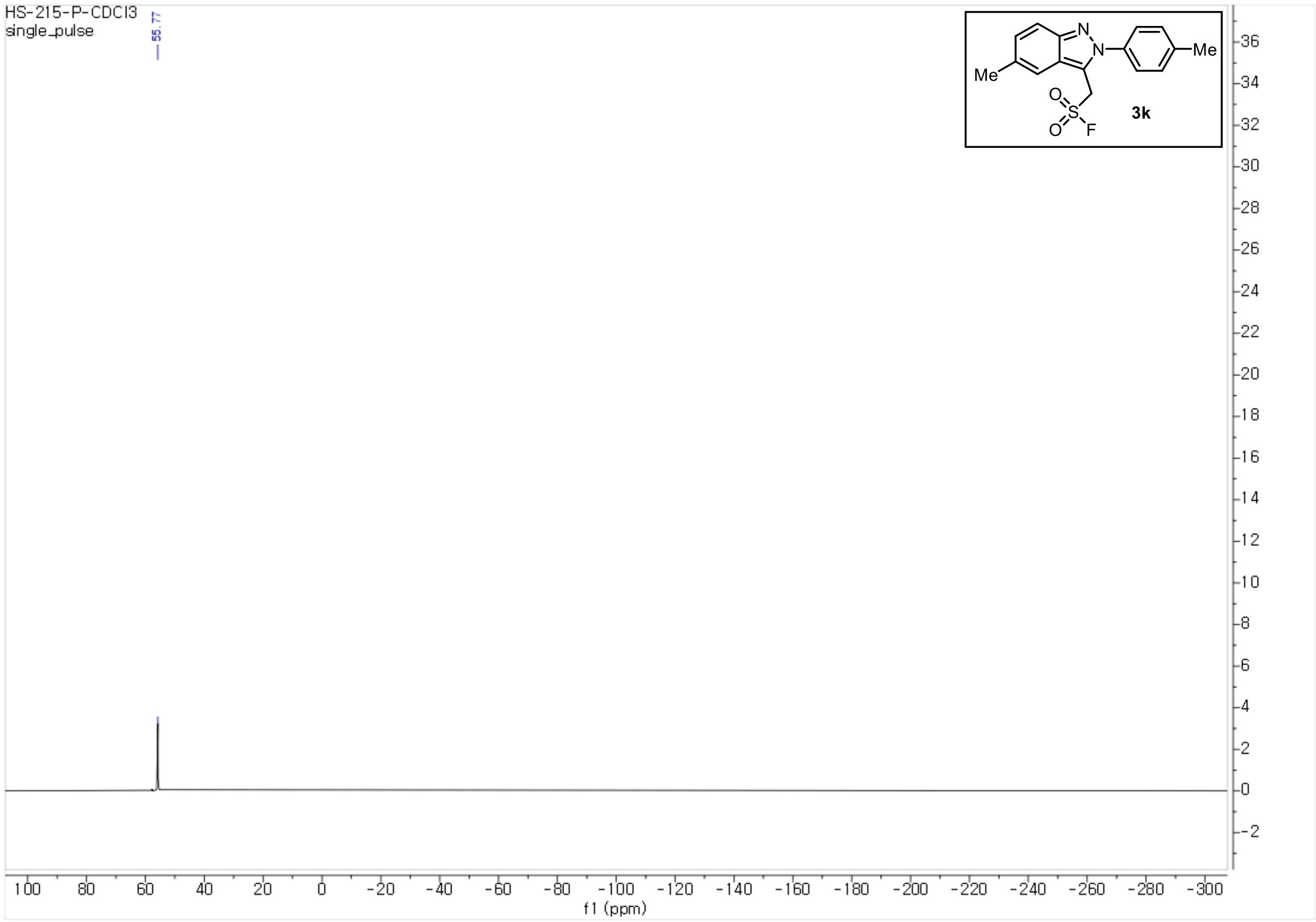
HS-215-P-CDCl3
single_pulse
55.77
Me
N
N
Me
O
S
O
F
3k
f1 (ppm)

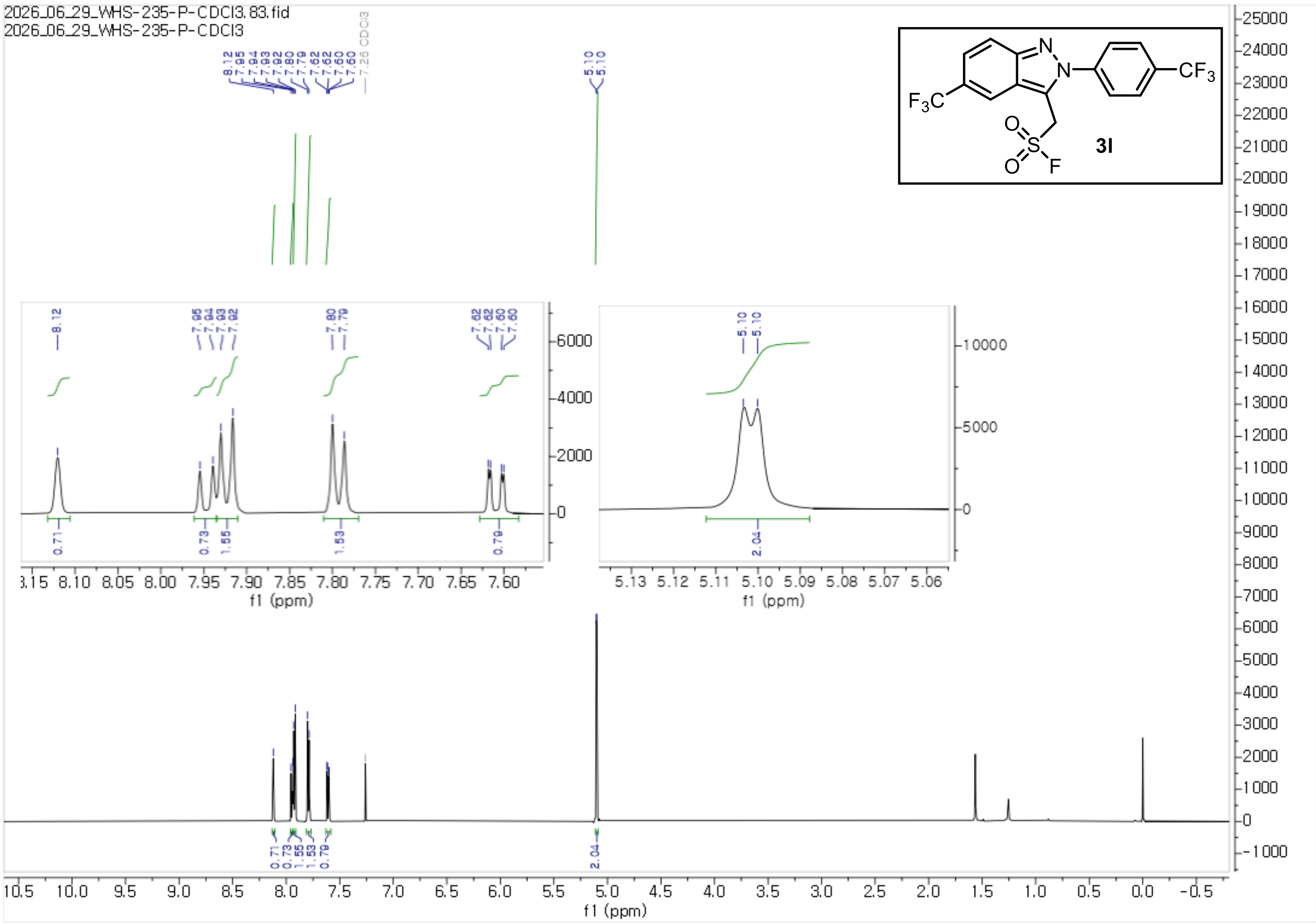
2026_06_29_WHS-235-P-CDCl3.83.fid
2026_06_29_WHS-235-P-CDCl3
3l
f1 (ppm)

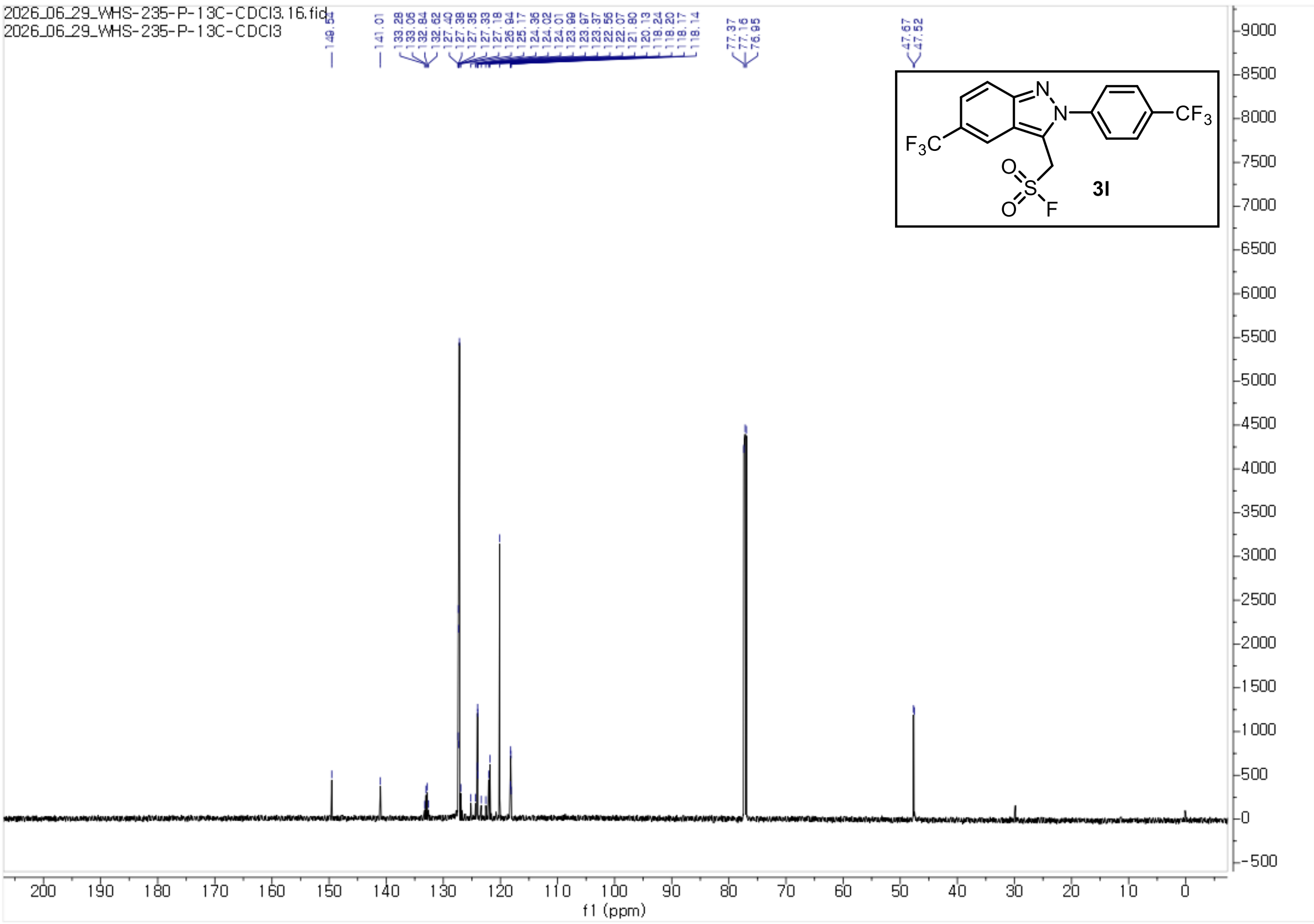
2026_06_29_WHS-235-P-13C-CDCl3.16.fid
2026_06_29_WHS-235-P-13C-CDCl3
3l
f1 (ppm)

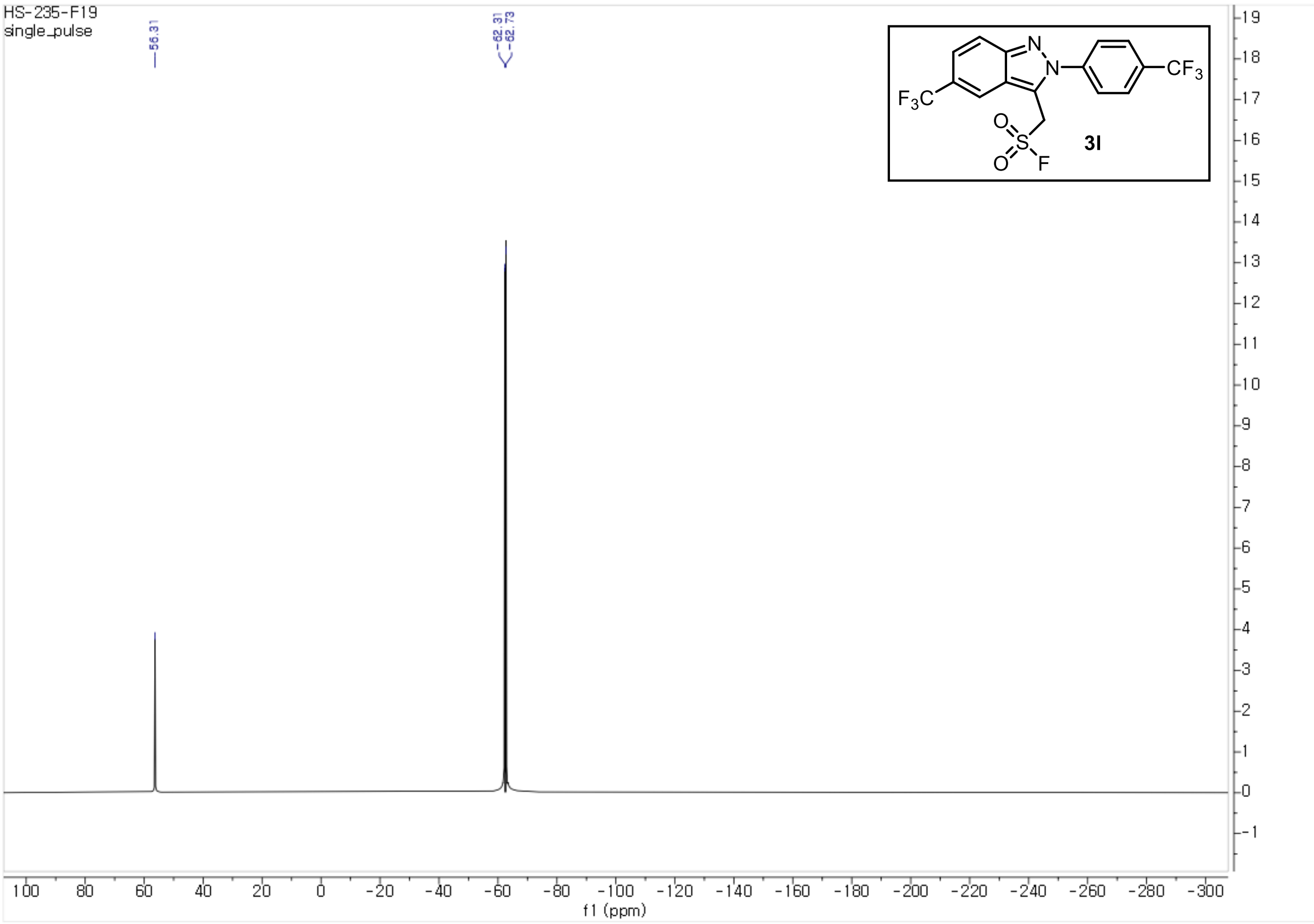
HS-235-F19
single_pulse
56.31
-62.31
-62.73
F3C
N
N
CF3
O
S
O
F
3l
19 18 17 16 15 14 13 12 11 10 9 8 7 6 5 4 3 2 1 0 -1
100 80 60 40 20 0 -20 -40 -60 -80 -100 -120 -140 -160 -180 -200 -220 -240 -260 -280 -300
f1 (ppm)

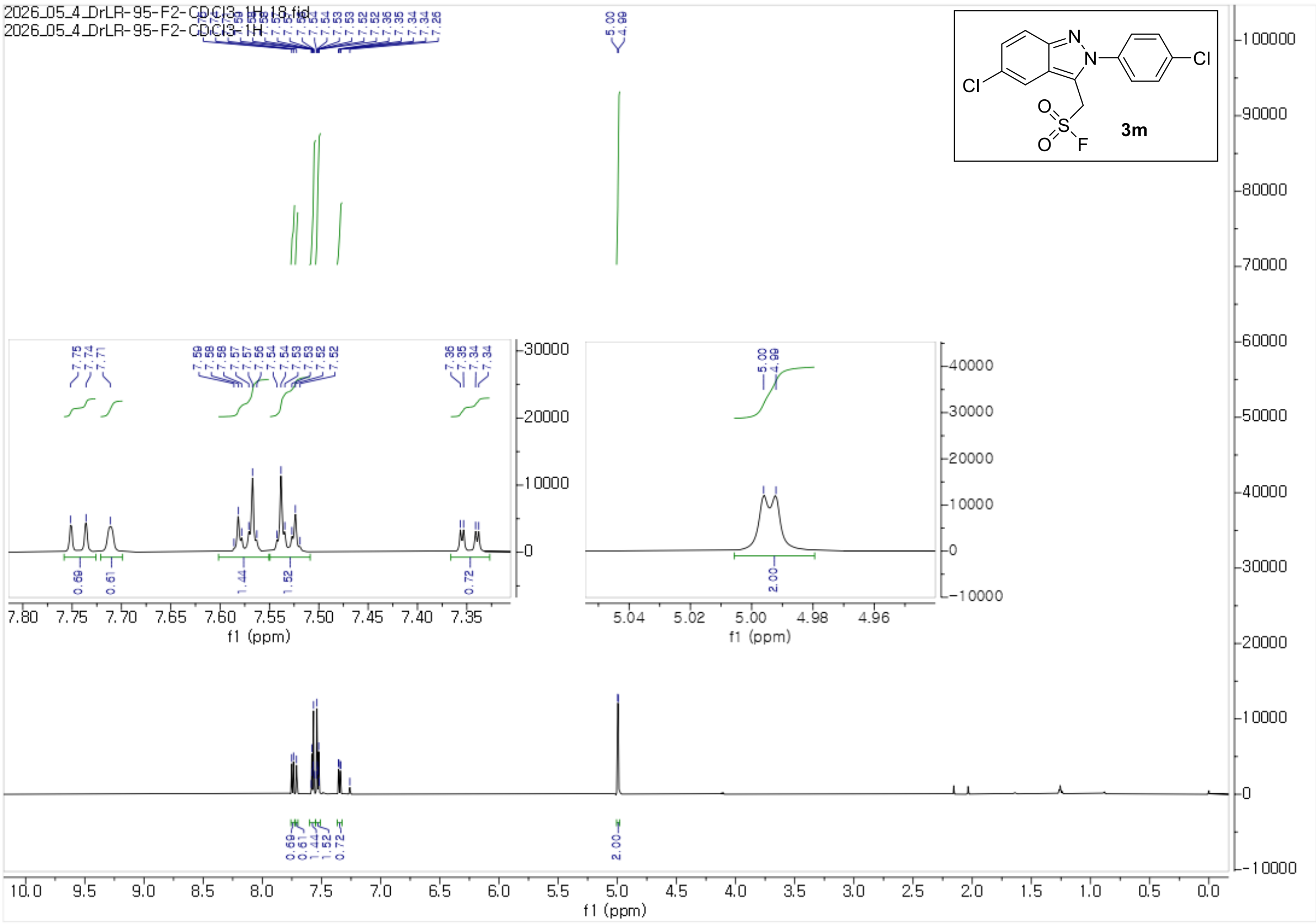
2026_05_4_DrLR-95-F2-CDCl3-1H.18.fid
2026_05_4_DrLR-95-F2-CDCl3-1H
5.00
4.99
3m
f1 (ppm)

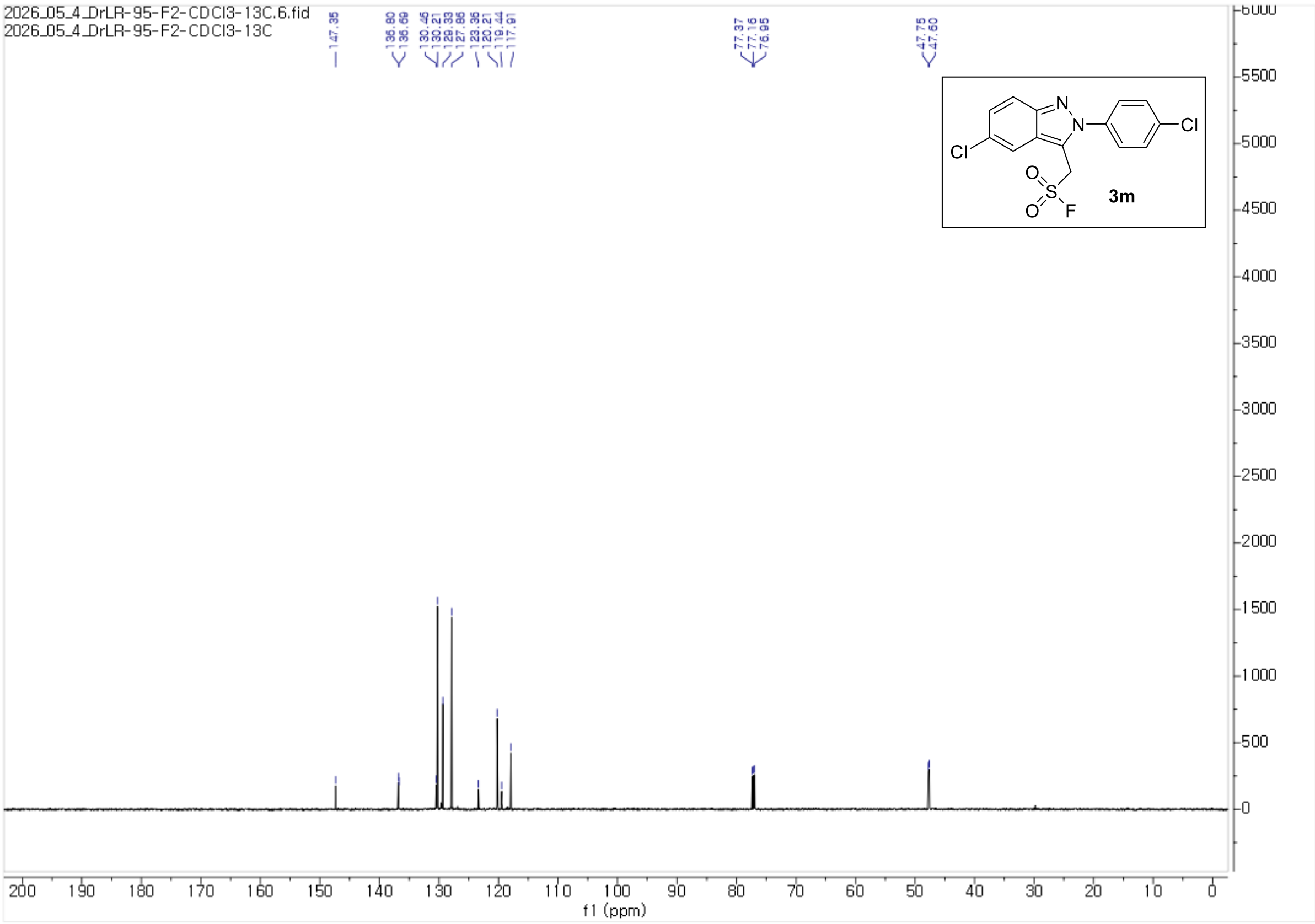
2026_05_4_DrLR-95-F2-CDCl3-13C.6.fid
2026_05_4_DrLR-95-F2-CDCl3-13C
147.35
136.80
136.59
130.46
130.21
129.33
127.86
123.36
120.21
119.44
117.91
77.37
77.16
76.95
47.75
47.60
3m
f1 (ppm)

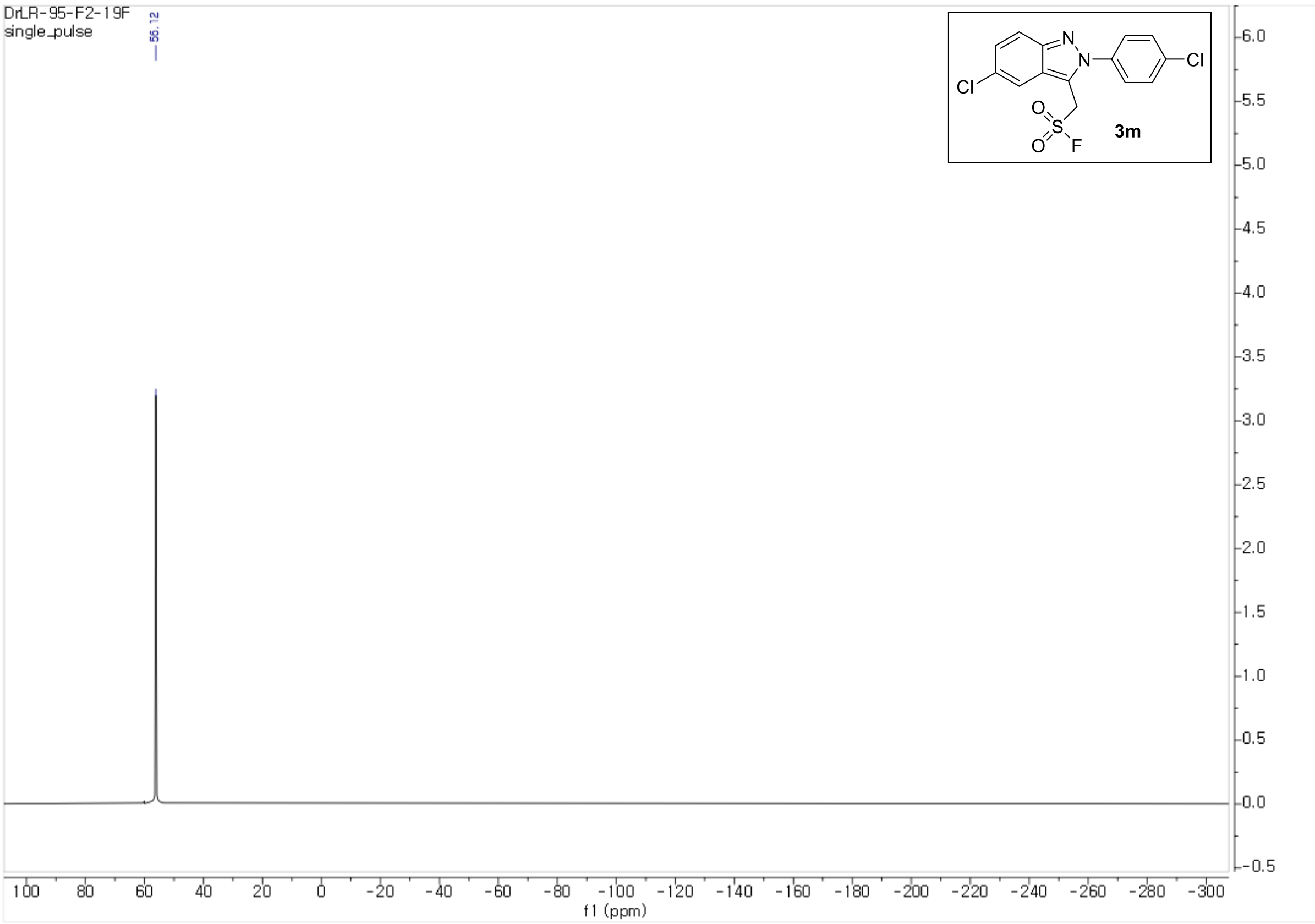
DrLR-95-F2-19F
single_pulse
56.12
Cl
N
N
Cl
O
S
O
F
3m
f1 (ppm)

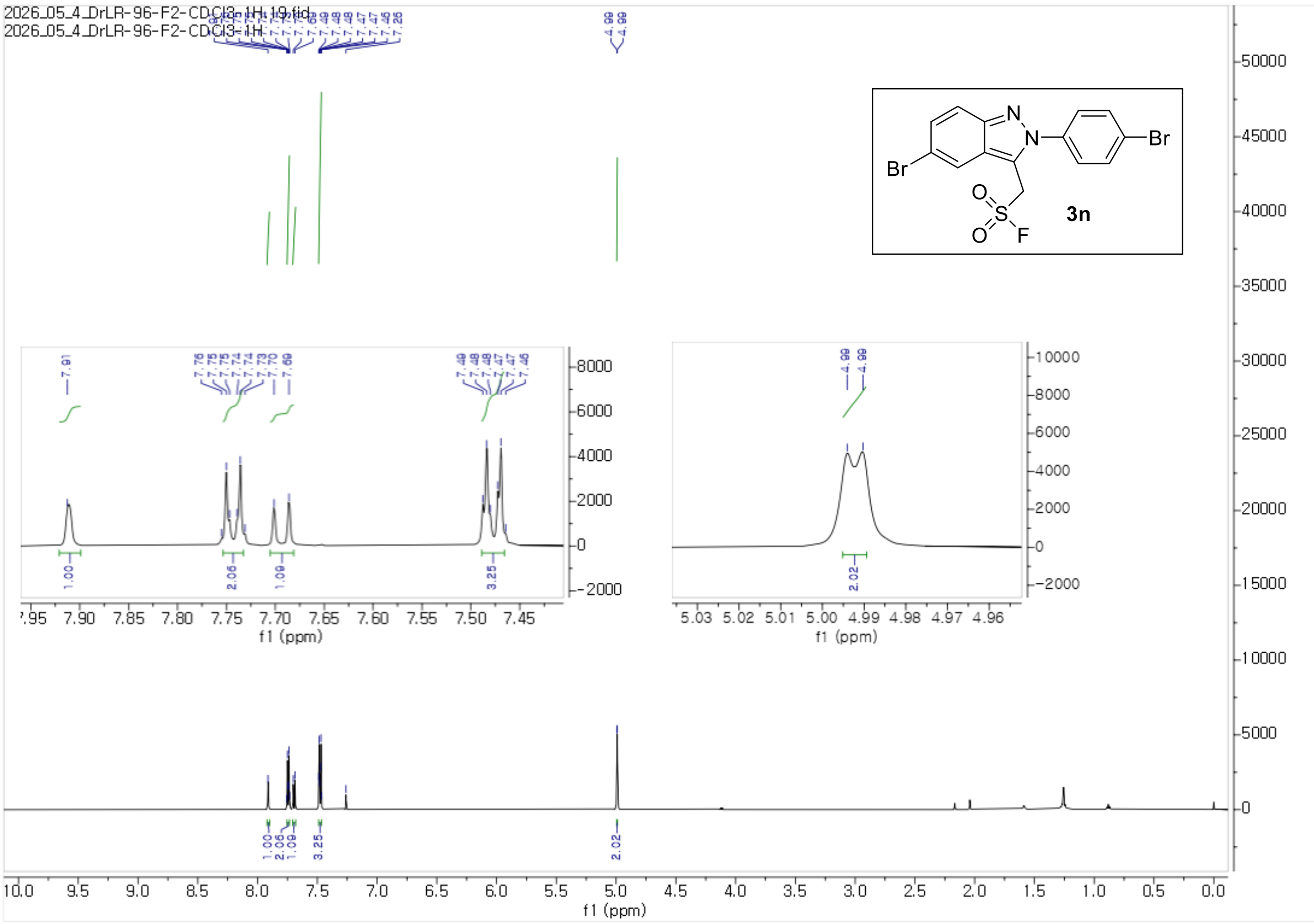

2026_05_4_DrLR-96-F2-CDCl3-1H.19.fid
2026_05_4_DrLR-96-F2-CDCl3-1H
3n
Br
N
F
O
S
f1 (ppm)

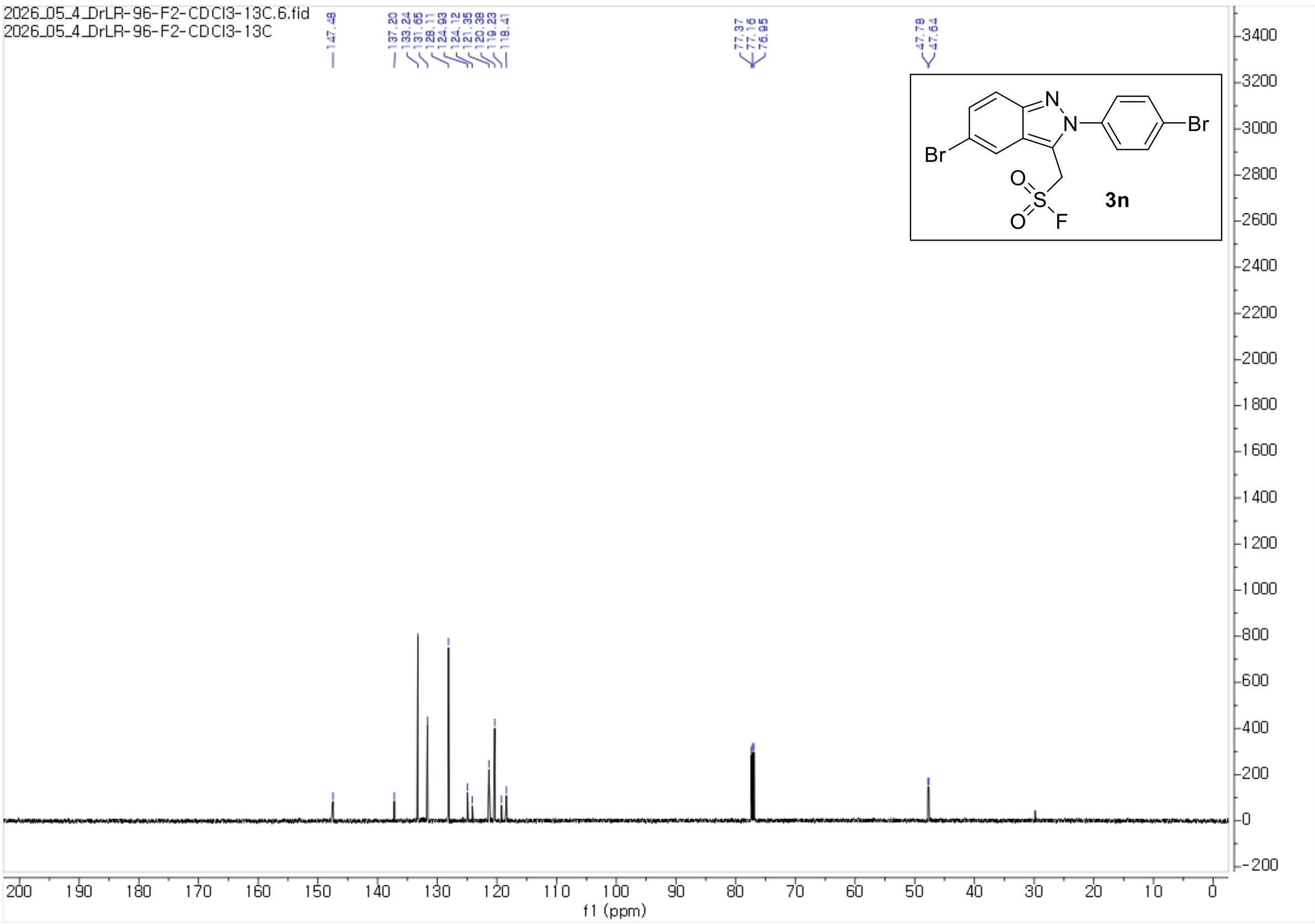

2026_05_4_DrLR-96-F2-CDCl3-13C.6.fid
2026_05_4_DrLR-96-F2-CDCl3-13C
147.48
137.20
133.24
131.65
128.11
124.93
124.12
121.35
120.38
119.23
118.41
77.37
77.16
76.95
47.78
47.64
3n
f1 (ppm)

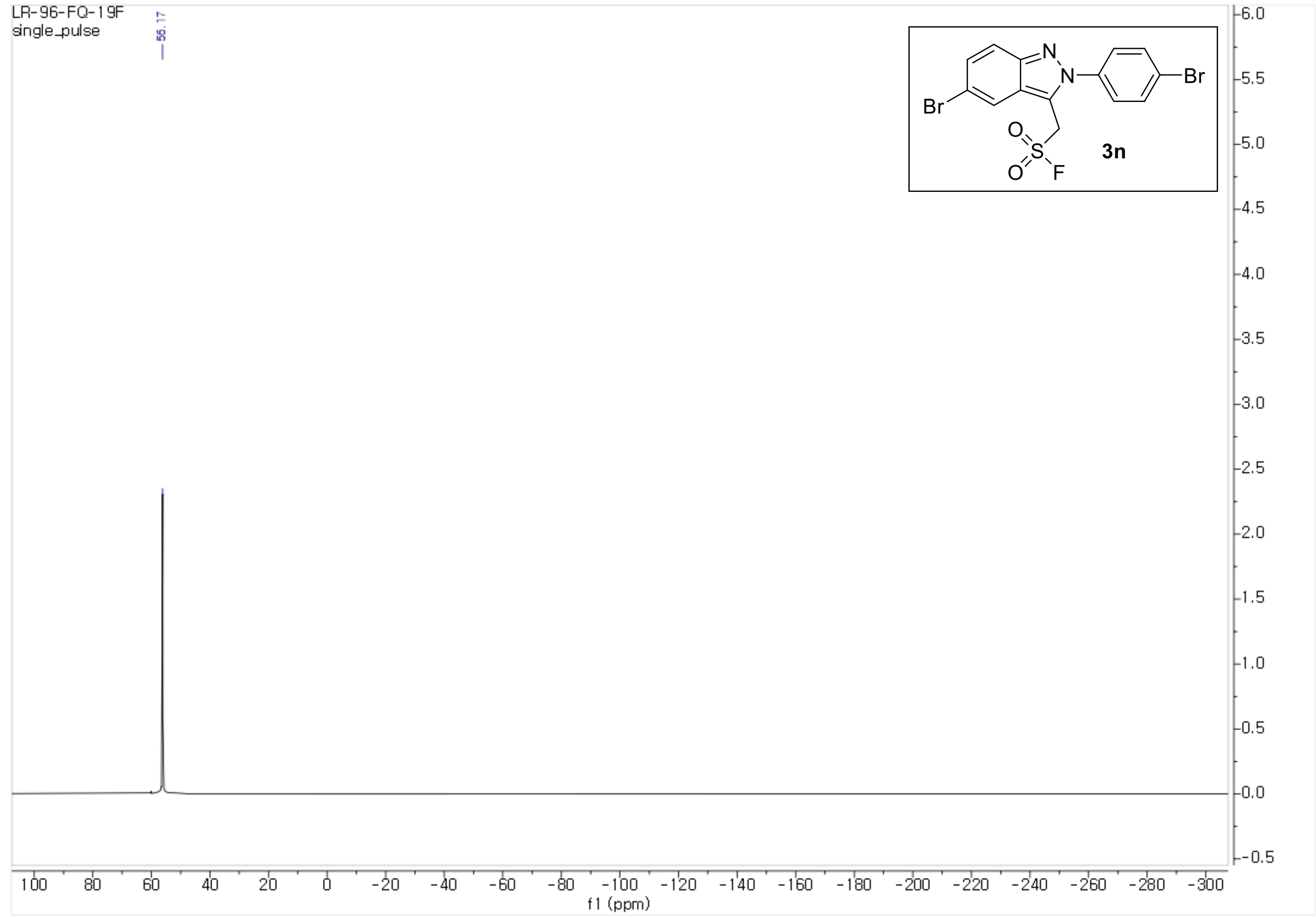
LR-96-FQ-19F
single_pulse
56.17
Br
N
N
Br
O
S
O
F
3n
f1 (ppm)

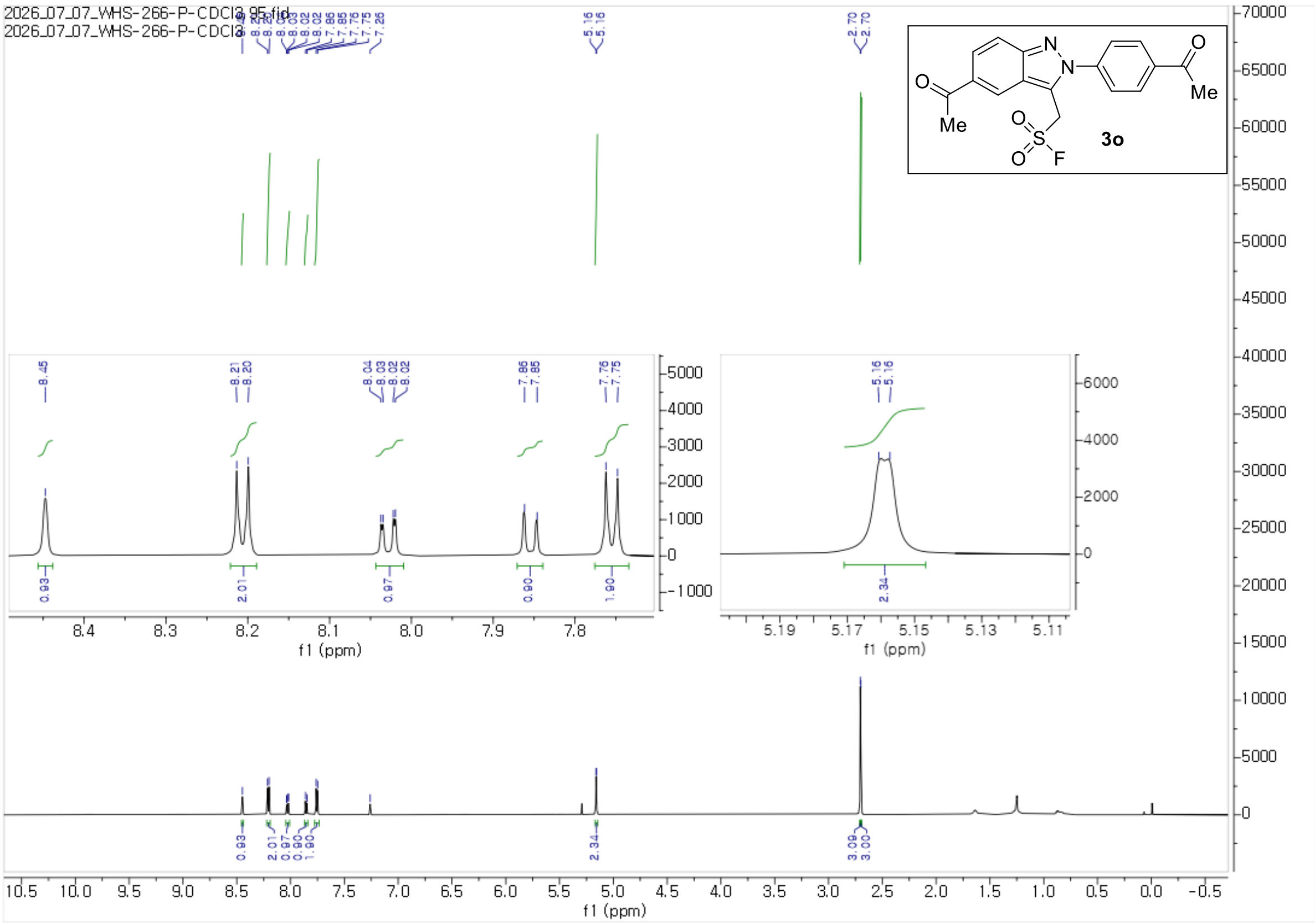

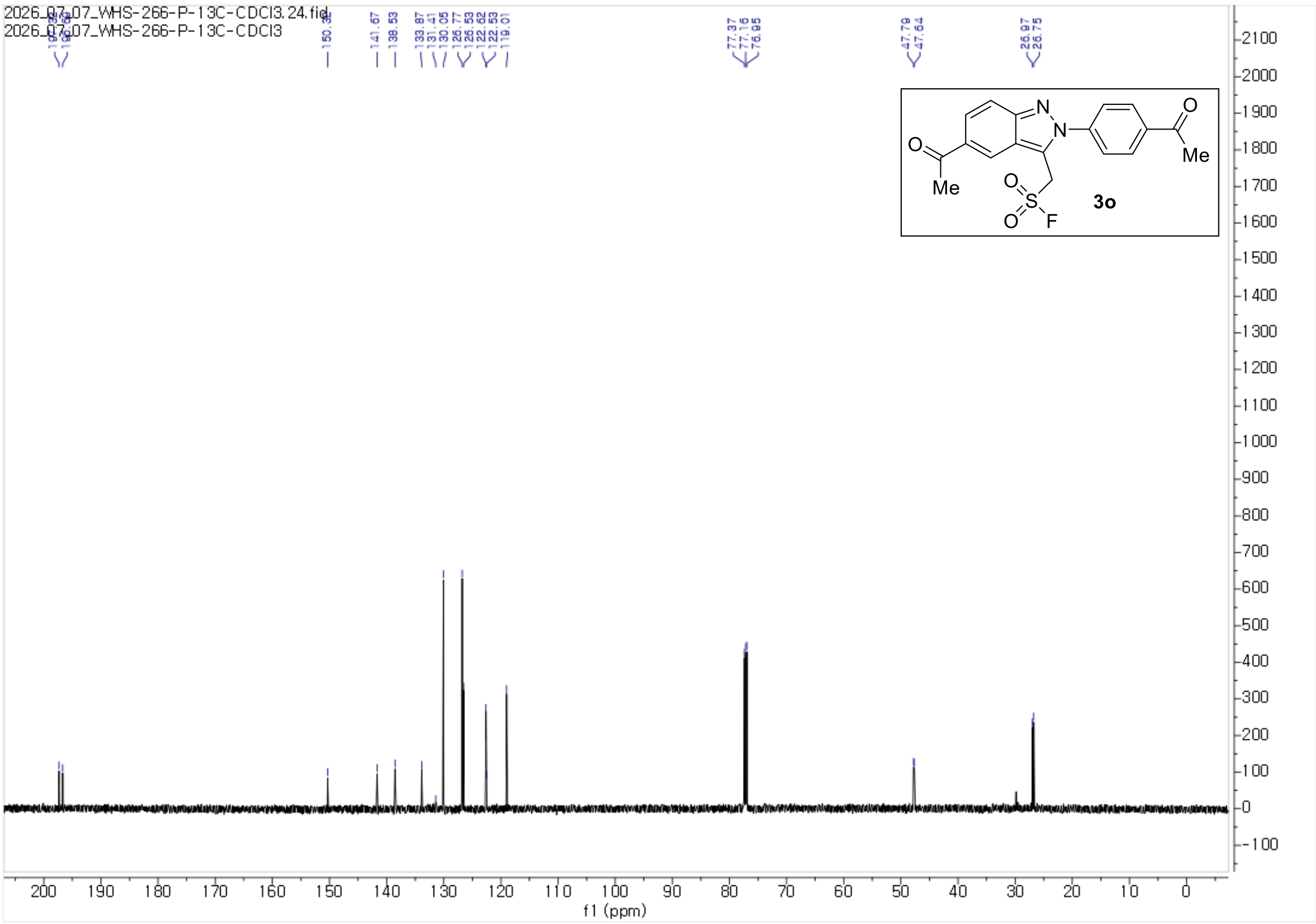

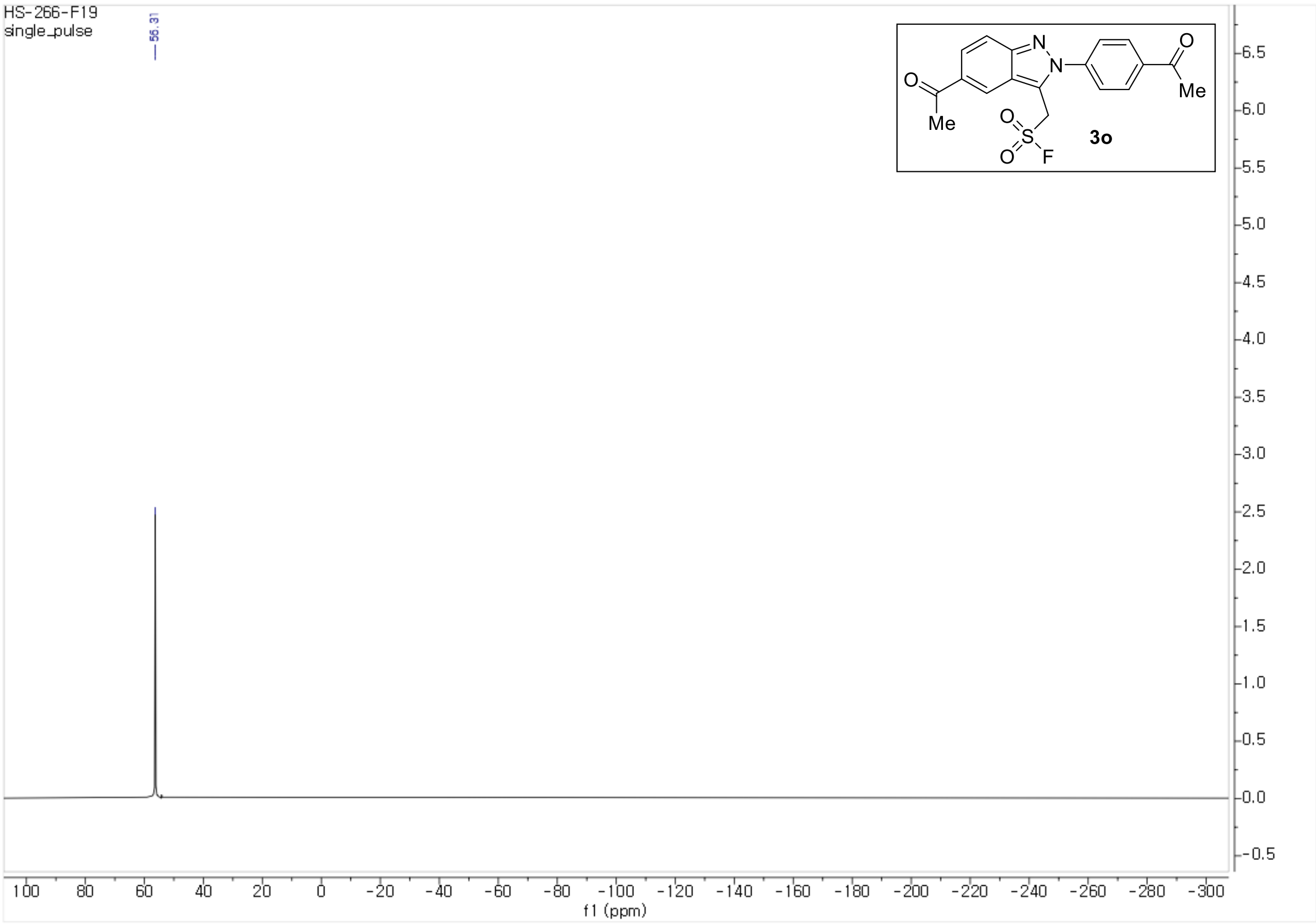
HS-266-F19
single_pulse
56.31
3o
Me
Me
O
O
N
N
S
F
f1 (ppm)

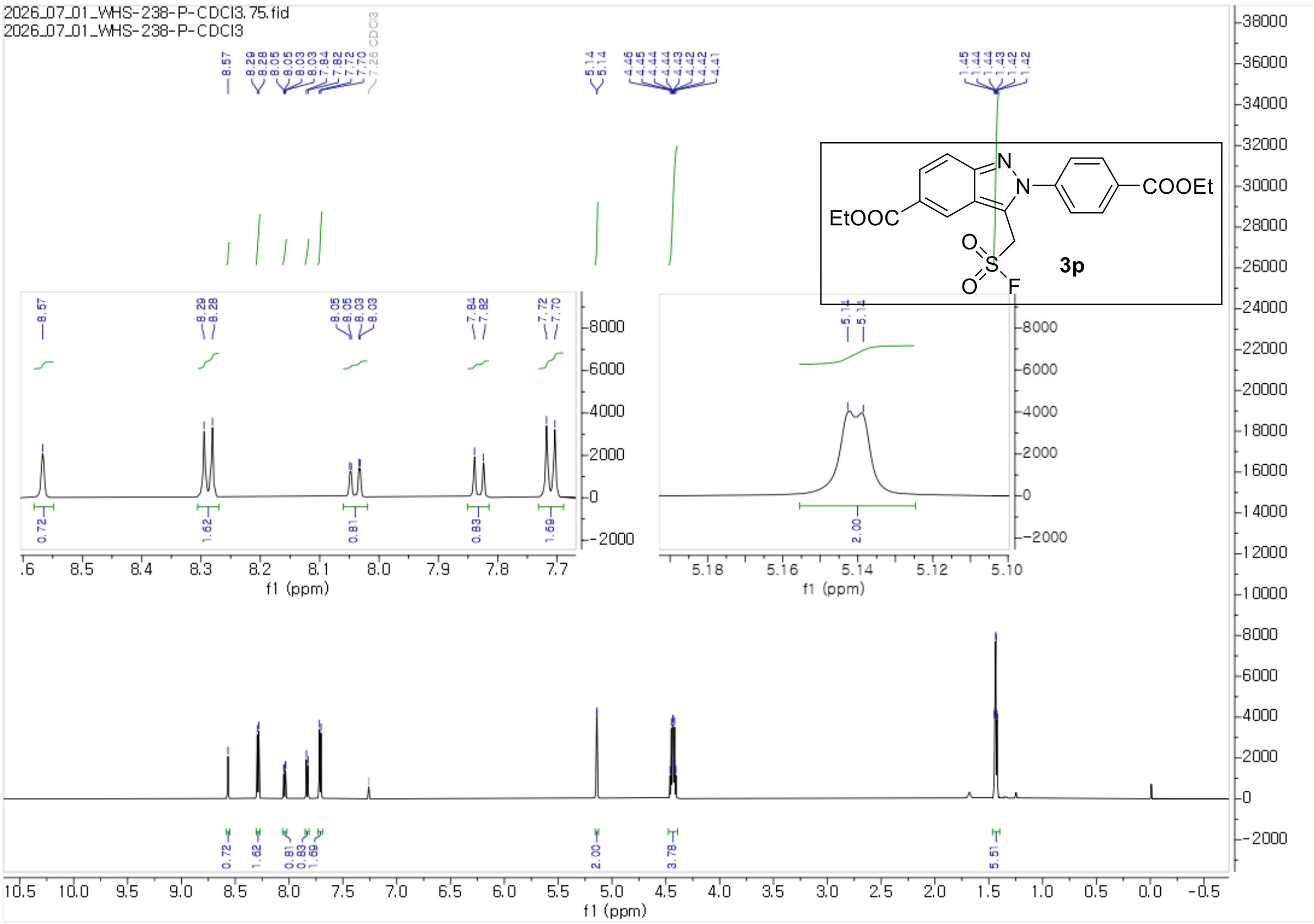
2026_07_01_WHS-238-P-CDCl3.75.fid
2026_07_01_WHS-238-P-CDCl3
EtOOC
COOEt
3p
f1 (ppm)

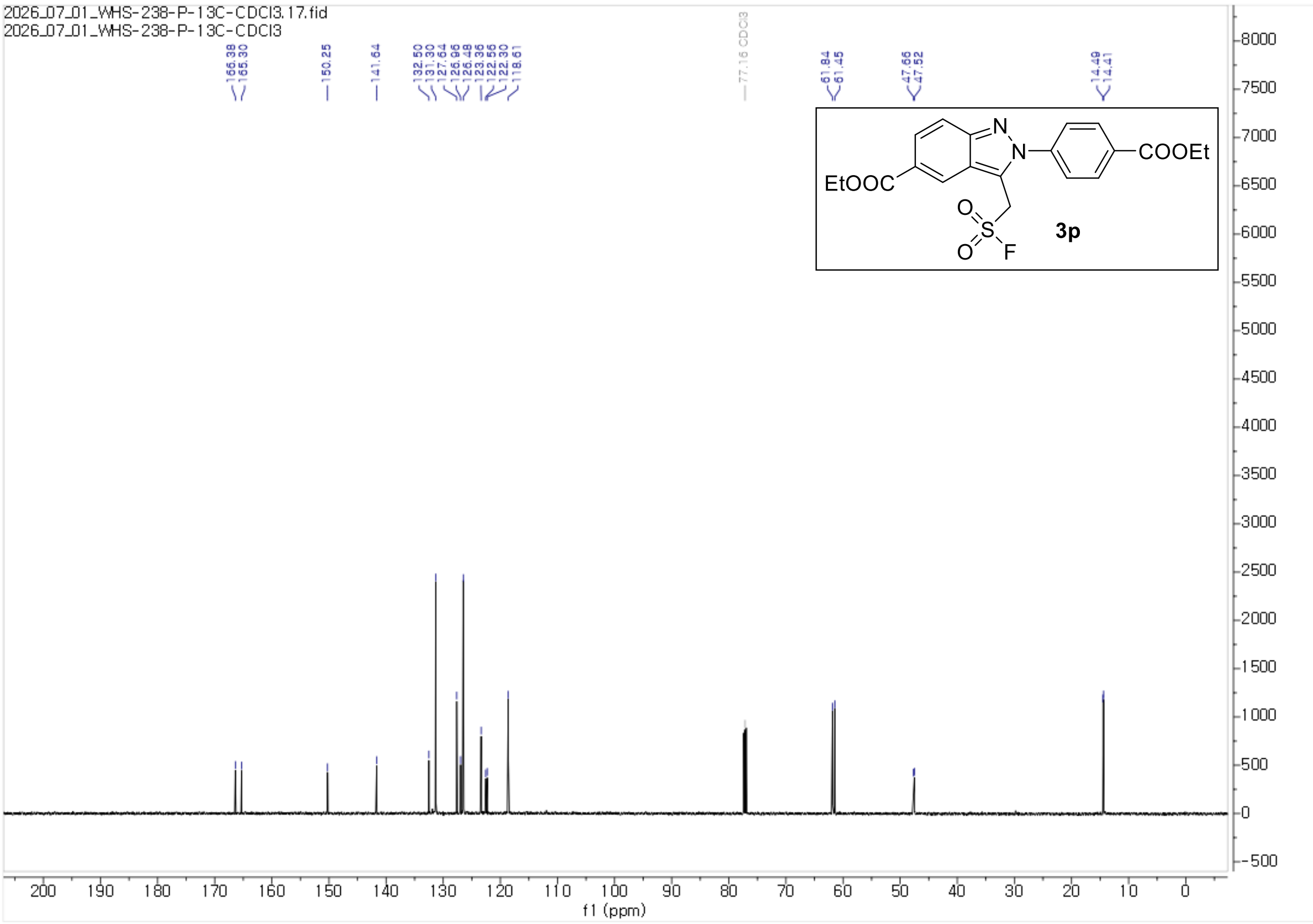
2026_07_01_WHS-238-P-13C-CDCl3.17.fid
2026_07_01_WHS-238-P-13C-CDCl3
EtOOC
COOEt
3p
f1 (ppm)

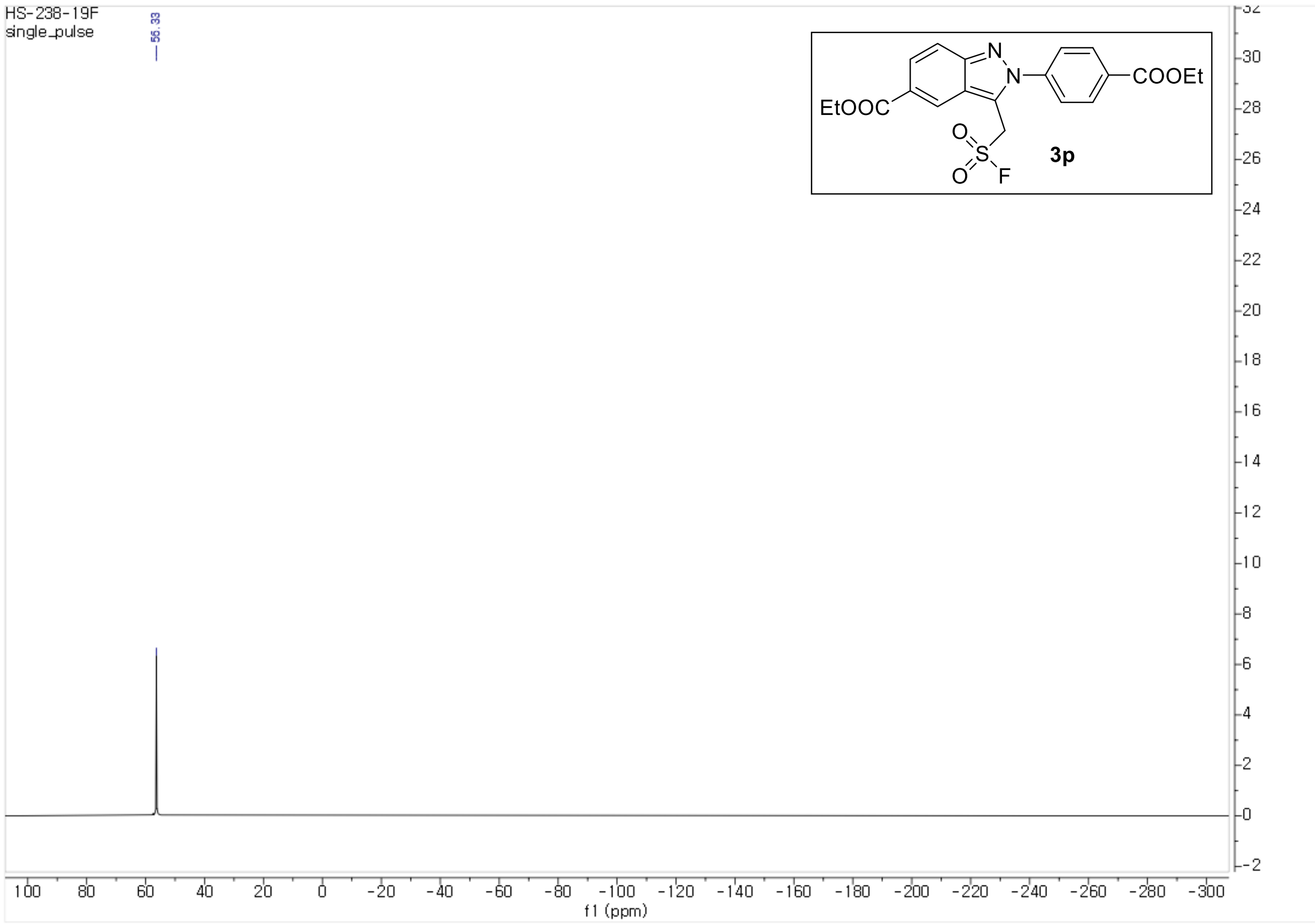
HS-238-19F
single_pulse
55.33
EtOOC
N
N
COOEt
O
S
O
F
3p
f1 (ppm)

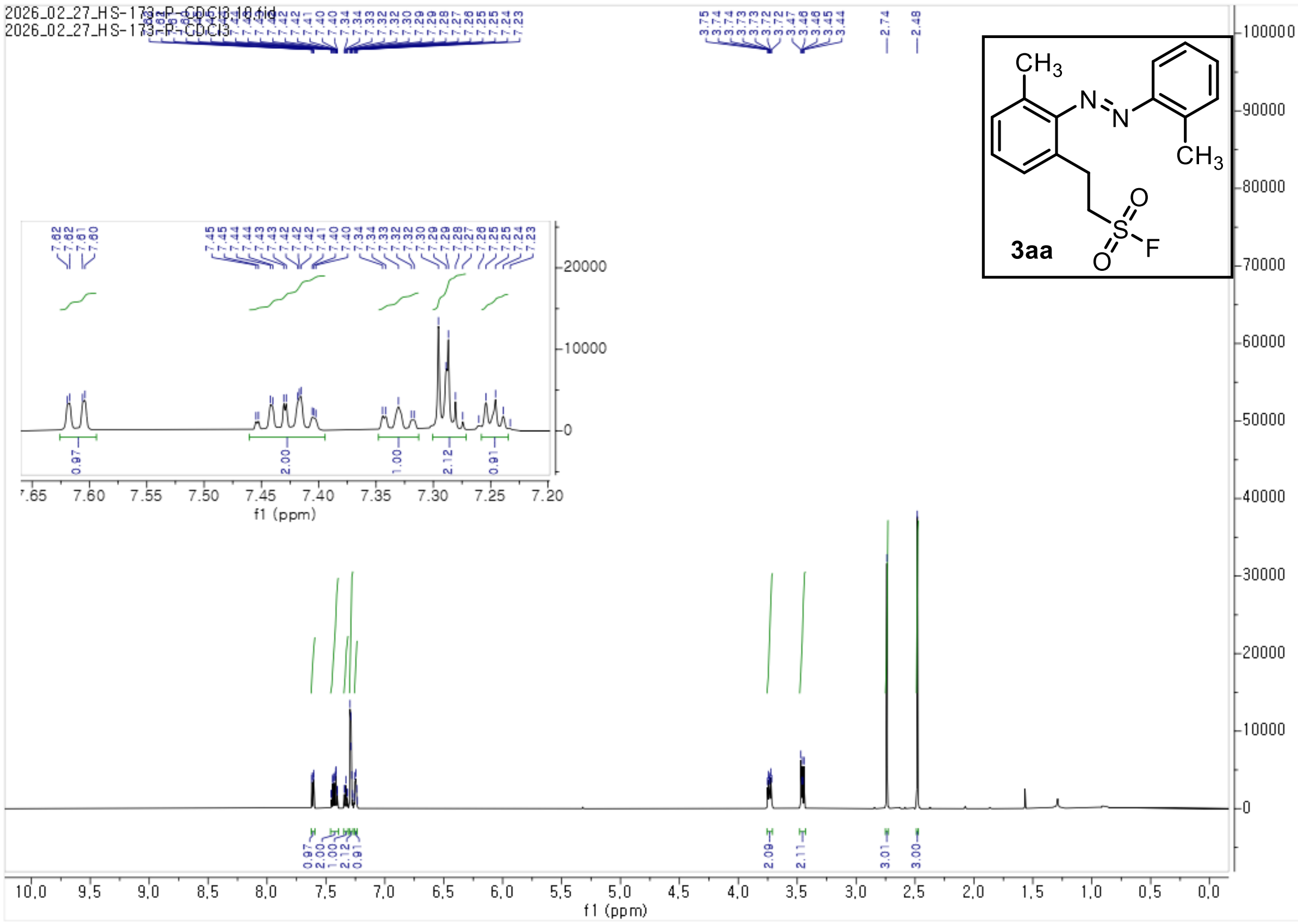
2026_02_27_HS-173-P-CDCl3.10.fid
2026_02_27_HS-173-P-CDCl3
3aa
f1 (ppm)

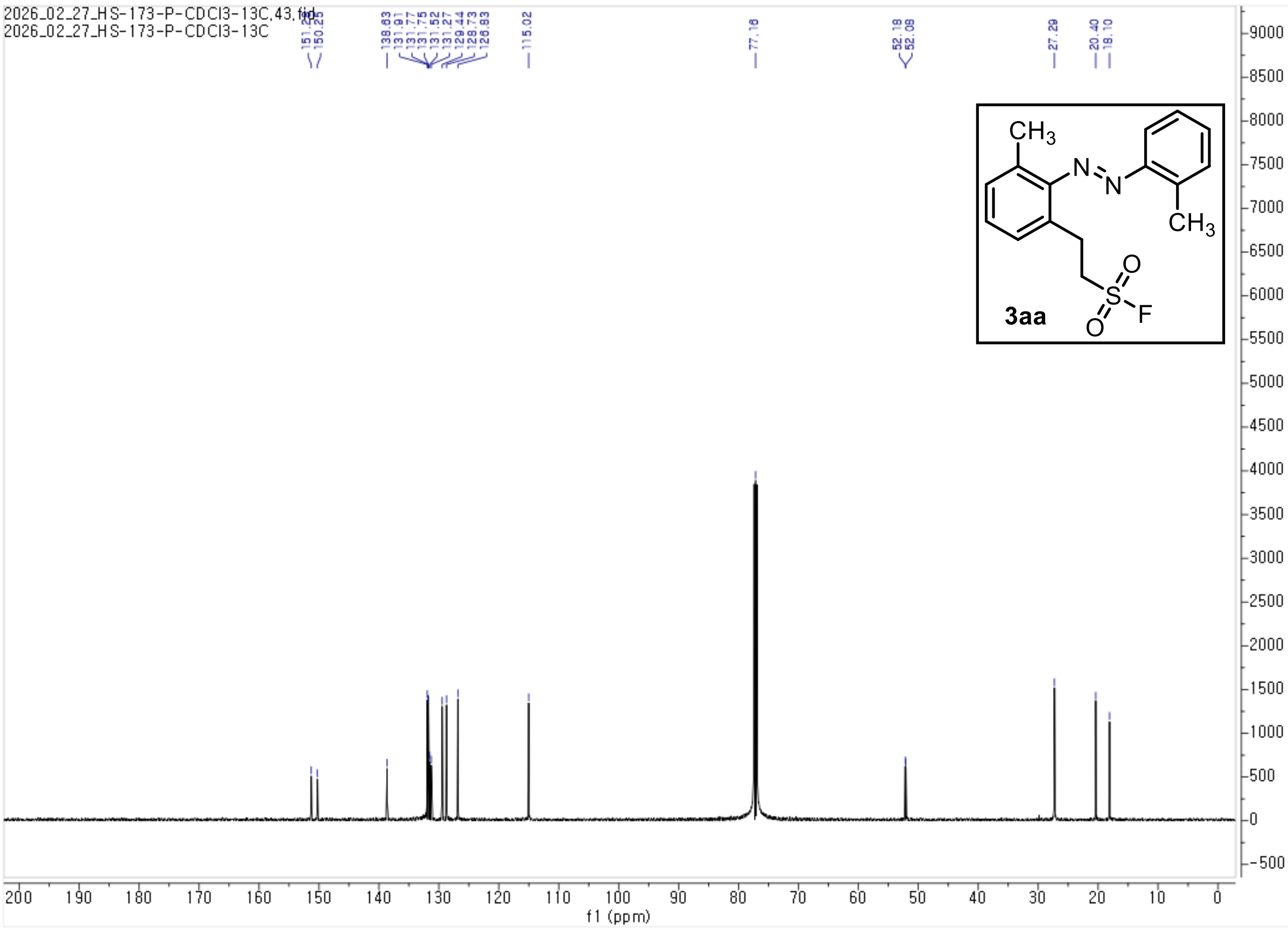
2026_02_27_HS-173-P-CDCl3-13C.43.fid
2026_02_27_HS-173-P-CDCl3-13C
3aa
f1 (ppm)

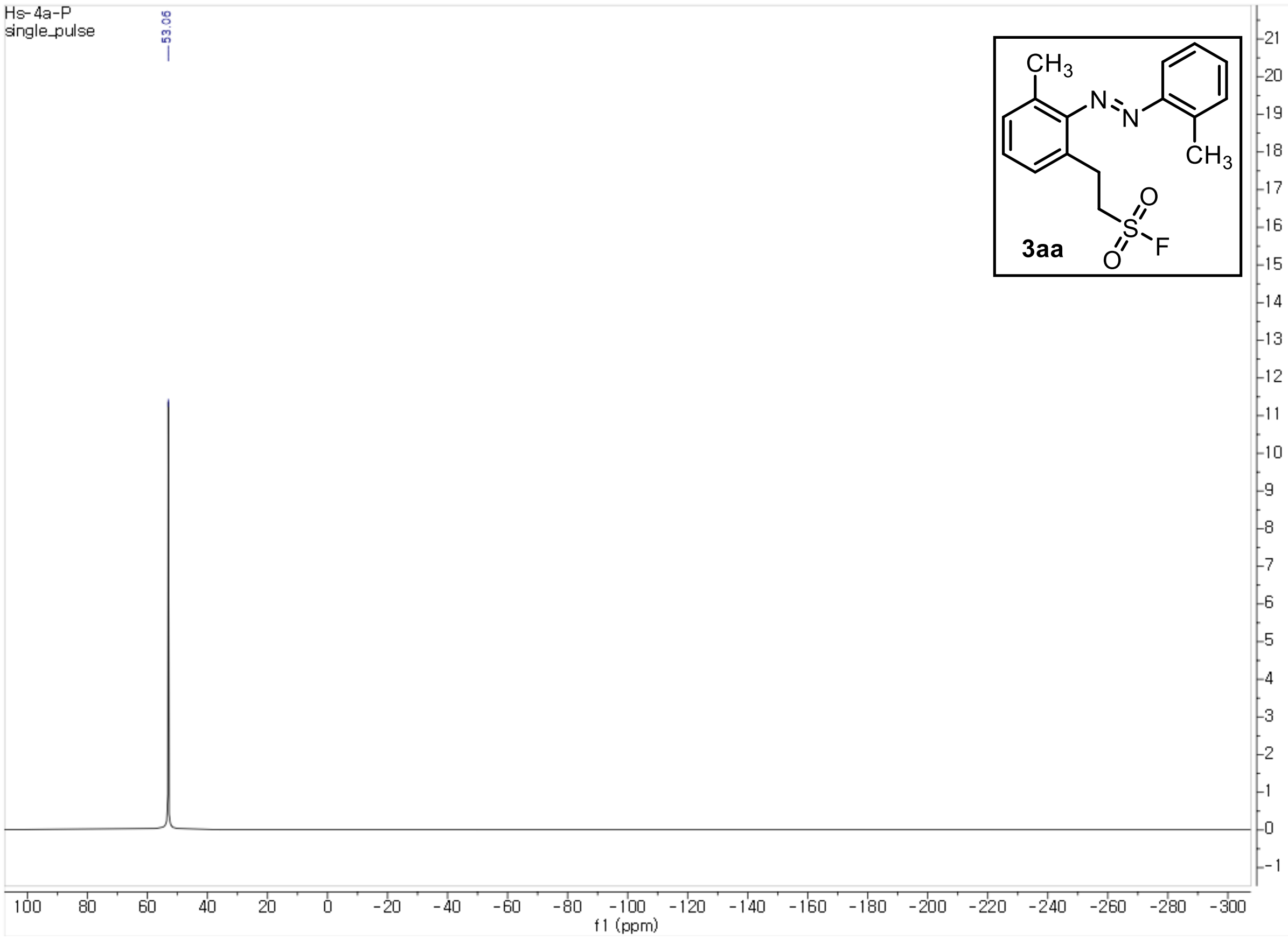
Hs-4a-P
single_pulse
53.05
3aa
CH3
N
N
CH3
S
O
O
F
f1 (ppm)

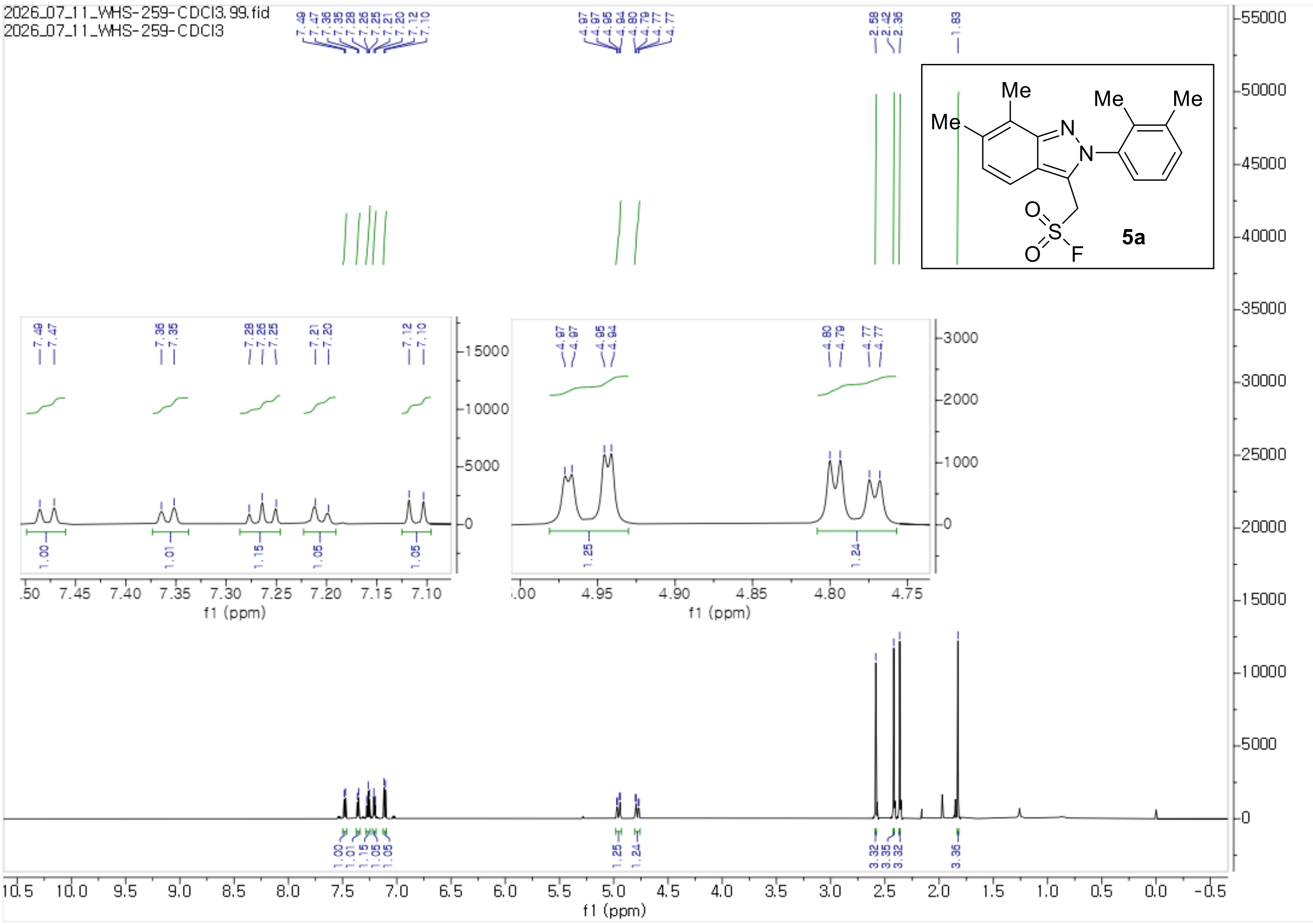
2026_07_11_WHS-259-CDCl3.99.fid
2026_07_11_WHS-259-CDCl3
5a
f1 (ppm)

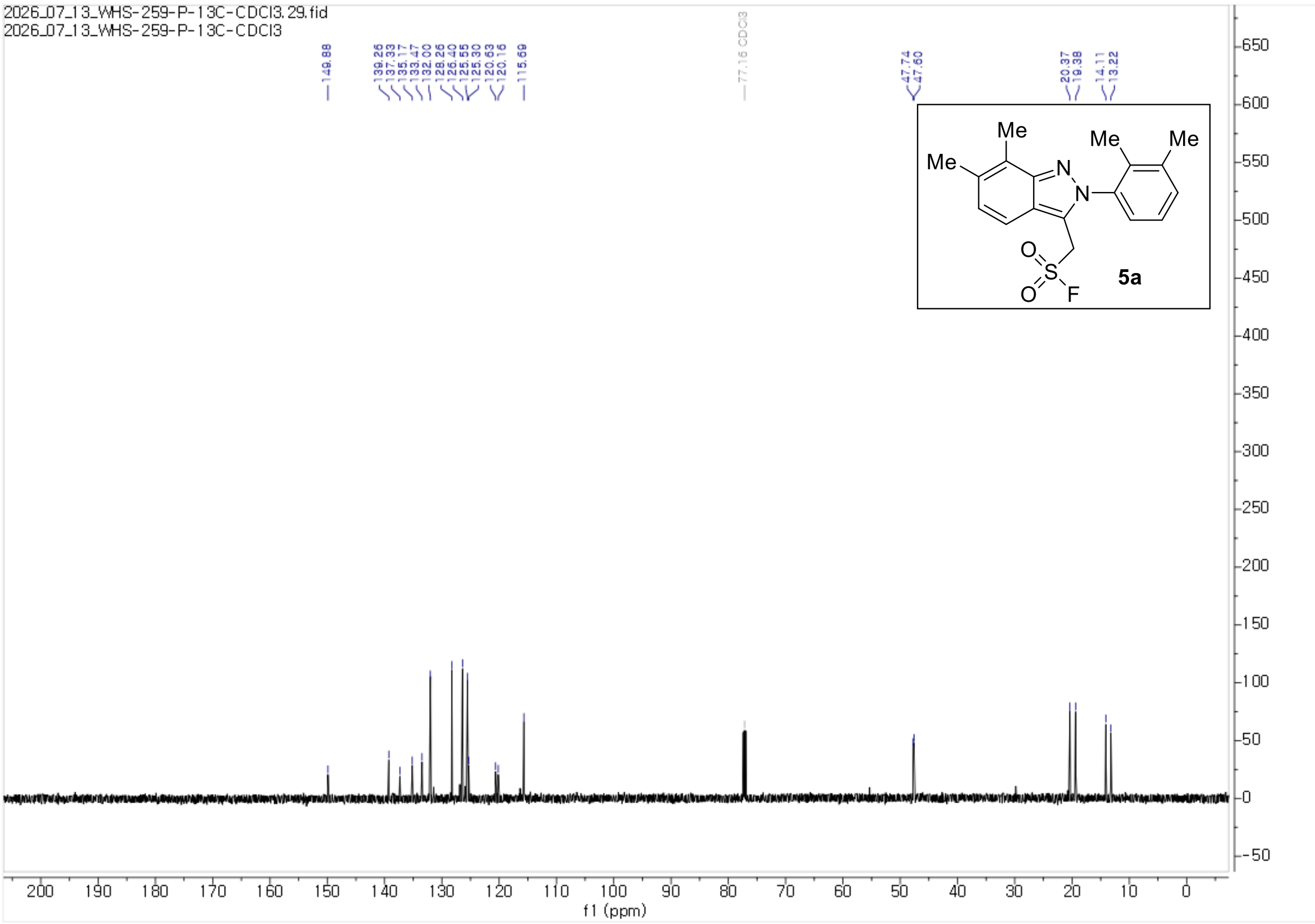
2026_07_13_WHS-259-P-13C-CDCl3.29.fid
2026_07_13_WHS-259-P-13C-CDCl3
5a
f1 (ppm)

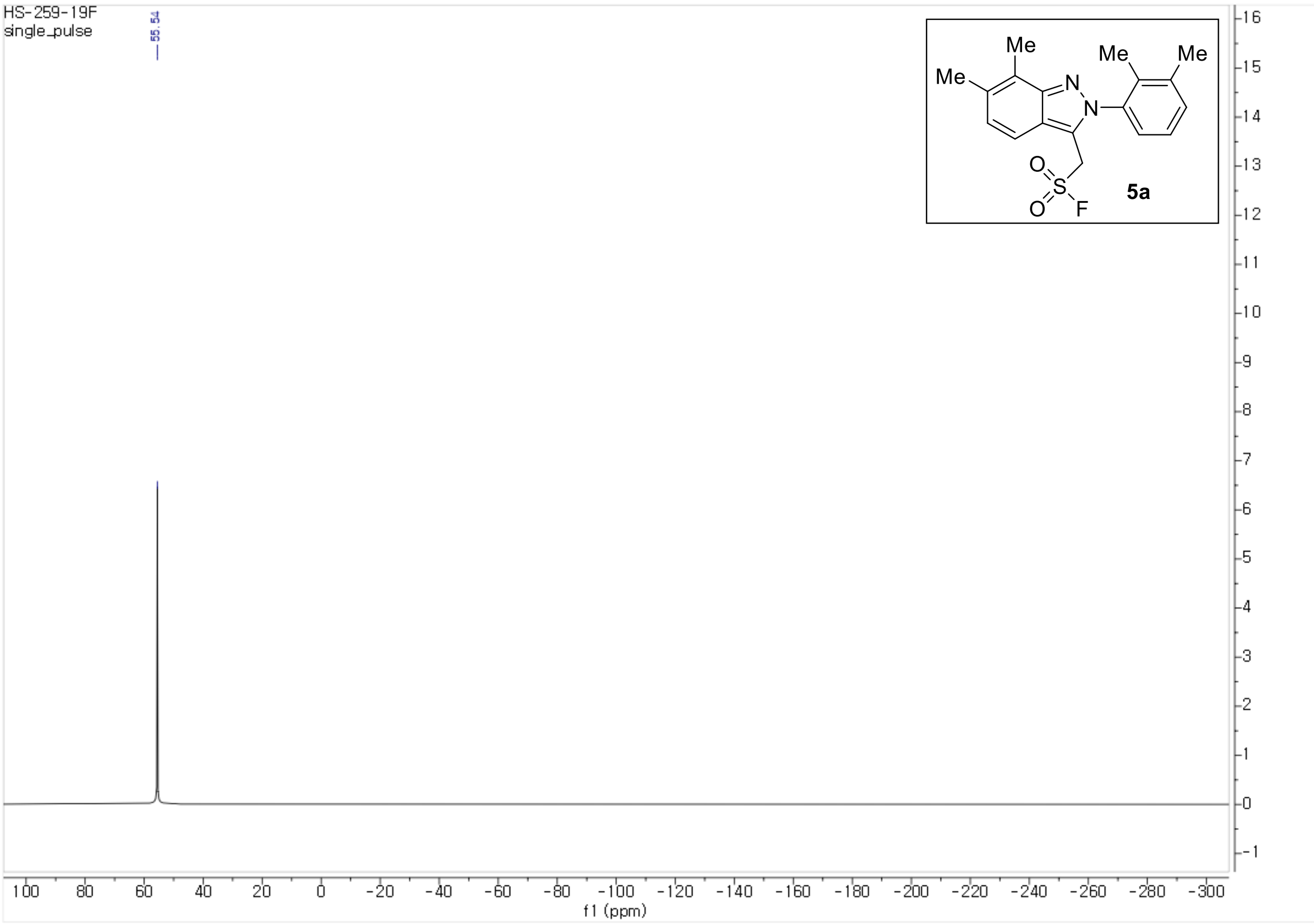
HS-259-19F
single_pulse
55.54
Me
Me
Me
Me
N
N
O
S
O
F
5a
100 80 60 40 20 0 -20 -40 -60 -80 -100 -120 -140 -160 -180 -200 -220 -240 -260 -280 -300
f1 (ppm)
16 15 14 13 12 11 10 9 8 7 6 5 4 3 2 1 0 -1

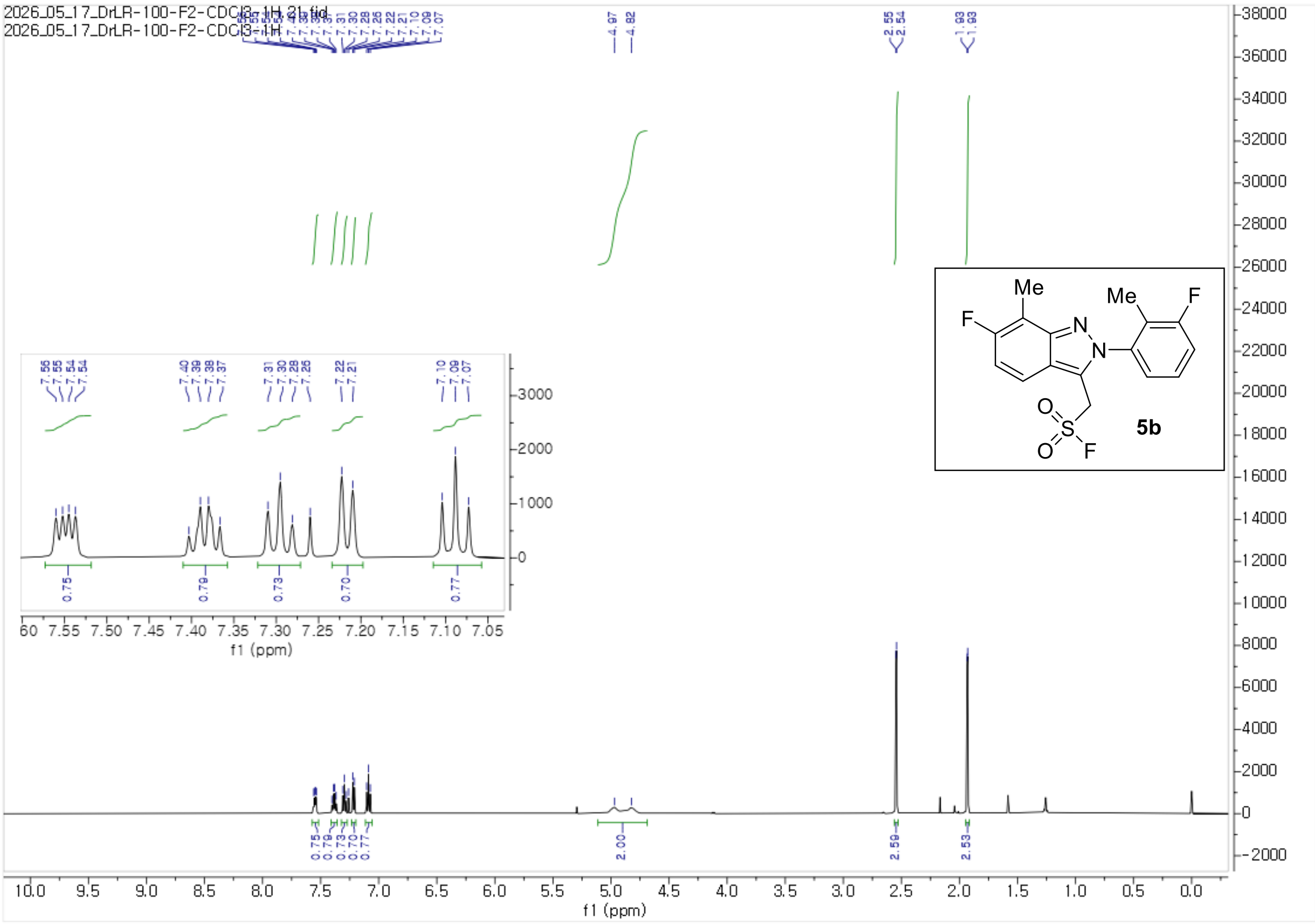
2026_05_17_DrLR-100-F2-CDCl3-1H.21.fid
2026_05_17_DrLR-100-F2-CDCl3-1H
5b
f1 (ppm)

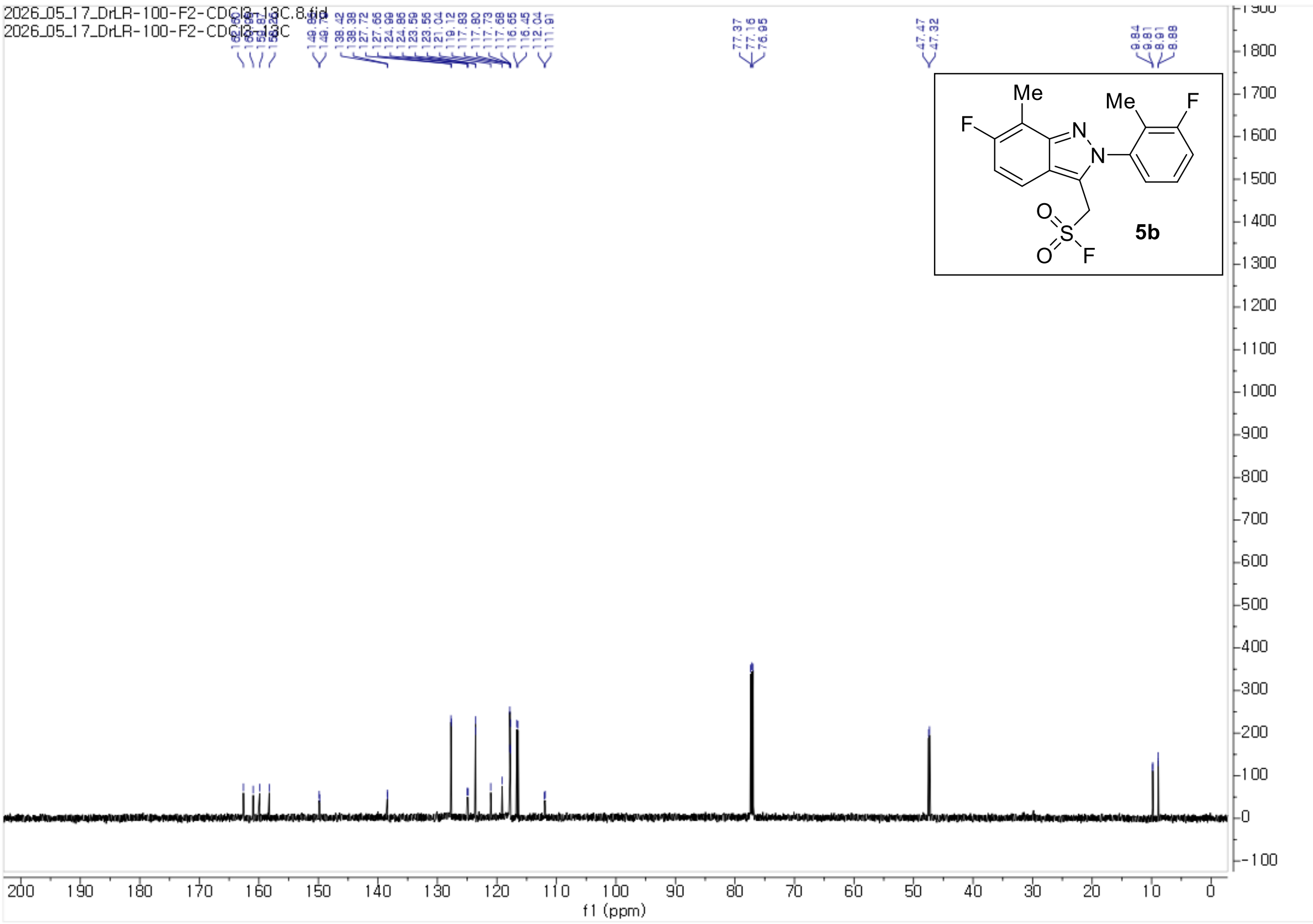
2026_05_17_DrLR-100-F2-CDCl3-13C.8.fid
2026_05_17_DrLR-100-F2-CDCl3-13C
5b
f1 (ppm)

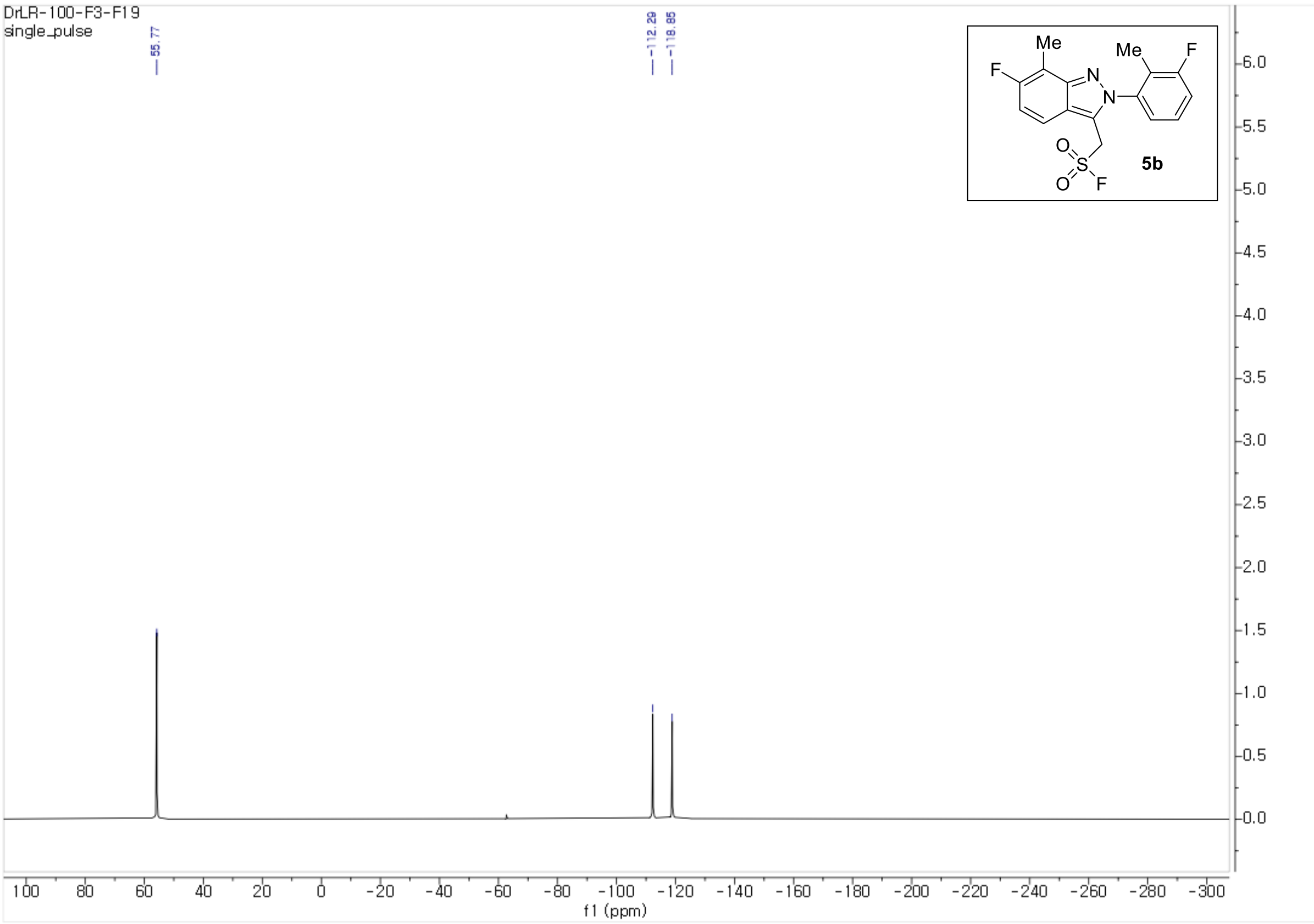
DrLR-100-F3-F19
single_pulse
55.77
-112.29
-118.85
Me
F
N
N
Me
F
O
S
O
F
5b
f1 (ppm)

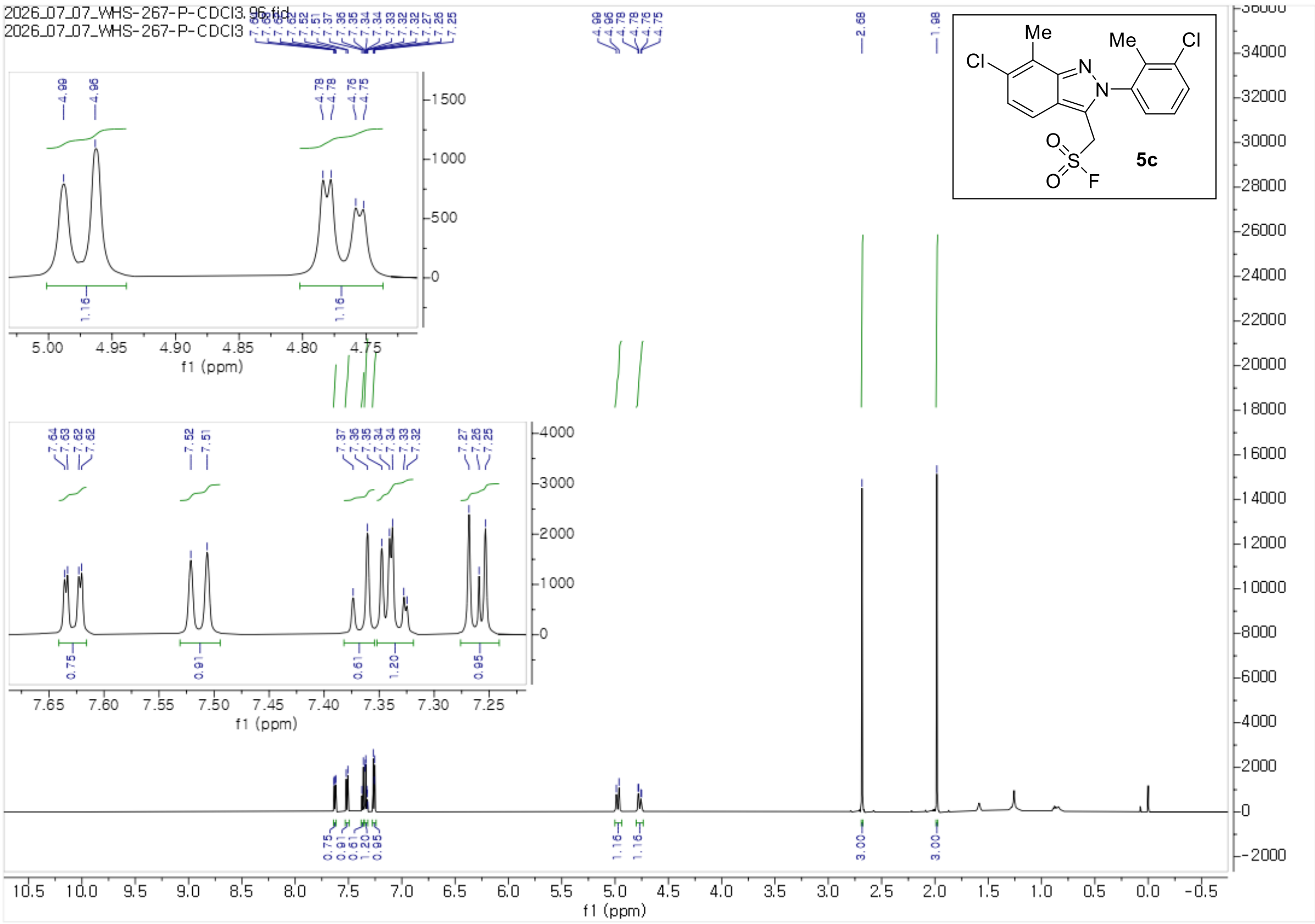

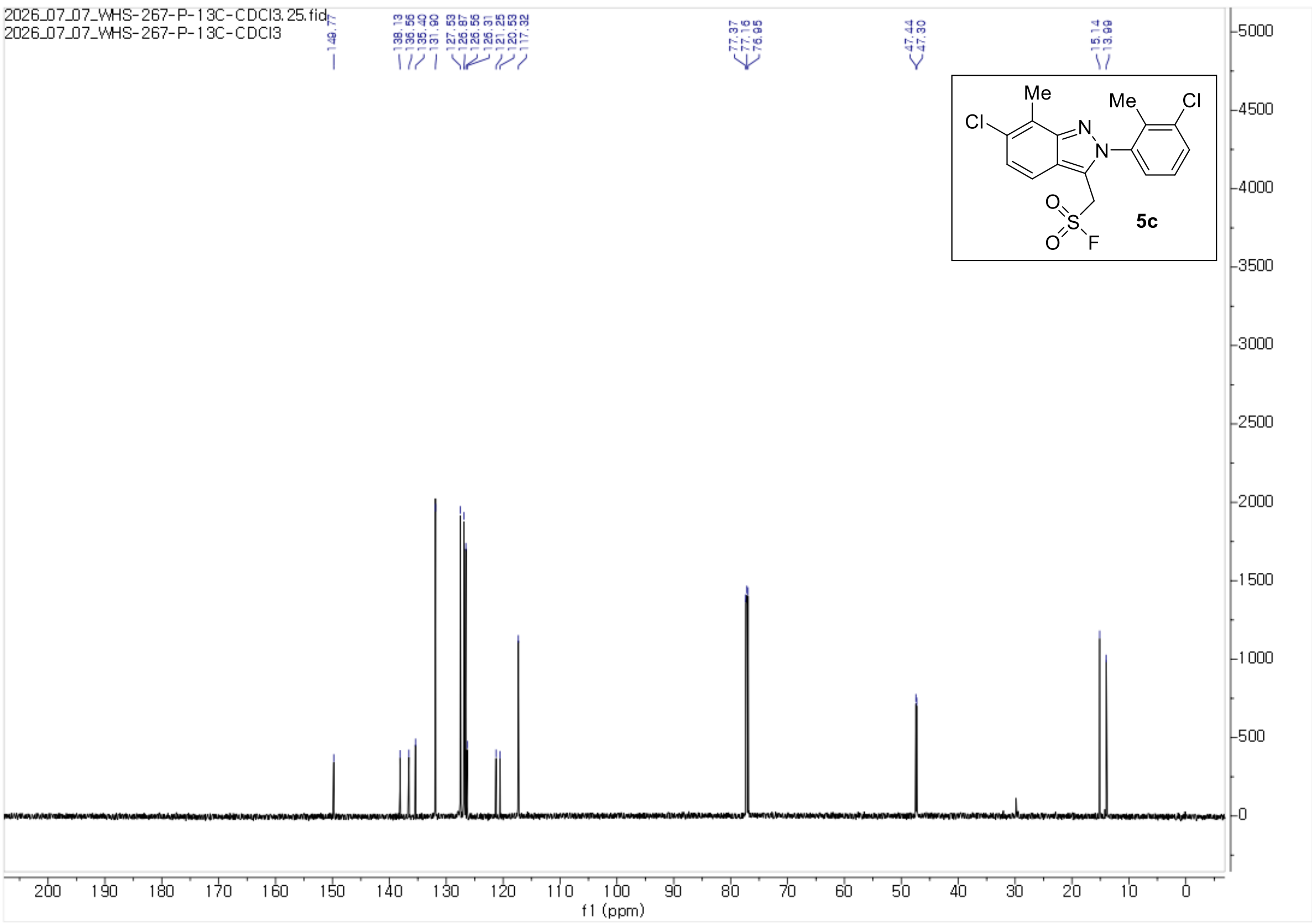

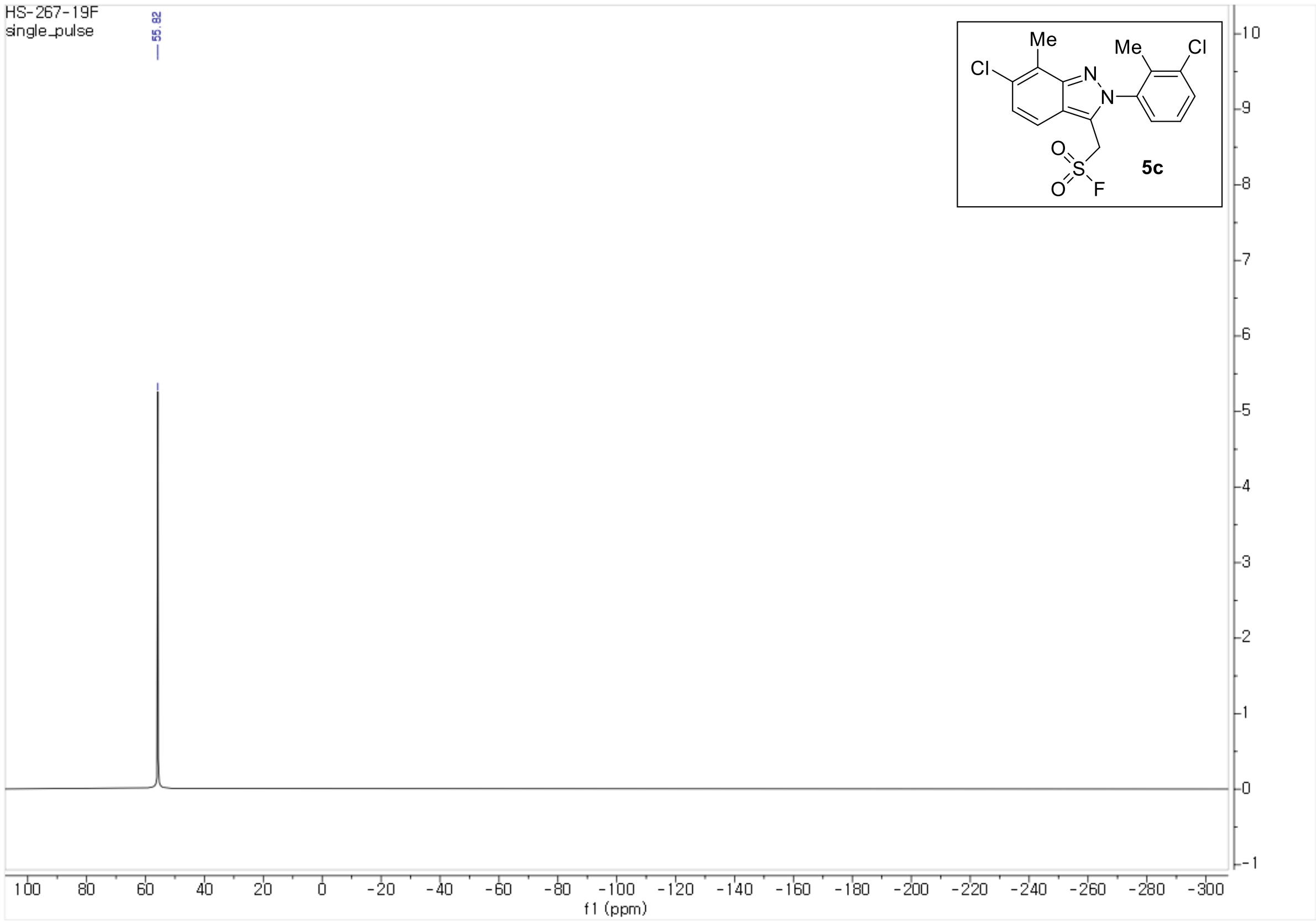
HS-267-19F
single_pulse
55.82
Me
Cl
N
N
Me
Cl
O
S
O
F
5c
f1 (ppm)

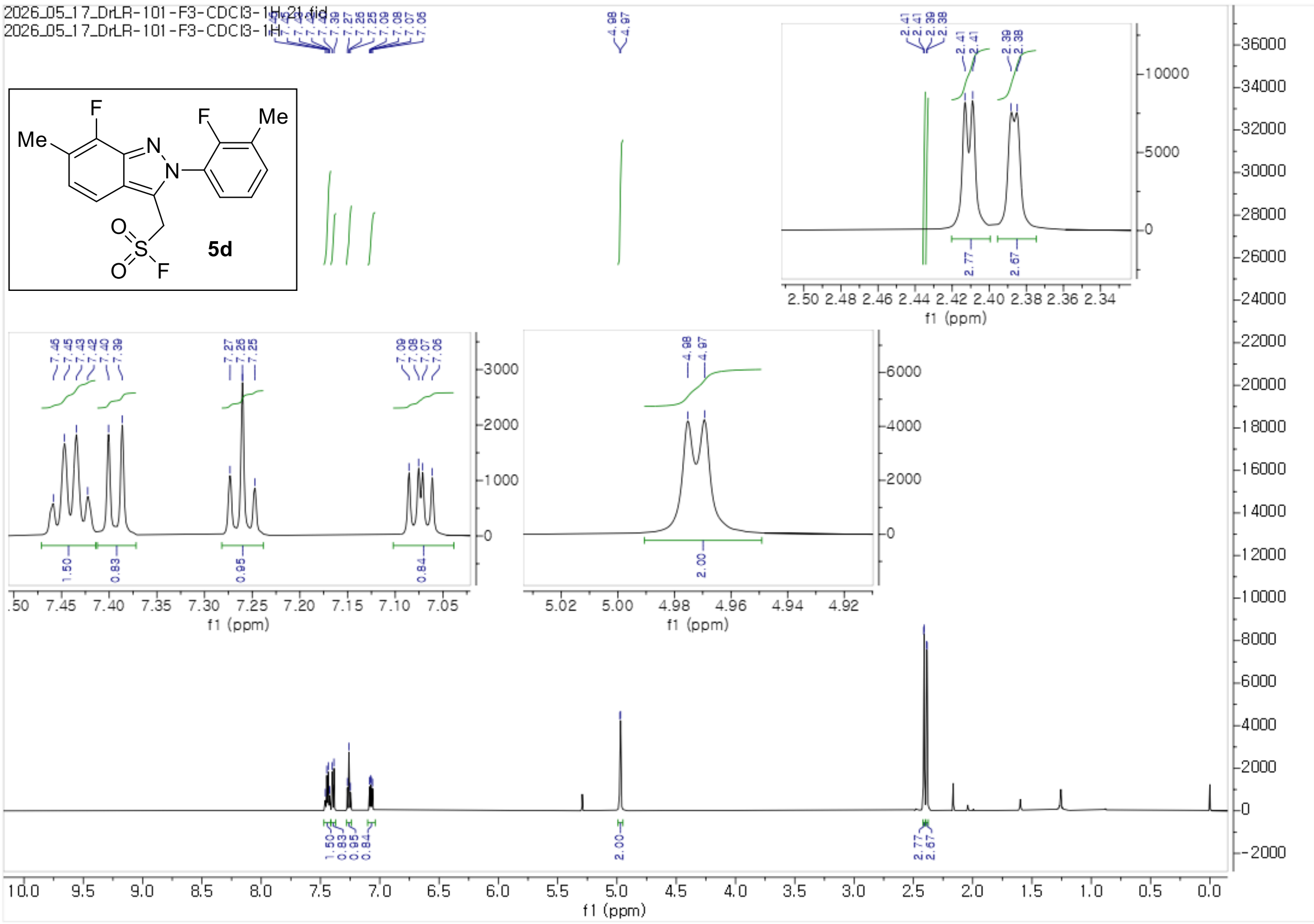

2026_05_17_DrLR-101-F3-CDCl3-1H.21.fid
2026_05_17_DrLR-101-F3-CDCl3-1H
5d
f1 (ppm)

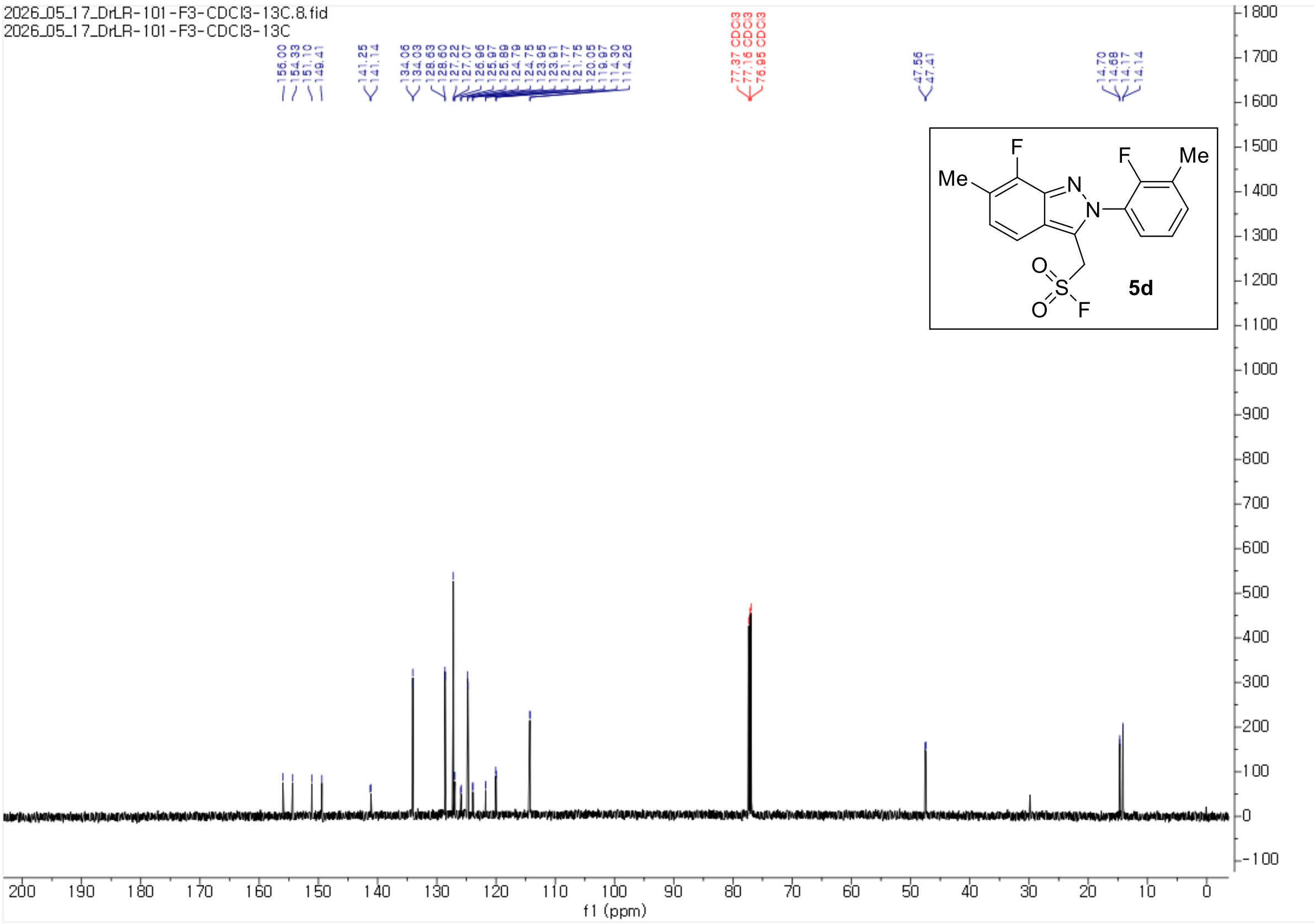

2026_05_17_DrLR-101-F3-CDCl3-13C.8.fid
2026_05_17_DrLR-101-F3-CDCl3-13C
5d
f1 (ppm)

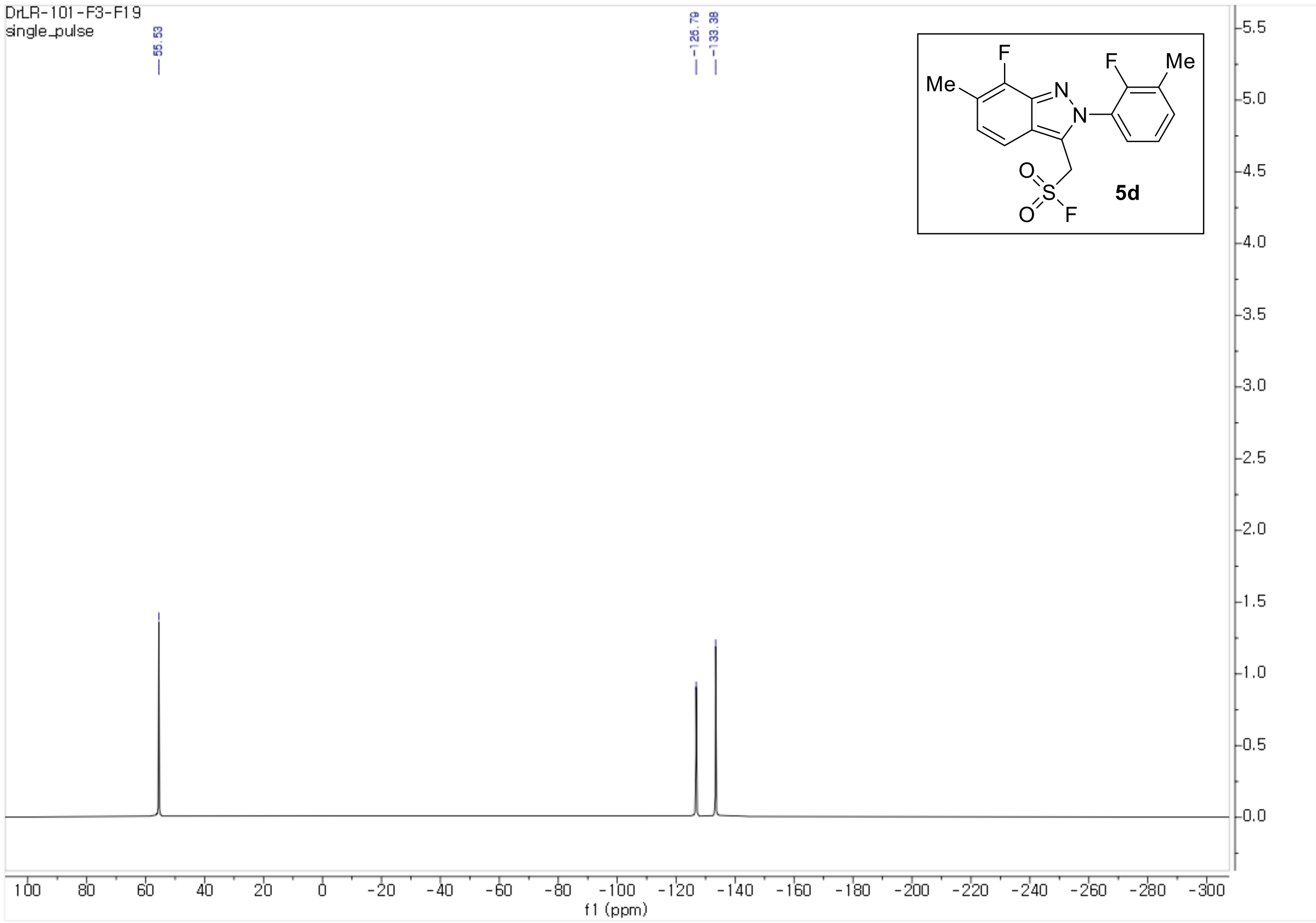
DrLR-101-F3-F19
single_pulse
55.53
-125.79
-133.38
F
Me
N
N
F
Me
O
S
O
F
5d
f1 (ppm)

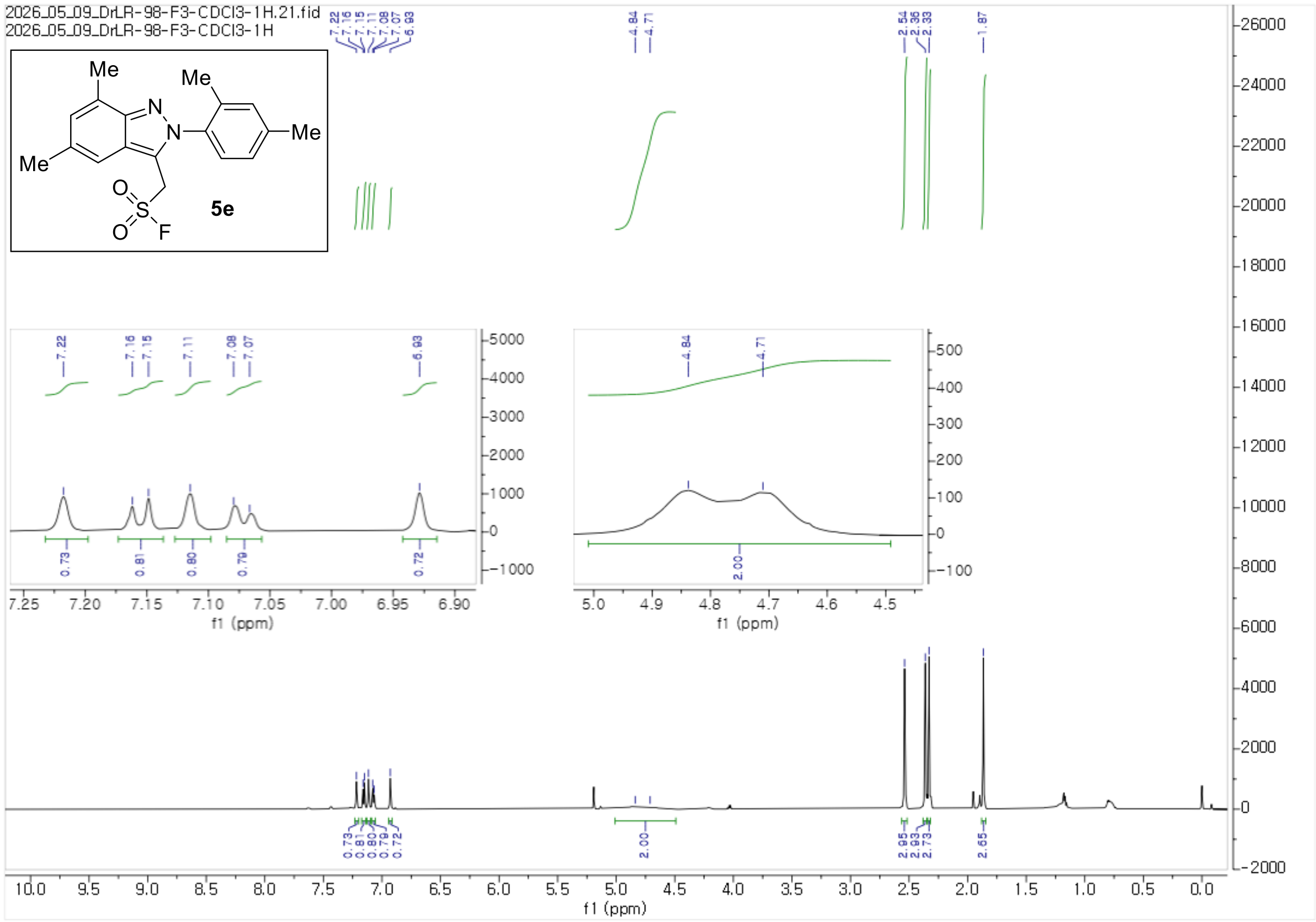

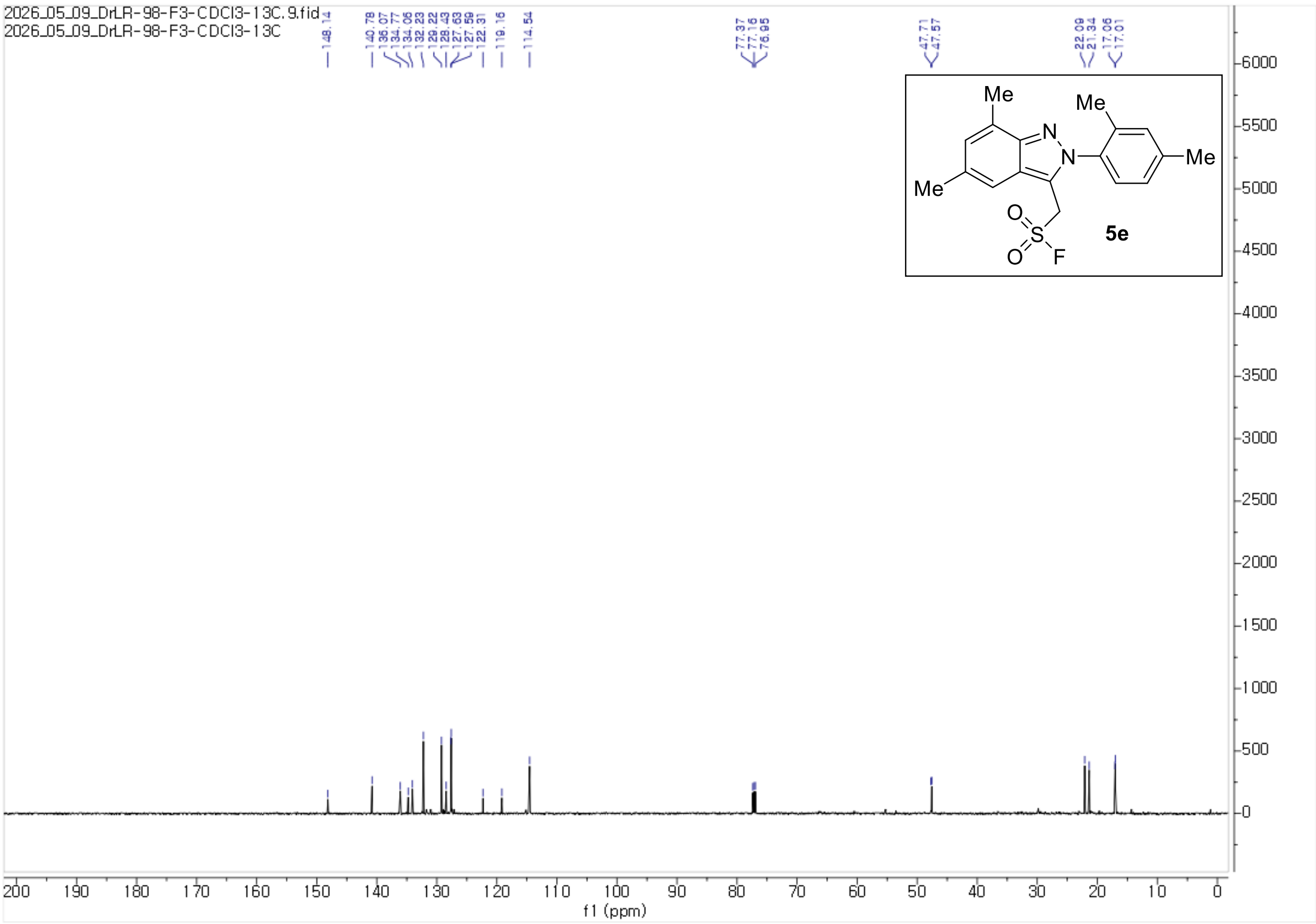

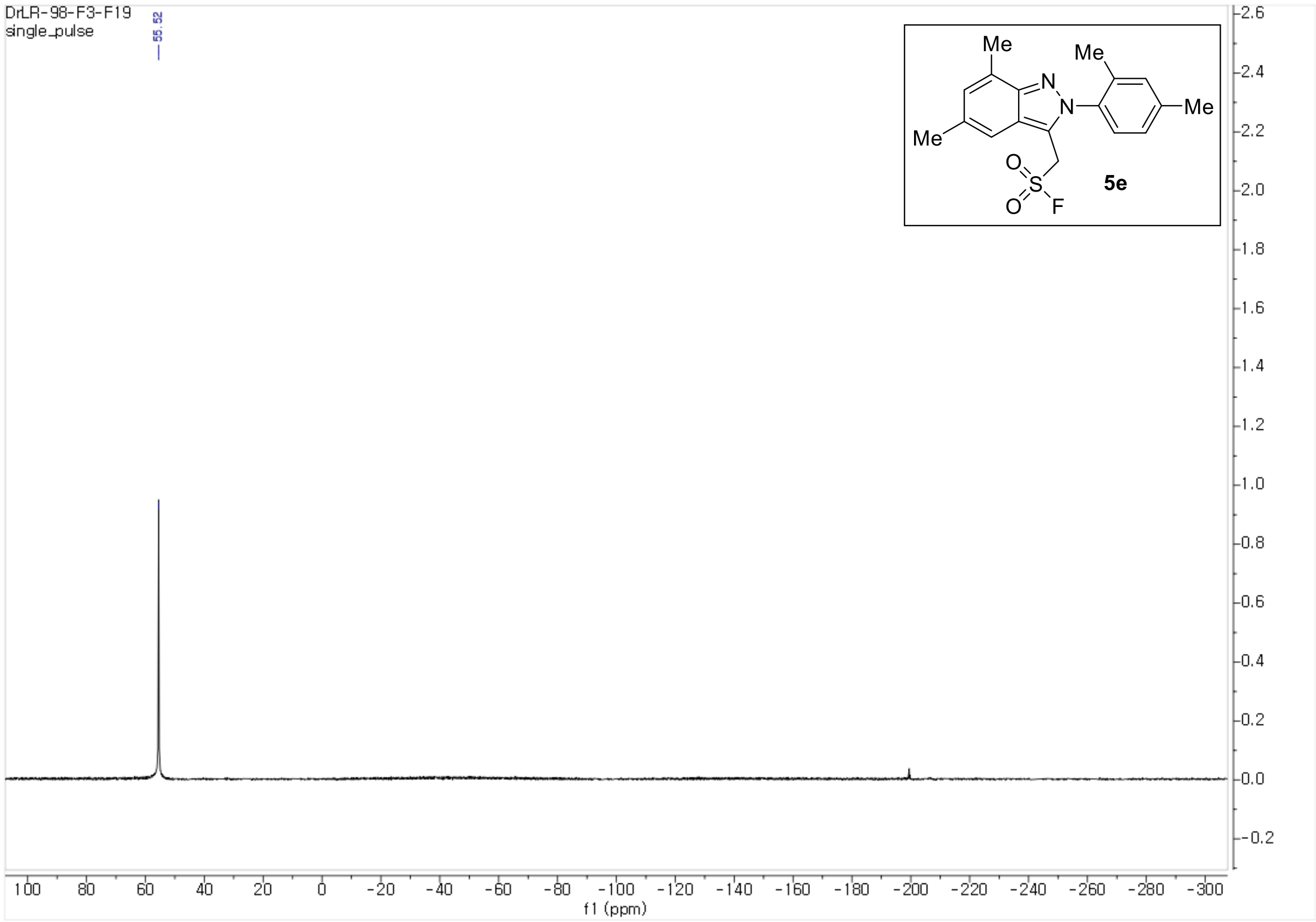
DrLR-98-F3-F19
single_pulse
55.52
Me
Me
N
N
Me
Me
O
S
O
F
5e
f1 (ppm)

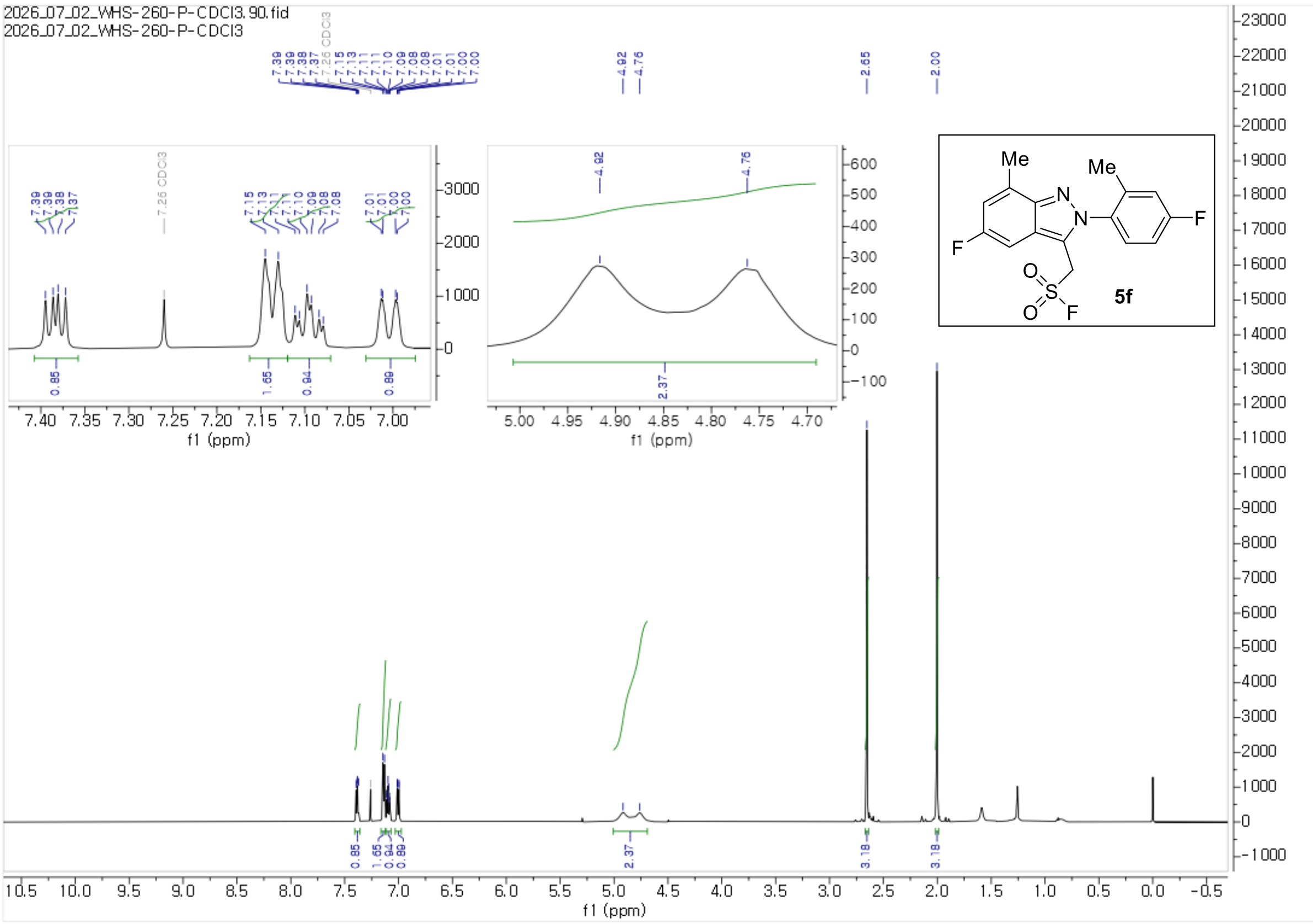
2026_07_02_WHS-260-P-CDCl3.90.fid
2026_07_02_WHS-260-P-CDCl3
5f
f1 (ppm)

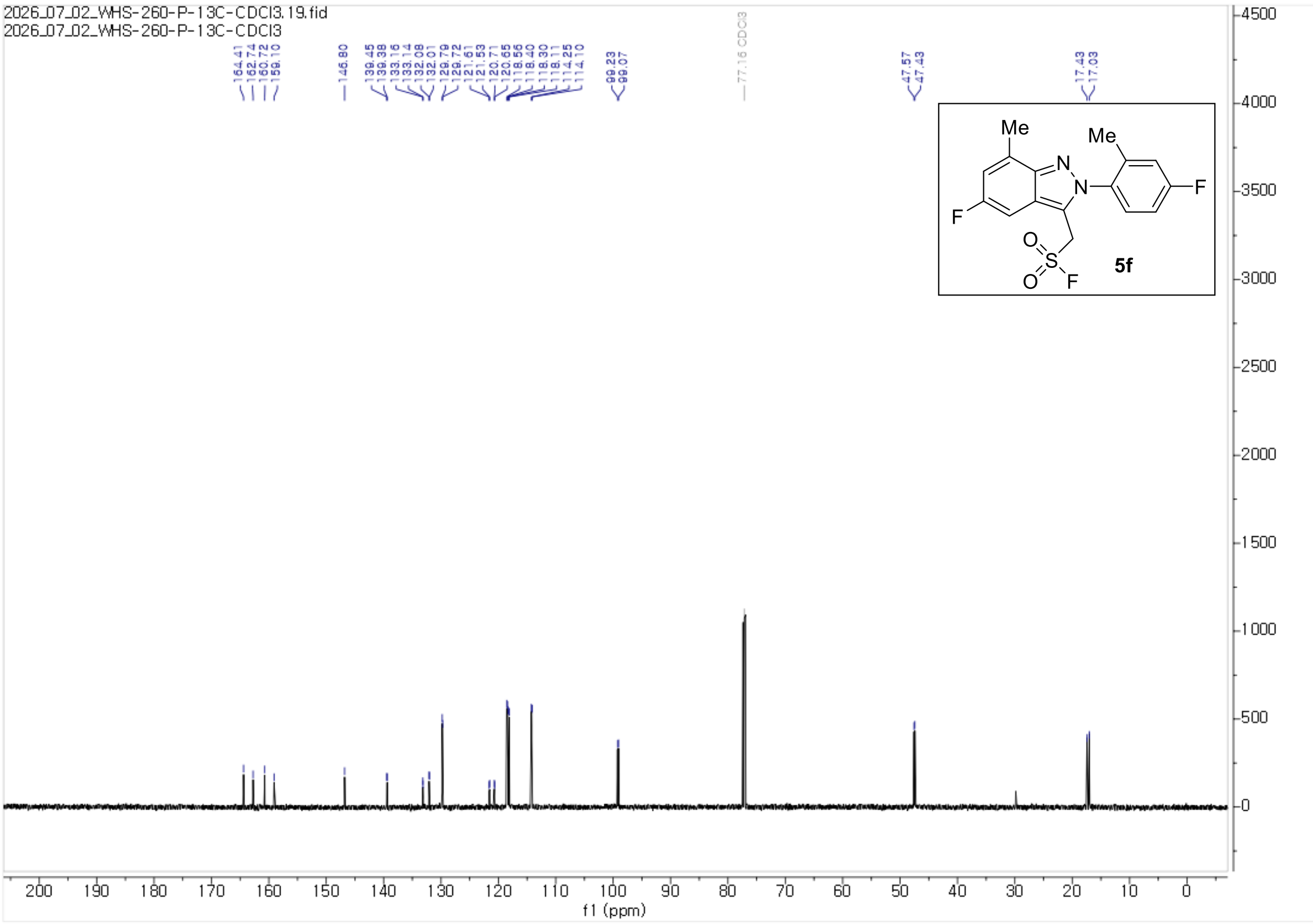
2026_07_02_WHS-260-P-13C-CDCl3.19.fid
2026_07_02_WHS-260-P-13C-CDCl3
5f
f1 (ppm)

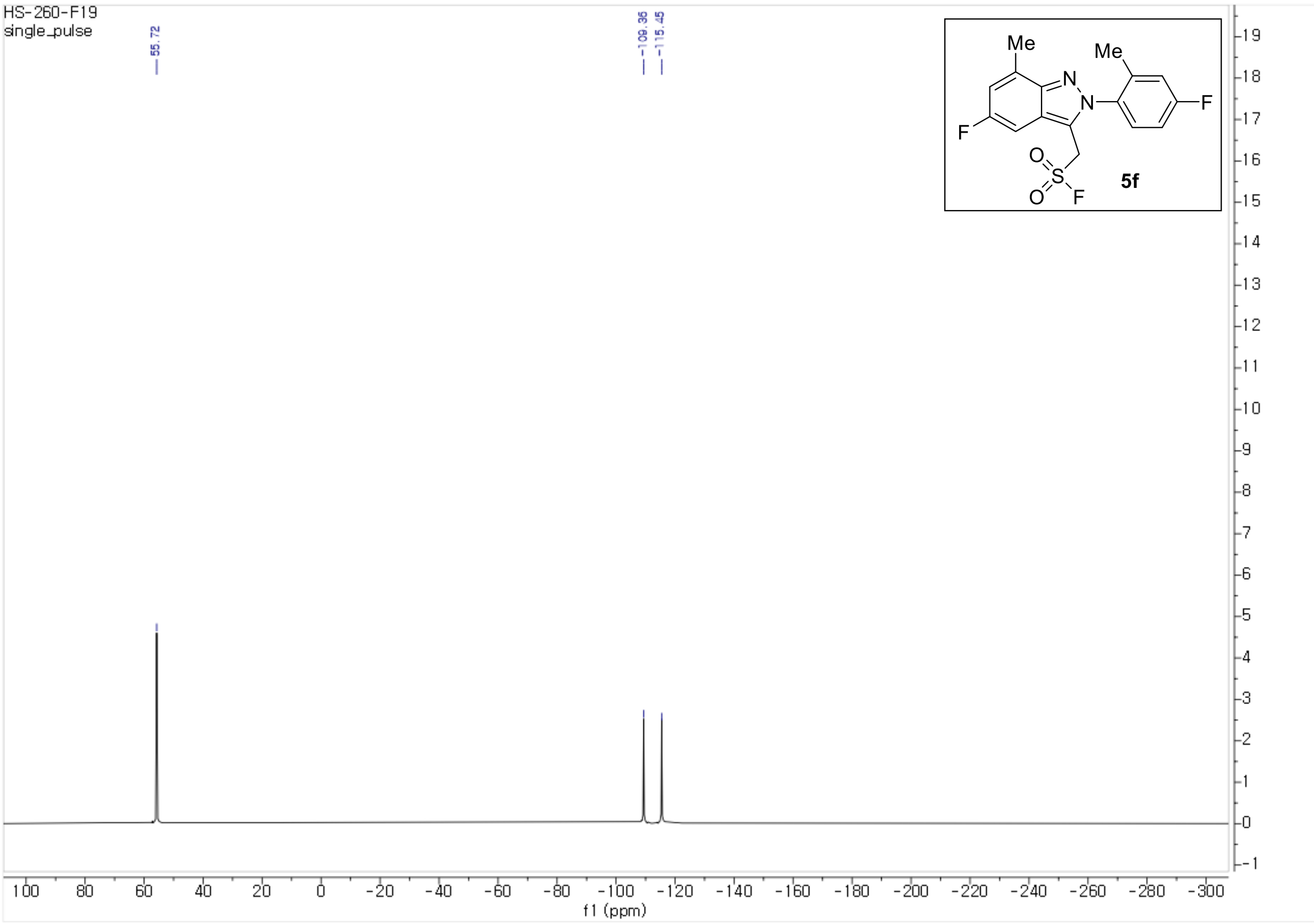
HS-260-F19
single_pulse
55.72
-109.36
-115.45
Me
Me
N
N
F
F
O
S
O
F
5f
19
18
17
16
15
14
13
12
11
10
9
8
7
6
5
4
3
2
1
0
-1
100
80
60
40
20
0
-20
-40
-60
-80
-100
-120
-140
-160
-180
-200
-220
-240
-260
-280
-300
f1 (ppm)

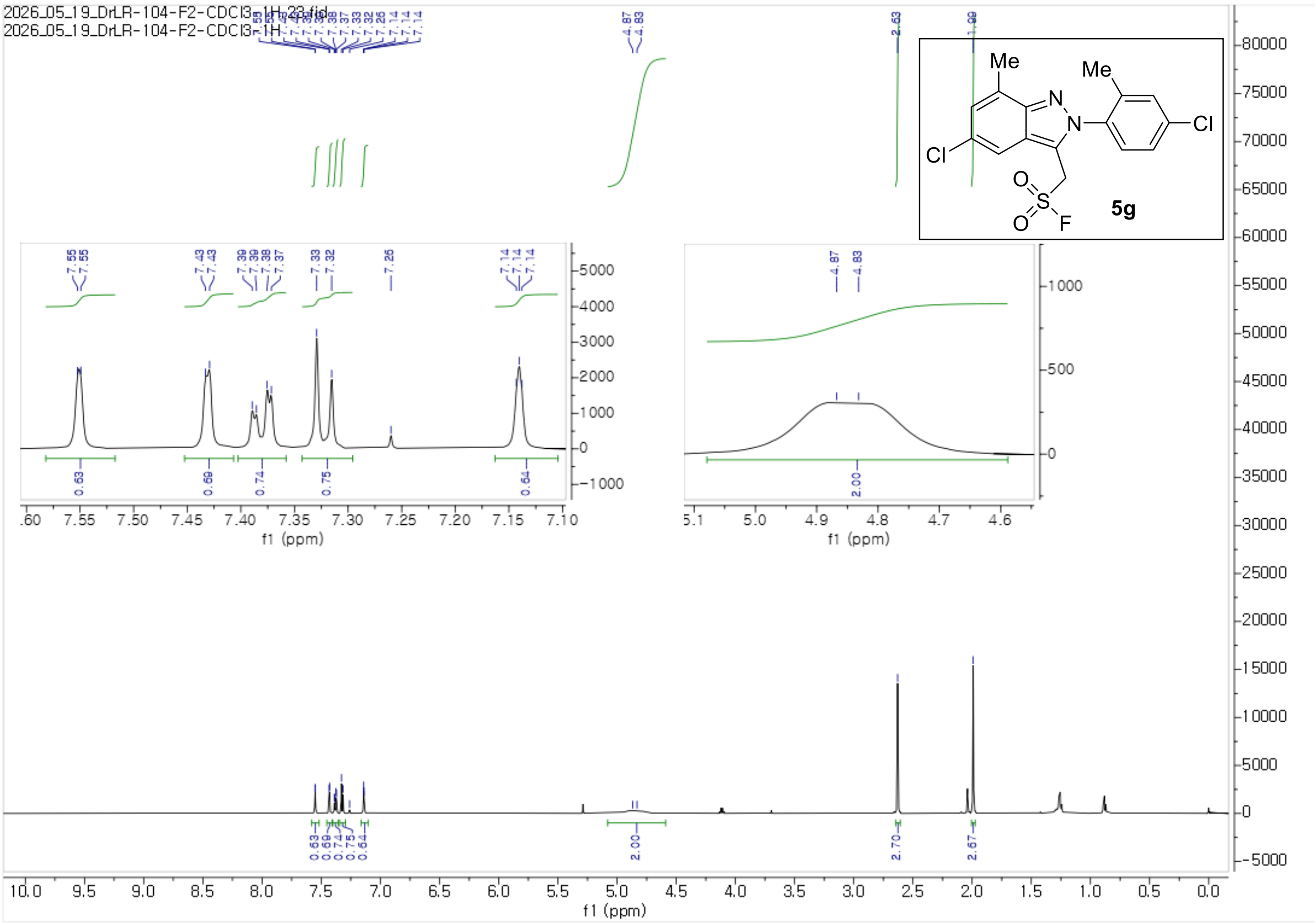

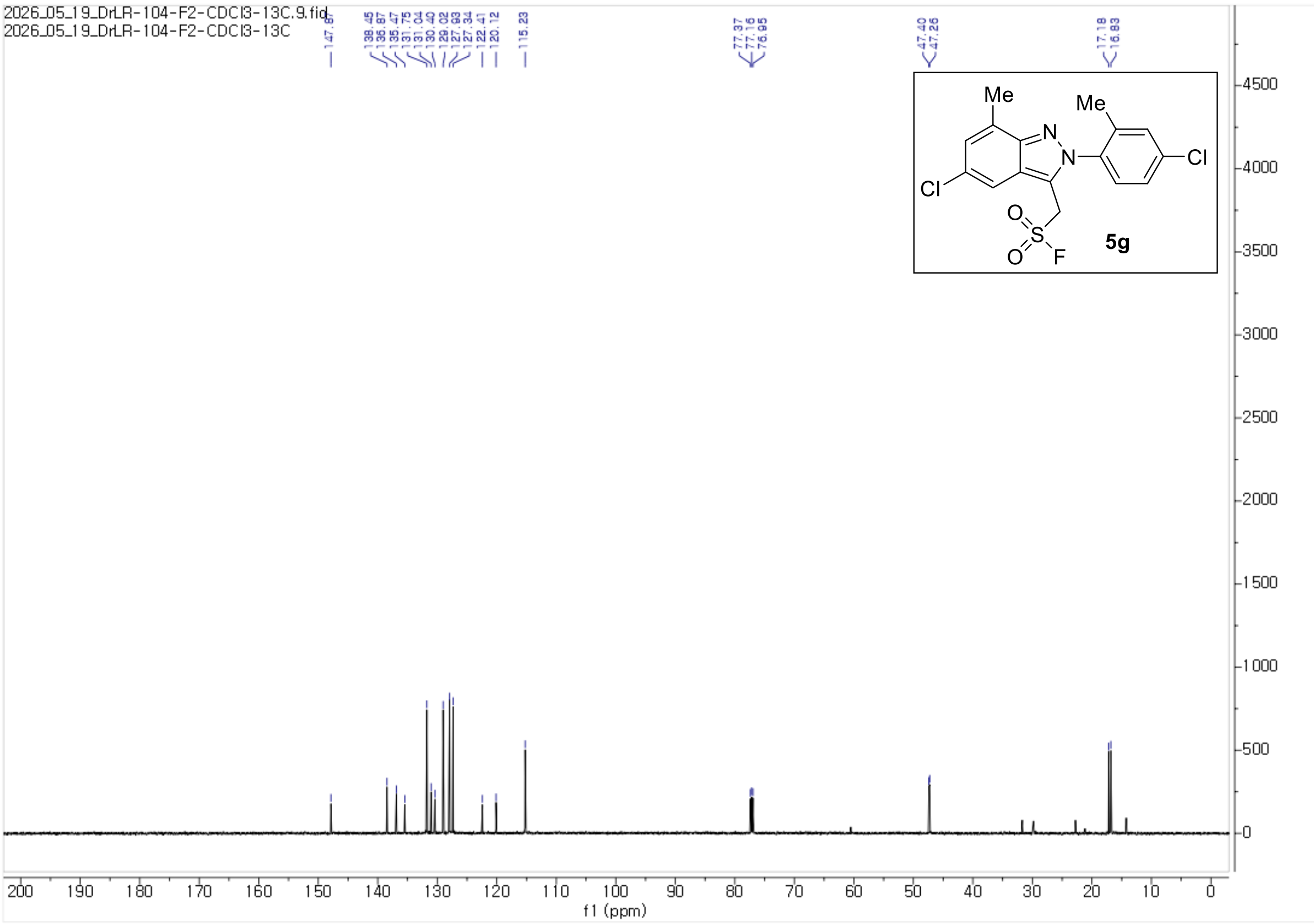

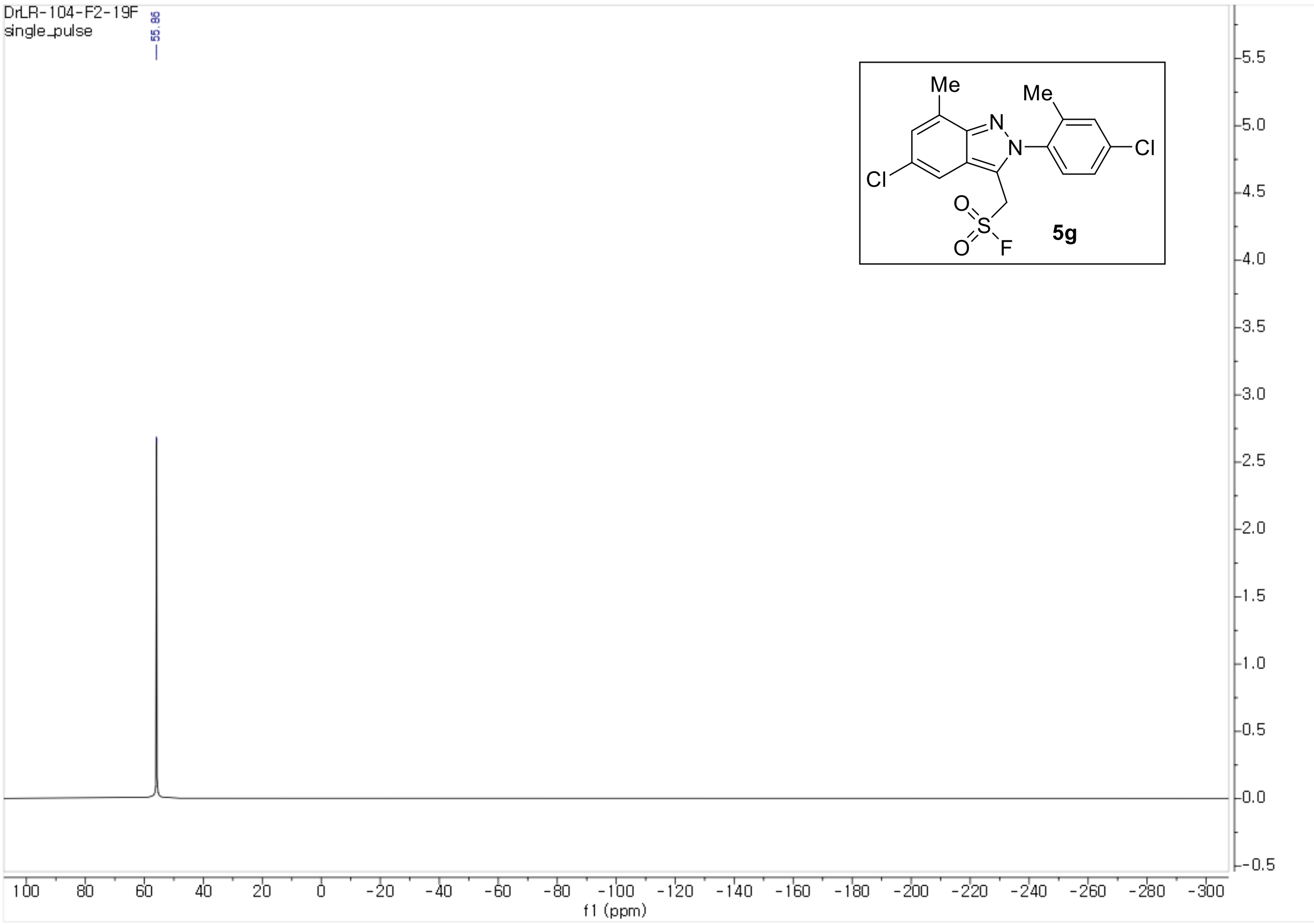
DrLR-104-F2-19F
single_pulse
55.86
Me
Me
N
N
Cl
Cl
O
S
O
F
5g
f1 (ppm)

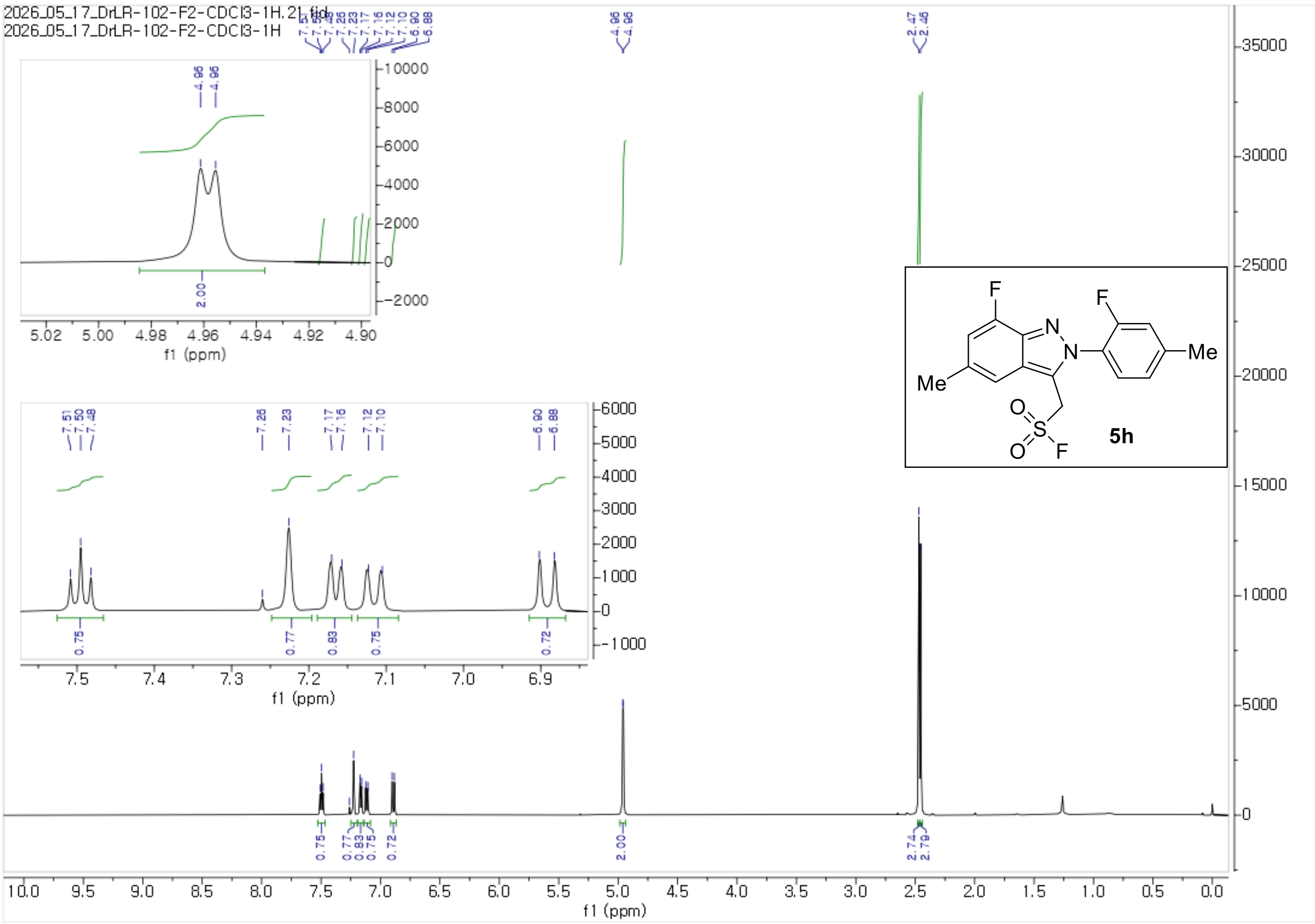
2026_05_17_DrLR-102-F2-CDCl3-1H.21.fid
2026_05_17_DrLR-102-F2-CDCl3-1H
5h
Me
F
N
N
F
Me
O
S
O
F
f1 (ppm)

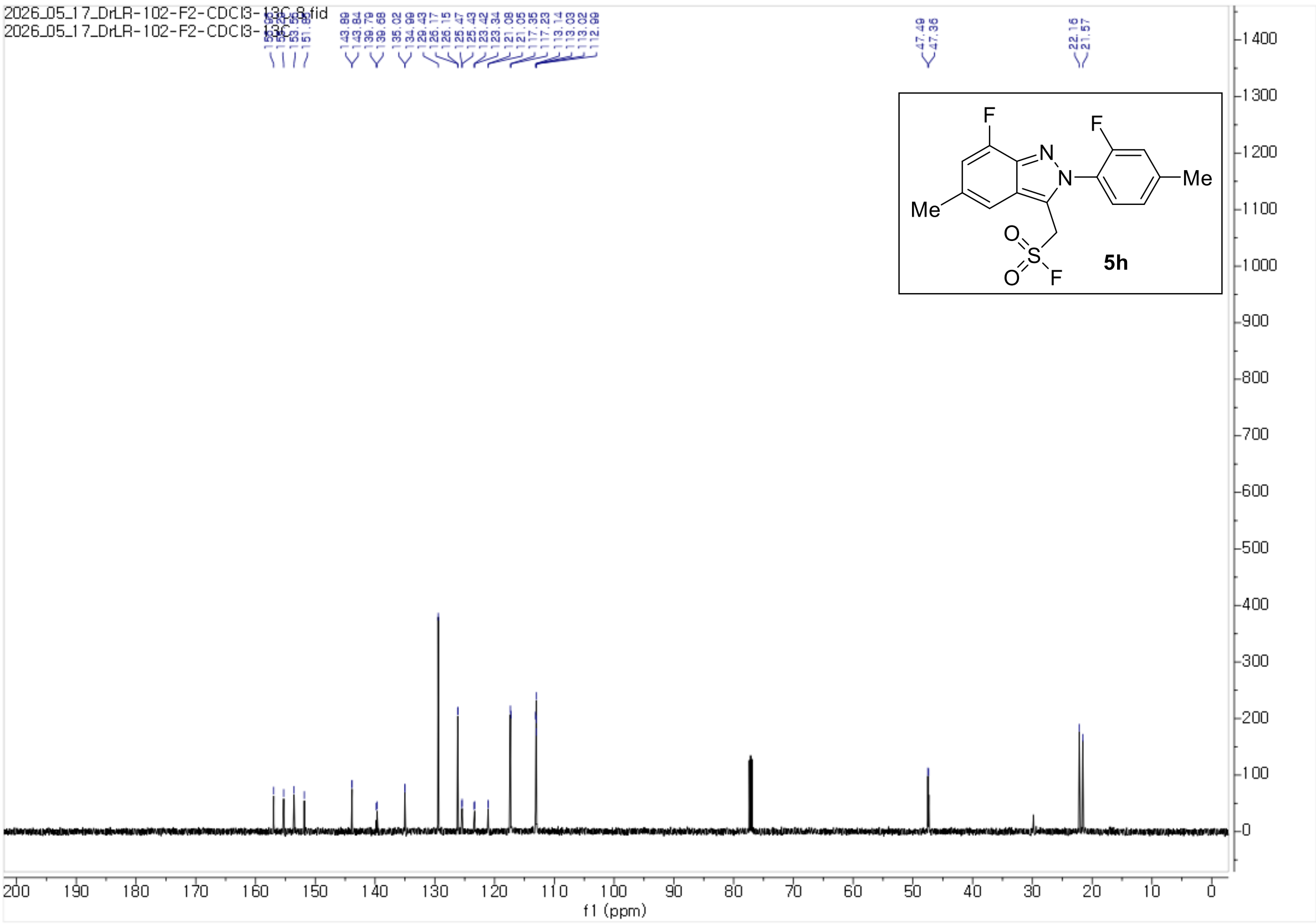
2026_05_17_DrLR-102-F2-CDCl3-13C.8.fid
5h
Me
F
N
N
F
Me
O
S
O
F
f1 (ppm)

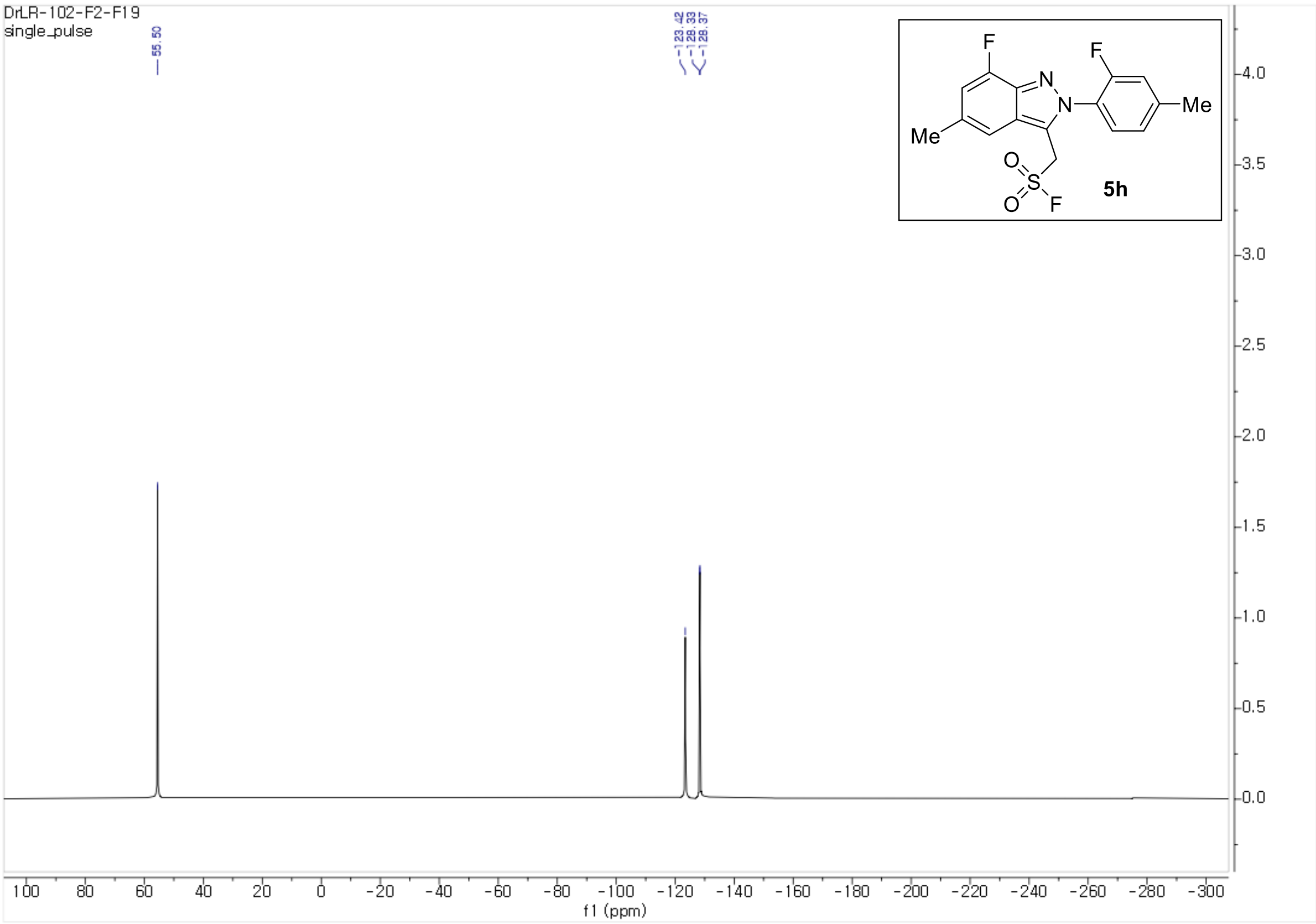
DrLR-102-F2-F19
single_pulse
55.50
-123.42
-128.33
-128.37
F
F
N
N
Me
Me
O
S
O
F
5h
4.0
3.5
3.0
2.5
2.0
1.5
1.0
0.5
0.0
100
80
60
40
20
0
-20
-40
-60
-80
-100
-120
-140
-160
-180
-200
-220
-240
-260
-280
-300
f1 (ppm)

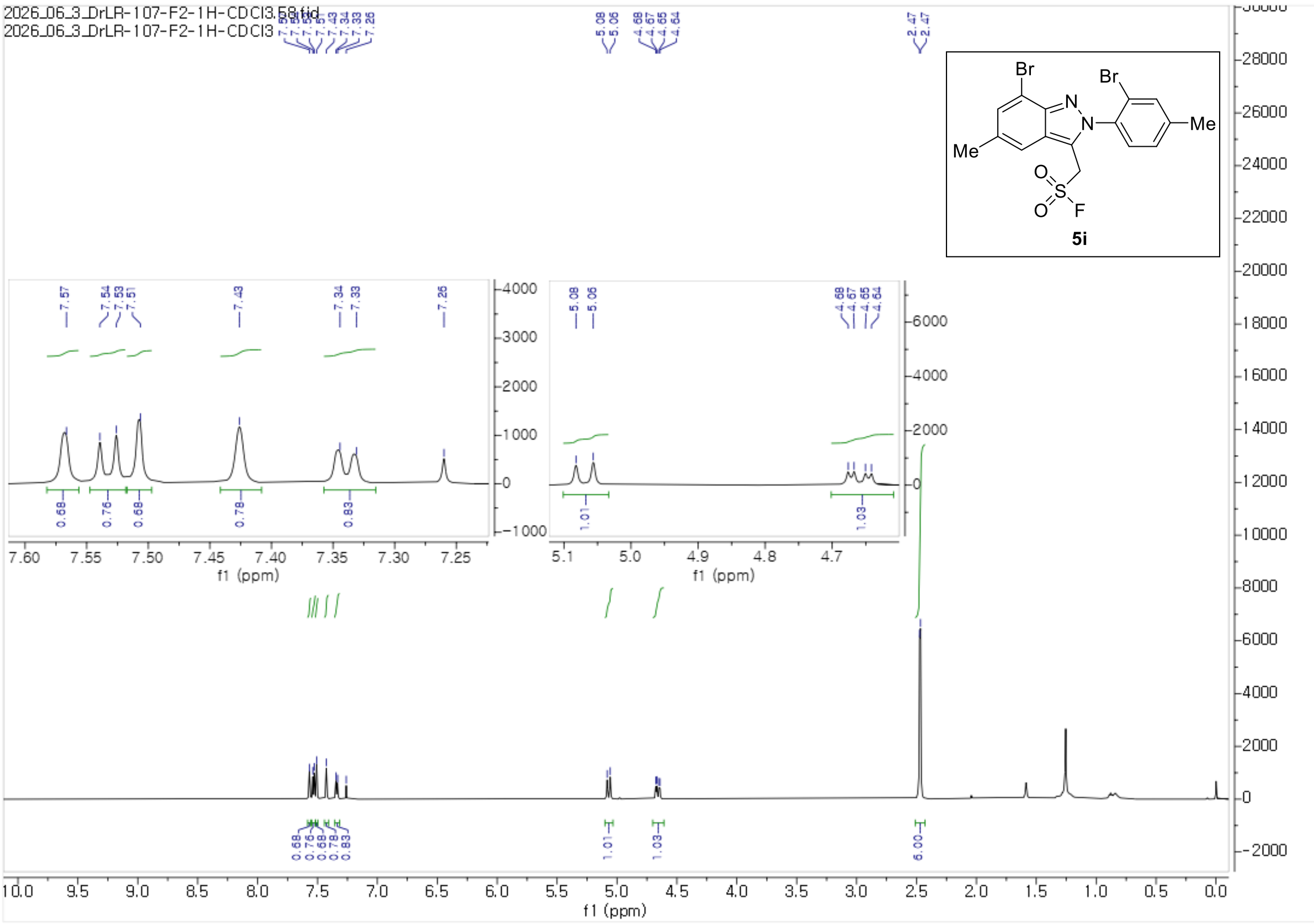

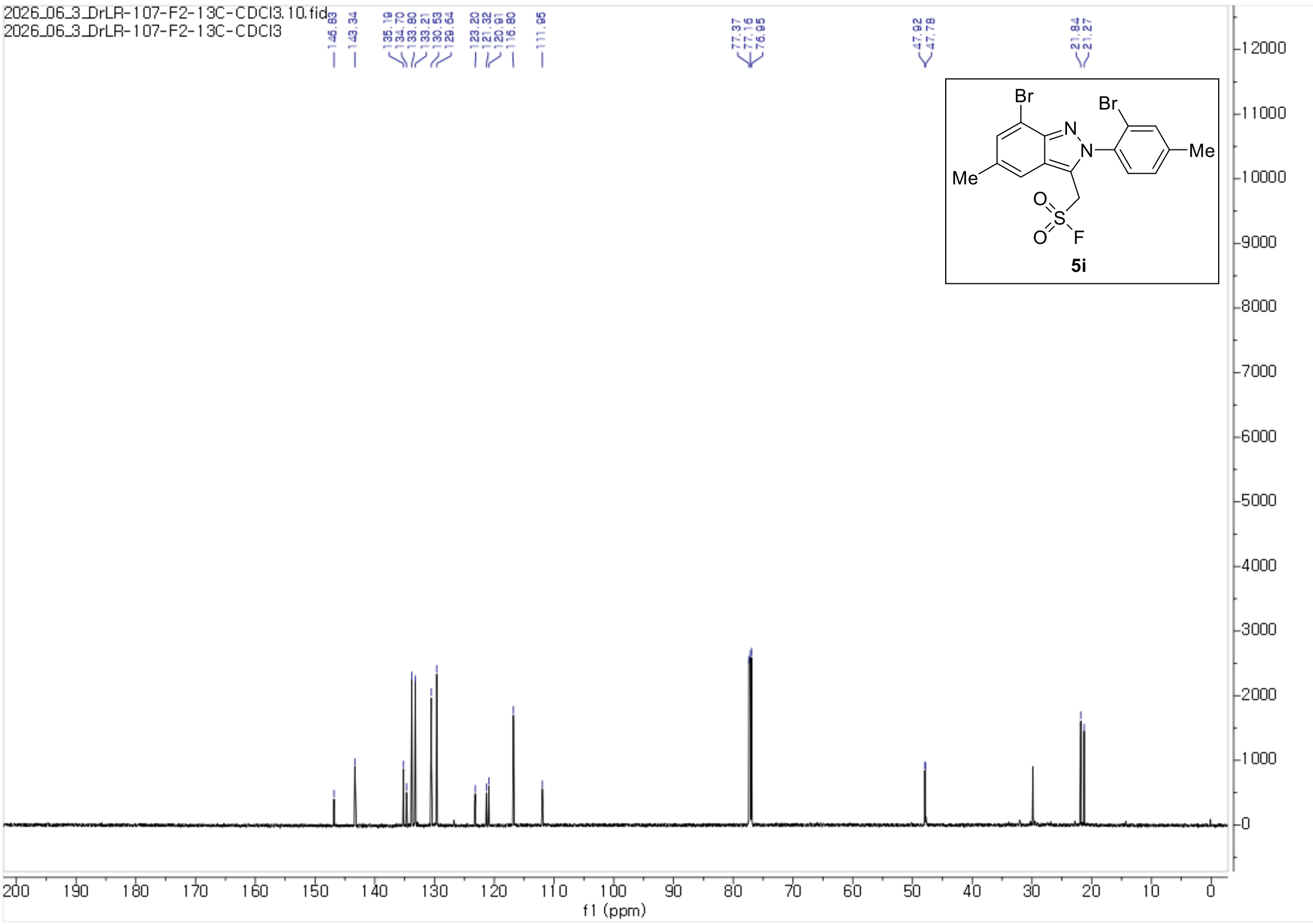

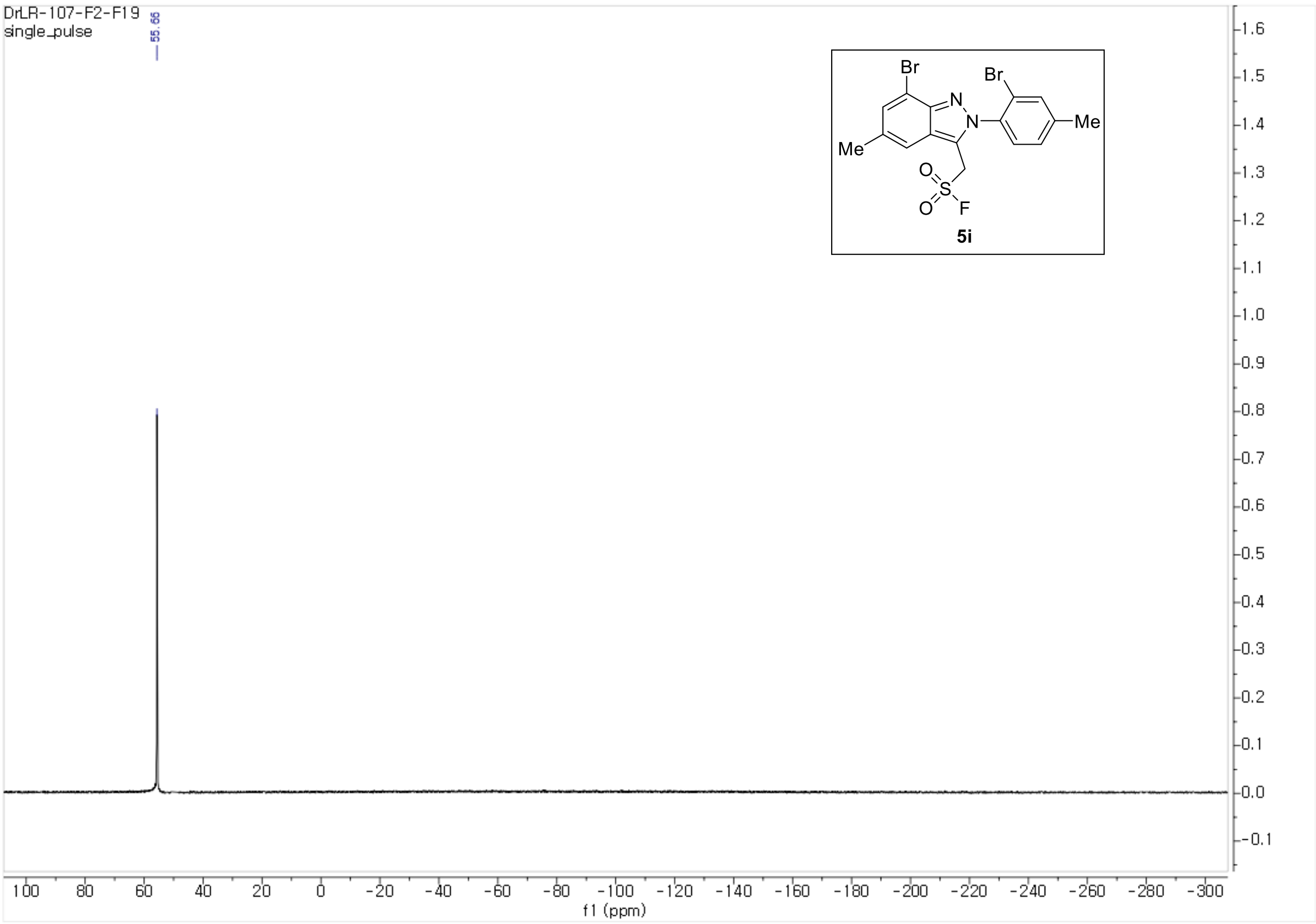
DrLR-107-F2-F19
single_pulse
55.66
Br
Br
N
N
Me
Me
O
S
O
F
5i
f1 (ppm)

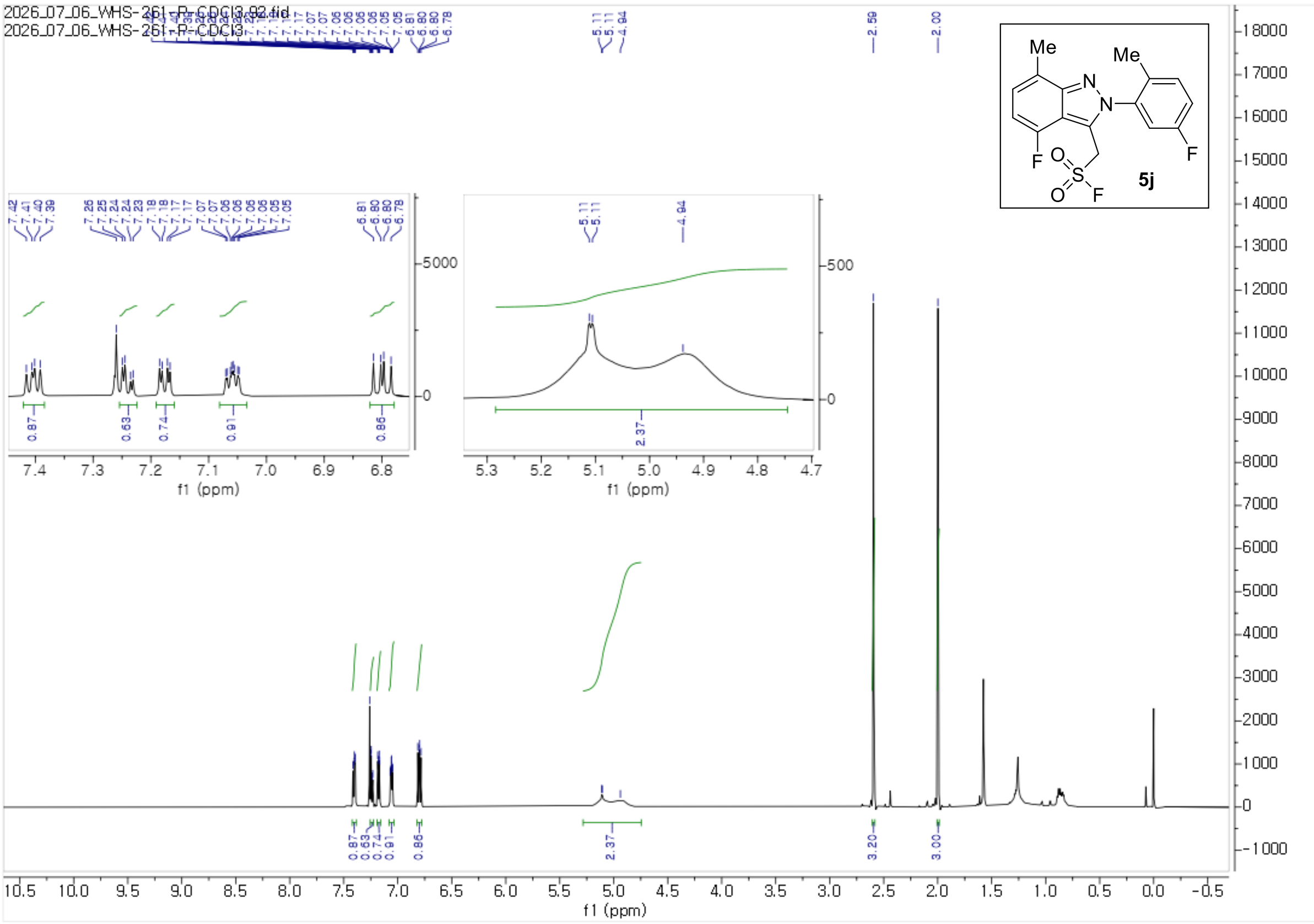

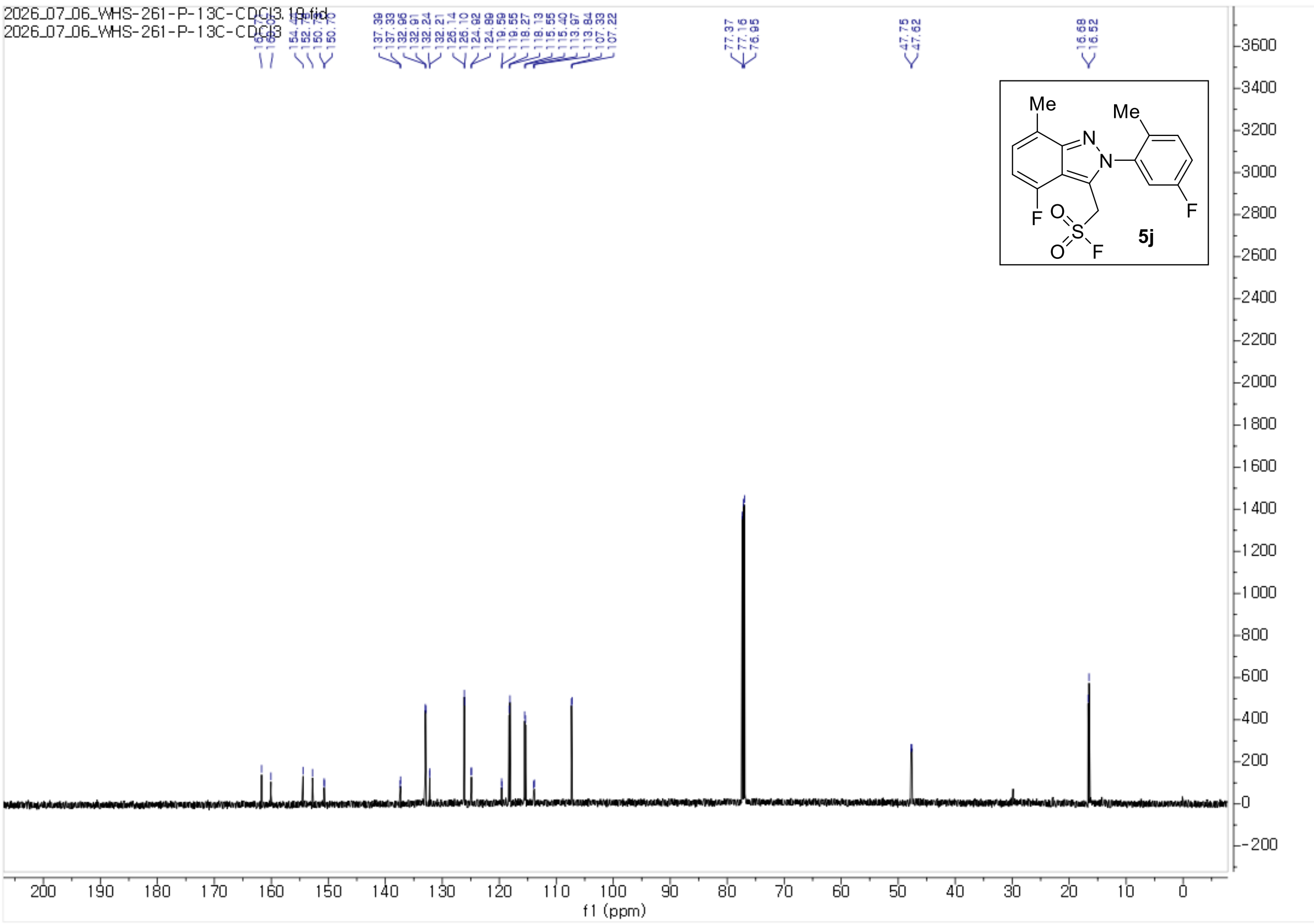

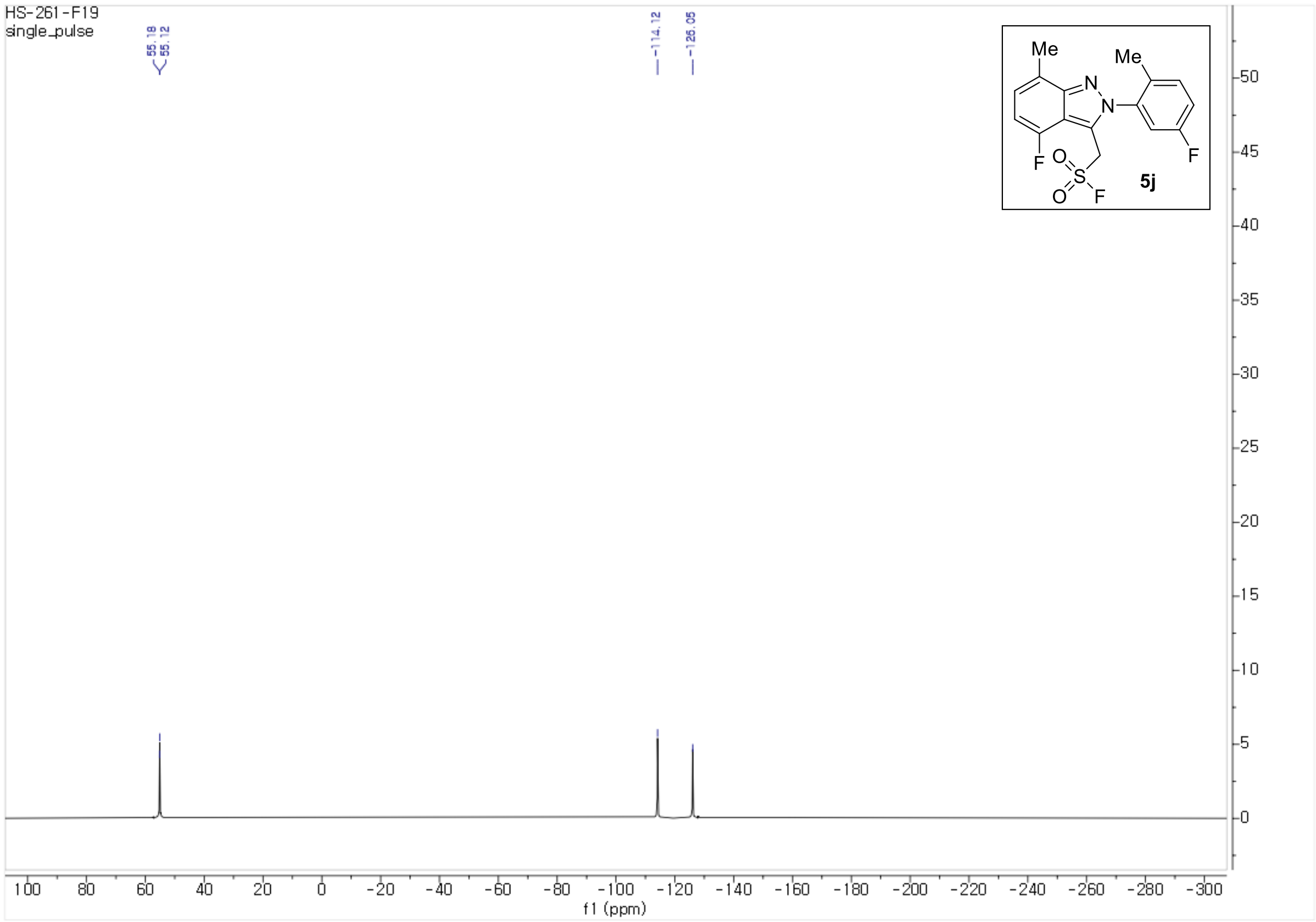
HS-261-F19
single_pulse
55.18
55.12
-114.12
-126.05
Me
Me
N
N
F
O
S
O
F
F
5j
f1 (ppm)

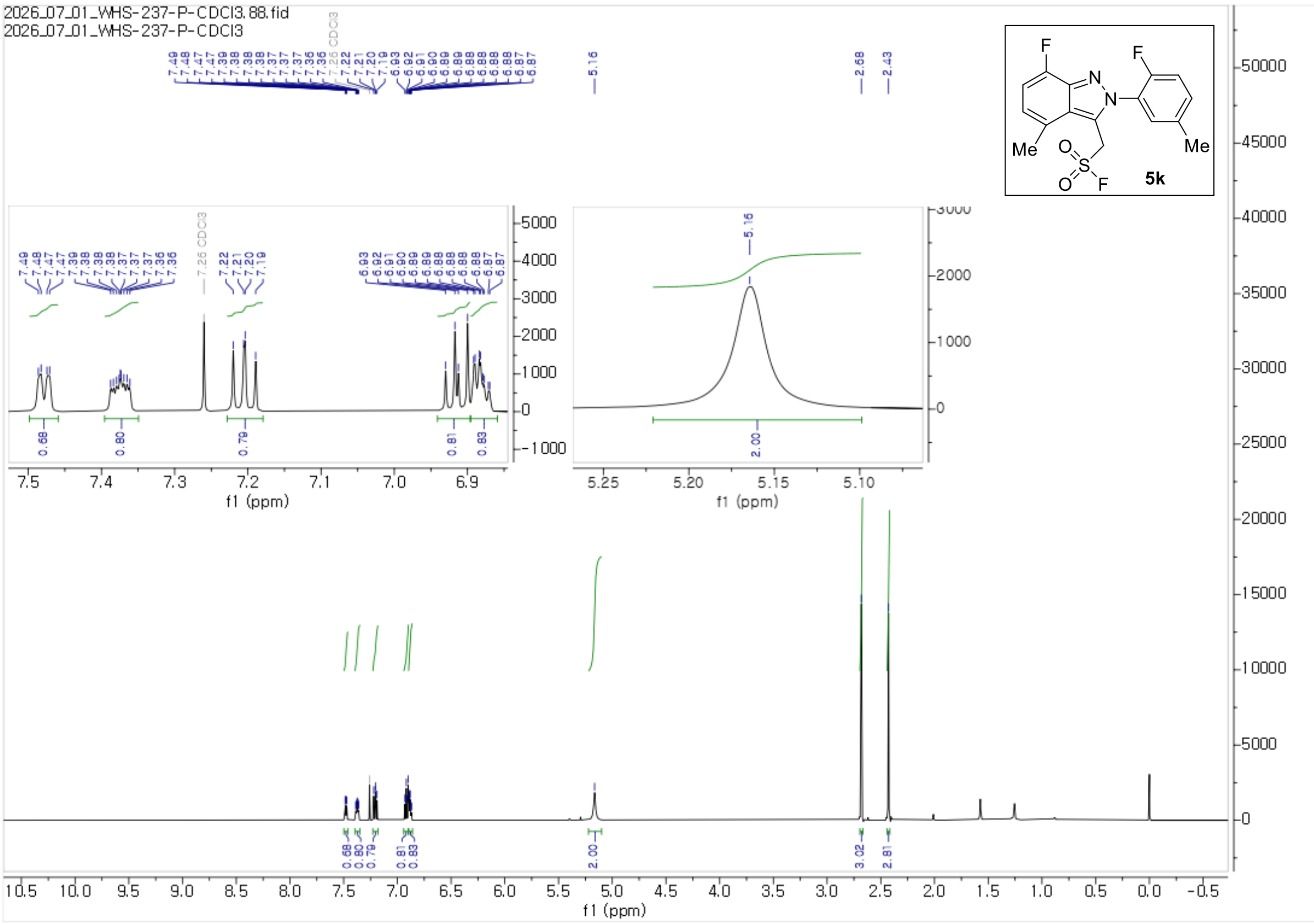

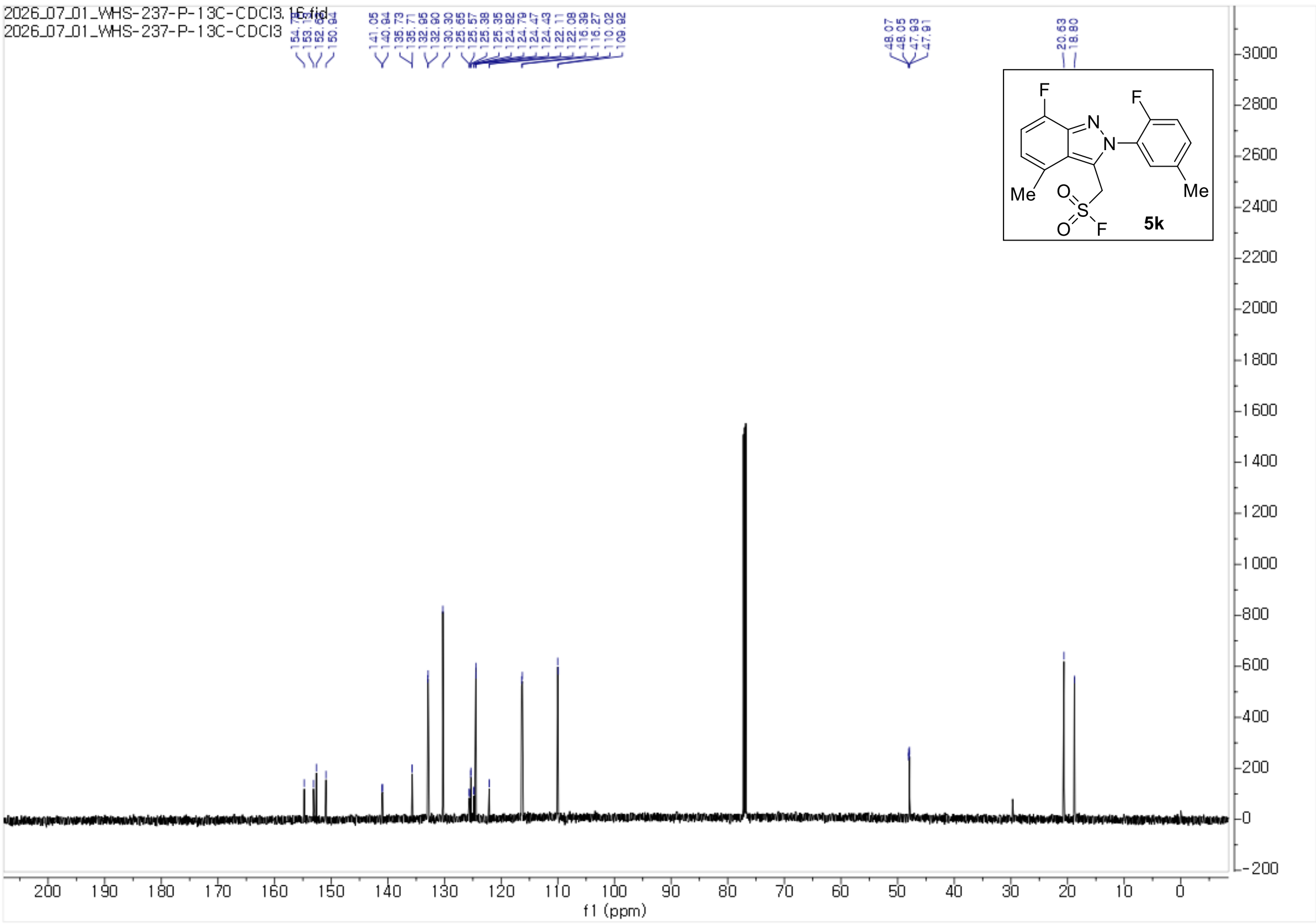

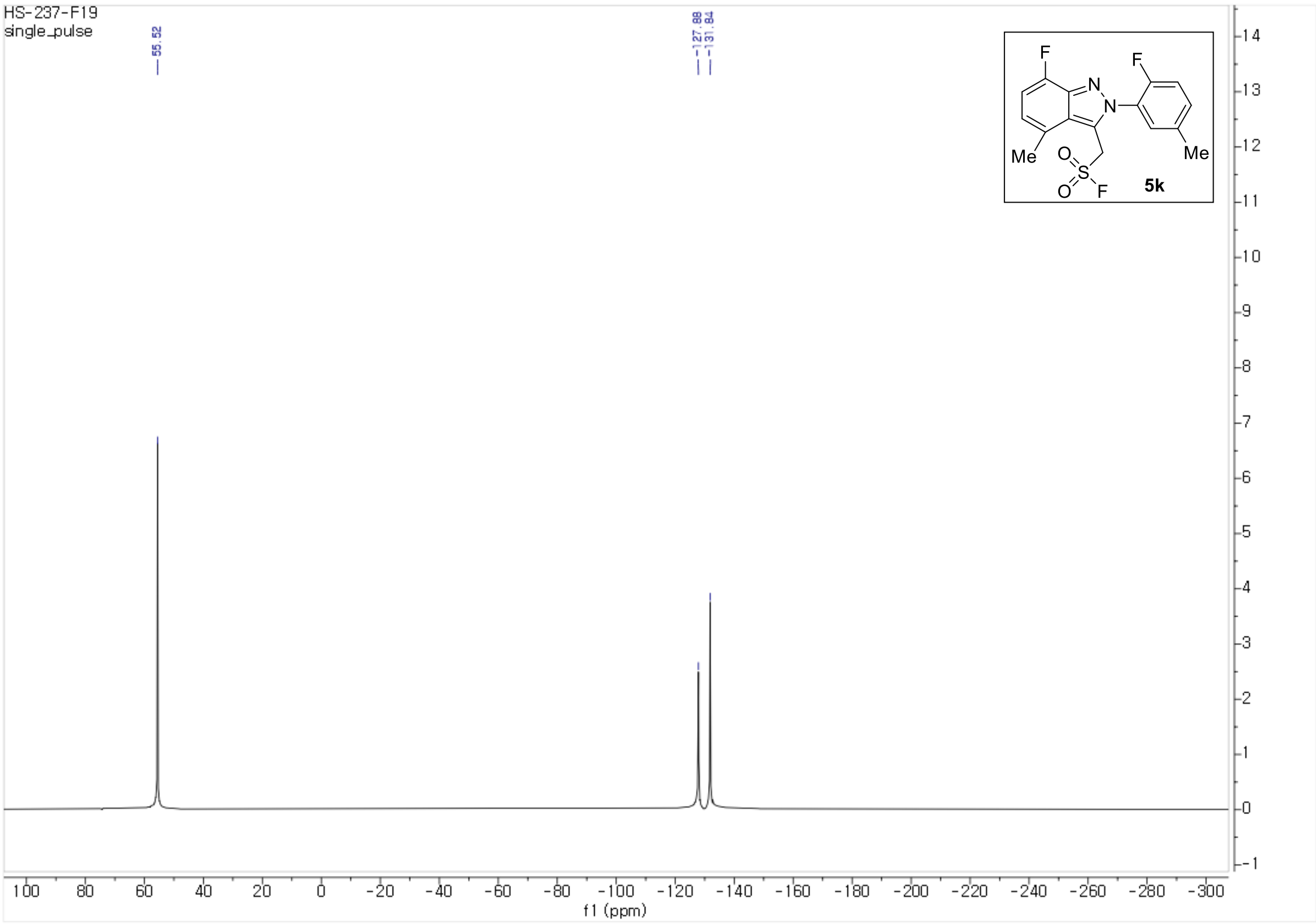
HS-237-F19
single_pulse
55.52
-127.88
-131.84
F
N
N
F
Me
O
S
O
F
Me
5k
f1 (ppm)

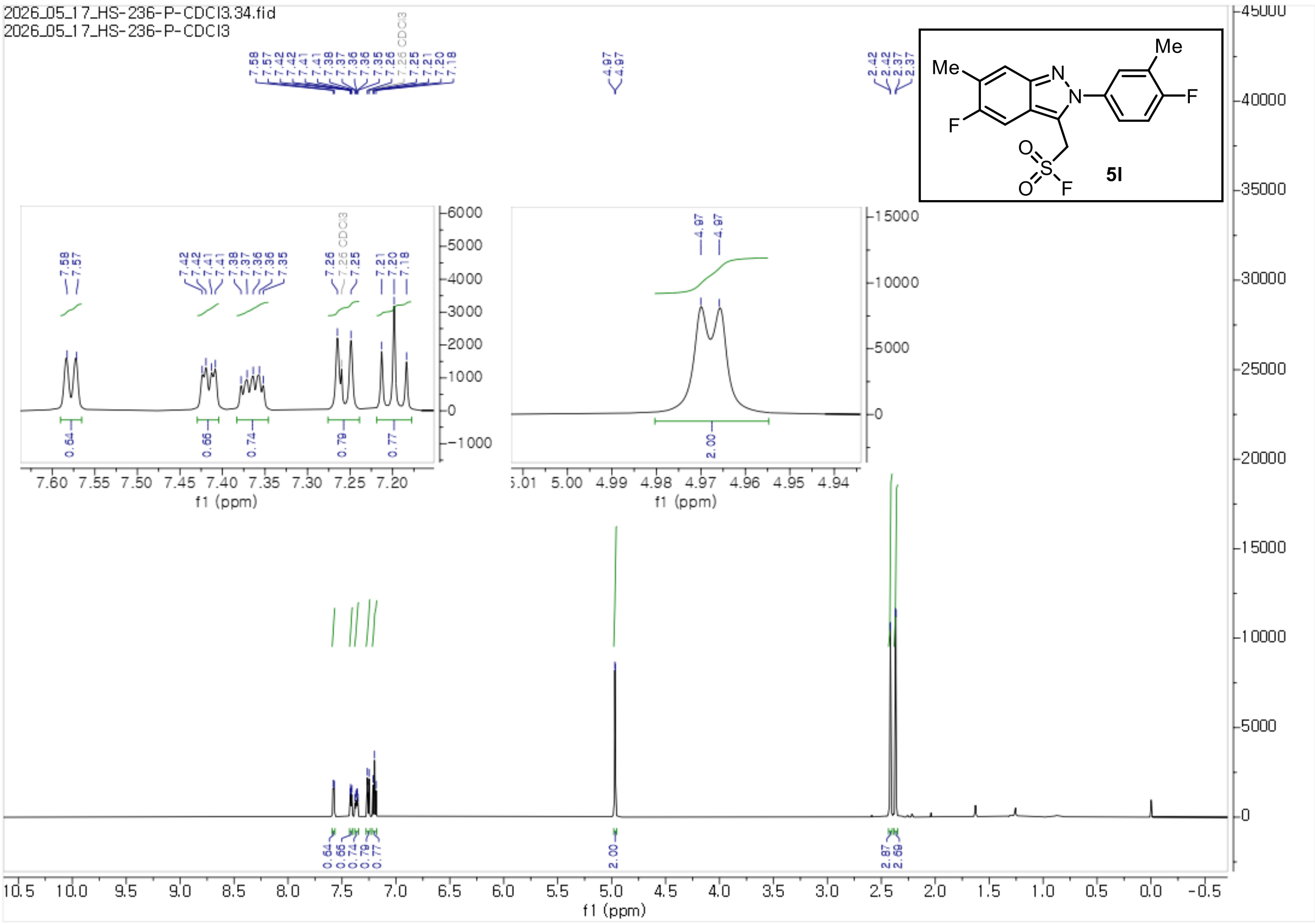
2026_05_17_HS-236-P-CDCl3.34.fid
2026_05_17_HS-236-P-CDCl3
5l
f1 (ppm)

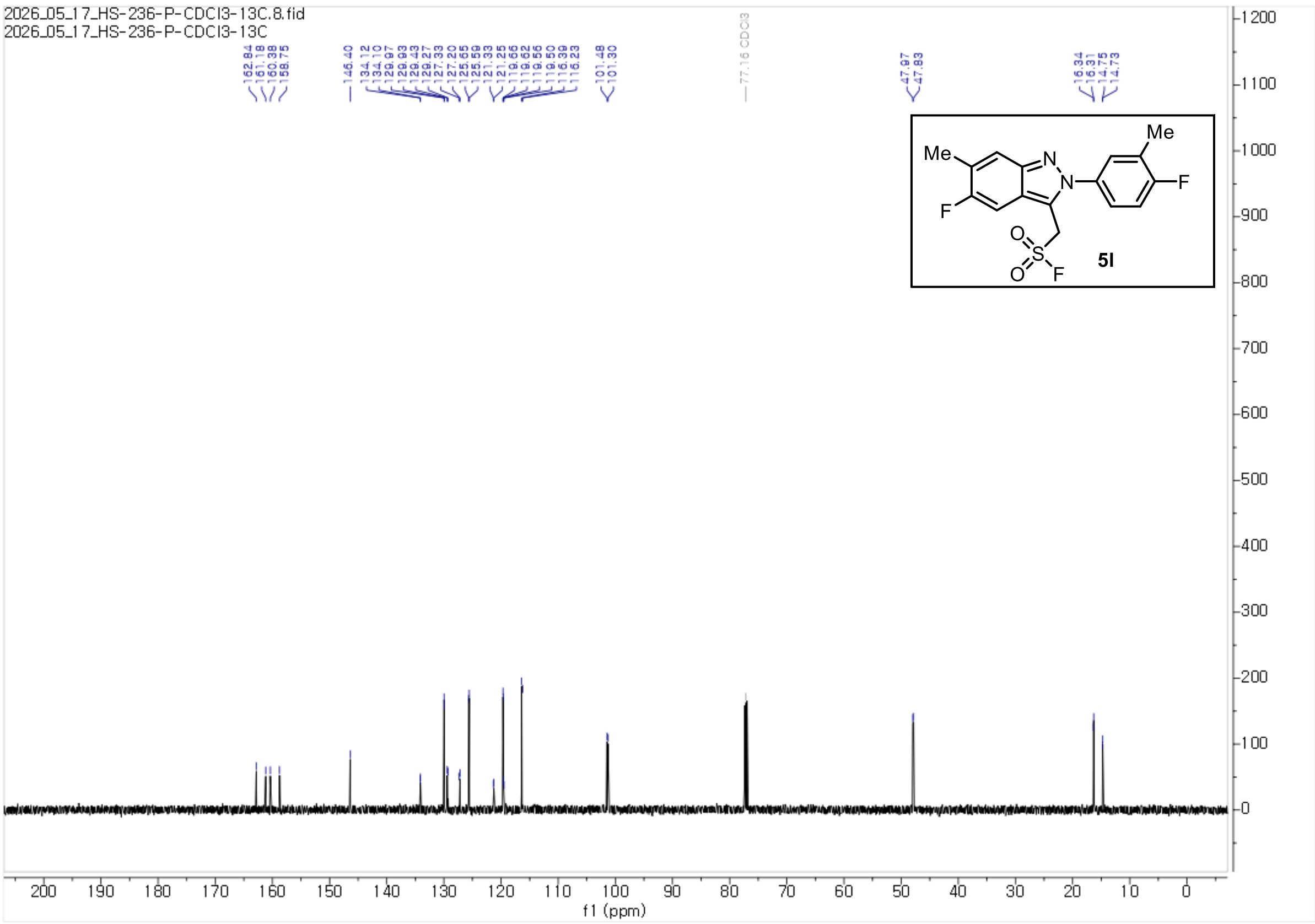
2026_05_17_HS-236-P-CDCl3-13C.8.fid
2026_05_17_HS-236-P-CDCl3-13C
5l
f1 (ppm)

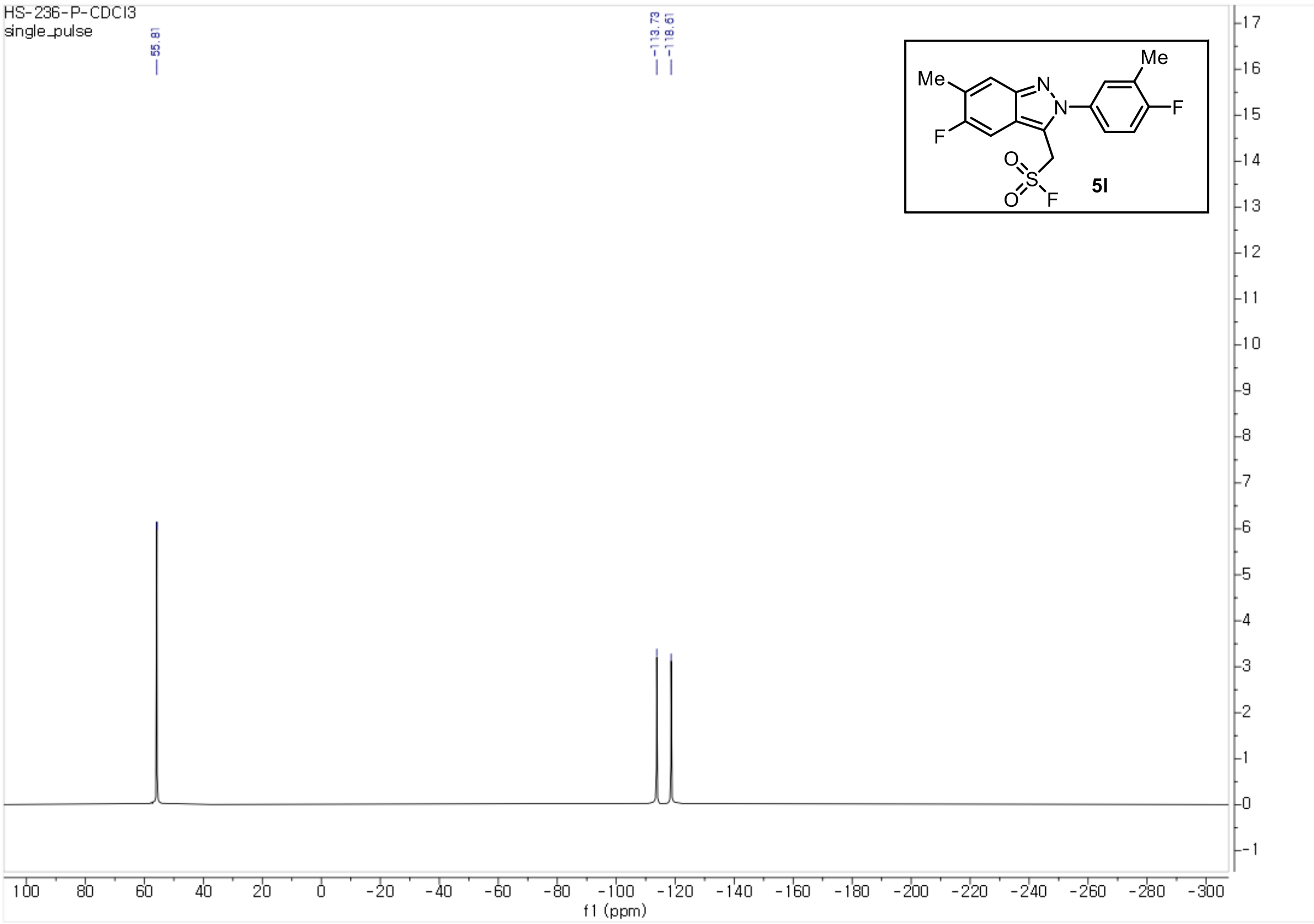
HS-236-P-CDCl3
single_pulse
55.81
-113.73
-118.61
Me
N
N
Me
F
F
O
S
O
F
5l
f1 (ppm)

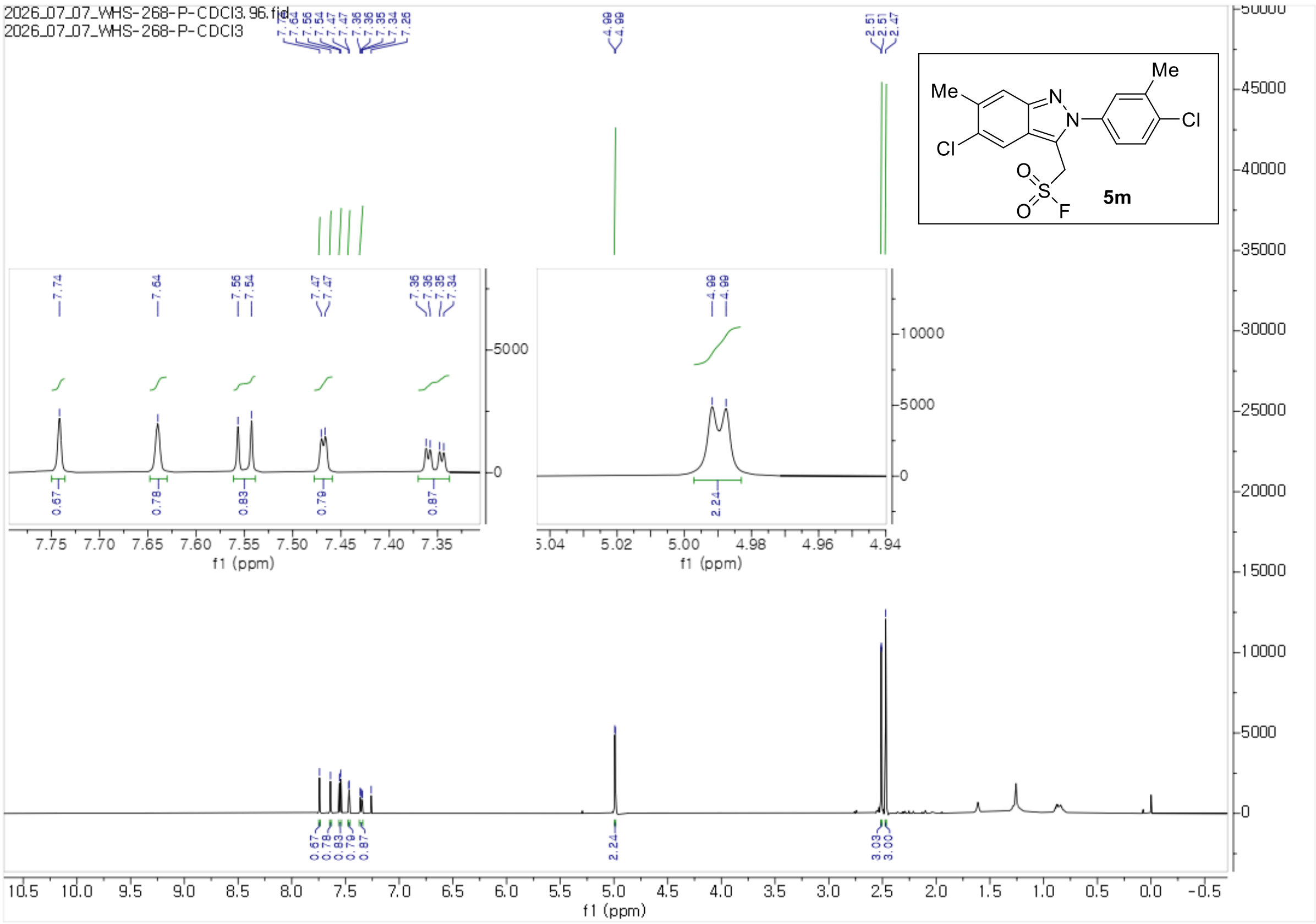

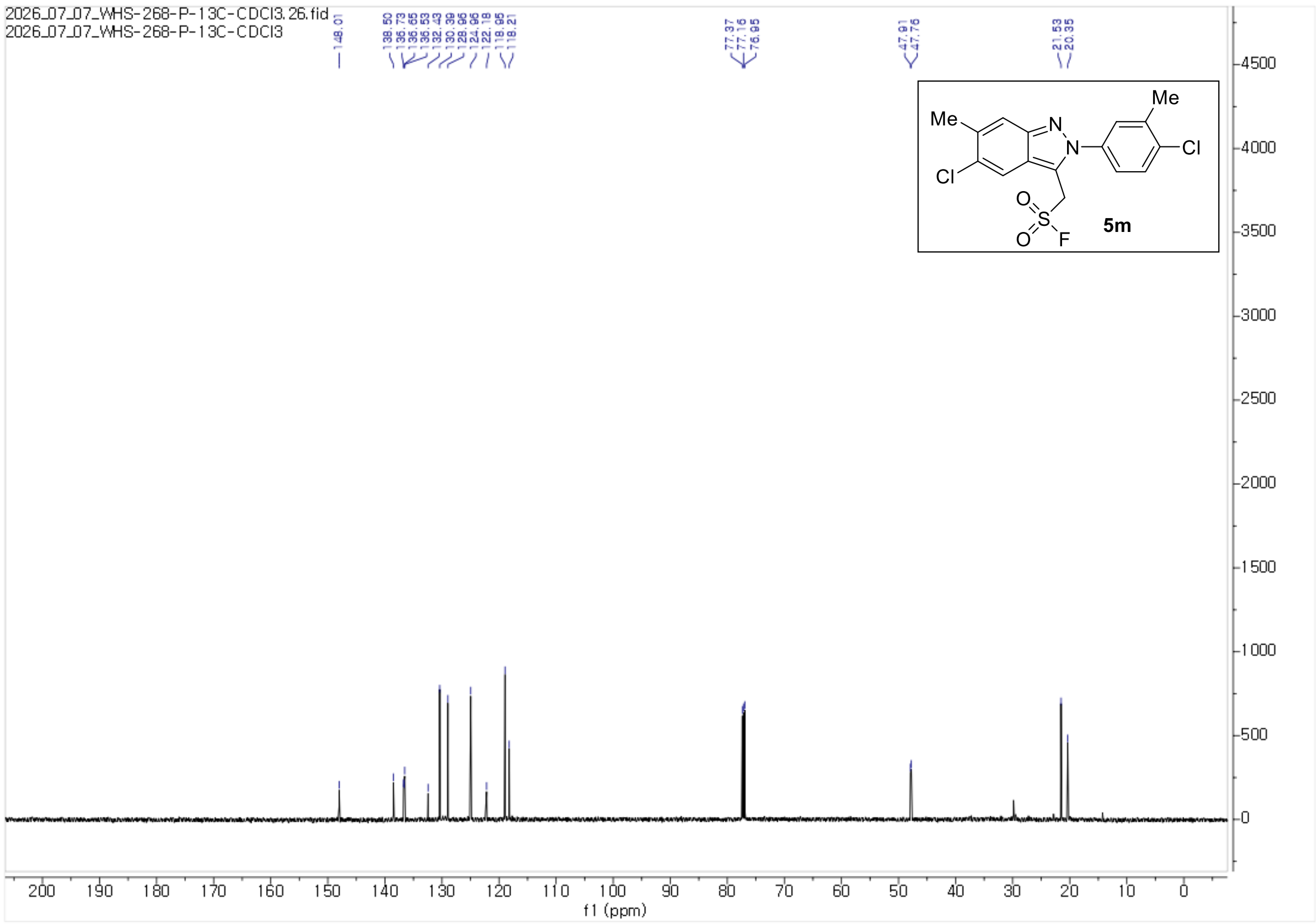

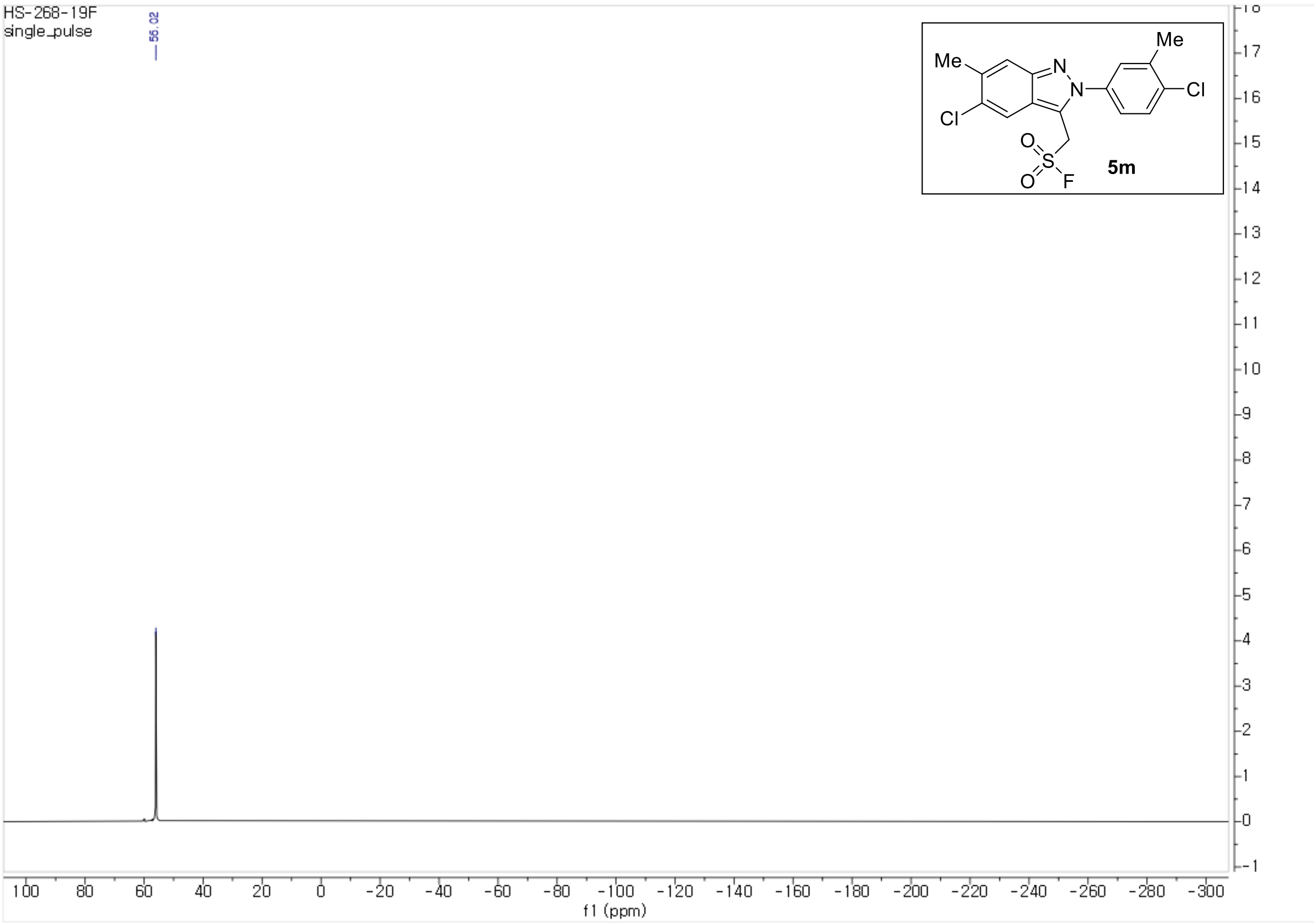

HS-268-19F
single_pulse
55.02
Me
N
N
Me
Cl
Cl
O
S
O
F
5m
f1 (ppm)

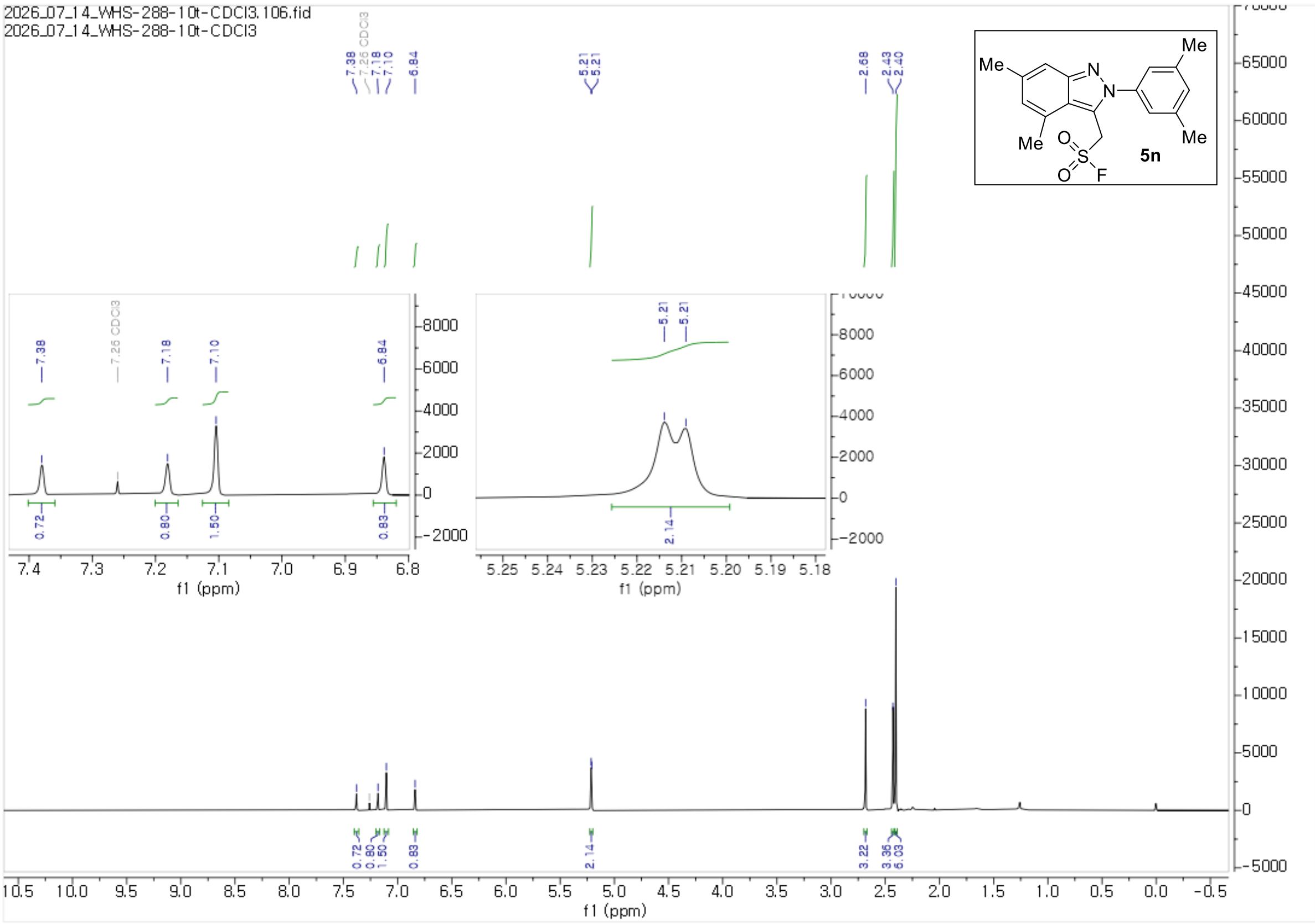
2026_07_14_WHS-288-10t-CDCl3.106.fid
2026_07_14_WHS-288-10t-CDCl3
5n
f1 (ppm)

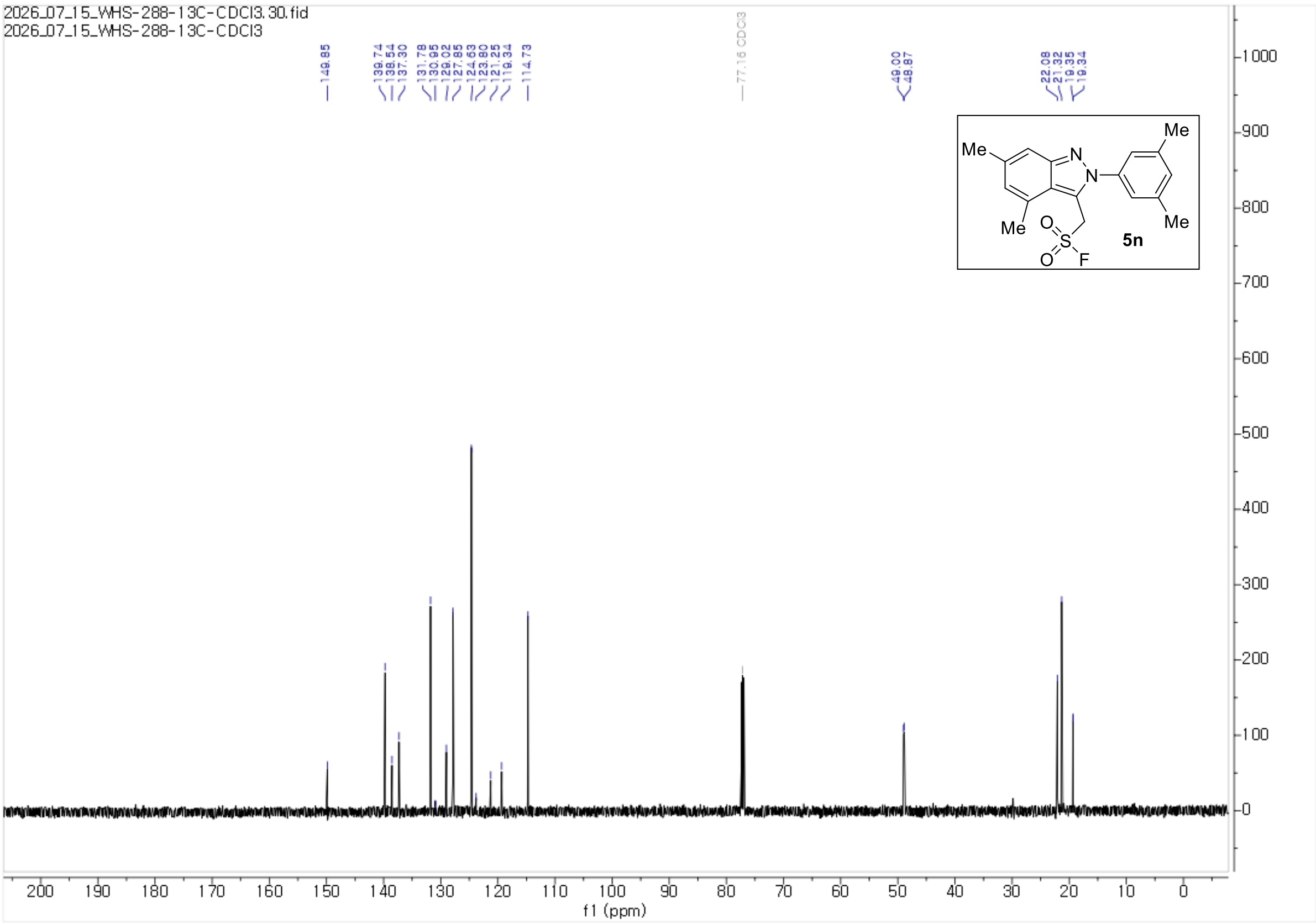
2026_07_15_WHS-288-13C-CDCl3.30.fid
2026_07_15_WHS-288-13C-CDCl3
5n
f1 (ppm)

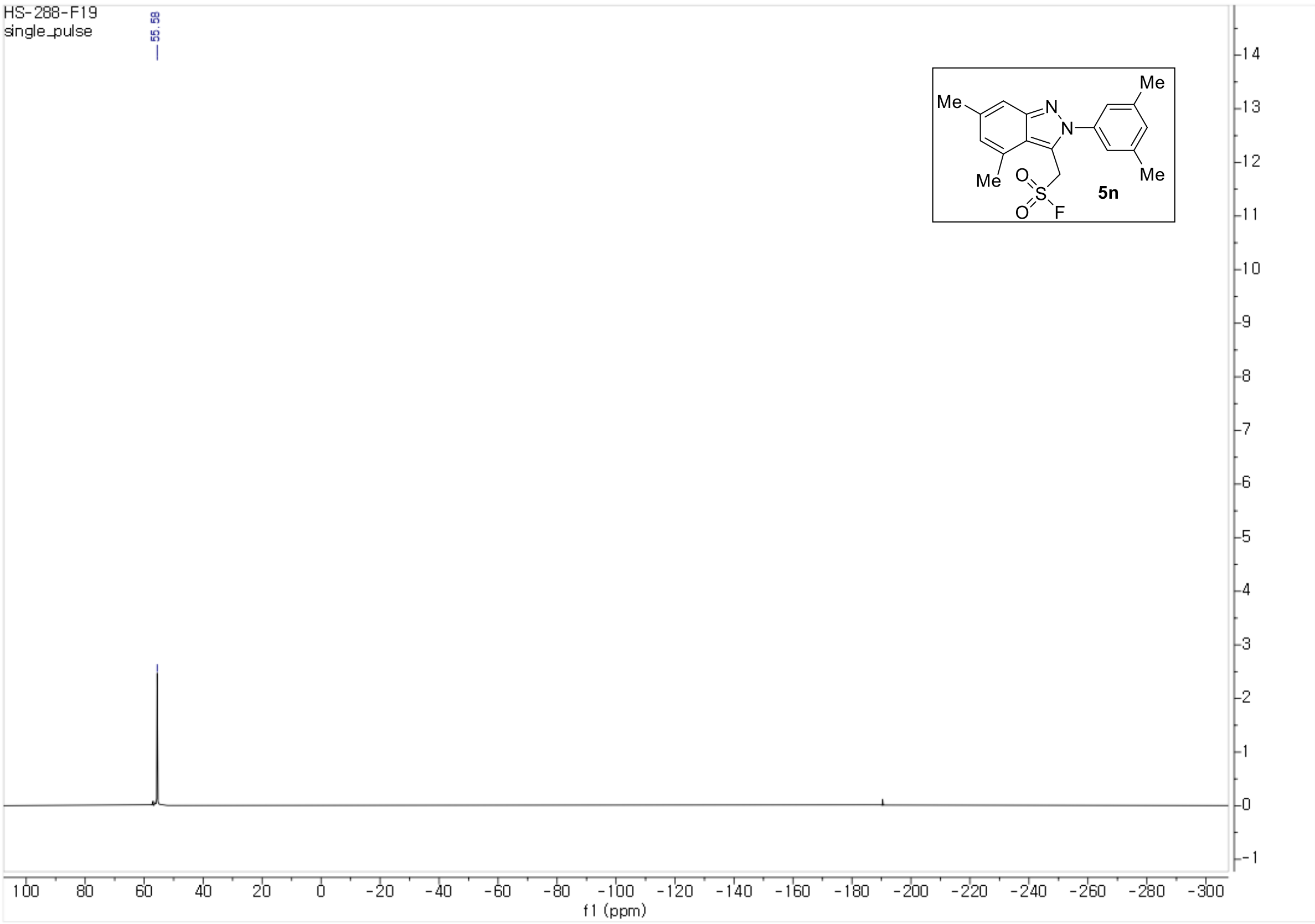
HS-288-F19
single_pulse
55.59
5n
f1 (ppm)

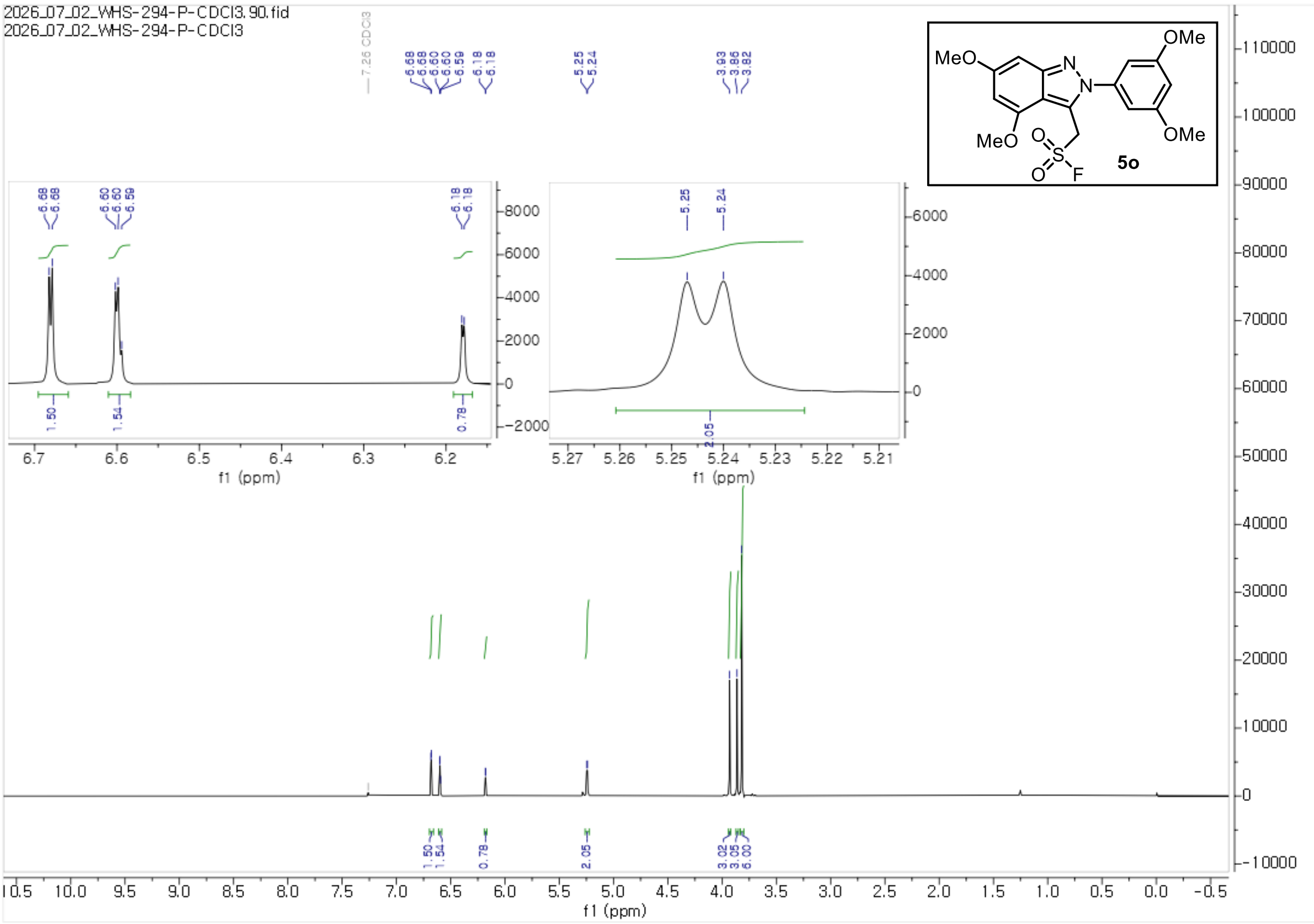
2026_07_02_WHS-294-P-CDCl3.90.fid
2026_07_02_WHS-294-P-CDCl3
7.26 CDCl3
6.68
6.68
6.60
6.60
6.59
6.18
6.18
5.25
5.24
3.93
3.86
3.82
1.50
1.54
0.78
2.05
3.02
3.05
6.00
MeO
N
N
OMe
MeO
O
S
O
F
OMe
5o
f1 (ppm)

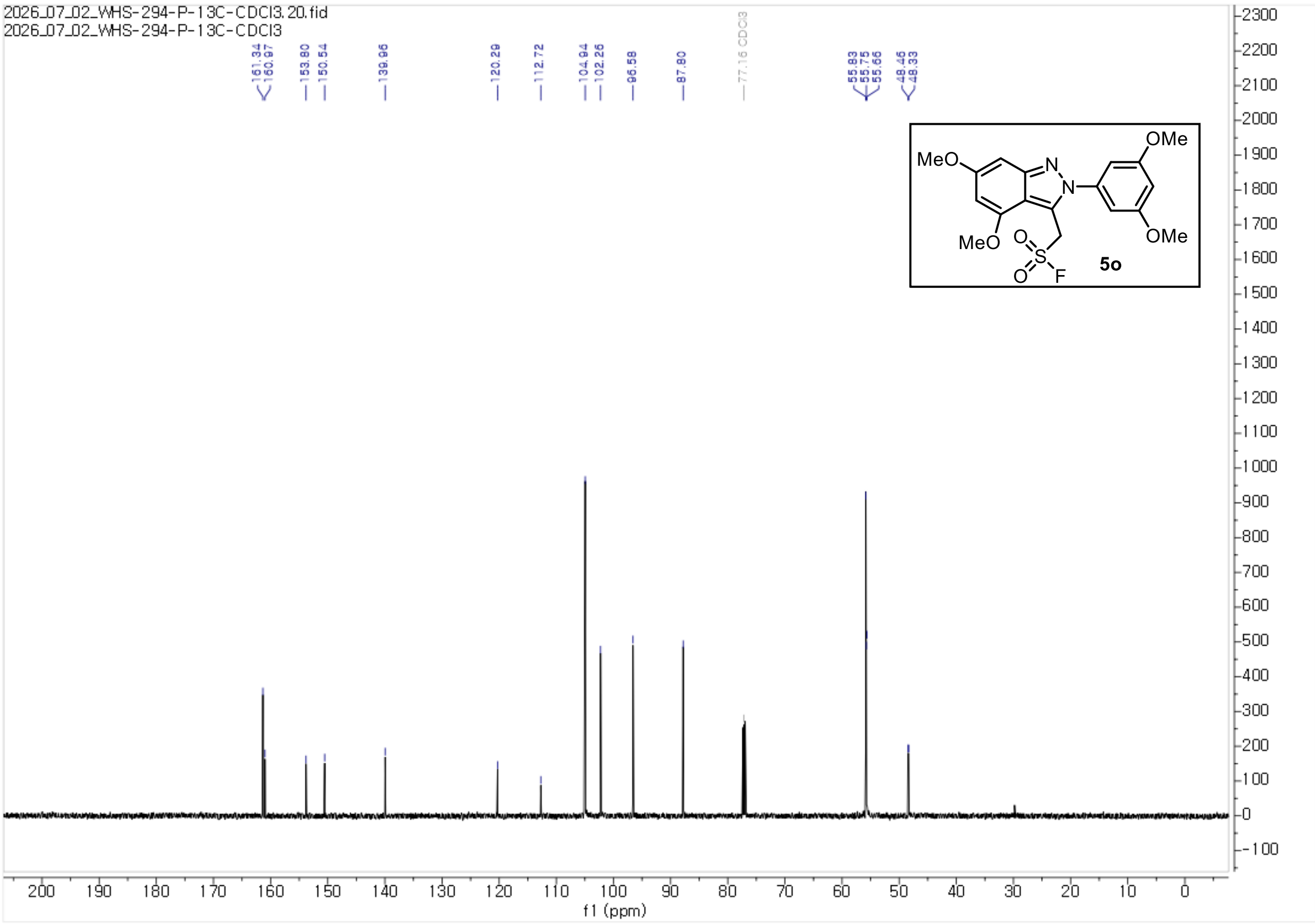
2026_07_02_WHS-294-P-13C-CDCl3.20.fid
2026_07_02_WHS-294-P-13C-CDCl3
161.34
160.97
153.80
150.54
139.96
120.29
112.72
104.94
102.26
96.58
87.80
77.16 CDCl3
55.83
55.75
55.66
48.46
48.33
MeO
N
N
OMe
MeO
O
S
O
F
OMe
5o
f1 (ppm)

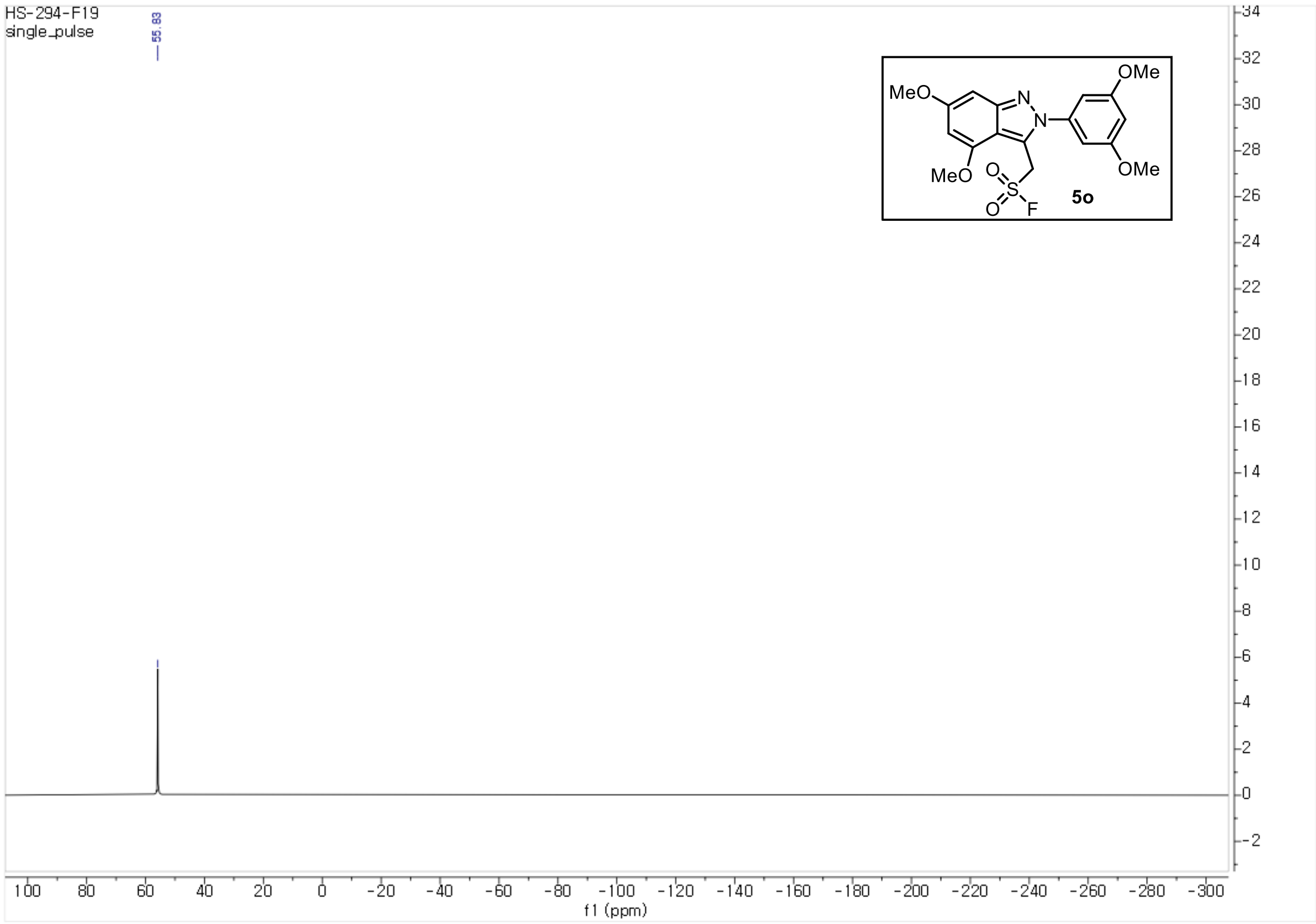
HS-294-F19
single_pulse
55.83
MeO
N
N
OMe
MeO
O
S
O
F
OMe
5o
f1 (ppm)

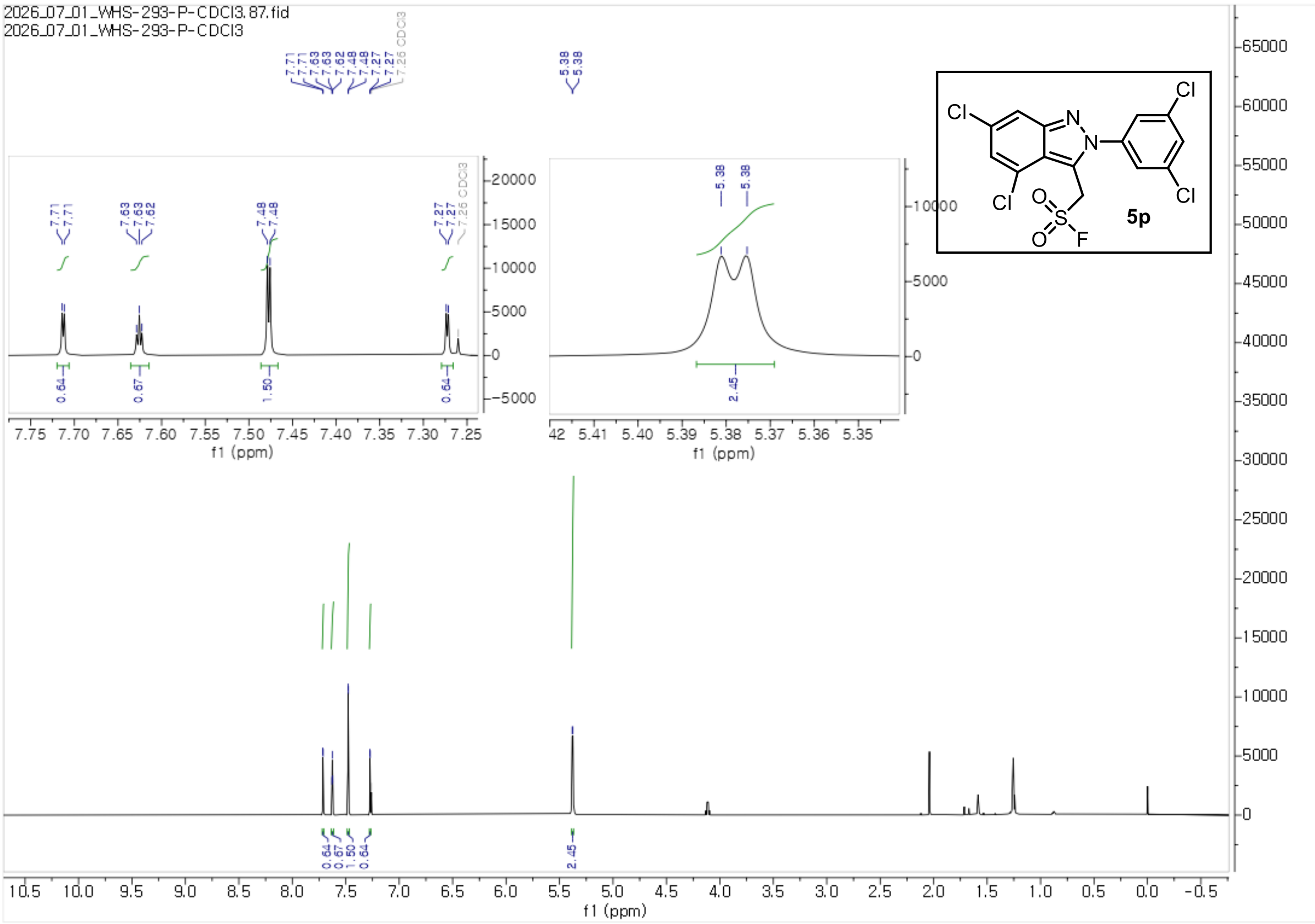
2026_07_01_WHS-293-P-CDCl3.87.fid
2026_07_01_WHS-293-P-CDCl3
5p
f1 (ppm)

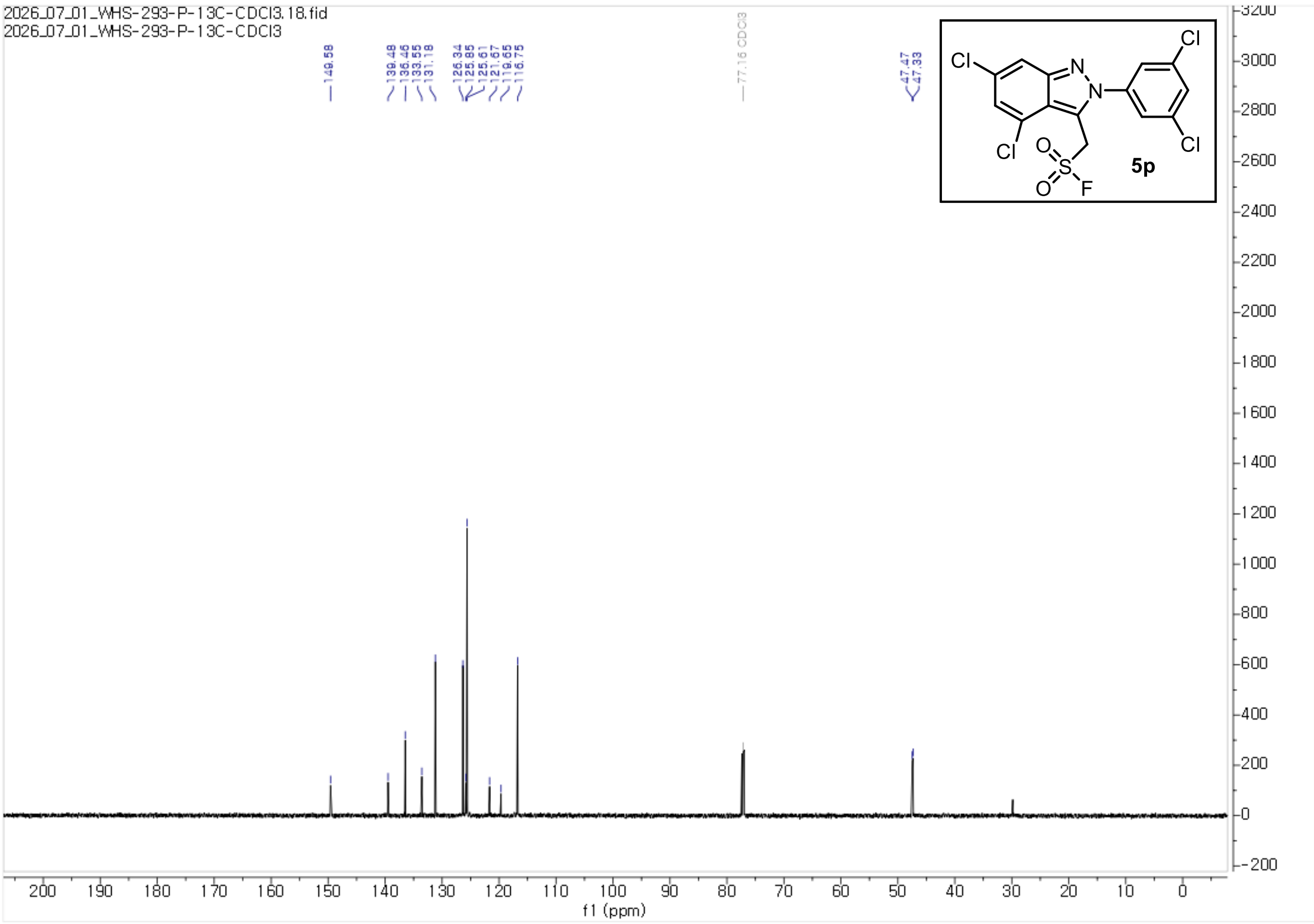
2026_07_01_WHS-293-P-13C-CDCl3.18.fid
2026_07_01_WHS-293-P-13C-CDCl3
5p
f1 (ppm)

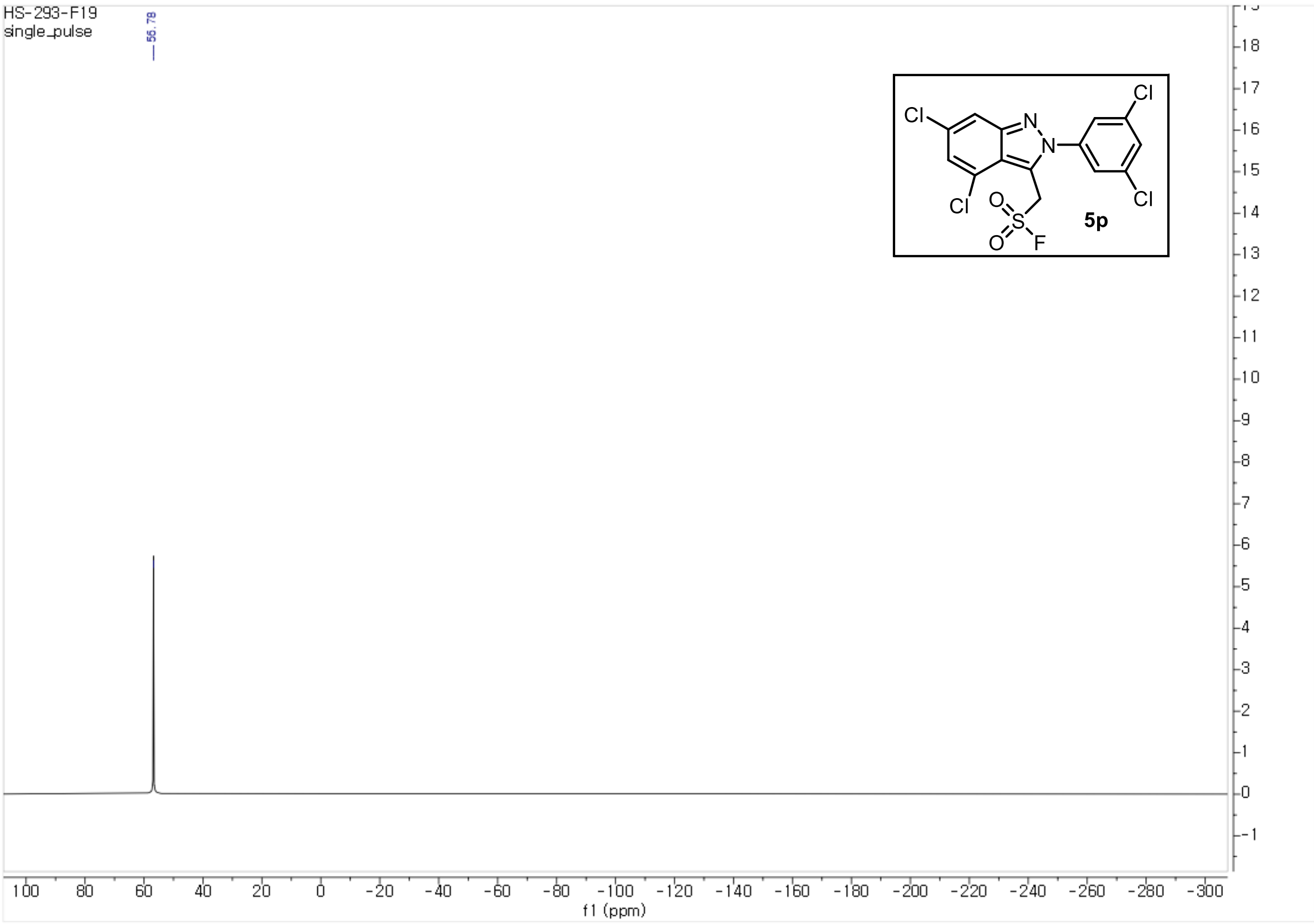
HS-293-F19
single_pulse
55.78
Cl
N
N
Cl
Cl
O
S
O
F
Cl
5p
f1 (ppm)

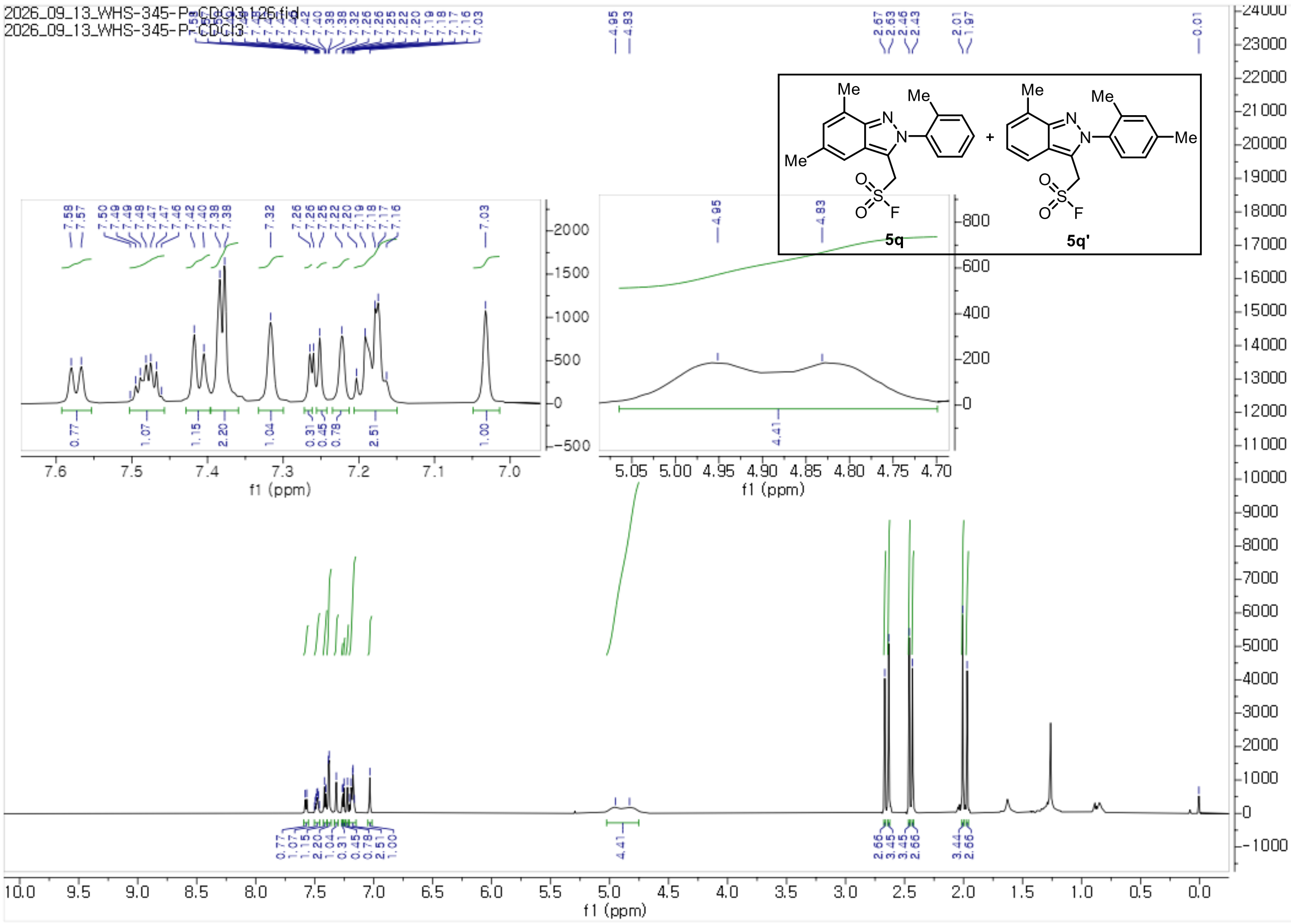
2026_09_13_WHS-345-P-CDCl3-1H.26.fid
2026_09_13_WHS-345-P-CDCl3
5q
5q'
f1 (ppm)

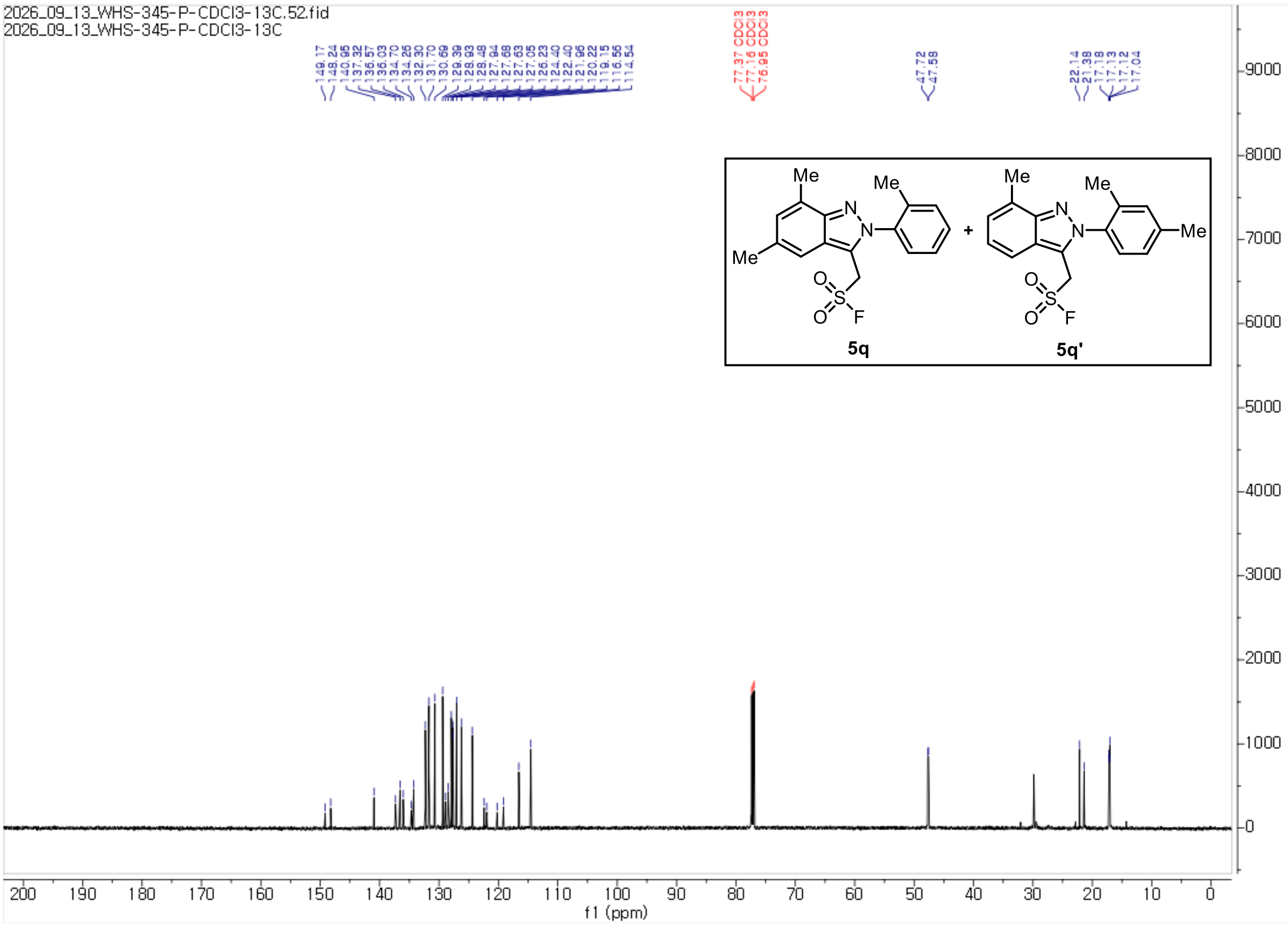
2026_09_13_WHS-345-P-CDCl3-13C.52.fid
2026_09_13_WHS-345-P-CDCl3-13C
5q
5q'
f1 (ppm)

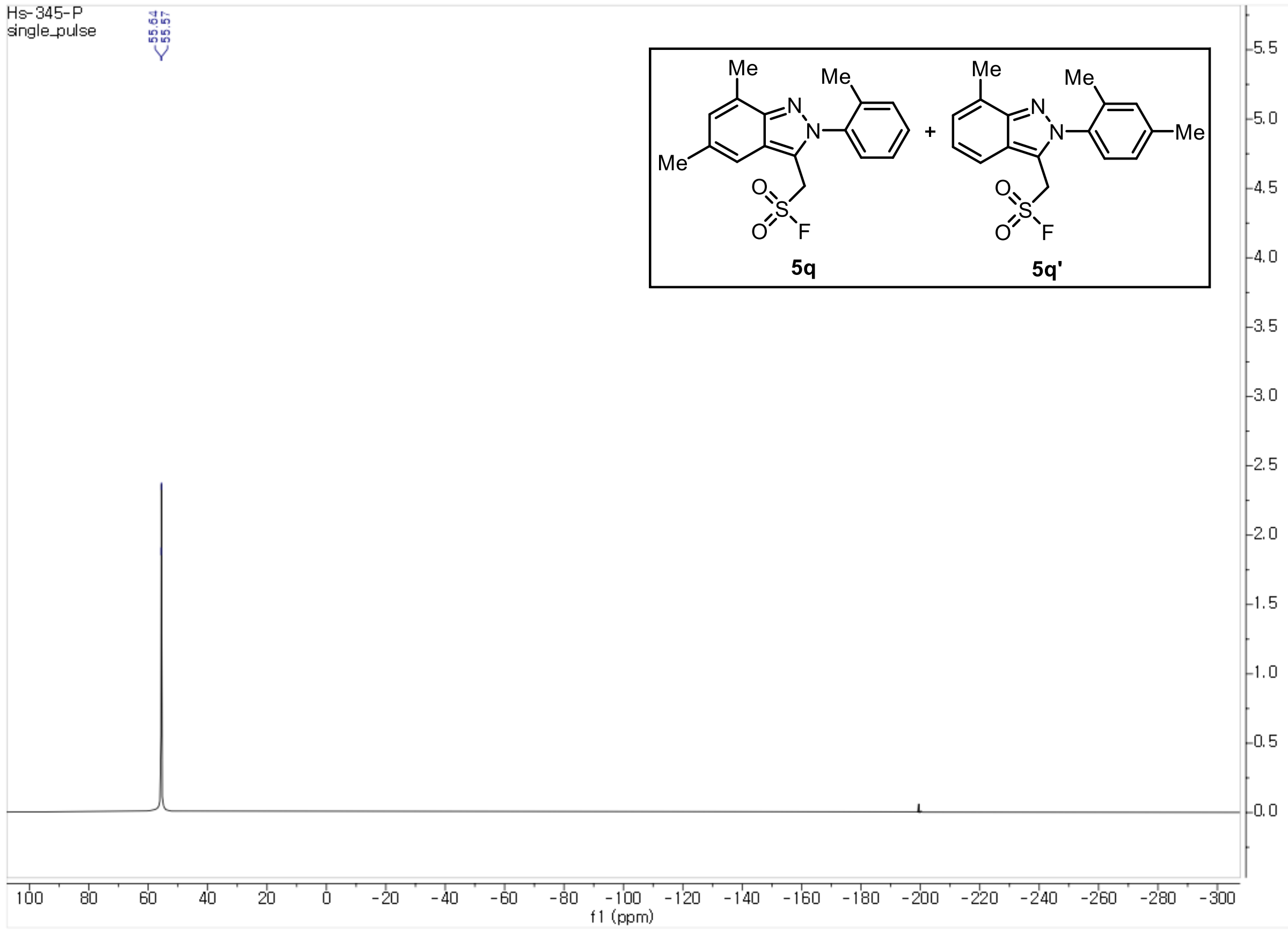
Hs-345-P
single_pulse
55.64
55.57
Me
Me
N
N
Me
+
Me
Me
N
N
Me
O
S
O
F
O
S
O
F
5q
5q'
f1 (ppm)

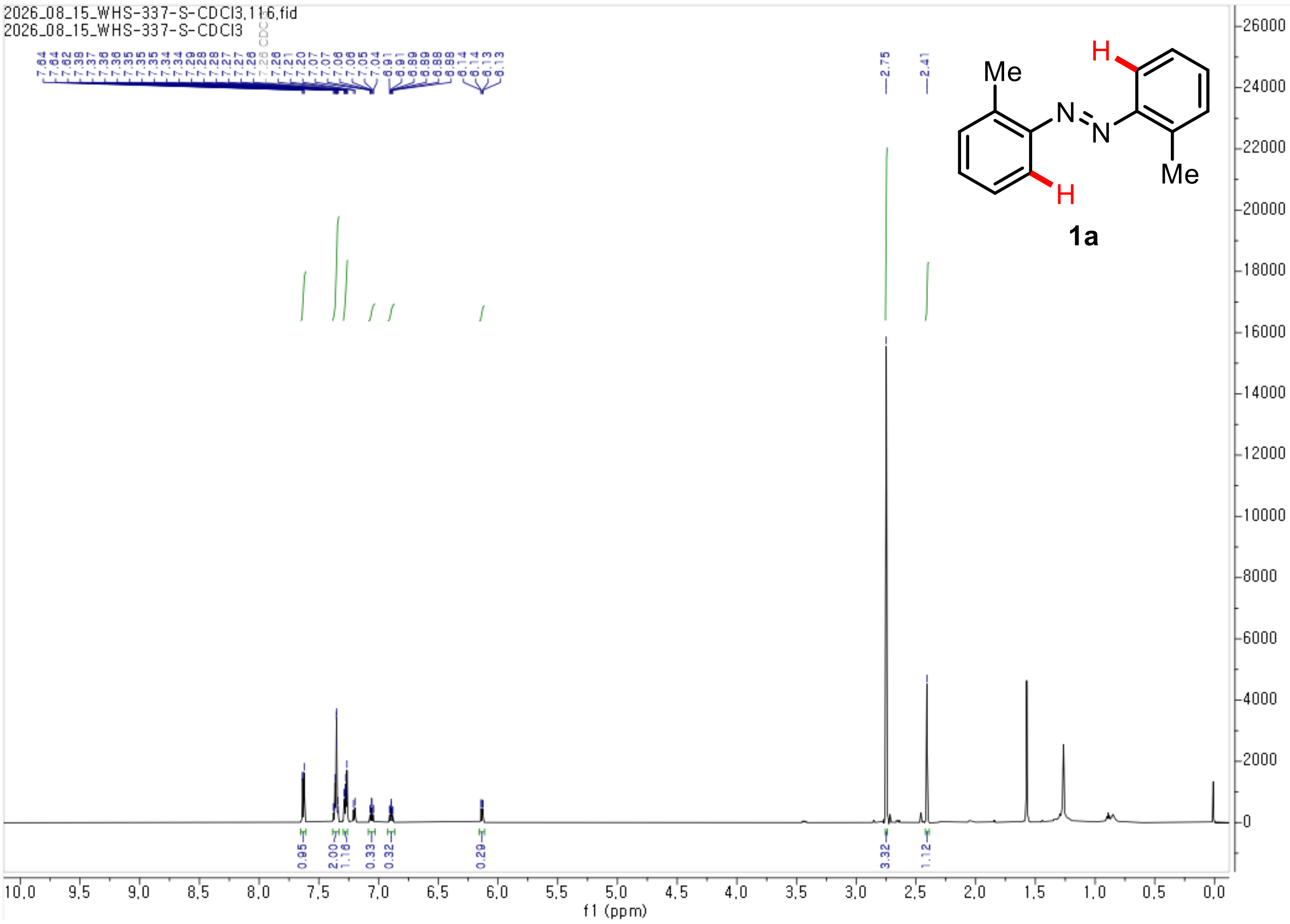
2026_08_15_WHS-337-S-CDCl3,11,fid
2026_08_15_WHS-337-S-CDCl3
Me
H
N
N
H
Me
1a
f1 (ppm)

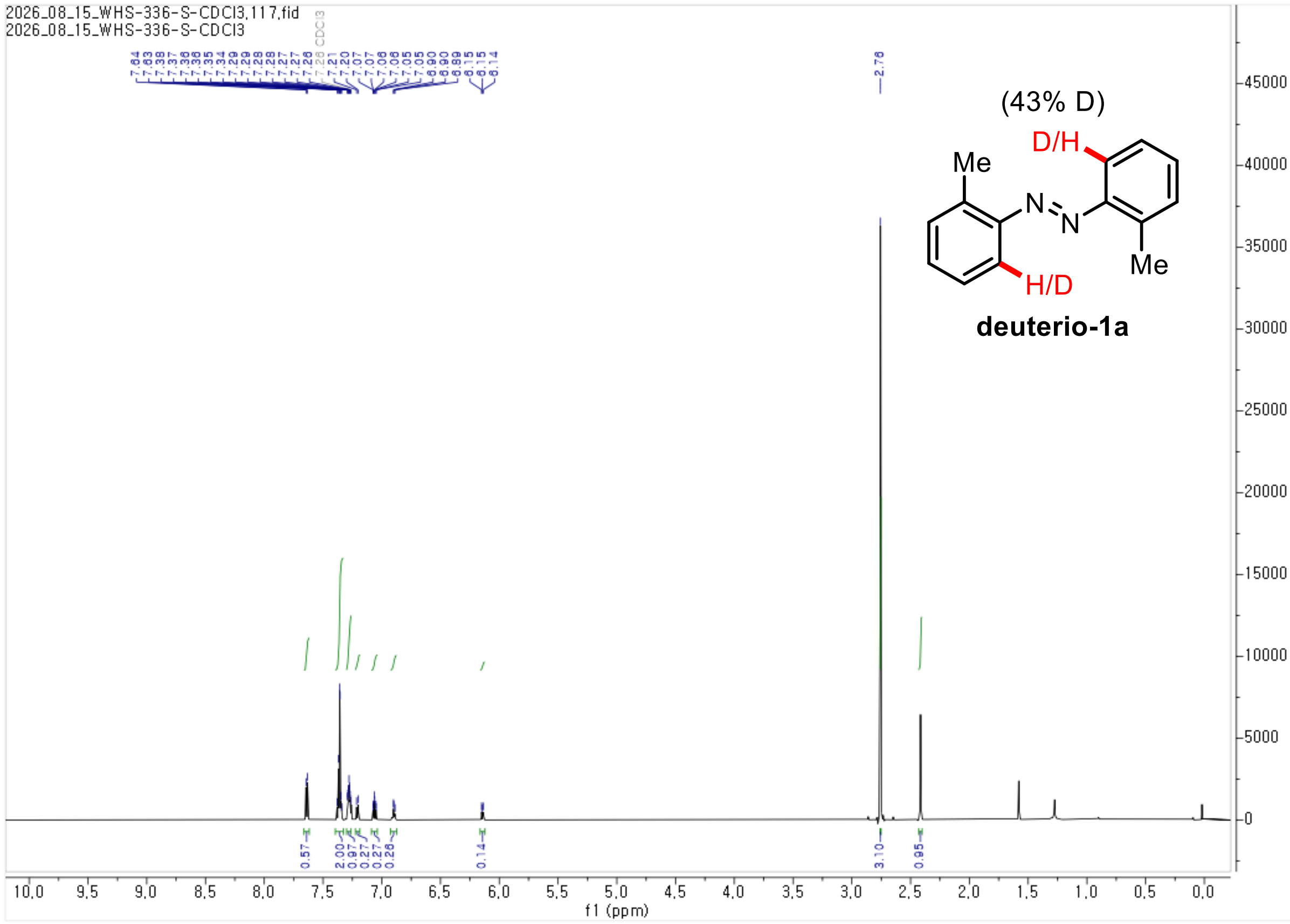
2026_08_15_WHS-336-S-CDCl3.117.fid
2026_08_15_WHS-336-S-CDCl3
(43% D)
D/H
H/D
Me
Me
N
N
deuterio-1a
f1 (ppm)

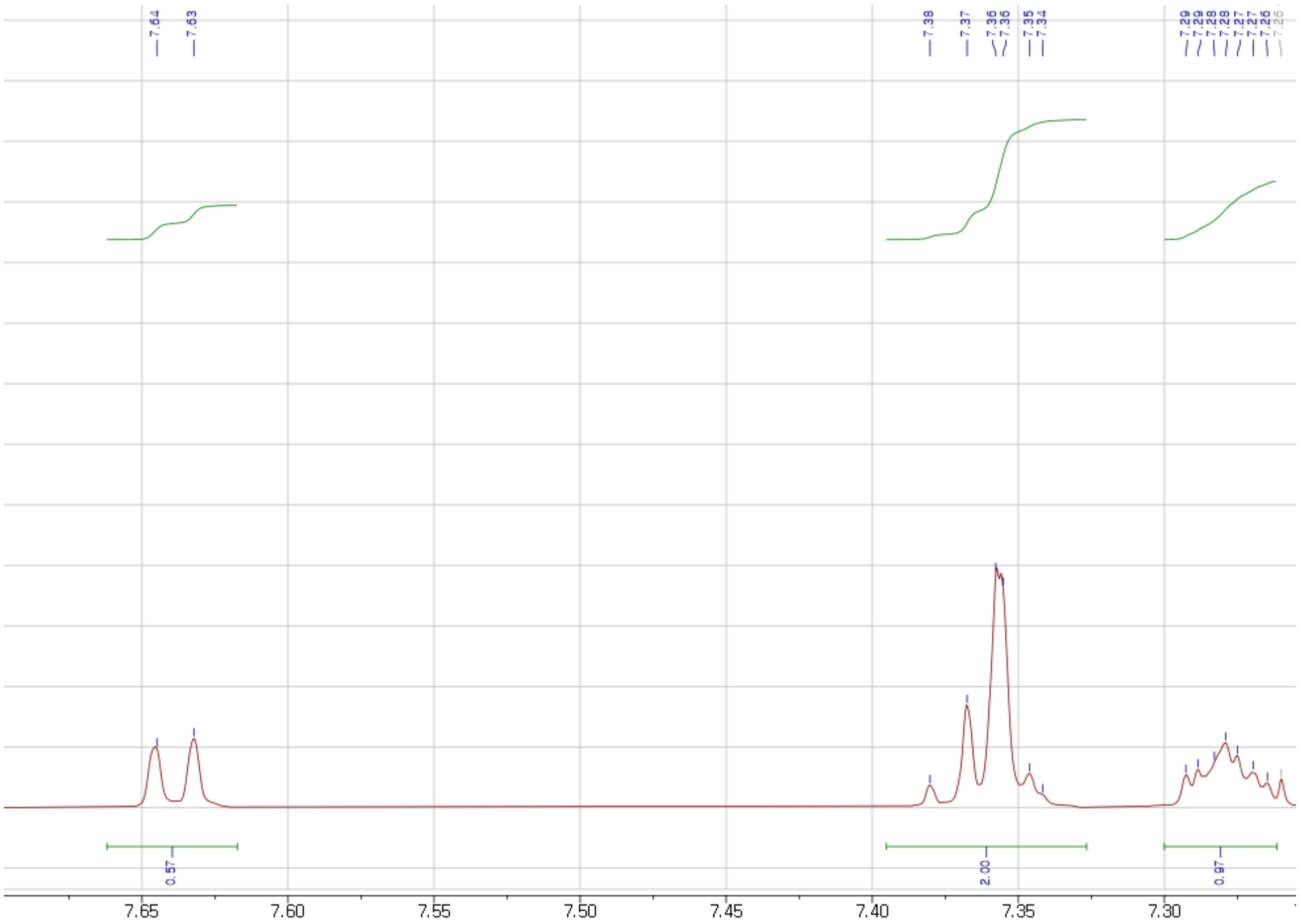

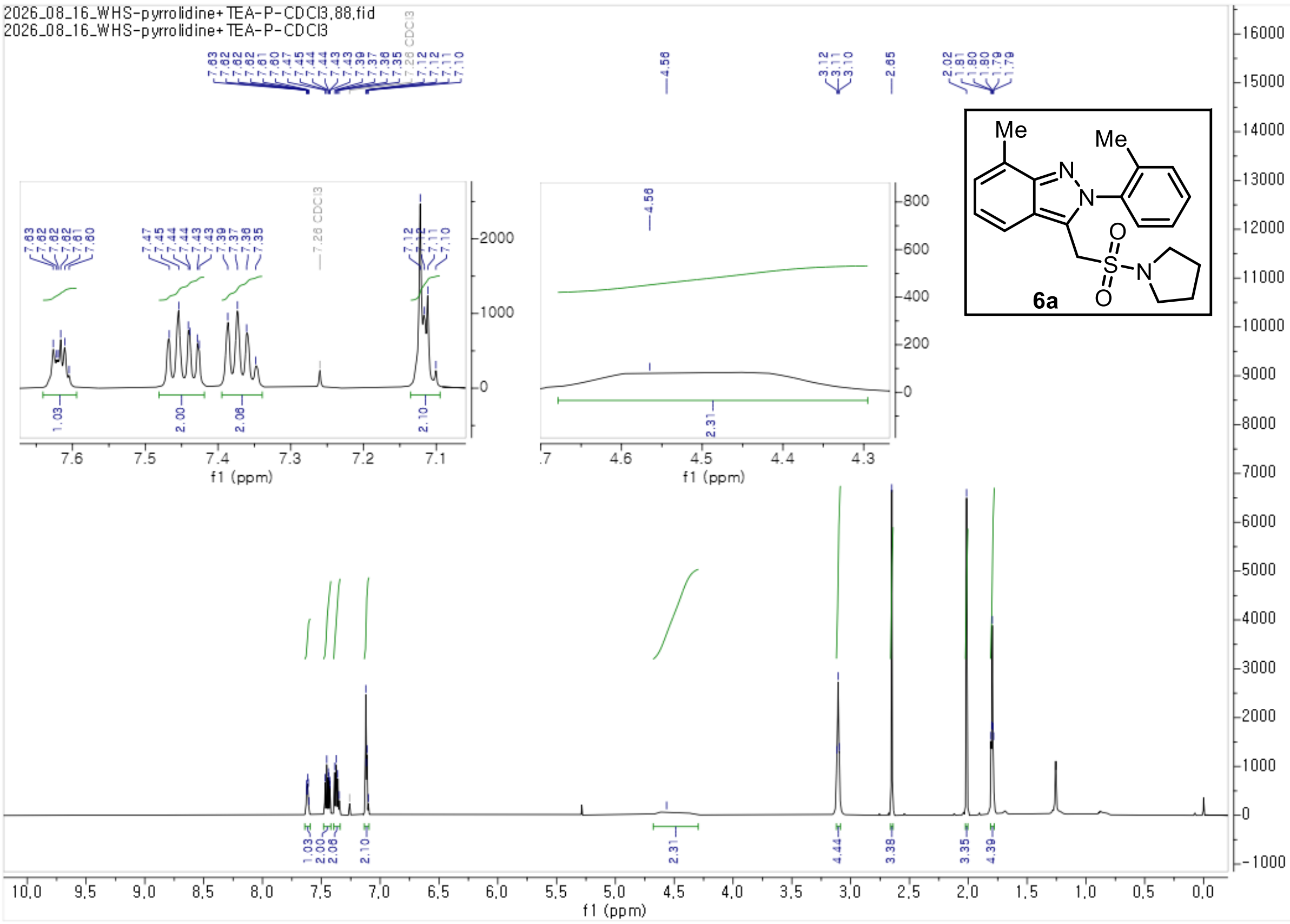

2026_08_16_WHS-pyrrolidine+TEA-P-CDCl3,88.fid
2026_08_16_WHS-pyrrolidine+TEA-P-CDCl3
Me
Me
N
N
O
S
O
N
6a
f1 (ppm)

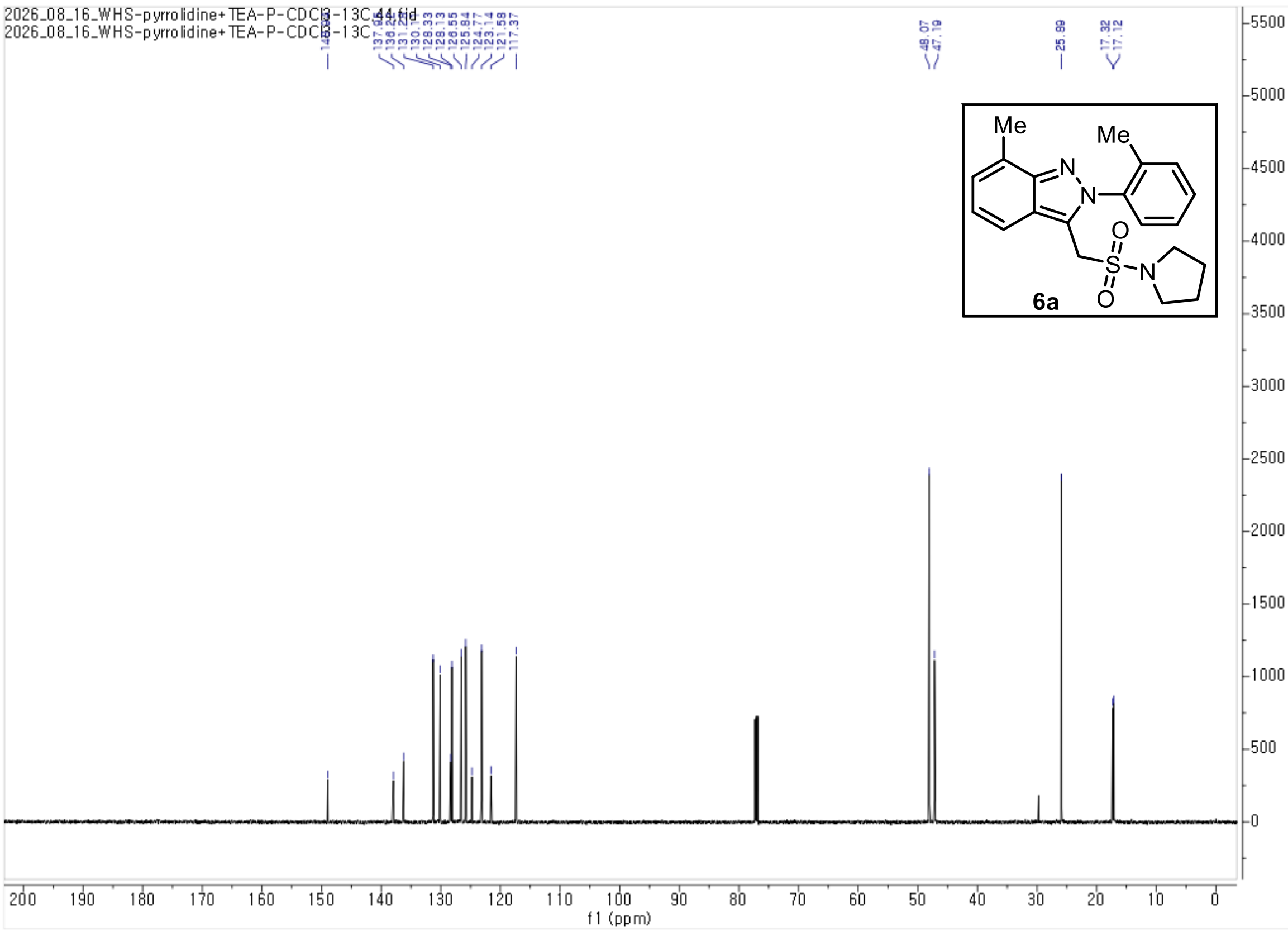

2026_08_16_WHS-pyrrolidine+TEA-P-CDCl3-13C.44.fid
2026_08_16_WHS-pyrrolidine+TEA-P-CDCl3-13C
48.07
47.19
25.89
17.32
17.12
Me
Me
N
N
O
S
O
N
6a
f1 (ppm)

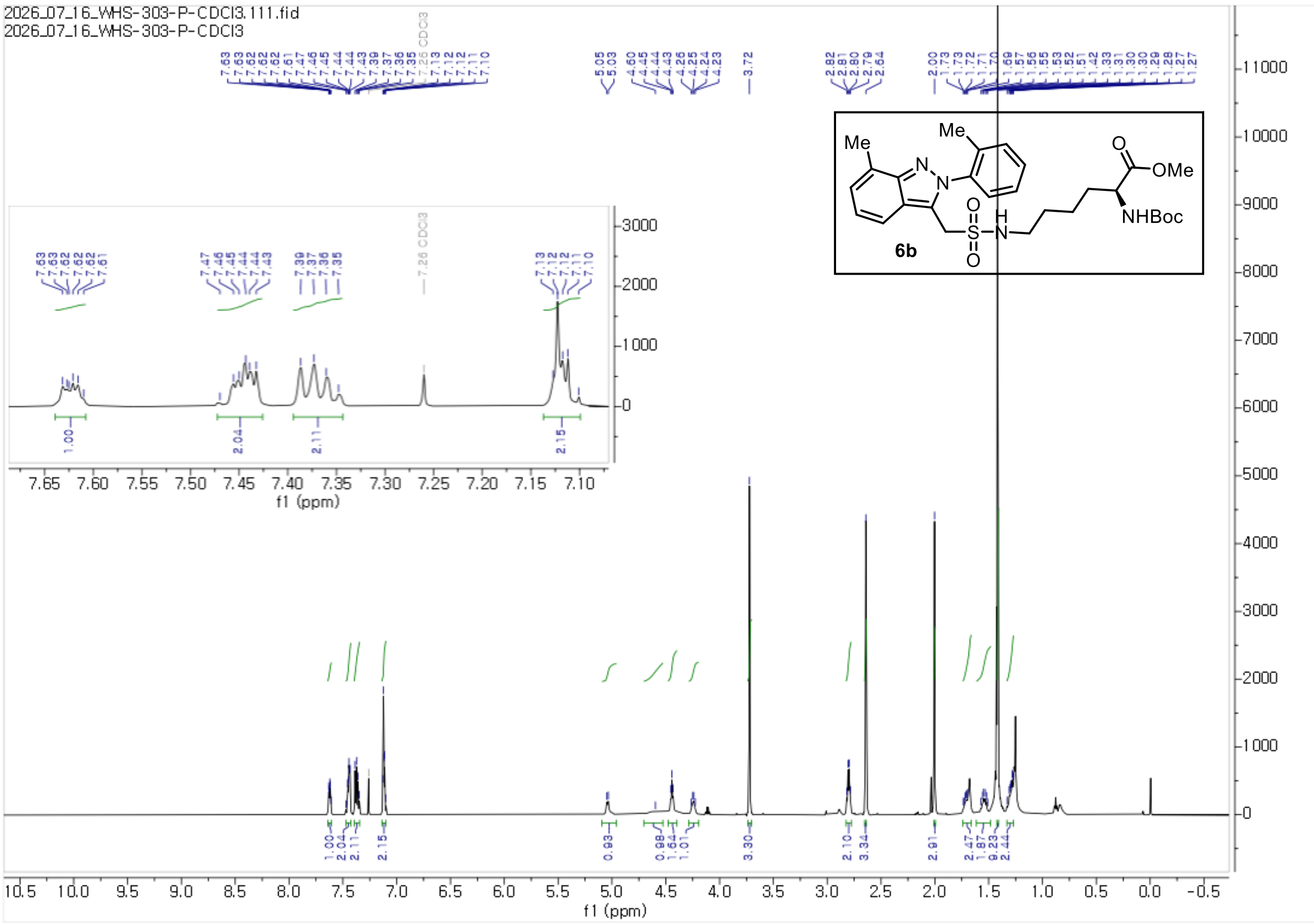

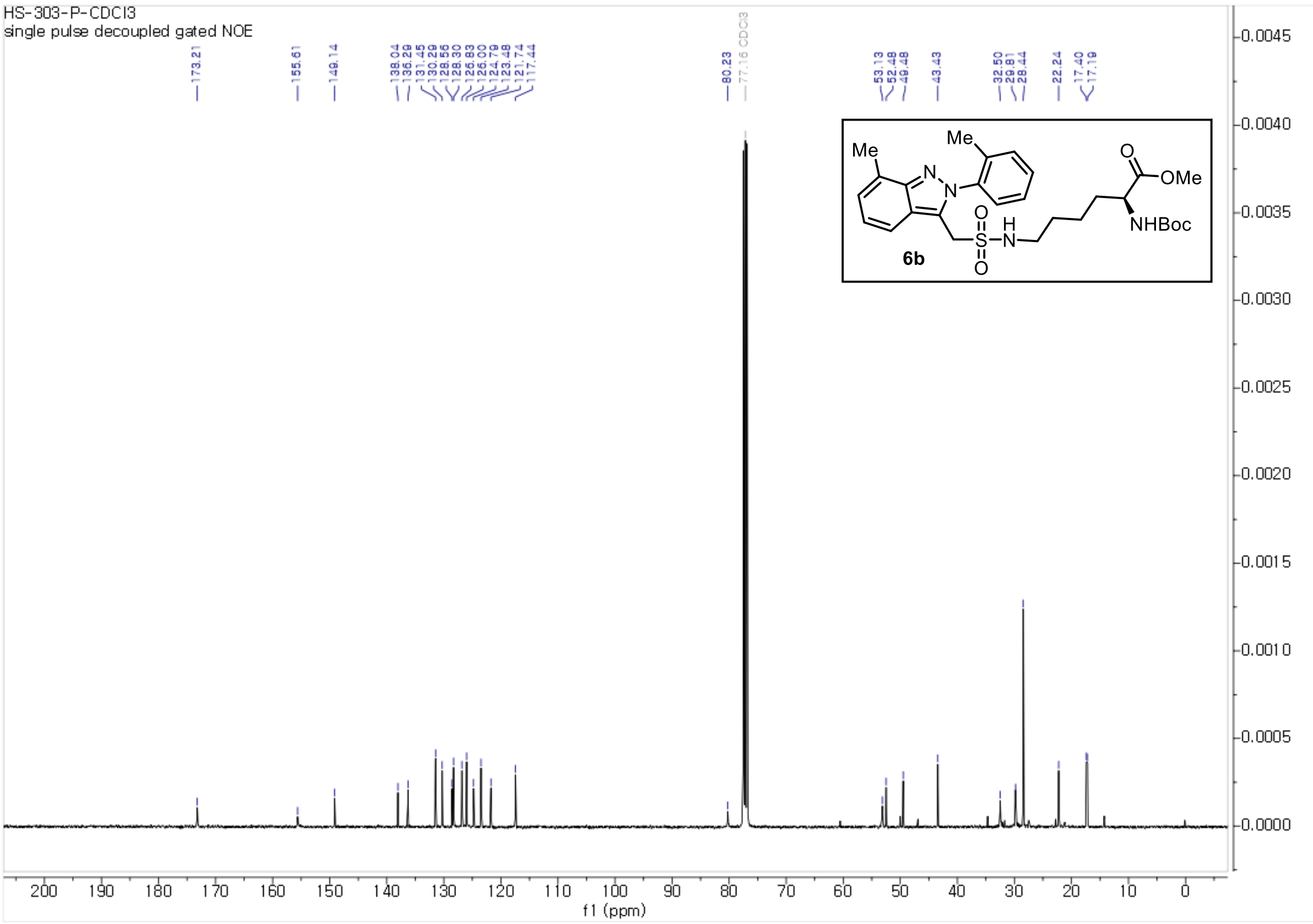

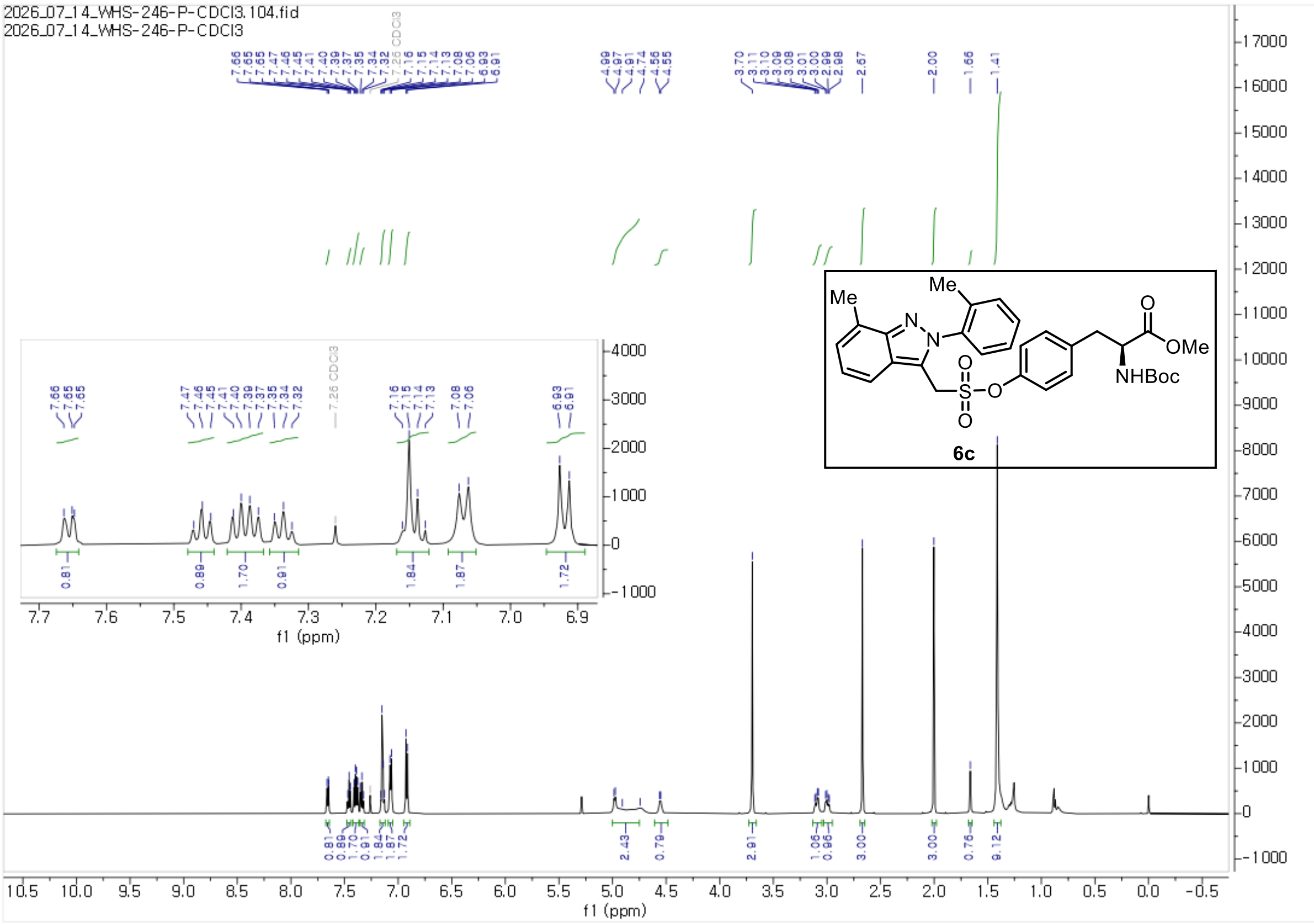
2026_07_14_WHS-246-P-CDCl3.104.fid
2026_07_14_WHS-246-P-CDCl3
Me
Me
N
N
O
OMe
NHBoc
S
O
6c
f1 (ppm)

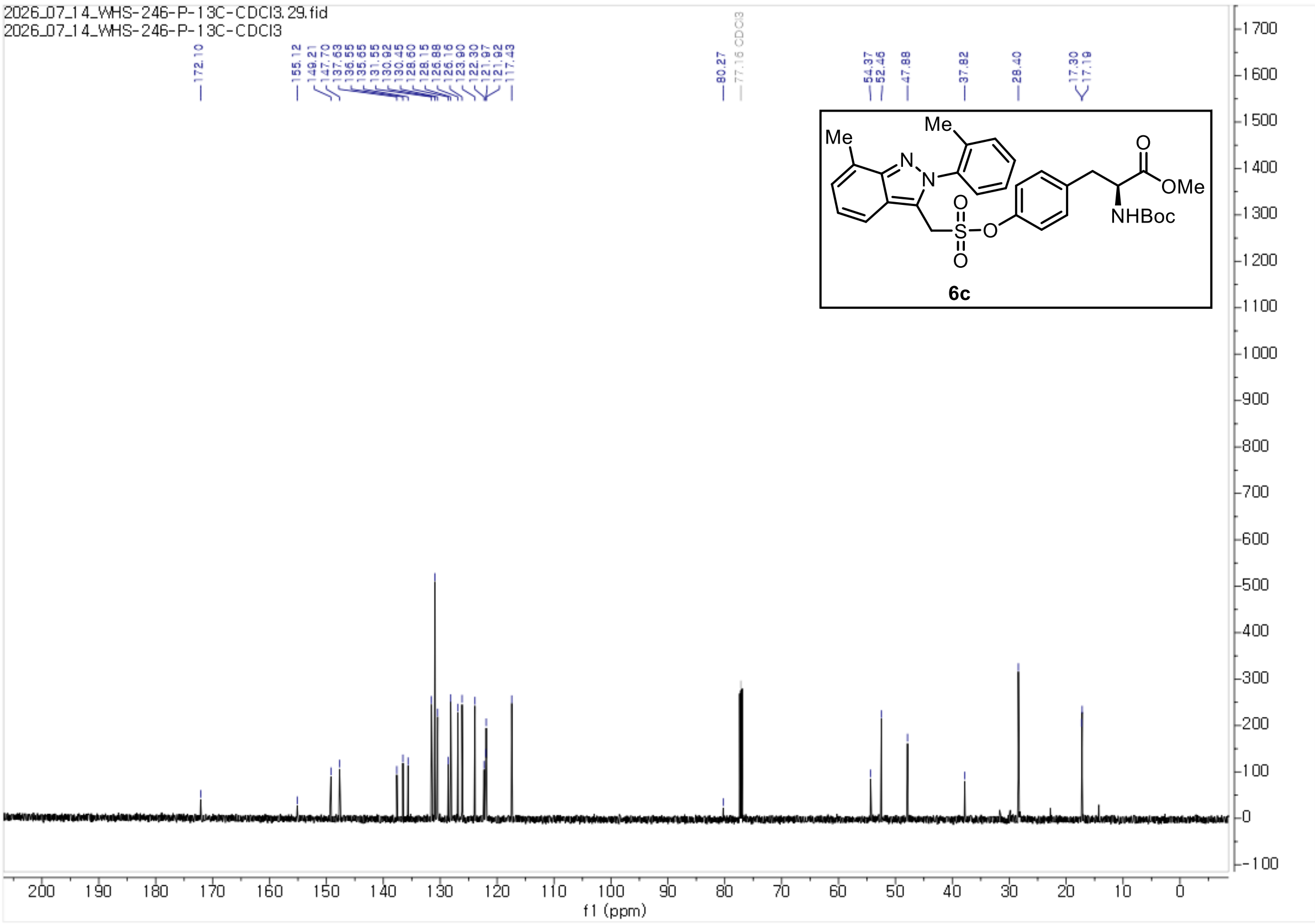
2026_07_14_WHS-246-P-13C-CDCl3.29.fid
2026_07_14_WHS-246-P-13C-CDCl3
Me
Me
N
N
O
OMe
NHBoc
S
O
6c
f1 (ppm)

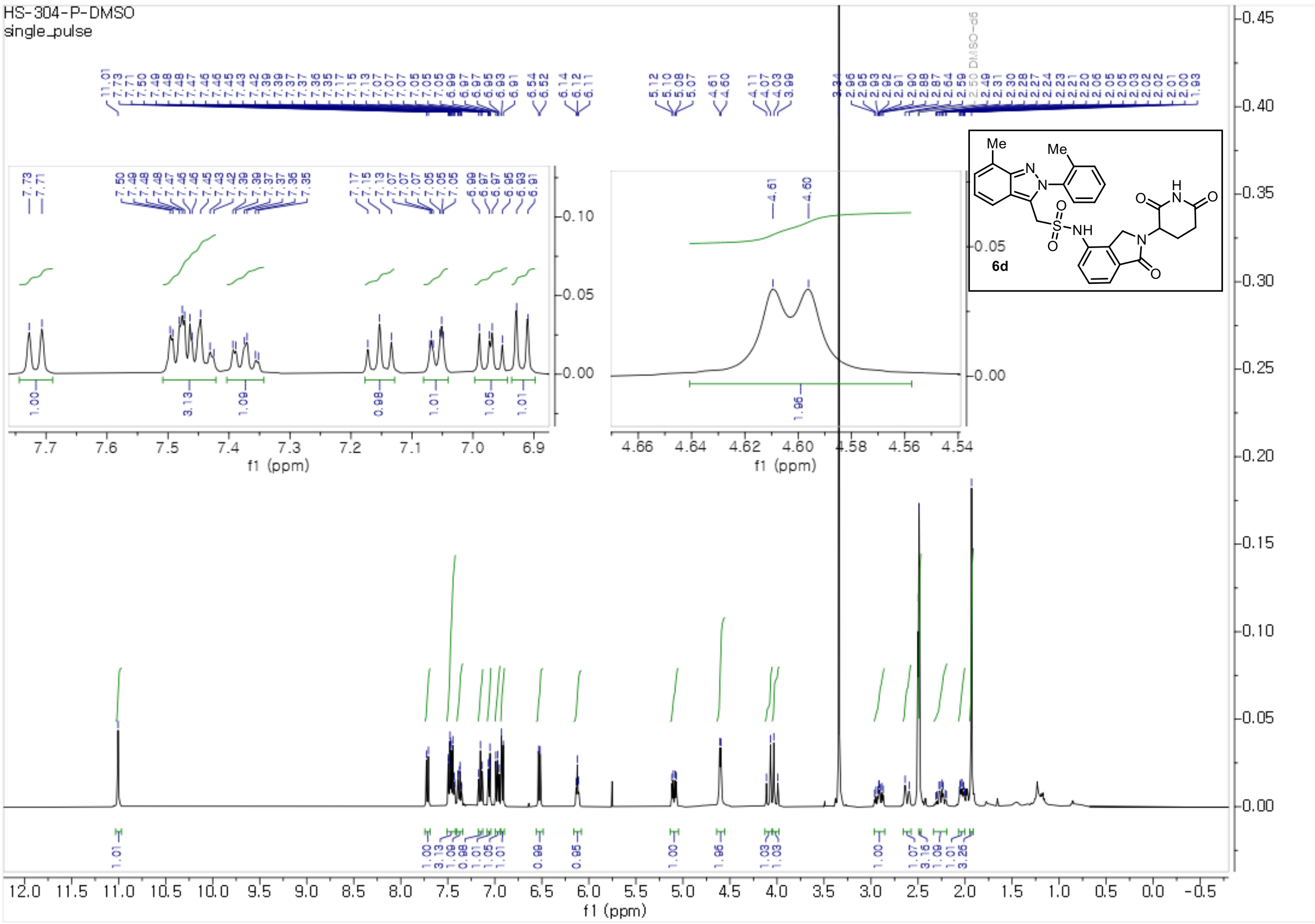

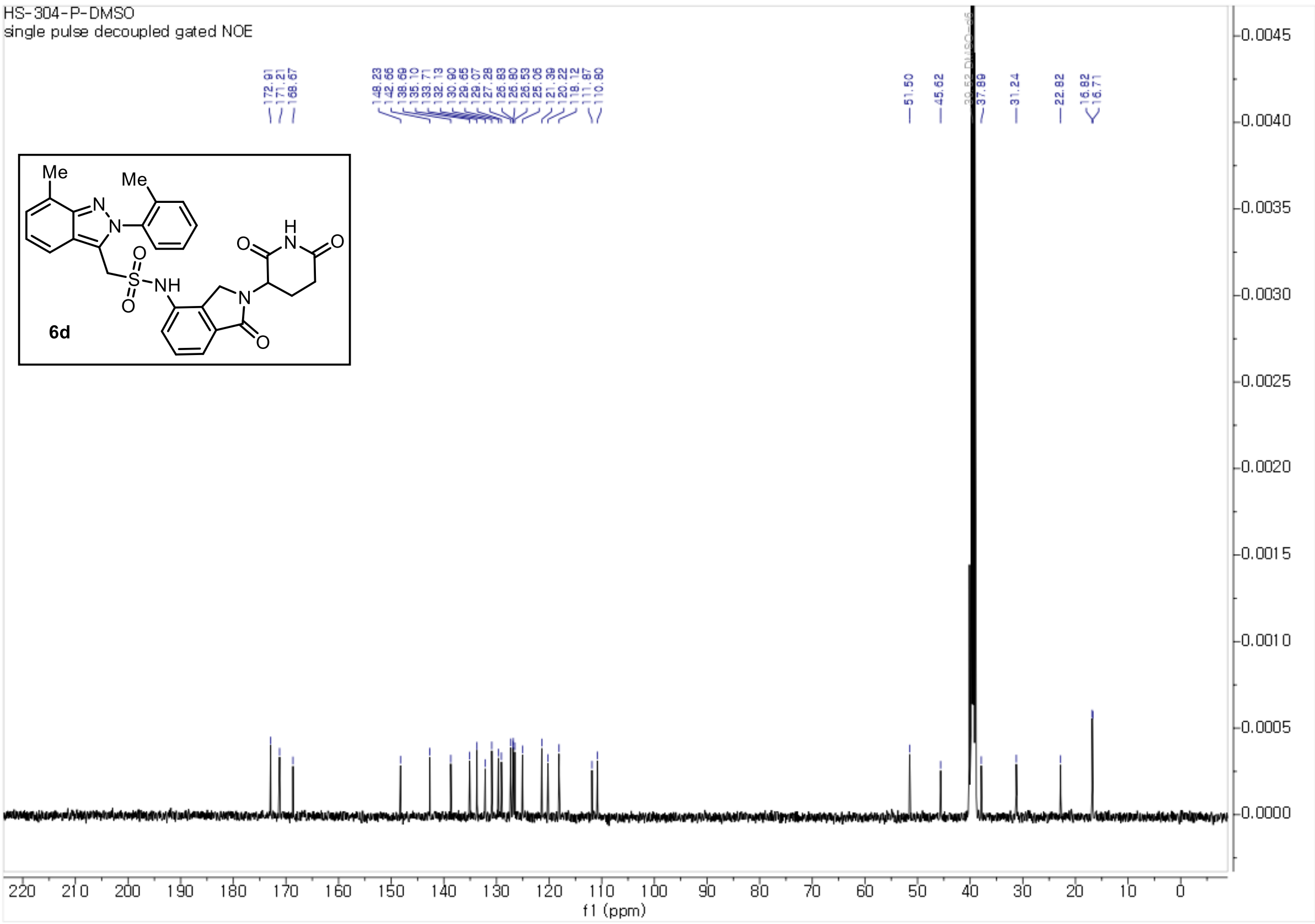

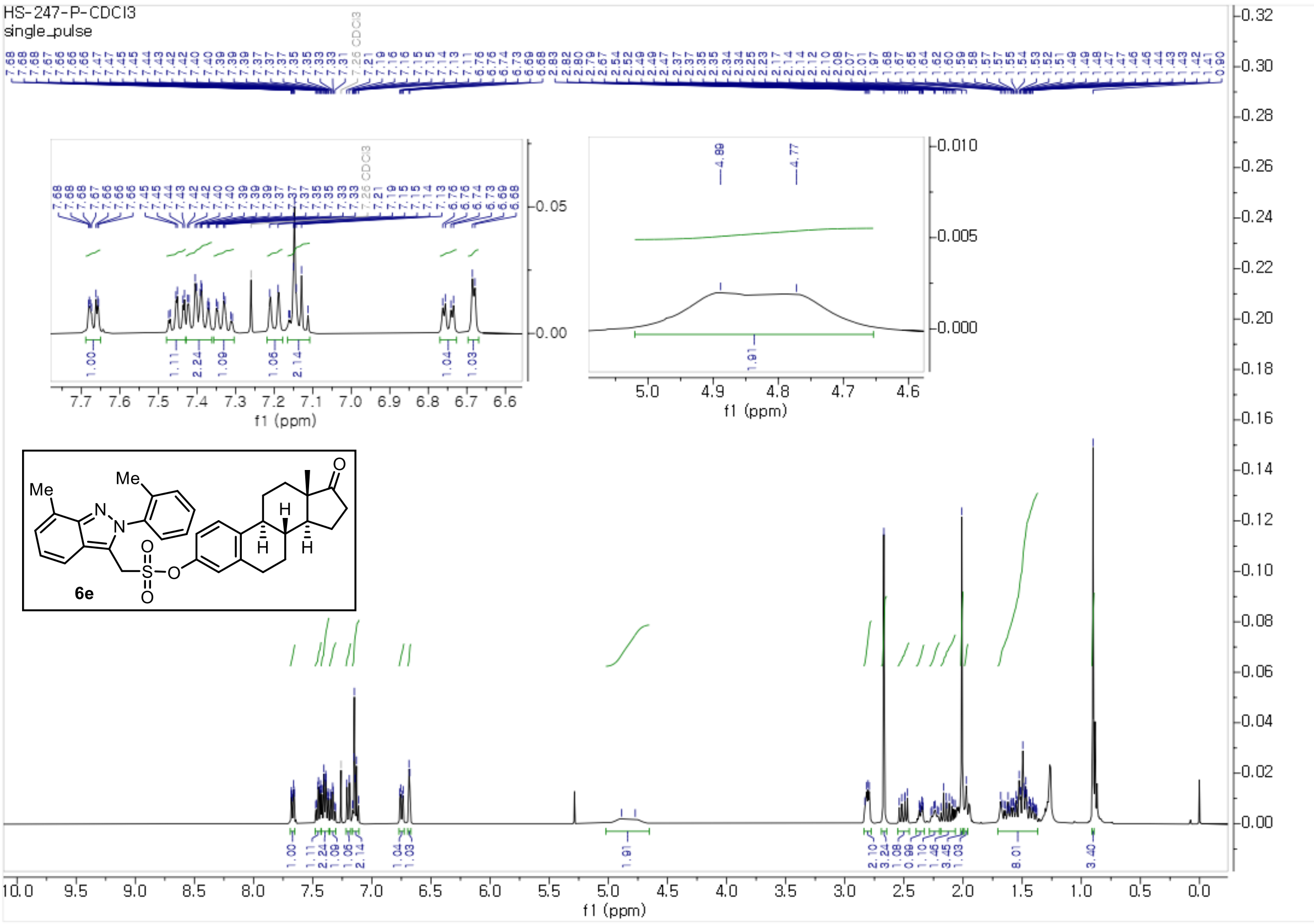

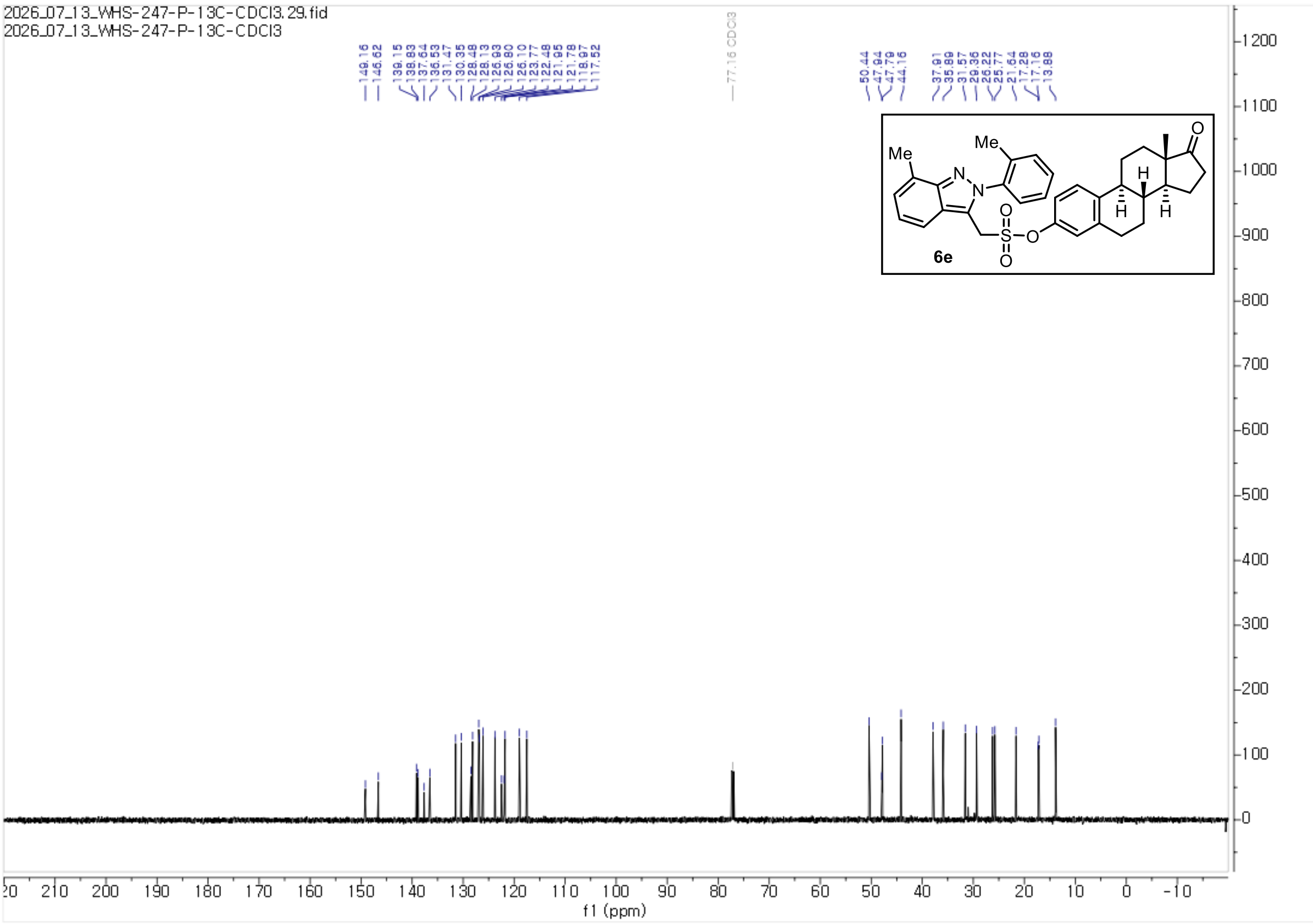

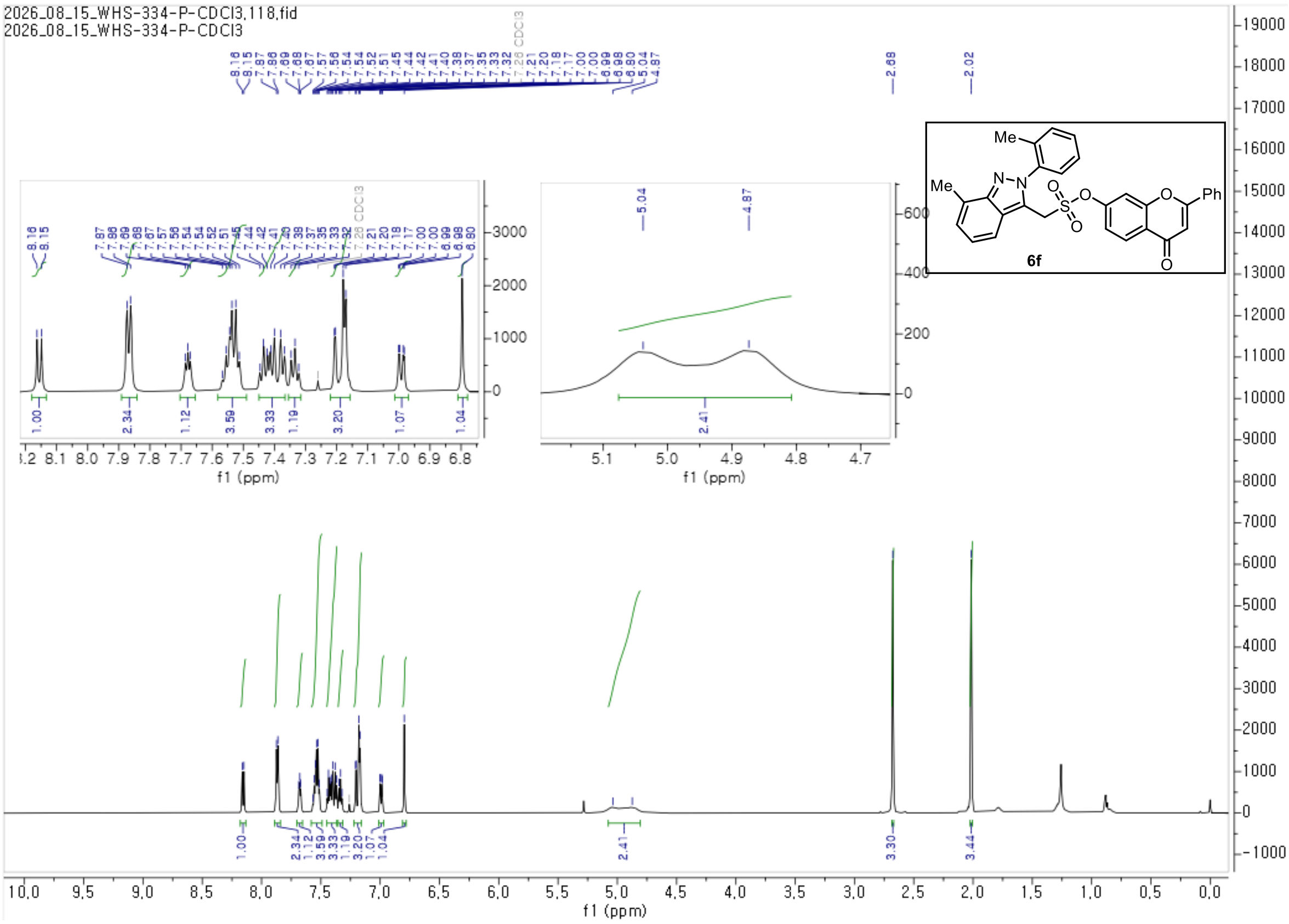
2026_08_15_WHS-334-P-CDCl3,118,fid
2026_08_15_WHS-334-P-CDCl3
Me
Me
N
N
S
O
O
O
O
Ph
O
6f
f1 (ppm)

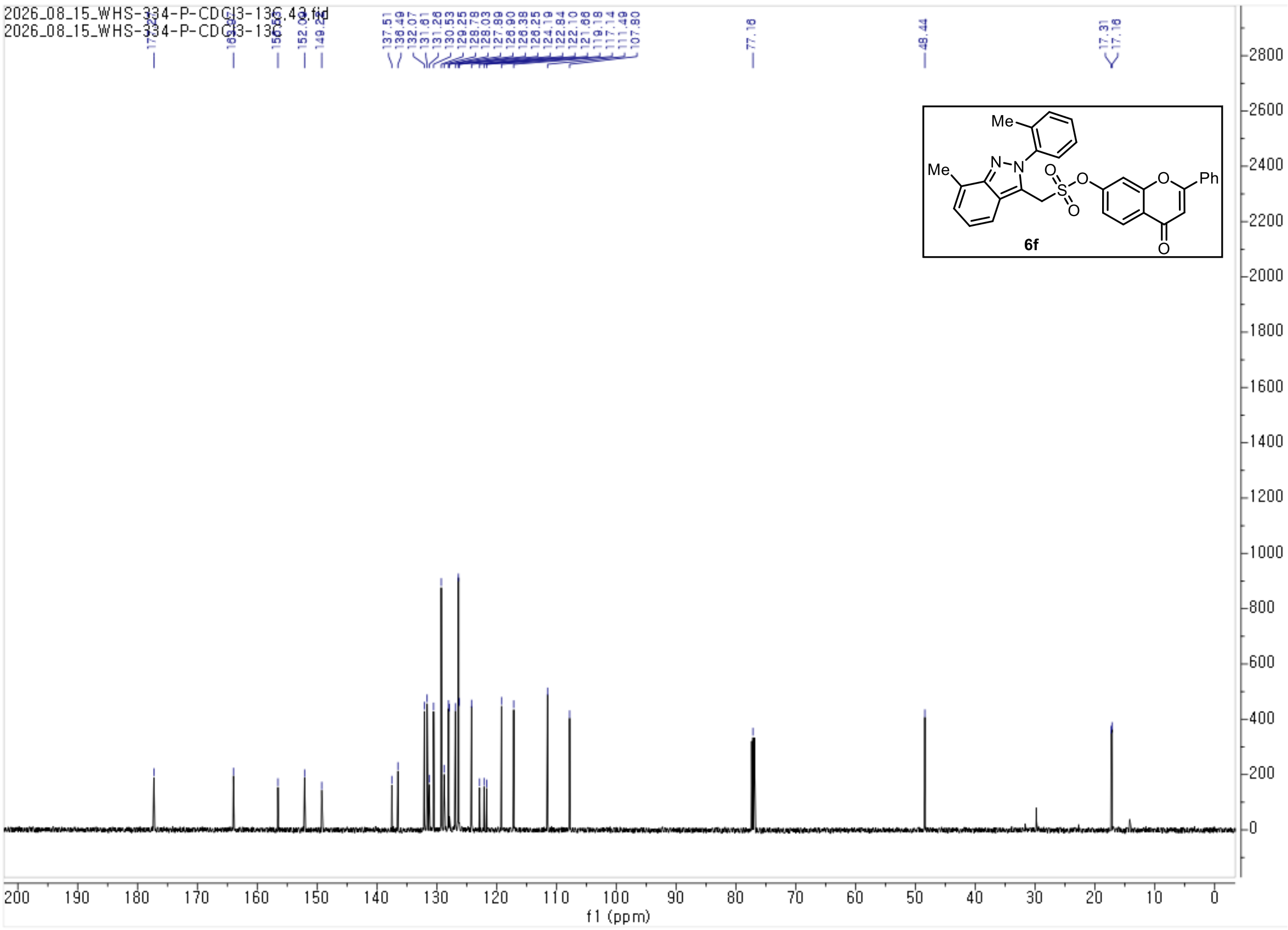
Me
Me
N
N
S
O
O
O
O
Ph
O
6f
f1 (ppm)

9. HR-MS analytical data of the products

Compound 3a

[Elemental Composition] Page: 1
Data : EI-1-HS-209-002 Date : 03-Jul-2026 15:23
Sample: High resolution GC Mass (JMS-700)
Note : SMLab-Mass Analysis & Research Center
Inlet : Direct Ion Mode : EI+
RT : 0.29 min Scan#: (9,10)+12
Elements : C 17/0, H 16/0, F 1/0, N 2/0, O 3/0, S 2/0
Mass Tolerance : 1000ppm, 3mmu if m/z < 3, 5mmu if m/z > 5
Unsaturation (U.S.) : -0.5 - 80.0

Observed m/z	Int%	Err[ppm / mmu]	U.S.	Composition
318.0834	100.0	-1.4 / -0.5	11.0	C 16 H 15 F N 2 O 2 S

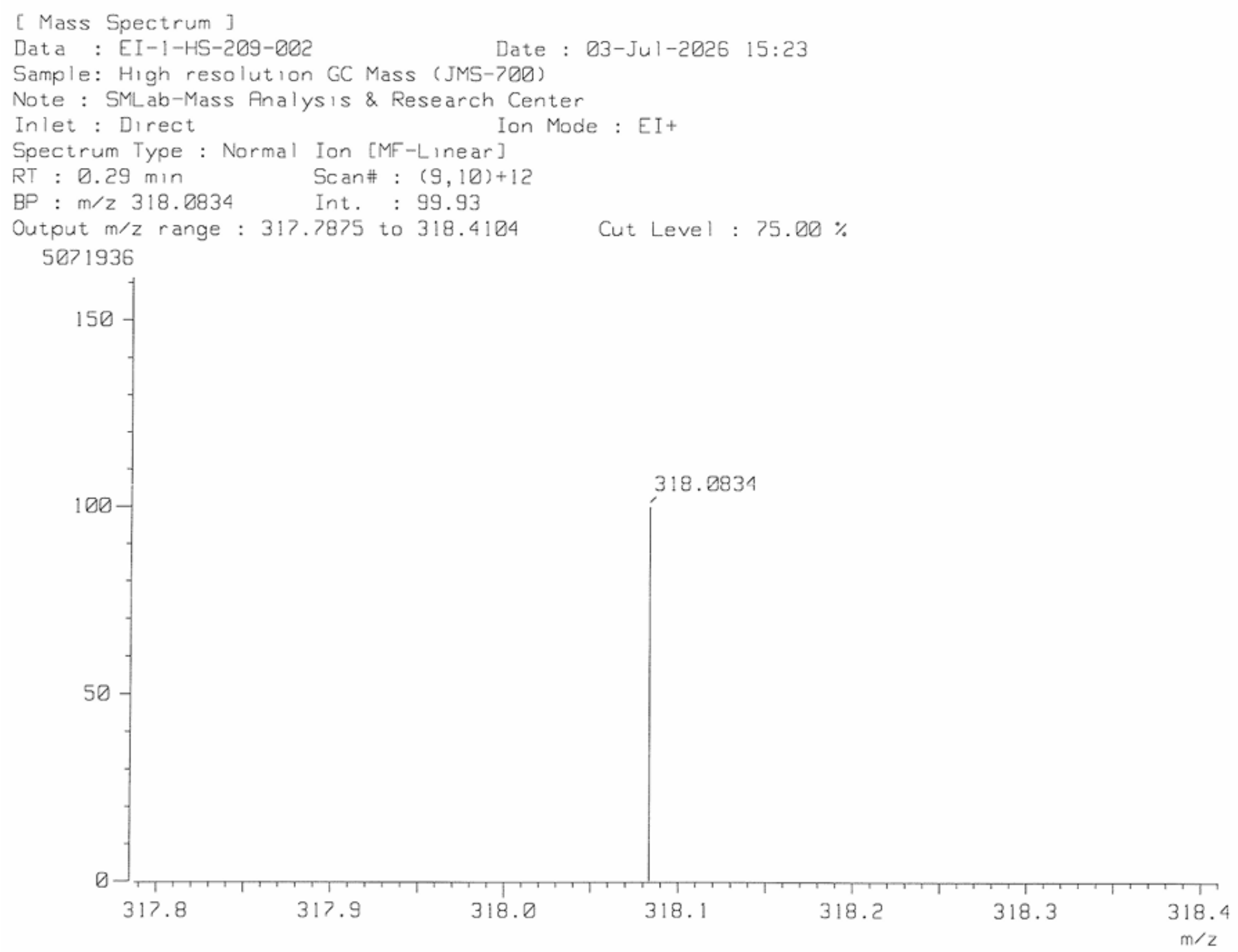

Compound 3b

[Elemental Composition] Page: 1
Data : EI-HS-214-002 Date : 08-Jul-2026 17:54
Sample: High resolution GC Mass (JMS-700)
Note : SMLab-Mass Analysis & Research Center
Inlet : Direct Ion Mode : EI+
RT : 0.47 min Scan#: 15
Elements : C 20/0, H 20/0, F 1/0, N 4/0, O 2/0, S 1/0
Mass Tolerance : 1000ppm, 3mmu if m/z < 3, 5mmu if m/z > 5
Unsaturation (U.S.) : -0.5 - 80.0

Observed m/z	Int%	Err[ppm / mmu]	U.S.	Composition
346.1146	100.0	-1.5 / -0.5	11.0	C 18 H 19 F N 2 O 2 S

[Mass Spectrum]
Data : EI-HS-214-002 Date : 08-Jul-2026 17:54
Sample: High resolution GC Mass (JMS-700)
Note : SMLab-Mass Analysis & Research Center
Inlet : Direct Ion Mode : EI+
Spectrum Type : Normal Ion [MF-Linear]
RT : 0.47 min Scan# : 15
BP : m/z 346.1146 Int. : 50.05
Output m/z range : 345.8036 to 346.4200 Cut Level : 73.53 %

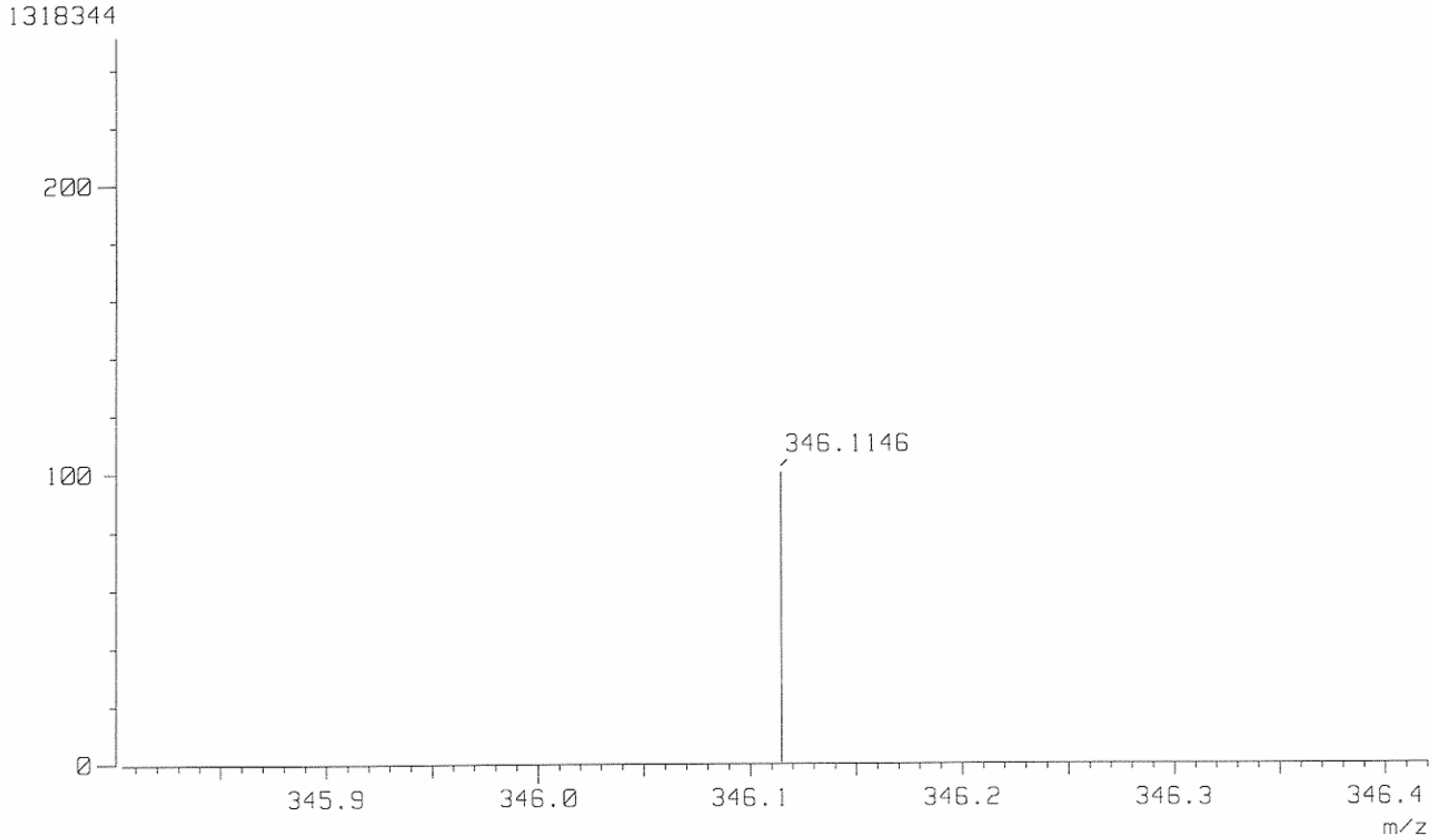

Compound 3c

[Elemental Composition]
Data : EI-28-Code-LR-38-F3-019   Date : 04-Jul-2026 09:57   Page: 1
Sample: High resolution GC Mass (JMS-700)
Note : SMLab-Mass Analysis & Research Center
Inlet : Direct   Ion Mode : EI+
RT : 0.60 min   Scan#: 19
Elements : C 15/0, H 14/0, F 3/0, N 2/0, O 3/0, S 1/0
Mass Tolerance : 1000ppm, 3mmu if m/z < 3, 5mmu if m/z > 5
Unsaturation (U.S.) : -0.5 - 80.0

Observed m/z	Int%	Err[ppm / mmu]	U.S.	Composition
326.0336	100.0	-0.4 / -0.1	11.0	C 14 H 9 F 3 N 2 O 2 S

[Mass Spectrum]
Data : EI-28-Code-LR-38-F3-019   Date : 04-Jul-2026 09:57
Sample: High resolution GC Mass (JMS-700)
Note : SMLab-Mass Analysis & Research Center
Inlet : Direct   Ion Mode : EI+
Spectrum Type : Normal Ion [MF-Linear]
RT : 0.60 min   Scan# : 19
BP : m/z 326.0336   Int. : 35.99
Output m/z range : 325.7115 to 326.3140   Cut Level : 78.68 %

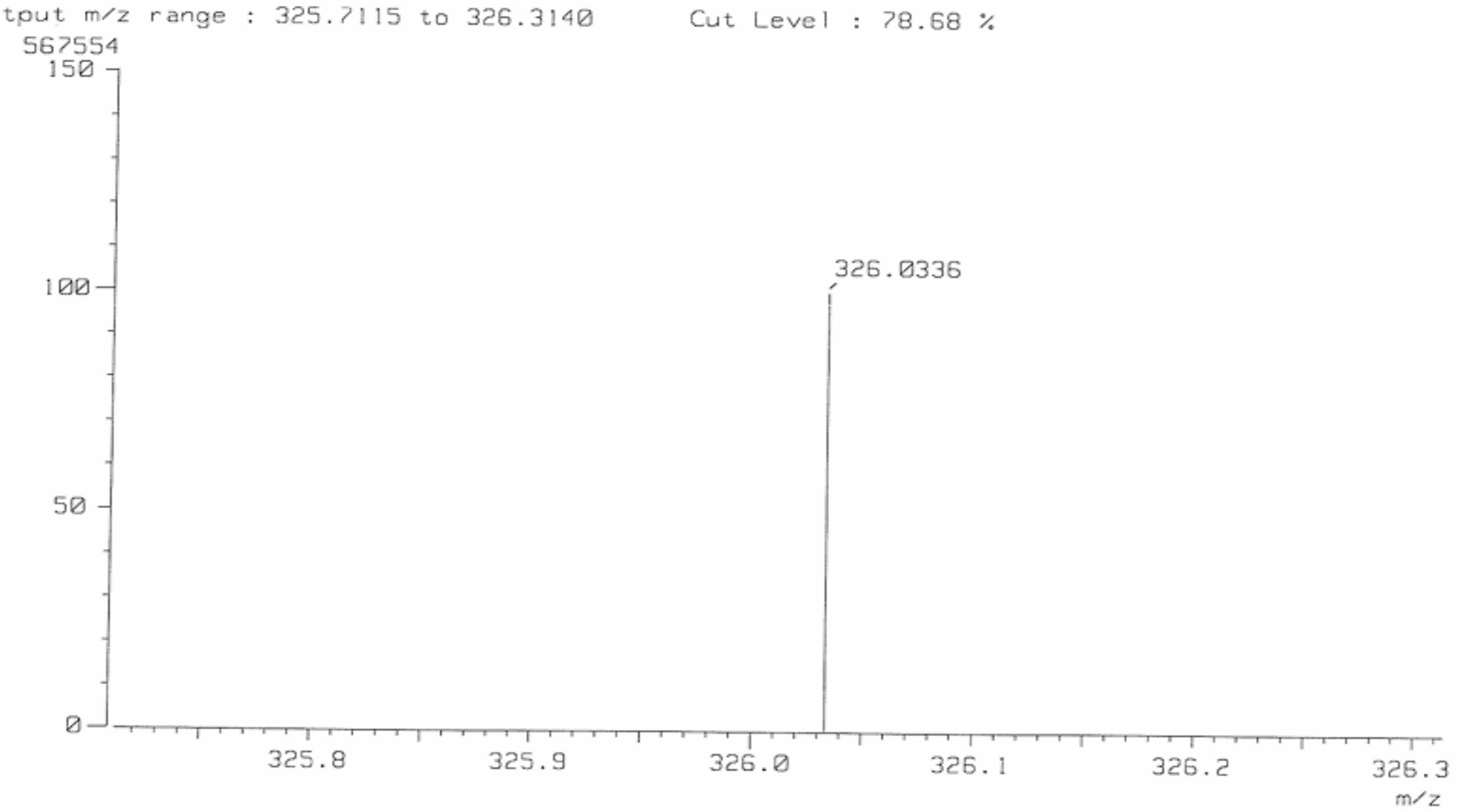

Compound 3d

```
[ Elemental Composition ]                                        Page: 1
Data   : EI-36-Code-LR-103-F3-005     Date : 04-Jul-2026 09:35
Sample: High resolution GC Mass (JMS-700)
Note : SMLab-Mass Analysis & Research Center
Inlet : Direct                        Ion Mode : EI+
RT : 1.17 min                         Scan#: (28,44)
Elements : C 15/0, H 10/0, Cl 2/0, F 1/0, N 2/0, O 3/0, S 1/0
Mass Tolerance       : 1000ppm, 3mmu if m/z < 3, 5mmu if m/z > 5
Unsaturation (U.S.) : -0.5 - 80.0

Observed m/z  Int%   Err[ppm / mmu]    U.S.  Composition
  357.9742   100.0     -0.9 /  -0.3   11.0  C 14 H 9 Cl 2 F N 2 O 2 S
```

```
[ Mass Spectrum ]
Data   : EI-36-Code-LR-103-F3-005     Date : 04-Jul-2026 09:35
Sample: High resolution GC Mass (JMS-700)
Note : SMLab-Mass Analysis & Research Center
Inlet : Direct                        Ion Mode : EI+
Spectrum Type : Normal Ion [MF-Linear]
RT : 1.17 min           Scan# : (28,44)
BP : m/z 357.9742       Int.   : 8.45
Output m/z range : 357.6682 to 358.2556      Cut Level : 47.84 %
```

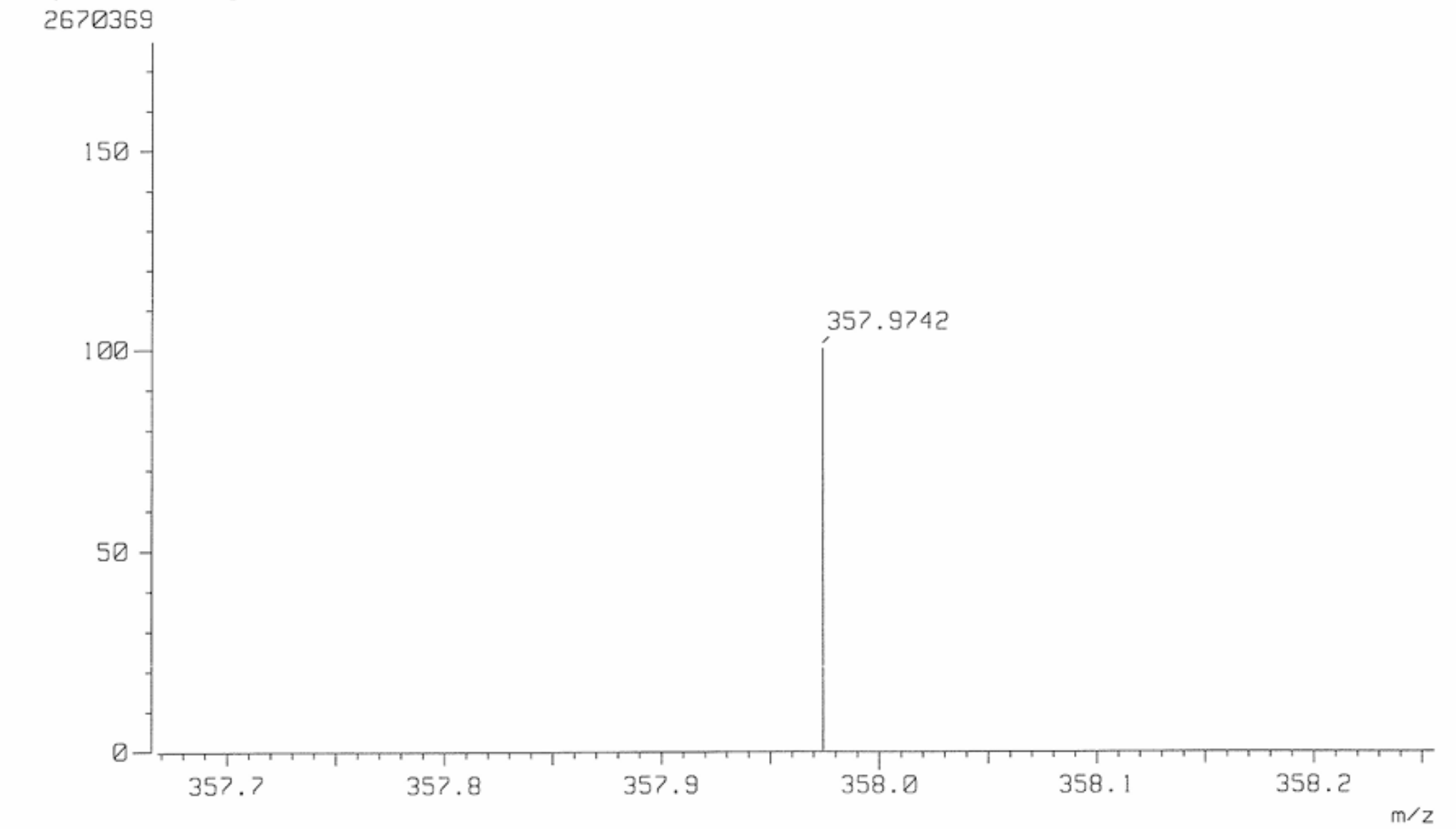

Compound 3e

```
[ Elemental Composition ]                                              Page: 1
Data   : EI-12-HS-250-025                 Date : 03-Jul-2026 16:04
Sample: High resolution GC Mass (JMS-700)
Note : SMLab-Mass Analysis & Research Center
Inlet : Direct                            Ion Mode : EI+
RT : 2.10 min                             Scan#: 64
Elements : C 20/0, H 20/0, F 1/0, N 2/0, O 3/0, S 1/0
Mass Tolerance        : 1000ppm, 3mmu if m/z < 3, 5mmu if m/z > 5
Unsaturation (U.S.) : -0.5 - 80.0

Observed m/z Int%   Err[ppm / mmu]    U.S.  Composition
  346.1152   100.0     +0.1 /  +0.0   11.0  C 18 H 19 F N 2 O 2 S
```

```
[ Mass Spectrum ]
Data   : EI-12-HS-250-025              Date : 03-Jul-2026 16:04
Sample: High resolution GC Mass (JMS-700)
Note : SMLab-Mass Analysis & Research Center
Inlet : Direct                         Ion Mode : EI+
Spectrum Type : Normal Ion [MF-Linear]
RT : 2.10 min           Scan# : 64
BP : m/z 346.1152       Int.  : 71.61
Output m/z range : 345.7537 to 346.4530      Cut Level : 71.32 %
```

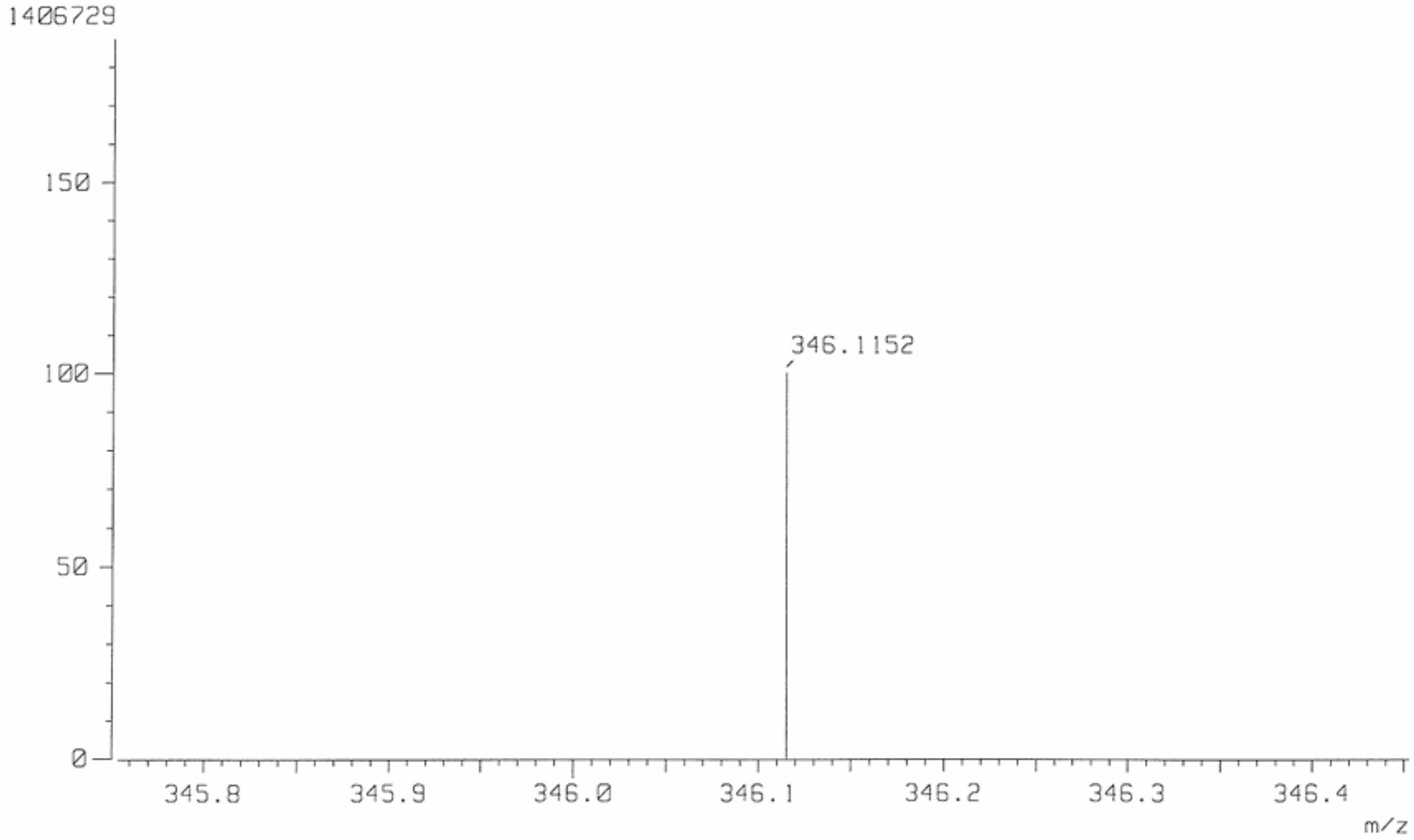

Compound 3f

[Elemental Composition]   Page: 1
Data : EI-5-HS-220-008   Date : 03-Jul-2026 15:34
Sample: High resolution GC Mass (JMS-700)
Note : SMLab-Mass Analysis & Research Center
Inlet : Direct   Ion Mode : EI+
RT : 1.04 min   Scan#: (31,33)
Elements : C 20/0, H 25/0, F 1/0, N 2/0, O 2/0, S 1/0
Mass Tolerance : 1000ppm, 3mmu if m/z < 3, 5mmu if m/z > 5
Unsaturation (U.S.) : -0.5 - 80.0

Observed m/z	Int%	Err[ppm / mmu]	U.S.	Composition
374.1465	23.8	+0.2 / +0.1	11.0	C 20 H 23 F N 2 O 2 S

[Mass Spectrum]
Data : EI-5-HS-220-008   Date : 03-Jul-2026 15:34
Sample: High resolution GC Mass (JMS-700)
Note : SMLab-Mass Analysis & Research Center
Inlet : Direct   Ion Mode : EI+
Spectrum Type : Normal Ion [MF-Linear]
RT : 1.04 min   Scan# : (31,33)
BP : m/z 317.9681   Int. : 96.88
Output m/z range : 373.8229 to 374.4768   Cut Level : 12.50 %

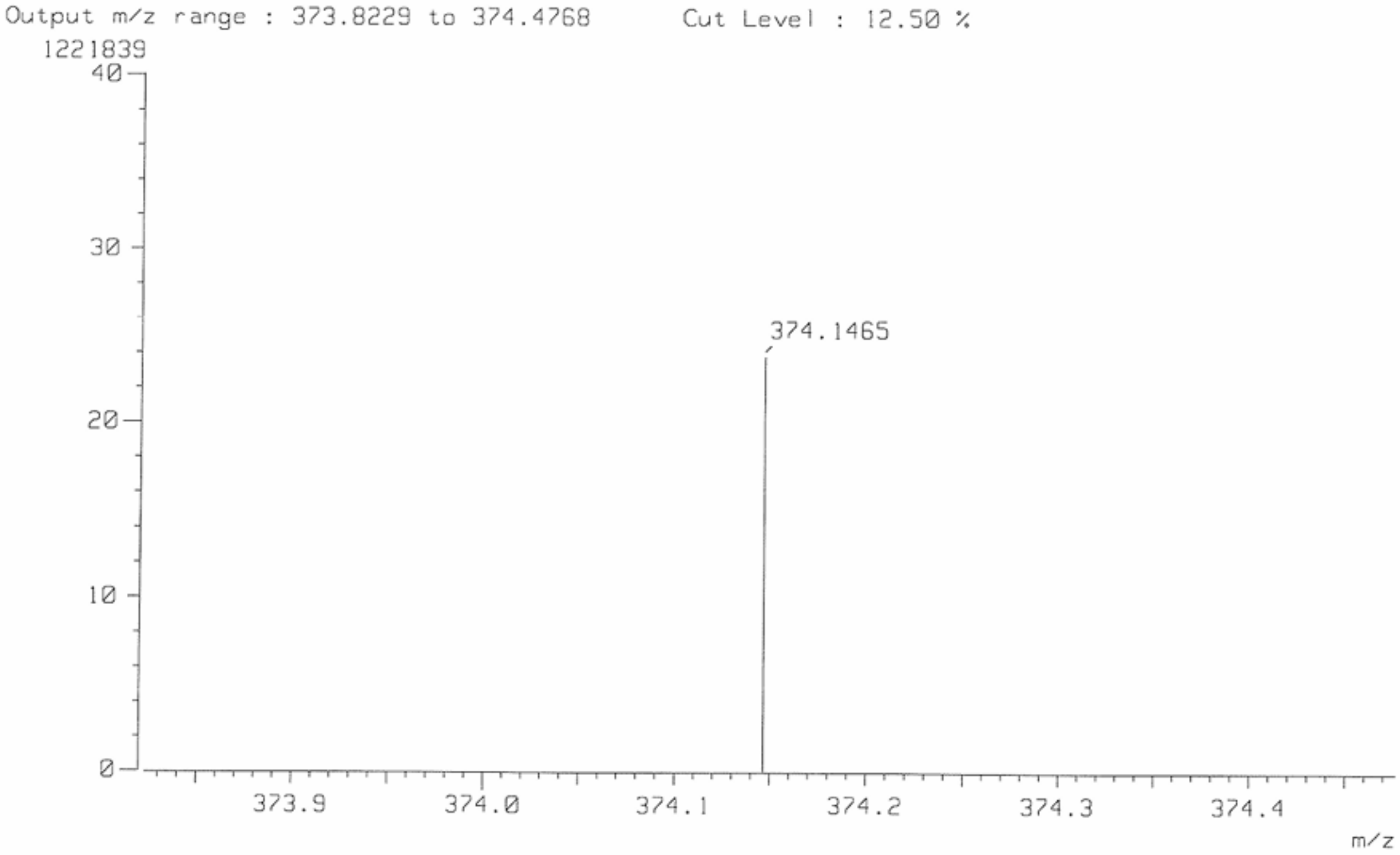

Compound 3g

```
[ Elemental Composition ]                                              Page: 1
Data   : EI-39-Code-LR-112-F2-020     Date : 04-Jul-2026 10:00
Sample: High resolution GC Mass (JMS-700)
Note : SMLab-Mass Analysis & Research Center
Inlet : Direct                        Ion Mode : EI+
RT : 1.57 min                         Scan#: 48
Elements : C 14/0, H 14/0, F 3/0, N 2/0, O 3/0, S 1/0
Mass Tolerance      : 1000ppm, 3mmu if m/z < 3, 5mmu if m/z > 5
Unsaturation (U.S.) : -0.5 - 80.0
```

Observed m/z	Int%	Err [ppm / mmu]	U.S.	Composition
326.0333	100.0	-1.2 / -0.4	11.0	C 14 H 9 F 3 N 2 O 2 S

```
[ Mass Spectrum ]
Data   : EI-39-Code-LR-112-F2-020     Date : 04-Jul-2026 10:00
Sample: High resolution GC Mass (JMS-700)
Note : SMLab-Mass Analysis & Research Center
Inlet : Direct                        Ion Mode : EI+
Spectrum Type : Normal Ion [MF-Linear]
RT : 1.57 min          Scan# : 48
BP : m/z 326.0333      Int.  : 51.85
Output m/z range : 325.6868 to 326.3103      Cut Level : 69.10 %
```

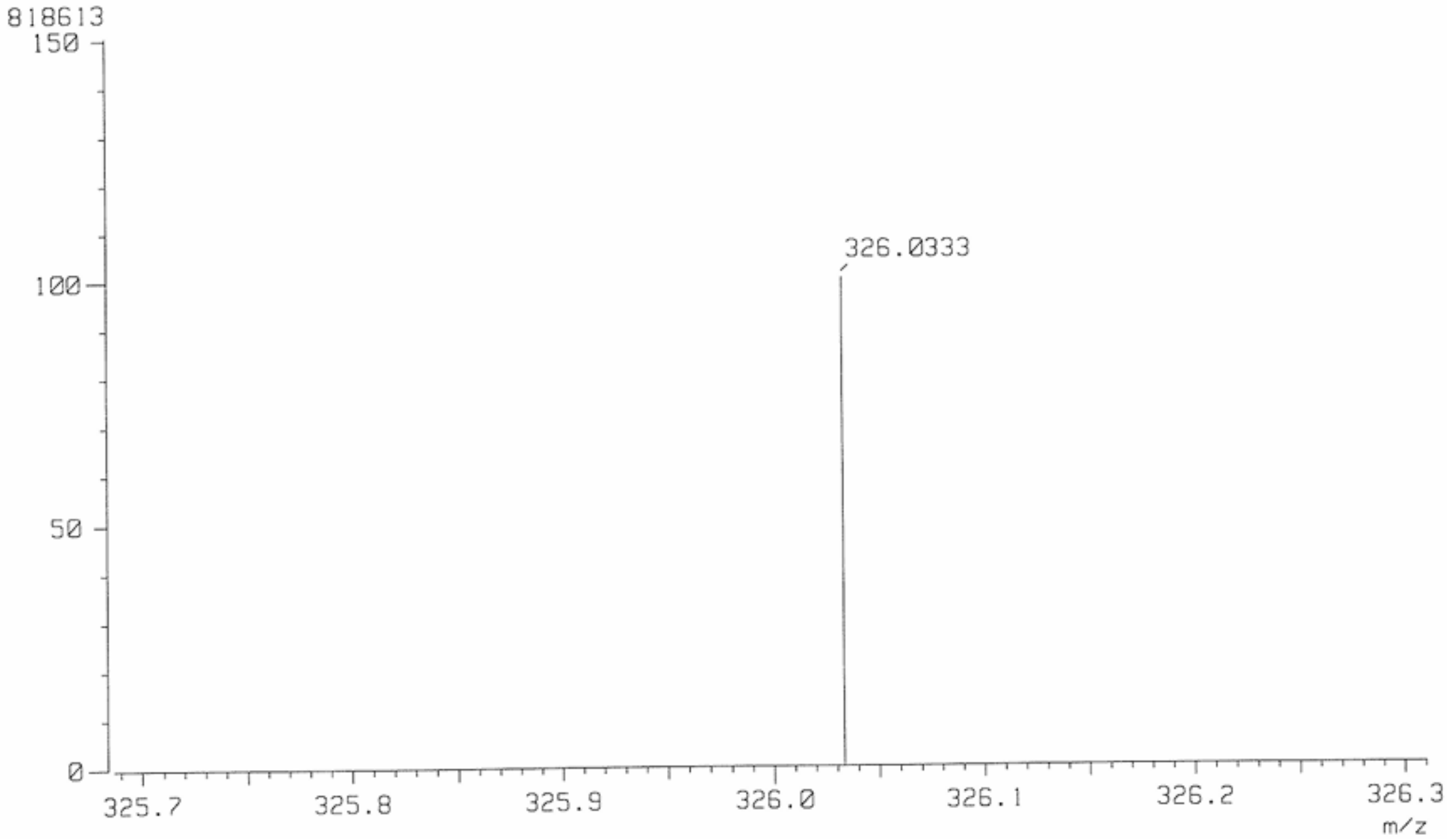

Compound 3h

```
[ Elemental Composition ]                                              Page: 1
Data   : EI-29-Code-LR-92-F3-023      Date : 04-Jul-2026 10:05
Sample: High resolution GC Mass (JMS-700)
Note : SMLab-Mass Analysis & Research Center
Inlet : Direct                         Ion Mode : EI+
RT : 1.27 min                          Scan#: (38,40)
Elements : C 15/0, H 14/0, Br 2/0, F 1/0, N 2/0, O 3/0, S 1/0
Mass Tolerance       : 1000ppm, 3mmu if m/z < 3, 5mmu if m/z > 5
Unsaturation (U.S.) : -0.5 - 80.0

Observed m/z Int%   Err[ppm / mmu]    U.S.  Composition
  445.8737    49.0     +0.4 /  +0.2   11.0  C 14 H 9 Br 2 F N 2 O 2 S
```

```
[ Mass Spectrum ]
Data   : EI-29-Code-LR-92-F3-023      Date : 04-Jul-2026 10:05
Sample: High resolution GC Mass (JMS-700)
Note : SMLab-Mass Analysis & Research Center
Inlet : Direct                         Ion Mode : EI+
Spectrum Type : Normal Ion [MF-Linear]
RT : 1.27 min           Scan# : (38,40)
BP : m/z 447.8712       Int.   : 52.94
Output m/z range : 445.4921 to 446.1763      Cut Level : 28.68 %
```

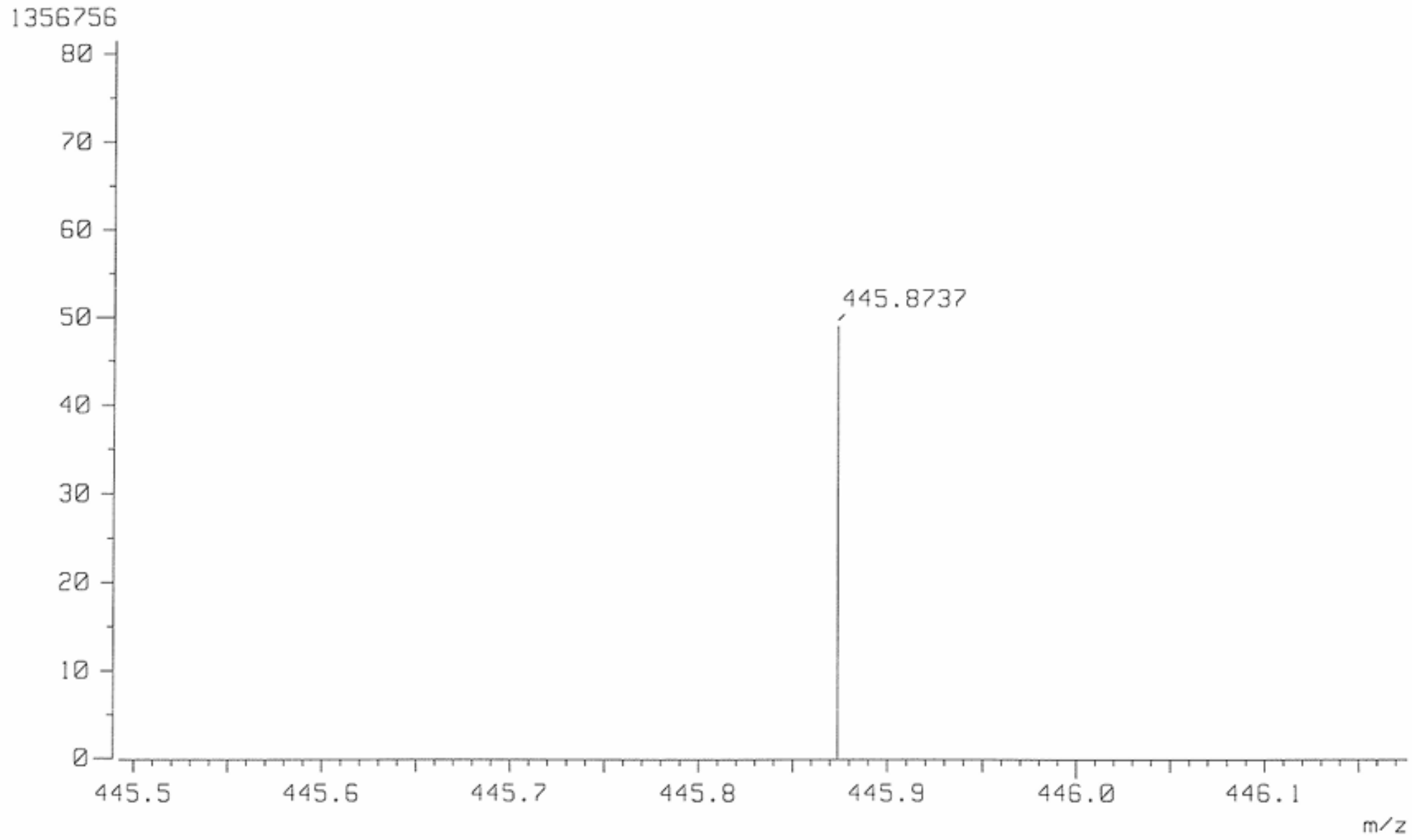

Compound 3i

```
[ Elemental Composition ]                                          Page: 1
Data   : EI-18-HS-265-014               Date : 03-Jul-2026 16:53
Sample: High resolution GC Mass (JMS-700)
Note : SMLab-Mass Analysis & Research Center
Inlet : Direct                          Ion Mode : EI+
RT : 0.25 min                           Scan#: (8,9)+12+16+20+23
Elements : C 22/0, H 20/0, F 1/0, N 2/0, O 7/0, S 1/0
Mass Tolerance       : 1000ppm, 3mmu if m/z < 3, 5mmu if m/z > 5
Unsaturation (U.S.) : -0.5 - 80.0

Observed m/z Int%   Err[ppm / mmu]    U.S.  Composition
  434.0950   100.0     +0.4 /  +0.2   13.0  C 20 H 19 F N 2 O 6 S
```

```
[ Mass Spectrum ]
Data   : EI-18-HS-265-014            Date : 03-Jul-2026 16:53
Sample: High resolution GC Mass (JMS-700)
Note : SMLab-Mass Analysis & Research Center
Inlet : Direct                       Ion Mode : EI+
Spectrum Type : Normal Ion [MF-Linear]
RT : 0.25 min          Scan# : (8,9)+12+16+20+23
BP : m/z 434.0950      Int.  : 34.76
Output m/z range : 433.8213 to 434.4085      Cut Level : 75.74 %
```

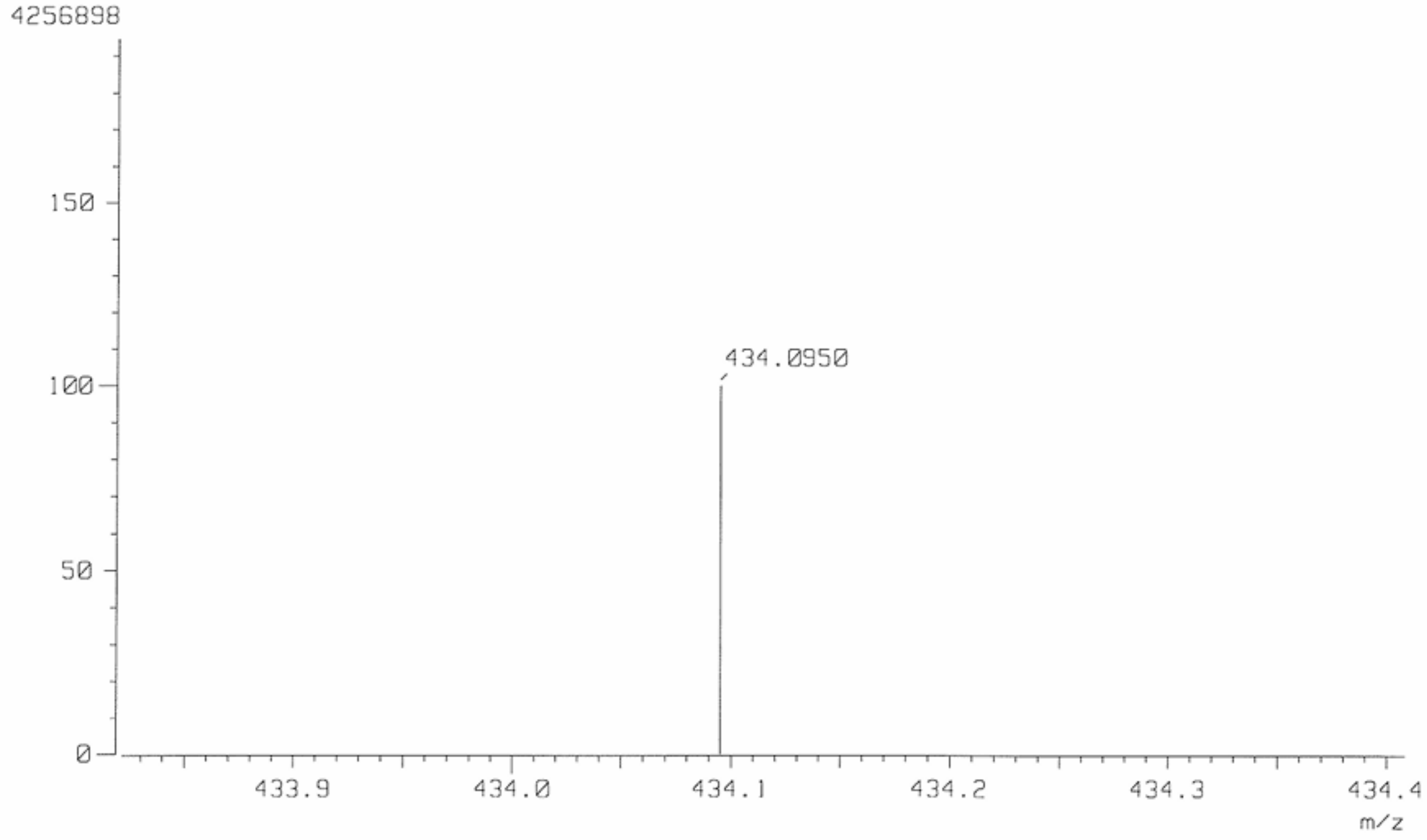

Compound 3j

```
[ Elemental Composition ]                                                    Page: 1
Data   : EI-17-HS-264-009                 Date : 03-Jul-2026 16:43
Sample: High resolution GC Mass (JMS-700)
Note : SMLab-Mass Analysis & Research Center
Inlet : Direct                            Ion Mode : EI+
RT : 0.80 min                             Scan#: 25
Elements : C 15/0, H 12/0, F 1/0, N 2/0, O 3/0, S 1/0
Mass Tolerance       : 1000ppm, 3mmu if m/z < 3, 5mmu if m/z > 5
Unsaturation (U.S.) : -0.5 - 80.0

Observed m/z Int%   Err[ppm / mmu]    U.S.  Composition
  290.0525    72.6     -0.1 /  +0.0   11.0  C 14 H 11 F N 2 O 2 S
```

```
[ Mass Spectrum ]
Data   : EI-17-HS-264-009             Date : 03-Jul-2026 16:43
Sample: High resolution GC Mass (JMS-700)
Note : SMLab-Mass Analysis & Research Center
Inlet : Direct                        Ion Mode : EI+
Spectrum Type : Normal Ion [MF-Linear]
RT : 0.80 min           Scan# : 25
BP : m/z 280.9864       Int.   : 88.13
Output m/z range : 289.7019 to 290.3839       Cut Level : 57.35 %
```

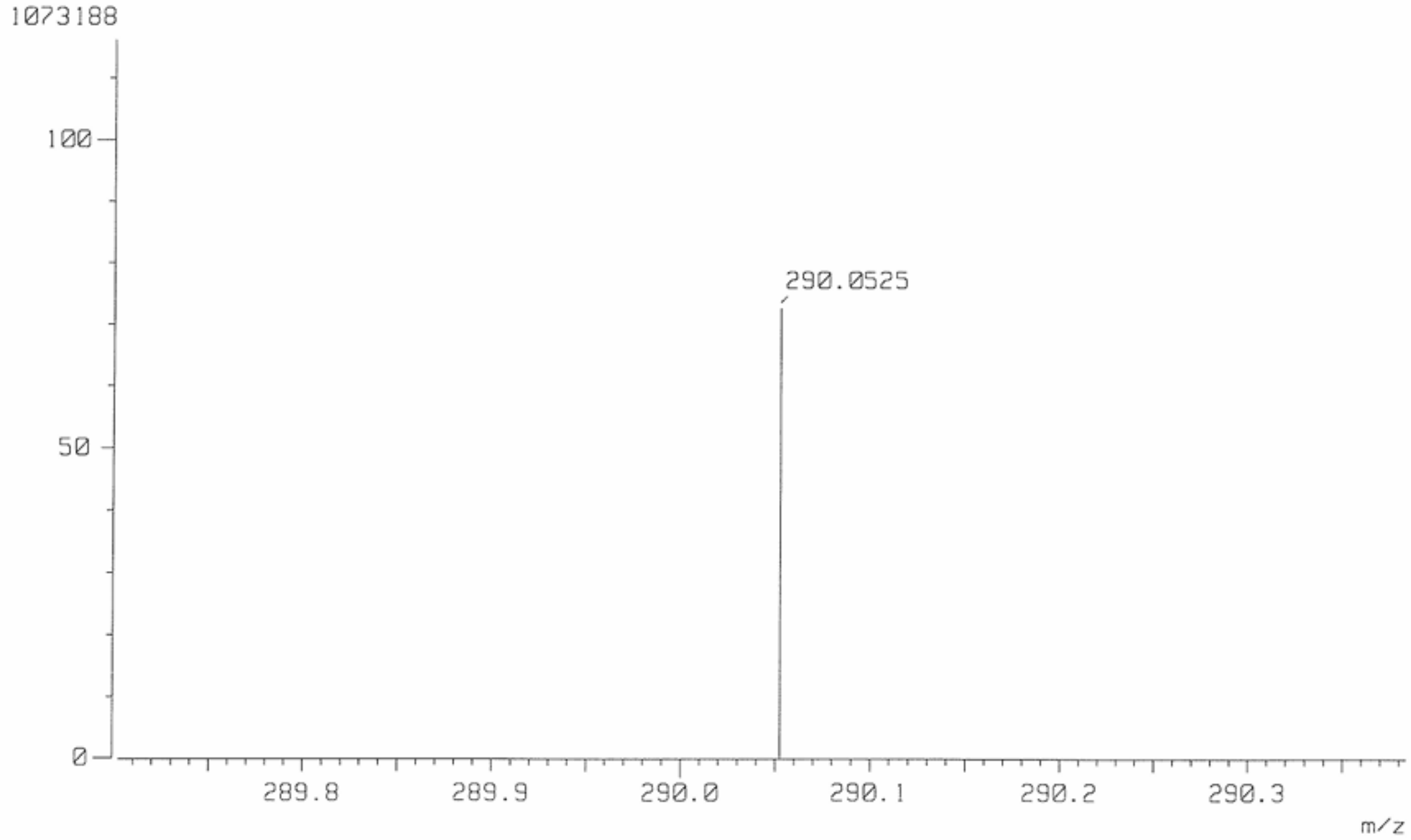

Compound 3k

```
[ Elemental Composition ]                                                    Page: 1
Data   : EI-2-HS-215-004                  Date : 03-Jul-2026 15:26
Sample: High resolution GC Mass (JMS-700)
Note : SMLab-Mass Analysis & Research Center
Inlet : Direct                            Ion Mode : EI+
RT : 0.90 min                             Scan#: (23,33)
Elements : C 17/0, H 16/0, F 1/0, N 2/0, O 3/0, S 2/0
Mass Tolerance       : 1000ppm, 3mmu if m/z < 3, 5mmu if m/z > 5
Unsaturation (U.S.) : -0.5 - 80.0

Observed m/z Int%   Err[ppm / mmu]    U.S.  Composition
  318.0834   100.0     -1.4 /  -0.5   11.0  C 16 H 15 F N 2 O 2 S
```

```
[ Mass Spectrum ]
Data   : EI-2-HS-215-004                Date : 03-Jul-2026 15:26
Sample: High resolution GC Mass (JMS-700)
Note : SMLab-Mass Analysis & Research Center
Inlet : Direct                          Ion Mode : EI+
Spectrum Type : Normal Ion [MF-Linear]
RT : 0.90 min          Scan# : (23,33)
BP : m/z 318.0834      Int.   : 36.23
Output m/z range : 317.7204 to 318.4415      Cut Level : 75.94 %
```

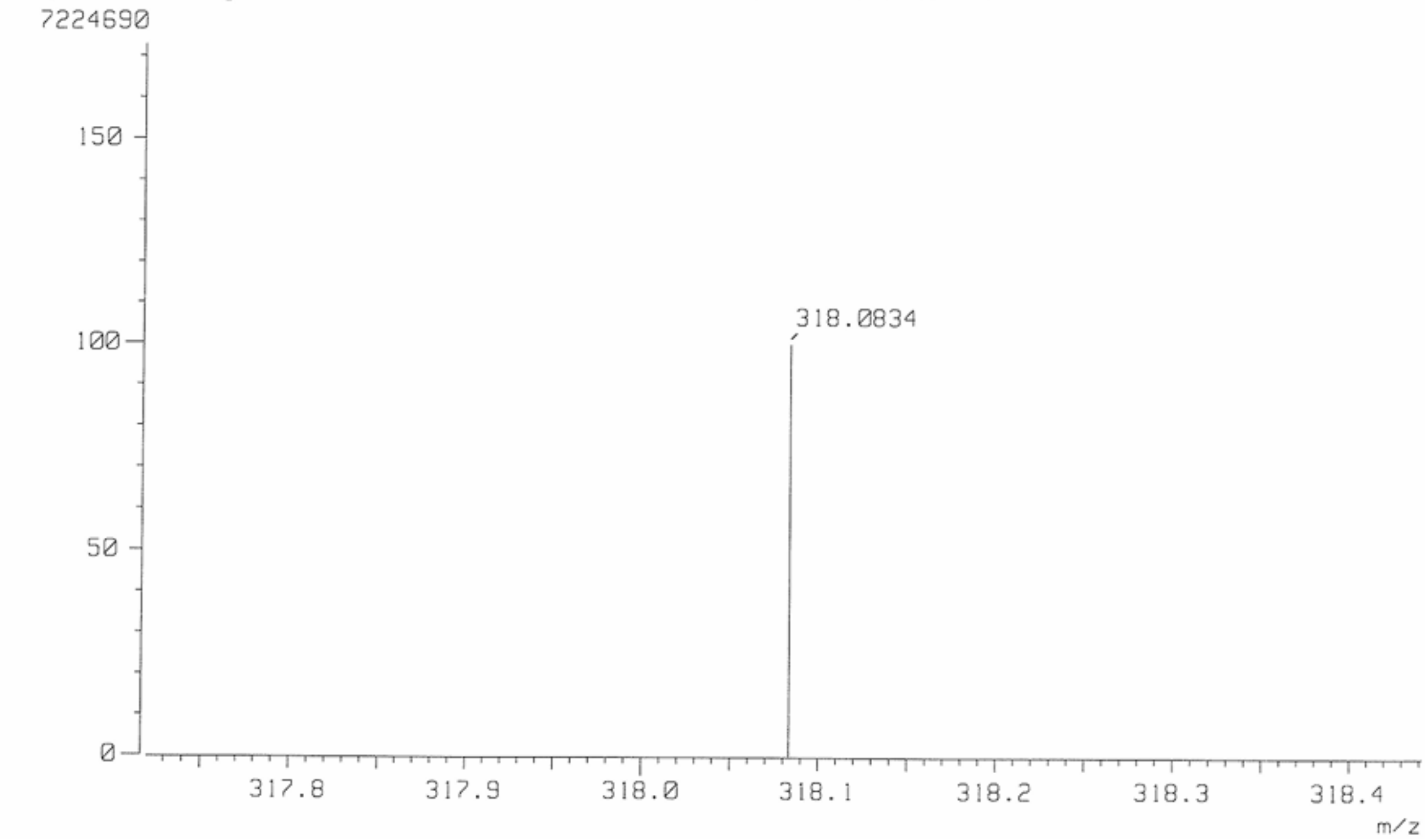

Compound 3l

```
[ Elemental Composition ]                                              Page: 1
Data   : EI-6-HS-235-011                 Date : 03-Jul-2026 15:38
Sample: High resolution GC Mass (JMS-700)
Note : SMLab-Mass Analysis & Research Center
Inlet : Direct                           Ion Mode : EI+
RT : 0.77 min                            Scan#: 24
Elements : C 18/0, H 10/0, F 7/0, N 2/0, O 2/0, S 1/0
Mass Tolerance       : 1000ppm, 3mmu if m/z < 3, 5mmu if m/z > 5
Unsaturation (U.S.) : -0.5 - 80.0

Observed m/z Int%   Err[ppm / mmu]   U.S.  Composition
  426.0270   100.0     -0.6 /  -0.3   11.0  C 16 H 9 F 7 N 2 O 2 S
```

```
[ Mass Spectrum ]
Data   : EI-6-HS-235-011             Date : 03-Jul-2026 15:38
Sample: High resolution GC Mass (JMS-700)
Note : SMLab-Mass Analysis & Research Center
Inlet : Direct                       Ion Mode : EI+
Spectrum Type : Normal Ion [MF-Linear]
RT : 0.77 min           Scan# : 24
BP : m/z 426.0270       Int.   : 58.65
Output m/z range : 425.6586 to 426.3903      Cut Level : 63.97 %
```

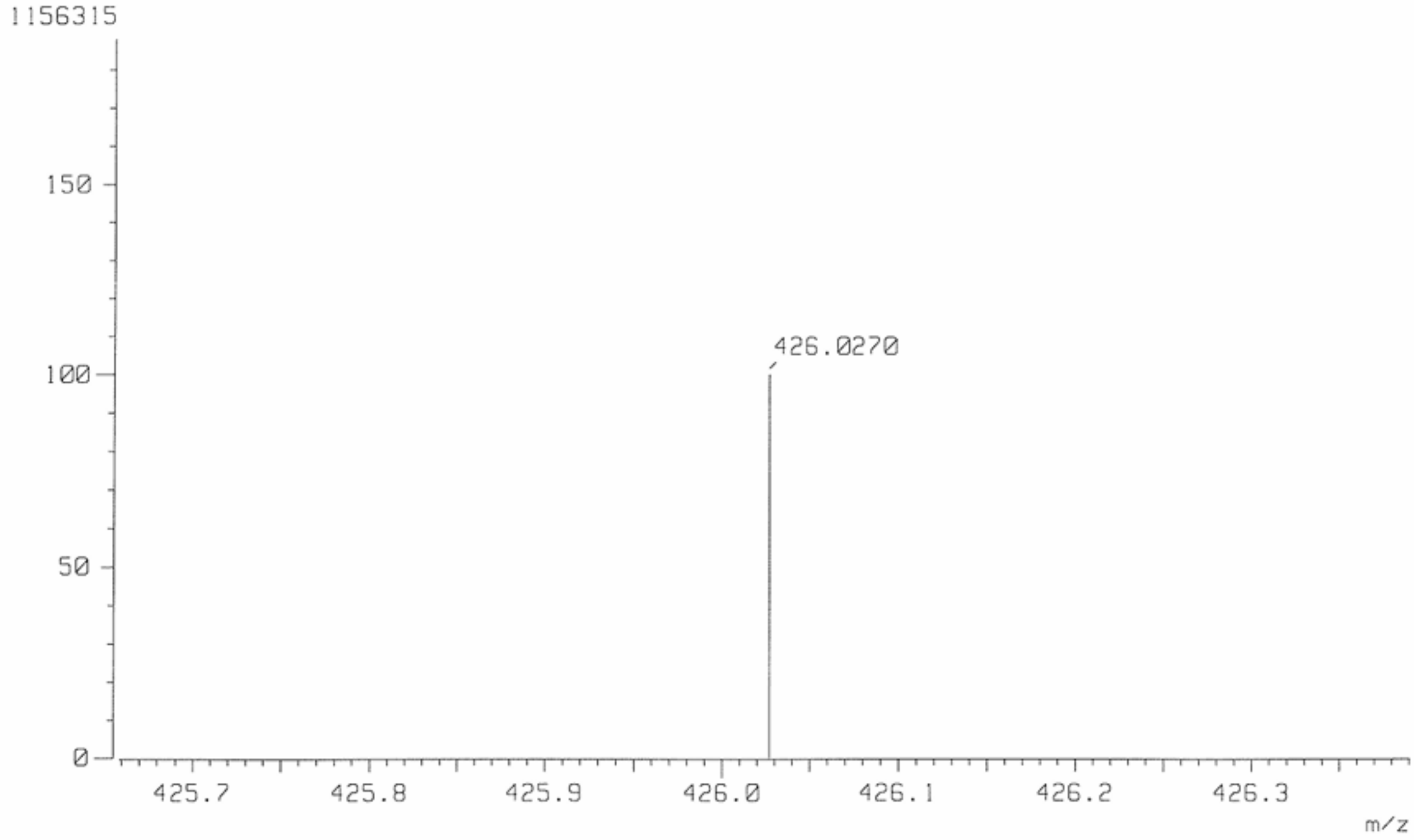

Compound 3m

```
[ Elemental Composition ]                                                    Page: 1
Data   : EI-30-Code-LR-95-F2-004      Date : 04-Jul-2026 09:33
Sample: High resolution GC Mass (JMS-700)
Note : SMLab-Mass Analysis & Research Center
Inlet : Direct                         Ion Mode : EI+
RT : 0.45 min                          Scan#: (14,15)
Elements : C 14/0, H 14/0, Cl 2/0, F 1/0, N 2/0, O 3/0, S 1/0
Mass Tolerance       : 1000ppm, 3mmu if m/z < 3, 5mmu if m/z > 5
Unsaturation (U.S.) : -0.5 - 80.0

Observed m/z Int%   Err[ppm / mmu]    U.S.  Composition
  357.9748    74.0     +0.6 /  +0.2   11.0  C 14 H 9 Cl 2 F N 2 O 2 S
```

```
[ Mass Spectrum ]
Data  : EI-30-Code-LR-95-F2-004      Date : 04-Jul-2026 09:33
Sample: High resolution GC Mass (JMS-700)
Note : SMLab-Mass Analysis & Research Center
Inlet : Direct                       Ion Mode : EI+
Spectrum Type : Normal Ion [MF-Linear]
RT : 0.45 min          Scan# : (14,15)
BP : m/z 330.9792      Int.  : 56.55
Output m/z range : 357.5989 to 358.3101      Cut Level : 59.56 %
```

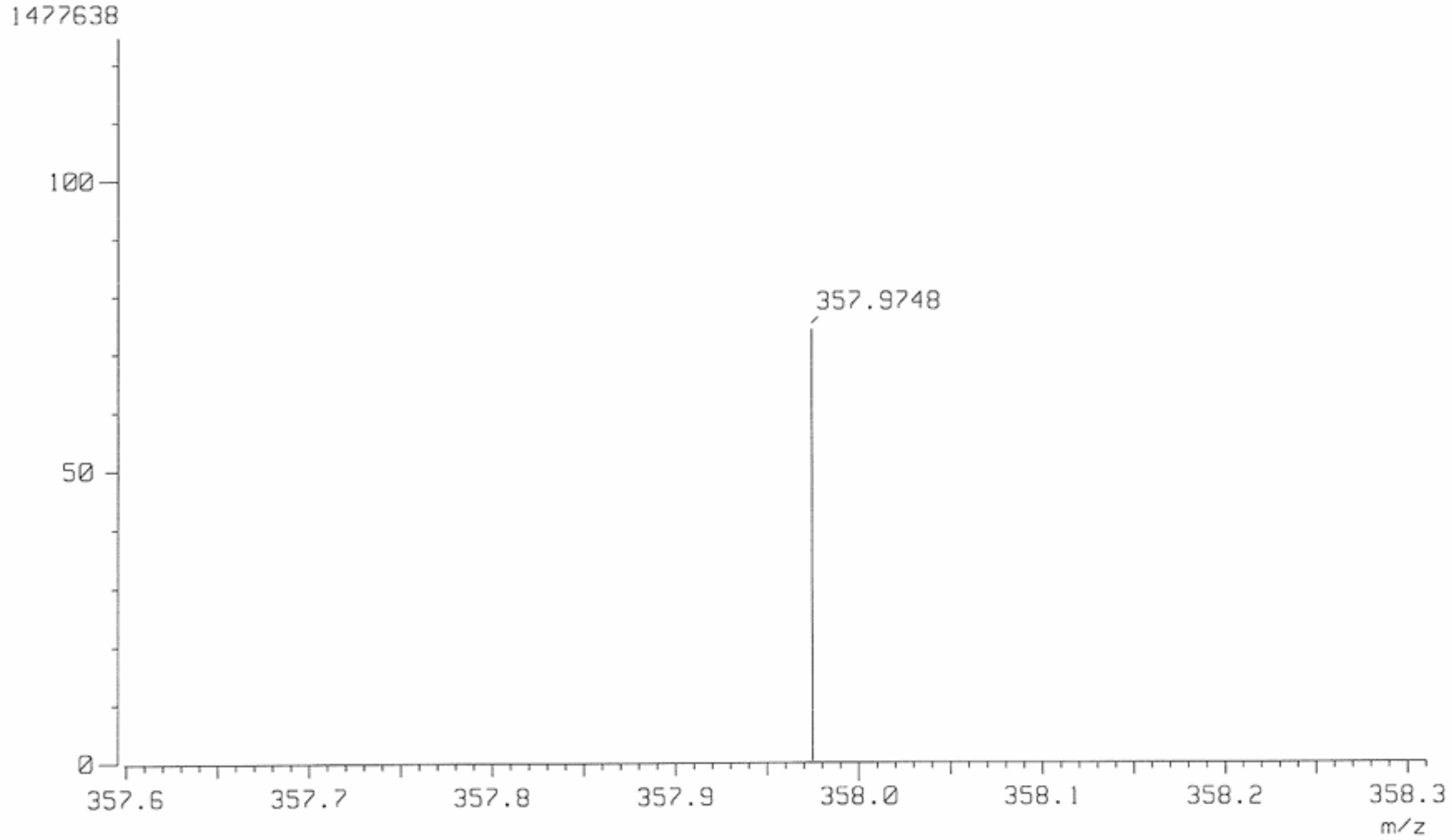

Compound 3n

[Elemental Composition] Page: 1
Data : EI-31-Code-LR-96-F2-024 Date : 04-Jul-2026 10:08
Sample: High resolution GC Mass (JMS-700)
Note : SMLab-Mass Analysis & Research Center
Inlet : Direct Ion Mode : EI+
RT : 1.20 min Scan#: (31,43)
Elements : C 15/0, H 14/0, Br 2/0, F 1/0, N 2/0, O 3/0, S 1/0
Mass Tolerance : 1000ppm, 3mmu if m/z < 3, 5mmu if m/z > 5
Unsaturation (U.S.) : -0.5 - 80.0

Observed m/z	Int%	Err[ppm / mmu]	U.S.	Composition
445.8737	49.9	+0.4 / +0.2	11.0	C 14 H 9 Br 2 F N 2 O 2 S

[Mass Spectrum]
Data : EI-31-Code-LR-96-F2-024 Date : 04-Jul-2026 10:08
Sample: High resolution GC Mass (JMS-700)
Note : SMLab-Mass Analysis & Research Center
Inlet : Direct Ion Mode : EI+
Spectrum Type : Normal Ion [MF-Linear]
RT : 1.20 min Scan# : (31,43)
BP : m/z 447.8712 Int. : 61.12
Output m/z range : 445.5535 to 446.1893 Cut Level : 34.56 %

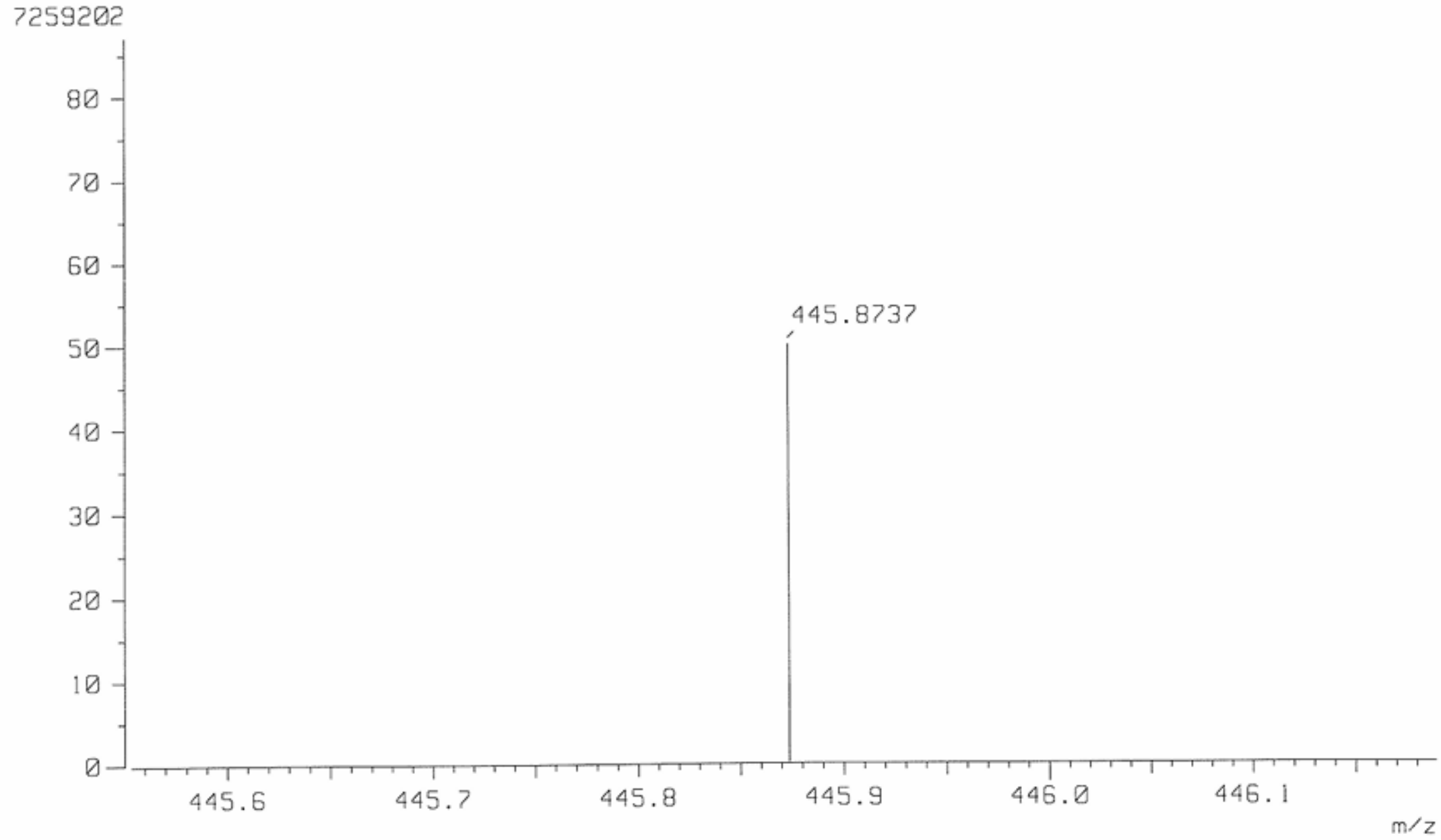

Compound 3o

```
[ Elemental Composition ]                                                    Page: 1
Data   : EI-19-HS-266-018               Date : 03-Jul-2026 17:00
Sample: High resolution GC Mass (JMS-700)
Note : SMLab-Mass Analysis & Research Center
Inlet : Direct                          Ion Mode : EI+
RT : 0.44 min                           Scan#: 14+16
Elements : C 20/0, H 18/0, F 1/0, N 2/0, O 5/0, S 1/0
Mass Tolerance       : 1000ppm, 3mmu if m/z < 3, 5mmu if m/z > 5
Unsaturation (U.S.) : -0.5 - 80.0

Observed m/z Int%   Err[ppm / mmu]    U.S.  Composition
  374.0737   100.0     +0.2 /  +0.1   13.0  C 18 H 15 F N 2 O 4 S
```

```
[ Mass Spectrum ]
Data   : EI-19-HS-266-018            Date : 03-Jul-2026 17:00
Sample: High resolution GC Mass (JMS-700)
Note : SMLab-Mass Analysis & Research Center
Inlet : Direct                       Ion Mode : EI+
Spectrum Type : Normal Ion [MF-Linear]
RT : 0.44 min           Scan# : 14+16
BP : m/z 374.0737       Int.   : 59.79
Output m/z range : 373.6907 to 374.3827      Cut Level : 74.26 %
```

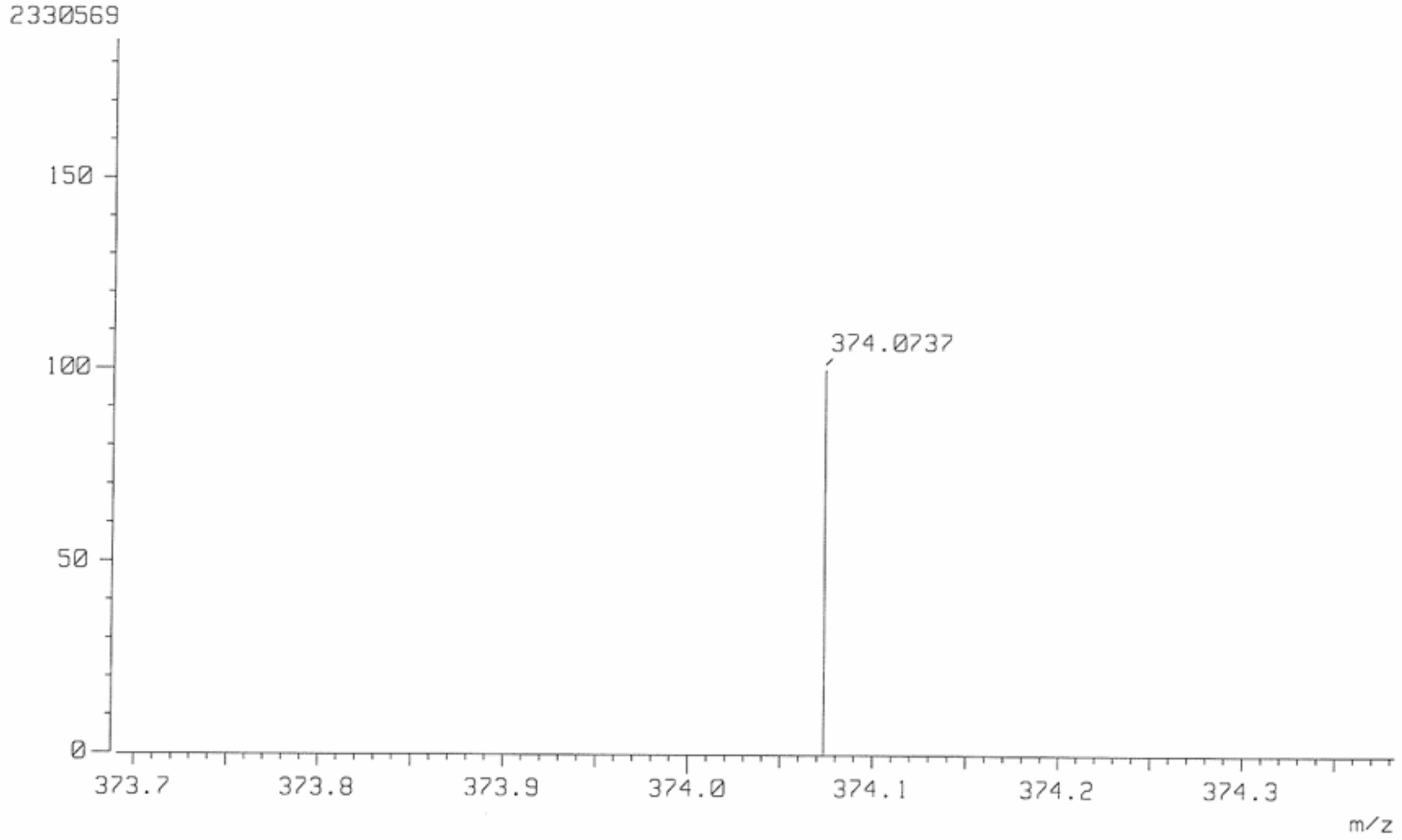

Compound 3p

[Elemental Composition]   Page: 1
Data : EI-9-HS-238-013   Date : 03-Jul-2026 15:42
Sample: High resolution GC Mass (JMS-700)
Note : SMLab-Mass Analysis & Research Center
Inlet : Direct   Ion Mode : EI+
RT : 0.94 min   Scan#: 29+25+23+22
Elements : C 21/0, H 20/0, F 1/0, N 2/0, O 7/0, S 1/0
Mass Tolerance : 1000ppm, 3mmu if m/z < 3, 5mmu if m/z > 5
Unsaturation (U.S.) : -0.5 - 80.0

Observed m/z	Int%	Err[ppm / mmu]	U.S.	Composition
434.0944	33.9	-1.0 / -0.4	13.0	C 20 H 19 F N 2 O 6 S

[Mass Spectrum]
Data : EI-9-HS-238-013   Date : 03-Jul-2026 15:42
Sample: High resolution GC Mass (JMS-700)
Note : SMLab-Mass Analysis & Research Center
Inlet : Direct   Ion Mode : EI+
Spectrum Type : Normal Ion [MF-Linear]
RT : 0.94 min   Scan# : 29+25+23+22
BP : m/z 374.1496   Int. : 90.05
Output m/z range : 433.7915 to 434.3655   Cut Level : 8.09 %

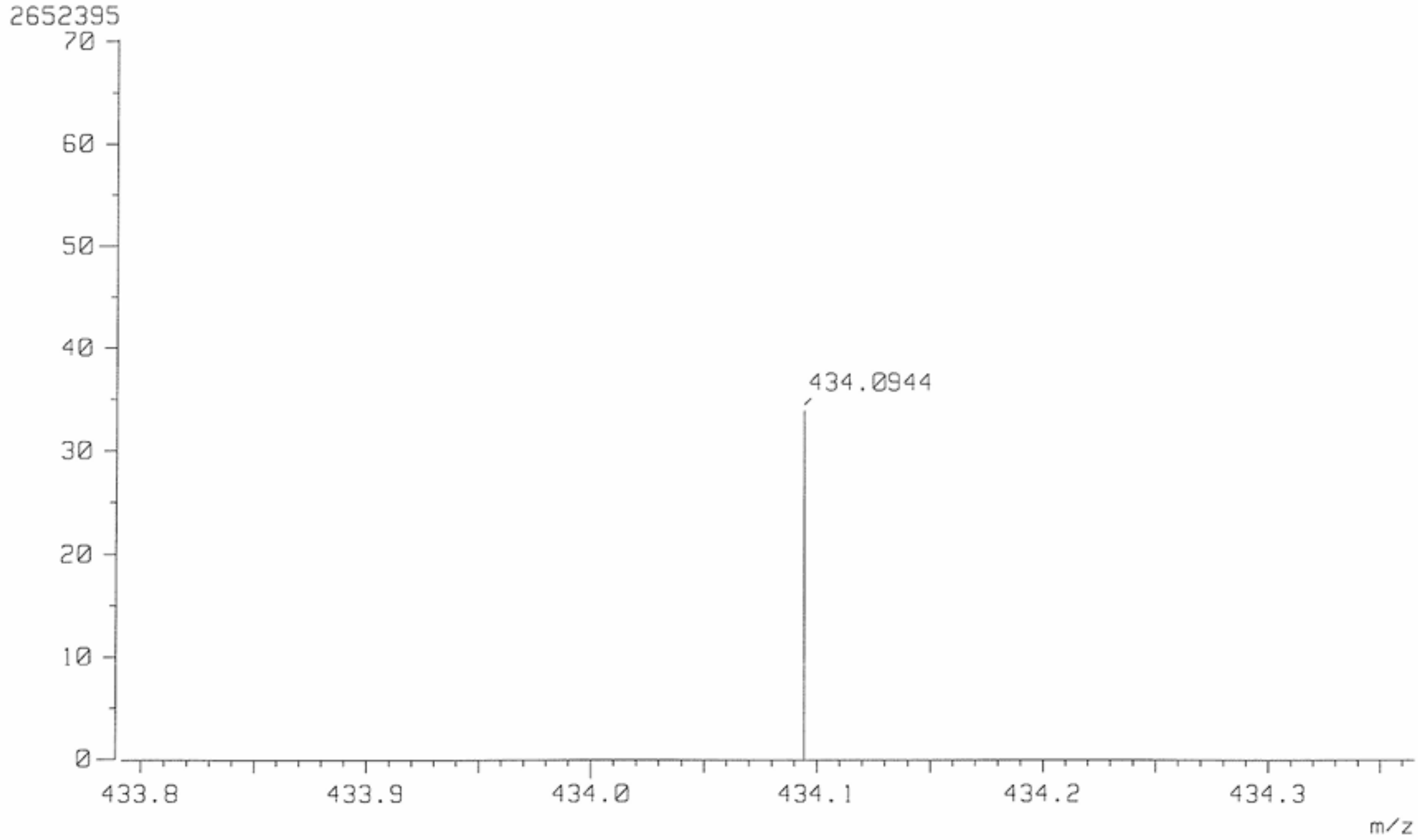

Compound 3aa

[Elemental Composition]

Data : EI-1-HS-4a-007 Date : 16-Sep-2026 15:19 Page: 1

Sample: High resolution GC Mass (JMS-700)

Note : SMLab-Mass Analysis & Research Center

Inlet : Direct Ion Mode : EI+

RT : 0.20 min Scan#: 7

Elements : C 17/0, H 20/0, F 1/0, N 2/0, O 2/0, S 1/0

Mass Tolerance : 1000ppm, 3mmu if m/z < 3, 5mmu if m/z > 5

Unsaturation (U.S.) : -0.5 - 80.0

Observed m/z	Int%	Err[ppm / mmu]	U.S.	Composition
320.0990	100.0	-1.5 / -0.5	10.0	C 16 H 17 F N 2 O 2 S

[Mass Spectrum]

Data : EI-1-HS-4a-007 Date : 16-Sep-2026 15:19

Sample: High resolution GC Mass (JMS-700)

Note : SMLab-Mass Analysis & Research Center

Inlet : Direct Ion Mode : EI+

Spectrum Type : Normal Ion [MF-Linear]

RT : 0.20 min Scan# : 7

BP : m/z 320.0990 Int. : 399.92

Output m/z range : 319.8122 to 320.3468 Cut Level : 80.88 %

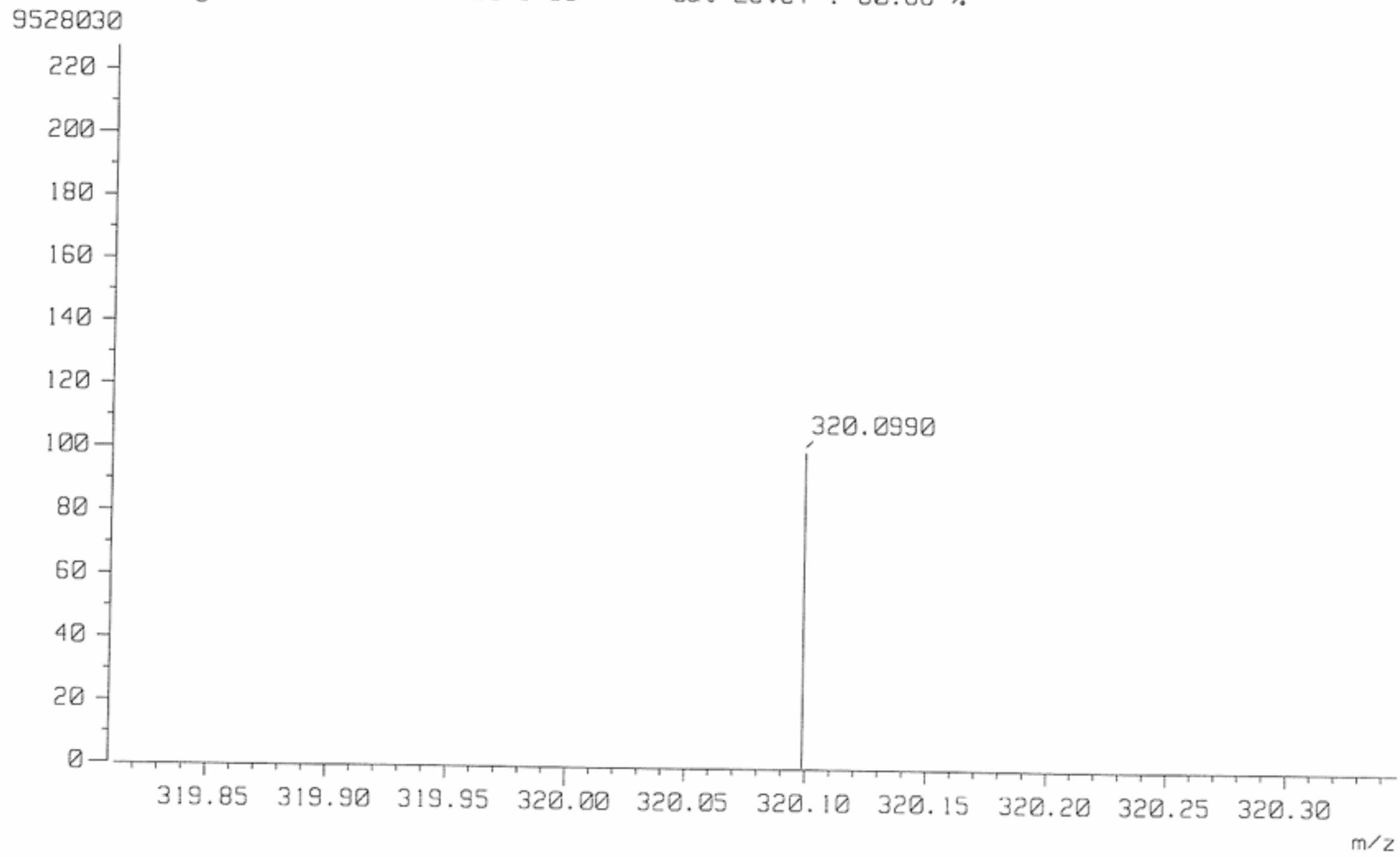

Compound 5a

[Elemental Composition]　　Page: 1
Data : EI-13-HS-259-002　　Date : 03-Jul-2026 16:11
Sample: High resolution GC Mass (JMS-700)
Note : SMLab-Mass Analysis & Research Center
Inlet : Direct　　Ion Mode : EI+
RT : 0.94 min　　Scan#: 29
Elements : C 20/0, H 20/0, F 1/0, N 2/0, O 3/0, S 1/0
Mass Tolerance : 1000ppm, 3mmu if m/z < 3, 5mmu if m/z > 5
Unsaturation (U.S.) : -0.5 - 80.0

Observed m/z	Int%	Err[ppm / mmu]	U.S.	Composition
346.1147	99.0	-1.1 / -0.4	11.0	C 18 H 19 F N 2 O 2 S

[Mass Spectrum]
Data : EI-13-HS-259-002　　Date : 03-Jul-2026 16:11
Sample: High resolution GC Mass (JMS-700)
Note : SMLab-Mass Analysis & Research Center
Inlet : Direct　　Ion Mode : EI+
Spectrum Type : Normal Ion [MF-Linear]
RT : 0.94 min　　Scan# : 29
BP : m/z 330.9792　　Int. : 49.14
Output m/z range : 345.7822 to 346.4178　　Cut Level : 72.79 %

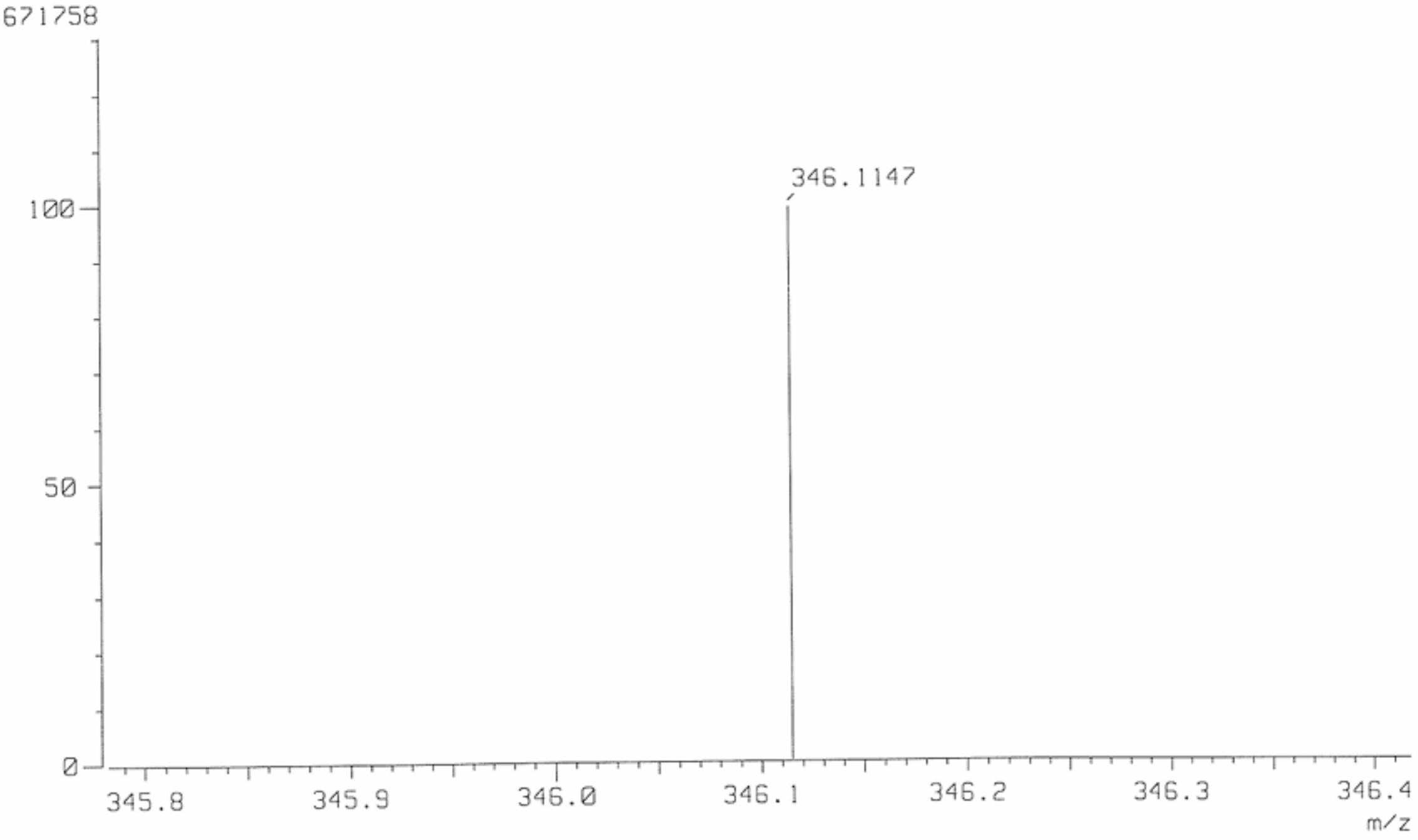

Compound 5b

```
[ Elemental Composition ]                                                    Page: 1
Data   : EI-33-Code-LR-100-F2-010      Date : 04-Jul-2026 09:44
Sample: High resolution GC Mass (JMS-700)
Note : SMLab-Mass Analysis & Research Center
Inlet : Direct                         Ion Mode : EI+
RT : 0.55 min                          Scan#: (16,19)+21+22
Elements : C 17/0, H 14/0, F 3/0, N 2/0, O 3/0, S 1/0
Mass Tolerance      : 1000ppm, 3mmu if m/z < 3, 5mmu if m/z > 5
Unsaturation (U.S.) : -0.5 - 80.0

Observed m/z Int%   Err[ppm / mmu]    U.S.  Composition
  354.0651   100.0     +0.2 /  +0.1   11.0  C 16 H 13 F 3 N 2 O 2 S
```

```
[ Mass Spectrum ]
Data   : EI-33-Code-LR-100-F2-010      Date : 04-Jul-2026 09:44
Sample: High resolution GC Mass (JMS-700)
Note : SMLab-Mass Analysis & Research Center
Inlet : Direct                         Ion Mode : EI+
Spectrum Type : Normal Ion [MF-Linear]
RT : 0.55 min            Scan# : (16,19)+21+22
BP : m/z 354.0651        Int.   : 28.62
Output m/z range : 353.7304 to 354.3839       Cut Level : 77.21 %
```

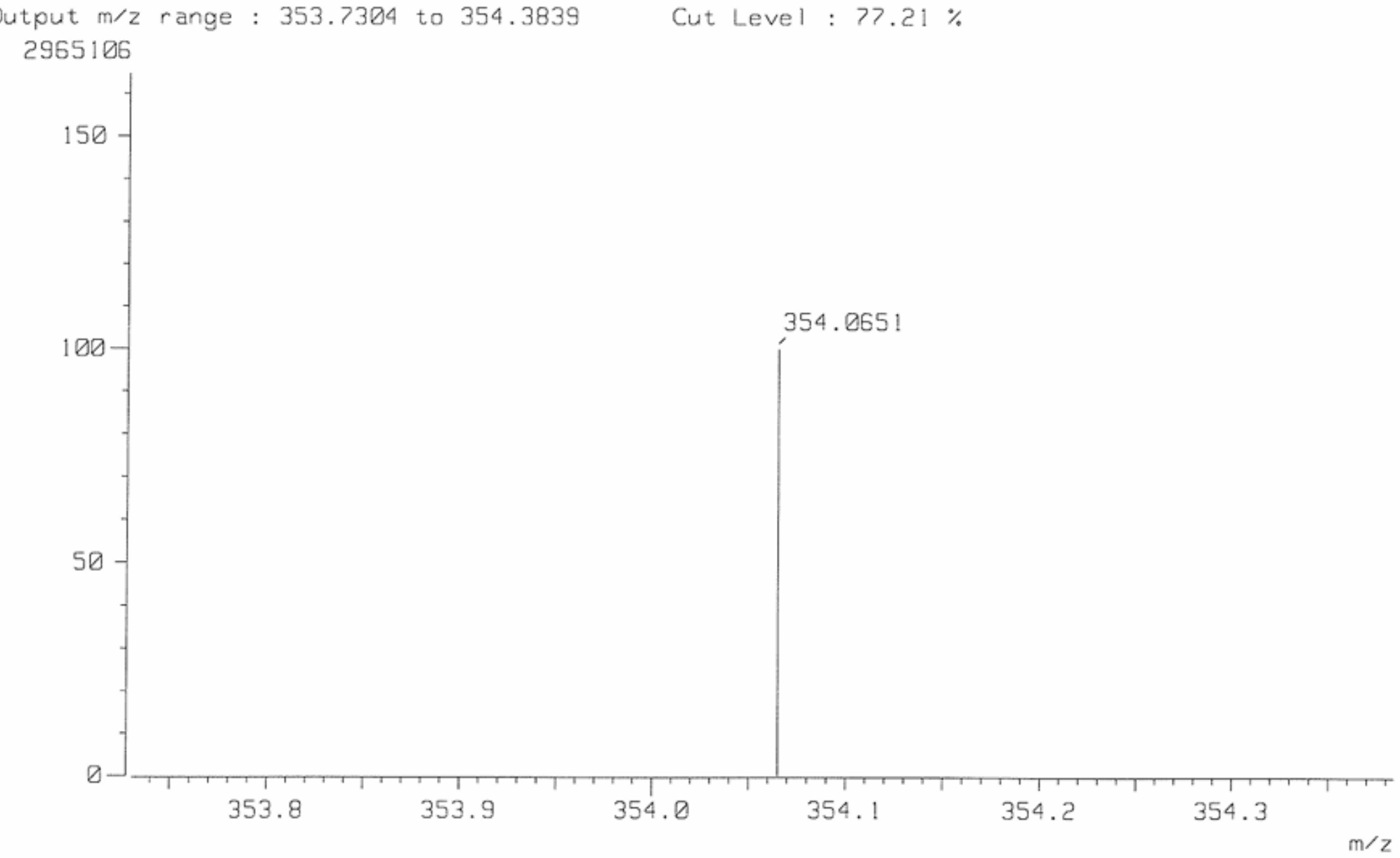

Compound 5c

[Elemental Composition] Page: 1
Data : EI-20-HS-267-019 Date : 03-Jul-2026 17:03
Sample: High resolution GC Mass (JMS-700)
Note : SMLab-Mass Analysis & Research Center
Inlet : Direct Ion Mode : EI+
RT : 0.57 min Scan#: (17,19)+26
Elements : C 17/0, H 14/0, Cl 2/0, F 1/0, N 2/0, O 3/0, S 1/0
Mass Tolerance : 1000ppm, 3mmu if m/z < 3, 5mmu if m/z > 5
Unsaturation (U.S.) : -0.5 - 80.0

Observed m/z	Int%	Err[ppm / mmu]	U.S.	Composition
386.0059	100.0	-0.1 / +0.0	11.0	C 16 H 13 Cl 2 F N 2 O 2 S

[Mass Spectrum]
Data : EI-20-HS-267-019 Date : 03-Jul-2026 17:03
Sample: High resolution GC Mass (JMS-700)
Note : SMLab-Mass Analysis & Research Center
Inlet : Direct Ion Mode : EI+
Spectrum Type : Normal Ion [MF-Linear]
RT : 0.57 min Scan# : (17,19)+26
BP : m/z 386.0059 Int. : 37.78
Output m/z range : 385.7055 to 386.2889 Cut Level : 47.92 %

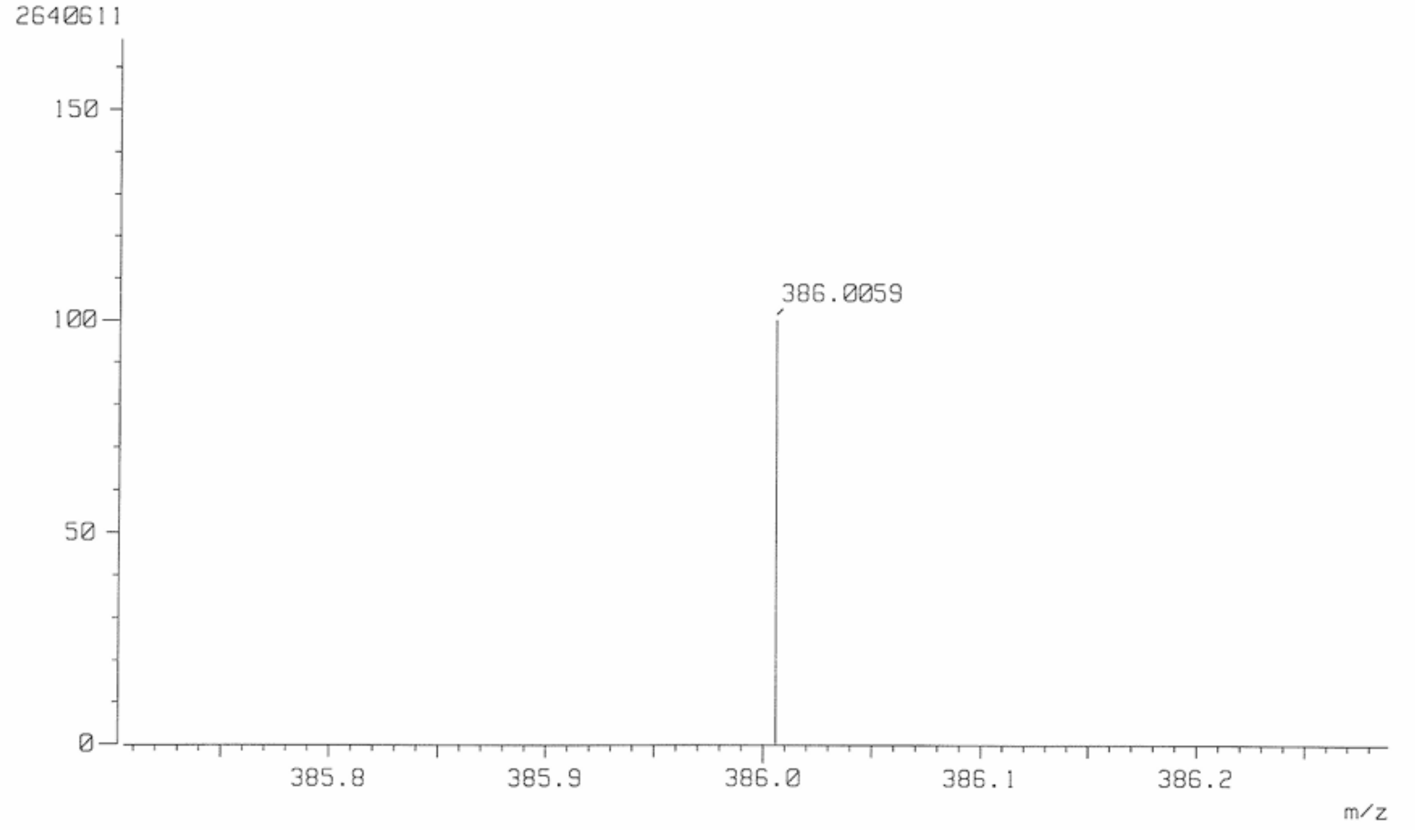

Compound 5d

```
[ Elemental Composition ]                                              Page: 1
Data   : EI-34-Code-LR-101-F3-008      Date : 04-Jul-2026 09:39
Sample: High resolution GC Mass (JMS-700)
Note : SMLab-Mass Analysis & Research Center
Inlet : Direct                         Ion Mode : EI+
RT : 0.44 min                          Scan#: (13,15)
Elements : C 17/0, H 14/0, F 3/0, N 2/0, O 3/0, S 1/0
Mass Tolerance       : 1000ppm, 3mmu if m/z < 3, 5mmu if m/z > 5
Unsaturation (U.S.) : -0.5 - 80.0

Observed m/z Int%   Err[ppm / mmu]    U.S.  Composition
  354.0648   100.0     -0.6 /  -0.2   11.0  C 16 H 13 F 3 N 2 O 2 S
```

```
[ Mass Spectrum ]
Data   : EI-34-Code-LR-101-F3-008      Date : 04-Jul-2026 09:39
Sample: High resolution GC Mass (JMS-700)
Note : SMLab-Mass Analysis & Research Center
Inlet : Direct                         Ion Mode : EI+
Spectrum Type : Normal Ion [MF-Linear]
RT : 0.44 min           Scan# : (13,15)
BP : m/z 354.0648       Int.  : 67.41
Output m/z range : 353.7226 to 354.3914       Cut Level : 78.68 %
```

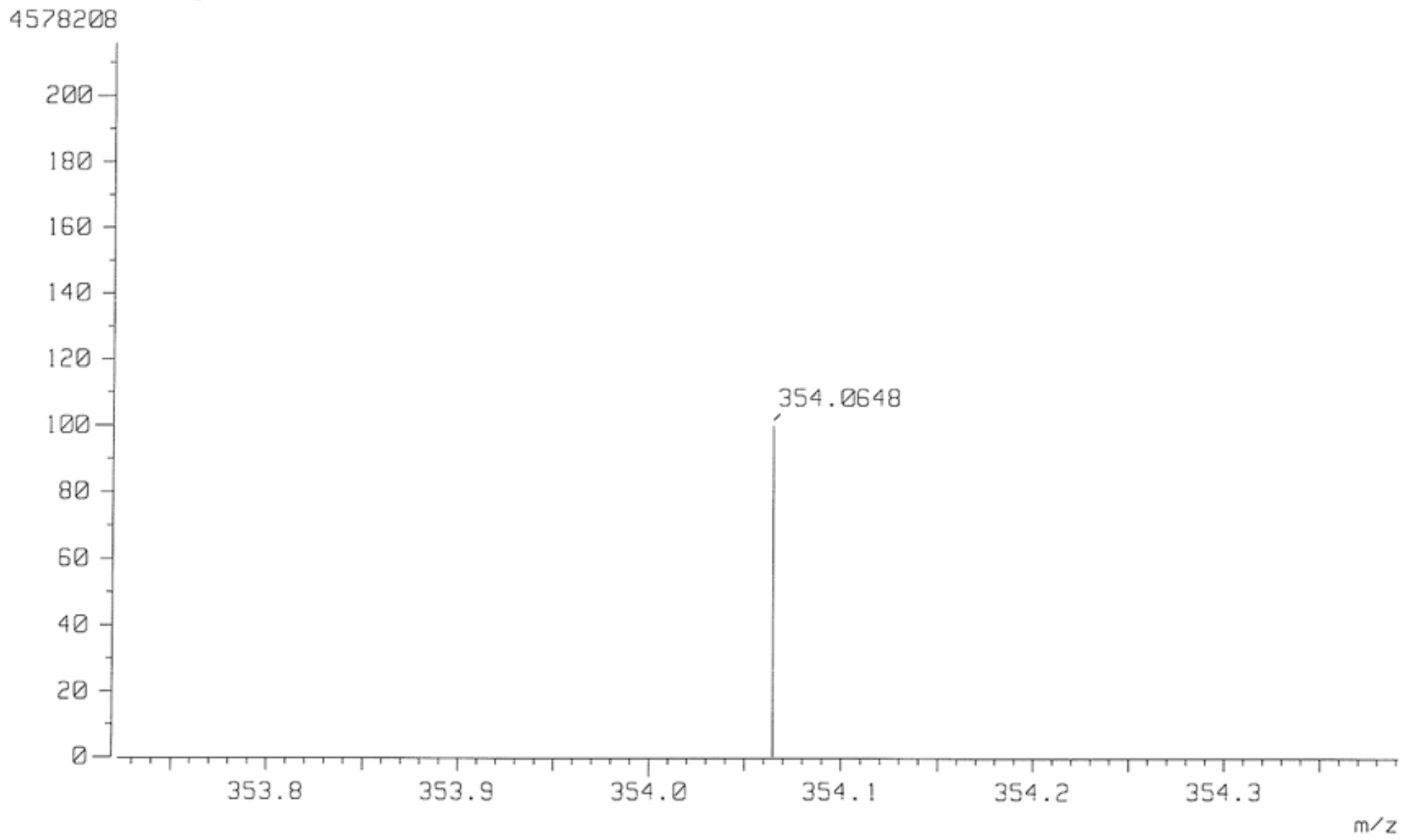

Compound 5e

[Elemental Composition] Page: 1
Data : EI-32-Code-LR-98-F3-002 Date : 04-Jul-2026 09:29
Sample: High resolution GC Mass (JMS-700)
Note : SMLab-Mass Analysis & Research Center
Inlet : Direct Ion Mode : EI+
RT : 0.62 min Scan#: (19,20)
Elements : C 20/0, H 20/0, F 1/0, N 2/0, O 3/0, S 1/0
Mass Tolerance : 1000ppm, 3mmu if m/z < 3, 5mmu if m/z > 5
Unsaturation (U.S.) : -0.5 - 80.0

Observed m/z	Int%	Err[ppm / mmu]	U.S.	Composition
346.1150	100.0	-0.3 / -0.1	11.0	C 18 H 19 F N 2 O 2 S

[Mass Spectrum]
Data : EI-32-Code-LR-98-F3-002 Date : 04-Jul-2026 09:29
Sample: High resolution GC Mass (JMS-700)
Note : SMLab-Mass Analysis & Research Center
Inlet : Direct Ion Mode : EI+
Spectrum Type : Normal Ion [MF-Linear]
RT : 0.62 min Scan# : (19,20)
BP : m/z 346.1150 Int. : 77.80
Output m/z range : 345.8177 to 346.3625 Cut Level : 74.26 %

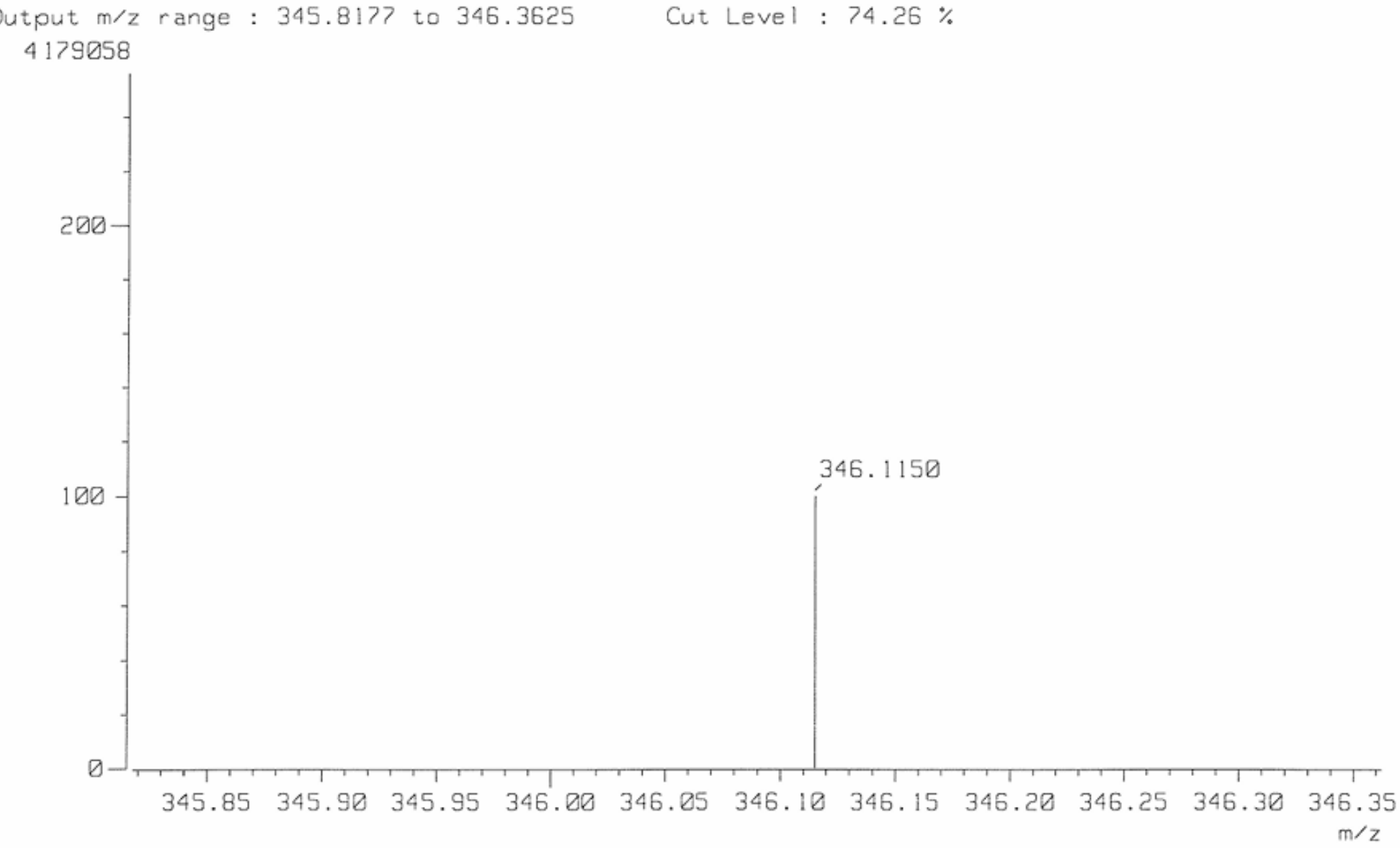

Compound 5f

```
[ Elemental Composition ]                                              Page: 1
Data   : EI-14-HS-260-021              Date : 03-Jul-2026 15:57
Sample: High resolution GC Mass (JMS-700)
Note : SMLab-Mass Analysis & Research Center
Inlet : Direct                         Ion Mode : EI+
RT : 0.74 min                          Scan#: 23
Elements : C 18/0, H 15/0, F 3/0, N 2/0, O 3/0, S 1/0
Mass Tolerance       : 1000ppm, 3mmu if m/z < 3, 5mmu if m/z > 5
Unsaturation (U.S.) : -0.5 - 80.0

Observed m/z  Int%    Err[ppm / mmu]    U.S.  Composition
  354.0646    95.0      -1.2 /  -0.4    11.0  C 16 H 13 F 3 N 2 O 2 S
```

```
[ Mass Spectrum ]
Data   : EI-14-HS-260-021              Date : 03-Jul-2026 15:57
Sample: High resolution GC Mass (JMS-700)
Note : SMLab-Mass Analysis & Research Center
Inlet : Direct                         Ion Mode : EI+
Spectrum Type : Normal Ion [MF-Linear]
RT : 0.74 min          Scan# : 23
BP : m/z 330.9750      Int.  : 65.81
Output m/z range : 353.7357 to 354.3895      Cut Level : 64.71 %
```

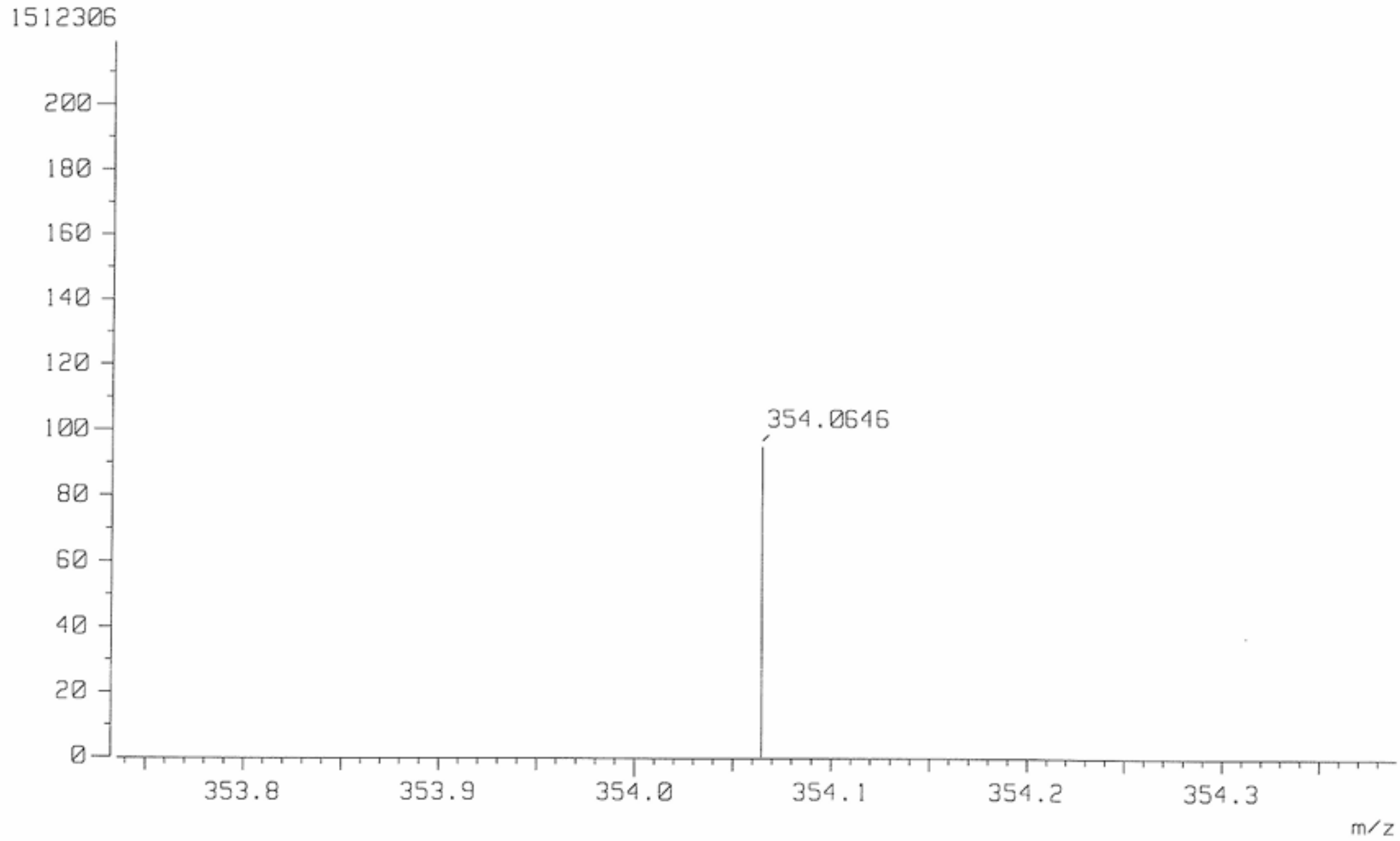

Compound 5g

[Elemental Composition] Page: 1
Data : EI-37-Code-LR-104-F2-016 Date : 04-Jul-2026 09:53
Sample: High resolution GC Mass (JMS-700)
Note : SMLab-Mass Analysis & Research Center
Inlet : Direct Ion Mode : EI+
RT : 1.17 min Scan#: (35,37)
Elements : C 17/0, H 14/0, Cl 2/0, F 1/0, N 2/0, O 3/0, S 1/0
Mass Tolerance : 1000ppm, 3mmu if m/z < 3, 5mmu if m/z > 5
Unsaturation (U.S.) : -0.5 - 80.0

Observed m/z	Int%	Err[ppm / mmu]	U.S.	Composition
386.0054	89.5	-1.3 / -0.5	11.0	C 16 H 13 Cl 2 F N 2 O 2 S

[Mass Spectrum]
Data : EI-37-Code-LR-104-F2-016 Date : 04-Jul-2026 09:53
Sample: High resolution GC Mass (JMS-700)
Note : SMLab-Mass Analysis & Research Center
Inlet : Direct Ion Mode : EI+
Spectrum Type : Normal Ion [MF-Linear]
RT : 1.17 min Scan# : (35,37)
BP : m/z 330.9807 Int. : 62.03
Output m/z range : 385.6056 to 386.3035 Cut Level : 73.53 %

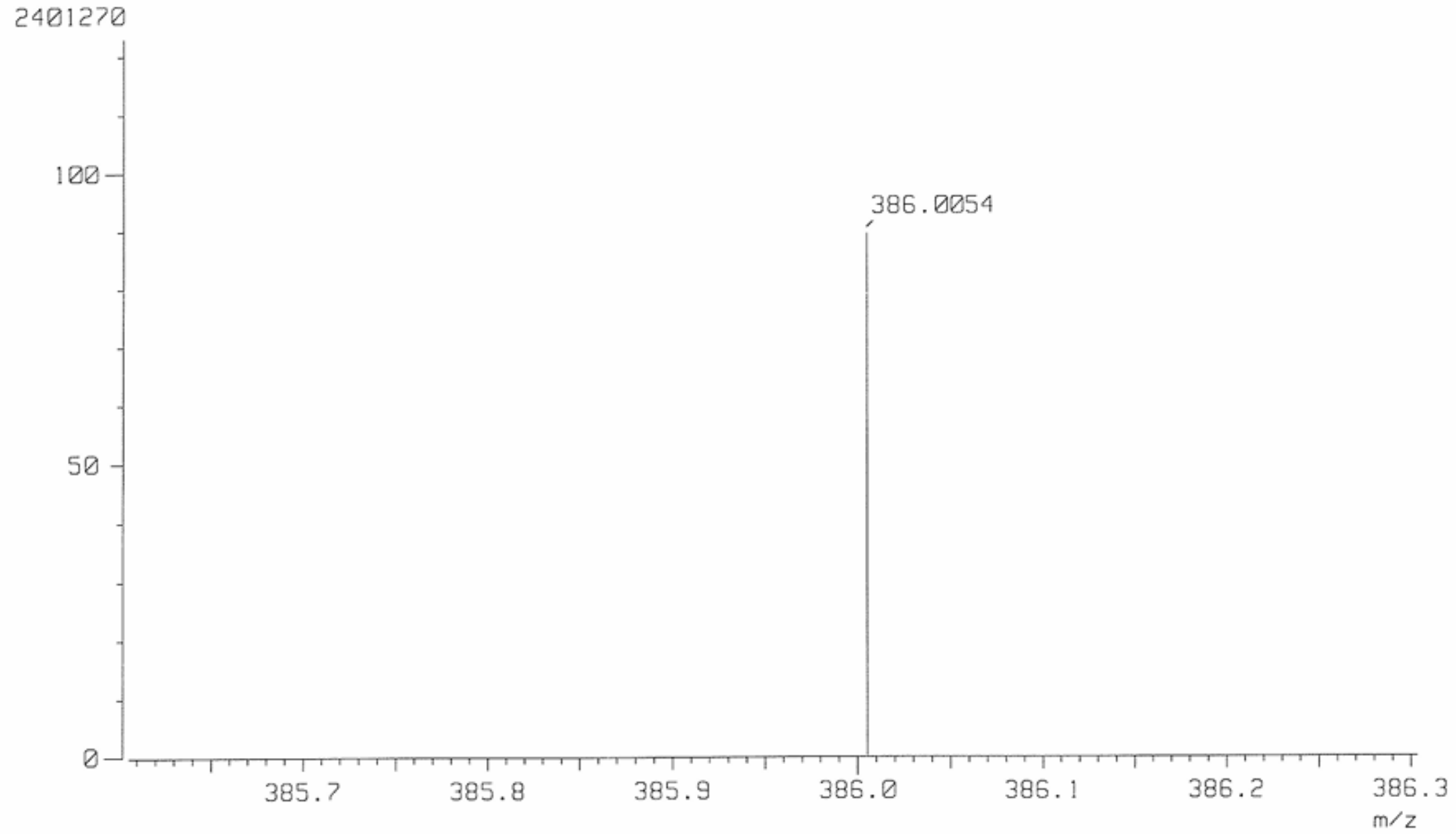

Compound 5h

```
[ Elemental Composition ]                                              Page: 1
Data   : EI-35-Code-LR-102-F2-009     Date : 04-Jul-2026 09:41
Sample: High resolution GC Mass (JMS-700)
Note : SMLab-Mass Analysis & Research Center
Inlet : Direct                        Ion Mode : EI+
RT : 0.84 min                         Scan#: 26
Elements : C 17/0, H 14/0, F 3/0, N 2/0, O 3/0, S 1/0
Mass Tolerance       : 1000ppm, 3mmu if m/z < 3, 5mmu if m/z > 5
Unsaturation (U.S.) : -0.5 - 80.0

Observed m/z Int%   Err[ppm / mmu]    U.S.  Composition
  354.0650    64.4      +0.1 /  +0.0    11.0  C 16 H 13 F 3 N 2 O 2 S
```

```
[ Mass Spectrum ]
Data   : EI-35-Code-LR-102-F2-009     Date : 04-Jul-2026 09:41
Sample: High resolution GC Mass (JMS-700)
Note : SMLab-Mass Analysis & Research Center
Inlet : Direct                        Ion Mode : EI+
Spectrum Type : Normal Ion [MF-Linear]
RT : 0.84 min          Scan# : 26
BP : m/z 330.9814      Int.   : 49.04
Output m/z range : 353.7044 to 354.3787      Cut Level : 46.32 %
```

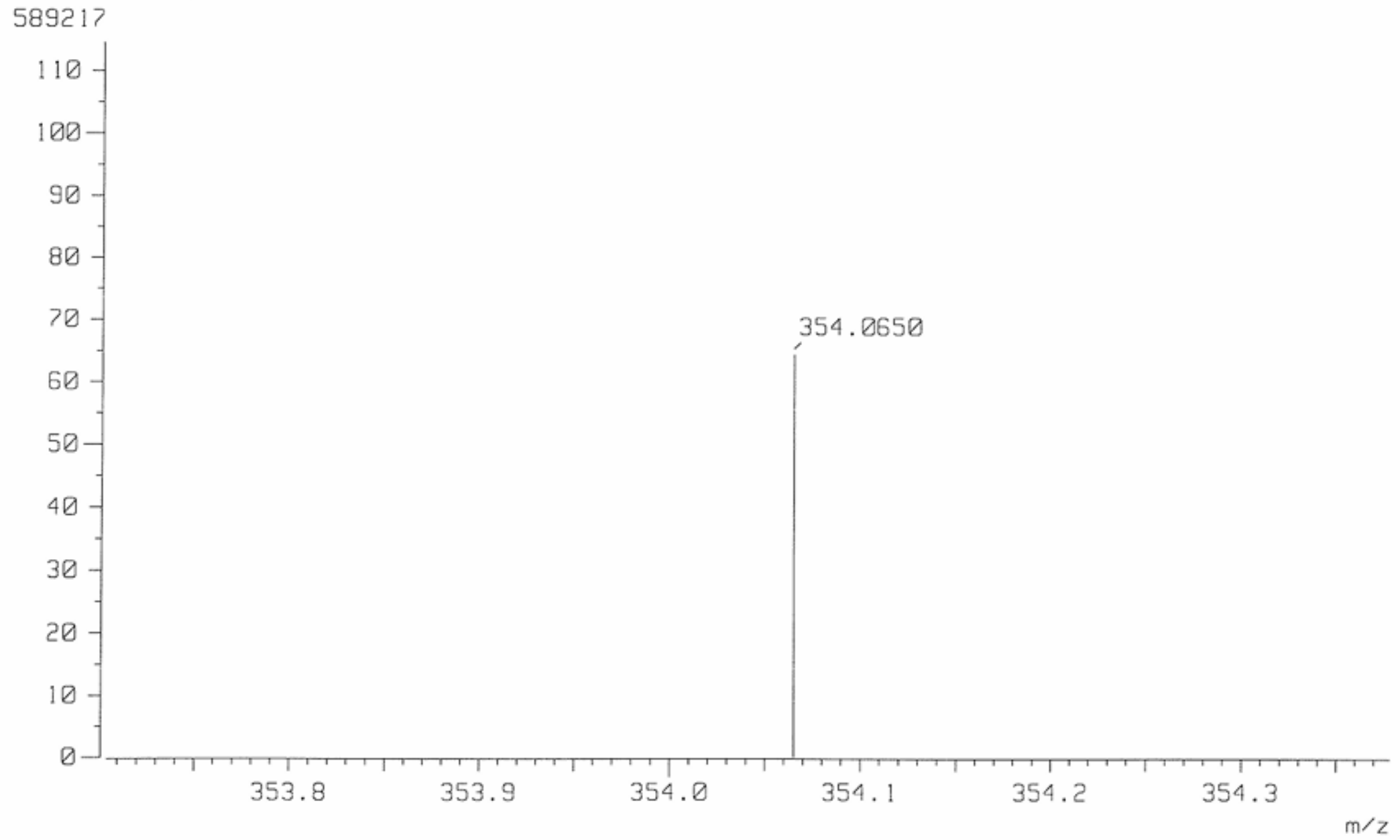

Compound 5i

[Elemental Composition] Page: 1
Data : EI-38-Code-LR-107-F2-027 Date : 04-Jul-2026 10:12
Sample: High resolution GC Mass (JMS-700)
Note : SMLab-Mass Analysis & Research Center
Inlet : Direct Ion Mode : EI+
RT : 0.62 min Scan#: (19,20)
Elements : C 17/0, H 14/0, Br 2/0, F 1/0, N 2/0, O 3/0, S 1/0
Mass Tolerance : 1000ppm, 3mmu if m/z < 3, 5mmu if m/z > 5
Unsaturation (U.S.) : -0.5 - 80.0

Observed m/z	Int%	Err[ppm / mmu]	U.S.	Composition
473.9047	52.1	-0.3 / -0.2	11.0	C 16 H 13 Br 2 F N 2 O 2 S

[Mass Spectrum]
Data : EI-38-Code-LR-107-F2-027 Date : 04-Jul-2026 10:12
Sample: High resolution GC Mass (JMS-700)
Note : SMLab-Mass Analysis & Research Center
Inlet : Direct Ion Mode : EI+
Spectrum Type : Normal Ion [MF-Linear]
RT : 0.62 min Scan# : (19,20)
BP : m/z 475.9027 Int. : 40.35
Output m/z range : 473.5868 to 474.1896 Cut Level : 37.50 %

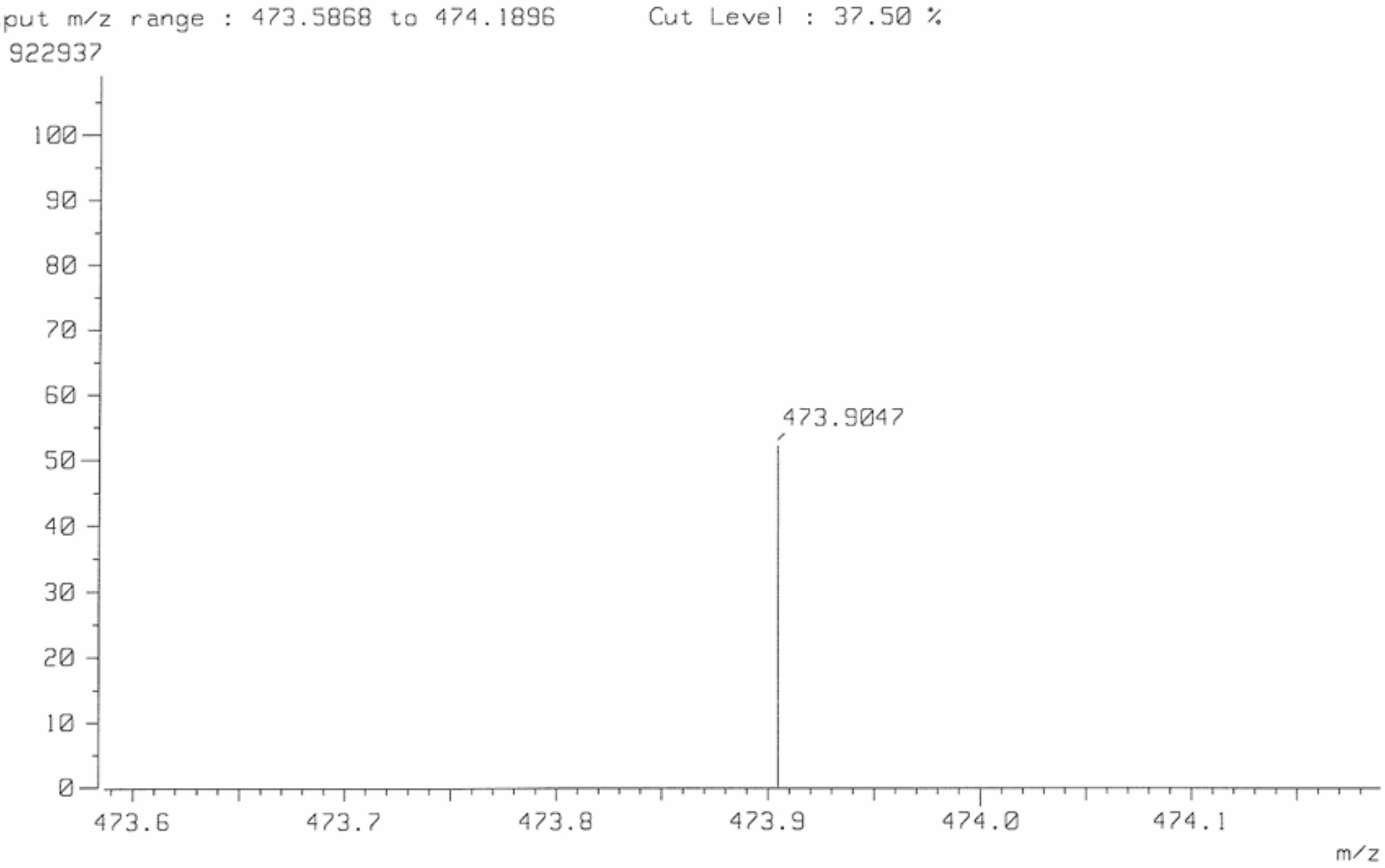

Compound 5j

[Elemental Composition]   Page: 1
Data   : EI-15-HS-261-024   Date : 03-Jul-2026 16:01
Sample: High resolution GC Mass (JMS-700)
Note : SMLab-Mass Analysis & Research Center
Inlet : Direct   Ion Mode : EI+
RT : 1.27 min   Scan#: 39+40+39
Elements : C 18/0, H 15/0, F 3/0, N 2/0, O 3/0, S 2/0
Mass Tolerance   : 1000ppm, 3mmu if m/z < 3, 5mmu if m/z > 5
Unsaturation (U.S.) : -0.5 - 80.0

Observed m/z	Int%	Err[ppm / mmu]	U.S.	Composition
354.0650	96.6	+0.1 / +0.0	11.0	C 16 H 13 F 3 N 2 O 2 S

[Mass Spectrum]
Data   : EI-15-HS-261-024   Date : 03-Jul-2026 16:01
Sample: High resolution GC Mass (JMS-700)
Note : SMLab-Mass Analysis & Research Center
Inlet : Direct   Ion Mode : EI+
Spectrum Type : Normal Ion [MF-Linear]
RT : 1.27 min   Scan# : 39+40+39
BP : m/z 330.9821   Int. : 40.23
Output m/z range : 353.6933 to 354.3838   Cut Level : 69.12 %
2117045

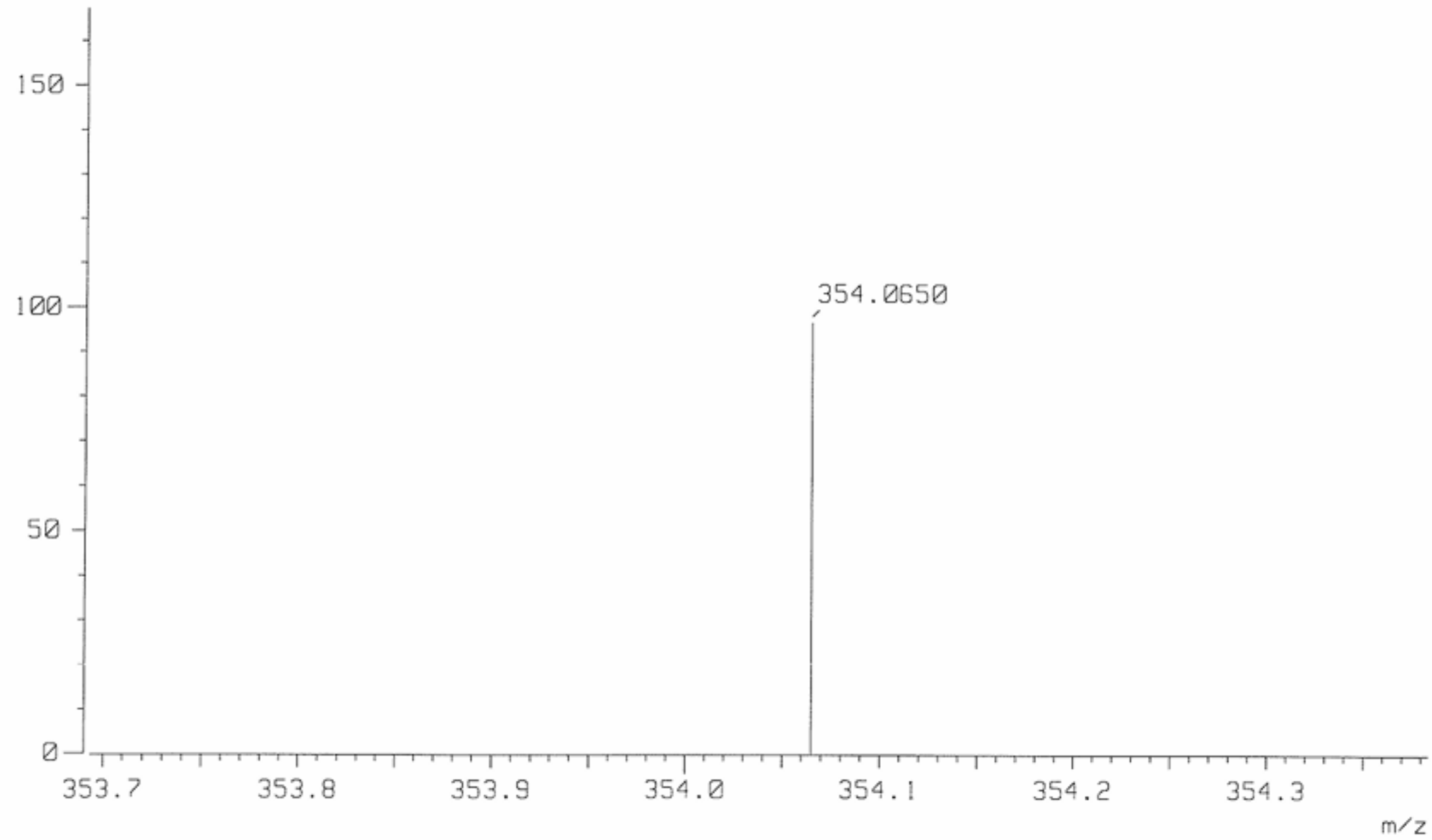

Compound 5k

[Elemental Composition]
Page: 1
Data : EI-8-HS-237-019 Date : 03-Jul-2026 15:54
Sample: High resolution GC Mass (JMS-700)
Note : SMLab-Mass Analysis & Research Center
Inlet : Direct Ion Mode : EI+
RT : 0.57 min Scan#: 18
Elements : C 17/0, H 14/0, F 3/0, N 2/0, O 2/0, S 1/0
Mass Tolerance : 1000ppm, 3mmu if m/z < 3, 5mmu if m/z > 5
Unsaturation (U.S.) : -0.5 - 80.0

Observed m/z	Int%	Err[ppm / mmu]	U.S.	Composition
354.0647	100.0	-0.8 / -0.3	11.0	C 16 H 13 F 3 N 2 O 2 S

[Mass Spectrum]
Data : EI-8-HS-237-019 Date : 03-Jul-2026 15:54
Sample: High resolution GC Mass (JMS-700)
Note : SMLab-Mass Analysis & Research Center
Inlet : Direct Ion Mode : EI+
Spectrum Type : Normal Ion [MF-Linear]
RT : 0.57 min Scan# : 18
BP : m/z 354.0647 Int. : 65.15
Output m/z range : 353.7457 to 354.3908 Cut Level : 39.33 %

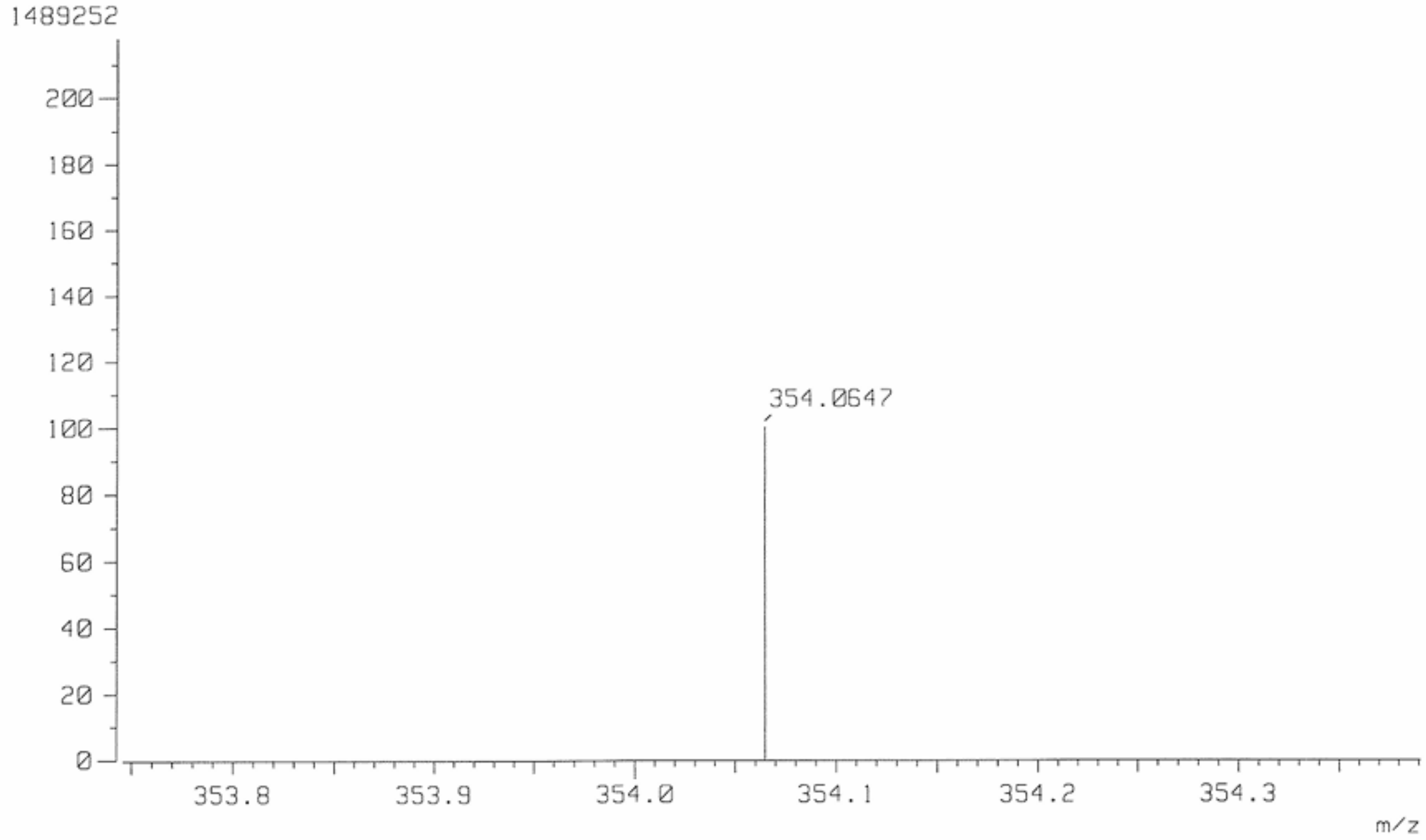

Compound 5l

```
[ Elemental Composition ]                                              Page: 1
Data   : EI-7-HS-236-017                Date : 03-Jul-2026 15:50
Sample: High resolution GC Mass (JMS-700)
Note : SMLab-Mass Analysis & Research Center
Inlet : Direct                          Ion Mode : EI+
RT : 1.32 min                           Scan#: (38,43)
Elements : C 17/0, H 15/0, F 3/0, N 2/0, O 2/0, S 1/0
Mass Tolerance        : 1000ppm, 3mmu if m/z < 3, 5mmu if m/z > 5
Unsaturation (U.S.) : -0.5 - 80.0

Observed m/z Int%   Err[ppm / mmu]    U.S.  Composition
  354.0650   100.0     +0.0 /  +0.0   11.0  C 16 H 13 F 3 N 2 O 2 S
```

```
[ Mass Spectrum ]
Data   : EI-7-HS-236-017              Date : 03-Jul-2026 15:50
Sample: High resolution GC Mass (JMS-700)
Note : SMLab-Mass Analysis & Research Center
Inlet : Direct                        Ion Mode : EI+
Spectrum Type : Normal Ion [MF-Linear]
RT : 1.32 min          Scan# : (38,43)
BP : m/z 354.0650      Int.   : 20.58
Output m/z range : 353.6934 to 354.3286      Cut Level : 62.76 %
```

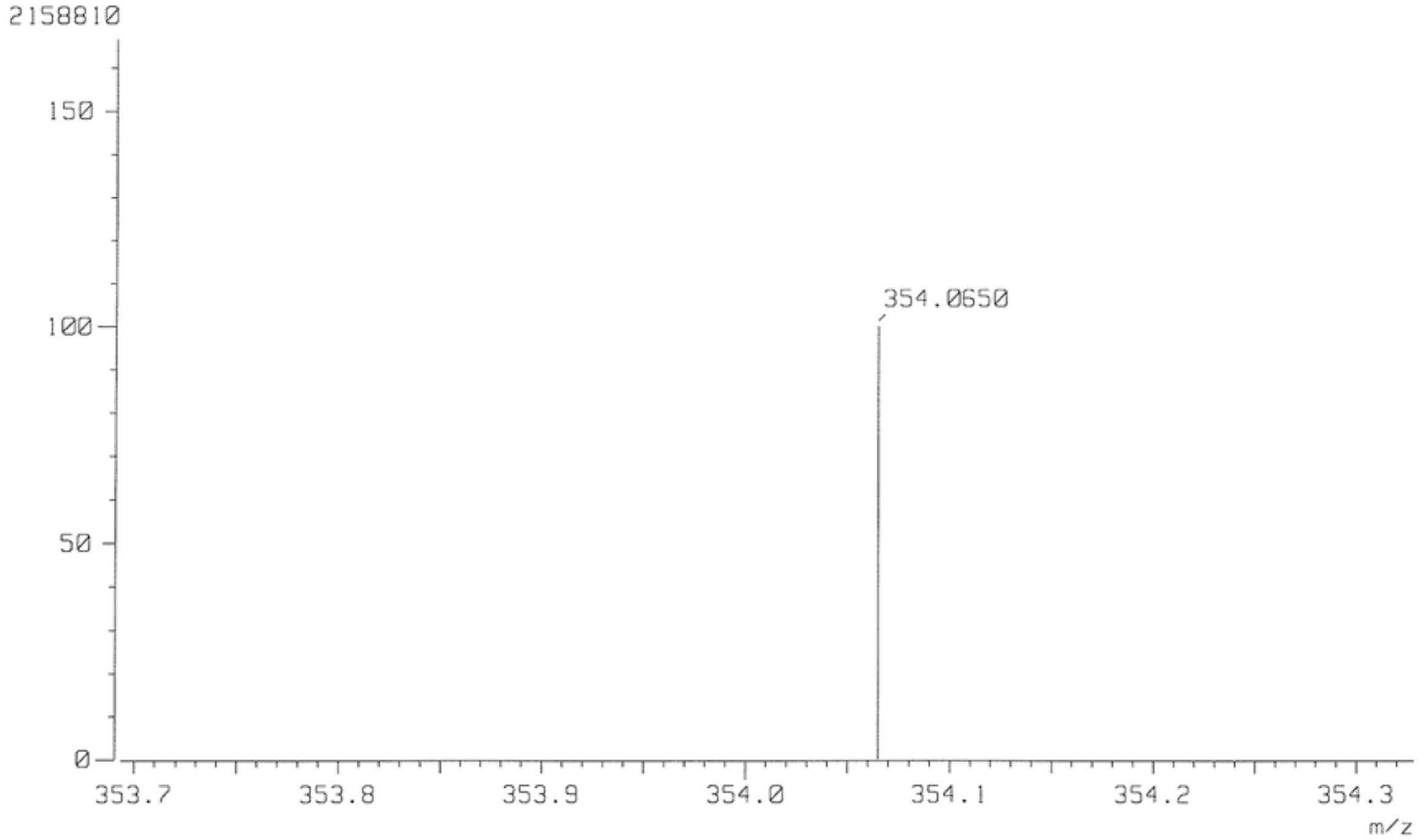

Compound 5m

[Elemental Composition] Page: 1
Data : EI-21-HS-268-001 Date : 04-Jul-2026 08:52
Sample: High resolution GC Mass (JMS-700)
Note : SMLab-Mass Analysis & Research Center
Inlet : Direct Ion Mode : EI+
RT : 1.74 min Scan#: 53
Elements : C 17/0, H 14/0, Cl 2/0, F 1/0, N 2/0, O 3/0, S 1/0
Mass Tolerance : 1000ppm, 3mmu if m/z < 3, 5mmu if m/z > 5
Unsaturation (U.S.) : -0.5 - 80.0

Observed m/z	Int%	Err[ppm / mmu]	U.S.	Composition
386.0057	100.0	-0.5 / -0.2	11.0	C 16 H 13 Cl 2 F N 2 O 2 S

[Mass Spectrum]
Data : EI-21-HS-268-001 Date : 04-Jul-2026 08:52
Sample: High resolution GC Mass (JMS-700)
Note : SMLab-Mass Analysis & Research Center
Inlet : Direct Ion Mode : EI+
Spectrum Type : Normal Ion [MF-Linear]
RT : 1.74 min Scan# : 53
BP : m/z 386.0057 Int. : 86.93
Output m/z range : 385.6309 to 386.3767 Cut Level : 80.15 %

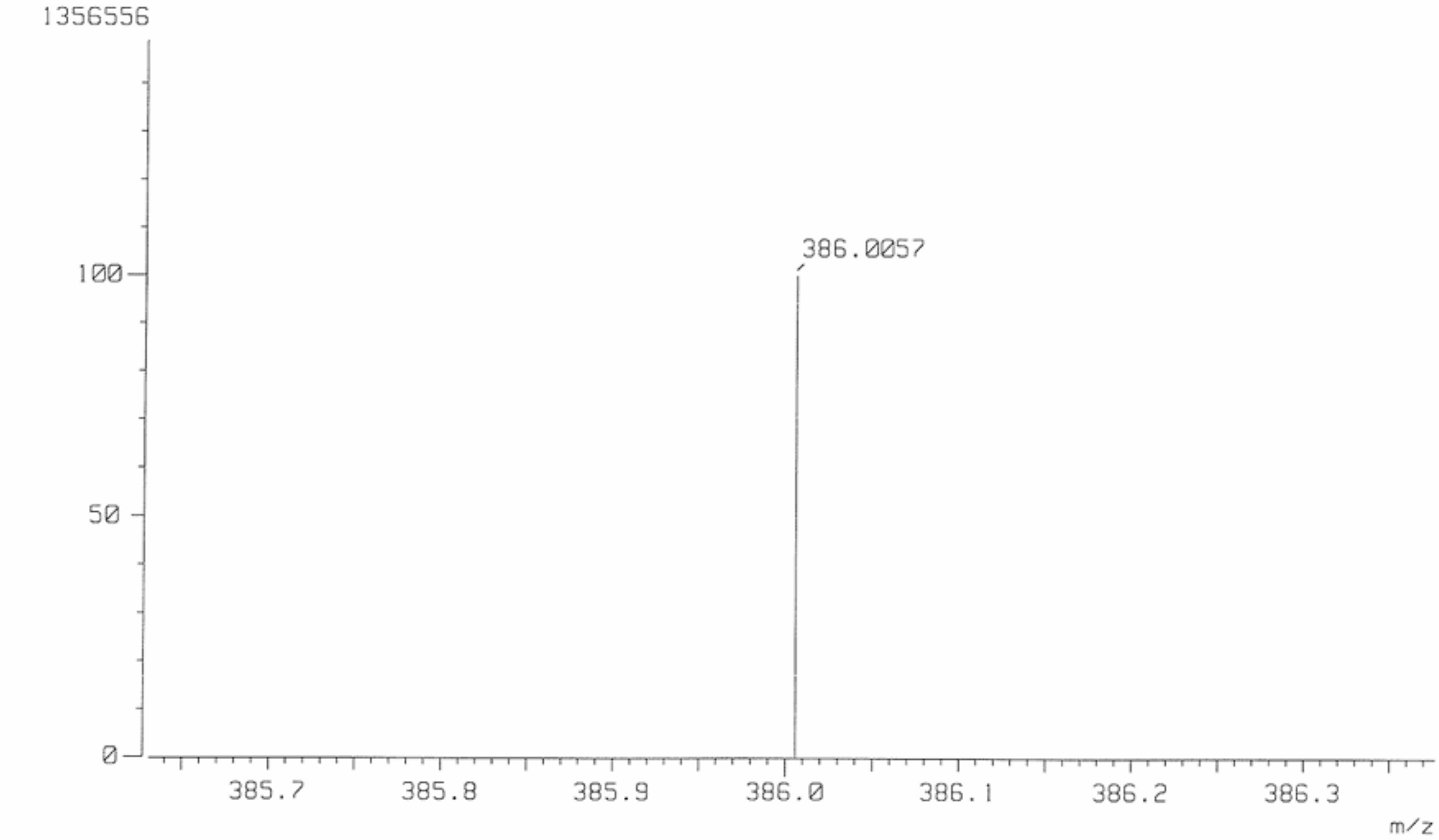

Compound 5n

```
[ Elemental Composition ]                                          Page: 1
Data   : EI-HS288-HR006                 Date : 04-Jul-2026 10:46
Sample: High resolution GC Mass (JMS-700)
Note : SMLab-Mass Analysis & Research Center
Inlet : Direct                          Ion Mode : EI+
RT : 1.22 min                           Scan#: (37,38)
Elements : C 20/0, H 20/0, F 1/0, N 2/0, O 3/0, S 1/0
Mass Tolerance       : 1000ppm, 3mmu if m/z < 3, 5mmu if m/z > 5
Unsaturation (U.S.) : -0.5 - 80.0

Observed m/z Int%   Err[ppm / mmu]    U.S.  Composition
  346.1153     7.4     +0.5 /  +0.2   11.0  C 18 H 19 F N 2 O 2 S
```

```
[ Mass Spectrum ]
Data   : EI-HS288-HR006              Date : 04-Jul-2026 10:46
Sample: High resolution GC Mass (JMS-700)
Note : SMLab-Mass Analysis & Research Center
Inlet : Direct                       Ion Mode : EI+
Spectrum Type : Normal Ion [MF-Linear]
RT : 1.22 min          Scan# : (37,38)
BP : m/z 322.1659      Int.  : 44.68
Output m/z range : 345.8145 to 346.4016      Cut Level : 7.35 %
```

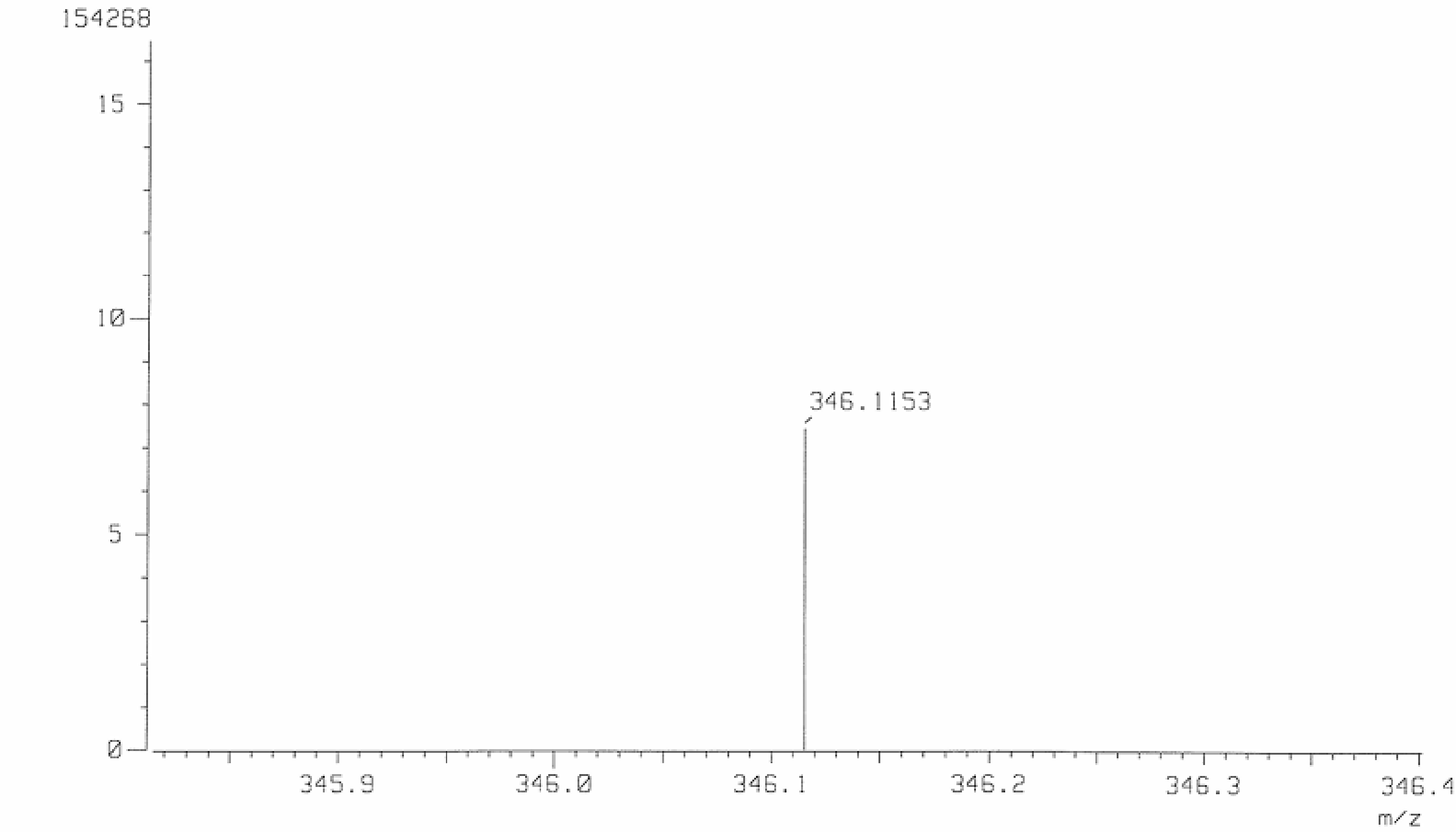

Compound 5o

```
[ Elemental Composition ]                                              Page: 1
Data   : EI-27-HS-294-004              Date : 04-Jul-2026 10:18
Sample: High resolution GC Mass (JMS-700)
Note : SMLab-Mass Analysis & Research Center
Inlet : Direct                         Ion Mode : EI+
RT : 1.02 min                          Scan#: (30,33)
Elements : C 20/0, H 20/0, F 1/0, N 2/0, O 6/0, S 1/0
Mass Tolerance      : 1000ppm, 3mmu if m/z < 3, 5mmu if m/z > 5
Unsaturation (U.S.) : -0.5 - 80.0

Observed m/z Int%   Err[ppm / mmu]    U.S.  Composition
  410.0946   100.0     -0.4 /  -0.2   11.0  C 18 H 19 F N 2 O 6 S
```

```
[ Mass Spectrum ]
Data   : EI-27-HS-294-004              Date : 04-Jul-2026 10:18
Sample: High resolution GC Mass (JMS-700)
Note : SMLab-Mass Analysis & Research Center
Inlet : Direct                         Ion Mode : EI+
Spectrum Type : Normal Ion [MF-Linear]
RT : 1.02 min          Scan# : (30,33)
BP : m/z 410.0946      Int.  : 74.04
Output m/z range : 409.7812 to 410.3832      Cut Level : 72.28 %
```

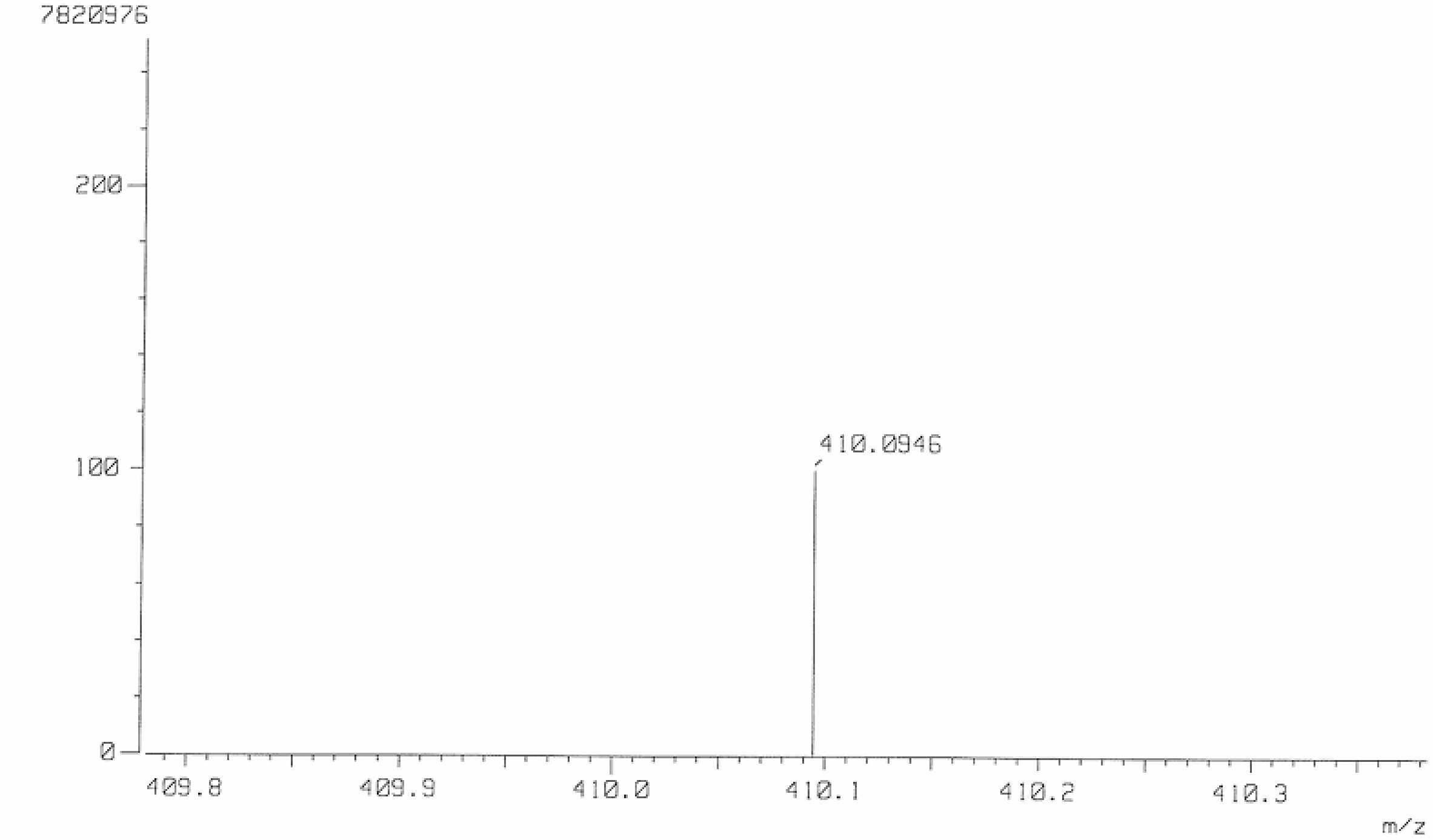

Compound 5p

```
[ Elemental Composition ]                                              Page: 1
Data   : EI-26-HS-293-003                  Date : 04-Jul-2026 10:16
Sample: High resolution GC Mass (JMS-700)
Note : SMLab-Mass Analysis & Research Center
Inlet : Direct                             Ion Mode : EI+
RT : 0.35 min                              Scan#: (11,12)+10+7
Elements : C 15/0, H 9/0, Cl 4/0, F 1/0, N 2/0, O 3/0, S 1/0
Mass Tolerance      : 1000ppm, 3mmu if m/z < 3, 5mmu if m/z > 5
Unsaturation (U.S.) : -0.5 - 80.0

Observed m/z Int%   Err[ppm / mmu]   U.S.  Composition
  425.8961    75.0     -1.4 /  -0.6   11.0  C 14 H 7 Cl 4 F N 2 O 2 S
```

```
[ Mass Spectrum ]
Data   : EI-26-HS-293-003              Date : 04-Jul-2026 10:16
Sample: High resolution GC Mass (JMS-700)
Note : SMLab-Mass Analysis & Research Center
Inlet : Direct                         Ion Mode : EI+
Spectrum Type : Normal Ion [MF-Linear]
RT : 0.35 min          Scan# : (11,12)+10+7
BP : m/z 427.8942      Int.   : 72.72
Output m/z range : 425.4651 to 426.2671      Cut Level : 51.47 %
```

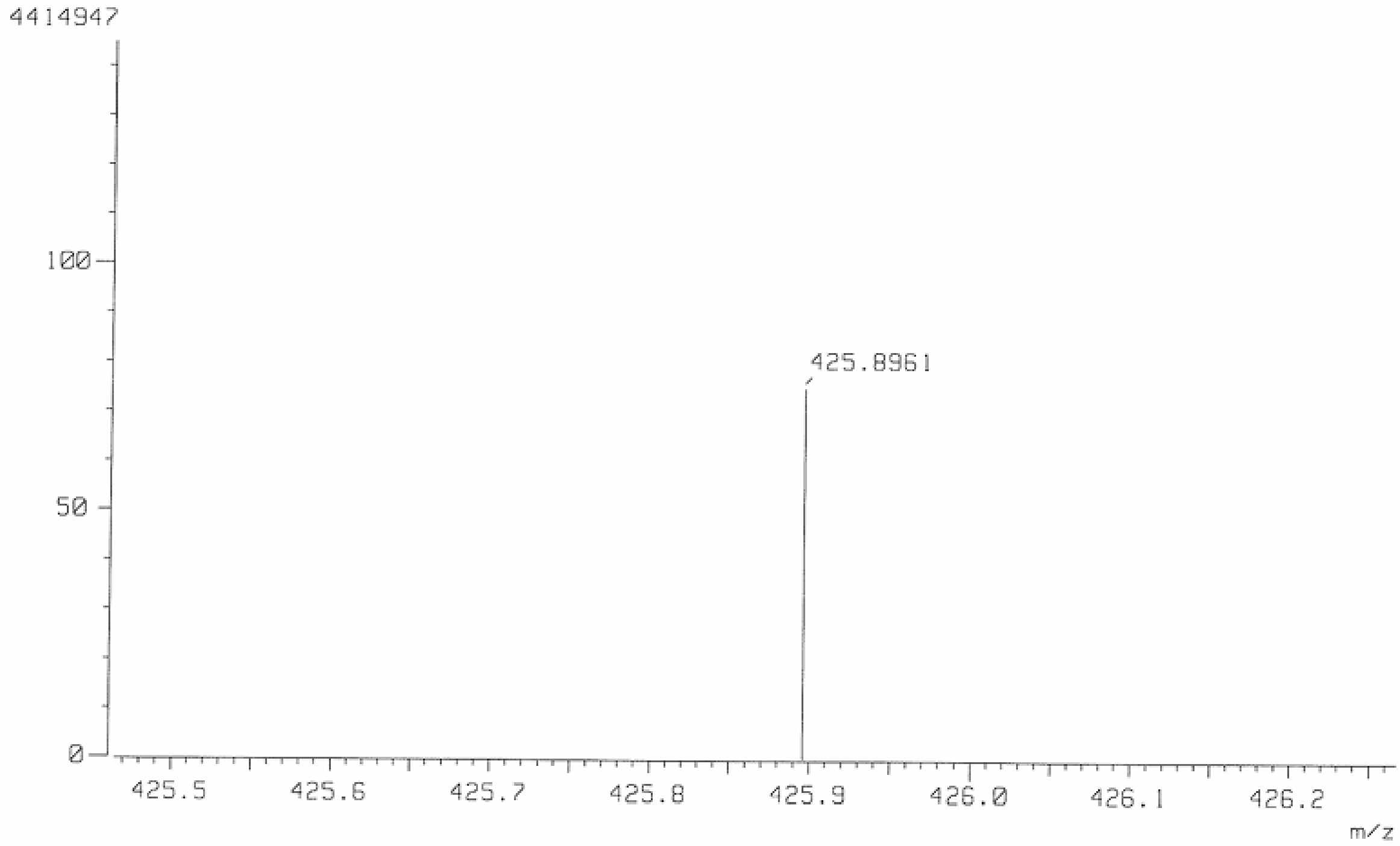

Compound 5q and 5q'

[Elemental Composition]
Data : EI-4-HS-345-P-010 Date : 16-Sep-2026 15:26 Page: 1
Sample: High resolution GC Mass (JMS-700)
Note : SMLab-Mass Analysis & Research Center
Inlet : Direct Ion Mode : EI+
RT : 0.14 min Scan#: 5+8
Elements : C 18/0, H 20/0, F 1/0, N 2/0, O 2/0, S 1/0
Mass Tolerance : 1000ppm, 3mmu if m/z < 3, 5mmu if m/z > 5
Unsaturation (U.S.) : -0.5 - 80.0

Observed m/z	Int%	Err[ppm / mmu]	U.S.	Composition
332.0991	100.0	-1.3 / -0.4	11.0	C 17 H 17 F N 2 O 2 S

[Mass Spectrum]
Data : EI-4-HS-345-P-010 Date : 16-Sep-2026 15:26
Sample: High resolution GC Mass (JMS-700)
Note : SMLab-Mass Analysis & Research Center
Inlet : Direct Ion Mode : EI+
Spectrum Type : Normal Ion [MF-Linear]
RT : 0.14 min Scan# : 5+8
BP : m/z 332.0991 Int. : 247.23
Output m/z range : 331.7353 to 332.4460 Cut Level : 72.79 %

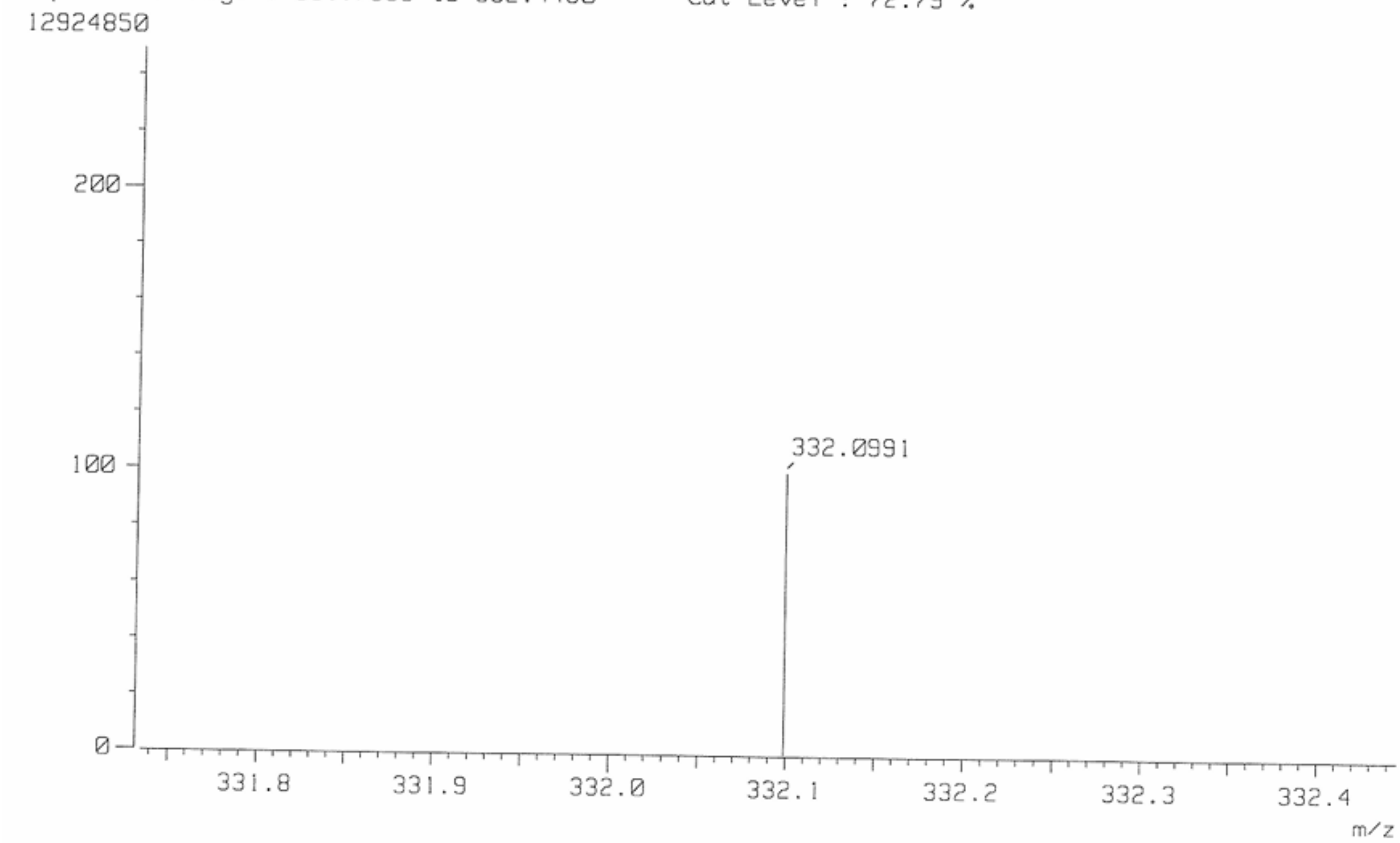

Compound 6a

[Elemental Composition]　　　　　　　　　　　　　　　　　　　　Page: 1
Data : EI-3-HS-335-P-014　　　　　　Date : 16-Sep-2026 15:45
Sample: High resolution GC Mass (JMS-700)
Note : SMLab-Mass Analysis & Research Center
Inlet : Direct　　　　　　　　　　　　Ion Mode : EI+
RT : 0.30 min　　　　　　　　　　　　Scan#: 10
Elements : C 22/0, H 24/0, N 3/0, O 3/0, S 1/0
Mass Tolerance : 1000ppm, 3mmu if m/z < 3, 5mmu if m/z > 5
Unsaturation (U.S.) : -0.5 - 80.0

Observed m/z	Int%	Err[ppm / mmu]	U.S.	Composition
369.1507	100.0	-1.0 / -0.4	12.0	C 20 H 23 N 3 O 2 S

[Mass Spectrum]
Data : EI-3-HS-335-P-014　　　　　　Date : 16-Sep-2026 15:45
Sample: High resolution GC Mass (JMS-700)
Note : SMLab-Mass Analysis & Research Center
Inlet : Direct　　　　　　　　　　　　Ion Mode : EI+
Spectrum Type : Normal Ion [MF-Linear]
RT : 0.30 min　　　　　Scan# : 10
BP : m/z 369.1507　　　Int. : 125.47
Output m/z range : 368.8396 to 369.4700　　　Cut Level : 70.59 %

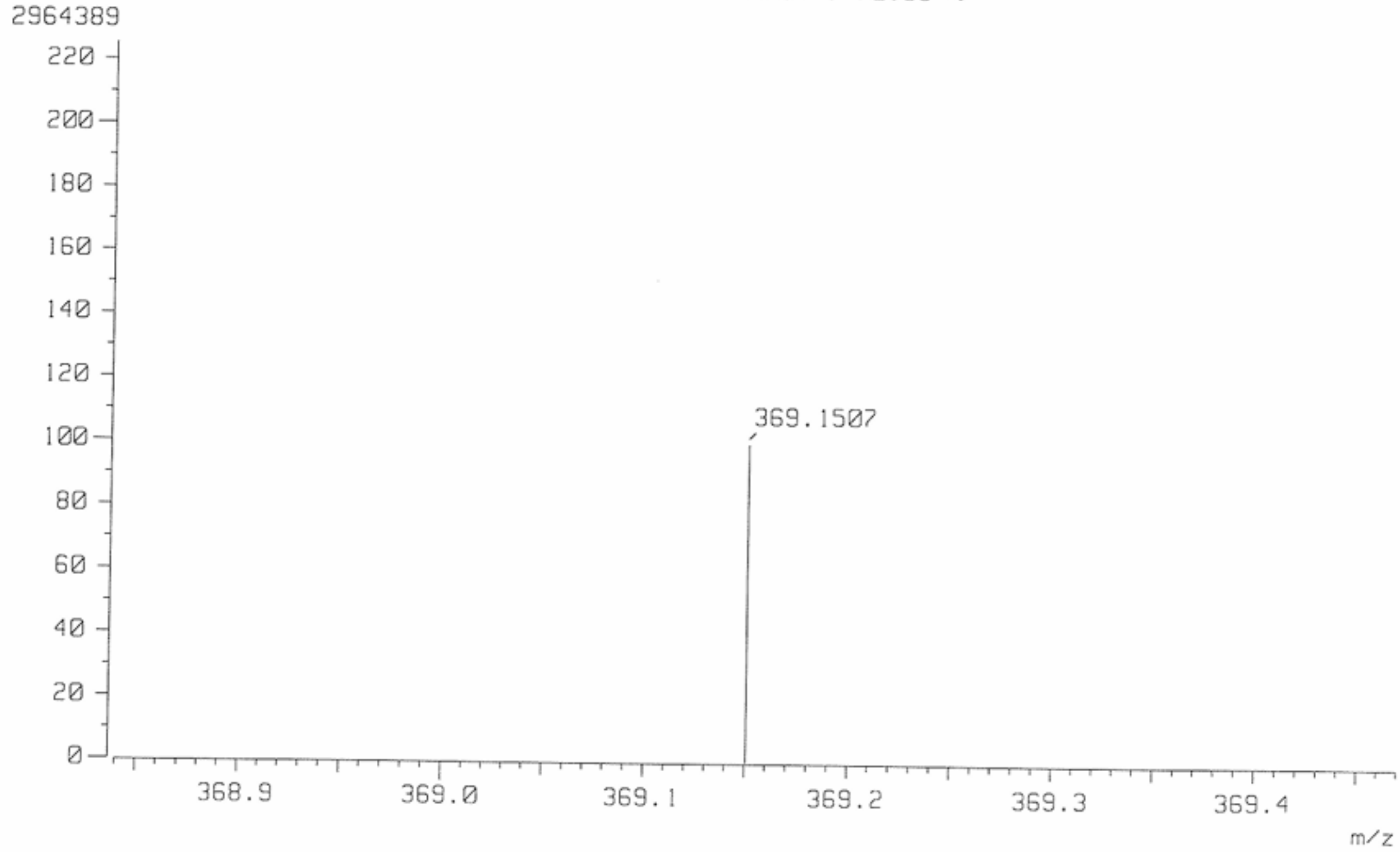

Compound 6b

[Elemental Composition] Page: 1
Data : EI-HS272-re-001 Date : 20-Jul-2026 12:56
Sample: High resolution GC Mass (JMS-700)
Note : SMLab-Mass Analysis & Research Center
Inlet : Direct Ion Mode : EI+
RT : 2.70 min Scan#: 82
Elements : C 29/0, H 40/0, N 4/0, O 7/0, S 1/0
Mass Tolerance : 1000ppm, 3mmu if m/z < 3, 5mmu if m/z > 5
Unsaturation (U.S.) : -0.5 - 80.0

Observed m/z	Int%	Err[ppm / mmu]	U.S.	Composition
558.2509	42.7	-0.6 / -0.4	13.0	C 28 H 38 N 4 O 6 S

[Mass Spectrum]
Data : EI-HS272-re-001 Date : 20-Jul-2026 12:56
Sample: High resolution GC Mass (JMS-700)
Note : SMLab-Mass Analysis & Research Center
Inlet : Direct Ion Mode : EI+
Spectrum Type : Normal Ion [MF-Linear]
RT : 2.70 min Scan# : 82
BP : m/z 508.2835 Int. : 69.90
Output m/z range : 557.9486 to 558.5346 Cut Level : 30.88 %

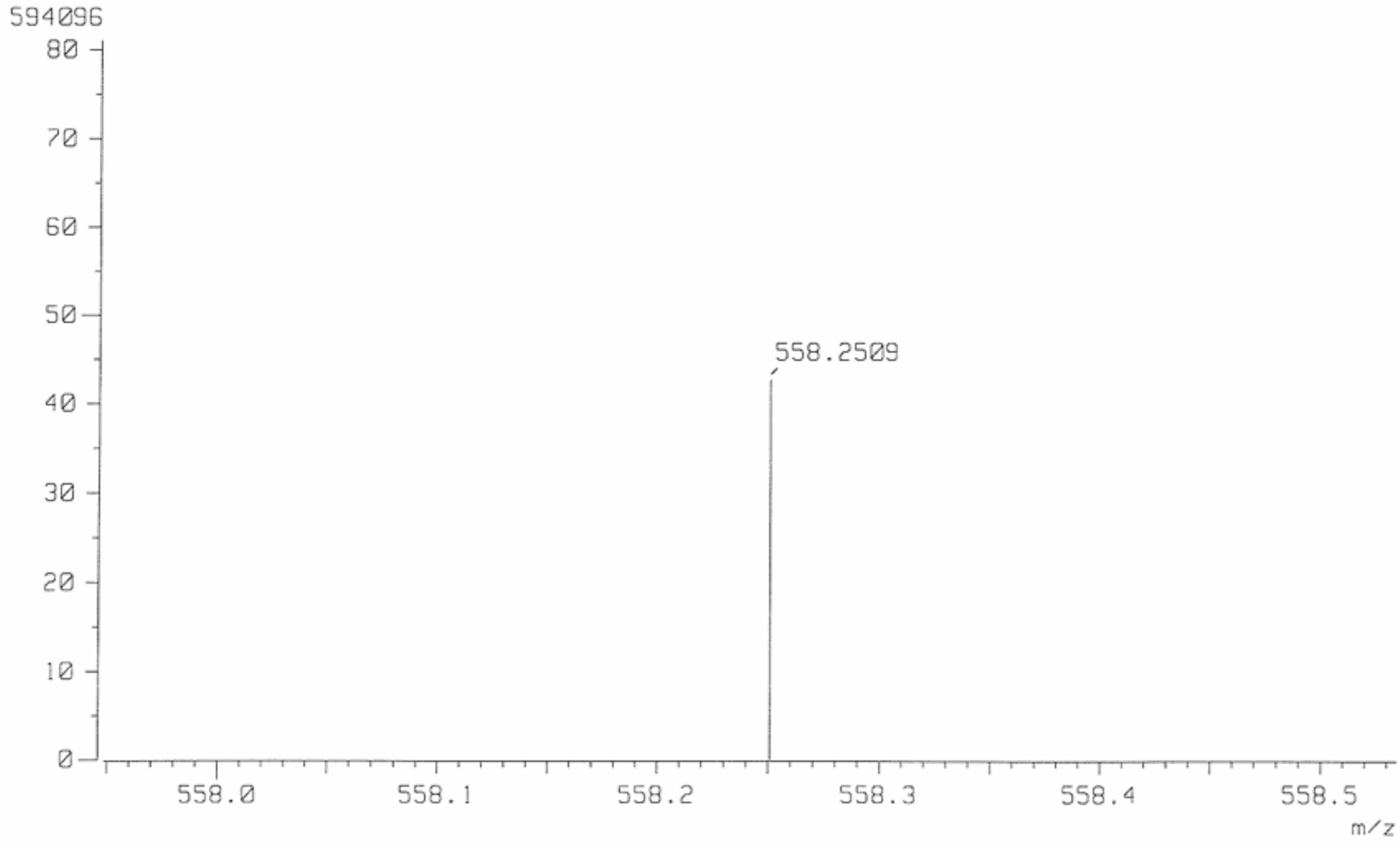

Compound 6c

```
[ Elemental Composition ]                                                  Page: 1
Data   : EI-10-HS-246-004               Date : 03-Jul-2026 16:30
Sample: High resolution GC Mass (JMS-700)
Note : SMLab-Mass Analysis & Research Center
Inlet : Direct                          Ion Mode : EI+
RT : 0.29 min                           Scan#: (9,10)+18
Elements : C 33/0, H 36/0, N 3/0, O 8/0, S 1/0
Mass Tolerance      : 1000ppm, 3mmu if m/z < 3, 5mmu if m/z > 5
Unsaturation (U.S.) : -0.5 - 80.0

Observed m/z Int%   Err[ppm / mmu]    U.S.  Composition
  593.2191   100.0     -0.8 /  -0.5   17.0  C 31 H 35 N 3 O 7 S
```

```
[ Mass Spectrum ]
Data   : EI-10-HS-246-004           Date : 03-Jul-2026 16:30
Sample: High resolution GC Mass (JMS-700)
Note : SMLab-Mass Analysis & Research Center
Inlet : Direct                      Ion Mode : EI+
Spectrum Type : Normal Ion [MF-Linear]
RT : 0.29 min          Scan# : (9,10)+18
BP : m/z 593.2191      Int.   : 24.80
Output m/z range : 592.8818 to 593.5088       Cut Level : 48.55 %
```

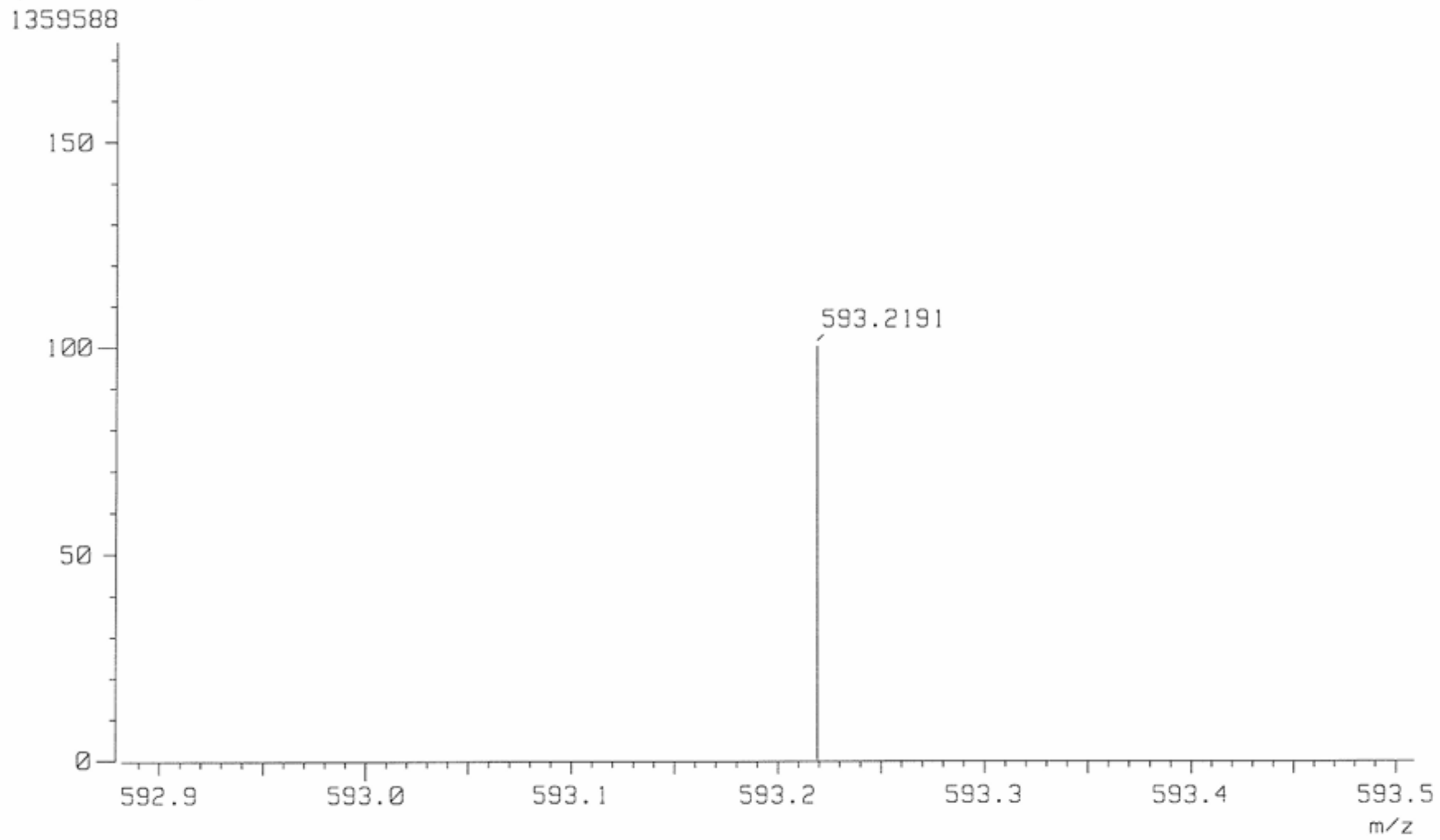

Compound 6d

[Elemental Composition]　　Page: 1
Data : FAB-HS303-R-020　　Date : 19-Jul-2026 11:57
Sample: -
Note : -
Inlet : Direct　　Ion Mode : FAB-
RT : 4.20 min　　Scan#: (84,86)
Elements : C 30/0, H 27/0, N 5/0, O 5/0, S 1/0
Mass Tolerance : 1000ppm, 3mmu if m/z < 3, 5mmu if m/z > 5
Unsaturation (U.S.) : -0.5 - 80.0

Observed m/z	Int%	Err[ppm / mmu]	U.S.	Composition
556.1662	14.9	+1.3 / +0.7	20.5	C 29 H 26 N 5 O 5 S

[Mass Spectrum]
Data : FAB-HS303-R-020　　Date : 19-Jul-2026 11:57
Sample: -
Note : -
Inlet : Direct　　Ion Mode : FAB-
Spectrum Type : Normal Ion [MF-Linear]
RT : 4.20 min　　Scan# : (84,86)
BP : m/z 459.1310　　Int. : 6.60
Output m/z range : 555.8041 to 556.4866　　Cut Level : 9.56 %

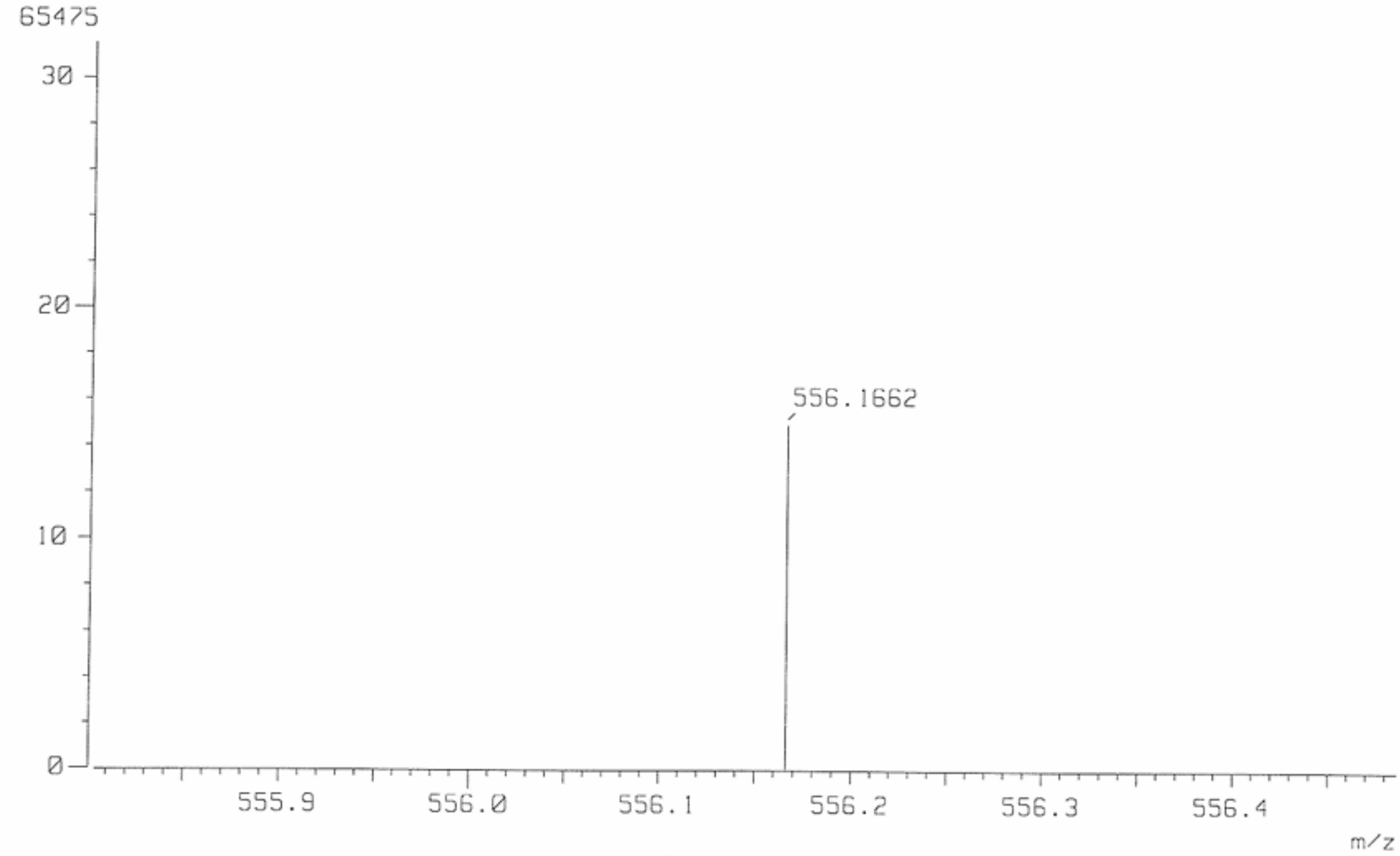

Compound 6e

[Elemental Composition] Page: 1

Data : EI-11-HS-247-006 Date : 03-Jul-2026 16:36
Sample: High resolution GC Mass (JMS-700)
Note : SMLab-Mass Analysis & Research Center
Inlet : Direct Ion Mode : EI+
RT : 0.30 min Scan#: 10+10+11
Elements : C 35/0, H 37/0, N 2/0, O 5/0, S 1/0
Mass Tolerance : 1000ppm, 3mmu if m/z < 3, 5mmu if m/z > 5
Unsaturation (U.S.) : -0.5 - 80.0

Observed m/z	Int%	Err[ppm / mmu]	U.S.	Composition
568.2395	100.0	-0.1 / -0.1	19.0	C 34 H 36 N 2 O 4 S

[Mass Spectrum]
Data : EI-11-HS-247-006 Date : 03-Jul-2026 16:36
Sample: High resolution GC Mass (JMS-700)
Note : SMLab-Mass Analysis & Research Center
Inlet : Direct Ion Mode : EI+
Spectrum Type : Normal Ion [MF-Linear]
RT : 0.30 min Scan# : 10+10+11
BP : m/z 568.2395 Int. : 71.42
Output m/z range : 567.9261 to 568.5631 Cut Level : 71.32 %

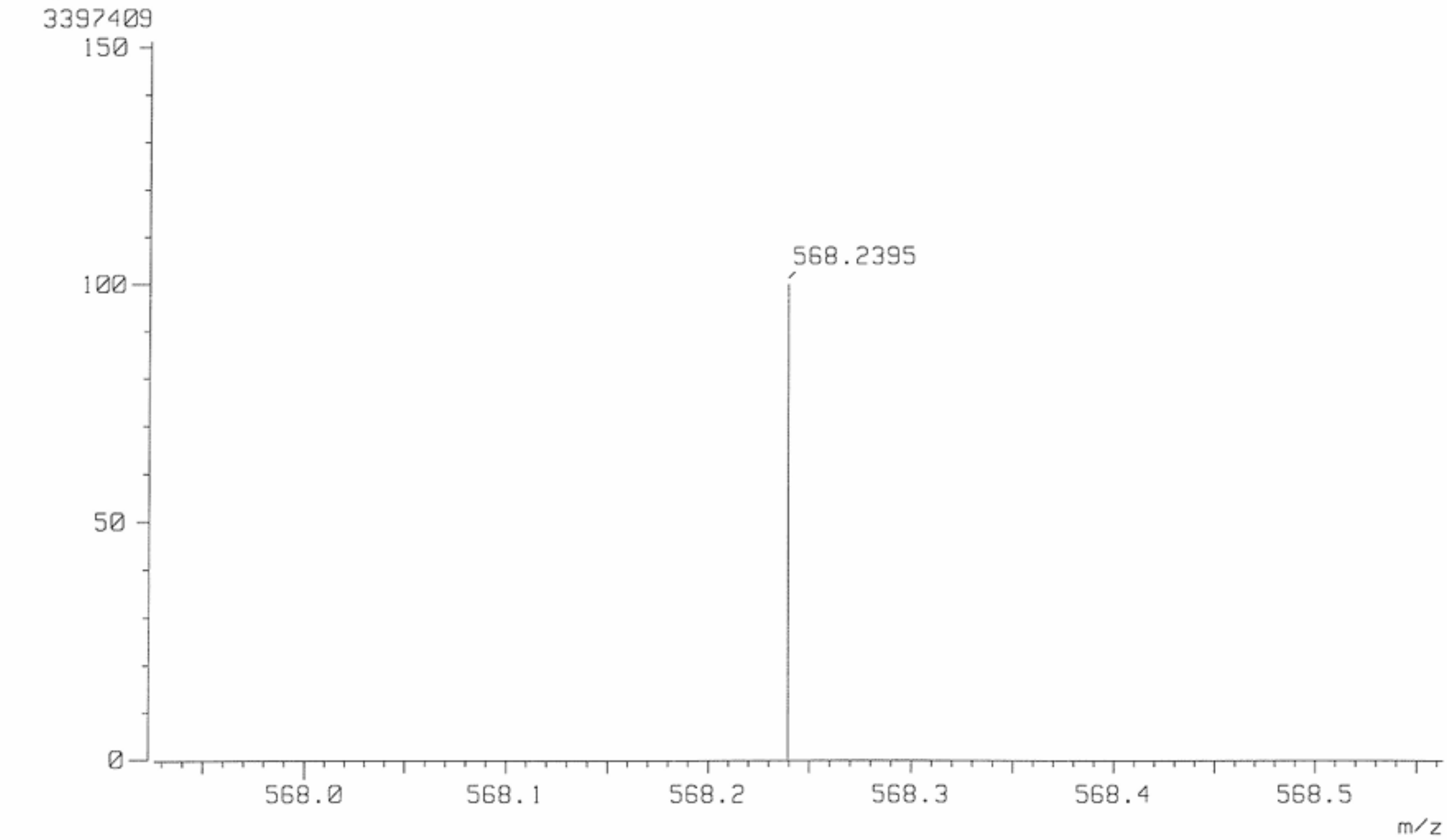

Compound 6f

[Elemental Composition]
Data : FAB-HS-334-P-007 Date : 16-Sep-2026 16:56 Page: 1
Sample: High resolution GC Mass(JMS-700)
Note : SMlab-Mass Analysis & Research Center
Inlet : Direct Ion Mode : FAB+
RT : 1.44 min Scan#: (42,46)+14+6
Elements : C 32/0, H 25/0, N 2/0, O 6/0, S 1/0
Mass Tolerance : 1000ppm, 3mmu if m/z < 3, 5mmu if m/z > 5
Unsaturation (U.S.) : -0.5 - 80.0

Observed m/z	Int%	Err[ppm / mmu]	U.S.	Composition
537.1486	96.8	+0.3 / +0.1	21.5	C 31 H 25 N 2 O 5 S

[Mass Spectrum]
Data : FAB-HS-334-P-007 Date : 16-Sep-2026 16:56
Sample: High resolution GC Mass(JMS-700)
Note : SMlab-Mass Analysis & Research Center
Inlet : Direct Ion Mode : FAB+
Spectrum Type : Normal Ion [MF-Linear]
RT : 1.44 min Scan# : (42,46)+14+6
BP : m/z 473.1785 Int. : 16.07
Output m/z range : 536.9069 to 537.4038 Cut Level : 55.01 %

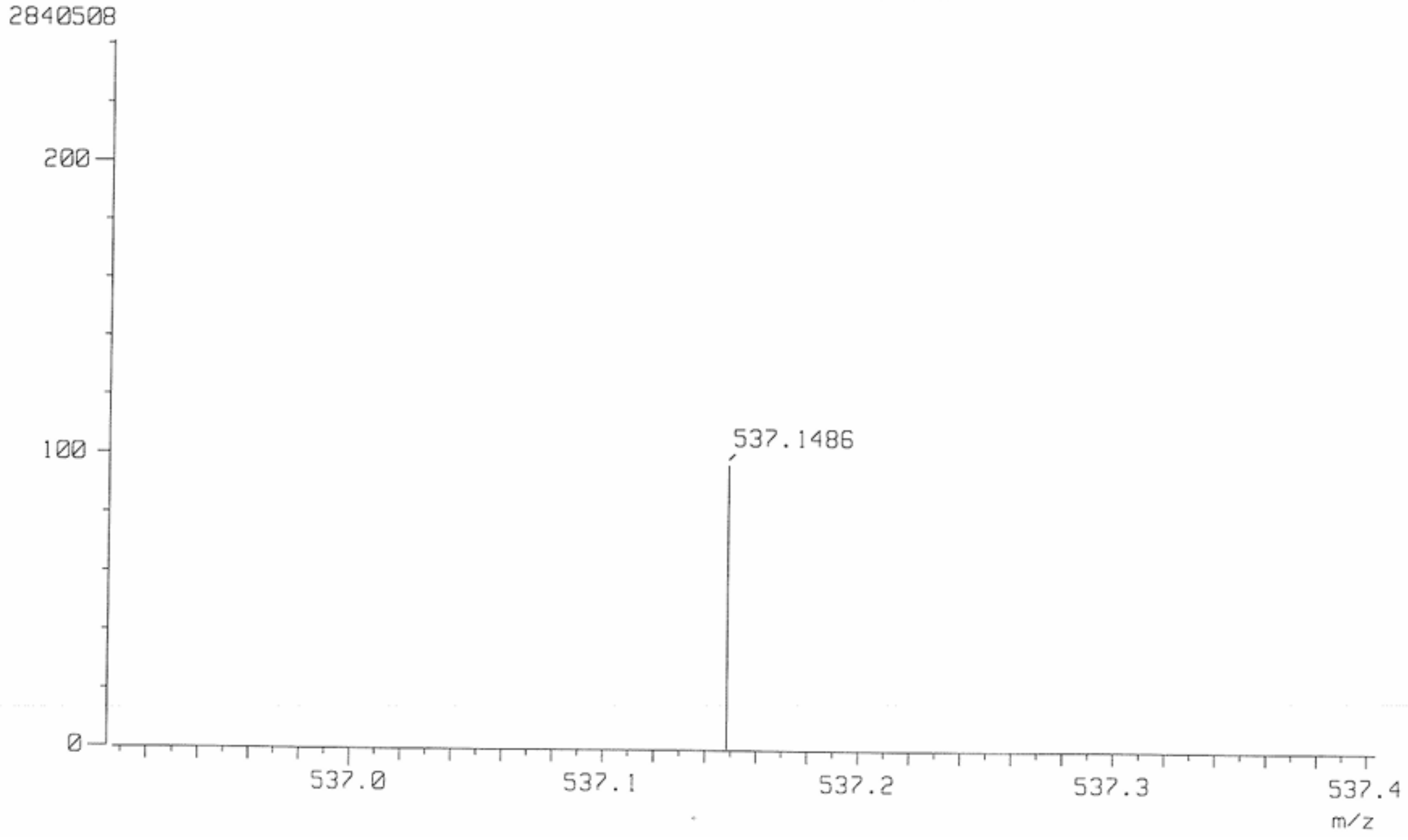

Supplement: Supplementary file 1 [file RA-OLF-D6RA09632H-s001.pdf]
